# sSPhos: A General Ligand for Enantioselective Arylative Phenol Dearomatization via Electrostatically-Directed Palladium Catalysis

**DOI:** 10.1021/jacs.3c10663

**Published:** 2023-11-16

**Authors:** Max Kadarauch, David M. Whalley, Robert J. Phipps

**Affiliations:** Yusuf Hamied Department of Chemistry, University of Cambridge, Lensfield Road, Cambridge, CB2 1EW, United Kingdom

## Abstract

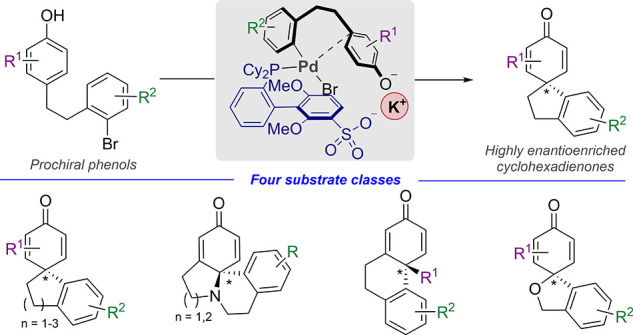

Arylative phenol dearomatization affords complex, cyclohexanone-based scaffolds from simple starting materials, and asymmetric versions allow access to valuable enantioenriched structures. However, bespoke chiral ligands must typically be identified for each new scaffold variation. We have addressed this limitation by applying the concept of electrostatically-directed palladium catalysis whereby the chiral sulfonated ligand sSPhos engages in electrostatic interactions with a phenolate substrate via its associated alkali metal cation. This approach allows access to highly enantioenriched spirocyclohexadienones, a process originally reported by Buchwald and co-workers in a predominantly racemic manner. In addition, sSPhos is proficient at forming two other distinct scaffolds, which had previously required fundamentally different chiral ligands, as well as a novel oxygen-linked scaffold. We envisage that the broad generality displayed by sSPhos will facilitate the expansion of this important reaction type and highlight the potential of this unusual design principle, which harnesses attractive electrostatic interactions.

Phenol dearomatization is exceptionally useful for building up three-dimensional molecular complexity.^1^ Although the energetic barriers can be high, the products possess versatile functionality and often a new stereocenter. Dearomatization of phenols is typically more challenging than naphthols, indoles, pyrroles, and the like because of lower electron density. While many methods rely on highly electrophilic reagents, transition metal catalysis has recently expanded the breadth of accessible transformations.^2^ This has enabled arylative dearomatizations during which a new arene substituent is introduced during the dearomatizing event (early methods for arylative dearomatization relying on stoichiometric lead,^3^ bismuth,^4^ and iodine^5^ arylating reagents possessed various limitations). A pioneering advance was reported by Buchwald and co-workers in 2011 with the palladium-catalyzed intramolecular arylation of phenols, which produced spirocyclohexadienones bearing all-carbon quaternary centers (Figure 1A, upper).^6^ The scope was explored using an achiral phosphine ligand (**L1**) but it included two preliminary enantioselective results, one phenol and one naphthol. For the phenol example, **L2** allowed 81% ee in the intramolecular dearomatization of **1a** (Figure 1A, lower). Since Buchwald’s original report, a number of important developments on palladium-catalyzed arylative phenol dearomatization to form different scaffolds, from the groups of You^7^ and Tang,^8^ have been made.^9^ This includes asymmetric variants using TADDOL-derived chiral phosphoramidites^7b^ and *P*-chiral biaryl monophosphine ligands,^8a^ respectively. It is evident that success for a new substrate class requires extensive ligand evaluation and tailoring, a feature which hinders more rapid development and widespread use of this important reaction type.^10^

**Figure 1:**
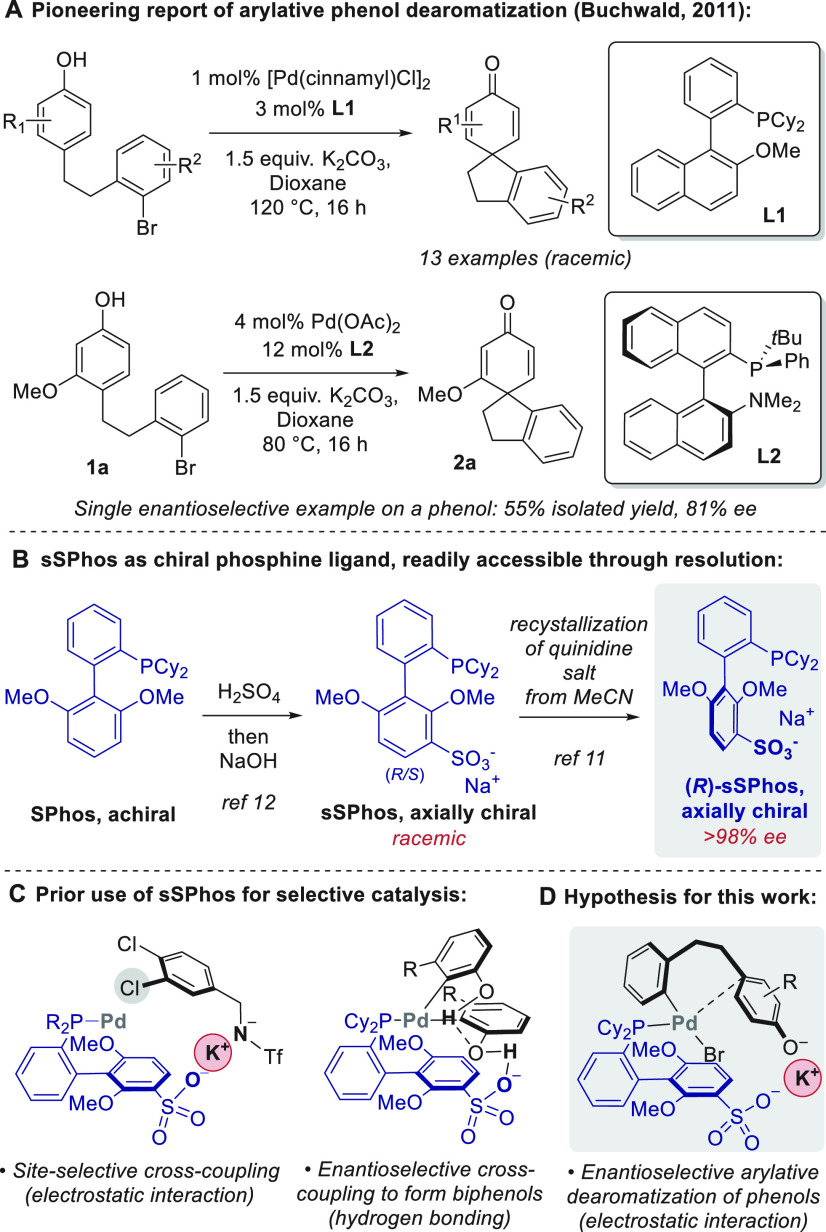
Previous arylative phenol dearomatization and use of sSPhos as a bifunctional ligand.

We recently reported the use of enantiopure sSPhos as an unexplored chiral, bifunctional phosphine ligand, which can be readily obtained by diastereoselective recrystallization of (*rac*)-sSPhos as its quinidinium salt (Figure 1B).^11^ Originally reported by Anderson and Buchwald as a water-soluble ligand for cross-coupling,^12^ we initially utilized (*rac*)-sSPhos for control of site selectivity in the cross-coupling of polyhalogenated arenes. Therein, we introduced the concept of electrostatically directed palladium catalysis, whereby an anionic ligand interacts with an anionic substrate via a bridging alkali metal cation through electrostatic interactions (Figure 1C, left).^13,14^ We subsequently found enantiopure sSPhos to be highly proficient in controlling enantioselectivity in Suzuki–Miyaura couplings to form 2,2′-biphenols, an outcome we tentatively attributed to an organizing network of hydrogen bonds between the ligand sulfonate group and the phenolic hydroxyls on the coupling partners (Figure 1C, right).^11^ On the basis of these precedents, we hypothesized that enantiopure sSPhos might be an effective ligand for enantiocontrol in the Buchwald arylative dearomatization reaction. Mechanistically, in the presence of a strong base, it is likely that phenolate formation occurs and that the subsequent palladation of this phenolate may be selectivity-determining.^7b,15^ We envisaged that an attractive electrostatic interaction might occur between the alkali metal cation of the phenolate and the sulfonate group of the ligand, akin to those we invoked in our prior work, thereby providing organization in a chiral environment (Figure 1D). More broadly, we were optimistic that, by exploiting electrostatic interactions with phenolate intermediates, sSPhos might constitute a generally applicable ligand for enantioselective, Pd-catalyzed arylative phenol deromatization across a diverse range of scaffolds.^16^

We began with conditions similar to those identified in Buchwald’s study^6^ by using [Pd(cinnamyl)Cl]_2_ and K_2_CO_3_ in dioxane at 110 °C and (*R*)-sSPhos as the ligand (Table 1, entry 1). Pleasingly, spirocycle **2a** was formed in 76% yield with encouraging enantioselectivity. An evaluation of palladium sources (entries 2–4) revealed that Pd_2_dba_3_ afforded significant improvement (63% ee), as did switching the base to KOH (entry 5, 84%). Aromatic solvents provided no improvement (entries 6 and 7), but addition of water as a cosolvent increased yield and enantioselectivity in all cases (entries 8–10).^17^ A PhMe:H_2_O biphasic mixture proved to be optimal and afforded **2a** in 98% yield and 92% ee with the absolute configuration determined by X-ray diffraction (entry 10). Reactivity remained excellent at 90 °C but with no improvement in ee (entry 11). Various group 1 metal hydroxides were tested, which gave very similar enantioselectivity outcomes (entries 12–14).^18^

**Table 1:**
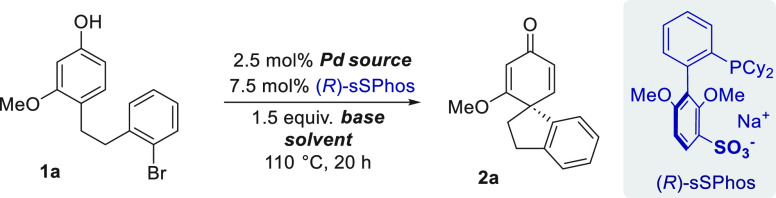
Reaction Optimization

entry	Pd source	base	solvent	yield (%)^a^	ee (%)^b^
1	[PdCl(cinnamyl)]_2_	K_2_CO_3_	dioxane	76	44
2	[PdCl(allyl)]_2_	K_2_CO_3_	dioxane	48	34
3	Pd(OAc)_2_	K_2_CO_3_	dioxane	13	27
4	Pd_2_dba_3_	K_2_CO_3_	dioxane	81	63
5	Pd_2_dba_3_	KOH	dioxane	48	84
6	Pd_2_dba_3_	KOH	PhCF_3_	41	83
7	Pd_2_dba_3_	KOH	PhMe	60	83
8	Pd_2_dba_3_	KOH	dioxane/H_2_O (10:1)	66	89
9	Pd_2_dba_3_	KOH	PhCF_3_/H_2_O (10:1)	91	91
10	Pd_2_dba_3_	KOH	PhMe/H_2_O (10:1)	99 (98)	92
11^c^	Pd_2_dba_3_	KOH	PhMe/H_2_O (10:1)	95	91
12	Pd_2_dba_3_	LiOH	PhMe/H_2_O (10:1)	84	95
13	Pd_2_dba_3_	NaOH	PhMe/H_2_O (10:1)	98	92
14	Pd_2_dba_3_	CsOH	PhMe/H_2_O (10:1)	87	95

a Yields determined by ^1^H NMR with reference to a dibromomethane internal standard. Value in parentheses refers to isolated yield.

b ee determined by SFC analysis of the crude reaction mixture, except entry 10.

c Reaction temperature 90 °C.

We evaluated the scope of the dearomatization and were pleased to find that phenols substituted with methyl and phenyl at the *meta* position also gave a high ee (Scheme 1, **2b**, **2c**). Conversion to **2c** was low, likely because of hindrance at the forming spirocyclic stereocenter. We were curious as to whether substitution at the phenol *ortho* position would give good outcomes with the facial differentiation being further from the forming stereocenter. Indeed, high enantioselectivities were maintained for these substrates encompassing phenyl (**2d**), methoxy (**2e**), and methyl (**2f**) substituents. The limits of electronic tolerance on the phenol are displayed by an *ortho*-fluoro substitution: **2g** was obtained in low yield but still remarkably high enantioselectivity given the small size of the differentiating substituent.^19^ Substitution at two adjacent positions of the phenol ring, including a naphthol, also worked well (**2h**, **2i**), and the tether between the two arenes was extended to afford tetralin derivative **2j**. Further extension leading to a seven-membered ring also delivered very high enantioselectivity (**2k**), although the low yield reflected the present reactivity limit. Substitution of the lower ring gave good outcomes with both electron-poor (**2l**) and -rich (**2m**) examples. A substrate bearing both chloride and bromide reacted selectivity at the bromide (**2n**, 96% ee). A Boc-protected amine was tolerated (**2o**), as was an ester (**2p**). A methyl adjacent to the bromide on the lower ring gave a significant ee reduction (**2q**). Finally, fluorine-containing **2r** and **2s** were obtained smoothly. When the reaction was scaled to 1 mmol, a small increase in enantioselectivity for **2r** was observed (Scheme 1B). This led us to assess a lower 2 mol % loading of Pd (3 mol % sSPhos) at this larger scale with excellent results still obtained for **2l**.

**Scheme 1:**
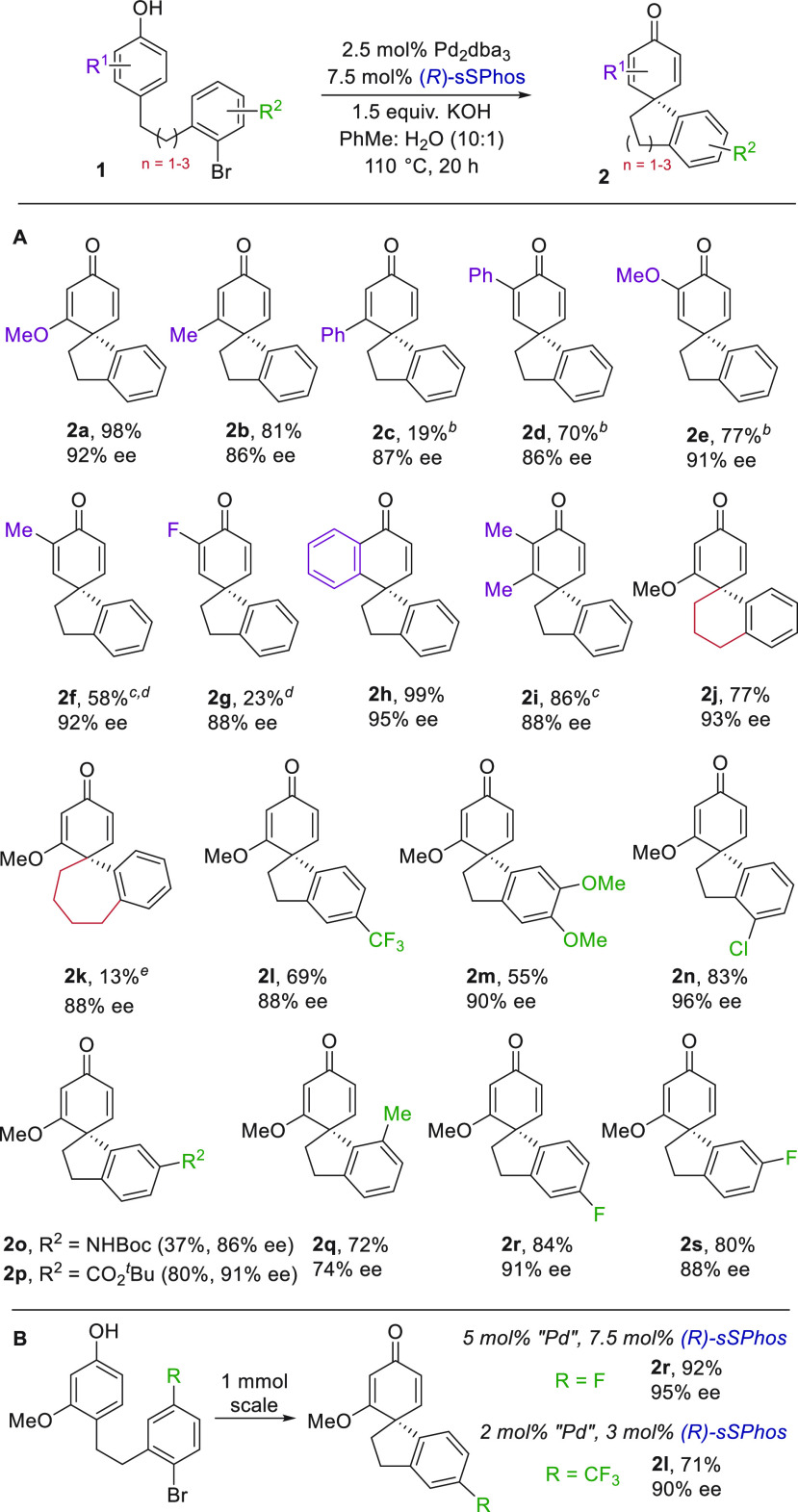
Scope of Arylative Phenol Dearomatization on Substrates Related to **1a** Yields are isolated; ee values determined by SFC. 48 h reaction time. Used 3.0 equiv of KOH. 5 mol % Pd_2_dba_3_ and 15 mol % (*R*)-sSPhos for 48 h. Reaction time of 92 h.

Having demonstrated the effectiveness of sSPhos on Buchwald’s original arylative dearomatization scaffold, we sought to evaluate how generally applicable it might be. We next targeted arylative dearomatization of the *para*-aminophenol class of substrates reported by You and co-workers racemically in 2014^7a^ and enantioselectively in 2020 (Scheme 2).^7b^ These substrates are notable as they map directly onto the skeleton of the Erythrina alkaloids.^1b,1d,2b^ Excellent results could be achieved with only 2.5 mol % Pd to give dearomatized **4a** in good yield and excellent enantioselectivity. Usefully, an aryl chloride could also be used as a the starting material. A larger ring in the heterocyclic starting material gave excellent results (**4b**), and we evaluated several substituents of varying electronic character on the lower ring (**4c**–**4f**). Dimethoxy-substituted **4g**, upon hydrogenation, leads directly to (−)-3-demethoxyerythratidinone (**5**), as previously demonstrated by You and co-workers.^7^

**Scheme 2:**
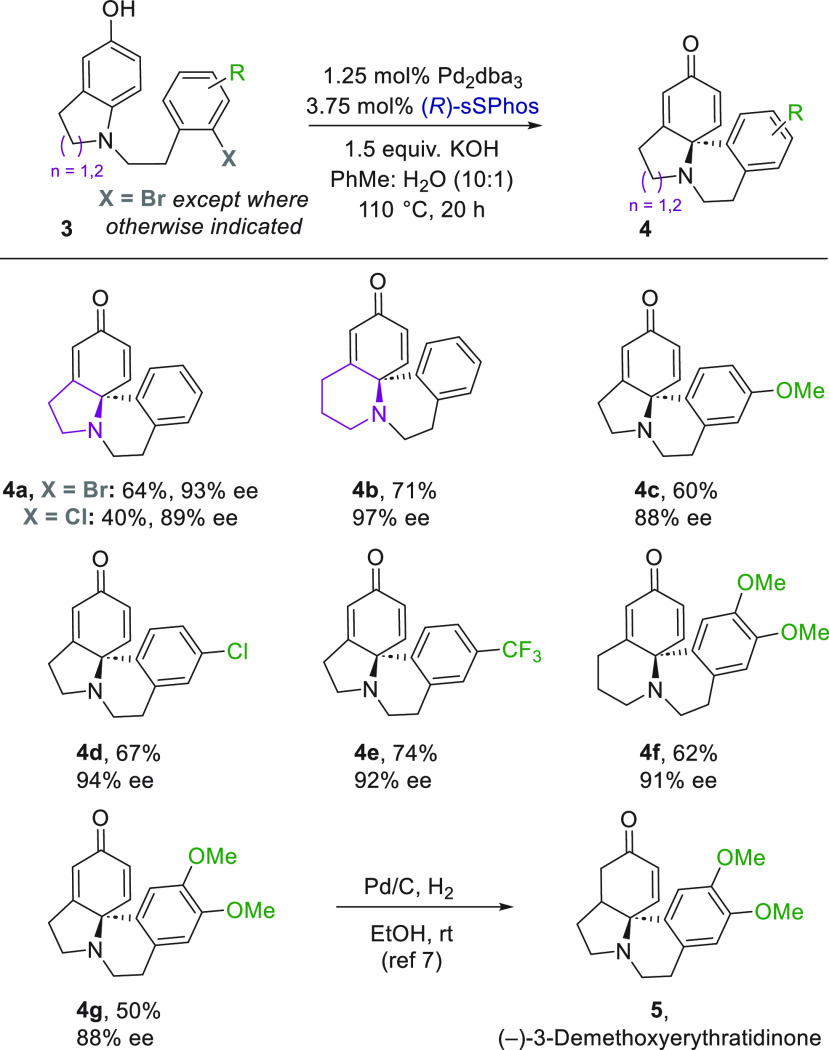
Enantioselective Arylative Dearomatization of *para*-Amino Phenols

To further test the generality of sSPhos, we benchmarked it on a third distinct substrate class, previously reported by Tang and co-workers who elegantly applied it to natural product synthesis (Scheme 3).^8a−8c,8e^ With little modification to the conditions, excellent results could be obtained for chiral phenanthrenone derivatives related to **7a**. Several analogues were demonstrated by varying the phenol *para* substituent (**7b**), as well as the lower ring substituent (**7c**–**7e**).

**Scheme 3:**
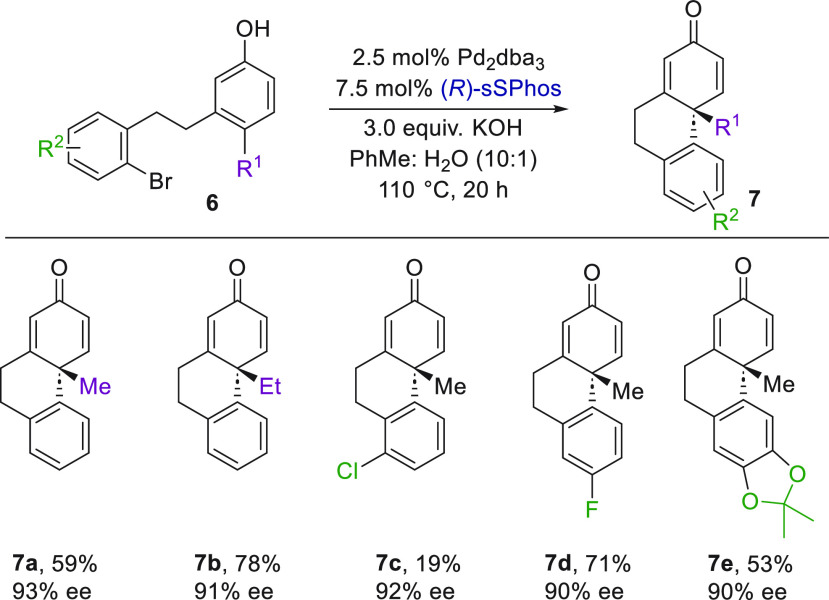
Arylative Dearomatization to Give Chiral Phenanthrenone Derivatives

The substrates so far have generated products possessing all-carbon (Schemes 1 and 3) and α-tertiary amine (Scheme 2) quaternary stereocenters. We questioned whether this might be extended to *O*-linked substrates to form α-tertiary ethers at the spirocyclic stereocenter. Such motifs have not, to the best of our knowledge, been formed so far using arylative phenoldearomatization, even racemically. The resulting scaffold features in a number of natural products, including members of the urnucratin^20^ and kadsulignan^21^ families and parvifloral F.^22^ Pleasingly, methyl-substituted **9a** and methoxy-substituted **9b** were obtained in 82% and 83% ee and **9c**, which bears a chloride on the lower ring and methyls on the upper, was obtained in 93% ee (Scheme 4). The low to moderate yields are attributed to decomposition of the electron-rich starting material under the reaction conditions. Nevertheless, these results underline the generality of sSPhos as a chiral ligand for arylative phenol dearomatization, in the context of an as-yet-unexplored scaffold.

**Scheme 4:**
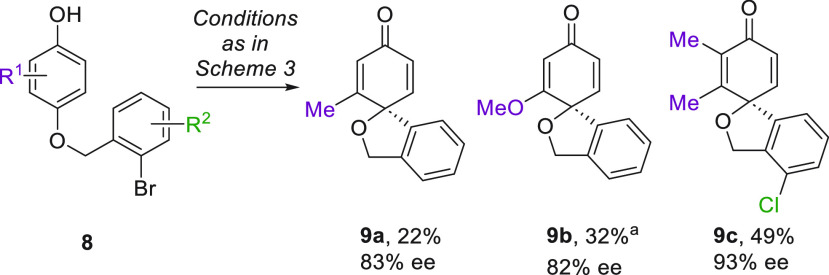
Extension to Spiroheterocyclic Scaffolds Incorporating Oxygen NaOH (3.0 equiv) used in place of KOH.

We sought to probe the interactions responsible for the effectiveness of sSPhos. The anticipated p*K*_a_ difference between a phenol and KOH would suggest that the potassium phenolate salt is formed under the reaction conditions, a scenario supported by NMR studies (see the Supporting Information). Phenolate formation would mean it is unlikely that ligand–substrate hydrogen bonding is occurring. We carried out the reaction in anhydrous toluene by comparing the standard phenol with a TMS-protected variant (Scheme 5A). The identical ee values provide further evidence against hydrogen bonding playing a role in selectivity because the latter conditions feature no feasible proton source. Furthermore, use of the preformed potassium phenolate salt as the substrate returned the ee to the exact value (92%) obtained when running the reaction under the optimized conditions with water. We speculate that the presence of water in the optimized conditions assists in rapid potassium phenolate formation, which is crucial for high yield and enantioselectivity. We next sought evidence for the proposed electrostatic interaction involving a bridging metal cation (Figure 1D). During optimization, no significant variation in enantioselectivity had been observed between the various alkali metal cations when they were evaluated in toluene/water (Table 1). However, differences between them were observed in dioxane, which suggests possible involvement in the selectivity-determining step.^18^ Crucially, replacement of the alkali metal cation with either tetrabutylammonium or tetrabutylphosphonium was found to be detrimental to both yield and ee, thereby suggesting that favorable organization in the enantiodetermining transition state cannot be maintained with these bulky cations (Scheme 5B). Accordingly, reduction of the length of the alkyl chains in tetramethylammonium hydroxide largely restored both metrics.

**Scheme 5:**
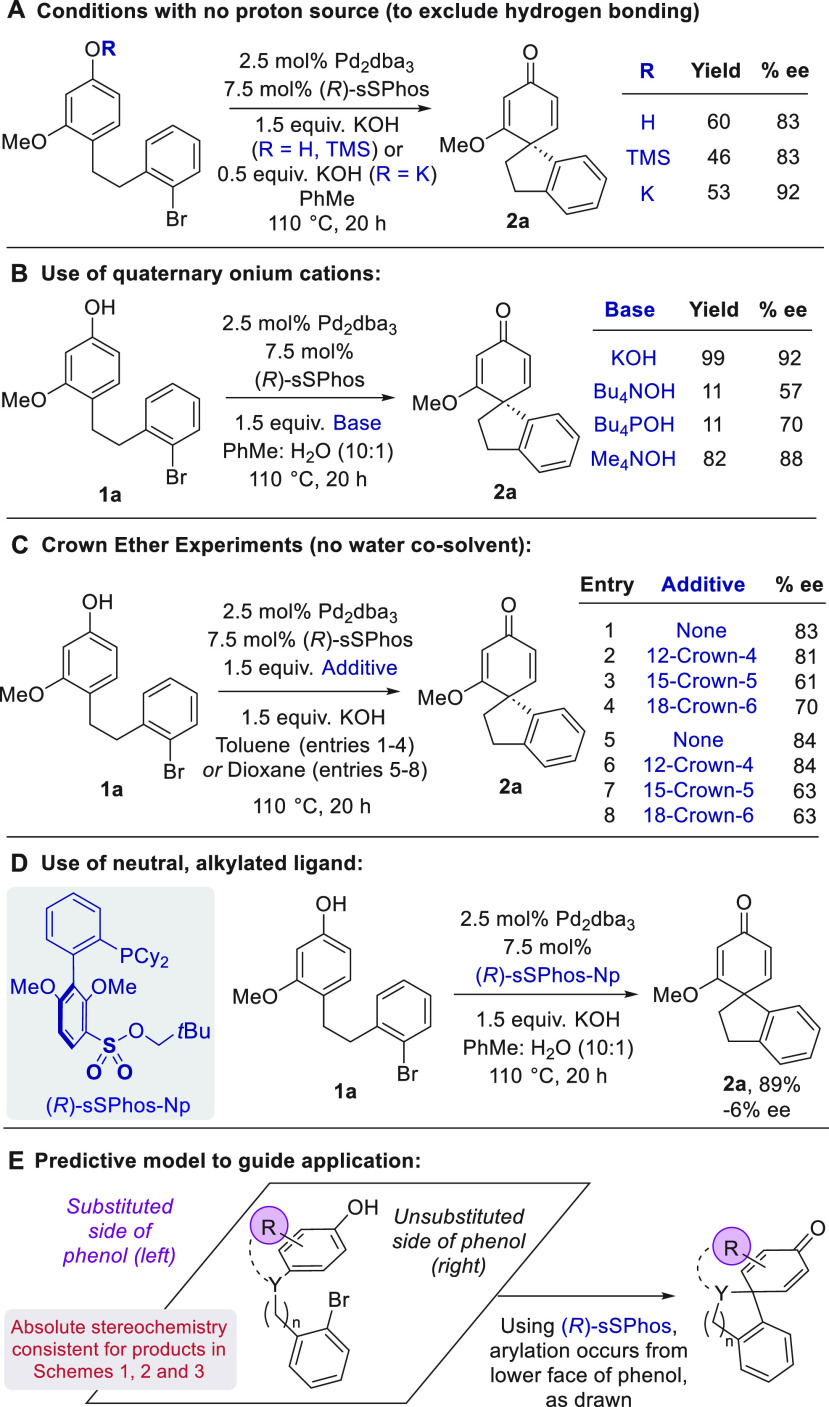
Control Experiments to Probe Ligand–Substrate Interactions and Predictive Model

We further probed the importance of the alkali metal cation by the addition of stoichiometric crown ethers of varying size (Scheme 5C).^23^ In toluene, almost no effect on ee was observed by the addition of 12-crown-4, as expected, given its small size relative to K^+^ (entry 2 vs 1).^24^ However, 15-crown-5 and 18-crown-6 both gave reduced ee, which suggests that binding to the cation disrupts the substrate–ligand organization to some extent (entries 3 and 4). A similar outcome was observed in dioxane (entries 5–8). Finally, we sought to remove the charge from the ligand altogether to rule out the possibility that sSPhos might be exerting enantiocontrol through simple steric repulsion. Accordingly, a neopentyl sulfonate ester derivative of the ligand gave only −6% ee (Scheme 5D). The absolute stereochemistry of the products from Schemes 1,^25^^2^,^7b^ and 3(8a) could all be reliably determined. In all cases, use of (*R*)-sSPhos is consistent with arylation occurring from the lower face of the phenol when it is depicted with its substituted side to the left and the unsubstituted to the right (Scheme 5E).

In summary, enantiopure sSPhos, easily obtained via resolution, is an extremely general chiral ligand for the Pd-catalyzed intramolecular arylative dearomatization of phenols. Using Buchwald’s pioneering report, which afforded spirocyclohexadienones bearing all-carbon quaternary centers in a predominatly racemic manner, as a forum for demonstrating its effectiveness, we subsequently extended this to two other substrate classes. We also report several oxygen-linked substrates, which have not to date been explored, that give rise to spiroheterocyclic α-tertiary ethers. These results, combined with our prior work applying sSPhos to asymmetric biphenol synthesis, underscore the remarkable ability of sSPhos to exert enantiocontrol in palladium-catalyzed reactions involving versatile phenolic substrates.

## Supporting Information Available

Additional optimization, full experimental details, and characterization data for compounds (PDF)
