# Supplementary material for: sSPhos: A General Ligand for Enantioselective Arylative Phenol Dearomatization via Electrostatically-Directed Palladium Catalysis
Source: J Am Chem Soc. 2023 Nov 16;145(47):25553–8. doi: 10.1021/jacs.3c10663 (PMC10690801; doi:10.1021/jacs.3c10663)

Supporting Information

sSPhos: A General Ligand for Enantioselective Arylative Phenol Dearomatization via Electrostatically-Directed Palladium Catalysis

Max Kadarauch, David M. Whalley and Robert J. Phipps*

Yusuf Hamied Department of Chemistry, University of Cambridge, Lensfield Road, Cambridge, CB2 1EW, United Kingdom.

Table of Contents

General Experimental **3**

Extended Reaction Optimization Data **5**

Solvent Screen with K_2CO_3 as the Base 5
Investigation of Bases with 1,4-Dioxane or PhMe as the Solvent 5
Investigation of Bases with PhMe: H_2O as the Solvent 6
Investigation of a $Pd(dba)_2$ Palladium Pre-catalyst 6
Investigation of Catalyst Loadings and Ratio of Pd: Ligand under Final Optimized Conditions 7
Effect of Base Loadings on Yield and Enantioselectivity 8

Unsuccessful Substrates **8**

Experiments to Probe Deprotonation of Phenol 1a Under Reaction Conditions **9**

Extended Data for Crown Ether Control Experiments **11**

Crown Ether Experiments with KOH as the Base 11
Crown Ether Experiments with NaOH as the Base 11

Determination of Product Absolute Stereochemistry **12**

Synthesis of Starting Materials in Scheme 1 .... **13**

General Procedure A: Phosphonium Salt Synthesis 13
General Route 1: Synthesis of Starting Materials via Benzyl-protected Phenols 13
General Procedure B: Wittig Alkene Synthesis 13
General Procedure C: Alkene Hydrogenation 14
General Route 2: Synthesis of Starting Materials via Methoxy-protected Phenols 14
General Procedure D: Deprotection of Methoxy-protected Phenols 14
General Route 3: Synthesis of Starting Materials via Unprotected Phenols 15
General Procedure E: Diimide Reduction of Alkenes 15

Synthesis of Starting Materials in Scheme 2 .... **43**

General Procedure F: Synthesis of *para*–Amino Phenol Starting Materials *via* Reductive Amination 43
General Procedure G: Wittig Homologation of Substituted 2-Bromobenzaldehydes 44

Synthesis of Starting Materials in Scheme 3 .... **53**

General Route 4: Synthesis of *meta*-Tethered Substrates via Methoxy-protected Phenols 53

Synthesis of Starting Materials in Scheme 4 .... **59**

General Procedure H: Synthesis of *O*–Linked Phenols *via* Alkylation with 2-Bromobenzyl Bromide 59

Synthesis of Starting Materials in Scheme 5 .... **62**

Synthesis of Alkylated Ligand **64**

Characterization of Products in Scheme 1 .... **67**

General Procedure J: Palladium/(*R*)–sSPhos-Catalyzed Dearomatization of Phenols 67

Characterization of Products in Scheme 2 .... **83**

General Procedure K: Palladium/(*R*)–sSPhos-Catalyzed Dearomatization of *para*-Amino Phenols ... 83

Characterization of Products in Scheme 3 .... **88**

General Procedure L: Palladium/(*R*)–sSPhos-Catalyzed Dearomatization of *meta*-Tethered Phenols 88

Characterization of Products in Scheme 4 .... **92**

General Procedure M: Palladium/(*R*)–sSPhos-Catalyzed Dearomatization of Oxygen-linked Phenols 92

References **95**

Chiral SFC/HPLC Traces **97**

NMR Spectra **137**

General Experimental

Solvents: THF, MeOH, CH_2Cl_2, and PhMe were purified by distillation on site under inert atmosphere via the following processes. THF was pre-dried over sodium wire and then distilled from calcium hydride and lithium aluminum hydride. MeOH, CH_2Cl_2, and PhMe were distilled from calcium hydride. H_2O was sparged with nitrogen for 30 minutes prior to use.

Reagents: All reagents were used as supplied from commercial sources with no further purification. KOH and NaOH were finely ground using a pestle and mortar. (*R*)–sSPhos was prepared by resolution of (*rac*)–sSPhos by preparative SFC, according to our previous publication.[1] Alternatively, (*R*)–sSPhos can also be obtained *via* recrystallization of diastereomeric quinidine salts, according to our previous publication.[1]

Chromatography: Flash column chromatography was performed using silica gel 60 (0.040-0.063 μM) from Breckland scientific supplies or silica gel 60A (40-63 μM) from Fluorochem. Crude compounds were dried directly onto silica gel and then loaded onto a pre-equilibrated silica column eluting with the solvent system specified under a positive pressure of air. Thin layer chromatography (TLC) was performed using 0.25 mm E. Merck silica plates (60F-254). The plates were visualized using ultraviolet radiation (254 nm) or a potassium permanganate stain where appropriate.

Reactions: Optimization experiments were carried out on a 0.10 mmol scale in 4 mL 13 mm crimp ring vials. For the scope examples, the reactions were carried out in either 4 mL crimp vials or 0.5 – 2 mL Biotage microwave vials.

Data collection: ^{1}H NMR spectra were recorded on 400 MHz QNP cryoprobe, 400 MHz AVIII HD smart probe, 400 MHz Advance III HD, 400 MHz Neo Prodigy, 500 MHz DCH Cryoprobe, 600 MHz Advance BBI and 700 MHz TXO Cryoprobe spectrometers. The chemical shifts, reported in parts per million (δ ppm) were recorded relative to the residual undeuterated solvent ($CDCl_3$: 7.26 ppm, MeOD: 3.31 ppm, Acetone-d_6: 2.05 ppm, CD_3CN: 1.96 ppm, DMSO-d_6: 2.50 ppm, toluene-d_6: 2.09 ppm). Multiplicity is recorded as follows: s = singlet, d = doublet, t = triplet, q = quartet, pent = pentet, sext = sextet, m = multiplet, br = broad with associated combinations. Coupling constant (*J*, Hz) and peak integrations (nH) are also reported. ^{13}C NMR spectra were recorded on the same machines with complete proton decoupling. The chemical shifts, reported in parts per million (δ ppm) were recorded relative to the residual undeuterated solvent ($CDCl_3$: 77.16 ppm, MeOD: 49.00 ppm, Acetone-d_6: 29.84, CD_3CN: 118.26, DMSO-

d_6: 39.52). ^{19}F NMR spectra were recorded on 400 MHz QNP cryoprobe, 400 MHz AVIII HD smart probe and 400 MHz Advance III HD spectrometers.

Chiral SFC Analysis: Performed on a Waters ACQUITY UPC2 System with DAICEL CHIRALPAK IA, IE, IG, IH, or IK columns (4.6 x 250 mm, 3.0 µm) in a mixed solvent system of supercritical CO_2 and MeOH. A system backpressure of 138 bar was used in all cases.

Chiral HPLC analysis: performed using a Waters ARC system with YMC CHIRAL ART SA (4.6 x 250 mm, 3.0 µm) or DAICEL CHIRALPAK AD-H and IG columns (4.6 x 250 mm, 3.0 µm) in a mixed solvent system of hexane and ^{i}PrOH.

High Resolution Mass Spectrometry (HRMS): Recorded on a Waters Micromass LCT Premier, a Waters Xevo G2-S QTOF, a Waters Synapt G2-Si, a Waters Vion IMS QTOF and AGILENT 6230 LC/TOF at the Department of Chemistry at the University of Cambridge. The ionization method is noted as either positive or negative electrospray ionisation (+/–ESI). Measured values are reported to 4 decimal places and are within ± 5 ppm of the calculated value. The calculated values are based on the most abundant isotope unless otherwise stated in the chemical formula.

Optical Rotations: measured in $CHCl_3$ on a Perkin Elmer 343 Polarimeter using a sodium lamp (λ = 589 nm, D-line). $[\alpha]_D$ values are reported at 25.0 °C in degrees mL g^{-1} dm^{-1} with concentration (c) in cg mL^{-1}.

X-Ray Crystallography: Data collection and analysis was performed by Dr Andrew Bond (University of Cambridge)

Racemic Reactions: Reactions to obtain racemic SFC or HPLC traces were run with (*rac*)-sSPhos.

Extended Reaction Optimization Data

Solvent Screen with K_2CO_3 as the Base

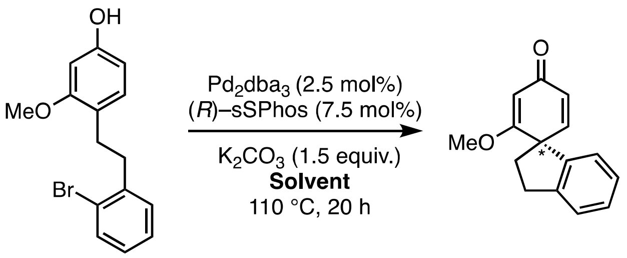

Entry	Solvent	Yield/ %[a]	ee/ %[b]
**1**	1,4-Dioxane	81	63
**2**[c]	1,4-Dioxane	63	59
**3**	PhMe	51	52
**4**	$PhCF_3$	54	74
**5**	1,4-Dioxane: H_2O (10:1)	91	85
**6**	PhMe: H_2O (10:1)	97	66
**7**[c]	PhMe: H_2O (10:1)	41	63
**8**	$PhCF_3$: H_2O (10:1)	65	70

[a] Yields determined by ^{1}H NMR with reference to a dibromomethane internal standard. [b] *ee* determined by chiral SFC analysis of the crude reaction mixture. [c] Carried out at 90 °C.

Investigation of Bases with 1,4-Dioxane or PhMe as the Solvent

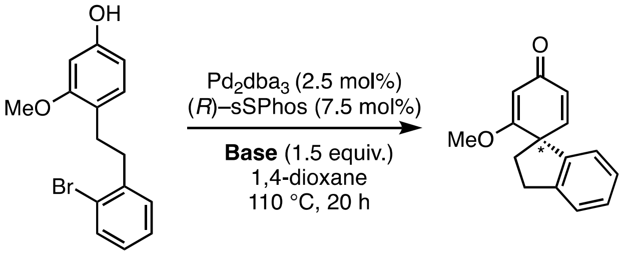

Entry	Base	Yield/ %[a]	ee/ %[b]
**1**	K_2CO_3	81	63
**2**	Li_2CO_3	trace	N.D.
**3**	Na_2CO_3	trace	N.D.
**4**	Rb_2CO_3	96	81
**5**	Cs_2CO_3	58	90
**6**	K_3PO_4	96	83

**7**	LiOH	18	67
**8**	NaOH	73	89
**9**	CsOH	49	89
**10**[c]	CsOH	48	92
**11**	KOH	48	84

[a] Yields determined by ^{1}H NMR with reference to a dibromomethane internal standard. [b]*ee* determined by chiral SFC analysis of the crude reaction mixture. [c] Reaction conducted with PhMe as the solvent.

Investigation of Bases with PhMe: H_2O as the Solvent

Optimal results were obtained with KOH, LiOH, NaOH, and CsOH, which are reported in Table 1 (entries 10, 12, 13, 14) of the manuscript. The results obtained with K_2CO_3, Rb_2CO_3, Cs_2CO_3, and K_3PO_4 are presented below.

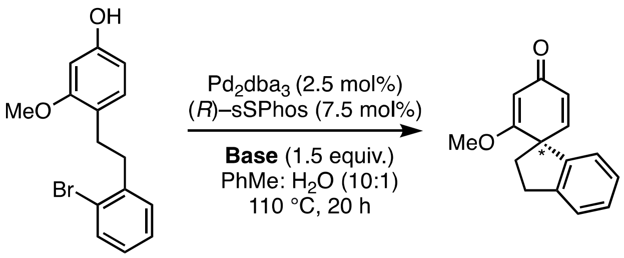

1a **2a**

Entry	Base	Yield/ %[a]	ee/ %[b]
**1**	K_2CO_3	97	66
**2**	Rb_2CO_3	87	77
**3**	Cs_2CO_3	89	79
**4**	K_3PO_4	99	77

[a] Yields determined by ^{1}H NMR with reference to a dibromomethane internal standard. [b]*ee* determined by chiral SFC analysis of the crude reaction mixture.

Investigation of a Pd(dba)$_2$ Palladium Pre-catalyst

Under the conditions deployed in entries 1-4 of table 1, Pd(dba)$_2$ afforded a higher enantioselectivity than Pd_2dba_3.

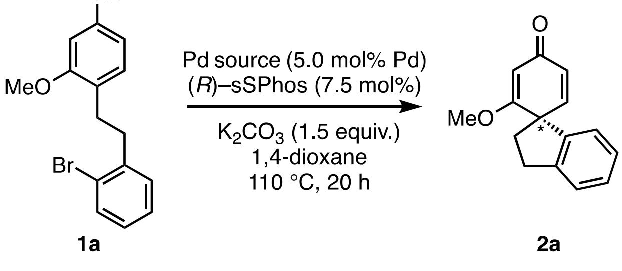

1a **2a**

Entry	Pd source	Yield/ %[a]	ee/ %[b]
**1**	Pd_2dba_3	81	63
**2**	$Pd(dba)_2$	74	70

[a] Yields determined by ^1H NMR with reference to a dibromomethane internal standard. [b]*ee* determined by chiral SFC analysis of the crude reaction mixture.

However, when $Pd(dba)_2$ was investigated under the final optimized conditions, Pd_2dba_3 performed slightly better (c.f. Table 1, entry 10: 99% NMR yield, 92% ee).

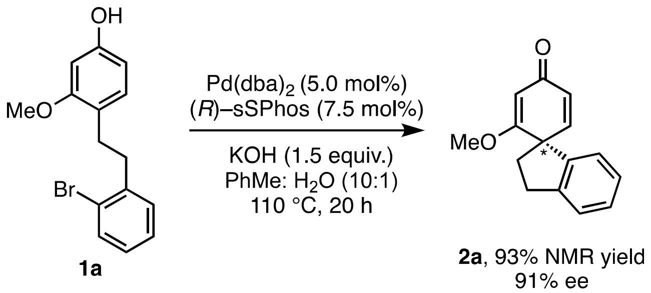

1a **2a**, 93% NMR yield 91% ee

Investigation of Catalyst Loadings and Ratio of Pd: Ligand under Final Optimized Conditions

Reducing the overall loadings of catalyst caused a slight decrease in yield and enantioselectivity (entries 1 and 2). Varying the Pd: ligand ratio had a negligible effect (entries 1, 3, and 4).

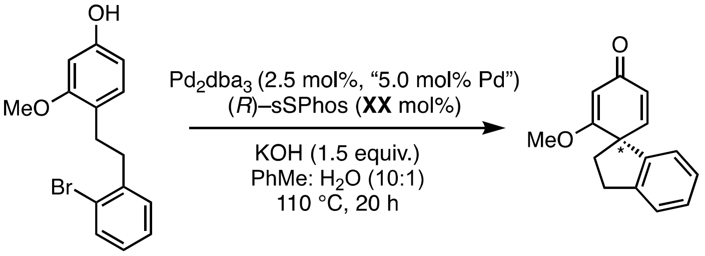

1a **2a**

Entry	Pd:L	Yield/ %[a]	ee/ %[b]
**1**	**1:1.5**	**99 (98)**	**92**
**2**[c]	1:1.5	94 (91)	89
**3**	1:2	99	92
**4**	1:1	99	91

[a] Yields determined by ^1H NMR with reference to a dibromomethane internal standard. Values in parentheses corresponds to the isolated sample. [b]*ee* determined by chiral SFC analysis of the crude reaction mixture, apart from entries 1 and 2. [c] Reaction conducted with "2.5 mol% Pd" and 3.75 mol% (*R*)–sSPhos

Effect of Base Loadings on Yield and Enantioselectivity

For several substrates, it was found that doubling loadings of base from 1.5 equiv. to 3.0 equiv. increased enantioselectivity, but decreased yield. This was observed to varying degrees on different substrates. Selected examples are presented below.

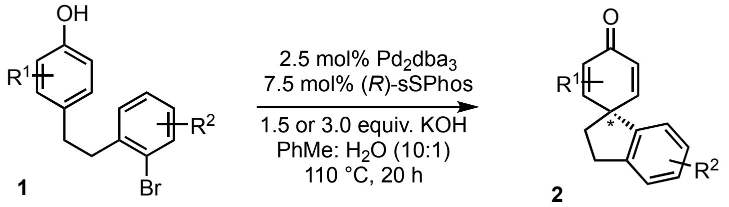

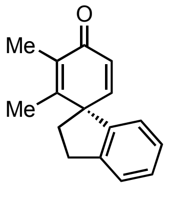

2i
1.5 equiv. KOH: 89%, 82% ee
3.0 equiv. KOH: 86%, 88% ee

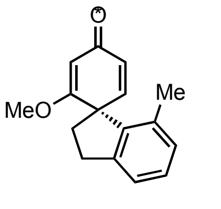

2q
1.5 equiv. KOH: 72%, 74% ee
3.0 equiv. KOH: 35%, 75% ee

Unsuccessful Substrates

A phenol containing an electron-withdrawing chloride substituent (**S1**), a catechol-derived starting material bearing two hydroxy substituents (**S2**), and a TIPS-protected starting material (**S3**) were all unsuccessful when tested under the following reaction conditions.

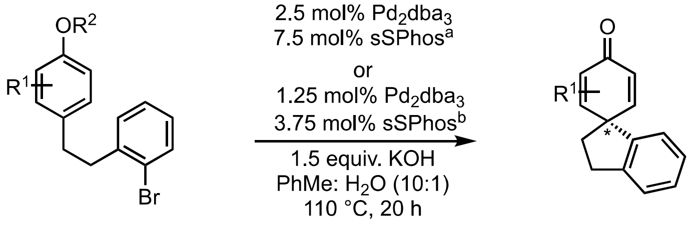

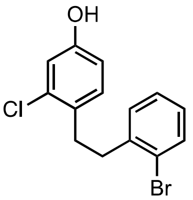

S1, < 5% NMR yield, 61% recovered starting material[a]

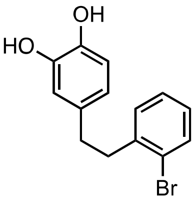

S2, no product detected, starting material degraded[b]

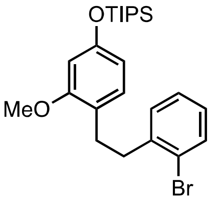

S3, trace product detected, 88% recovered starting material[a]

Yields determined by ^1H NMR with reference to a dibromomethane internal standard

Experiments to Probe Deprotonation of Phenol 1a Under Reaction Conditions

Phenol **1a** (30.7 mg, 0.100 mmol, 1.00 equiv.) and a 1,3,5-tri-*tert*-butylbenzene internal standard (24.6 mg, 0.100 mmol, 1.00 equiv.) were dissolved in d^8-PhMe (0.5 ml), and a ^{1}H NMR spectrum of the mixture was obtained. The solution was poured into a vial containing a magnetic stirrer bar, and KOH (8.4 mg, 0.15 mmol, 1.5 equiv.) was added. The vial was sealed, evacuated and backfilled with N_2 (3×), and heated to 110 °C and 900 rpm for 1 h to emulate the reaction conditions. It was noted that the resulting reaction mixture was homogeneous. The reaction was cooled, and a second ^{1}H NMR spectrum was obtained. The solution was poured back into a vial containing a magnetic stirrer bar and an additional portion of KOH (8.4 mg, 0.15 mmol, 1.5 equiv.) was added. The vial was sealed, evacuated and backfilled with N_2 (3×), and heated to 110 °C and 900 rpm for a further 1 h. It was noted that the resulting reaction mixture was still homogeneous. The reaction was cooled, and a third ^{1}H NMR spectrum was obtained. The three ^{1}H NMR spectra are shown below.

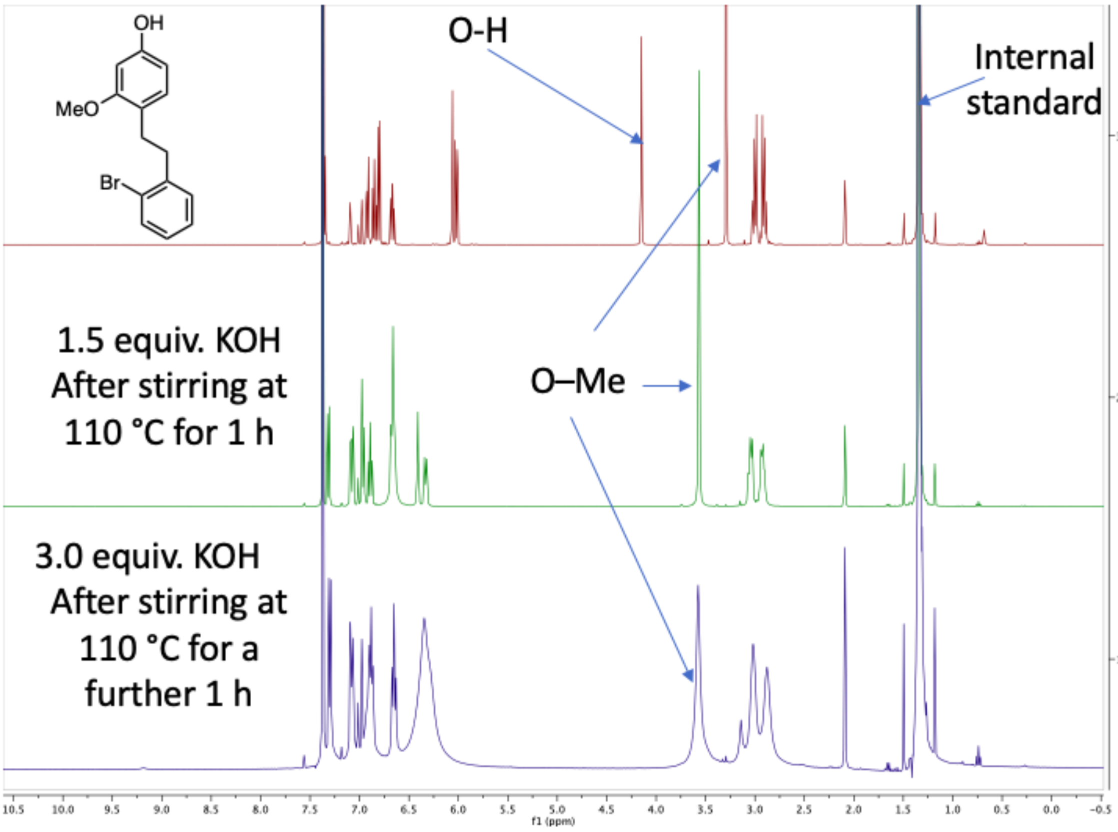

N.B. The reason for broad signals upon addition of 3.0 equiv. KOH was not determined.

It can be seen that the addition of 1.5 equiv. KOH and stirring at 110 °C for 1 h resulted in the disappearance of the O-H signal, as well as a downfield shift of signals corresponding to substituents on the phenol ring, most notably the O-Me signal. The addition of 3.0 equiv. KOH under the same conditions resulted in no further downfield shift. This suggests that deprotonation of phenol **1a** is complete upon addition of 1.5 equiv. KOH and heating at 110 °C for 1 h. Comparison of integrals of phenol **1a** to the internal standard suggested that no significant mass loss had occurred during this process.

The spectrum corresponding to the addition of 1.5 equiv. KOH was also compared to a ^{1}H NMR (PhMe-d_8) spectrum of an authentic sample of the potassium phenolate that had been independently prepared (see **Synthesis of Starting Materials in Scheme 5**). Good alignment of the chemical shifts provided further evidence for complete deprotonation.

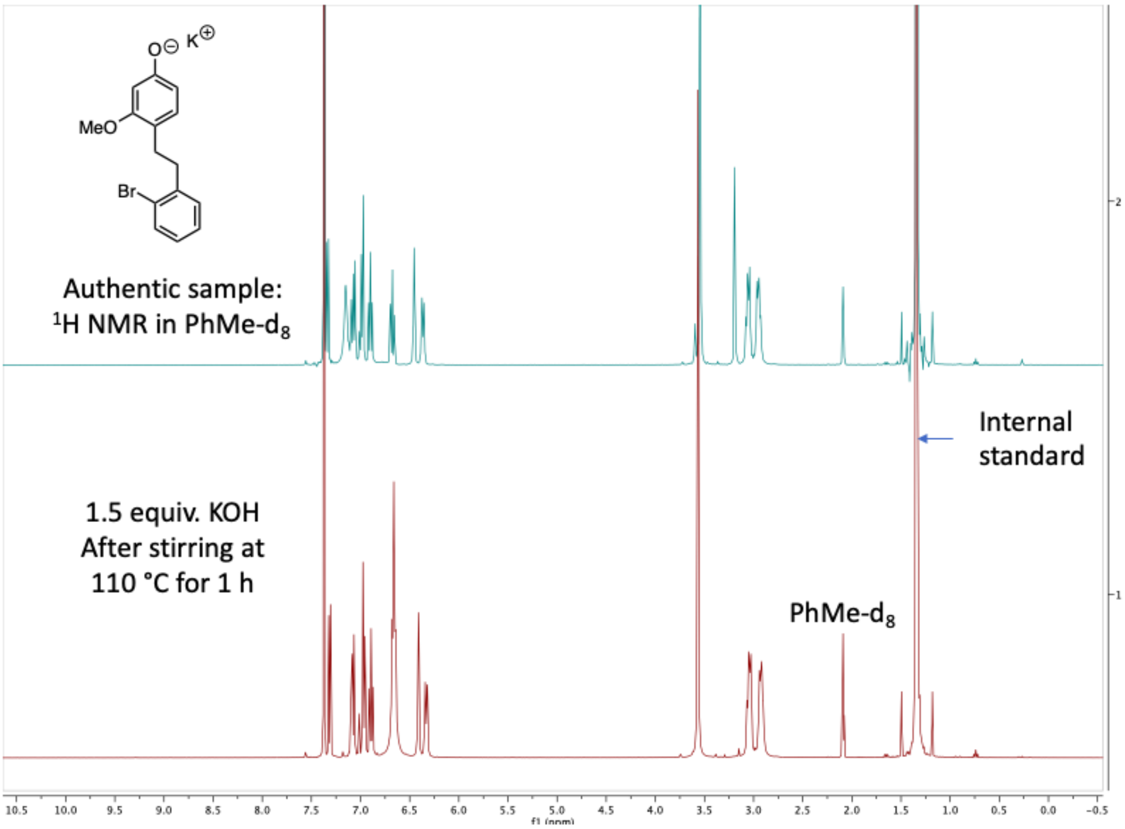

N.B. The Pd-catalyzed dearomatization reaction used a PhMe:H_2O (10:1) biphasic solvent mixture. Due to the difficulty obtaining ^{1}H NMR data for biphasic mixtures, neat d^8-PhMe was used as the solvent in these NMR studies. Since the results suggests that complete deprotonation of phenol **1a** occurs under these conditions, we reasoned that this would also be the case in the PhMe: H_2O (10:1) solvent used in the reaction, given the anticipated pKa difference between phenol **1a** and KOH in water.

Extended Data for Crown Ether Control Experiments

Crown Ether Experiments with KOH as the Base

Full data for crown ether experiments presented in Scheme 5C are presented below.

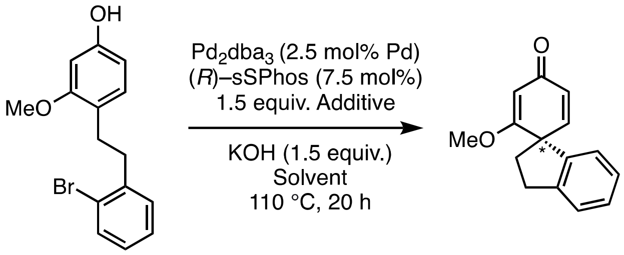

1a **2a**

Entry	Solvent	Additive	Yield/ %[a]	ee/ %[b]
**1**	PhMe	none	60	83
**2**	PhMe	12-Crown-4	62	81
**3**	PhMe	15-Crown-5	62	61
**4**	PhMe	18-Crown-6	71	70
**5**	1,4-Dioxane	none	48	84
**6**	1,4-Dioxane	12-Crown-4	51	84
**7**	1,4-Dioxane	15-Crown-5	41	63
**8**	1,4-Dioxane	18-Crown-6	61	63

[a] Yields determined by ^{1}H NMR with reference to a dibromomethane internal standard. Values in parentheses corresponds to the isolated sample. [b]*ee* determined by chiral SFC analysis of the crude reaction mixture

Crown Ether Experiments with NaOH as the Base

An analogous set of control experiments was performed with NaOH as the base, in PhMe and 1,4-dioxane.

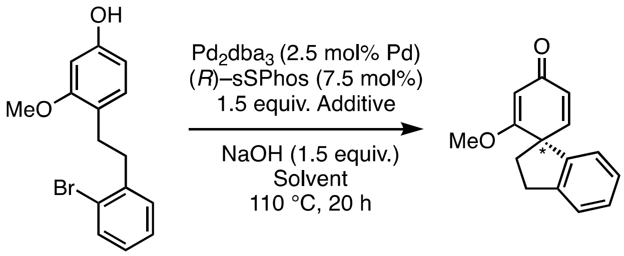

1a **2a**

Entry	Solvent	Additive	Yield/ %[a]	ee/ %[b]
**1**	PhMe	none	60	83

**2**	PhMe	12-Crown-4	27	68
**3**	PhMe	15-Crown-5	67	71
**4**	PhMe	18-Crown-6	34	66
**5**	1,4-Dioxane	none	73	89
**6**	1,4-Dioxane	12-Crown-4	41	86
**7**	1,4-Dioxane	15-Crown-5	51	65
**8**	1,4-Dioxane	18-Crown-6	55	53

[a] Yields determined by ^{1}H NMR with reference to a 1,2-dibromomethane internal standard. Values in parentheses corresponds to the isolated sample. [b]*ee* determined by chiral SFC analysis of the crude reaction mixture

Collectively, these results provide evidence for the involvement of the alkali metal cation in the enantiodetermining transition state.

Determination of Product Absolute Stereochemistry

Absolute stereochemistry of **2a** was determined as *R* by single crystal X-ray analysis, performed by Dr. Andrew Bond (deposited in the CCDC with deposition number 2290408).

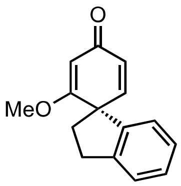

2a

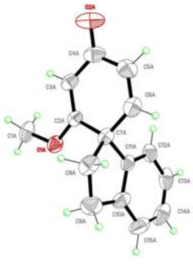

Absolute stereochemistry of the remaining products in Scheme 1 were assigned by analogy. Additionally, the optical rotation of **2c** was compared with that reported by Ming and co-workers.[2] This resulted in an assignment of *S* absolute stereochemistry for **2c**, consistent with the X-ray data for **2a** in terms of the face of the phenol from which arylation occurs:

2c: $[\alpha]_D^{25}$ = +317° (c 0.42, $CHCl_3$) for 87% ee. Literature value: **$[\alpha]_D^{25}$** = +407° (c 1.78, $CHCl_3$) for 99% ee)**.**[2]

Absolute stereochemistry of products in Scheme 2 were assigned as *R* by comparing the optical rotation of **4a** with that reported by You:

4a: $[\alpha]_D^{25}$ = +142° (c 0.85, $CHCl_3$) for 93% ee. Literature value: **$[\alpha]_D^{29}$** = +241° (c 0.20, $CHCl_3$) for 94% ee)**.**[3]

Absolute stereochemistry of products in Scheme 3 were assigned as *S* by comparing the optical rotation of **7a** with that obtained by Tang for the *R* enantiomer:

[α]$_D$^{25} = +253° (c. 0.42, $CHCl_3$) for 93% ee. Literature value: **[α]$_D$^{28}** = –269° (c 0.62, $CHCl_3$) for 92% ee, *R* enantiomer)**.**[4]

Based on the X-ray and optical rotation data, all products in Schemes 1-3 were found to have consistent absolute configuration in terms of the face of the phenol from which arylation occurs (see Scheme 5E). Stereochemistry of products in Scheme 4 (**9a**-**9c**) is assigned by analogy to these.

Synthesis of Starting Materials in Scheme 1

General Procedure A: Phosphonium Salt Synthesis

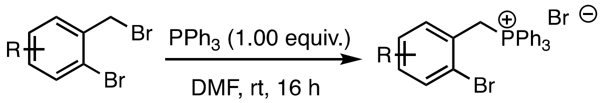

Adapted from the procedure reported by Buchwald.[5] 2-Bromobenzyl bromide derivative (1.00 equiv.) was dissolved in DMF (0.2 M) and stirred under nitrogen. PPh_3 (1.00 equiv.) was added, and the reaction stirred at room temperature for 16 h. The DMF was removed under a stream of air. The crude product was dissolved in minimal CH_2Cl_2 and precipitated with Et_2O. The suspension was filtered, and the precipitate was collected and dried under vacuum.

General Route 1: Synthesis of Starting Materials via Benzyl-protected Phenols

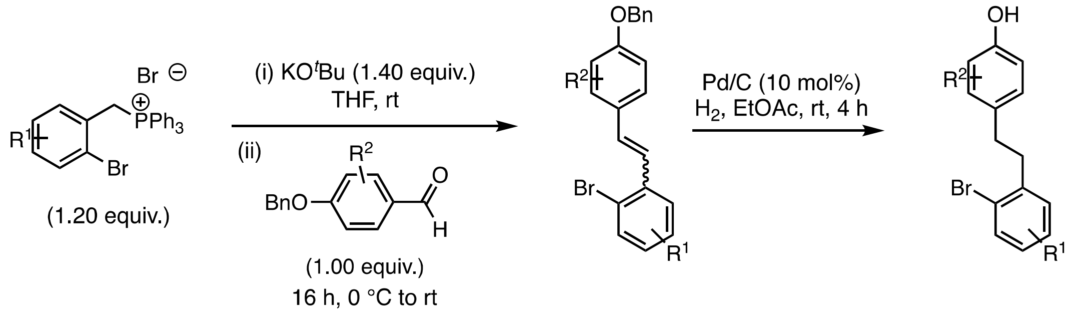

General Procedure B: Wittig Alkene Synthesis

Adapted from the procedure reported by Buchwald.[5] A 0.2 M solution of phosphonium salt (1.20 equiv.) in THF was cooled to 0 °C. KO^{t}Bu (1.40 equiv.) was added, and the reaction was stirred at 0 °C for 30 minutes. The aldehyde (1.00 equiv.) was added, and the reaction was warmed to

room temperature and stirred for 16 h. Upon completion, the reaction was quenched with water and the aqueous layer was extracted with EtOAc (3×). The combined organic extracts were washed with brine, dried over Na_2SO_4, concentrated under reduced pressure, and purified by flash column chromatography.

General Procedure C: Alkene Hydrogenation

Adapted from the procedure reported by Buchwald.[5] To a 0.1 M solution of alkene in EtOAc was added Pd/C (10 wt % loading Pd, 10 mol% Pd). The flask was capped with a septum and evacuated and backfilled with hydrogen three times. The reaction was stirred at room temperature under a hydrogen atmosphere, and monitored by ^{1}H NMR until completion (2–16 h) (N.B. longer reaction times resulted in significant Ar–Br protodehalogenation). The reaction was filtered through celite, eluting with EtOAc and MeOH, and concentrated under reduced pressure. The crude product was either purified by flash column chromatography, or subjected to the next step without further purification.

General Route 2: Synthesis of Starting Materials via Methoxy-protected Phenols

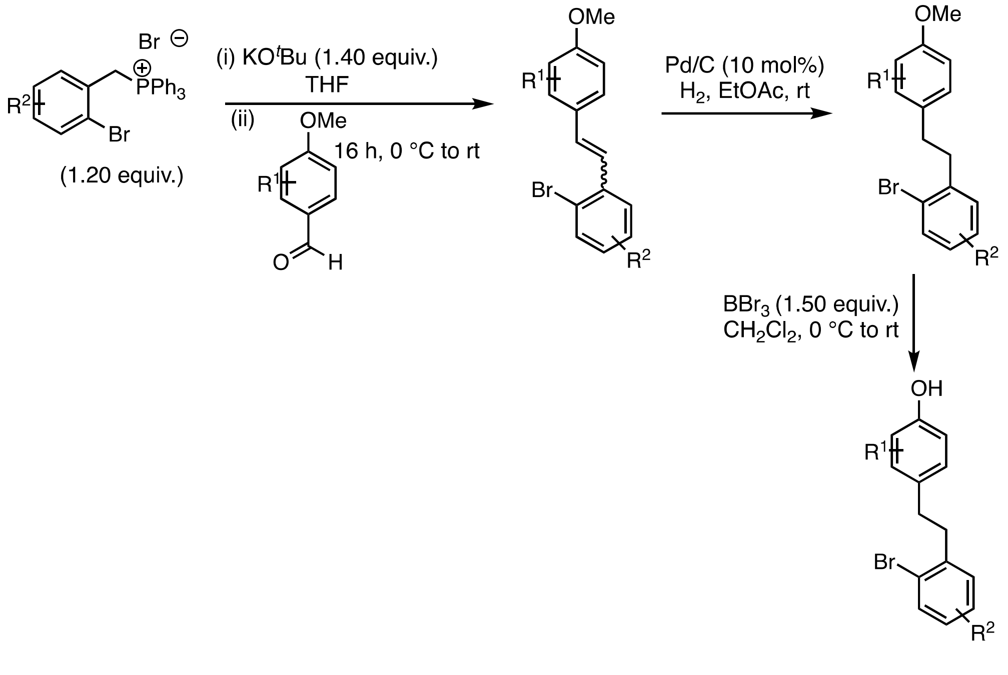

General Procedure D: Deprotection of Methoxy-protected Phenols

A 0.2 M solution of methoxy-protected phenol in CH_2Cl_2 was cooled to 0 °C under nitrogen. BBr_3 (1.50 equiv., 1.00 M in CH_2Cl_2) was added, and the reaction was stirred at 0 °C for 1 h. The reaction was warmed to room temperature and stirred until completion, as judged by TLC (approx. 3 h). Upon completion, H_2O and CH_2Cl_2 were added, and the organic layer was

separated. The aqueous layer was extracted with CH_2Cl_2 (2×). The combined organic extracts were washed with sat. $NaHCO_3$ (aq.), dried over Na_2SO_4, concentrated under reduced pressure, and purified by flash column chromatography.

General Route 3: Synthesis of Starting Materials via Unprotected Phenols

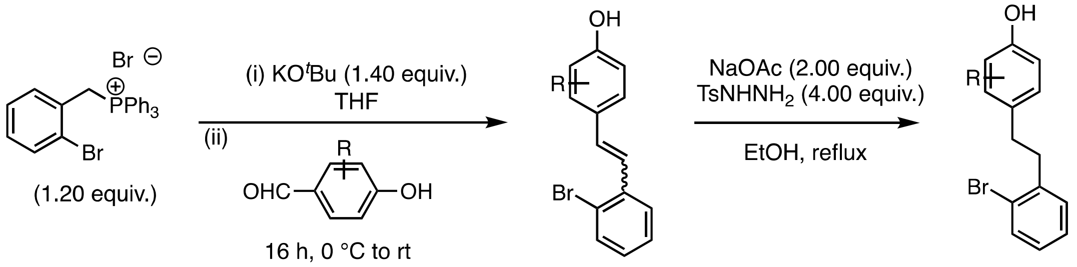

General Procedure E: Diimide Reduction of Alkenes

Adapted from the procedure reported by Tang.[4] To a 0.13 M solution of olefin in EtOH was added NaOAc (2.00 equiv.) and *p*-toluenesulfonyl hydrazide (4.00 equiv.). The reaction was heated to 95 °C under reflux until completion, as judged by ^{1}H NMR. Upon completion, the reaction was quenched with sat. NH_4Cl (aq.), diluted with H_2O, and extracted with EtOAc (3×). The combined organic extracts were washed with brine, dried over Na_2SO_4, concentrated under reduced pressure, and purified by flash column chromatography.

4-(benzyloxy)-2-Methoxybenzaldehyde (S4)

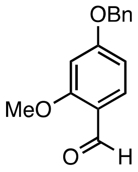

To a solution of 4-hydroxy-2-methoxybenzaldehyde (2.00 g, 13.2 mmol, 1.00 equiv.) in DMF (20 ml) was added K_2CO_3 (5.45 g, 39.5 mmol, 3.00 equiv.). The reaction was stirred for 30 minutes at room temperature. Benzyl bromide (4.15 g, 3.13 ml, 26.4 mmol, 2.00 equiv.) was added, and the reaction was stirred for 1 h at room temperature, at which point it was judged complete by TLC. DMF was removed under a stream of air. EtOAc and water were added to the residue and the layers were separated. The aqueous layer was extracted with EtOAc (1×). The combined organic extracts were dried over Na_2SO_4, concentrated under reduced pressure, and purified by flash column chromatography (20% EtOAc in petroleum ether) to afford the title compound as a colorless solid (3.00 g, 12.4 mmol, 94% yield).

^{1}H NMR (400 MHz, $CDCl_3$) δ 10.29 (1H, d, *J*=0.7 Hz), 7.81 (1H, d, *J*=8.7 Hz), 7.46 – 7.33 (5H, m), 6.62 (1H, ddd, *J*=8.7, 2.2, 0.7 Hz), 6.54 (1H, d, *J*=2.2 Hz), 5.13 (2H, s), 3.88 (3H, s).

^{13}C NMR (101 MHz, $CDCl_3$) δ 188.5, 165.4, 163.7, 136.1, 130.9, 128.9, 128.5, 127.7, 119.4, 106.6, 99.0, 70.5, 55.7.

Data in agreement with the literature.[6]

(2-bromobenzyl)Triphenylphosphonium bromide (S5)

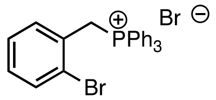

Preparation according to General Procedure A from 2-bromobenzyl bromide (15.0 g, 60.0 mmol) afforded the title compound as a colorless solid (30.7 g, quant.).

^{1}H NMR (500 MHz, $CDCl_3$) δ 7.78 (3H, t, *J*=7.4 Hz), 7.71 – 7.59 (12H, m), 7.54 (1H, dt, *J*=7.6, 2.3 Hz), 7.35 (1H, d, *J*=7.6 Hz), 7.17 (1H, t, *J*=7.6 Hz), 7.12 (1H, td, *J*=7.6, 2.0 Hz), 5.65 (2H, d, *J*=14.2 Hz).

^{13}C NMR (126 MHz, $CDCl_3$) δ 135.3 (d, *J*=3.0 Hz), 134.5 (d, *J*=9.9 Hz), 133.3 (d, *J*=4.8 Hz), 133.0 (d, *J*=3.2 Hz), 130.3 (d, *J*=12.6 Hz), 128.6 (d, *J*=3.5 Hz), 127.8 (d, *J*=8.9 Hz), 127.3 (d, *J*=6.7 Hz), 117.9, 117.2, 31.1 (d, *J*=48.5 Hz).

Data in agreement with the literature.[7]

4-(2-bromophenethyl)-3-Methoxyphenol (1a)

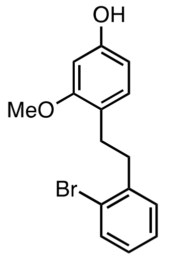

The title compound was prepared according to General Route 1. General Procedure B was performed with 4-(benzyloxy)-2-methoxybenzaldehyde (**S4**) (7.26 g, 30.0 mmol) and (2-bromobenzyl)triphenylphosphonium bromide (**S5**). The crude product was purified by flash column chromatography (0-10% Et_2O in petroleum ether) to afford the alkene as a yellow oil (10.39 g). General Procedure C was performed with the alkene (8.54 g, 21.6 mmol) (reaction

time: 4 h). The crude product was purified by flash column chromatography (10-20% Et_2O in petroleum ether) to afford the title compound as a colorless solid (4.46 g, 13.2 mmol, 44% yield over two steps).

^{1}H NMR (400 MHz, $CDCl_3$) δ 7.53 (1H, dd, *J*=8.0, 1.3 Hz), 7.21 – 7.12 (2H, m), 7.04 (1H, ddd, *J*=8.0, 7.0, 2.0 Hz), 6.93 (1H, d, *J*=8.0 Hz), 6.40 (1H, d, *J*=2.4 Hz), 6.31 (1H, dd, *J*=8.0, 2.4 Hz), 4.61 (1H, s), 3.78 (3H, s), 3.00 – 2.91 (2H, m), 2.90 – 2.77 (2H, m).

^{13}C NMR (126 MHz, $CDCl_3$) δ 158.7, 155.1, 141.7, 132.8, 130.7, 130.5, 127.6, 127.4, 124.6, 122.3, 106.7, 99.0, 55.5, 36.8, 30.2.

Data in agreement with the literature.[5]

4-(2-bromophenethyl)-3-Methylphenol (1b)

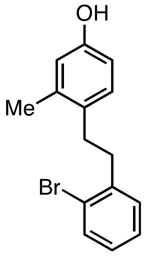

The title compound was prepared according to General Route 2. General Procedure B was performed with 4-methoxy-2-methylbenzaldehyde (1.24 g, 8.26 mmol) and (2-bromobenzyl)triphenylphosphonium bromide (**S5**). The crude product was purified by flash column chromatography (0-2% Et_2O in petroleum ether) to afford the alkene as a yellow oil (980 mg). General procedure C was performed with the alkene (980 mg, 3.23 mmol) (reaction time: 14 h), and the crude product subjected to the next step without further purification. General Procedure D was performed with the methoxy-protected phenol (762 mg, 2.50 mmol). The crude product was purified by flash column chromatography (10-20% EtOAc in petroleum ether) to afford the title compound as a colorless solid (624 mg, 2.14 mmol, 26% yield over 3 steps).

^{1}H NMR (400 MHz, $CDCl_3$) δ 7.55 (1H, dd, *J*=8.1, 1.3 Hz), 7.22 (1H, td, *J*=7.6, 1.3 Hz), 7.15 (1H, dd, *J*=7.6, 1.9 Hz), 7.07 (1H, td, *J*=7.6, 1.9 Hz), 7.02 (1H, d, *J*=8.1 Hz), 6.65 (1H, d, *J*=2.7 Hz), 6.62 (1H, dd, *J*=8.1, 2.7 Hz, 4.65 (1H, br s), 2.98 – 2.91 (2H, m), 2.86 – 2.78 (2H, m), 2.28 (3H, s).

^{13}C NMR (101 MHz, $CDCl_3$) δ 153.8, 141.3, 137.8, 132.9, 132.1, 130.7, 130.3, 127.8, 127.6, 124.5, 117.1, 112.8, 37.7, 33.0, 19.6.

Data in agreement with the literature.[5]

5-Methoxy-[1,1'-biphenyl]-2-carbaldehyde (S6)

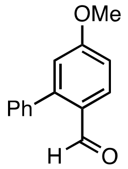

Prepared according to a modification of the procedure reported by Buchwald.[5] A round bottom flask and stirrer bar were dried with a heat gun under vacuum. 2-Bromo-4-methoxybenzaldehyde (5.00 g, 23.3 mmol, 1.00 equiv.), phenylboronic acid (4.25 g, 34.9 mmol, 1.50 equiv.), $Pd(OAc)_2$ (106 mg, 0.472 mmol, 2.00 mol%), SPhos (290 mg, 0.706 mmol, 3.00 mol%) and K_3PO_4 (9.89 g, 46.6 mmol, 2.00 equiv.) were added, and the flask was evacuated and backfilled with nitrogen (3×). 100 ml dry PhMe was added, and the reaction was heated to 60 °C for 16 h. Upon completion, the reaction was cooled to room temperature, filtered through celite, washing with EtOAc, concentrated under reduced pressure, and purified by flash column chromatography (5% EtOAc in petroleum ether) to afford the title compound as a yellow oil (4.61g, 21.7 mmol, 93% yield).

^{1}H NMR (700 MHz, $CDCl_3$) δ 9.84 (1H, s), 8.03 (1H, d, *J*=8.7 Hz), 7.48 – 7.42 (3H, m), 7.41 – 7.38 (2H, m), 7.01 (1H, ddd, *J*=8.7, 2.5, 1.0 Hz), 6.88 (1H, d, *J*=2.5 Hz), 3.91 (3H, s).

^{13}C NMR (176 MHz, $CDCl_3$) δ 191.2, 163.7, 148.7, 138.0, 130.1, 130.1, 128.5, 128.3, 127.5, 115.3, 114.1, 55.8.

Data in agreement with the literature.[8]

6-(2-bromophenethyl)-[1,1'-biphenyl]-3-ol (1c)

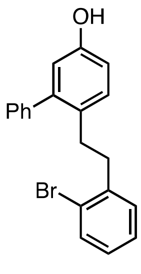

The title compound was prepared according to General Route 2. General Procedure B was performed with 5-methoxy-[1,1'-biphenyl]-2-carbaldehyde **(S6)** (1.75 g, 8.26 mmol) and (2-

bromobenzyl)triphenylphosphonium bromide (**S5**). The crude product was purified by flash column chromatography (0-2% Et_2O in petroleum ether) to afford the alkene as a yellow oil (2.28 g). General procedure C was performed with the alkene (1.00 g, 2.73 mmol) (reaction time: 24 h), and the crude product was subjected to the next step without further purification. General Procedure D was performed with the methoxy-protected phenol (assuming 2.73 mmol). The crude product was purified by flash column chromatography (10-20% EtOAc in petroleum ether) to afford the title compound as a yellow solid (174 mg, 0.49 mmol, 6% yield over 3 steps).

^{1}H NMR (400 MHz, $CDCl_3$) δ 7.46 (1H, ddd, *J*=8.0, 3.1, 1.3 Hz), 7.44 – 7.34 (3H, m), 7.31 – 7.25 (2H, m), 7.21 (1H, dd, *J*=8.2, 2.7 Hz), 7.12 (1H, tt, *J*=7.6, 1.6 Hz), 7.01 (1H, tt, *J*=7.6, 2.1 Hz), 6.87 (1H, dq, *J*=7.6, 1.6 Hz), 6.81 (1H, dt, *J*=8.2, 3.1 Hz), 6.72 (1H, t, *J*=3.1 Hz), 5.18 – 4.87 (1H, m), 2.90 – 2.70 (4H, m).

^{13}C NMR (101 MHz, $CDCl_3$) δ 153.6, 143.5, 141.5, 141.1, 132.8, 131.4, 130.9, 130.6, 129.2, 128.3, 127.7, 127.4, 127.1, 124.4, 116.9, 114.5, 38.4, 32.8.

HRMS m/z: $[M–H]^-$ calculated for $[C_{20}H_{16}BrO]^-$ 351.0390, found 351.0397. Δ = +2.0 ppm.

6-Methoxy-[1,1'-biphenyl]-3-carbaldehyde (S7)

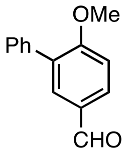

Prepared according to a modification of the procedure reported by Buchwald.[5] A round bottom flask and stirrer bar were dried with a heat gun under vacuum. 3-Bromo-4-methoxybenzaldehyde (2.00 g, 9.32 mmol, 1.00 equiv.), phenylboronic acid (1.70 g, 14.0 mmol, 1.50 equiv.), $Pd(OAc)_2$ (41.8 mg, 0.186 mmol, 2.00 mol%), SPhos (116 mg, 0.282 mmol, 3.00 mol%) and K_3PO_4 (3.95 g, 18.6 mmol, 2.00 equiv.) were added, and the flask was evacuated and backfilled with nitrogen (3×). 100 ml dry PhMe was added, and the reaction was heated to 60 °C for 16 h. Upon completion, the reaction was cooled to room temperature, filtered through celite, washing with EtOAc, concentrated under reduced pressure, and purified by flash column chromatography (10% EtOAc in petroleum ether) to afford the title compound as a colorless oil (1.89g, 8.92 mmol, 96% yield).

^{1}H NMR (400 MHz, $CDCl_3$) δ 9.94 (1H, s), 7.92 – 7.83 (2H, m), 7.56 – 7.51 (2H, m), 7.44 (2H, ddd, *J*=7.7, 6.5, 1.5 Hz), 7.38 (1H, td, *J*=7.3, 1.5 Hz), 7.10 (1H, d, *J*=8.1 Hz), 3.91 (3H, s)

^{13}**C NMR** (101 MHz, $CDCl_3$) δ 191.0, 161.6, 137.2, 132.5, 131.5, 131.5, 130.0, 129.6, 128.3, 127.7, 111.2, 56.0.

Data in agreement with the literature.[9]

5-(2-bromophenethyl)-[1,1'-biphenyl]-2-ol (1d)

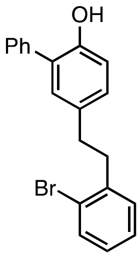

The title compound was prepared according to General Route 2. General Procedure B was performed with 6-methoxy-[1,1'-biphenyl]-3-carbaldehyde **(S7)** (1.75 g, 8.26 mmol) and (2-bromobenzyl)triphenylphosphonium bromide (**S5**). The crude product was purified by flash column chromatography (0-5% Et_2O in petroleum ether) to afford the alkene as a yellow oil (1.94 g). General procedure C was performed with the alkene (710 mg, 1.94 mmol) (reaction time: 16 h). The crude product was purified by flash column chromatography (0-5% Et_2O in petroleum ether) to afford the methoxy-protected phenol as a yellow oil (420 mg). General Procedure D was performed with the methoxy-protected phenol (420 mg, 1.14 mmol). The crude product was purified by flash column chromatography (10-20% EtOAc in petroleum ether) to afford the title compound as a yellow solid (181 mg, 0.51 mmol, 6% yield over 3 steps).

^{1}**H NMR** (400 MHz, $CDCl_3$) δ 7.55 (1H, dd, *J*=8.0, 1.3 Hz), 7.52 – 7.43 (4H, m), 7.43 – 7.36 (1H, m), 7.22 (1H, td, *J*=7.3, 1.3 Hz), 7.16 (1H, dd, *J*=7.6, 1.9 Hz), 7.12 (1H, dd, *J*=8.2, 2.3 Hz), 7.10 – 7.04 (2H, m), 6.92 (1H, d, *J*=8.2 Hz), 5.08 (1H, s), 3.07 – 2.99 (2H, m), 2.92 – 2.84 (2H, m).

^{13}**C NMR** (101 MHz, $CDCl_3$) δ 150.8, 141.1, 137.4, 133.9, 133.0, 130.7, 130.3, 129.4, 129.3, 129.2, 128.0, 128.0, 127.8, 127.5, 124.6, 115.9, 38.8, 35.5.

HRMS m/z: $[M–H]^-$ calculated for $[C_{20}H_{16}BrO]^-$ 351.0390, found 351.0383. Δ = –2.0 ppm.

4-(2-bromophenethyl)-2-Methoxyphenol (1e)

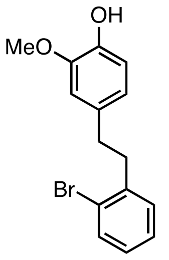

The title compound was prepared according to General Route 1. General Procedure B was performed with 4-(benzyloxy)-3-methoxybenzaldehyde (2.00 g, 8.26 mmol) and (2-bromobenzyl)triphenylphosphonium bromide (**S5**). The crude product was purified by flash column chromatography (10-30% Et_2O in petroleum ether) to afford the alkene as a colorless solid (1.96 g). General Procedure C was performed with the alkene (1.26 g, 3.19 mmol) (reaction time: 2 h). The crude product was purified by flash column chromatography (10-20% Et_2O in petroleum ether) to afford the title compound as a colorless oil (707 mg, 2.30 mmol, 28% yield over two steps).

^{1}H NMR (500 MHz, $CDCl_3$) δ 7.56 (1H, dd, *J*=8.0, 1.4 Hz), 7.21 (1H, td, *J*=7.4, 1.4 Hz), 7.14 (1H, dd, *J*=7.6, 1.8 Hz), 7.07 (1H, td, *J*=7.6, 1.8 Hz), 6.85 (1H, dd, *J*=8.1, 2.0 Hz), 6.73 (1H, dd, *J*=8.1, 1.9 Hz), 6.66 (1H, d, *J*=2.0 Hz), 5.53 – 5.47 (1H, m), 3.85 (3H, s), 3.04 – 2.98 (2H, m), 2.88 – 2.82 (2H, m).

^{13}C NMR (126 MHz, $CDCl_3$) δ 146.4, 144.0, 141.0, 133.5, 132.9, 130.7, 127.8, 127.5, 124.6, 121.2, 114.3, 111.3, 56.0, 38.8, 36.0.

HRMS m/z: $[M]^+$ calculated for $[C_{15}H_{15}BrO_2]^+$ 306.0250, found 306.0263. Δ = +4.2 ppm

4-(2-bromophenethyl)-2-Methylphenol (1f)

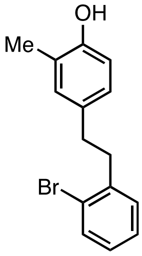

The title compound was prepared according to General Route 3. General Procedure B was performed with 4-hydroxy-3-methylbenzaldehyde (1.36 g, 10.0 mmol) and (2-bromobenzyl)triphenylphosphonium bromide (**S5**). The crude product was purified by flash column chromatography (10% EtOAc in petroleum ether) to afford the alkene as a colorless solid

(882 mg). General procedure E was performed with the alkene (578 mg, 2.00 mmol) (reaction time: 4 h). The crude product was purified by flash column chromatography (6-10% EtOAc in petroleum ether) to afford the title compound as a colorless solid (161 mg, 0.56 mmol, 6% yield over two steps).

^{1}H NMR (400 MHz, $CDCl_3$) δ 7.57 (1H, dd, *J*=8.0, 1.3 Hz), 7.26 – 7.14 (2H, m), 7.08 (1H, td, *J*=7.5, 2.0 Hz), 7.00 (1H, d, *J*=2.2 Hz), 6.95 (1H, dd, *J*=8.1, 2.2 Hz), 6.72 (1H, d, *J*=8.1 Hz), 4.74 – 4.62 (1H, m), 3.07 – 2.90 (2H, m), 2.90 – 2.75 (2H, m), 2.26 (3H, s).

^{13}C NMR (101 MHz, $CDCl_3$) δ 152.1, 141.2, 133.8, 132.9, 131.2, 130.6, 127.8, 127.5, 127.1, 124.5, 123.7, 114.9, 38.9, 35.5, 15.9.

HRMS m/z: $[M–2e]^{2+}$ calculated for $[C_{15}H_{15}BrO]^{2+}$ 145.0148, found 145.0154. Δ = + 4.2 ppm.

4-(2-bromophenethyl)-2-Fluorophenol (1g)

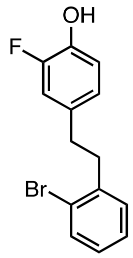

The title compound was prepared according to General Route 2. General Procedure B was performed with 3-fluoro-4-methoxybenzaldehyde (771 mg, 5.00 mmol) and (2-bromobenzyl)triphenylphosphonium bromide (**S5**). General procedure C was performed with the alkene (614 mg, 2.00 mmol) (reaction time: 3 h) and the crude product was subjected to the next step without further purification. General Procedure D was performed with the methoxy-protected phenol (assuming 2.00 mmol). The crude product was purified by flash column chromatography (10 - 40% EtOAc in petroleum ether) to afford the title compound as a brown solid (408 mg, 1.38 mmol, 28% over three steps).

^{1}H NMR (500 MHz, $CDCl_3$) δ 7.55 (1H, dd, *J*=7.9, 1.3 Hz), 7.21 (1H, td, *J*=7.4, 1.3 Hz), 7.12 (1H, dd, *J*=7.6, 1.8 Hz), 7.07 (1H, td, *J*=7.6, 1.8 Hz), 6.95 – 6.88 (2H, m), 6.85 (1H, dd, *J*=8.3, 2.0 Hz), 4.95 (1H, s), 3.02 – 2.95 (2H, m), 2.85 – 2.80 (2H, m).

^{13}C NMR ($CDCl_3$, 101 MHz) δ 150.9 (d, *J*=237.2 Hz), 141.7 (d, *J*=14.1 Hz), 140.6, 134.6 (d, *J*=6.1 Hz), 133.0, 130.7, 128.5 (d, *J*=9.2 Hz), 127.8 (d, *J*=41.2 Hz), 124.8 (d, *J*=3.4 Hz), 124.5, 117.1 (d, *J*=1.8 Hz), 115.6 (d, *J*=17.8 Hz), 38.5, 35.4 (d, *J*=1.3 Hz).

^{19}F NMR (471 MHz, $CDCl_3$) δ -141.3.

HRMS m/z: $[M–H]^–$ calculated for $[C_{14}H_{11}BrFO]^–$ 292.9983, found 292.9980. Δ = –1.0 ppm.

4-(benzyloxy)-1-Naphthaldehyde (S8)

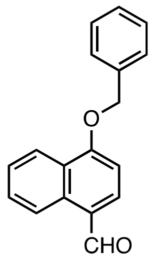

To a solution of 4-hydroxy-1-naphthaldehyde (4.13 g, 24.0 mmol, 1.00 equiv.) in DMF (40 ml) was added K_2CO_3 (9.94 g, 72.0 mmol, 3.00 equiv.). The reaction was stirred for 30 minutes at room temperature. Benzyl bromide (8.21 g, 5.70 ml, 48.0 mmol, 2.00 equiv.) was added, and the reaction was stirred for 16 h at room temperature. DMF was removed under a stream of air. EtOAc and water were added to the residue and the layers were separated. The aqueous layer was extracted with EtOAc (2×). The combined organic extracts were dried over Na_2SO_4, concentrated under reduced pressure, and purified by flash column chromatography (10-15% EtOAc in petroleum ether) to afford the title compound as a yellow solid (5.97 g, 22.8 mmol, 94% yield).

^{1}H NMR (500 MHz, $CDCl_3$) δ 10.24 – 10.16 (1H, m), 9.33 (1H, d, *J*=8.5 Hz), 8.42 (1H, d, *J*=8.5 Hz), 7.94 – 7.84 (1H, m), 7.71 (1H, ddd, *J*=8.5, 6.9, 1.5 Hz), 7.58 (1H, dd, *J*=8.5, 6.9 Hz), 7.53 (2H, d, *J*=7.6 Hz), 7.48 – 7.35 (3H, m), 6.98 (1H, t, *J*=7.4 Hz), 5.36 – 5.31 (2H, m).

^{13}C NMR (101 MHz, $CDCl_3$) δ 192.4, 160.0, 139.6, 136.1, 132.1, 129.7, 128.9, 128.5, 127.6, 126.6, 125.8, 125.3, 125.0, 122.7, 104.3, 70.7.

Data in agreement with the literature.[10]

4-(2-bromophenethyl)Naphthalen-1-ol (1h)

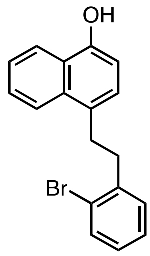

The title compound was prepared according to General Route 1. General Procedure B was performed with 4-(benzyloxy)-1-naphthaldehyde **(S8)** (1.31 g, 5.00 mmol) and (2-bromobenzyl)triphenylphosphonium bromide (**S5**), and the crude product was subjected to the next step without further purification. General procedure C was performed with the alkene (931 mg, assuming 2.24 mmol). The crude product was purified by flash column chromatography (10 – 20% EtOAc in petroleum ether) to afford the title compound as a colorless solid (91 mg, 0.28 mmol, 6% over two steps).

^{1}H NMR (400 MHz, $CDCl_3$) δ 8.29 – 8.19 (m, 1H), 8.18 – 8.10 (m, 1H), 7.58 (dd, *J* = 8.3, 1.4 Hz, 1H), 7.56 – 7.49 (m, 2H), 7.25 – 7.12 (m, 3H), 7.08 (ddd, *J* = 7.9, 7.0, 2.1 Hz, 1H), 6.74 (d, *J* = 7.6 Hz, 1H), 5.20 (s, 1H), 3.35 – 3.26 (m, 2H), 3.18 – 3.09 (m, 2H).

^{13}C NMR (101 MHz, $CDCl_3$) δ 150.3, 141.4, 133.0, 133.0, 130.8, 130.2, 127.9, 127.6, 126.6, 126.1, 125.1, 124.9, 124.5, 124.0, 122.4, 108.3, 38.0, 33.1.

HRMS m/z: $[M–H]^-$ calculated for $[C_{18}H_{14}BrO]^-$ 325.0234, found 325.0242, Δ = +2.5 ppm.

4-(2-bromophenethyl)-2,3-Dimethylphenol (1i)

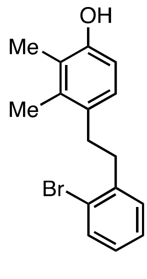

The title compound was prepared according to General Route 2. General Procedure B was performed with 2,3-dimethyl-4-methoxybenzaldehyde (821 mg, 5.00 mmol) and (2-bromobenzyl)triphenylphosphonium bromide (**S5**). General procedure C was performed with the alkene (818 mg, 2.0 mmol) (reaction time: 4 h) and purified with flash column chromatography (5 – 10% EtOAc in petroleum ether). General Procedure D was performed with the methoxy-protected phenol (536 mg, 1.86 mmol). The crude product was purified by flash column

chromatography (10-20% EtOAc in petroleum ether) to afford the title compound as a colorless solid (486 mg, 1.52 mmol, 30% over three steps).

^{1}H NMR (400 MHz, $CDCl_3$) δ 7.56 (dd, J = 7.9, 1.3 Hz, 1H), 7.25 – 7.17 (m, 2H), 7.08 (ddd, J = 7.9, 7.1, 2.0 Hz, 1H), 6.91 (d, J = 8.1 Hz, 1H), 6.60 (d, J = 8.1 Hz, 1H), 4.58 (s, 1H), 2.98 – 2.90 (m, 2H), 2.90 – 2.83 (m, 2H), 2.27 (s, 3H), 2.21 (s, 3H).

^{13}C NMR (101 MHz, $CDCl_3$) δ 152.0, 141.4, 136.7, 132.9, 132.2, 130.7, 127.8, 127.6, 127.3, 124.5, 122.9, 112.5, 38.0, 34.3, 15.7, 12.2.

HRMS m/z: $[M+H]^+$ calculated for $[C_{17}H_{19}BrO]^+$ 305.0536, found 305.0541, Δ = +1.6 ppm.

Synthesis of 4-(3-(2-bromophenyl)propyl)-3-methoxyphenol (1j)

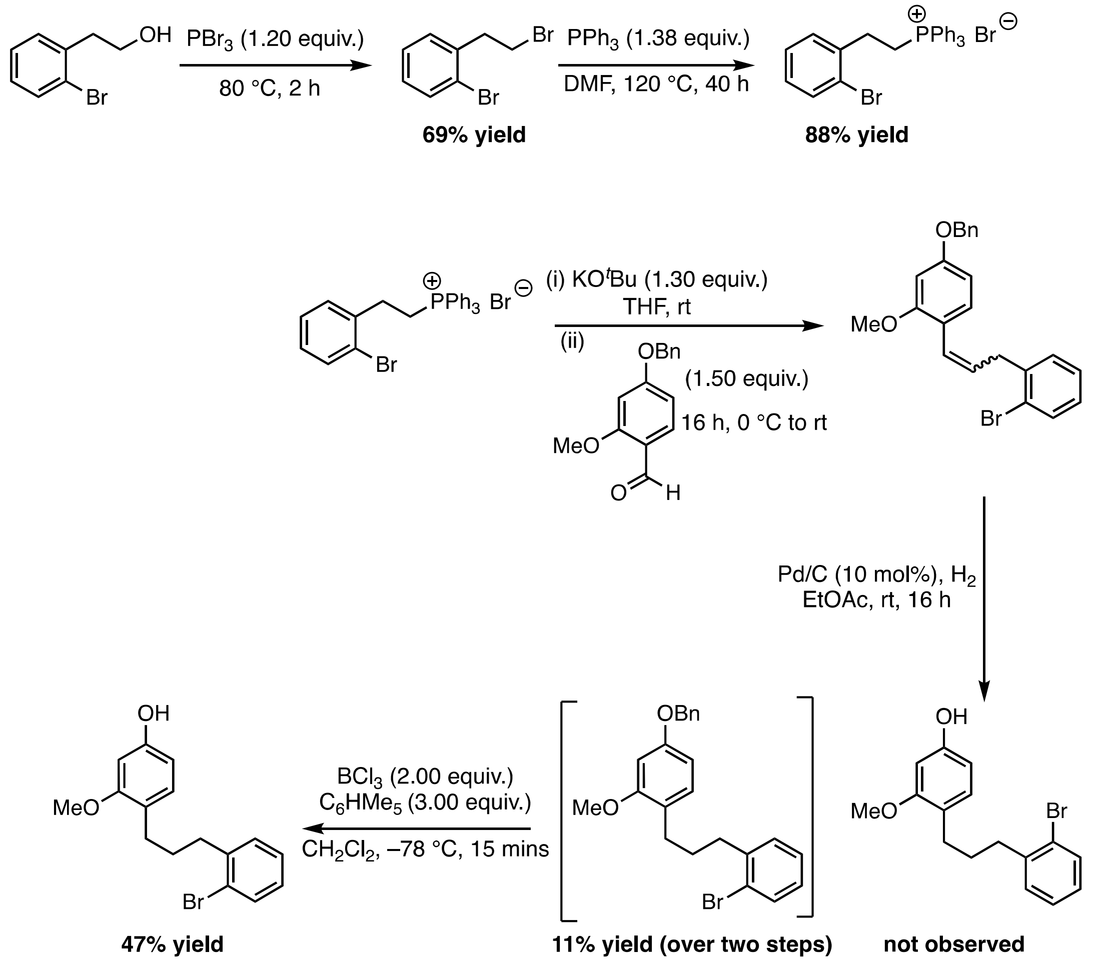

1-Bromo-2-(2-bromoethyl)benzene (S10)

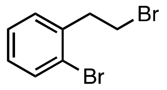

A microwave vial containing 2-bromophenethyl alcohol (5.00 g, 24.9 mmol, 1.00 equiv.) was cooled to 0 °C under N_2. PBr_3 (8.10 g, 2.84 ml, 29.9 mmol, 1.20 equiv.) was added dropwise, and the reaction was heated to 80 °C for 3 h. Upon completion, the reaction was poured onto cold water. Sat. $NaHCO_3$ (aq.) (100 ml) was added, and the mixture was stirred for 30 minutes. The aqueous layer was extracted with CH_2Cl_2 (3×). The combined organic extracts were washed with sat. $NaHCO_3$ (aq.) and brine, dried over Na_2SO_4, and concentrated under reduced pressure to afford the title compound as a colorless oil (4.55 g, 69% yield).

^{1}H NMR (500 MHz, $CDCl_3$) δ 7.55 (1H, dt, *J*=8.0, 0.9 Hz), 7.29 – 7.25 (2H, m), 7.16 – 7.11 (1H, m), 3.60 (2H, dd, *J*=8.0, 7.2 Hz), 3.30 (2H, t, *J*=7.6 Hz).

^{13}C NMR (126 MHz, $CDCl_3$) δ 138.2, 133.2, 131.3, 128.9, 127.7, 124.5, 39.7, 31.2.

Data in agreement with the literature.[11]

(2-bromophenethyl)Triphenylphosphonium bromide (S11)

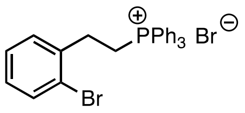

A vial was charged with 1-bromo-2-(2-bromoethyl)benzene **(S10)** (2.64 g, 10.0 mmol, 1.00 equiv.). PPh_3 (3.62 g, 13.8 mmol, 1.38 equiv.) and DMF (7 ml) were added. The vial was sealed and heated to 120 °C for 40 h. Upon completion, the vial was opened, and the DMF removed under a stream of air. The crude product was dissolved in minimal CH_2Cl_2 and precipitated with Et_2O. The suspension was filtered, and the precipitate was collected and dried under vacuum, to afford the title compound as a colorless solid (4.63 g, 8.80 mmol, 88% yield).

^{1}H NMR (400 MHz, $CDCl_3$) δ 7.90 – 7.80 (7H, m), 7.80 – 7.72 (3H, m), 7.71 – 7.62 (6H, m), 7.35 (1H, dd, *J*=8.0, 1.4 Hz), 7.24 – 7.16 (1H, m), 6.99 (1H, td, *J*=7.7, 1.7 Hz), 4.03 – 3.80 (2H, m), 3.21 – 2.99 (2H, m).

^{13}C NMR (101 MHz, $CDCl_3$) δ 137.52 – 137.19 (m), 135.46 – 135.00 (m), 134.11 – 133.57 (m), 132.36 (d, *J*=12.9 Hz), 130.81 – 130.48 (m), 129.08, 128.54 (d, *J*=3.9 Hz), 123.51, 118.73 – 117.77 (m), 117.93 – 116.73 (m), 28.57 (d, *J*=2.5 Hz), 23.56 (d, *J*=49.2 Hz).

^{31}P NMR (162 MHz, $CDCl_3$) δ 23.5.

HRMS m/z: $[M–Br]^+$ calculated for $[C_{26}H_{23}BrP]^+$ 445.0715, found 445.0718. Δ = +0.7 ppm.

4-(benzyloxy)-1-(3-(2-bromophenyl)propyl)-2-Methoxybenzene (S12)

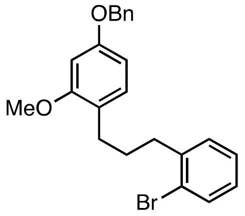

Wittig Alkene Synthesis: Adapted from the procedure reported by Stahl.[12] A heat gun-dried two-necked round bottom flask was charged with (2-bromophenethyl)triphenylphosphonium bromide (**S11**) (3.16 g, 6.00 mmol, 1.00 equiv.) under N_2. Dry THF (40 ml) was added via syringe. The solution was cooled to 0 °C and KO^{t}Bu (1.65 g, 7.80 mmol, 1.30 equiv.) was added slowly. The reaction was stirred at 0 °C for 15 minutes, and at room temperature for 45 minutes. The reaction was cooled back to 0 °C, and a solution of 4-(benzyloxy)-2-methoxybenzaldehyde (**S4**) (2.18 g, 9.00 mmol, 1.50 equiv.) in THF (20 ml) was added dropwise. The reaction was warmed to room temperature and stirred for 16 h. Upon completion, Et_2O and H_2O were added, and the layers were separated. The aqueous layer was extracted with Et_2O (2×). The combined organic extracts were washed with brine, dried over Na_2SO_4, concentrated under reduced pressure, and purified by flash column chromatography (0-5% EtOAc in petroleum ether) to afford the alkene as a yellow oil (1.37 g).

Alkene Hydrogenation: General Procedure C was performed with the alkene (1.10 g, 2.69 mmol) (reaction time: 16 h). [N.B. Benzyl deprotection was not observed.] The crude product was purified by flash column chromatography (1-2% Et_2O in petroleum ether) to afford the title compound as a yellow oil (259 mg, 0.63 mmol, 11% yield over two steps).

^{1}H NMR (500 MHz, $CDCl_3$) δ 7.52 (1H, d, *J*=8.0 Hz), 7.44 (2H, d, *J*=7.5 Hz), 7.39 (2H, t, *J*=7.5 Hz), 7.33 (1H, t, *J*=7.5 Hz), 7.24 – 7.17 (2H, m), 7.08 – 7.00 (2H, m), 6.53 (1H, d, *J*=2.4 Hz), 6.49 (1H, dd, *J*=8.1, 2.4 Hz), 5.04 (2H, s), 3.78 (3H, s), 2.76 (2H, t, *J*=8.0 Hz), 2.64 (2H, t, *J*=7.7 Hz), 1.89 (2H, p, *J*=7.8 Hz).

^{13}C NMR (126 MHz, $CDCl_3$) δ 158.5, 158.5, 142.1, 137.3, 132.8, 130.3, 130.0, 128.7, 128.1, 127.7, 127.5, 127.4, 124.7, 123.4, 104.8, 99.5, 70.3, 55.4, 36.1, 30.1, 29.5.

HRMS m/z: $[M+H]^+$ calculated for $[C_{23}H_{24}BrO_2]^+$ 411.0954, found 411.0973. Δ = +4.6 ppm.

4-(3-(2-bromophenyl)propyl)-3-Methoxyphenol (1j)

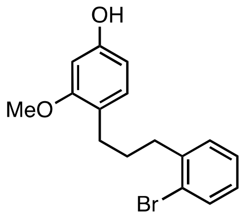

Adapted from the procedure reported by Tokuyama.[13] A flask was charged with 4-(benzyloxy)-1-(3-(2-bromophenyl)propyl)-2-methoxybenzene (**S12**) (205 mg, 0.50 mmol, 1.00 equiv.), pentamethylbenzene (222 mg, 1.50 mmol, 3.00 equiv.) and dry CH_2Cl_2 (3 ml) under N_2. The reaction was cooled to –78 °C and BCl_3 (1.00 M in heptane, 1.00 ml, 1.00 mmol, 2.00 equiv.) was added over 10 minutes. The reaction was stirred at –78 °C for 15 minutes. Upon completion, the reaction was quenched with $CHCl_3$: MeOH (10:1) (2 ml) at –78 °C and warmed to room temperature. The solvent was removed under a stream of air. The crude product was purified by flash column chromatography to afford the title compound as a yellow oil (75 mg, 0.23 mmol, 47% yield).

^{1}H NMR (400 MHz, $CDCl_3$) δ 7.52 (1H, d, *J*=7.9 Hz), 7.25 – 7.18 (2H, m), 7.04 (1H, ddd, *J*=8.7, 5.4, 3.7 Hz), 6.98 (1H, d, *J*=8.0 Hz), 6.40 (1H, d, *J*=2.4 Hz), 6.34 (1H, dd, *J*=8.0, 2.4 Hz), 4.81 (1H, s), 3.78 (3H, s), 2.80 – 2.71 (2H, m), 2.67 – 2.59 (2H, m), 1.88 (2H, tt, *J*=9.9, 6.7 Hz).

^{13}C NMR (126 MHz, $CDCl_3$) δ 158.6, 155.0, 142.1, 132.8, 130.3, 130.3, 127.5, 127.4, 124.7, 123.0, 106.5, 99.0, 55.4, 36.1, 30.1, 29.5.

HRMS m/z: $[M–H]^-$ calculated for $[C_{16}H_{16}BrO_2]^-$ 319.0339, found 319.0344. Δ = +1.6 ppm.

1-Bromo-2-(3-bromopropyl)benzene (S13)

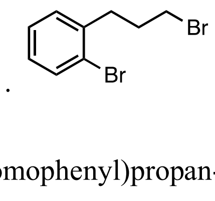

A microwave vial containing 3-(2-bromophenyl)propan-1-ol (5.00 g, 23.3 mmol, 1.00 equiv.) was cooled to 0 °C under N_2. PBr_3 (7.56 g, 2.65 ml, 27.9 mmol, 1.20 equiv.) was added dropwise, and the reaction was heated to 80 °C for 3 h. Upon completion, the reaction was poured onto cold water. Sat. $NaHCO_3$ (aq.) (100 ml) was added, and the mixture was stirred for 30 minutes. The

aqueous layer was extracted with CH_2Cl_2 (3×). The combined organic extracts were washed with sat. $NaHCO_3$ (aq.) and brine, dried over Na_2SO_4, and concentrated under reduced pressure to afford the title compound as a colorless oil (4.38 g, 68% yield).

^{1}H NMR (400 MHz, $CDCl_3$) δ 7.58 – 7.50 (1H, m), 7.29 – 7.22 (2H, m), 7.08 (1H, ddd, *J*=8.0, 6.2, 2.9 Hz), 3.44 (2H, t, *J*=6.6 Hz), 2.97 – 2.84 (2H, m), 2.27 – 2.11 (2H, m).

^{13}C NMR (101 MHz, $CDCl_3$) δ 140.0, 133.1, 130.8, 128.1, 127.6, 124.6, 34.6, 33.1, 32.6.

Data in agreement with the literature.[14]

(3-(2-bromophenyl)propyl)Triphenylphosphonium bromide (S14)

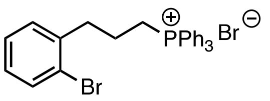

A vial was charged with 1-bromo-2-(3-bromopropyl)benzene (**S13**) (2.78 g, 10.0 mmol, 1.00 equiv.). PPh_3 (3.62 g, 13.8 mmol, 1.38 equiv.) and DMF (7 ml) were added. The vial was sealed and heated to 120 °C for 40 h. Upon completion, the vial was opened, and the DMF removed under a stream of air. The crude product was dissolved in minimal CH_2Cl_2 and precipitated with Et_2O. The suspension was filtered, and the precipitate was collected and dried under vacuum, to afford the title compound as a colorless solid (5.09 g, 9.43 mmol, 94% yield).

^{1}H NMR (400 MHz, $CDCl_3$) δ 7.81 – 7.72 (9H, m), 7.68 – 7.62 (6H, m), 7.54 (1H, dd, *J*=7.6, 1.7 Hz), 7.42 (1H, dd, *J*=7.6, 1.2 Hz), 7.22 (1H, td, *J*=7.6, 1.2 Hz), 7.03 (1H, td, *J*=7.6, 1.7 Hz), 3.94 – 3.86 (2H, m), 3.16 (2H, t, *J*=7.5 Hz), 2.06 – 1.89 (2H, m).

^{13}C NMR (101 MHz, $CDCl_3$) δ 139.6 (d, *J*=1.3 Hz), 135.1 (d, *J*=3.0 Hz), 133.8 (d, *J*=10.0 Hz), 132.8, 132.3, 130.6 (d, *J*=12.6 Hz), 128.4, 128.0, 124.2, 118.4 (d, *J*=85.9 Hz), 36.4 (d, *J*=17.3 Hz), 22.6 (d, *J*=3.8 Hz), 22.1 (d, *J*=50.9 Hz).

Data in agreement with the literature.[14]

4-(4-(2-bromophenyl)butyl)-3-Methoxyphenol (1k)

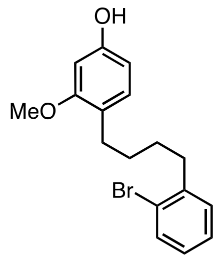

Wittig Alkene Synthesis: Adapted from the procedure reported by Stahl.[12] A heat gun-dried two-necked round bottom flask was charged with (3-(2-bromophenyl)propyl)triphenylphosphonium bromide (**S14**) (3.24 g, 6.00 mmol, 1.00 equiv.) under N_2. Dry THF (40 ml) was added via syringe. The solution was cooled to 0 °C and KO^{t}Bu (1.65 g, 7.80 mmol, 1.30 equiv.) was added slowly. The reaction was stirred at 0 °C for 15 minutes, and at room temperature for 45 minutes. The reaction was cooled back to 0 °C, and a solution of 4-(benzyloxy)-2-methoxybenzaldehyde (**S4**) (2.18 g, 9.00 mmol, 1.50 equiv.) in THF (20 ml) was added dropwise. The reaction was warmed to room temperature and stirred for 16 h. Upon completion, Et_2O and H_2O were added, and the layers were separated. The aqueous layer was extracted with Et_2O (2×). The combined organic extracts were washed with brine, dried over Na_2SO_4, concentrated under reduced pressure, and purified by flash column chromatography (0-5% EtOAc in petroleum ether) to afford the alkene as a colorless oil (1.87 g).

Alkene Hydrogenation and Benzyl Deprotection: General Procedure C was performed with the alkene (1.49 g, 3.52 mmol) (reaction time: 16 h). The crude product was purified by flash column chromatography (10% EtOAc in petroleum ether) to afford the title compound as a yellow oil (272 mg, 0.81 mmol, 14% yield over two steps).

^{1}H NMR (400 MHz, $CDCl_3$) δ 7.51 (1H, d, *J*=7.5 Hz), 7.22 – 7.18 (2H, m), 7.03 (1H, ddd, *J*=8.0, 5.4, 3.6 Hz), 6.96 (1H, d, *J*=8.0 Hz), 6.39 (1H, d, *J*=2.4 Hz), 6.33 (1H, dd, *J*=8.0, 2.4 Hz), 4.65 (1H, s), 3.78 (3H, s), 2.79 – 2.71 (2H, m), 2.61 – 2.54 (2H, m), 1.71 – 1.57 (4H, m).

^{13}C NMR (101 MHz, $CDCl_3$) δ 158.6, 154.8, 142.2, 132.8, 130.5, 130.2, 127.5, 127.4, 124.6, 123.3, 106.5, 98.9, 55.4, 36.2, 29.8, 29.8, 29.4.

HRMS m/z: $[M–H]^-$ calculated for $[C_{17}H_{18}BrO_2]^-$ 333.0496, found 333.0498. Δ = +0.6 ppm.

(2-bromo-5-(trifluoromethyl)benzyl)Triphenylphosphonium bromide (S15)

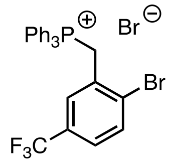

Reduction of 2-Bromo-5-trifluoromethylbenzaldehyde: 2-Bromo-5-trifluoromethylbenzaldehyde (3.80 g, 15.0 mmol, 1.00 equiv.) was dissolved in anhydrous methanol (30 mL) and cooled to 0 °C. Sodium borohydride (2.27 g, 60.0 mmol, 4.00 equiv.) was added in portions. The reaction was warmed to room temperature and stirred for 16 h. The reaction mixture was quenched with water and extracted with EtOAc (3×). The combined organic extracts were dried over $MgSO_4$, filtered, and the solvent removed under reduced pressure. The crude product was subjected to the next step without further purification.

Alkyl Bromide Formation: The crude product was dissolved in anhydrous CH_2Cl_2 (20 mL) and cooled to 0 °C. PBr_3 (23.9 g, 8.39 mL, 88.3 mmol, 5.89 equiv.) was added dropwise under a nitrogen atmosphere, and the reaction was stirred for 1 hour at room temperature. The reaction mixture was added slowly to ice water and extracted with CH_2Cl_2 (3×). The combined organic extracts were dried over $MgSO_4$, filtered, and the solvent removed under reduced pressure. The crude product was purified with a silica plug (100% petroleum ether), and subjected to the next step without further purification.

Phosphonium Salt Synthesis: General Procedure A was performed with the alkyl bromide (assuming 15.0 mmol), which afforded the title compound as a colorless solid (1.74 g, 3.01 mmol, 20% yield over three steps).

^{1}H NMR (700 MHz, MeCN-*d*$_3$) δ 7.93 – 7.89 (m, 3H), 7.73 (d, *J* = 8.4 Hz, 1H), 7.71 – 7.67 (m, 6H), 7.62 – 7.57 (m, 6H), 7.57 – 7.53 (m, 1H), 7.30 (s, 1H), 4.90 (d, *J* = 14.4 Hz, 2H).

^{13}C NMR (176 MHz, MeCN-*d*$_3$) δ 136.7 (d, *J* = 3.2 Hz), 135.6 (d, *J* = 3.1 Hz), 135.4 (d, *J* = 10.0 Hz), 131.7 (d, *J* = 6.3 Hz), 131.3 (d, *J* = 12.6 Hz), 130.7 (qd, *J* = 33.3, 3.4 Hz), 130.2 (d, *J* = 8.4 Hz), 129.9 (p, *J* = 4.1 Hz), 128.3 (p, *J* = 3.7 Hz), 124.4 (q, *J* = 271.8 Hz), 117.4 (d, *J* = 86.5 Hz), 31.8 (d, *J* = 50.6 Hz).

^{19}F NMR (471 MHz, MeCN-*d*$_3$) δ -63.7.

^{31}P NMR (203 MHz, MeCN-*d*$_3$) δ 22.4.

HRMS m/z: $[M]^+$ calculated for $[C_{26}H_{20}BrF_3P]^+$ 499.0433, found 499.0439. Δ = +1.3 ppm.

4-(2-bromo-5-(trifluoromethyl)phenethyl)-3-Methoxyphenol (1l)

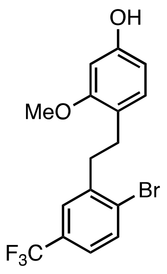

The title compound was prepared according to General Route 1. General Procedure B was performed with (2-bromo-5-(trifluoromethyl)benzyl)triphenylphosphonium bromide (**S15**) (1.74 g, 3.00 mmol) and 4-(benzyloxy)-2-methoxybenzaldehyde (**S4**) (606 mg, 2.50 mmol). General Procedure C was performed with the alkene (931 mg, 2.01 mmol). The crude product was purified by flash column chromatography (20% EtOAc in petroleum ether) to afford the title compound as a colorless solid (591 mg, 1.58 mmol, 79% over two steps).

^{1}H NMR (400 MHz, $CDCl_3$) δ 7.65 (dd, J = 8.3, 0.9 Hz, 1H), 7.36 (d, J = 2.3 Hz, 1H), 7.29 (dd, J = 8.3, 2.3 Hz, 1H), 6.91 (d, J = 8.1 Hz, 1H), 6.40 (d, J = 2.4 Hz, 1H), 6.32 (dd, J = 8.1, 2.4 Hz, 1H), 4.79 (s, 1H), 3.77 (s, 3H), 3.10 – 2.97 (m, 2H), 2.85 (dd, J = 9.4, 6.2 Hz, 2H).

^{13}C NMR (101 MHz, $CDCl_3$) δ 158.7, 155.4, 142.7, 133.2, 130.5, 129.8 (q, J = 32.7 Hz), 128.5 (q, J = 1.7 Hz), 127.3 (q, J = 3.7 Hz), 124.2 (q, J = 3.7 Hz), 124.1 (q, J = 272.2 Hz), 121.5, 106.7, 99.0, 55.4, 36.8, 29.9.

^{19}F NMR (376 MHz, $CDCl_3$) δ -63.6.

HRMS m/z: $[M+K]^+$ calculated for $[C_{16}H_{14}BrF_3KO_2]^+$ 412.9761, found 412.9777. Δ = +4.0 ppm.

(2-bromo-4,5-dimethoxybenzyl)Triphenylphosphonium bromide (S16)

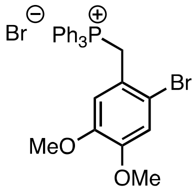

Preparation according to General Procedure A from 2-bromo-4,5-dimethoxybenzyl bromide (4.65 g, 15.0 mmol) afforded the title compound as a colorless solid (6.09 g, 10.6 mmol, 71%).

^{1}H NMR (400 MHz, MeCN-d_3) δ 7.93 – 7.86 (m, 3H), 7.69 (ddt, J = 8.5, 7.6, 2.7 Hz, 6H), 7.62 – 7.55 (m, 6H), 7.00 (d, J = 0.7 Hz, 1H), 6.55 (d, J = 2.2 Hz, 1H), 4.81 (d, J = 13.7 Hz, 2H), 3.78 (s, 3H), 3.35 (s, 3H).

^{13}C NMR (101 MHz, MeCN-d_3) δ 151.3 (d, J = 3.5 Hz), 149.8 (d, J = 3.5 Hz), 136.3 (d, J = 3.0 Hz), 135.4 (d, J = 10.0 Hz), 131.1 (d, J = 12.6 Hz), 119.2 (d, J = 9.0 Hz), 118.0 (d, J = 85.9 Hz), 117.7 (d, J = 7.5 Hz), 116.8 (d, J = 3.0 Hz), 115.4 (d, J = 4.2 Hz), 56.9, 56.3, 31.5 (d, J = 49.8 Hz).

^{31}P NMR (162 MHz, MeCN-d_3) δ 26.8.

HRMS m/z: $[M]^+$ calculated for $[C_{27}H_{25}BrO_2P]^+$ 491.0770, found 491.0778. Δ = +1.6 ppm.

4-(2-bromo-4,5-dimethoxyphenethyl)-3-Methoxyphenol (1m)

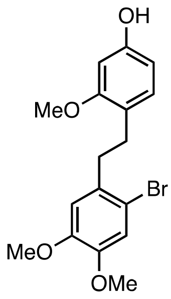

The title compound was prepared according to General Route 1. General Procedure B was performed using (2-bromo-4,5-dimethoxybenzyl)triphenylphosphonium bromide (**S16**) (3.43 g, 6.00 mmol) and 4-(benzyloxy)-2-methoxybenzaldehyde (**S4**) (1.21 g, 5.00 mmol). General Procedure C was performed with the alkene (911 mg, 2.00 mmol). The crude product was purified by flash column chromatography (20% EtOAc in petroleum ether) to afford the title compound as a colorless solid (225 mg, 0.61 mmol, 12% over two steps).

^{1}H NMR (500 MHz, Acetone-d_6) δ 8.12 (s, 1H), 7.05 (s, 1H), 6.86 (d, J = 8.1 Hz, 1H), 6.76 (s, 1H), 6.45 (d, J = 2.3 Hz, 1H), 6.32 (dd, J = 8.1, 2.3 Hz, 1H), 3.80 (s, 3H), 3.78 (s, 3H), 3.73 (s, 3H), 2.88 – 2.84 (m, 2H), 2.78 – 2.74 (m, 2H).

^{13}C NMR (126 MHz, Acetone-d_6) δ 159.4, 158.0, 149.7, 149.3, 134.33, 131.0, 121.1, 116.7, 114.9, 114.3, 107.4, 99.7, 56.4, 56.2, 55.6, 36.9, 31.0.

HRMS m/z: $[M–H]^-$ calculated for $[C_{17}H_{18}BrO_4]^-$ 365.0394, found 365.0403. Δ = +2.5 ppm.

Bromo(2-bromo-6-chlorobenzyl)triphenylphosphonium bromide (S17)

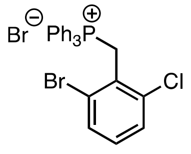

Preparation according to General Procedure A from 2-bromo-6-chlorobenzyl bromide (4.27 g, 15.0 mmol) afforded the title compound as a colorless solid (6.09 g, 11.1 mmol, 74%).

^{1}H NMR (400 MHz, MeCN-*d*$_3$) δ 7.88 (tq, *J* = 7.3, 1.7 Hz, 3H), 7.71 – 7.58 (m, 12H), 7.48 (dd, *J* = 8.1, 1.2 Hz, 1H), 7.37 – 7.32 (m, 1H), 7.23 (td, *J* = 8.1, 2.5 Hz, 1H), 5.02 (d, *J* = 14.1 Hz, 2H).

^{13}C NMR (101 MHz, MeCN-*d*$_3$) δ 137.1 (d, *J* = 5.9 Hz), 136.4 (d, *J* = 3.1 Hz), 135.4 (d, *J* = 10.0 Hz), 133.4 (d, *J* = 3.2 Hz), 132.5 (d, *J* = 3.9 Hz), 131.1 (d, *J* = 12.8 Hz), 130.6 (d, *J* = 3.2 Hz), 128.8 (d, *J* = 9.0 Hz), 127.9 (d, *J* = 6.2 Hz), 118.2 (d, *J* = 85.9 Hz), 32.8 (d, *J* = 50.9 Hz).

^{31}P NMR (162 MHz, MeCN-*d*$_3$) δ 25.5.

HRMS m/z: $[M]^+$ calculated for $[C_{25}H_{20}BrClP]^+$ 465.0169, found 465.0174. Δ = +1.1 ppm.

4-(2-bromo-6-chlorophenethyl)-3-Methoxyphenol (1n)

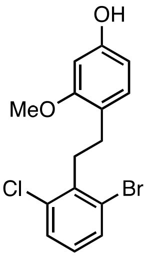

The title compound was prepared according to General Route 1. General Procedure B was performed using bromo(2-bromo-6-chlorobenzyl)triphenylphosphonium bromide (**S17**) (3.28 g, 6.00 mmol) and 4-(benzyloxy)-2-methoxybenzaldehyde (**S4**) (1.21 g, 5.00 mmol). General Procedure C was performed with the alkene (683 mg, 1.59 mmol). The crude product was purified by flash column chromatography (10 – 20% EtOAc in petroleum ether) to afford the title compound as a colorless solid (375 mg, 1.10 mmol, 18% over two steps).

^{1}H NMR (400 MHz, $CDCl_3$) δ 7.46 (dd, *J* = 8.0, 1.2 Hz, 1H), 7.31 (dd, *J* = 8.1, 1.2 Hz, 1H), 7.02 – 6.94 (m, 2H), 6.41 (d, *J* = 2.4 Hz, 1H), 6.34 (dd, *J* = 8.0, 2.4 Hz, 1H), 4.74 (s, 1H), 3.80 (s, 3H), 3.30 – 3.13 (m, 2H), 2.90 – 2.75 (m, 2H).

^{13}C NMR (101 MHz, $CDCl_3$) δ 158.9, 155.3, 139.7 135.4, 131.6, 130.4, 128.9, 128.0, 125.8, 122.2, 106.6, 98.9, 55.5, 34.8, 28.4.

HRMS m/z: [M-H]$^-$ calculated for $[C_{15}H_{13}BrClO_2]^-$ 338.9793, found 338.9800. Δ = +2.0 ppm.

***tert*-Butyl (3-bromo-4-(bromomethyl)phenyl)carbamate (S18)**

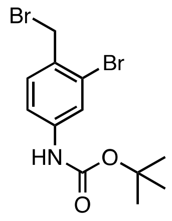

Boc Protection: 3-Bromo-4-methylaniline (1.86 g, 10.0 mmol, 1.00 equiv.) was dissolved in anhydrous THF (10 mL) and di-*tert*-butyl dicarbonate (2.18 g, 10.0 mmol, 1.00 equiv.) was added at room temperature. The reaction mixture was stirred to 55 °C for two hours and allowed to cool to room temperature. Additional di-*tert*-butyl dicarbonate (1.09 g, 5.00 mmol, 0.50 equiv.) was added and the reaction was heated to 55 °C for a further 1 hour. The reaction mixture was quenched with sat. NH_4Cl (aq.) and extracted with CH_2Cl_2 (3×). The combined organic extracts were dried with $MgSO_4$, filtered and the solvent removed under reduced pressure. The crude product was subjected to the next step without further purification.

Benzylic Bromination: The crude product was dissolved in 1,2-dichloroethane (100 mL) and AIBN (1.64 g, 10.0 mmol, 1.00 equiv.) was added in portions at room temperature. *N*-Bromosuccinimide (1.96 g, 11.0 mmol, 1.10 equiv.) was added in portions, and the reaction mixture was heated under reflux at 85 °C for 3 hours. The reaction was cooled to room temperature, quenched with 10% w/w aqueous sodium thiosulfate solution, extracted with CH_2Cl_2 (3×), and washed with brine. The combined organic extracts were dried over $MgSO_4$, filtered, and the solvent removed under reduced pressure. Purification via flash column chromatography (10 – 20% EtOAc in petroleum ether) afforded the title compound as a pale yellow solid (1.93 g, 5.29 mmol, 53% over two steps).

^{1}H NMR (400 MHz, $CDCl_3$) δ 7.73 (d, J = 2.3 Hz, 1H), 7.34 (d, J = 8.4 Hz, 1H), 7.22 (dd, J = 8.4, 2.3 Hz, 1H), 6.53 (s, 1H), 4.58 (s, 2H), 1.51 (s, 9H).

^{13}C NMR (101 MHz, $CDCl_3$) δ 152.3, 139.9, 131.7, 131.3, 125.1, 122.7, 117.7, 81.5, 33.7, 28.4.

HRMS m/z: The compound did not ionize.

***tert*-Butyl (3-bromo-4-(4-hydroxy-2-methoxyphenethyl)phenyl)Carbamate (1o)**

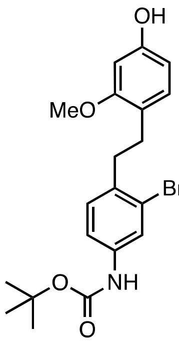

<u>Phosphonium Salt Synthesis:</u> *tert*-Butyl (3-bromo-4-(bromomethyl)phenyl)carbamate (**S18**) (1.83 g, 5.00 mmol, 1.00 equiv.) was dissolved in anhydrous DMF (4 mL). PPh_3 (1.31 g, 5.00 mmol, 1.00 equiv.) was added, and the reaction stirred for 3 hours at room temperature under N_2. The crude product was suspended in CH_2Cl_2 and Et_2O added. The precipitate filtered off to afford the phosphonium bromide as a colorless solid. The crude product was carried forward without further purification.

The title compound was prepared according to General Route 1. General procedure B was performed with crude (2-bromo-4-((*tert*-butoxycarbonyl)amino)benzyl)triphenylphosphonium bromide (1.57 g, 2.50 mmol) and 4-(benzyloxy)-2-methoxybenzaldehyde (**S4**) (505 mg, 2.08 mmol). General Procedure C was performed with the alkene (510 mg, 1.00 mmol). The crude product was purified by flash column chromatography (10-30% EtOAc in petroleum ether) to afford the title compound as a colorless solid (89 mg, 0.21 mmol, 10% over two steps).

^{1}H NMR (400 MHz, $CDCl_3$) δ 7.66 (s, 1H), 7.11 (d, J = 8.2 Hz, 1H), 7.02 (d, J = 8.2 Hz, 1H), 6.90 (d, J = 8.0 Hz, 1H), 6.39 (d, J = 2.2 Hz, 2H), 6.30 (dd, J = 8.0, 2.2 Hz, 1H), 3.78 (s, 3H), 2.89 (dd, J = 9.2, 5.7 Hz, 2H), 2.78 (dd, J = 9.8, 5.7 Hz, 2H), 1.51 (s, 9H).

^{13}C NMR (101 MHz, Acetone-d_6) δ 159.4, 158.0, 153.6, 139.8, 135.9, 131.3, 130.9, 124.6, 122.6, 121.1, 118.2, 107.4, 99.7, 55.6, 36.8, 31.1, 28.5, 28.5.

HRMS m/z: $[M–H]^–$ calculated for $[C_{20}H_{23}BrNO_4]^–$ 420.0816, found 420.0800, Δ = –3.7 ppm.

***tert*-Butyl 3-bromo-4-methylbenzoate (S19)**

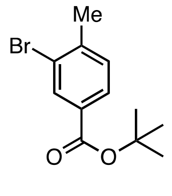

3-Bromo-4-methylbenzoic acid (1.63 g, 7.58 mmol, 1.00 equiv.) was suspended in CH_2Cl_2 (12 mL). DMAP (232 mg, 1.90 mmol, 0.250 equiv.) was added, followed by di-*tert*-butyl decarbonate (1.99 g, 9.10 mmol, 1.20 equiv.). The reaction was stirred at room temperature for 17 h, then quenched with the addition of water. The reaction mixture was extracted three times with CH_2Cl_2, dried with $MgSO_4$, filtered, and the solvent removed under reduced pressure. The crude product was purified with flash column chromatography (10% EtOAc in petroleum ether) to afford the title compound as a colourless liquid (847 mg, 3.12 mmol, 41%).

^{1}H NMR (400 MHz, $CDCl_3$) δ 8.12 (d, *J* = 1.8 Hz, 1H), 7.81 (dd, *J* = 7.9, 1.8 Hz, 1H), 7.26 (dd, *J* = 7.9, 0.7 Hz, 1H), 2.43 (d, *J* = 0.7 Hz, 3H), 1.58 (s, 9H).

^{13}C NMR (101 MHz, $CDCl_3$) δ 164.6, 142.8, 133.4, 131.5, 130.6, 128.4, 124.7, 81.5, 28.3, 23.3.

Data in agreement with literature.[15]

(2-bromo-4-(*tert*-butoxycarbonyl)benzyl)Triphenylphosphonium bromide (S20)

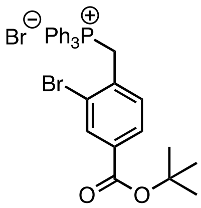

Benzylic Bromination: *N*-Bromo succinimide (756 mg, 4.25 mmol, 1.10 equiv.) and AIBN (64 mg, 0.39 mmol, 10 mol%) were added to a stirred solution of *tert*-butyl 3-bromo-4-methylbenzoate (1.05 g, 3.86 mmol, 1.00 equiv.) in 1,2-dichloroethane (40 mL). The reaction mixture was heated to 85 °C for 2 hours, then allowed to cool to room temperature. The 1,2-dichloroethane was removed under reduced pressure, and the crude reaction mixture passed through a silica plug (10% Et_2O in petroleum ether). The Et_2O/petroleum ether was removed under reduced pressure. The crude product was subjected to the next step without further purification.

Phosphonium Salt Synthesis: Preparation according to General Procedure A afforded the title compound as a colorless solid (1.19 g, 1.95 mmol, 51% yield over two steps).

^{1}H NMR (400 MHz, $CDCl_3$) δ 7.87 (s, 1H), 7.78 – 7.70 (m, 3H), 7.68 – 7.54 (m, 14H), 5.59 (d, *J* = 14.8 Hz, 2H), 1.47 (d, *J* = 1.4 Hz, 9H).

^{13}C NMR (101 MHz, $CDCl_3$) δ 163.5, 135.3 (d, *J* = 3.2 Hz), 134.2 (d, *J* = 10.0 Hz), 133.6 (d, *J* = 3.7 Hz), 133.4 (d, *J* = 3.3 Hz), 133.0 (d, *J* = 5.0 Hz), 132.0 (d, *J* = 9.0 Hz), 130.3 (d, *J* = 12.8

Hz), 128.9 (d, J = 3.5 Hz), 126.9 (d, J = 6.4 Hz), 116.9 (d, J = 86.1 Hz), 82.1, 30.9 (d, J = 48.7 Hz), 28.0.

^{31}P NMR (162 MHz, $CDCl_3$) δ 22.6.

HRMS m/z: The compound did not ionize.

***tert*-Butyl 3-bromo-4-(4-hydroxy-2-methoxyphenethyl)benzoate (1p)**

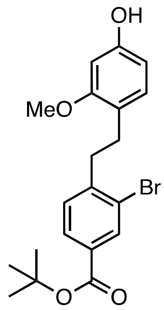

The title compound was prepared according to General Route 1. General Procedure B was performed with (2-bromo-4-(*tert*-butoxycarbonyl)benzyl)triphenylphosphonium bromide (**S20**) (992 mg, 1.62 mmol) and 4-(benzyloxy)-2-methoxybenzaldehyde (**S4**) (328 mg, 1.35 mmol). General Procedure C was performed with the alkene (495 mg, 1.00 mmol). The crude product was purified by flash column chromatography (10 – 30% EtOAc in petroleum ether) to afford the title compound as a viscous oil (338 mg, 0.83 mmol, 61% over two steps).

^{1}H NMR (400 MHz, $CDCl_3$) δ 8.16 (d, J = 1.7 Hz, 1H), 7.82 (dd, J = 8.0, 1.7 Hz, 1H), 7.18 (d, J = 8.0 Hz, 1H), 6.90 (d, J = 8.0 Hz, 1H), 6.45 (d, J = 2.3 Hz, 1H), 6.38 (dd, J = 8.0, 2.3 Hz, 1H), 6.32 (s, 1H), 3.75 (s, 3H), 3.01 (dd, J = 9.4, 6.1 Hz, 2H), 2.84 (dd, J = 9.4, 6.2 Hz, 2H), 1.62 (s, 9H).

^{13}C NMR (101 MHz, $CDCl_3$) δ 165.2, 158.5, 155.6, 146.7, 133.7, 131.1, 130.5, 130.4, 128.3, 124.3, 121.2, 106.8, 99.0, 82.0, 55.3, 36.8, 29.9, 28.2.

HRMS $[M–H]^-$ calculated for $[C_{20}H_{22}{}^{81}BrO_4]^-$ 407.0687, found 407.0674. Δ = – 3.2 ppm.

(2-bromo-3-methylphenyl)Methanol (S21)

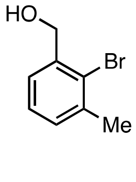

2-Bromo-3-methylbenzoic acid (4.30 g, 20.0 mmol, 1.00 equiv.) was dissolved in anhydrous THF (20 mL). The solution was cooled to 0 °C, and BH_3•THF solution (1.00 M, 60.0 mL, 60.0 mmol, 3.00 equiv.) was added slowly under N_2 atmosphere. The reaction was warmed to room temperature and stirred for 16 h. The reaction mixture was cooled back down to 0 °C. Sat. K_2CO_3 (aq.) was added dropwise until effervescence ceased, followed by an excess of H_2O. The aqueous layer was extracted with EtOAc (3×). The combined organic extracts were dried over $MgSO_4$, filtered, and the solvent removed under reduced pressure. The crude product was purified by flash column chromatography (5-20% EtOAc in petroleum ether) to afford the title compound as a colorless solid (2.26 g. 11.2 mmol, 56%).

^{1}H NMR (400 MHz, $CDCl_3$) δ 7.33 – 7.28 (m, 1H), 7.25 – 7.17 (m, 2H), 4.76 (s, 2H), 2.43 (s, 3H), 2.04 (s, 1H).

^{13}C NMR (101 MHz, $CDCl_3$) δ 140.2, 138.7, 130.2, 127.4, 126.5, 125.3, 65.9, 23.5.

Data in agreement with literature.[16]

(2-bromo-3-methylbenzyl)Triphenylphosphonium bromide (S22)

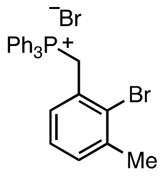

(2-bromo-3-methylphenyl)Methanol (**S21**) (2.61 g, 13.0 mmol, 1.00 equiv.) was dissolved in anhydrous CH_2Cl_2 (50 mL). PBr_3 (7.04 g, 2.47 mL, 26.0 mmol, 2.00 equiv.) was added dropwise under nitrogen at room temperature. The reaction mixture was stirred for 16 h. Upon completion, the reaction was cooled to 0 °C and quenched with water. The aqueous phase was extracted with Et_2O (3×). The combined organic extracts were dried over $MgSO_4$, filtered and the solvent removed under reduced pressure. The crude product (2.94 g, 11.1 mmol) was carried forward to General Procedure A, without further purification, to afford the title compound as a colorless solid (4.97 g, 9.44 mmol, 73%).

^{1}H NMR (400 MHz, $CDCl_3$) δ 7.83 – 7.74 (m, 3H), 7.73 – 7.55 (m, 12H), 7.37 (dt, J = 7.6, 2.3 Hz, 1H), 7.15 (dt, J = 7.6, 2.3 Hz, 1H), 7.07 (td, J = 7.6, 0.9 Hz, 1H), 5.69 (d, J = 14.2 Hz, 2H), 2.20 (s, 3H).

^{13}C NMR (101 MHz, $CDCl_3$) δ 139.3 (d, J = 3.1 Hz), 135.3 (d, J = 3.0 Hz), 134.4 (d, J = 9.9 Hz), 131.1 (d, J = 4.1 Hz), 130.3 (d, J = 5.3 Hz), 130.2 (d, J = 12.6 Hz), 129.9 (d, J = 6.8 Hz), 127.9 – 127.8 (m), 117.4 (d, J = 85.7 Hz), 31.8 (d, J = 48.3 Hz), 24.1.

^{31}P NMR (162 MHz, $CDCl_3$) δ 22.7.

HRMS m/z: $[M]^+$ calculated for $[C_{26}H_{23}BrP]^+$ 445.0715, found 445.0719. Δ = +0.9 ppm.

1-(4-(benzyloxy)-2-methoxyphenethyl)-2-Bromo-3-methylbenzene (1q)

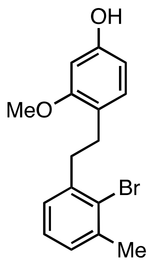

The title compound was prepared according to General Route 1. General produced B was performed with (2-bromo-3-methylbenzyl)triphenylphosphonium bromide (**S22**) (4.97 g, 9.44 mmol) and 4-(benzyloxy)-2-methoxybenzaldehyde (**S4**) (1.91g, 7.87 mmol). General Procedure C was performed with the alkene (818 mg, 2.00 mmol). The crude product was purified by flash column chromatography (10-20% EtOAc in petroleum ether) to afford the title compound as a colorless solid (178 mg, 0.55 mmol, 7% over two steps).

^{1}H NMR (500 MHz, $CDCl_3$) δ 7.12 – 7.06 (m, 2H), 7.01 (dd, J = 6.4, 2.9 Hz, 1H), 6.96 (d, J = 8.0 Hz, 1H), 6.41 (d, J = 2.4 Hz, 1H), 6.33 (dd, J = 8.0, 2.4 Hz, 1H), 4.74 (s, 1H), 3.79 (s, 3H), 3.07 – 2.94 (m, 2H), 2.90 – 2.79 (m, 2H), 2.44 (s, 3H).

^{13}C NMR (126 MHz, $CDCl_3$) δ 158.7, 155.1, 142.2, 138.5, 130.4, 128.5, 128.0, 127.3, 126.8, 122.5, 106.6, 98.9, 55.5, 37.6, 30.2, 24.2.

HRMS m/z: $[M–H]^-$ calculated for $[C_{16}H_{16}BrO_2]^-$ 319.0339, found 319.0332. Δ = –2.1 ppm.

(2-bromo-5-fluorobenzyl)Triphenylphosphonium bromide (S23)

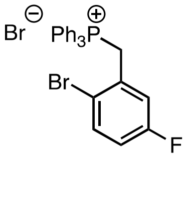

Preparation according to General Procedure A from 2-bromo-5-fluorobenzyl bromide (4.02 g, 15.0 mmol) afforded the title compound as a colorless solid (7.00 g, 13.2 mmol, 88% yield).

^{1}H NMR (500 MHz, MeCN-d_3) δ 7.96 – 7.81 (m, 3H), 7.69 (td, *J* = 8.1, 3.7 Hz, 6H), 7.61 (ddd, *J* = 12.8, 8.1, 1.4 Hz, 6H), 7.51 (ddd, *J* = 8.9, 5.4, 0.9 Hz, 1H), 7.13 – 7.01 (m, 1H), 7.00 – 6.92 (m, 1H), 4.93 (d, *J* = 14.5 Hz, 2H).

^{13}C NMR (101 MHz, DMSO-d_6) δ 160.9 (dd, *J* = 246.5, 3.9 Hz), 135.5 (d, *J* = 3.1 Hz), 135.1 (dd, *J* = 8.5, 2.9 Hz), 134.1 (d, *J* = 10.2 Hz), 130.3 (d, *J* = 12.6 Hz), 130.1 – 129.9 (m), 121.29 – 120.5 (m), 119.2 (dd, *J* = 24.2, 4.7 Hz), 118.3 – 118.0 (m), 116.8 (d, *J* = 85.6 Hz), 29.3 (d, *J* = 48.7 Hz).

^{19}F NMR (376 MHz, MeCN-d_3) δ -114.3 (d, *J* = 2.8 Hz).

^{31}P NMR (162 MHz, MeCN-d_3) δ 22.4 (d, *J* = 3.0 Hz).

HRMS m/z: $[M]^+$ calculated for $[C_{25}H_{20}BrFP]^+$ 449.0465, found 449.0471. Δ = +1.5 ppm.

4-(2-bromo-5-fluorophenethyl)-3-Methoxyphenol (1r)

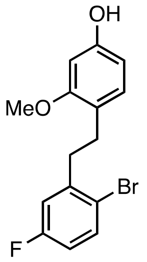

The title compound was prepared according to General Route 1. General Procedure B was performed using (2-bromo-5-fluorobenzyl)triphenylphosphonium bromide (**S23**) (3.18 g, 6.00 mmol) and 4-(benzyloxy)-2-methoxybenzaldehyde (**S4**) (1.21 g, 5.00 mmol). General Procedure C was performed with the alkene (818 mg, 1.98 mmol). The crude product was purified by flash column chromatography (20% EtOAc in petroleum ether) to afford the title compound as a colorless solid (546 mg, 1.68 mmol, 34% over two steps).

^{1}H NMR (400 MHz, $CDCl_3$) δ 7.47 (dd, *J* = 8.8, 5.4 Hz, 1H), 6.92 (d, *J* = 8.0 Hz, 1H), 6.88 (dd, *J* = 9.5, 3.1 Hz, 1H), 6.78 (ddd, *J* = 8.8, 8.0, 3.1 Hz, 1H), 6.41 (d, *J* = 2.4 Hz, 1H), 6.32 (dd, *J* = 8.0, 2.4 Hz, 1H), 4.77 (s, 1H), 3.79 (s, 3H), 3.00 – 2.90 (m, 2H), 2.86 – 2.75 (m, 2H).

^{13}**C NMR** (101 MHz, $CDCl_3$) δ 162.0 (d, J = 246.0 Hz), 158.7, 155.3, 143.9 (d, J = 7.4 Hz), 133.7 (d, J = 8.2 Hz), 130.5, 121.8, 118.6 (d, J = 3.2 Hz), 117.4 (d, J = 22.2 Hz), 114.6 (d, J = 22.5 Hz), 106.6, 99.0, 55.4, 36.8 (d, J = 1.6 Hz), 29.9.

^{19}**F NMR** (376 MHz, $CDCl_3$) δ -115.6.

HRMS m/z: $[M+H]^+$ calculated for $[C_{15}H_{15}BrFO_2]^+$ 325.0234, found 325.0249. Δ = +4.6 ppm.

2-Bromo-4-fluorobenzyl triphenylphosphonium bromide (S24)

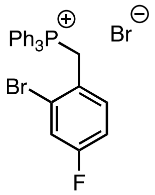

Preparation according to General Procedure A from 2-bromo-4-fluorobenzyl bromide (4.02 g, 15.0 mmol) afforded the title compound as a colorless solid (7.95 g, 15.0 mmol, quant.).

^{1}**H NMR** (400 MHz, MeCN-d_3) δ 7.89 (td, J = 7.5, 1.9 Hz, 3H), 7.74 – 7.59 (m, 12H), 7.33 (dd, J = 8.5, 2.8 Hz, 1H), 7.26 (ddd, J = 8.5, 5.8, 2.8 Hz, 1H), 7.03 (td, J = 8.5, 2.8 Hz, 1H), 5.05 (d, J = 14.1 Hz, 2H).

^{13}**C NMR** (101 MHz, MeCN-d_3) δ 163.3 (dd, J = 251.7, 4.2 Hz), 136.4 (d, J = 3.1 Hz), 135.3 (d, J = 10.0 Hz), 134.5 (dd, J = 9.0, 4.8 Hz), 131.2 (d, J = 12.8 Hz), 127.9 (dd, J = 9.9, 6.5 Hz), 124.9 (dd, J = 8.5, 3.7 Hz), 121.5 (dd, J = 25.1, 3.1 Hz), 117.4, 116.6 (dd, J = 21.7, 3.4 Hz), 30.9 (d, J = 50.2 Hz).

^{19}**F NMR** (376 MHz, MeCN-d_3) δ -111.4 (d, J = 6.7 Hz).

^{31}**P NMR** (162 MHz, MeCN-d_3) δ 22.1 (d, J = 6.7 Hz).

HRMS m/z: $[M]^+$ calculated for $[C_{25}H_{20}BrFP]^+$ 449.0465, found 449.0465. Δ = 0.0 ppm.

4-(2-bromo-4-fluorophenethyl)-3-Methoxyphenol (1s)

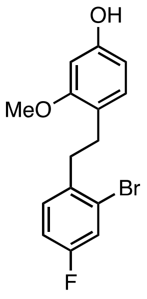

The title compound was prepared according to General Route 1. General Procedure B was performed using 2-bromo-4-fluorobenzyl triphenylphosphonium bromide (**S24**) (3.18 g, 6.0 mmol) and 4-(benzyloxy)-2-methoxybenzaldehyde (**S4**) (1.21g, 5.00 mmol). General Procedure C was performed with the alkene (827 mg, 2.00 mmol). The crude product was purified by flash column chromatography (10-20% EtOAc in petroleum ether) to afford the title compound as a colorless solid (487 mg, 1.50 mmol, 30% over two steps).

^{1}H NMR (400 MHz, $CDCl_3$) δ 7.28 (dd, J = 8.4, 2.7 Hz, 1H), 7.07 (dd, J = 8.4, 6.1 Hz, 1H), 6.94 – 6.87 (m, 2H), 6.40 (d, J = 2.4 Hz, 1H), 6.31 (dd, J = 8.0, 2.4 Hz, 1H), 4.76 (s, 1H), 3.78 (s, 3H), 2.99 – 2.89 (m, 2H), 2.84 – 2.76 (m, 2H).

^{13}C NMR (101 MHz, $CDCl_3$) δ 160.8 (d, J = 248.0 Hz), 158.7, 155.2, 137.5, 131.3 (d, J = 8.2 Hz), 130.5, 124.3 (d, J = 9.6 Hz), 121.9, 119.8 (d, J = 24.3 Hz), 114.3 (d, J = 20.8 Hz), 106.6, 98.9, 55.4, 35.9, 30.2 (d, J = 1.3 Hz).

^{19}F NMR (376 MHz, $CDCl_3$) δ -116.6.

HRMS m/z: $[M–H]^-$ calculated for $[C_{15}H_{13}BrFO_2]^-$ 323.0088, found 323.0084. Δ = –1.4 ppm.

Synthesis of Starting Materials in Scheme 2

General Procedure F: Synthesis of *para*–Amino Phenol Starting Materials *via* Reductive Amination

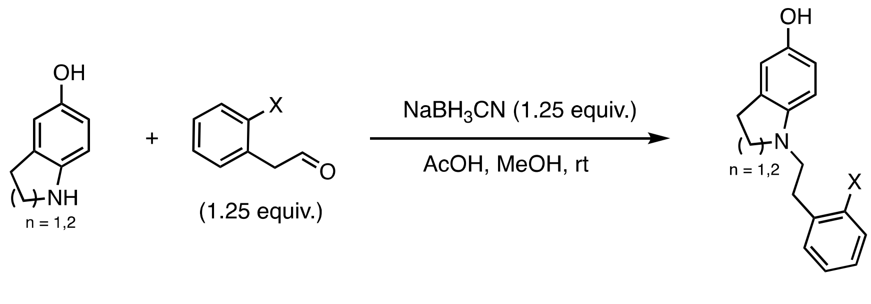

Adapted from the procedure reported by You.[17] Reaction was conducted in a flask, open to air. To a 0.4 M solution of secondary amine (1.00 equiv.) in methanol was added a 0.17 M solution of aldehyde (1.25 equiv.) in methanol. $NaBH_3CN$ (1.25 equiv.) was added, followed by AcOH (0.1 ml/mmol of amine starting material). The reaction was stirred at room temperature and monitored by TLC (approx. 1 h). Upon completion, the reaction was quenched with sat. $NaHCO_3$ (aq.) and concentrated under a stream of air to remove methanol. Water was added and the mixture was extracted with EtOAc (3×). The combined organic extracts were washed with brine, dried over Na_2SO_4, concentrated under reduced pressure, and purified by flash column chromatography.

General Procedure G: Wittig Homologation of Substituted 2-Bromobenzaldehydes

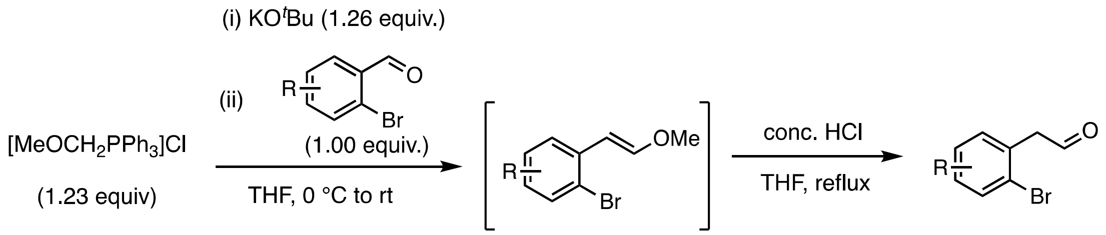

Step 1: Adapted from the procedure reported by Fan.[18] A heat gun-dried two-necked round bottom flask was charged with $MeOCH_2PPh_3Cl$ (5.69 g, 16.6 mmol, 1.23 equiv.) and dry THF (35 ml) under N_2. The solution was cooled to 0 °C, and KO^{t}Bu (1.90 g, 17.0 mmol, 1.26 equiv.) was added over 20 minutes. The reaction for 45 minutes at 0 °C, after which the substituted 2-bromobenzaldehyde (13.5 mmol, 1.00 equiv.) was added over 10 minutes. The reaction was warmed to room temperature and stirred until the substituted 2-bromobenzaldehyde aldehyde was consumed, as judged by ^1H NMR (approx. 3 h). Upon completion, sat. NH_4Cl (aq.) was added slowly, and the reaction stirred for a further 15 minutes. The reaction mixture was diluted with Et_2O and the layers were separated. The aqueous layer was extracted with Et_2O (1×). The combined organic extracts were washed with brine, dried over Na_2SO_4, and concentrated under reduced pressure. The crude product was subjected to the next step without further purification.

Step 2: The crude product was dissolved in THF (35 ml), and conc. HCl (4 ml) was added. The reaction was refluxed at 70 °C for 4 h. The reaction mixture was diluted with Et_2O, washed with sat. $NaHCO_3$ (aq.), dried over Na_2SO_4, concentrated under reduced pressure, and purified by flash column chromatography.

Indolin-5-ol (S25)

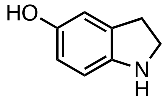

Adapted from the procedure reported by Stanley.[19] To a solution of 5-hydroxyindole (3.00 g, 22.5 mmol, 1.00 equiv.) in acetic acid (55 ml) was added $NaBH_3CN$ (3.54 g, 56.3 mmol, 2.50 equiv.). The reaction was stirred for 1 h at room temperature. Upon completion, the reaction was diluted with H_2O (50 ml), brought to pH 9 by addition of KOH pellets, and extracted with EtOAc (3×). The combined organic layers were washed with H_2O and brine, dried over Na_2SO_4, and concentrated under reduced pressure. Soluble impurities were removed by trituration with EtOAc. The residue was dried under reduced pressure, to afford the title compound as a brown solid (1.39 g, 10.3 mmol, 46% yield).

^{1}H NMR (400 MHz, MeOD) δ 6.65 (1H, d, *J*=2.5 Hz), 6.61 (1H, d, *J*=8.3 Hz), 6.50 (1H, dd, *J*=8.3, 2.5 Hz), 3.42 (2H, t, *J*=8.2 Hz), 3.33 (1H, q, *J*=2.0, 1.3 Hz), 2.96 (2H, t, *J*=8.2 Hz).

^{13}C NMR (101 MHz, MeOD) δ 152.5, 144.7, 133.3, 114.5, 113.1, 113.1, 48.3, 31.5.

HRMS m/z: $[M+H]^+$ calculated for $[C_8H_{10}NO]^+$ 136.0757, found 136.0753. Δ = –2.9 ppm.

2-(2-bromophenyl)Acetaldehyde (S26)

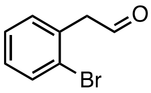

A solution of 2-(2-bromophenyl)ethan-1-ol (3.62 g, 18.0 mmol, 1.00 equiv.) in CH_2Cl_2 (60 ml) was cooled to 0 °C under N_2. Dess–Martin periodinane (9.33 g, 22.0 mmol, 1.22 equiv.) was added in portions. The reaction was warmed to room temperature and stirred for 2 h. Upon completion, the reaction was quenched with sat. $NaHCO_3$ (aq.) (60 ml) and filtered, washing the residue with CH_2Cl_2. The organic layer was separated, and the aqueous layer was extracted with CH_2Cl_2 (3×). The combined organic extracts were dried over Na_2SO_4, concentrated under reduced pressure, and purified by flash column chromatography (5% EtOAc in petroleum ether) to afford the title compound as a yellow oil (2.31 g, 11.6 mmol, 64% yield).

^{1}H NMR (400 MHz, $CDCl_3$) δ 9.76 (1H, t, *J*=1.8 Hz), 7.61 (1H, d, *J*=7.6 Hz), 7.31 (1H, td, *J*=7.6, 1.3 Hz), 7.24 (1H, dd, *J*=7.6, 1.8 Hz), 7.18 (1H, td, *J*=7.6, 1.8 Hz), 3.86 (2H, d, *J*=1.8 Hz).

^{13}C NMR (101 MHz, $CDCl_3$) δ 198.4, 133.1, 132.7, 131.9, 129.4, 128.0, 125.1, 50.6.

Data in agreement with the literature.[20]

1-(2-bromophenethyl)Indolin-5-ol (3a)

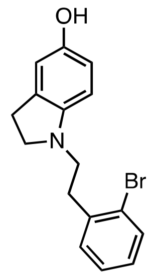

Prepared according to General Procedure F from indolin-5-ol (**S25**) (270 mg, 2.00 mmol) and 2-(2-bromophenyl)acetaldehyde (**S26**) (498 mg, 2.50 mmol). Purification by flash column chromatography (20-50% EtOAc in petroleum ether) afforded the title compound as a brown solid (336 mg, 1.06 mmol, 53% yield).

^{1}H NMR (500 MHz, Acetone-d_6) δ 7.59 (1H, d, J=7.7 Hz), 7.50 – 7.39 (1H, m), 7.32 (1H, t, J=7.7 Hz), 7.16 (1H, td, J=7.7, 1.7 Hz), 6.63 (1H, d, J=2.5 Hz), 6.52 (1H, dd, J=8.3, 2.5 Hz), 6.42 (1H, d, J=8.3 Hz), 3.35 (2H, t, J=8.1 Hz), 3.23 (2H, dd, J=9.4, 6.2 Hz), 3.03 (2H, dd, J=9.4, 6.2 Hz), 2.89 – 2.79 (2H, m).

^{13}C NMR (126 MHz, Acetone-d_6) δ 150.9, 146.4, 140.4, 133.5, 132.2, 132.1, 129.0, 128.7, 124.9, 113.8, 113.4, 108.6, 54.4, 51.1, 34.2, 29.5.

Data in agreement with the literature.[17]

2-(2-chlorophenyl)Acetaldehyde (S27)

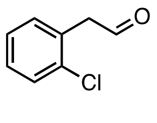

A solution of 2-(2-chlorophenyl)ethan-1-ol (2.82 g, 18.0 mmol, 1.00 equiv.) in CH_2Cl_2 (60 ml) was cooled to 0 °C under N_2. Dess–Martin periodinane (9.33 g, 22.0 mmol, 1.22 equiv.) was added in portions. The reaction was warmed to room temperature and stirred for 2 h. Upon completion, the reaction was quenched with sat. $NaHCO_3$ (aq.) (60 ml) and filtered, washing the residue with CH_2Cl_2. The organic layer was separated, and the aqueous layer was extracted with CH_2Cl_2 (3×). The combined organic extracts were dried over Na_2SO_4, concentrated under reduced pressure, and purified by flash column chromatography (5% EtOAc in petroleum ether) to afford the title compound as a yellow oil (1.09 g, 7.05 mmol, 39% yield).

^{1}H NMR (400 MHz, $CDCl_3$) δ 9.75 (1H, s), 7.48 – 7.37 (1H, m), 7.32 – 7.17 (3H, m), 3.84 (2H, t, *J*=1.4 Hz).

^{13}C NMR (101 MHz, $CDCl_3$) δ 198.3, 134.7, 131.8, 130.9, 129.8, 129.2, 127.3, 48.3.

Data in agreement with the literature.[17]

1-(2-chlorophenethyl)Indolin-5-ol (3a')

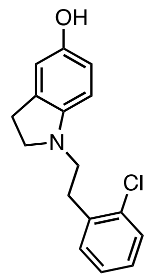

Prepared according to General Procedure F from indolin-5-ol (**S25**) (270 mg, 2.00 mmol) and 2-(2-chlorophenyl)acetaldehyde (**S27**) (388 mg, 2.50 mmol). Purification by flash column chromatography (20% EtOAc in petroleum ether) afforded the title compound as a yellow oil (292 mg, 1.07 mmol, 53% yield).

^{1}H NMR (500 MHz, Acetone-*d_6*) δ 7.49 (1H, s), 7.41 (2H, ddd, *J*=9.4, 7.3, 1.9 Hz), 7.30 – 7.19 (2H, m), 6.63 (1H, d, *J*=2.5 Hz), 6.53 (1H, dd, *J*=8.3, 2.5 Hz), 6.40 (1H, d, *J*=8.3 Hz), 3.33 (2H, t, *J*=8.1 Hz), 3.23 (2H, dd, *J*=9.3, 6.2 Hz), 3.02 (2H, dd, *J*=9.3, 6.2 Hz), 2.84 (2H, t, *J*=8.1 Hz).

^{13}C NMR (500 MHz, Acetone-*d_6*) δ 150.9, 146.4, 138.6, 134.4, 132.2, 132.1, 130.2, 128.8, 128.0, 113.8, 113.4, 108.6, 54.3, 51.0, 31.7, 29.5.

Data in agreement with the literature.[17]

1-(2-bromophenethyl)-1,2,3,4-Tetrahydroquinolin-6-ol (3b)

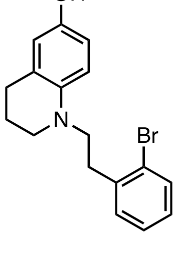

Prepared according to General Procedure F from 1,2,3,4-tetrahydroquinolin-6-ol (298 mg, 2.00 mmol) and 2-(2-bromophenyl)acetaldehyde (**S26**) (498 mg, 2.50 mmol). Purification by flash column chromatography (20-30% EtOAc in petroleum ether) afforded the title compound as a red solid (524 mg, 1.58 mmol, 79% yield).

^{1}H NMR (400 MHz, Acetone-d_6) δ 7.58 (1H, dd, *J*=7.6, 1.3 Hz), 7.38 (1H, dd, *J*=7.6, 1.8 Hz), 7.32 (1H, td, *J*=7.6, 1.3 Hz), 7.16 (1H, td, *J*=7.6, 1.8 Hz), 6.65 (1H, d, *J*=8.7 Hz), 6.55 (1H, dd, *J*=8.7, 2.9 Hz), 6.46 (1H, d, *J*=2.9 Hz), 3.48 – 3.40 (2H, m), 3.21 (2H, t, *J*=5.5 Hz), 3.00 (2H, t, *J*=7.7 Hz), 2.66 (2H, t, *J*=6.3 Hz), 1.87 (2H, p, *J*=6.3 Hz).

^{13}C NMR (126 MHz, Acetone-d_6) δ 149.0, 140.4, 139.3, 133.5, 132.3, 129.0, 128.7, 124.9, 124.6, 117.0, 114.5, 113.3, 52.8, 50.1, 33.1, 28.9, 23.3.

Data in agreement with the literature.[17]

2-(2-bromo-5-methoxyphenyl)Acetaldehyde (S28)

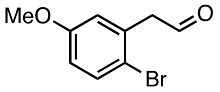

Prepared according to General Procedure G with 2-bromo-5-methoxybenzaldehyde (2.90 g, 13.5 mmol). Purification by flash column chromatography (5-10% EtOAc in petroleum ether) afforded the title compound as a yellow oil (636 mg, 2.78 mmol, 21% yield).

^{1}H NMR (400 MHz, $CDCl_3$,) δ 9.73 (1H, t, *J*=1.8 Hz), 7.48 (1H, d, *J*=8.7 Hz), 6.77 (1H, d, *J*=3.0 Hz), 6.74 (1H, dd, *J*=8.7, 3.0 Hz), 3.81 (2H, d, *J*=1.8 Hz), 3.78 (3H, s).

^{13}C NMR (101 MHz, $CDCl_3$) δ 198.3, 159.3, 133.7, 133.5, 117.5, 115.4, 115.0, 55.6, 50.8.

Data in agreement with the literature.[21]

1-(2-bromo-5-methoxyphenethyl)Indolin-5-ol (3c)

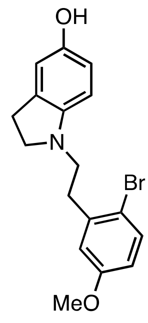

Prepared according to General Procedure F from indolin-5-ol (**S25**) (135 mg, 1.00 mmol) and 2-(2-bromo-5-methoxyphenyl)acetaldehyde (**S28**) (229 mg, 1.25 mmol). Purification by flash column chromatography (20-30% EtOAc in petroleum ether) afforded the title compound as an orange solid (201 mg, 0.58 mmol, 58% yield).

^{1}H NMR (400 MHz, Acetone-d_6) δ 7.49 – 7.43 (2H, m), 7.02 (1H, d, *J*=3.1 Hz), 6.75 (1H, dd, *J*=8.8, 3.1 Hz), 6.63 (1H, s), 6.52 (1H, d, *J*=8.0 Hz), 6.43 (1H, d, *J*=8.0 Hz), 3.78 (3H, s), 3.35 (2H, t, *J*=8.1 Hz), 3.23 (2H, dd, *J*=9.4, 6.2 Hz), 2.98 (2H, dd, *J*=9.4, 6.2 Hz), 2.85 (2H, t, *J*=8.1 Hz).

^{13}C NMR (101 MHz, Acetone-d_6) δ 160.4, 151.0, 146.5, 141.4, 134.2, 132.3, 117.7, 115.2, 114.8, 113.9, 113.5, 108.8, 55.9, 54.4, 51.2, 34.5, 29.6.

Data in agreement with the literature.[17]

2-(2-bromo-5-chlorophenyl)Acetaldehyde (S29)

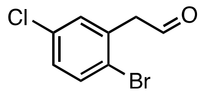

Prepared according to General Procedure G with 2-bromo-5-chlorobenzaldehyde (2.96 g, 13.5 mmol). Purification by flash column chromatography (5-7.5% EtOAc in petroleum ether) afforded the title compound as a yellow solid (800 mg, 3.43 mmol, 25% yield).

^{1}H NMR (500 MHz, $CDCl_3$) δ 9.77 – 9.74 (1H, m), 7.53 (1H, dd, *J*=8.6, 2.3 Hz), 7.23 (1H, d, *J*=2.3 Hz), 7.17 (1H, dt, *J*=8.6, 2.3 Hz), 3.84 (2H, d, *J*=1.6 Hz).

^{13}C NMR (126 MHz, $CDCl_3$) δ 197.3, 134.5, 134.1, 133.9, 131.7, 129.5, 122.9, 50.4.

Data in agreement with the literature.[22]

1-(2-bromo-5-chlorophenethyl)Indolin-5-ol (3d)

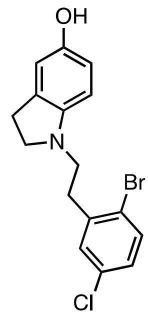

Prepared according to General Procedure F from indolin-5-ol (**S25**) (135 mg, 1.00 mmol) and 2-(2-bromo-5-chlorophenyl)acetaldehyde (**S29**) (291 mg, 1.25 mmol). Purification by flash column chromatography (10-20% EtOAc in petroleum ether) afforded the title compound as a yellow solid (226 mg, 0.64 mmol, 64% yield).

^{1}H NMR (400 MHz, Acetone-d_6) δ 7.60 (1H, d, *J*=8.5 Hz), 7.50 (1H, d, *J*=2.6 Hz), 7.45 (1H, s), 7.20 (1H, dd, *J*=8.5, 2.6 Hz), 6.62 (1H, d, *J*=2.4 Hz), 6.52 (1H, dd, *J*=8.4, 2.4 Hz), 6.44 (1H, d, *J*=8.4 Hz), 3.35 (2H, t, *J*=8.2 Hz), 3.26 (2H, dd, *J*=9.2, 6.2 Hz), 3.03 (2H, dd, *J*=9.2, 6.2 Hz), 2.85 (2H, t, *J*=8.2 Hz).

^{13}C NMR (101 MHz, Acetone-d_6) δ 151.0, 146.3, 142.6, 134.9, 133.9, 132.2, 131.8, 129.0, 123.1, 113.9, 113.4, 108.7, 54.4, 50.8, 34.1, 29.5.

HRMS m/z: $[M+H]^+$ calculated for $[C_{16}H_{16}BrClNO]^+$ 352.0098, found 352.0082. Δ = –4.5 ppm.

2-(2-bromo-5-(trifluoromethyl)phenyl)Acetaldehyde (S30)

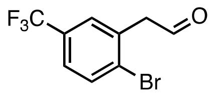

Prepared according to General Procedure G with 2-bromo-5-trifluoromethylbenzaldehyde (3.42 g, 13.5 mmol). Purification by flash column chromatography (5-7.5% EtOAc in petroleum ether) afforded the title compound as a yellow oil (1.17 g, 4.38 mmol, 32% yield).

^{1}H NMR (500 MHz, $CDCl_3$) δ 9.79 (1H, t, *J*=1.4 Hz), 7.74 (1H, d, *J*=8.3 Hz), 7.49 (1H, d, *J*=2.2 Hz), 7.44 (1H, dd, *J*=8.3, 2.2 Hz), 3.95 (2H, d, *J*=1.4 Hz).

^{13}C NMR (126 MHz, $CDCl_3$) δ 196.9, 134.0, 133.7, 130.5 (q, *J*=33.0 Hz), 129.0 (q, *J*=1.5 Hz), 128.6 (q, *J*=3.7 Hz), 126.1 (q, *J*=3.7 Hz), 123.7 (q, *J*=272.2), 50.5.

Data in agreement with the literature.[23]

1-(2-bromo-5-(trifluoromethyl)phenethyl)Indolin-5-ol (3e)

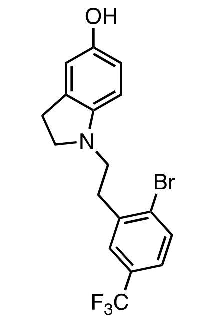

Prepared according to General Procedure F from indolin-5-ol (**S25**) (135 mg, 1.00 mmol) and 2-(2-bromo-5-(trifluoromethyl)phenyl)acetaldehyde (**S30**) (334 mg, 1.25 mmol). Purification by flash column chromatography (10-20% EtOAc in petroleum ether) afforded the title compound as a yellow solid (267 mg, 0.69 mmol, 69% yield).

^{1}H NMR (400 MHz, Acetone-d_6) δ 7.84 (1H, d, J=8.3 Hz), 7.80 (1H, d, J=2.3 Hz), 7.50 (1H, dd, J=8.3, 2.3 Hz), 7.46 (1H, s), 6.63 (1H, d, J=2.5 Hz), 6.52 (1H, dd, J=8.3, 2.5 Hz), 6.44 (1H, d, J=8.3 Hz), 3.36 (2H, t, J=8.1 Hz), 3.30 (2H, dd, J=9.1, 6.1 Hz), 3.14 (2H, dd, J=9.1, 6.1 Hz), 2.85 (2H, t, J=8.1 Hz).

^{13}C NMR (101 MHz, Acetone-d_6) δ 151.0, 146.3, 142.0, 134.5, 132.2, 130.4 (q, J=32.5 Hz), 129.2, 128.7 (q, J=3.7 Hz), 125.6 (q, J=3.7 Hz), 125.0 (q, J=276.6), 113.8, 113.4, 108.7, 54.4, 50.8, 34.2, 29.5.

Data in agreement with the literature.[17]

2-(2-bromo-4,5-dimethoxyphenyl)Acetaldehyde (S31)

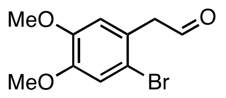

Prepared according to a modification of General Procedure G with 2-bromo-4,5-dimethoxybenzaldehyde (3.31 g, 13.5 equiv.), 1.85 equiv. $MeOCH_2PPh_3Cl$ (8.58 g, 25.0 mmol), and 1.89 equiv. KO^{t}Bu (2.86 g, 25.5 mmol). Purification by flash column chromatography (5-10% EtOAc in petroleum ether) afforded the title compound as a yellow solid (539 mg, 2.08 mmol, 15% yield). N.B. The product contained 20% an unidentified impurity, which was removed in the next step.

^{1}H NMR (400 MHz, $CDCl_3$) δ 9.71 (1H, t, *J*=1.8 Hz), 7.06 (1H, s), 6.69 (1H, s), 3.86 (3H, s), 3.84 (3H, s), 3.77 (2H, d, *J*=1.8 Hz).

^{13}C NMR (101 MHz, $CDCl_3$) δ 198.7, 149.2, 148.8, 124.3, 115.7, 115.1, 114.1, 56.3, 56.2, 50.3.

Data in agreement with the literature.[24]

1-(2-bromo-4,5-dimethoxyphenethyl)-1,2,3,4-Tetrahydroquinolin-6-ol (3f)

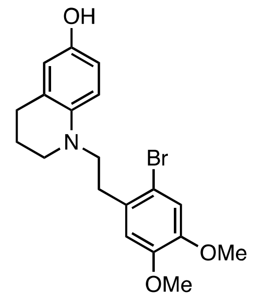

Prepared according to General Procedure F from 1,2,3,4-tetrahydroquinolin-6-ol (100 mg, 0.67 mmol) and 2-(2-bromo-4,5-dimethoxyphenyl)acetaldehyde (**S31**) (218 mg, 0.84 mmol). Purification by flash column chromatography (20-30% EtOAc in petroleum ether) afforded the title compound as an orange solid (137 mg, 0.35 mmol, 52% yield).

^{1}H NMR (400 MHz, Acetone-d_6) δ 7.09 (1H, s), 6.95 (1H, s), 6.65 (1H, d, *J*=8.7 Hz), 6.54 (1H, d, *J*=8.7 Hz), 6.45 (1H, s), 3.81 (3H, s), 3.78 (3H, s), 3.41 (2H, t, *J*=7.7 Hz), 3.24 – 3.17 (2H, m), 2.92 (2H, t, *J*=7.7 Hz), 2.65 (2H, t, *J*=6.4 Hz), 1.94 – 1.79 (2H, m).

^{13}C NMR (126 MHz, Acetone-d_6) δ 149.9, 149.6, 148.9, 139.3, 132.2, 124.5, 117.0, 116.7, 115.3, 114.5, 114.4, 113.3, 56.4, 56.2, 52.8, 50.1, 32.8, 28.9, 23.3.

Data in agreement with the literature.[17]

1-(2-bromo-4,5-dimethoxyphenethyl)Indolin-5-ol (3g)

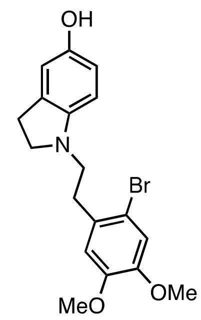

Prepared according to General Procedure F from indolin-5-ol (**S25**) (135 mg, 1.00 mmol) and 2-(2-bromo-4,5-dimethoxyphenyl)acetaldehyde (**S31**) (324 mg, 1.25 mmol). Purification by flash column chromatography (20-30% EtOAc in petroleum ether) afforded the title compound as an orange solid (253 mg, 0.67 mmol, 67% yield).

^{1}H NMR (500 MHz, Acetone-d_6) δ 7.43 (1H, s), 7.09 (1H, s), 7.04 (1H, s), 6.62 (1H, s), 6.52 (1H, d, *J*=8.3 Hz), 6.42 (1H, d, *J*=8.3 Hz), 3.81 (3H, s), 3.80 (3H, s), 3.34 (2H, t, *J*=7.7 Hz), 3.20 (2H, dd, *J*=9.0, 6.8 Hz), 2.94 (2H, dd, *J*=9.0, 6.8 Hz), 2.84 (2H, t, *J*=7.7 Hz).

^{13}C NMR (126 MHz, Acetone-d_6) δ 150.8, 150.0, 149.7, 146.5, 132.2, 132.1, 116.8, 115.3, 114.5, 113.8, 113.4, 108.6, 56.5, 56.3, 54.4, 51.3, 33.9, 29.5.

Data in agreement with the literature.[17]

Synthesis of Starting Materials in Scheme 3

General Route 4: Synthesis of *meta*-Tethered Substrates via Methoxy-protected Phenols

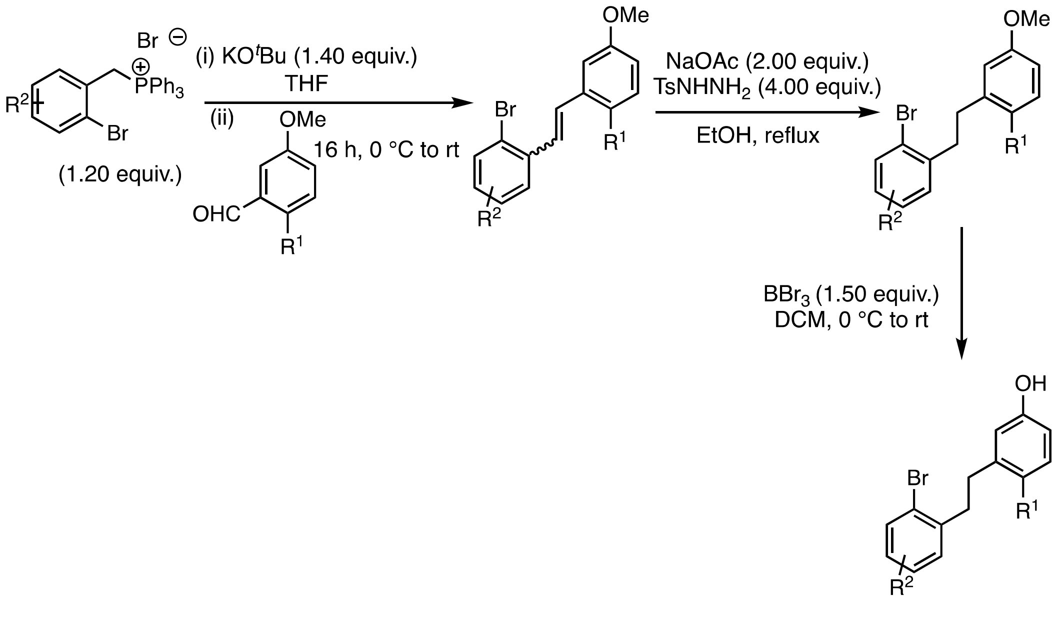

5-Methoxy-2-methylbenzaldehyde (S32)

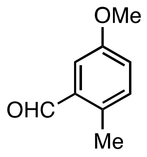

Prepared according to a modification of the procedure reported by Tang.[4] An oven-dried flask was charged with 2-bromo-5-methoxybenzaldehyde (5.74 g, 26.7 mmol, 1.00 equiv.), methylboronic acid (4.81 g, 80.1 mmol, 3.00 equiv.), K_3PO_4 (8.51 g, 40.1 mmol, 1.50 equiv.), SPhos (219 mg, 0.534 mmol, 2.00 mol%), and Pd_2dba_3 (245 mg, 0.267 mmol, 1.00 mol%). The flask was sealed, and PhMe (120 ml) was added via syringe. The flask was evacuated and backfilled with N_2 (3×). The reaction was heated to 110 °C under reflux for 16 h. The reaction was cooled to room temperature and filtered through celite, washing with EtOAc. The filtrate was concentrated under reduced pressure and purified by flash column chromatography (5-10% EtOAc in petroleum ether) to afford the title compound as a yellow oil (3.46 g, 23.1 mmol, 86% yield).

^{1}H NMR (400 MHz, $CDCl_3$) δ 10.27 (1H, d, J = 1.1 Hz), 7.31 (1H, t, J = 2.3 Hz), 7.15 (1H, dd, J = 8.3, 1.8 Hz), 7.03 (1H, dt, J = 8.3, 2.3 Hz), 3.88 – 3.76 (3H, m), 2.59 (3H, d, J = 1.1 Hz).

^{13}C NMR (101 MHz, $CDCl_3$) δ 192.2, 158.2, 134.8, 133.1, 132.9, 120.9, 114.2, 55.6, 18.2.

Data in agreement with the literature.[25]

3-(2-bromophenethyl)-4-Methylphenol (6a)

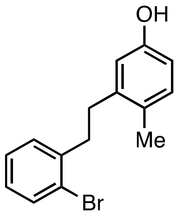

The title compound was prepared according to literature precedent from 5-methoxy-2-methylbenzaldehyde (**S32**) and (2-bromobenzyl)triphenylphosphonium bromide (**S5**) (General Route 4, General Procedures B, E, and D) as a colorless solid (182 mg, 0.63 mmol, 34% yield).[4]

^{1}H NMR (400 MHz, $CDCl_3$) δ 7.57 (dd, J = 7.9, 1.3 Hz, 1H), 7.24 (t, J = 7.4 Hz, 1H), 7.18 (dd, J = 7.4, 2.0 Hz, 1H), 7.09 (ddd, J = 9.1, 7.4, 1.9 Hz, 1H), 7.02 (d, J = 8.1 Hz, 1H), 6.70 (d, J = 2.7 Hz, 1H), 6.62 (dd, J = 8.1, 2.7 Hz, 1H), 4.70 (s, 1H), 3.01 – 2.95 (m, 2H), 2.88 – 2.81 (m, 2H), 2.26 (s, 3H).

^{13}C NMR (101 MHz, $CDCl_3$) δ 153.7, 141.2, 141.1, 133.0, 131.3, 130.6, 128.4, 127.9, 127.6, 124.5, 116.0, 113.1, 37.2, 33.8, 18.5.

Data in agreement with the literature.[4]

2-Ethyl-5-methoxybenzaldehyde (S33)

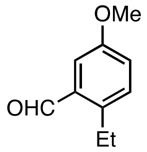

Prepared according to a modification of the procedure reported by Tang.[4] An oven-dried flask was charged with 2-bromo-5-methoxybenzaldehyde (2.00 g, 9.30 mmol, 1.00 equiv.), ethylboronic acid (2.04 g, 27.6 mmol, 3.00 equiv.), K_3PO_4 (6.86 g, 32.3 mmol, 3.47 equiv.), SPhos (76 mg, 0.19 mmol, 2.0 mol%), and Pd_2dba_3 (84 mg, 0.092 mmol, 1.0 mol%). The flask was sealed and evacuated and backfilled with N_2 (3×). PhMe (40 ml) was added via syringe. The reaction was heated to 110 °C under reflux for 16 h. The reaction was cooled to room temperature and filtered through celite, washing with EtOAc. The filtrate was concentrated under reduced pressure and purified by flash column chromatography (5-10% EtOAc in petroleum ether) to afford the title compound as a yellow oil (1.28 g, 7.80 mmol, 84% yield).

^{1}H NMR (500 MHz, $CDCl_3$) δ 10.29 (1H, s), 7.35 (1H, d, J=2.9 Hz), 7.20 (1H, d, J=8.4 Hz), 7.08 (1H, dd, J=8.4, 2.9 Hz), 3.84 (3H, d, J=0.7 Hz), 2.99 (2H, q, J=7.6 Hz), 1.25 (3H, t, J=7.6 Hz).

^{13}C NMR (126 MHz, $CDCl_3$) δ 191.8, 158.2, 139.9, 134.2, 131.6, 121.4, 113.5, 55.6, 24.7, 17.1.

Data in agreement with the literature.[25]

3-(2-bromophenethyl)-4-Ethylphenol (6b)

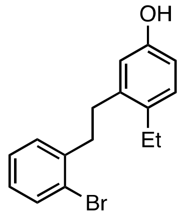

The title compound was prepared according to General Route 4. General Procedure B was performed with 2-ethyl-5-methoxybenzaldehyde (**S33**) (1.09 g, 6.64 mmol) and (2-bromobenzyl)triphenylphosphonium bromide (**S5**). The crude product was purified by flash column chromatography (0-2% Et_2O in petroleum ether) to afford the alkene as a yellow oil (1.53 g). General procedure E was performed with the alkene (793 mg, 2.50 mmol) (reaction time: 12 h). The crude product was purified by flash column chromatography (0-5% Et_2O in petroleum ether) to afford the methoxy-protected phenol as a yellow oil (760 mg). General Procedure D was

performed with the methoxy-protected phenol (415 mg, assuming 1.30 mmol). The crude product was purified by flash column chromatography (10% EtOAc in petroleum ether) to afford the title compound as a colorless solid (98.5 mg, 0.323 mmol, 5% yield over 3 steps).

^{1}H NMR (400 MHz, $CDCl_3$) δ 7.56 (1H, dd, *J*=7.9, 1.2 Hz), 7.25 – 7.17 (2H, m), 7.11 – 7.07 (1H, m), 7.05 (1H, d, *J*=8.1 Hz), 6.70 (1H, d, *J*=2.8 Hz), 6.65 (1H, dd, *J*=8.1, 2.8 Hz), 4.52 (1H, s), 3.03 – 2.93 (2H, m), 2.92 – 2.77 (2H, m), 2.62 (2H, q, *J*=7.5 Hz), 1.19 (3H, t, *J*=7.5 Hz).

^{13}C NMR (101 MHz, $CDCl_3$) δ 153.6, 141.2, 140.6, 134.6, 133.0, 130.6, 129.7, 127.9, 127.7, 124.5, 116.1, 113.3, 38.0, 33.2, 24.9, 15.8.

Data in agreement with the literature.[4]

3-(2-bromo-6-chlorophenethyl)-4-Methylphenol (6c)

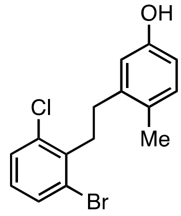

The title compound was prepared according to General Route 4. General Procedure B was performed with (2-bromo-6-chlorobenzyl)triphenylphosphonium bromide (**S17**) (3.28 g, 6.00 mmol) and 5-methoxy-2-methylbenzaldehyde (**S32**) (751 mg, 5.00 mmol). The crude alkene product was subjected to General Procedures E and D without further purification. The final crude product was purified by flash column chromatography (10-20% EtOAc in petroleum ether) to afford the title compound as a colorless solid (65 mg, 0.20 mmol, 4% yield over three steps).

^{1}H NMR (400 MHz, MeCN-*d*$_3$) δ 7.55 (dd, *J* = 8.1, 1.2 Hz, 1H), 7.41 (dd, *J* = 8.1, 1.2 Hz, 1H), 7.10 (t, *J* = 8.1 Hz, 1H), 6.98 (d, *J* = 8.1 Hz, 1H), 6.68 (d, *J* = 2.7 Hz, 1H), 6.62 – 6.54 (m, 2H), 3.17 – 3.08 (m, 2H), 2.80 – 2.71 (m, 2H), 2.29 (s, 3H).

^{13}C NMR (101 MHz, MeCN-*d*$_3$) δ 156.1, 141.5, 139.7, 135.8, 132.8, 132.1, 130.1 129.8, 128.1, 126.2, 116.8, 114.1, 36.0, 32.5, 18.5.

HRMS m/z: $[M+H]^+$ calculated for $[C_{15}H_{15}BrClO]^+$ 324.9989, found 324.9993. Δ = +1.2 ppm.

3-(2-bromo-5-fluorophenethyl)-4-Methylphenol (6d)

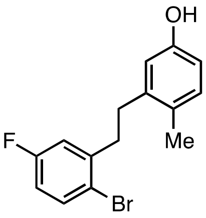

The title compound was prepared according to General Route 4. General Procedure B was performed with (2-bromo-5-fluorobenzyl)triphenylphosphonium bromide (**S23**) (3.18 g, 6.00 mmol) and 5-methoxy-2-methylbenzaldehyde (**S32**) (751 mg, 5.00 mmol). The crude alkene product (964 mg, assuming 3.00 mmol) was subjected to General Procedures E and D without further purification. The final crude product was purified by flash column chromatography (100% $CHCl_3$) to afford the title compound as a colorless solid (85 mg, 0.27 mmol, 5% yield over three steps).

^{1}H NMR (400 MHz, $CDCl_3$) δ 7.53 (dd, *J* = 8.8, 5.4 Hz, 1H), 7.04 (d, *J* = 8.2 Hz, 1H), 6.92 (dd, *J* = 9.3, 3.1 Hz, 1H), 6.84 (ddd, *J* = 8.7, 7.9, 3.1 Hz, 1H), 6.70 (d, *J* = 2.7 Hz, 1H), 6.64 (dd, *J* = 8.2, 2.7 Hz, 1H), 4.64 (s, 1H), 2.99 – 2.94 (m, 2H), 2.88 – 2.82 (m, 2H), 2.27 (s, 3H).

^{13}C NMR (101 MHz, $CDCl_3$) δ 162.1 (d, *J* = 246.6 Hz), 153.8, 143.2 (d, *J* = 7.4 Hz), 140.7, 134.0 (d, *J* = 8.2 Hz), 131.4, 128.3, 118.5 (d, *J* = 3.1 Hz), 117.4 (d, *J* = 22.5 Hz), 115.9, 115.1 (d, *J* = 22.3 Hz), 113.2, 37.2, 33.5, 18.5.

^{19}F NMR (376 MHz, $CDCl_3$) δ -116.1.

Data in agreement with the literature.[4]

1-Bromo-4,5-dimethoxy-2-(5-methoxy-2-methylphenethyl)benzene (S34)

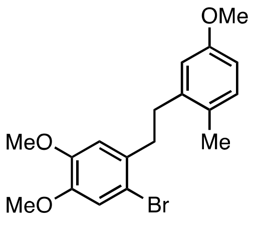

The title compound was prepared according to General Route 4. General Procedure B was performed with (2-bromo-4,5-dimethoxybenzyl)triphenylphosphonium bromide (**S16**) (3.43 g, 6.00 mmol) and 5-methoxy-2-methylbenzaldehyde (**S32**) (751 mg, 5.00 mmol). The crude alkene product (964 mg, assuming 2.65 mmol) was subjected to General Procedure E without further purification. The crude product was purified by flash column chromatography (10-20% EtOAc

in petroleum ether) to afford the title compound as a colorless solid (442 mg, 1.21 mmol, 24% over two steps).

^{1}H NMR (400 MHz, $CDCl_3$) δ 7.07 – 7.01 (m, 2H), 6.72 (d, *J* = 2.8 Hz, 1H), 6.68 (dd, *J* = 8.2, 2.8 Hz, 1H), 6.56 (s, 1H), 3.86 (s, 3H), 3.77 (s, 6H), 2.96 – 2.87 (m, 2H), 2.87 – 2.79 (m, 2H), 2.23 (s, 3H).

^{13}C NMR (101 MHz, $CDCl_3$) δ 158.0, 148.3, 148.1, 140.9, 133.0, 131.0, 128.3, 115.6, 115.1, 114.1, 113.4, 111.3, 56.3, 56.1, 55.4, 36.9, 34.1, 18.5.

HRMS m/z: $[M+Na]^+$ calculated for $[C_{18}H_{21}BrNaO_3]^+$ 387.0566, found 387.0555. Δ = –3.0 ppm.

4-Bromo-5-(5-hydroxy-2-methylphenethyl)benzene-1,2-diol (S35)

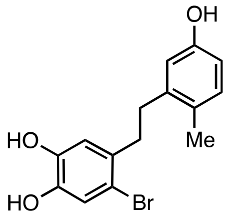

1-Bromo-4,5-dimethoxy-2-(5-methoxy-2-methylphenethyl)benzene (**S34**) (400 mg, 1.10 mmol, 1.00 equiv.) was dissolved in anhydrous CH_2Cl_2 (18 mL) and cooled to 0 °C under nitrogen. BBr_3 (1.00 M in CH_2Cl_2, 4.40 mL, 4.40 mmol) was added dropwise. The reaction was slowly warmed to room temperature and stirred for 5 hours. Upon completion, the reaction was cooled to 0 °C and slowly quenched with H_2O, and extracted with CH_2Cl_2 (3×). The combined organic extracts were dried over $MgSO_4$, filtered, and the solvent removed under reduced pressure to afford the title compound as a colorless solid (206 mg, 0.64 mmol, 58% yield), without further purification.

^{1}H NMR (400 MHz, MeCN-d_3) δ 7.00 (s, 1H), 6.95 (d, *J* = 8.2 Hz, 1H), 6.80 – 6.65 (m, 4H), 6.62 (d, *J* = 2.7 Hz, 1H), 6.55 (dd, *J* = 8.2, 2.7 Hz, 1H), 2.84 – 2.69 (m, 4H), 2.20 (s, 3H).

^{13}C NMR (101 MHz, MeCN-d_3) δ 155.9, 145.2, 144.7, 141.9, 133.6, 131.9, 128.0, 119.8, 118.0, 116.8, 113.8, 113.5, 37.0, 34.6, 18.5.

HRMS m/z: $[M–H]^–$ calculated for $[C_{15}H_{14}BrO_3]^–$ 321.0132, found 321.0130. Δ = –0.6 ppm.

3-(2-(6-bromo-2,2-dimethylbenzo[*d*][1,3]dioxol-5-yl)ethyl)-4-Methylphenol (6e)

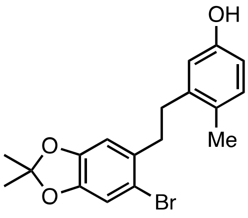

A 4 mL crimped top vial was charged with a stirrer bar and 4-bromo-5-(5-hydroxy-2-methylphenethyl)benzene-1,2-diol (**S35**) (64 mg, 0.20 mmol, 1.00 equiv.). The vial was sealed and evacuated and backfilled with N_2 (3×). Anhydrous benzene (0.5 mL) and anhydrous acetone (0.1 mL) were added, followed by the dropwise addition of neat PCl_3 (c.a. 6 drops) under nitrogen. The reaction was stirred at room temperature overnight. Upon completion, the reaction mixture was purified directly by flash column chromatography (20% EtOAc in petroleum ether) to afford the title compound as a colorless, viscous oil (22 mg, 0.061 mmol, 30%). The procedure was repeated on the same scale to obtain enough material for the palladium-catalyzed dearomatization.

^{1}H NMR (400 MHz, $CDCl_3$) δ 7.01 (d, *J* = 8.1 Hz, 1H), 6.92 (s, 1H), 6.70 (d, *J* = 2.7 Hz, 1H), 6.63 – 6.58 (m, 2H), 4.67 (s, 1H), 2.89 – 2.80 (m, 2H), 2.80 – 2.72 (m, 2H), 2.26 (s, 3H), 1.67 (s, 6H).

^{13}C NMR (126 MHz, $CDCl_3$) δ 153.8, 147.3, 146.8, 141.3, 133.5, 131.3, 128.3, 119.1, 115.9, 113.7, 113.0, 112.6, 109.9, 37.1, 34.2, 25.9, 18.6.

HRMS m/z: $[M–H]^-$ calculated for $[C_{18}H_{18}BrO_3]^-$ 361.0445, found 361.0428. Δ = –4.8 ppm.

Synthesis of Starting Materials in Scheme 4

General Procedure H: Synthesis of *O*–Linked Phenols *via* Alkylation with 2-Bromobenzyl Bromide

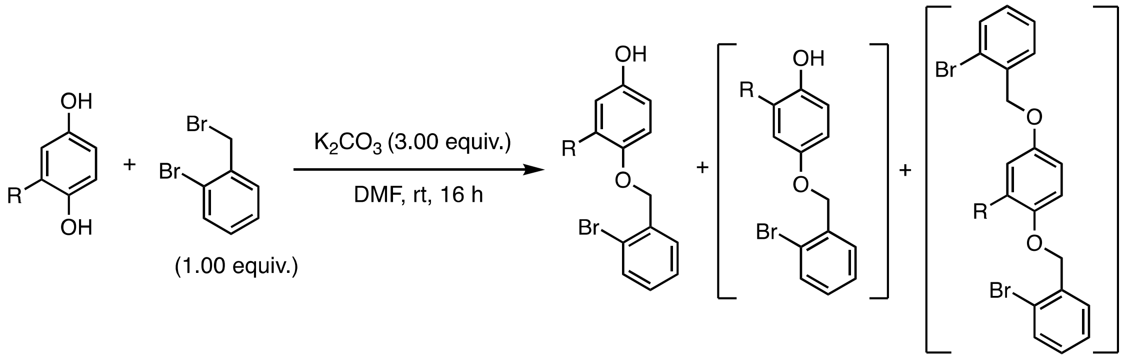

To a 0.7 M solution of substituted hydroquinone in DMF was added K_2CO_3 (3.00 equiv.). The reaction was stirred at room temperature for 30 minutes. 2-Bromobenzyl bromide (1.00 equiv.) was added, and the reaction was stirred for 16 h at room temperature. Upon completion, the DMF was removed under a stream of air. EtOAc and sat. NH_4Cl (aq.) were added, and the layers were separated. The aqueous phase was further extracted with EtOAc (2×). The combined organic extracts were dried over Na_2SO_4, and concentrated under reduced pressure. The desired product was isolated by flash column chromatography.

4-((2-bromobenzyl)oxy)-3-Methylphenol (8a)

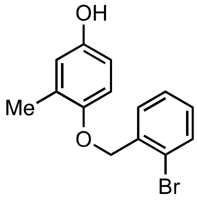

Prepared according to General Procedure H from 2-methylbenzene-1,4-diol (1.74 g, 14.0 mmol). Purification by flash column chromatography (10-15% EtOAc in petroleum ether) afforded the title compound as a colorless solid (159 mg, 0.54 mmol, 4% yield).

^{1}H NMR (500 MHz, $CDCl_3$) δ 7.62 – 7.54 (2H, m), 7.34 (1H, td, *J*=7.5, 1.3 Hz), 7.21 – 7.16 (1H, m), 6.76 (1H, d, *J*=8.7 Hz), 6.69 (1H, dd, *J*=3.1, 0.7 Hz), 6.61 (1H, ddd, *J*=8.7, 3.1, 0.7 Hz), 5.06 (2H, s), 4.36 (1H, s), 2.28 (3H, s).

^{13}C NMR (126 MHz, $CDCl_3$) δ 151.0, 149.6, 137.1, 132.6, 129.1, 128.8, 128.7, 127.7, 122.2, 118.1, 113.2, 112.7, 70.3, 16.6.

[N.B. The structure of this isomer was assigned based on an HMBC interaction between O-**H** (δ = 4.36) and 2 × C_{Ar} (C–H) signals (δ = 118.1, 112.7)].

HRMS m/z: $[M+H]^+$ calculated for $[C_{14}H_{14}BrO_2]^+$ 293.0172, found 293.0182. Δ = +3.4 ppm.

4-((2-bromobenzyl)oxy)-3-Methoxyphenol (8b)

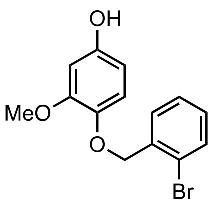

Prepared according to General Procedure H from 2-methoxybenzene-1,4-diol (980 mg, 7.00 mmol). Purification by flash column chromatography (10-20% EtOAc in petroleum ether) afforded the title compound as a yellow solid (176 mg, 0.57 mmol, 8% yield).

^{1}H NMR (400 MHz, $CDCl_3$) δ 7.60 – 7.52 (2H, m), 7.31 (1H, td, *J*=7.6, 1.3 Hz), 7.15 (1H, td, *J*=7.6, 1.8 Hz), 6.73 (1H, d, *J*=8.6 Hz), 6.49 (1H, d, *J*=2.9 Hz), 6.28 (1H, dd, *J*=8.6, 2.9 Hz), 5.13 (2H, s), 4.69 (1H, s), 3.86 (3H, s).

^{13}C NMR (101 MHz, $CDCl_3$) δ 151.1, 151.0, 142.1, 136.9, 132.6, 129.2, 129.0, 127.6, 122.2, 116.2, 106.2, 101.0, 71.6, 56.1.

[N.B. The structure of this isomer was assigned based on an HMBC interaction between O-**H** (δ = 4.69) and 2 × C_{Ar} (C–H) signals (δ = 106.2, 101.0)].

HRMS m/z: $[M+H]^+$ calculated for $[C_{14}H_{14}BrO_3]^+$ 309.0121, found 309.0112. Δ = –2.9 ppm.

4-((2-bromo-6-chlorobenzyl)oxy)-2,3-Dimethylphenol (8c)

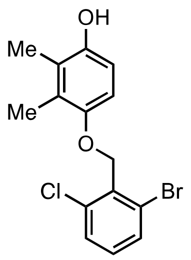

To a solution of 2,3-dimethylbenzene-1,4-diol (931 mg, 6.75 mmol, 3.00 equiv.) in DMF (5 ml) was added K_2CO_3 (1.24 g, 9.00 mmol, 4.00 equiv.). The reaction was stirred at room temperature for 30 minutes. 1-Bromo-2-(bromomethyl)-3-chlorobenzene (639 mg, 2.25 mmol, 1.00 equiv.) was added, and the reaction was stirred for 16 h at room temperature. Upon completion, the DMF was removed under a stream of air. EtOAc and sat. NH_4Cl (aq.) were added, and the layers were separated. The aqueous phase was further extracted with EtOAc (2×). The combined organic extracts were dried over Na_2SO_4, and concentrated under reduced pressure. The desired product was purified by flash column chromatography (5-7.5% EtOAc in petroleum ether) to afford the title compound as a colorless solid (432 mg, 1.26 mmol, 56% yield).

^{1}H NMR (500 MHz, $CDCl_3$) δ 7.55 (1H, dd, *J*=8.1, 1.2 Hz), 7.40 (1H, dd, *J*=8.1, 1.2 Hz), 7.16 (1H, td, *J*=8.1, 0.8 Hz), 6.85 (1H, d, *J*=8.6 Hz), 6.63 (1H, d, *J*=8.6 Hz), 5.20 (2H, s), 4.47 (1H, s), 2.18 (6H, app d, *J*=2.2 Hz).

^{13}C NMR (126 MHz, $CDCl_3$) δ 151.2, 148.5, 137.0, 134.4, 131.9, 130.7, 129.2, 128.5, 127.2, 124.2, 112.4, 112.2, 69.8, 12.5, 12.3.

HRMS m/z: $[M]^+$ calculated for $[C_{15}H_{14}BrClO_2]^+$ 339.9860, found 339.9863. Δ = +0.9 ppm.

Synthesis of Starting Materials in Scheme 5

(4-(2-bromophenethyl)-3-methoxyphenoxy)Trimethylsilane (S37)

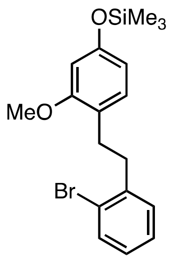

To a solution 4-(2-bromophenethyl)-3-methoxyphenol (**1a**) (307 mg, 1.00 mmol, 1.00 equiv.) in CH_2Cl_2 (6 ml) was added NEt_3 (303 mg, 417 µl, 3.00 mmol, 3.00 equiv.) under nitrogen. The mixture was stirred for 10 minutes, after which trimethylsilyl chloride (262 mg, 306 µL, 2.40 mmol, 2.40 equiv.) was added, followed by an additional portion of CH_2Cl_2 (6 ml). The reaction was stirred at room temperature for 16 h. Upon completion, the solvent was evaporated under a stream of air. The residue was dissolved in Et_2O and filtered to remove solids. The filtrate was concentrated under reduced pressure to afford the title compound as a colorless oil (379 mg, 1.00 mmol, quant.) without further purification.

^{1}H NMR (500 MHz, $CDCl_3$) δ 7.54 (1H, d, *J*=7.6 Hz), 7.19 (1H, t, *J*=7.6 Hz), 7.14 (1H, dd, *J*=7.6, 2.0 Hz), 7.04 (1H, td, *J*=7.6, 2.0 Hz), 6.93 (1H, d, *J*=8.0 Hz), 6.39 (1H, d, *J*=2.3 Hz), 6.36 (1H, dd, *J*=8.0, 2.3 Hz), 3.77 (3H, s), 2.98 (2H, dd, *J*=10.1, 6.0 Hz), 2.89 – 2.80 (2H, m), 0.28 (9H, s).

^{13}C NMR (101 MHz, $CDCl_3$) δ 158.4, 154.8, 141.8, 132.8, 130.7, 130.2, 127.5, 127.3, 124.7, 122.9, 111.3, 103.6, 55.4, 36.7, 30.2, 0.4.

HRMS m/z: $[M+H]^+$ calculated for $[C_{18}H_{23}BrO_2Si]^+$ 379.0723, found 379.0716. Δ = –1.8 ppm.

Potassium 4-(2-bromophenethyl)-3-methoxyphenolate (S38)

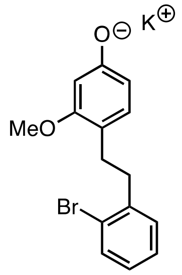

4-(2-bromophenethyl)-3-Methoxyphenol (**1a**) (307 mg, 1.00 mmol, 1.00 equiv.) and KOH (57.2 mg, 1.02 mmol, 1.02 equiv.) were dissolved in methanol. The solvent was removed under a stream of nitrogen, and the residue was concentrated under reduced pressure, to afford the title compound as an orange solid.

^{1}H NMR (400 MHz, MeOD) δ 7.49 (1H, d, *J*=7.7 Hz), 7.21 – 7.14 (1H, m), 7.11 (1H, dd, *J*=7.7, 1.9 Hz), 7.02 (1H, td, *J*=7.7, 1.9 Hz), 6.71 (1H, d, *J*=8.2 Hz), 6.32 (1H, d, *J*=2.2 Hz), 6.19 (1H, dd, *J*=8.2, 2.2 Hz), 3.72 (3H, s), 2.89 (2H, dd, *J*=9.4, 6.2 Hz), 2.72 (2H, dd, *J*=9.4, 6.2 Hz).

^{13}C NMR (101 MHz, MeOD) δ 163.6, 159.7, 143.2, 133.6, 131.9, 131.0, 128.5, 128.4, 125.2, 118.4, 109.9, 101.7, 55.5, 38.3, 31.4.

HRMS m/z: $[M+H]^+$ calculated for $[C_{15}H_{15}BrKO_2]^+$ 344.9887, found 344.9895. Δ = +2.3.

To verify that complete deprotonation was taking place under these conditions, the following recrystallization procedure was also performed:

The orange solid (**S38**) was dissolved in THF. Hexane was added slowly until some precipitation was observed, and the mixture was recrystallized in a freezer overnight. The mother liqueur was removed, and the crystals were concentrated under reduced pressure. To account for the possibility that the crystals contained water or solvent molecules, the molecular weight of the sample was determined by ^{1}H NMR with the addition of a dibromomethane internal standard, and found to be 455 gmol^{-1}. The yield of the recrystallization was calculated (181 mg, 0.40 mmol, 40% yield).

An upfield shift of signals corresponding to phenol ring substituents was observed compared to **1a** (see below). A comparison of the spectra before and after recrystallization suggests that the addition of 1.02 equiv. KOH in MeOH is sufficient for complete deprotonation to occur.

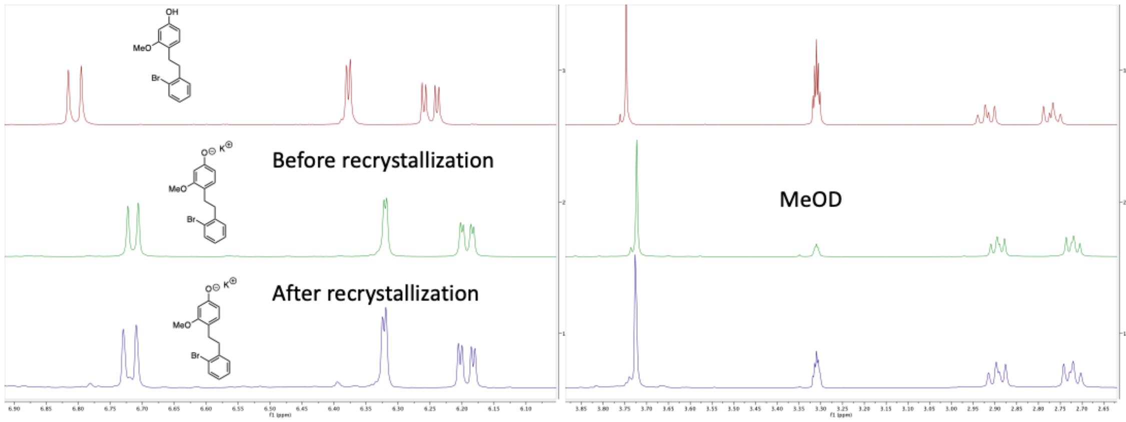

A 0.1 mmol variant of this procedure, omitting the recrystallization, was therefore performed for the control experiment in scheme 5A.

Synthesis of Alkylated Ligand

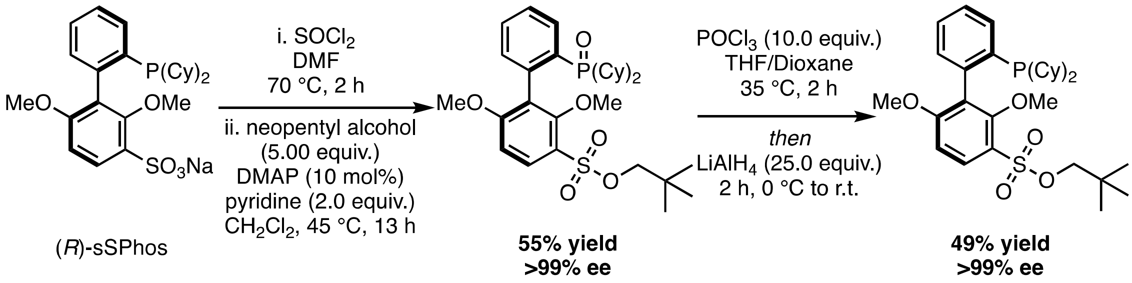

(*R*)-Neopentyl 2'-(dicyclohexylphosphoryl)-2,6-dimethoxy-[1,1'-biphenyl]-3-sulfonate (S39)

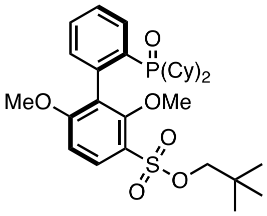

Step 1: A 20 mL crimped top microwave vial was charged with (*R*)-Na-sSPhos (257 mg, 0.500 mmol, 1.00 equiv.) and subjected to three nitrogen/vacuum cycles. Thionyl chloride (5 mL) was added at room temperature under a nitrogen atmosphere, followed by anhydrous DMF (0.5 mL). The solution was stirred at 70 °C for 2 h. Upon completion, the excess thionyl chloride was removed under a stream of air. The crude reaction mixture was cooled to 0 °C and diluted with EtOAc (5 mL), and excess thionyl chloride was quenched by the dropwise addition of water (5 mL). The mixture was extracted with EtOAc (3×). The combined organic extracts were dried over $MgSO_4$, filtered, and the solvent removed under reduced pressure. The crude sulfonyl

chloride product was dried under high vacuum for 1 hour and subjected to the next step without further purification.

Step 2: A 5 mL crimped top microwave vial was charged with neopentyl alcohol (220 mg, 2.50 mmol, 5.00 equiv.) and DMAP (6 mg, 0.05 mmol, 10 mol%), and was subjected to three nitrogen/vacuum cycles. Crude sulfonyl chloride was dissolved in CH_2Cl_2 (2.5 mL) and added under a nitrogen atmosphere, followed by anhydrous pyridine (AcroSeal Extra Dry) (79 mg, 81 μL, 1.0 mmol, 2.0 equiv.). The reaction was heated to 45 °C and stirred for 13 hours. The solvent was removed under a stream of air and the crude reaction mixture purified by flash column chromatography, to afford the title compound as a colorless solid (159 mg, 0.276 mmol, 55%, > 99% ee).

^{1}H NMR (400 MHz, $CDCl_3$) δ 7.91 (d, *J* = 8.9 Hz, 1H), 7.57 – 7.42 (m, 3H), 7.29 (dd, *J* = 7.2, 2.4 Hz, 1H), 6.75 (d, *J* = 8.9 Hz, 1H), 3.85 (s, 2H), 3.73 (s, 3H), 3.43 (s, 3H), 1.93 – 1.68 (m, 9H), 1.61 (dd, *J* = 15.1, 7.2 Hz, 4H), 1.45 (ddt, *J* = 17.5, 12.1, 4.9 Hz, 2H), 1.31 – 1.07 (m, 7H), 0.98 (s, 9H).

^{13}C NMR (101 MHz, $CDCl_3$) δ 162.2, 157.5, 138.7 (d, *J* = 4.0 Hz), 133.6 (d, *J* = 8.9 Hz), 132.0, 131.5, 130.9 – 130.7 (m), 130.3 (d, *J* = 2.7 Hz), 127.3 (d, *J* = 11.1 Hz), 126.6 (d, *J* = 2.6 Hz), 121.9, 105.4, 80.1, 61.8, 55.8, 38.6 (d, *J* = 66.3 Hz), 37.0 (d, *J* = 67.0 Hz), 31.9, 27.0 (d, *J* = 3.3 Hz), 26.8 (d, *J* = 3.5 Hz), 26.7 (d, *J* = 12.5 Hz), 26.5, 26.4 (d, *J* = 2.9 Hz), 26.0 – 25.8 (m), 25.6 – 25.5 (m), 25.3 (d, *J* = 2.9 Hz).

^{31}P NMR (162 MHz, $CDCl_3$) δ 45.7.

HRMS m/z: $[M+H]^+$ calculated for $[C_{31}H_{46}O_6PS]^+$ 577.2747, found 577.2764. Δ = +2.9 ppm.

Chiral HPLC Analysis: CHIRALPAK IG (Hexane: ^{i}PrOH, 85:15, 1.25 mL min^{-1}, 40 °C, 210 nm) indicated > 99% ee, t_R= 18.15 (minor), 20.24 (major) minutes.

$[\alpha]_D^{25}$ = + 25.2° (c. 0.79, $CHCl_3$).

(*R*)-Neopentyl 2'-(dicyclohexylphosphaneyl)-2,6-dimethoxy-[1,1'-biphenyl]-3-sulfonate [(*R*)-sSPhos-Np]

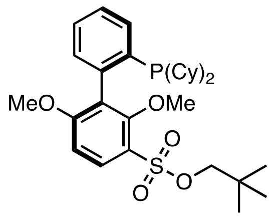

A 20 mL crimped top microwave vial was charged with (*R*)-neopentyl 2'-(dicyclohexylphosphoryl)-2,6-dimethoxy-[1,1'-biphenyl]-3-sulfonate (**S39**) (144 mg, 0.250 mmol) and subjected to three nitrogen/vacuum cycles. THF (3 mL) and 1,4-dioxane (0.70 mL) were added under nitrogen, followed the dropwise addition of phosphoryl chloride (383 mg, 234 µL, 2.50 mmol, 10.0 equiv.). The reaction was stirred at 35 °C for 2 hours. The reaction mixture was then cooled to 0 °C and $LiAlH_4$ (2.00 M in THF, 3.12 mL, 6.25 mmol, 25.0 equiv.) was added dropwise (*caution: exotherm*). The reaction was stirred at room temperature for 2 hours. Upon completion, the reaction was quenched with the slow addition of EtOAc (2 mL), methanol (2 mL) and water (2 mL). Additional water (20 mL) was added to the quenched reaction mixture. The aqueous phase was extracted with chloroform (3×). The combined organic extracts were dried over $MgSO_4$, filtered, and the solvent removed under reduced pressure on a rotary evaporator (keeping the water bath at room temperature). The crude product was purified by flash column chromatography (50% EtOAc in petroleum ether) to afford the title compound as a colorless solid (68.7 mg, 0.123 mmol, 49%, > 99% ee).

^{1}H NMR (400 MHz, $CDCl_3$) δ 7.94 (d, *J* = 8.9 Hz, 1H), 7.60 (d, *J* = 6.3 Hz, 1H), 7.44 – 7.34 (m, 2H), 7.25 – 7.20 (m, 1H), 6.76 (d, *J* = 8.9 Hz, 1H), 3.82 (s, 2H), 3.74 (s, 3H), 3.34 (s, 3H), 2.06 – 1.95 (m, 1H), 1.78 – 1.48 (m, 11H), 1.36 – 1.03 (m, 10H), 0.99 (s, 9H).

^{13}C NMR (101 MHz, $CDCl_3$) δ (101 MHz, $CDCl_3$) δ 162.6, 157.1 (d, *J* = 1.3 Hz), 140.7 (d, *J* = 32.3 Hz), 136.7 (d, *J* = 20.7 Hz), 132.9 (d, *J* = 3.5 Hz), 131.8 – 131.6 (m), 128.2, 127.3, 127.1 (d, *J* = 7.0 Hz), 122.1, 105.5, 80.2 (d, *J* = 2.7 Hz), 61.3, 55.8, 35.5 (d, *J* = 15.5 Hz), 33.1 (d, *J* = 13.6 Hz), 32.0, 30.4 – 29.9 (m), 29.4 (d, *J* = 12.6 Hz), 28.9 (d, *J* = 6.6 Hz), 27.9 – 27.5 (m), 27.5 – 27.1 (m), 26.5.

^{31}P NMR (162 MHz, $CDCl_3$) δ -8.9.

HRMS m/z: $[M+H]^+$ calculated for $[C_{31}H_{46}O_5PS]^+$ 561.2798, found 561.2812. Δ = + 2.5 ppm.

Chiral HPLC Analysis: CHIRALPAK IG (Hexane: ^{i}PrOH, 95:05, 1.25 mL min^{-1}, 40 °C, 210 nm) indicated > 99% ee, t_R= 5.29 (minor), 5.84 (major) minutes.

$[\alpha]_D^{25}$ = + 2.12° (c. 0.79, $CHCl_3$).

Characterization of Products in Scheme 1

General Procedure J: Palladium/(*R*)–sSPhos-Catalyzed Dearomatization of Phenols

A vial containing a magnetic stirrer bar was charged with phenol starting material (0.100 mmol), crushed KOH (8.4 mg, 0.15 mmol, 1.5 equiv.), (*R*)–sSPhos (3.8 mg, 0.0075 mmol, 7.5 mol%), and Pd_2dba_3 (2.3 mg, 0.0025 mmol, 2.5 mol%). The vial was sealed, and evacuated and backfilled with N_2 (3×). PhMe (0.50 mL) and H_2O (50 µL) were added under nitrogen. The reaction was stirred at 110 °C and 900 rpm for 20 h. Upon completion, the vial was opened, and the solvent was removed under a stream of air. Sat. NH_4Cl (aq.) (0.5 ml) was added, and the aqueous layer was extracted with chloroform (3 × 0.5 ml). The combined organic extracts were dried over $MgSO_4$ and purified by flash column chromatography or preparatory TLC.

(*R*)-2-Methoxy-2',3'-dihydrospiro[cyclohexane-1,1'-indene]-2,5-dien-4-one (2a)

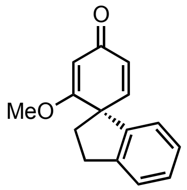

Prepared according to General Procedure J with 4-(2-bromophenethyl)-3-methoxyphenol **(1a)** (30.7 mg, 0.100 mmol) as the starting material. Purification by flash column chromatography (20% EtOAc in petroleum ether) afforded the title compound as a colorless solid (22.2 mg, 0.098 mmol, 98% yield, 92% ee).

^{1}H NMR (400 MHz, $CDCl_3$) δ 7.30 (1H, d, *J*=7.5 Hz), 7.24 (1H, t, *J*=7.5 Hz), 7.15 (1H, t, *J*=7.5 Hz), 6.88 (1H, d, *J*=7.5 Hz), 6.61 (1H, d, *J*=9.7 Hz), 6.16 (1H, dd, *J*=9.7, 1.6 Hz), 5.64 (1H, d, *J*=1.6 Hz), 3.63 (3H, s), 3.25 (1H, ddd, *J*=15.4, 8.9, 6.0 Hz), 3.12 (1H, ddd, *J*=15.4, 8.7, 6.0 Hz), 2.61 (1H, ddd, *J*=13.1, 8.9, 6.0 Hz), 2.25 (1H, ddd, *J*=13.1, 8.7, 6.0 Hz).

^{13}C NMR (101 MHz, $CDCl_3$) δ 188.7, 178.3, 149.0, 144.7, 142.8, 128.3, 127.1, 125.5, 125.2, 123.7, 102.0, 55.9, 55.9, 36.5, 31.9.

Chiral SFC Analysis: CHIRALPAK IG (CO_2:MeOH, 90:10, 2.5 mL min^{-1}, 40 °C, 220 nm) indicated 92% ee, t_R = 6.41 (minor), 7.74 (major) minutes. The absolute configuration was determined by X-ray diffraction (see **Determination of Product Absolute Stereochemistry**).

[α]$_D^{25}$ = +41.8° (c 1.09, $CHCl_3$).

Data in agreement with the literature.[5]

Control Experiment 5D:

Prepared according to General Procedure J with 4-(2-bromophenethyl)-3-methoxyphenol **(1a)** (30.7 mg, 0.100 mmol) as the starting material, and (*R*)-sSPhos-Np (4.2 mg, 0.0075 mmol, 7.5 mol%) in place of (*R*)-sSPhos as the ligand. Purification by flash column chromatography (20% EtOAc in petroleum ether) afforded the title compound as a colorless solid (20.2 mg, 0.089 mmol, 89% yield, -6% ee).

Chiral SFC Analysis: CHIRALPAK IG (CO_2:MeOH, 90:10, 2.5 mL min^{-1}, 40 °C, 220 nm) indicated -6% ee, t_R = 6.32 (major), 7.65 (minor) minutes.

(*S*)-2-Methyl-2',3'-dihydrospiro[cyclohexane-1,1'-indene]-2,5-dien-4-one (2b)

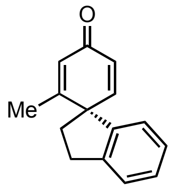

Prepared according to General Procedure J with 4-(2-bromophenethyl)-3-methylphenol (**1b**) (29.1 mg, 0.100 mmol) as the starting material. Purification by flash column chromatography (10-20% EtOAc in petroleum ether) afforded the title compound as a yellow solid (17.0 mg, 0.081 mmol, 81% yield, 86% ee).

^{1}H NMR (400 MHz, $CDCl_3$) δ 7.32 (1H, dt, *J*=7.4, 1.1 Hz), 7.25 (1H, td, *J*=7.4, 1.1 Hz), 7.16 (1H, td, *J*=7.4, 1.1 Hz), 6.86 (1H, d, *J*=10.1 Hz), 6.82 (1H, d, *J*=7.4 Hz), 6.22 – 6.18 (2H, m), 3.31 – 3.10 (2H, m), 2.43 (1H, ddd, *J*=13.6, 8.5, 7.2 Hz), 2.27 (1H, ddd, *J*=13.6, 8.5, 6.1 Hz), 1.77 (3H, d, *J*=1.2 Hz).

^{13}C NMR (101 MHz, $CDCl_3$) δ 187.0, 162.5, 153.8, 144.2, 143.1, 128.3, 127.6, 127.4, 125.6, 125.4, 124.1, 57.0, 35.9, 31.9, 20.1.

Chiral SFC Analysis: CHIRALPAK IG (CO_2:MeOH, 90:10, 2.5 mL min^{-1}, 40 °C, 220 nm) indicated 86% ee, t_R = 7.49 (minor), 7.97 (major) minutes.

[α]$_D^{25}$ = +58.5° (c 0.79, $CHCl_3$).

Data in agreement with the literature.[5]

(*S*)-2-Phenyl-2',3'-dihydrospiro[cyclohexane-1,1'-indene]-2,5-dien-4-one (2c)

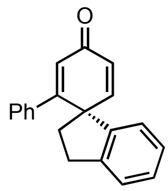

Prepared according to General Procedure J with 6-(2-bromophenethyl)-[1,1'-biphenyl]-3-ol (**1c**) (35.3 mg, 0.100 mmol) as the starting material, and a reaction time of 48 h. Purification by preparatory TLC (30% EtOAc in petroleum ether and 30% acetone in petroleum ether) afforded the title compound as a colorless solid (5.1 mg, 0.019 mmol, 19% yield, 87% ee).

^{1}H NMR (700 MHz, $CDCl_3$) δ 7.30 (1H, td, *J*=7.4, 1.2 Hz), 7.28 – 7.22 (3H, m), 7.18 – 7.14 (2H, m), 7.12 – 7.09 (1H, m), 6.92 (1H, d, *J*=9.9 Hz), 6.78 – 6.74 (2H, m), 6.39 (1H, d, *J*=1.8 Hz), 6.29 (1H, dd, *J*=9.9, 1.8 Hz), 3.00 (1H, ddd, *J*=15.9, 9.0, 6.2 Hz), 2.55 (1H, ddd, *J*=15.9, 9.0, 5.9 Hz), 2.35 (1H, ddd, *J*=13.5, 9.0, 6.2 Hz), 2.28 (1H, ddd, *J*=13.5, 9.0, 5.9 Hz).

^{13}C NMR (176 MHz, $CDCl_3$) δ 187.2, 163.3, 154.3, 145.0, 141.9, 138.9, 128.6, 128.6, 128.6, 128.2, 128.0, 127.6, 125.5, 125.2, 124.6, 56.8, 36.0, 31.5.

Chiral SFC Analysis: CHIRALPAK IG (CO_2:MeOH, 85:15, 2.5 mL min^{-1}, 40 °C, 220 nm) indicated 87% ee, t_R = 5.89 (minor), 8.44 (major) minutes.

$[\alpha]_D^{25}$ = +317° (c 0.42, $CHCl_3$). Literature value: **$[\alpha]_D^{25}$** = +407° (c 1.78, $CHCl_3$) for 99% ee.

Data in agreement with the literature.[2]

(*S*)-3-Phenyl-2',3'-dihydrospiro[cyclohexane-1,1'-indene]-2,5-dien-4-one (2d)

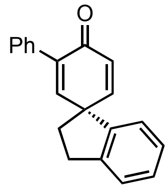

Prepared according to General Procedure J with 5-(2-bromophenethyl)-[1,1'-biphenyl]-2-ol (**1d**) (35.3 mg, 0.100 mmol) as the starting material, and a reaction time of 48 h. Purification by flash

column chromatography (0-10% EtOAc in petroleum ether) afforded the title compound as a yellow solid (19.0 mg, 0.070 mmol, 70% yield, 86% ee).

^{1}H NMR (500 MHz, $CDCl_3$) δ 7.45 – 7.41 (2H, m), 7.40 – 7.25 (5H, m), 7.20 (1H, t, *J*=7.5 Hz), 7.02 (1H, d, *J*=7.5 Hz), 6.98 (1H, d, *J*=2.9 Hz), 6.95 (1H, dd, *J*=9.7, 2.9 Hz), 6.42 (1H, d, *J*=9.7 Hz), 3.21 (2H, t, *J*=7.3 Hz), 2.44 (2H, t, *J*=7.3 Hz).

^{13}C NMR (126 MHz, $CDCl_3$) δ 185.0, 151.7, 150.3, 144.0, 142.8, 137.7, 136.0, 129.0, 128.4, 128.3, 128.2, 128.0, 127.4, 125.6, 124.3, 54.4, 37.9, 31.2.

HRMS m/z: $[M+H]^+$ calculated for $[C_{20}H_{17}O]^+$ 273.1274, found 273.1278. Δ = +1.5 ppm.

Chiral SFC Analysis: CHIRALPAK IE (CO_2:MeOH, 85:15, 2.5 mL min^{-1}, 40 °C, 220 nm) indicated 86% ee, t_R = 7.97 (minor), 8.60 (major) minutes.

$[\alpha]_D^{25}$ = +82.5° (c 0.95, $CHCl_3$).

(*S*)-3-Methoxy-2',3'-dihydrospiro[cyclohexane-1,1'-indene]-2,5-dien-4-one (2e)

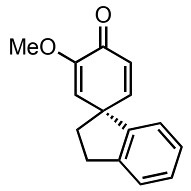

Prepared according to General Procedure J with 4-(2-bromophenethyl)-2-methoxyphenol (**1e**) (30.7 mg, 0.100 mmol) as the starting material, and a reaction time of 48 h. Purification by flash column chromatography (20% EtOAc in petroleum ether) afforded the title compound as a yellow solid (17.4 mg, 0.077 mmol, 77% yield, 91% ee).

^{1}H NMR (400 MHz, $CDCl_3$) δ 7.33 (1H, d, *J*=7.5 Hz), 7.25 (1H, td, *J*=7.5, 1.2 Hz), 7.17 (1H, t, *J*=7.5 Hz), 6.95 (1H, d, *J*=7.5 Hz), 6.91 (1H, dd, *J*=9.8, 2.6 Hz), 6.29 (1H, d, *J*=9.8 Hz), 5.86 (1H, d, *J*=2.6 Hz), 3.65 (3H, s), 3.18 (2H, t, *J*=7.2 Hz), 2.45 – 2.30 (2H, m).

^{13}C NMR (101 MHz, $CDCl_3$) δ 181.5, 153.0, 150.6, 143.6, 143.4, 128.2, 127.3, 126.7, 125.5, 124.0, 120.5, 55.0, 54.4, 38.7, 31.1.

HRMS m/z: $[M+H]^+$ calculated for $[C_{15}H_{14}O_2]^+$ 227.1067, found 227.1067. Δ = 0.0 ppm.

Chiral SFC Analysis: CHIRALPAK IG (CO_2:MeOH, 90:10, 2.5 mL min^{-1}, 40 °C, 220 nm) indicated 91% ee, t_R = 8.44 (minor), 9.41 (major) minutes.

[α]$_D$^{25} = +119° (c 0.87, $CHCl_3$).

(*S*)-3-Methyl-2',3'-dihydrospiro[cyclohexane-1,1'-indene]-2,5-dien-4-one (2f)

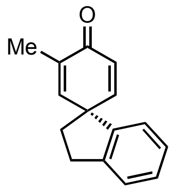

Prepared according to a modification of General Procedure J with 4-(2-bromophenethyl)-2-methylphenol (**1f**) (29.1 mg, 0.100 mmol) as the starting material, double the usual loadings of KOH (16.8 mg, 0.30 mmol, 3.0 equiv.), (*R*)–sSPhos (7.6 mg, 0.015 mmol, 15 mol%) and Pd_2dba_3 (4.6 mg, 0.0050 mmol, 5.0 mol%), and a reaction time of 48 h. Purification by preparatory TLC (10% EtOAc in PhMe and 30% acetone in petroleum ether) afforded the title compound as a colorless solid (12.3 mg, 0.058 mmol, 58% yield, 92% ee).

^{1}H NMR (400 MHz, $CDCl_3$) δ 7.32 (1H, dt, *J*=7.5, 1.2 Hz), 7.27 – 7.21 (1H, m), 7.16 (1H, td, *J*=7.5, 1.2 Hz), 6.92 (1H, d, *J*=7.5 Hz), 6.88 (1H, dd, *J*=9.8, 2.9 Hz), 6.71 (1H, dq, *J*=2.9, 1.4 Hz), 6.29 (1H, d, *J*=9.8 Hz), 3.16 (2H, t, *J*=7.3 Hz), 2.33 (2H, td, *J*=7.3, 1.0 Hz), 1.92 (3H, d, *J*=1.4 Hz).

^{13}C NMR (126 MHz, $CDCl_3$) δ 187.1, 152.6, 148.3, 144.0, 143.0, 133.8, 128.2, 127.4, 127.2, 125.5, 124.2, 54.1, 37.7, 31.2, 16.1.

HRMS m/z: $[M+H]^+$ calculated for $[C_{15}H_{15}O]^+$ 211.1117, found 211.1113. Δ = –1.9 ppm.

Chiral HPLC Analysis: CHIRALPAK IG (Hexane: ^{i}PrOH, 97:03, 1.25 mL min^{-1}, 40 °C, 250 nm) indicated 92% ee, t_R = 11.01 (minor), 12.53 (major) minutes.

[α]$_D$^{25} = +16.3° (c 0.82, $CHCl_3$).

(*S*)-3-Fluoro-2',3'-dihydrospiro[cyclohexane-1,1'-indene]-2,5-dien-4-one (2g)

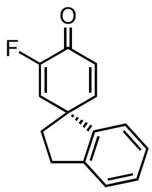

Prepared according to a modification of General Procedure J with 4-(2-bromophenethyl)-2-fluorophenol (**1g**) (29.5 mg, 0.100 mmol) as the starting material, double the usual loadings of (*R*)–sSPhos (7.6 mg, 0.015 mmol, 15 mol%) and Pd_2dba_3 (4.6 mg, 0.0050 mmol, 5.0 mol%), and a reaction time of 48 h. Purification by flash column chromatography (10% EtOAc in petroleum ether) and preparatory TLC (30% EtOAc in petroleum ether) afforded the title compound as a colorless solid (5.0 mg, 0.023 mmol, 23% yield, 88% ee).

^{1}H NMR (500 MHz, $CDCl_3$) δ 7.34 (1H, d, *J*=7.6 Hz), 7.31 – 7.25 (1H, m), 7.20 (1H, t, *J*=7.6 Hz), 6.97 (1H, d, *J*=7.6 Hz), 6.93 (1H, dd, *J*=9.8, 2.6 Hz), 6.47 (1H, dd, *J*=12.7, 2.6 Hz), 6.31 (1H, dd, *J*=9.8, 7.0 Hz), 3.18 (2H, t, *J*=7.3 Hz), 2.48 – 2.33 (2H, m).

^{13}C NMR (126 MHz, $CDCl_3$) δ 179.0 (d, *J*=21.2 Hz), 153.4 (d, *J*=263.8 Hz), 153.3 (d, *J*=2.5 Hz), 143.7, 141.5 (d, *J*=2.0 Hz), 128.7, 128.4 (d, *J*=11.5 Hz), 127.5, 126.9 (d, *J*=4.4 Hz), 125.7, 124.1, 54.9 (d, *J*=5.6 Hz), 37.8 (d, *J*=2.0 Hz), 31.2.

^{19}F NMR (376 MHz, $CDCl_3$) δ -131.9.

HRMS m/z: $[M+H]^+$ calculated for $[C_{14}H_{12}FO]^+$ 215.0867, found 215.0862. Δ = –2.3 ppm.

Chiral SFC Analysis: CHIRALPAK IA (CO_2:MeOH, 96:04, 2.5 mL min^{-1}, 40 °C, 240 nm) indicated 88% ee, t_R= 5.05 (minor), 5.39 (major) minutes.

$[\alpha]_D^{25}$ = +32.1° (c 0.33, $CHCl_3$).

(*R*)-2,3-Dihydro-4'*H*-spiro[indene-1,1'-naphthalen]-4'-one (2h)

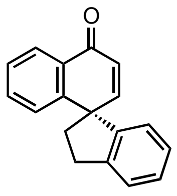

Prepared according to General Procedure J with 4-(2-bromophenethyl)naphthalen-1-ol (**1h**) (32.7 mg, 0.100 mmol) as the starting material. Purification by flash column chromatography (10-20%

EtOAc in petroleum ether) afforded the title compound as a yellow solid (24.4 mg, 0.099 mmol, 99%, 95% ee).

^{1}H NMR (400 MHz, $CDCl_3$) δ 8.22 (dd, *J* = 7.8, 1.6 Hz, 1H), 7.47 (td, *J* = 7.5, 1.6 Hz, 1H), 7.41 – 7.36 (m, 2H), 7.29 – 7.23 (m, 1H), 7.12 (td, *J* = 7.8, 1.2 Hz, 2H), 7.01 (d, *J* = 10.1 Hz, 1H), 6.71 (d, *J* = 7.6 Hz, 1H), 6.41 (d, *J* = 10.1 Hz, 1H), 3.38 – 3.21 (m, 2H), 2.69 – 2.50 (m, 2H).

^{13}C NMR (101 MHz, $CDCl_3$) δ 185.3, 153.3, 149.0, 146.4, 143.8, 132.9, 131.4, 128.3, 128.0, 127.5, 127.1, 126.5, 125.7, 125.2, 124.9, 54.5, 41.9, 31.5.

Chiral SFC Analysis: CHIRALPAK IG (CO_2:MeOH, 90:10, 2.5 mL min^{-1}, 40 °C, 220 nm) indicated 95% ee, t_R= 8.54 (minor), 10.48 (major) minutes.

$[\alpha]_D^{25}$ = +182° (c. 0.31, $CHCl_3$).

Data in agreement with the literature.[5]

(*S*)-2,3-Dimethyl-2',3'-dihydrospiro[cyclohexane-1,1'-indene]-2,5-dien-4-one (2i)

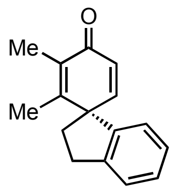

Prepared according to General Procedure J with 4-(2-bromophenethyl)-2,3-dimethylphenol (**1i**) (30.5 mg, 0.100 mmol) as the starting material, and double the usual loadings of KOH (16.8 mg, 0.30 mmol, 3.0 equiv.). Purification by flash column chromatography (10% EtOAc in petroleum ether) afforded the title compound as an off-white solid (19.4 mg, 0.086 mmol, 86% yield, 88% ee).

^{1}H NMR (500 MHz, $CDCl_3$) δ 7.33 (dt, *J* = 7.5, 1.1 Hz, 1H), 7.26 (td, *J* = 7.5, 1.2 Hz, 1H), 7.17 (td, *J* = 7.5, 1.1 Hz, 1H), 6.86 (d, *J* = 9.8 Hz, 1H), 6.80 (ddd, *J* = 7.5, 1.2, 0.6 Hz, 1H), 6.22 (d, *J* = 9.8 Hz, 1H), 3.31 – 3.15 (m, 2H), 2.46 (ddd, *J* = 13.4, 9.2, 8.2 Hz, 1H), 2.23 (ddd, *J* = 13.4, 8.6, 4.6 Hz, 1H), 1.98 (d, *J* = 0.9 Hz, 3H), 1.75 (d, *J* = 0.9 Hz, 3H).

^{13}C NMR (126 MHz, $CDCl_3$) δ 186.3, 155.5, 152.6, 144.2, 143.8, 132.7, 128.1, 127.3, 125.4, 124.9, 124.1, 57.5, 35.9, 31.7, 17.4, 11.2.

HRMS m/z: $[M+H]^+$ calculated for $[C_{16}H_{17}O]^+$ 225.1274, found 225.1275. Δ = +0.5 ppm.

Chiral SFC Analysis: CHIRALPAK IG (CO_2:MeOH, 90:10, 2.5 mL min^{-1}, 40 °C, 220 nm) indicated 88% ee, t_R= 5.73 (minor), 7.09 (major) minutes.

$[\alpha]_D^{25}$ = + 86.7° (c. 0.38, $CHCl_3$).

(*R*)-2-Methoxy-3',4'-dihydro-2'*H*-spiro[cyclohexane-1,1'-naphthalene]-2,5-dien-4-one (2j)

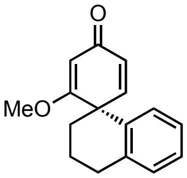

Prepared according to General Procedure J with 4-(3-(2-bromophenyl)propyl)-3-methoxyphenol (**1j**) (32.1 mg, 0.100 mmol) as the starting material. Purification by flash column chromatography (20-40% EtOAc in petroleum ether) afforded the title compound as a colorless solid (18.6 mg, 0.077 mmol, 77% yield, 93% ee).

^{1}H NMR (400 MHz, $CDCl_3$) δ 7.19 – 7.13 (2H, m), 7.07 (1H, ddd, *J*=8.5, 6.0, 2.8 Hz), 6.86 (1H, d, *J*=7.7 Hz), 6.81 (1H, d, *J*=9.9 Hz), 6.11 (1H, dd, *J*=9.9, 1.6 Hz), 5.72 (1H, d, *J*=1.6 Hz), 3.65 (3H, s), 3.01 – 2.75 (2H, m), 2.34 (1H, ddd, *J*=13.1, 11.4, 3.2 Hz), 2.12 – 1.99 (1H, m), 2.00 – 1.82 (2H, m).

^{13}C NMR (101 MHz, $CDCl_3$) δ 188.5, 180.2, 151.1, 137.3, 134.3, 130.1, 127.9, 127.4, 126.6, 124.3, 102.8, 55.9, 47.1, 34.0, 29.7, 19.5.

HRMS m/z: $[M+H]^+$ calculated for $[C_{16}H_{17}O_2]^+$ 241.1223, found 241.1219. Δ = –1.7 ppm.

Chiral SFC Analysis: CHIRALPAK IG (CO_2:MeOH, 90:10, 2.5 mL min^{-1}, 40 °C, 220 nm) indicated 93% ee, t_R = 7.43 (minor), 9.69 (major) minutes.

$[\alpha]_D^{25}$ = +122° (c 1.24, $CHCl_3$).

(*R*)-2'-Methoxy-6,7,8,9-tetrahydrospiro[benzo[7]annulene-5,1'-cyclohexane]-2',5'-dien-4'-one (2k)

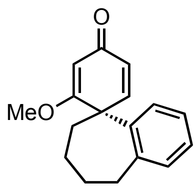

Prepared according to General Procedure J with 4-(4-(2-bromophenyl)butyl)-3-methoxyphenol (**1k**) (33.5 mg, 0.100 mmol) as the starting material, and a reaction time of 92 h. Purification by preparatory TLC (40% EtOAc in petroleum ether) afforded the title compound as a yellow solid (3.3 mg, 0.013 mmol, 13% yield, 88% ee).

^{1}H NMR (500 MHz, $CDCl_3$) δ 7.18 – 7.05 (3H, m), 6.96 (1H, d, *J*=9.9 Hz), 6.89 (1H, d, *J*=7.8 Hz), 6.15 (1H, dd, *J*=9.9, 1.8 Hz), 5.61 (1H, d, *J*=1.8 Hz), 3.69 (3H, s), 3.22 (1H, ddd, *J*=14.3, 8.6, 5.1 Hz), 2.80 (1H, dt, *J*=18.4, 4.7 Hz), 2.53 (1H, dd, *J*=13.2, 10.5 Hz), 1.94 – 1.71 (5H, m).

^{13}C NMR (126 MHz, $CDCl_3$) δ 188.3, 181.0, 150.5, 141.6, 137.6, 131.6, 129.7, 127.7, 126.8, 124.7, 101.4, 55.9, 53.1, 34.7, 33.1, 26.7, 22.1.

HRMS m/z: $[M+H]^+$ calculated for $[C_{17}H_{19}O_2]^+$ 255.1380, found 255.1372. Δ = –3.1 ppm.

Chiral SFC Analysis: CHIRALPAK IK (CO_2:MeOH, 90:10, 2.5 mL min^{-1}, 40 °C, 220 nm) indicated 88% ee, t_R = 8.01 (major), 8.45 (minor) minutes.

$[\alpha]_D^{25}$ = +43.6° (c 0.22, $CHCl_3$).

(*R*)-2-Methoxy-5'-(trifluoromethyl)-2',3'-dihydrospiro[cyclohexane-1,1'-indene]-2,5-dien-4-one (2l)

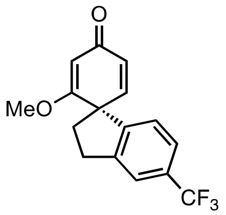

Prepared according to General Procedure J with 4-(2-bromo-5-(trifluoromethyl)phenethyl)-3-methoxyphenol (**1l**) (37.5 mg, 0.100 mmol) as the starting material. Purification by flash column chromatography (20-30% EtOAc in petroleum ether) afforded the title compound as a colorless viscous oil (20.4 mg, 0.069 mmol, 69% yield, 88% ee).

^{1}H NMR (500 MHz, $CDCl_3$) δ 7.59 – 7.53 (m, 1H), 7.42 (ddd, *J* = 8.0, 1.8, 0.9 Hz, 1H), 6.99 (d, *J* = 8.0 Hz, 1H), 6.58 (d, *J* = 9.8 Hz, 1H), 6.21 (dd, *J* = 9.8, 1.5 Hz, 1H), 5.66 (d, *J* = 1.5 Hz, 1H), 3.65 (s, 3H), 3.30 (ddd, *J* = 15.6, 8.9, 6.1 Hz, 1H), 3.17 (ddd, *J* = 15.6, 8.9, 5.9 Hz, 1H), 2.66 (ddd, *J* = 13.2, 8.9, 5.9 Hz, 1H), 2.31 (ddd, *J* = 13.2, 8.9, 6.1 Hz, 1H).

^{13}C NMR (126 MHz, $CDCl_3$) δ 188.3, 177.4, 147.9, 147.0 (d, *J* = 1.3 Hz), 145.6, 130.8 (q, *J* = 32.0 Hz), 126.3, 124.4 (q, *J* = 3.8 Hz), 124.3 (q, *J* = 272.4 Hz), 124.2, 122.3 (q, *J* = 3.8 Hz), 102.2, 56.0, 55.6, 36.7, 31.8.

^{19}F NMR (376 MHz, $CDCl_3$) δ -62.2.

HRMS m/z: $[M+H]^+$ calculated for $[C_{16}H_{14}F_3O_2]^+$ 295.0940, found 295.0940. Δ = 0.0 ppm.

Chiral SFC Analysis: CHIRALPAK IA (CO_2:MeOH, 95:05, 2.5 mL min^{-1}, 40 °C, 220 nm) indicated 88% ee, t_R= 5.13 (minor), 5.32 (major) minutes.

$[\alpha]_D^{25}$ = + 42.0° (c. 0.17, $CHCl_3$).

<u>1.0 mmol scale Reaction:</u>

A vial containing a magnetic stirrer bar was charged with 4-(2-bromo-5-(trifluoromethyl)phenethyl)-3-methoxyphenol (**1l**) (375 mg, 1.00 mmol, 1.00 equiv.), $Pd_2(dba)_3$ (9.2 mg, 0.010 mmol, 1.0 mol%), (*R*)-sSPhos (15.4 mg, 0.030 mmol, 3.0 mol%), and crushed KOH (84 mg, 1.5 mmol, 1.5 eq). The vial was sealed, and evacuated and backfilled with N_2 (3×). PhMe (5.0 mL) and H_2O (0.50 mL) were added under nitrogen. The reaction was stirred at 110 °C and 900 rpm for 20 h. The reaction mixture was quenched with sat. NH_4Cl (aq.) and extracted with chloroform (3×). The combined organic extracts were dried over $MgSO_4$, filtered, and the solvent removed under reduced pressure. The crude product was purified by flash column chromatography to afford the title compound as a colorless viscous oil (210 mg, 0.71 mmol, 71% yield, 90% ee).

^{1}H NMR (400 MHz, $CDCl_3$) δ 7.59 – 7.51 (m, 1H), 7.41 (ddd, *J* = 8.0, 1.8, 0.9 Hz, 1H), 6.98 (d, *J* = 8.0 Hz, 1H), 6.58 (d, *J* = 9.8 Hz, 1H), 6.19 (dd, *J* = 9.8, 1.5 Hz, 1H), 5.65 (d, *J* = 1.5 Hz, 1H), 3.64 (s, 3H), 3.29 (ddd, *J* = 15.7, 8.9, 6.2 Hz, 1H), 3.17 (ddd, *J* = 15.7, 8.9, 5.9 Hz, 1H), 2.66 (ddd, *J* = 13.2, 8.9, 5.9 Hz, 1H), 2.31 (ddd, *J* = 13.2, 8.9, 6.2 Hz, 1H).

^{13}C NMR (101 MHz, $CDCl_3$) δ 188.2, 177.4, 147.8, 147.0 (q, *J* = 1.3 Hz), 145.6, 130.8 (q, *J* = 31.9 Hz), 126.3, 124.4 (q, *J* = 3.7 Hz), 124.3 (q, *J* = 272.4 Hz), 124.1, 122.3 (q, *J* = 3.7 Hz), 102.2, 56.0, 55.6, 36.7, 31.8.

^{19}F NMR (376 MHz, $CDCl_3$) δ -62.2.

HRMS m/z: $[M+H]^+$ calculated for $[C_{16}H_{14}F_3O_2]^+$ 295.0940, found 295.0942. Δ = +0.9 ppm.

Chiral SFC Analysis: CHIRALPAK IA (CO_2:MeOH, 95:05, 2.5 mL min^{-1}, 40 °C, 250 nm) indicated 90% ee, t_R= 4.89 (minor), 5.08 (major) minutes.

$[\alpha]_D^{25}$= + 38.0° (c 0.66, $CHCl_3$).

(*R*)-2,5',6'-Trimethoxy-2',3'-dihydrospiro[cyclohexane-1,1'-indene]-2,5-dien-4-one (2m)

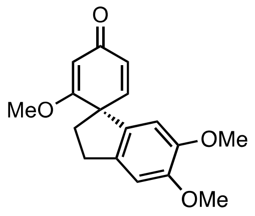

Prepared according to General Procedure J with 4-(2-bromo-4,5-dimethoxyphenethyl)-3-methoxyphenol (**1m**) (36.7 mg, 0.100 mmol) as the starting material. Purification by flash column chromatography (10-30% EtOAc in petroleum ether) afforded the title compound as a colorless viscous oil (15.8 mg, 0.055 mmol, 55% yield, 90% ee).

^{1}H NMR (400 MHz, $CDCl_3$) δ 6.81 (s, 1H), 6.60 (d, *J* = 9.7 Hz, 1H), 6.33 (s, 1H), 6.16 (dd, *J* = 9.7, 1.5 Hz, 1H), 5.63 (d, *J* = 1.5 Hz, 1H), 3.87 (s, 3H), 3.76 (s, 3H), 3.65 (s, 3H), 3.24 – 3.12 (m, 1H), 3.11 – 2.99 (m, 1H), 2.59 (ddd, *J* = 13.1, 8.9, 5.7 Hz, 1H), 2.25 (ddd, *J* = 13.1, 8.8, 6.1 Hz, 1H).

^{13}C NMR (101 MHz, $CDCl_3$) δ 188.8, 178.7, 149.7, 149.6, 148.8, 136.7, 134.0, 125.5, 107.9, 106.1, 101.9, 56.2, 56.1, 56.0, 55.9, 37.0, 31.9.

HRMS m/z: $[M+H]^+$ calculated for $[C_{17}H_{19}O_4]^+$ 287.1278, found 287.1269. Δ = –2.9 ppm.

Chiral SFC Analysis: CHIRALPAK IG (CO_2:MeOH, 90:10, 2.5 mL min^{-1}, 40 °C, 220 nm) indicated 90% ee, t_R= 6.20 (major), 12.95 (minor) minutes.

$[\alpha]_D^{25}$= + 79.3° (c. 0.28, $CHCl_3$).

(*R*)-4'-Chloro-2-methoxy-2',3'-dihydrospiro[cyclohexane-1,1'-indene]-2,5-dien-4-one (2n)

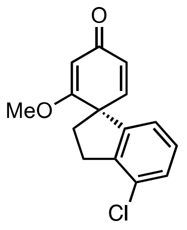

Prepared according to General Procedure J with 4-(2-bromo-6-chlorophenethyl)-3-methoxyphenol (**1n**) (34.1 mg, 0.100 mmol) as the starting material. Purification by flash column chromatography (10-20% EtOAc in petroleum ether) afforded the title compound as a colorless solid (21.6 mg, 0.083 mmol, 83% yield, 96% ee).

^{1}H NMR (400 MHz, $CDCl_3$) δ 7.24 (dd, *J* = 7.8, 1.0 Hz, 1H), 7.11 (tt, *J* = 7.8, 1.0 Hz, 1H), 6.77 (dd, *J* = 7.8, 1.0 Hz, 1H), 6.59 (d, *J* = 9.8 Hz, 1H), 6.17 (dd, *J* = 9.8, 1.6 Hz, 1H), 5.62 (d, *J* = 1.6 Hz, 1H), 3.64 (s, 3H), 3.34 – 3.21 (m, 1H), 3.15 (ddd, *J* = 16.5, 8.9, 5.7 Hz, 1H), 2.61 (ddd, *J* = 13.2, 9.0, 5.7 Hz, 1H), 2.27 (ddd, *J* = 13.2, 8.9, 6.2 Hz, 1H).

^{13}C NMR (101 MHz, $CDCl_3$) δ 188.4, 177.7, 148.3, 144.8, 143.2, 131.4, 128.8, 128.5, 125.9, 122.3, 101.9, 56.6, 56.0, 35.8, 31.4.

HRMS m/z: [M+H]$^+$ calculated for $[C_{15}H_{14}ClO_2]^+$ 261.0677, found 261.0674. Δ = –0.9 ppm.

Chiral SFC Analysis: CHIRALPAK IG (CO_2:MeOH, 90:10, 2.5 mL min^{-1}, 40 °C, 220 nm) indicated 96% ee, t_R= 7.15 (minor), 9.20 (major) minutes.

$[\alpha]_D^{25}$ = + 31.8° (c. 0.31, $CHCl_3$).

***tert*-Butyl (*R*)-(2-methoxy-4-oxo-2',3'-dihydrospiro[cyclohexane-1,1'-indene]-2,5-dien-6'-yl)Carbamate (2o)**

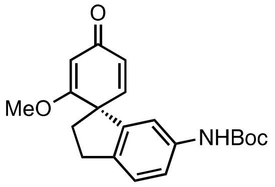

Prepared according to General Procedure J with *tert*-butyl (3-bromo-4-(4-hydroxy-2-methoxyphenethyl)phenyl)carbamate (**1o**) (42.2 mg, 0.100 mmol) as the starting material. Purification by flash column chromatography (30% EtOAc in petroleum ether) afforded the title compound as a pale yellow solid (12.7 mg, 0.037 mmol, 37% yield, 86% ee).

^{1}H NMR (400 MHz, $CDCl_3$) δ 7.34 – 7.27 (m, 1H), 7.20 (d, *J* = 8.2 Hz, 1H), 6.82 (d, *J* = 2.1 Hz, 1H), 6.59 (d, *J* = 9.8 Hz, 1H), 6.45 (s, 1H), 6.16 (dd, *J* = 9.8, 1.6 Hz, 1H), 5.62 (d, *J* = 1.6 Hz, 1H), 3.64 (s, 3H), 3.18 (ddd, *J* = 15.3, 8.8, 6.1 Hz, 1H), 3.05 (ddd, *J* = 15.3, 8.7, 5.9 Hz, 1H), 2.59 (ddd, *J* = 13.2, 8.8, 5.9 Hz, 1H), 2.24 (ddd, *J* = 13.2, 8.7, 6.1 Hz, 1H), 1.47 (s, 9H).

^{13}C NMR (101 MHz, $CDCl_3$) δ 188.6, 178.3, 152.8, 149.0, 143.7, 139.3, 137.6, 125.7, 125.5, 119.2, 114.1, 102.0, 80.7, 60.5, 55.9, 37.1, 31.3, 28.5.

HRMS m/z: $[M+H]^+$ calculated for $[C_{20}H_{24}NO_4]^+$ 342.1700, found 342.1693. Δ = –1.9 ppm.

Chiral SFC Analysis: CHIRALPAK IG (CO_2:MeOH, 85:15, 2.5 mL min^{-1}, 40 °C, 206 nm), indicated 86% ee, t_R= 8.60 (major), 9.86 (minor) minutes.

$[\alpha]_D^{25}$ = + 50.8° (c. 0.14, $CHCl_3$).

***tert*-Butyl (*R*)-2-methoxy-4-oxo-2',3'-dihydrospiro[cyclohexane-1,1'-indene]-2,5-diene-6'-carboxylate (2p)**

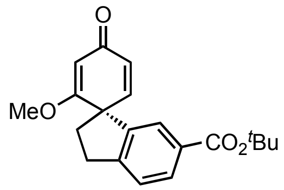

Prepared according to General Procedure J with *tert*-butyl 3-bromo-4-(4-hydroxy-2-methoxyphenethyl)benzoate (**1p**) (40.7 mg, 0.100 mmol) as the starting material. Purification by flash column chromatography (20-40% EtOAc in petroleum ether) afforded the title compound as a colorless viscous oil (26.2 mg, 0.080 mmol, 80% yield, 91% ee).

^{1}H NMR (400 MHz, $CDCl_3$) δ 7.89 (dd, *J* = 7.9, 1.6 Hz, 1H), 7.47 (d, *J* = 1.6 Hz, 1H), 7.33 (dd, *J* = 7.9, 0.9 Hz, 1H), 6.58 (d, *J* = 9.8 Hz, 1H), 6.20 (dd, *J* = 9.8, 1.5 Hz, 1H), 5.64 (d, *J* = 1.5 Hz, 1H), 3.64 (s, 3H), 3.33 – 3.23 (m, 1H), 3.18 – 3.09 (m, 1H), 2.62 (ddd, *J* = 13.2, 8.9, 5.6 Hz, 1H), 2.29 (ddd, *J* = 13.2, 8.9, 6.5 Hz, 1H), 1.55 (s, 9H).

^{13}C NMR (101 MHz, $CDCl_3$) δ 188.4, 177.8, 165.6, 149.8, 148.4, 143.3, 131.4, 129.8, 126.1, 125.0, 124.9, 102.0, 81.3, 55.9, 55.5, 36.9, 32.0, 28.3.

HRMS m/z: $[M+H]^+$ calculated for $[C_{20}H_{23}O_4]^+$ 327.1591, found 327.1590. Δ = – 0.3 ppm.

Chiral SFC Analysis: CHIRALPAK IG (CO_2:MeOH, 87:13, 2.5 mL min^{-1}, 40 °C, 230 nm) indicated 91% ee, t_R = 5.02 (major), 5.39 (minor) minutes.

[α]D[25] = + 26.5° (c. 1.30, $CHCl_3$).

(*R*)-2-Methoxy-7'-methyl-2',3'-dihydrospiro[cyclohexane-1,1'-indene]-2,5-dien-4-one (2q)

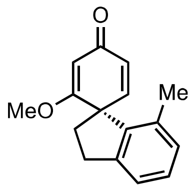

Prepared according to General Procedure J with 1-(4-(benzyloxy)-2-methoxyphenethyl)-2-bromo-3-methylbenzene (**1q**) (32.1 mg, 0.100 mmol) as the starting material. Purification by flash column chromatography (20-40% EtOAc in petroleum ether) afforded the title compound as a colorless viscous oil (17.2 mg, 0.072 mmol, 72% yield, 74% ee).

^{1}H NMR (400 MHz, $CDCl_3$) δ 7.21 – 7.11 (m, 2H), 6.98 – 6.89 (m, 1H), 6.65 (d, *J* = 9.8 Hz, 1H), 6.22 (dd, *J* = 9.8, 1.5 Hz, 1H), 5.73 (d, *J* = 1.5 Hz, 1H), 3.70 (s, 3H), 3.23 – 3.02 (m, 2H), 2.58 (ddd, *J* = 12.9, 9.1, 8.1 Hz, 1H), 2.20 (ddd, *J* = 12.9, 8.1, 4.8 Hz, 1H), 1.99 (s, 3H).

^{13}C NMR (101 MHz, $CDCl_3$) δ 188.4, 178.4, 148.1, 145.1, 140.3, 135.5, 129.0, 128.6, 126.2, 122.9, 102.9, 56.2, 56.0, 38.7, 31.5, 17.7.

HRMS m/z: $[M+H]^+$ calculated for $[C_{16}H_{17}O_2]^+$ 241.1223, found 241.1220. Δ = –1.3 ppm.

Chiral SFC Analysis: CHIRALPAK IG (CO_2:MeOH, 90:10, 2.5 mL min^{-1}, 40 °C, 220 nm) indicated 74% ee, t_R= 6.62 (minor), 7.52 (major) minutes.

[α]D[25] = + 24.0° (c. 0.18, $CHCl_3$).

(*R*)-5'-Fluoro-2-methoxy-2',3'-dihydrospiro[cyclohexane-1,1'-indene]-2,5-dien-4-one (2r)

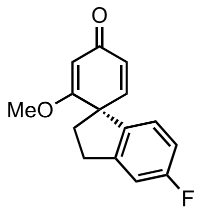

Prepared according to General Procedure J with 4-(2-bromo-5-fluorophenethyl)-3-methoxyphenol (**1r**) (32.5 mg, 0.100 mmol) as the starting material. Purification by flash column

chromatography (20-30% EtOAc in petroleum ether) afforded the title compound as a colorless viscous oil (20.5 mg, 0.084 mmol, 84% yield, 91% ee).

^{1}H NMR (400 MHz, $CDCl_3$) δ 6.97 (ddd, *J* = 8.8, 2.3, 1.2 Hz, 1H), 6.88 – 6.76 (m, 2H), 6.58 (d, *J* = 9.7 Hz, 1H), 6.17 (dd, *J* = 9.7, 1.6 Hz, 1H), 5.61 (d, *J* = 1.6 Hz, 1H), 3.64 (s, 3H), 3.23 (dddd, *J* = 16.2, 8.9, 6.3, 1.0 Hz, 1H), 3.15 – 3.03 (m, 1H), 2.62 (ddd, *J* = 13.1, 8.9, 5.7 Hz, 1H), 2.28 (ddd, *J* = 13.1, 8.8, 6.3 Hz, 1H).

^{13}C NMR (101 MHz, $CDCl_3$) δ 188.5, 178.1, 163.2 (d, *J* = 245.9 Hz), 148.8, 147.1 (d, *J* = 8.4 Hz), 138.3 (d, *J* = 2.3 Hz), 125.8, 124.9 (d, *J* = 9.1 Hz), 114.3 (d, *J* = 23.0 Hz), 112.3 (d, *J* = 22.5 Hz), 101.9, 55.9, 55.0, 37.0, 31.9 (d, *J* = 2.2 Hz).

^{19}F NMR (376 MHz, $CDCl_3$) δ -114.6.

HRMS m/z: $[M+H]^+$ calculated for $[C_{15}H_{14}FO_2]^+$ 245.0972, found 245.0969. Δ = –1.6 ppm.

Chiral SFC Analysis: CHIRALPAK IG (CO_2:MeOH, 90:10, 2.5 mL min^{-1}, 40 °C, 220 nm) indicated 91% ee, t_R= 5.58 (minor), 6.30 (major) minutes.

$[\alpha]_D^{25}$ = +40.3° (c. 0.58, $CHCl_3$).

<u>1 mmol reaction:</u>

A vial containing a magnetic stirrer bar was charged with 4-(2-bromo-5-fluorophenethyl)-3-methoxyphenol (**1r**) (325 mg, 1.00 mmol, 1.00 equiv.), $Pd_2(dba)_3$ (23 mg, 0.025 mmol, 2.5 mol%), (*R*)-sSPhos (38 mg, 0.075 mmol, 7.5 mol%), and crushed KOH (84 mg, 1.5 mmol, 1.5 equiv.). The vial was sealed, and evacuated and backfilled with N_2 (3×). PhMe (5.0 mL) and H_2O (0.50 mL) were added under nitrogen. The reaction was stirred at 110°C and 900 rpm for 20 h. The reaction mixture was quenched with sat. NH_4Cl (aq.) and extracted with chloroform (3×). The combined organic extracts were dried over $MgSO_4$, filtered, and the solvent removed under reduced pressure. The crude product was purified by flash column chromatography to afford the title compound as a colorless viscous oil (224 mg, 0.92 mmol, 92% yield, 95% ee).

^{1}H NMR (400 MHz, $CDCl_3$) δ 6.96 (ddt, *J* = 8.8, 1.8, 0.9 Hz, 1H), 6.87 – 6.77 (m, 2H), 6.58 (d, *J* = 9.8 Hz, 1H), 6.16 (dd, *J* = 9.8, 1.6 Hz, 1H), 5.61 (d, *J* = 1.6 Hz, 1H), 3.63 (s, 3H), 3.22 (dddd, *J* = 16.1, 8.9, 6.3, 1.0 Hz, 1H), 3.09 (dddd, *J* = 16.1, 8.9, 5.7, 1.0 Hz, 1H), 2.61 (ddd, *J* = 13.1, 8.9, 5.7 Hz, 1H), 2.28 (ddd, *J* = 13.1, 8.9, 6.3 Hz, 1H).

^{13}C NMR (101 MHz, $CDCl_3$) δ (101 MHz, $CDCl_3$) δ 188.4, 178.1, 163.1 (d, J = 245.8 Hz), 148.8, 147.1 (d, J = 8.3 Hz), 138.3 (d, J = 2.6 Hz), 125.7, 124.8 (d, J = 9.1 Hz), 114.2 (d, J = 23.0 Hz), 112.2 (d, J = 22.2 Hz), 101.8, 55.9, 55.0, 37.0, 31.9 (d, J = 2.1 Hz).

^{19}F NMR (376 MHz, $CDCl_3$) δ -114.6.

HRMS m/z: $[M+H]^+$ calculated for $[C_{15}H_{14}FO_2]^+$ 245.0972, found 245.0974. Δ = 0.8 ppm.

Chiral SFC Analysis: CHIRALPAK IG (CO_2:MeOH, 90:10, 2.5 mL min^{-1}, 40 °C, 220 nm) indicated 95% ee, t_R= 5.20 (minor), 5.90 (major) minutes.

$[\alpha]_D^{25}$ = + 45.3° (c 1.63, $CHCl_3$).

(*R*)-6'-Fluoro-2-methoxy-2',3'-dihydrospiro[cyclohexane-1,1'-indene]-2,5-dien-4-one (2s)

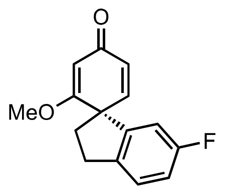

Prepared according to General Procedure J with 4-(2-bromo-4-fluorophenethyl)-3-methoxyphenol (**1s**) (32.5 mg, 0.100 mmol) as the starting material. Purification by flash column chromatography (10 – 20% EtOAc in petroleum ether) afforded the title compound as a light brown solid (19.5 mg, 0.080 mmol, 80% yield, 88% ee).

^{1}H NMR (400 MHz, $CDCl_3$) δ 7.25 – 7.17 (m, 1H), 6.93 (td, J = 8.6, 2.5 Hz, 1H), 6.59 (d, J = 9.8 Hz, 1H), 6.58 – 6.55 (m, 1H), 6.17 (dd, J = 9.8, 1.5 Hz, 1H), 5.64 (d, J = 1.5 Hz, 1H), 3.65 (s, 3H), 3.25 – 3.14 (m, 1H), 3.12 – 3.02 (m, 1H), 2.64 (ddd, J = 13.1, 8.9, 6.1 Hz, 1H), 2.28 (ddd, J = 13.1, 8.7, 5.9 Hz, 1H).

^{13}C NMR (101 MHz, $CDCl_3$) δ 188.2, 177.5, 162.3 (d, J = 245.3 Hz), 148.1, 144.8 (d, J = 7.4 Hz), 140.0 (d, J = 2.6 Hz), 126.1 (d, J = 8.6 Hz), 125.8, 115.3 (d, J = 22.3 Hz), 110.6 (d, J = 22.9 Hz), 102.1, 55.8, 55.7 (d, J = 2.1 Hz), 37.1, 31.0.

^{19}F NMR (376 MHz, $CDCl_3$) δ -116.9.

HRMS m/z: $[M+H]^+$ calculated for $[C_{15}H_{14}FO_2]^+$ 245.0972, found 245.0965. Δ = –3.0 ppm.

Chiral SFC Analysis: CHIRALPAK IG (CO_2:MeOH, 90:10, 2.5 mL min^{-1}, 40 °C, 220 nm), indicated 88% ee, t_R= 5.74 (minor), 6.24 (major) minutes.

[α]$_D$^{25} = + 51.2° (c. 0.25, $CHCl_3$).

Characterization of Products in Scheme 2

General Procedure K: Palladium/(*R*)–sSPhos-Catalyzed Dearomatization of *para*-Amino Phenols

A vial containing a magnetic stirrer bar was charged with *para*-amino phenol starting material (0.100 mmol), crushed KOH (8.4 mg, 0.15 mmol, 1.5 equiv.), (*R*)–sSPhos (1.9 mg, 0.00375 mmol, 3.75 mol%), and Pd_2dba_3 (1.1 mg, 0.00125 mmol, 1.25 mol%). The vial was sealed, and evacuated and backfilled with N_2 (3×). PhMe (0.50 mL) and H_2O (50 µL) were added under nitrogen. The reaction was stirred at 110 °C and 900 rpm for 20 h. Upon completion, the vial was opened, and the solvent was removed under a stream of air. Sat. NH_4Cl (aq.) (0.5 ml) was added, and the aqueous layer was extracted with chloroform (3 × 0.5 ml). The combined organic extracts were dried over $MgSO_4$ and purified by flash column chromatography.

(*R*)-5,6,8,9-Tetrahydro-3*H*-indolo[7a,1-*a*]isoquinolin-3-one (4a)

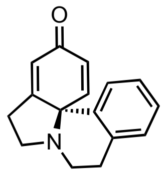

From **3a**: Prepared according to General Procedure K with 1-(2-bromophenethyl)indolin-5-ol (**3a**) (31.8 mg, 0.100 mmol) as the starting material. Purification by flash column chromatography (25% acetone in petroleum ether) afforded the title compound as an orange solid (15.2 mg, 0.064 mmol, 64% yield, 93% ee).

From **3a'**: Prepared according to General Procedure K with 1-(2-chlorophenethyl)indolin-5-ol (**3a'**) (27.3 mg, 0.100 mmol) as the starting material. Purification by flash column chromatography (25-50% acetone in petroleum ether) afforded the title compound as an orange solid (9.5 mg, 0.040 mmol, 40% yield, 89% ee).

^{1}H NMR (400 MHz, $CDCl_3$) δ 7.20 – 7.15 (1H, m), 7.14 – 7.07 (3H, m), 6.99 (1H, dd, *J*=7.8, 1.4 Hz), 6.30 – 6.22 (1H, m), 6.13 (1H, dd, *J*=9.8, 1.6 Hz), 3.58 – 3.46 (1H, m), 3.34 – 3.21 (3H, m), 3.15 (1H, td, *J*=9.9, 5.9 Hz), 2.72 – 2.51 (3H, m).

^{13}C NMR (101 MHz, $CDCl_3$) δ 187.4, 166.7, 147.9, 134.6, 131.3, 130.3, 127.7, 127.0, 126.9, 126.1, 123.2, 64.7, 46.8, 40.8, 28.1, 20.7.

Chiral SFC Analysis (from **3a**): CHIRALPAK IH (CO_2:MeOH, 90:10, 2.5 mL min^{-1}, 40 °C, 220 nm) indicated 93% ee, t_R = 7.56 (minor), 9.03 (major) minutes; (from **3a'**): CHIRALPAK IH (CO_2:MeOH, 90:10, 2.5 mL min^{-1}, 40 °C, 220 nm) indicated 89% ee, t_R = 7.70 (minor), 9.49 (major) minutes.

[α]$_D$^{25} = +142° (c 0.85, $CHCl_3$). Literature value: **[α]$_D$^{29}** = +241° (c 0.20, $CHCl_3$) for 94% ee)**.**[3]

Data in agreement with the literature.[3]

(*R*)-6,7,9,10-Tetrahydroisoquinolino[1,2-*j*]quinolin-3(5*H*)-one (4b)

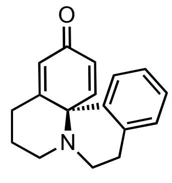

Prepared according to General Procedure K with 1-(2-bromophenethyl)-1,2,3,4-tetrahydroquinolin-6-ol (**3b**) (33.2 mg, 0.100 mmol) as the starting material. Purification by flash column chromatography (25% acetone in petroleum ether) afforded the title compound as an orange solid (17.9 mg, 0.071 mmol, 71% yield, 97% ee).

^{1}H NMR (500 MHz, $CDCl_3$) δ 7.27 (1H, d, *J*=10.2 Hz), 7.24 – 7.19 (1H, m), 7.18 – 7.13 (1H, m), 7.12 – 7.06 (1H, m), 6.86 (1H, dd, *J*=7.8, 1.4 Hz), 6.37 (1H, d, *J*=1.6 Hz), 6.13 – 6.05 (1H, m), 3.88 (1H, ddd, *J*=14.8, 11.8, 6.5 Hz), 3.28 (1H, ddd, *J*=18.2, 11.8, 8.0 Hz), 3.12 (1H, ddd, *J*=14.8, 8.0, 1.0 Hz), 2.94 (1H, td, *J*=11.8, 3.2 Hz), 2.89 – 2.82 (1H, m), 2.70 (1H, dd, *J*=17.9, 6.5 Hz), 2.48 – 2.40 (1H, m), 2.20 – 2.10 (1H, m), 1.95 – 1.75 (2H, m)

^{13}C NMR (126 MHz, $CDCl_3$) δ 186.7, 160.8, 149.6, 134.0, 133.0, 130.4, 128.0, 127.3, 127.0, 126.4, 125.1, 61.5, 48.7, 45.4, 33.0, 27.4, 21.7.

Chiral HPLC Analysis: CHIRALPAK IG (Hexane:^{i}PrOH, 85:15, 1.25 mL min^{-1}, 40 °C, 220 nm) indicated 97% ee, t_R = 11.82 (minor), 13.60 (major) minutes.

[α]$_D$^{25} = +140° (c 0.92, $CHCl_3$).

Data in agreement with the literature.[3]

(*R*)-11-Methoxy-5,6,8,9-tetrahydro-3*H*-indolo[7a,1-*a*]isoquinolin-3-one (4c)

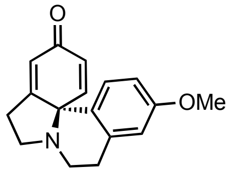

Prepared according to General Procedure K with 1-(2-bromo-5-methoxyphenethyl)indolin-5-ol (**3c**) (34.8 mg, 0.100 mmol) as the starting material. Purification by flash column chromatography (25-50% acetone in petroleum ether) afforded the title compound as a yellow solid (16.1 mg, 0.060 mmol, 60% yield, 88% ee).

^{1}H NMR (500 MHz, $CDCl_3$) δ 7.08 (1H, d, *J*=9.8 Hz), 6.90 (1H, d, *J*=8.6 Hz), 6.66 (1H, dd, *J*=8.6, 2.7 Hz), 6.62 (1H, d, *J*=2.7 Hz), 6.23 (1H, br s), 6.09 (1H, dd, *J*=9.8, 1.6 Hz), 3.76 (3H, s), 3.48 (1H, ddd, *J*=16.9, 13.3, 5.5 Hz), 3.31 – 3.18 (3H, m), 3.14 (1H, td, *J*=9.8, 5.9 Hz), 2.71 – 2.52 (3H, m).

^{13}C NMR (126 MHz, $CDCl_3$) δ 187.5, 166.8, 158.9, 148.2, 136.0, 128.3, 125.8, 123.0, 123.0, 114.6, 113.6, 64.3, 55.4, 46.7, 40.9, 28.1, 21.1.

Chiral SFC Analysis: CHIRALPAK IG (CO_2:MeOH, 85:15, 2.5 mL min^{-1}, 40 °C, 220 nm) indicated 88% ee, t_R = 10.53 (major), 13.54 (minor) minutes.

$[\alpha]_D^{25}$ = +114° (c 0.81, $CHCl_3$).

Data in agreement with the literature.[3]

(*R*)-11-Chloro-5,6,8,9-tetrahydro-3*H*-indolo[7a,1-*a*]isoquinolin-3-one (4d)

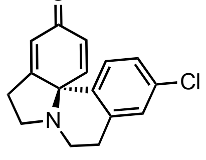

Prepared according to General Procedure K with 1-(2-bromo-5-chlorophenethyl)indolin-5-ol (**3d**) (35.2 mg, 0.100 mmol) as the starting material. Purification by flash column chromatography (25-50% acetone in petroleum ether) afforded the title compound as an orange solid (18.3 mg, 0.067 mmol, 67% yield, 94% ee).

^{1}**H NMR** ($CDCl_3$, 500 MHz) δ 7.12 (1H, d, *J*=2.2 Hz), 7.06 (2H, app dd, *J*=9.4, 2.9 Hz), 6.92 (1H, d, *J*=8.4 Hz), 6.30 – 6.23 (1H, m), 6.12 (1H, dd, *J*=9.9, 1.5 Hz), 3.48 (1H, ddd, *J*=13.9, 11.8, 5.6 Hz), 3.34 – 3.17 (3H, m), 3.11 (1H, td, *J*=10.0, 5.7 Hz), 2.73 – 2.51 (3H, m).

^{13}**C NMR** (126 MHz, $CDCl_3$) δ 187.2, 166.1, 147.4, 136.6, 133.5, 130.1, 130.0, 128.5, 127.4, 126.4, 123.4, 64.3, 46.8, 40.6, 28.0, 20.6.

HRMS m/z: $[M+H]^+$ calculated for $[C_{16}H_{15}ClNO]^+$ 272.0837, found 272.0838. Δ = +0.4 ppm.

Chiral SFC Analysis: CHIRALPAK IH (CO_2:MeOH, 90:10, 2.5 mL min^{-1}, 40 °C, 220 nm) indicated 94% ee, t_R = 8.21 (minor), 10.77 (major) minutes.

$[\alpha]_D^{25}$ = +162° (c 0.92, $CHCl_3$).

(*R*)-11-(trifluoromethyl)-5,6,8,9-Tetrahydro-3*H*-indolo[7a,1-*a*]isoquinolin-3-one (4e)

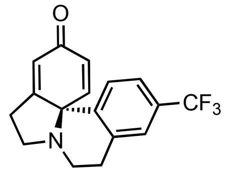

Prepared according to General Procedure K with 1-(2-bromo-5-(trifluoromethyl)phenethyl)indolin-5-ol (**3e**) (38.6 mg, 0.100 mmol) as the starting material. Purification by flash column chromatography (25-50% acetone in petroleum ether) afforded the title compound as a yellow solid (22.6 mg, 0.074 mmol, 74% yield, 92% ee).

^{1}**H NMR** (700 MHz, $CDCl_3$) δ 7.39 (1H, s), 7.33 (1H, d, *J*=8.2 Hz), 7.12 (1H, d, *J*=8.2 Hz), 7.08 (1H, d, *J*=9.8 Hz), 6.33 – 6.26 (1H, m), 6.16 (1H, d, *J*=9.8 Hz), 3.53 (1H, ddd, *J*=13.1, 11.3, 5.6 Hz), 3.37 – 3.26 (3H, m), 3.16 – 3.06 (1H, m), 2.73 – 2.66 (2H, m), 2.60 – 2.54 (1H, m).

^{13}**C NMR** (176 MHz, $CDCl_3$) δ 187.0, 165.8, 147.0, 136.0, 135.5, 130.0 (q, *J*=32.4 Hz), 127.7, 127.2 (q, *J*=3.8 Hz), 126.8, 123.9 (q, *J*=272.3 Hz), 123.7 (q, *J*=3.7 Hz), 123.6, 64.6, 46.9, 40.6, 28.0, 20.7.

^{19}**F NMR** (376 MHz, $CDCl_3$) δ -62.8.

Chiral SFC Analysis: CHIRALPAK IG (CO_2:MeOH, 85:15, 2.5 mL min^{-1}, 40 °C, 220 nm) indicated 92% ee, t_R = 3.35 (minor), 3.73 (major) minutes.

[α]$_D^{25}$ = +90.9° (c 1.13, $CHCl_3$).

Data in agreement with the literature.[3]

(*R*)-12,13-Dimethoxy-6,7,9,10-tetrahydroisoquinolino[1,2-*j*]quinolin-3(5*H*)-one (4f)

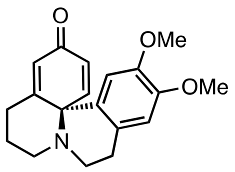

Prepared according to General Procedure K with 1-(2-bromo-4,5-dimethoxyphenethyl)-1,2,3,4-tetrahydroquinolin-6-ol **(3f)** (39.2 mg, 0.100 mmol) as the starting material. Purification by flash column chromatography (50% acetone in petroleum ether) afforded the title compound as a light brown solid (19.3 mg, 0.062 mmol, 62% yield, 91% ee).

^{1}H NMR (400 MHz, $CDCl_3$) δ 7.30 – 7.22 (1H, m), 6.61 (1H, s), 6.34 (1H, s), 6.27 (1H, s), 6.11 – 6.04 (1H, m), 3.84 (3H, s), 3.83 – 3.77 (1H, m), 3.67 (3H, s), 3.19 (1H, dtd, *J*=17.2, 7.8, 3.9 Hz), 3.08 (1H, ddd, *J*=14.6, 7.8, 3.0 Hz), 2.93 (1H, td, *J*=11.9, 3.0 Hz), 2.86 – 2.77 (1H, m), 2.58 (1H, ddd, *J*=17.2, 6.7, 2.6 Hz), 2.48 – 2.38 (1H, m), 2.14 (1H, tdd, *J*=13.1, 5.1, 2.0 Hz), 1.96 – 1.70 (2H, m).

^{13}C NMR (101 MHz, $CDCl_3$) δ 186.7, 161.0, 149.8, 148.9, 148.4, 126.8, 126.3, 124.8, 124.3, 112.5, 108.4, 61.1, 56.1, 55.9, 48.4, 45.4, 33.0, 27.3, 21.4.

Chiral HPLC Analysis: CHIRALPAK IG (Hexane:^{i}PrOH, 75:25, 1.25 mL min^{-1}, 40 °C, 220 nm) indicated 91% ee, t_R = 11.31 (major), 16.80 (minor) minutes.

[α]$_D^{25}$ = +379° (c 1.29, $CHCl_3$).

Data in agreement with the literature.[17]

(*R*)-11,12-Dimethoxy-5,6,8,9-tetrahydro-3*H*-indolo[7a,1-*a*]isoquinolin-3-one (4g)

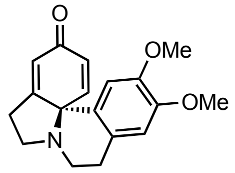

Prepared according to General Procedure K with 1-(2-bromo-4,5-dimethoxyphenethyl)indolin-5-ol (**3g**) (37.8 mg, 0.100 mmol) as the starting material. Purification by flash column chromatography (25-50% acetone in petroleum ether) afforded the title compound as an orange solid (14.9 mg, 0.050 mmol, 50% yield, 88% ee).

^{1}H NMR (400 MHz, $CDCl_3$) δ 7.10 (1H, d, *J*=9.8 Hz), 6.56 (1H, s), 6.40 (1H, s), 6.32 – 6.21 (1H, m), 6.13 (1H, dd, *J*=9.8, 1.6 Hz), 3.84 (3H, s), 3.69 (3H, s), 3.54 – 3.41 (1H, m), 3.32 – 3.06 (4H, m), 2.71 – 2.57 (2H, m), 2.52 (1H, dd, *J*=17.2, 5.6 Hz).

^{13}C NMR (101 MHz, $CDCl_3$) δ 187.4, 167.0, 148.8, 148.1, 148.1, 126.9, 126.0, 123.0, 122.6, 112.3, 108.7, 64.4, 56.0, 56.0, 46.7, 41.0, 28.1, 20.4.

Chiral SFC Analysis: CHIRALPAK IK (CO_2:MeOH, 85:15, 2.5 mL min^{-1}, 40 °C, 220 nm) indicated 88% ee, t_R = 9.75 (major), 11.66 (minor) minutes.

$[\alpha]_D^{25}$ = +160° (c 0.95, $CHCl_3$).

Data in agreement with the literature.[3]

Characterization of Products in Scheme 3

General Procedure L: Palladium/(*R*)–sSPhos-Catalyzed Dearomatization of *meta*-Tethered Phenols

A vial containing a magnetic stirrer bar was charged with phenol starting material (0.100 mmol), crushed KOH (16.8 mg, 0.300 mmol, 3.00 equiv.), (*R*)–sSPhos (3.8 mg, 0.0075 mmol, 7.5 mol%), and Pd_2dba_3 (2.3 mg, 0.0025 mmol, 2.5 mol%). The vial was sealed, and evacuated and backfilled with N_2 (3×). PhMe (0.50 mL) and H_2O (50 μL) were added under nitrogen. The reaction was stirred at 110 °C and 900 rpm for 20 h. Upon completion, the vial was opened, and the solvent was removed under a stream of air. Sat. NH_4Cl (aq.) (0.5 mL) was added, and the aqueous layer was extracted with chloroform (3 × 0.5 mL). The combined organic layers were dried over $MgSO_4$ and purified by flash column chromatography.

(*S*)-4a-Methyl-9,10-dihydrophenanthren-2(4a*H*)-one (7a)

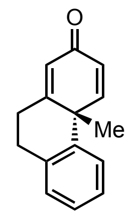

Prepared according to General Procedure L with 3-(2-bromophenethyl)-4-methylphenol (**6a**) (29.1 mg, 0.100 mmol) as the starting material. Purification by flash column chromatography (10-20% EtOAc in petroleum ether) afforded the title compound as a light yellow solid (12.5 mg, 0.59 mmol, 59%, 93% ee).

^{1}H NMR (400 MHz, $CDCl_3$) δ 7.50 – 7.44 (m, 2H), 7.30 – 7.25 (m, 1H), 7.21 – 7.11 (m, 2H), 6.31 (dd, *J* = 10.0, 1.9 Hz, 1H), 6.27 – 6.23 (m, 1H), 3.14 (ddd, *J* = 15.8, 6.1, 2.3 Hz, 1H), 3.05 – 2.95 (m, 1H), 2.88 (ddd, *J* = 12.3, 6.1, 1.4 Hz, 1H), 2.69 (ddd, *J* = 12.6, 5.5, 2.3 Hz, 1H), 1.62 (s, 3H).

^{13}C NMR (101 MHz, $CDCl_3$) δ 186.0, 166.4, 154.9, 139.7, 135.4, 129.5, 127.6, 127.1, 126.9, 126.2, 124.7, 43.8, 33.4, 33.1, 30.8.

HRMS m/z: $[M+H]^+$ calculated for $[C_{15}H_{15}O]^+$ 211.1117, found 211.1116. Δ = –0.8 ppm.

Chiral SFC Analysis: CHIRALPAK IG (CO_2:MeOH, 85:15, 1.25 mL min^{-1}, 40 °C, 240 nm) indicated 93% ee, t_R = 6.60 (major), 8.31 (minor) minutes.

$[\alpha]_D^{25}$ = +253° (c. 0.42, $CHCl_3$). Literature value: **$[\alpha]_D^{28}$** = –269° (c 0.62, $CHCl_3$) for (*R*)-**7a** enantiomer, 92% ee)**.**[4]

(*S*)-4a-Ethyl-9,10-dihydrophenanthren-2(4a*H*)-one (7b)

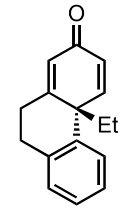

Prepared according to General Procedure L with 3-(2-bromophenethyl)-4-ethylphenol (**6b**) (30.5 mg, 0.100 mmol) as the starting material. Purification by flash column chromatography (10-20% EtOAc in petroleum ether) afforded the title compound as a yellow solid (17.5 mg, 0.078 mmol, 78% yield, 91% ee).

^{1}H NMR ($CDCl_3$, 400 MHz) δ 7.42 (1H, dd, *J*=7.9, 1.3 Hz), 7.31 (1H, d, *J*=10.1 Hz), 7.28 – 7.22 (1H, m), 7.18 (1H, td, *J*=7.3, 1.3 Hz), 7.16 – 7.12 (1H, m), 6.42 (1H, dd, *J*=10.1, 2.0 Hz), 6.39 – 6.33 (1H, m), 3.15 (1H, ddd, *J*=16.4, 6.7, 2.3 Hz), 3.06 – 2.95 (1H, m), 2.74 (1H, dddd, *J*=13.2, 11.8, 6.7, 1.5 Hz), 2.63 (1H, ddd, *J*=12.8, 6.2, 2.3 Hz), 2.13 – 1.91 (2H, m), 0.68 (3H, t, *J*=7.4 Hz).

^{13}C NMR (101 MHz, $CDCl_3$) δ 186.3, 163.9, 152.7, 140.4, 135.1, 130.0, 129.6, 126.9, 126.9 (2×C_{Ar}), 126.2, 48.3, 37.5, 33.0, 30.4, 8.6.

Chiral SFC Analysis: CHIRALPAK IE (CO_2:MeOH, 85:15, 2.5 mL min^{-1}, 40 °C, 220 nm) indicated 91% ee, t_R = 7.45 (major), 7.98 (minor) minutes.

$[\alpha]_D^{25}$ = +179° (c 0.82, $CHCl_3$).

Data in agreement with the literature.[4]

(*S*)-8-Chloro-4a-methyl-9,10-dihydrophenanthren-2(4a*H*)-one (7c)

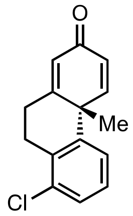

Prepared according to General Procedure L with 3-(2-bromo-6-chlorophenethyl)-4-methylphenol (**6c**) (32.6 mg, 0.100 mmol) as the starting material. Purification by flash column chromatography (30% Et_2O in petroleum ether and 10% EtOAc in PhMe) afforded the title compound as a colorless solid (4.7 mg, 0.019 mmol, 19% yield, 92% ee).

^{1}H NMR (400 MHz, $CDCl_3$) δ 7.47 – 7.39 (m, 2H), 7.31 (dd, *J* = 7.9, 1.4 Hz, 1H), 7.27 – 7.22 (m, 1H), 6.33 (dd, *J* = 10.1, 1.9 Hz, 1H), 6.30 – 6.27 (m, 1H), 3.47 – 3.37 (m, 1H), 2.93 – 2.83 (m, 2H), 2.81 – 2.74 (m, 1H), 1.67 (s, 3H).

^{13}C NMR (101 MHz, $CDCl_3$) δ 185.5, 165.0, 154.1, 141.9, 134.8, 133.4, 127.8, 127.8, 127.7, 124.7, 124.6, 43.5, 33.2, 30.8, 29.9.

HRMS m/z: $[M+H]^+$ calculated for $[C_{15}H_{14}ClO]^+$ 245.0728, found 245.0732. Δ = +1.6 ppm.

Chiral SFC Analysis: CHIRALPAK IA (CO_2:MeOH, 85:15, 2.5 mL min^{-1}, 40 °C, 224 nm) indicated 92% ee, t_R= 4.52 (minor), 4.96 (major) minutes.

[α]$_D^{25}$ = +155° (c. 0.19, $CHCl_3$).

(*S*)-7-Fluoro-4a-methyl-9,10-dihydrophenanthren-2(4a*H*)-one (7d)

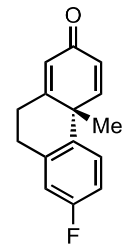

Prepared according to General Procedure L with 3-(2-bromo-5-fluorophenethyl)-4-methylphenol (**6d**) (30.9 mg, 0.100 mmol) as the starting material. Purification by flash column chromatography (10-20% EtOAc in petroleum ether) afforded the title compound as a pale yellow solid (16.2 mg, 0.071 mmol, 71%, 90% ee).

^{1}H NMR (400 MHz, $CDCl_3$) δ 7.45 – 7.39 (m, 2H), 6.97 (td, *J* = 8.5, 2.8 Hz, 1H), 6.83 (dd, *J* = 9.3, 2.8 Hz, 1H), 6.30 (dd, *J* = 10.0, 1.9 Hz, 1H), 6.26 – 6.21 (m, 1H), 3.11 (ddd, *J* = 15.7, 6.1, 2.2 Hz, 1H), 3.03 – 2.92 (m, 1H), 2.87 (ddd, *J* = 12.4, 6.1, 1.5 Hz, 1H), 2.68 (ddd, *J* = 12.7, 5.5, 2.2 Hz, 1H), 1.59 (s, 3H).

^{13}C NMR (101 MHz, $CDCl_3$) δ 185.7, 165.5, 161.2 (d, *J* = 246.6 Hz), 154.3, 137.6 (d, *J* = 7.4 Hz), 135.3, 127.7 (d, *J* = 8.3 Hz), 127.6, 124.8, 115.7 (d, *J* = 20.8 Hz), 114.2 (d, *J* = 21.3 Hz), 43.2, 33.3 (d, *J* = 1.2 Hz), 32.8 (d, *J* = 1.6 Hz), 30.4.

^{19}F NMR (376 MHz, $CDCl_3$) δ -116.0.

HRMS m/z: $[M+H]^+$ calculated for $[C_{15}H_{13}FO]^+$ 229.1023, found 229.1019. Δ = –1.7 ppm.

Chiral HPLC analysis: CHIRALPAK AD-H (Hexane: ^{i}PrOH, 90:10, 1.25 mL min^{-1}, 40 °C, 232 nm), indicated 90% ee, t_R= 7.33 (major), 8.45 (minor) minutes.

[α]$_D^{25}$ = + 230° (c. 0.52, $CHCl_3$).

(*S*)-9,9,11b-Trimethyl-6,11b-dihydrophenanthro[2,3-*d*][1,3]dioxol-3(5*H*)-one (7e)

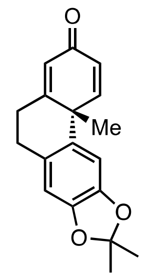

Prepared according to General Procedure L with 3-(2-(6-bromo-2,2-dimethylbenzo[*d*][1,3]dioxol-5-yl)ethyl)-4-methylphenol (**6e**) (36.3 mg, 0.100 mmol) as the starting material. Purification by flash column chromatography (20% EtOAc in petroleum ether) afforded the title compound as an orange solid (14.9 mg, 0.053 mmol, 53% yield, 90% ee).

^{1}H NMR (400 MHz, $CDCl_3$) δ 7.34 (d, *J* = 10.0 Hz, 1H), 6.80 (s, 1H), 6.47 (s, 1H), 6.27 (dd, *J* = 10.0, 1.9 Hz, 1H), 6.23 – 6.18 (m, 1H), 3.06 – 2.94 (m, 1H), 2.93 – 2.78 (m, 2H), 2.67 – 2.58 (m, 1H), 1.68 (s, 3H), 1.64 (s, 3H), 1.57 (s, 3H).

^{13}C NMR (101 MHz, $CDCl_3$) δ 186.2, 166.5, 154.9, 146.9, 146.5, 131.6, 128.1, 127.4, 124.4, 118.4, 108.7, 105.7, 43.7, 33.2, 33.1, 31.0, 26.0, 25.9.

HRMS m/z: $[M+H]^+$ calculated for $[C_{18}H_{19}O_3]^+$ 283.1329, found 283.1322. Δ = –2.4 ppm.

Chiral SFC Analysis: CHIRALPAK IG (CO_2:MeOH, 85:15, 2.5 mL min^{-1}, 40 °C, 220 nm) indicated 90% ee, t_R= 6.52 (major), 11.79 (minor) minutes.

$[\alpha]_D^{25}$= + 325° (c. 0.18, $CHCl_3$).

Characterization of Products in Scheme 4

General Procedure M: Palladium/(*R*)–sSPhos-Catalyzed Dearomatization of Oxygen-linked Phenols

A vial containing a magnetic stirrer bar was charged with phenol starting material (0.100 mmol), crushed KOH (16.8 mg, 0.300 mmol, 3.00 equiv.), (*R*)–sSPhos (3.8 mg, 0.0075 mmol, 7.5 mol%), and Pd_2dba_3 (2.3 mg, 0.0025 mmol, 2.5 mol%). The vial was sealed, and evacuated and backfilled with N_2 (3×). PhMe (0.50 mL) and H_2O (50 µL) were added under nitrogen. The reaction was stirred at 110 °C and 900 rpm for 48 h. Upon completion, the vial was opened, and the solvent was removed under a stream of air. Sat. NH_4Cl (aq.) (0.5 ml) was added, and the aqueous layer was extracted with chloroform (3 × 0.5 ml). The combined organic layers were dried over $MgSO_4$ and purified by flash column chromatography or preparatory TLC.

(*R*)-2-Methyl-3'*H*-spiro[cyclohexane-1,1'-isobenzofuran]-2,5-dien-4-one (9a)

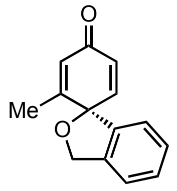

Prepared according to General Procedure M with 4-((2-bromobenzyl)oxy)-3-methylphenol **(8a)** (29.3 mg, 0.100 mmol) as the starting material. Purification by flash column chromatography (10% EtOAc in petroleum ether) and preparatory TLC (CH_2Cl_2) afforded the title compound as a colorless solid (4.7 mg, 0.022 mmol, 22% yield, 83% ee).

^{1}H NMR (400 MHz, $CDCl_3$) δ 7.41 – 7.24 (3H, m), 6.93 (1H, d, *J*=7.7 Hz), 6.82 (1H, dd, *J*=10.0, 2.5 Hz), 6.19 – 6.13 (1H, m), 6.12 – 6.09 (1H, m), 5.43 – 5.29 (2H, m), 1.79 – 1.70 (3H, m).

^{13}C NMR (101 MHz, $CDCl_3$) δ 186.3, 160.5, 149.2, 139.8, 138.7, 129.1, 128.5, 126.4, 126.3, 121.9, 121.8, 86.4, 74.4, 18.4.

HRMS m/z: $[M+H]^+$ calculated for $[C_{14}H_{13}O_2]^+$ 213.0910, found 213.0911. Δ = +0.5 ppm.

Chiral SFC Analysis: CHIRALPAK IK (CO_2:MeOH, 90:10, 2.5 mL min^{-1}, 40 °C, 250 nm) indicated 83% ee, t_R = 3.74 (major), 4.12 (minor) minutes.

$[\alpha]_D^{25}$ = +33.6° (c 0.24, $CHCl_3$).

(*S*)-2-Methoxy-3'*H*-spiro[cyclohexane-1,1'-isobenzofuran]-2,5-dien-4-one (9b)

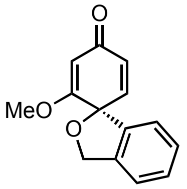

Prepared according to General Procedure M with 4-((2-bromobenzyl)oxy)-3-methoxyphenol **(8b)** (30.9 mg, 0.100 mmol) as the starting material, and NaOH (12.0 mg, 0.300 mmol, 3.00 equiv.) as the base (in place of KOH). Purification by preparatory TLC (40% EtOAc in petroleum ether) afforded the title compound as a brown solid (7.3 mg, 0.032 mmol, 32% yield, 82% ee).

^{1}H NMR (500 MHz, $CDCl_3$) δ 7.37 (1H, t, *J*=7.6 Hz), 7.31 (1H, d, *J*=7.6 Hz), 7.29 – 7.24 (1H, m), 6.99 (1H, d, *J*=7.6 Hz), 6.60 (1H, d, *J*=9.9 Hz), 6.17 (1H, d, *J*=9.9 Hz), 5.52 (1H, s), 5.40 (1H, d, *J*=11.8 Hz), 5.31 (1H, d, *J*=11.8 Hz), 3.63 (3H, s).

^{13}C NMR (126 MHz, $CDCl_3$) δ 187.6, 175.3, 145.0, 140.5, 138.6, 129.1, 128.1, 126.9, 121.7, 121.7, 101.1, 84.5, 74.9, 56.1.

HRMS m/z: $[M+H]^+$ calculated for $[C_{14}H_{12}O_3]^+$ 229.0859, found 229.0857. Δ = –0.9 ppm.

Chiral SFC Analysis: CHIRALPAK IG (CO_2:MeOH, 95:05, 2.5 mL min^{-1}, 40 °C, 220 nm) indicated 82% ee, t_R = 10.86 (minor), 13.25 (major) minutes.

$[\alpha]_D^{25}$ = +19.3° (c 0.49, $CHCl_3$).

(*R*)-4'-Chloro-2,3-dimethyl-3'*H*-spiro[cyclohexane-1,1'-isobenzofuran]-2,5-dien-4-one (9c)

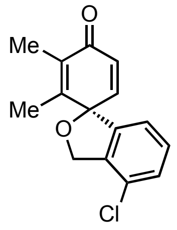

Prepared according to General Procedure M with 4-((2-bromo-6-chlorobenzyl)oxy)-2,3-dimethylphenol (**8c**) (34.2 mg, 0.100 mmol) as the starting material. Due to difficulty separating layers during extraction, an alternative work-up procedure was adopted. Upon completion of the reaction, the reaction mixture was filtered through celite, washing with EtOAc. The filtrate was concentrated under a stream of air. Purification by preparatory TLC (10% EtOAc in PhMe) afforded the title compound as a yellow solid (12.7 mg, 0.049 mmol, 49% yield, 93% ee).

^{1}H NMR (400 MHz, $CDCl_3$) δ 7.32 – 7.20 (2H, m), 6.82 – 6.73 (2H, m), 6.17 (1H, dd, *J*=9.9, 2.4 Hz), 5.36 (2H, app s), 1.92 (3H, s), 1.72 (3H, s).

^{13}C NMR (101 MHz, $CDCl_3$) δ 185.6, 152.5, 147.4, 141.6, 138.4, 131.8, 130.2, 129.0, 128.5, 126.2, 120.1, 87.6, 73.7, 15.3, 11.1.

HRMS m/z: $[M+H]^+$ calculated for $[C_{15}H_{14}ClO_2]^+$ 261.0677, found 261.0670. Δ = –2.7 ppm.

Chiral SFC Analysis: CHIRALPAK IK (CO_2:MeOH, 90:10, 2.5 mL min^{-1}, 40 °C, 220 nm) indicated 93% ee, t_R = 3.69 (major), 4.61 (minor) minutes.

[α]$_D$^{25} = +41.0° (c 0.85, $CHCl_3$).

References

(1) Pearce-Higgins, R.; Hogenhout, L. N.; Docherty, P. J.; Whalley, D. M.; Chuentragool, P.; Lee, N.; Lam, N. Y. S.; McGuire, T. M.; Valette, D.; Phipps, R. J. *J. Am. Chem. Soc.* **2022**, *144*, 15026–15032.

(2) Qiao, Y.; Bai, S.; Wu, X.-F.; Yang, Y.; Meng, H.; Ming, J. *Org. Lett.* **2022**, *24*, 1556–1560.

(3) Xu, R.-Q.; Yang, P.; Zheng, C.; You, S.-L. *Chin. J. Chem.* **2020**, *38*, 683–689.

(4) Du, K.; Guo, P.; Chen, Y.; Cao, Z.; Wang, Z.; Tang, W. *Angew. Chem. Int. Ed.* **2015**, *54*, 3033–3037.

(5) Rousseaux, S.; García-Fortanet, J.; Del Aguila Sanchez, M. A.; Buchwald, S. L. *J. Am. Chem. Soc.* **2011**, *133*, 9282–9285.

(6) Ran, L.; Li, H.; Chao, G.; Kang, X.; Lei, T.; Li, W. *Synlett.* **2020**, *31*, 1809–1812.

(7) Gohil, K.; Zain, M.; Kazmi, H.; Williams, F. J. *Org. Biomol. Chem* **2022**, *20*, 2187.

(8) Cong, X.; Tang, H.; Zeng, X. *J. Am. Chem. Soc.* **2015**, *137*, 14367–14372.

(9) So, C. M.; Lau, C. P.; Chan, A. S. C.; Kwong, F. Y. *J. Org. Chem.* **2008**, *73*, 7731–7734.

(10) Jiang, J.; Yuan, D.; Ma, C.; Song, W.; Lin, Y.; Hu, L.; Zhang, Y. *Org. Lett.* **2021**, *23*, 279–284.

(11) Fridianto, K. T.; Wen, Y.-P.; Lo, L.-C.; Lam, Y. *RSC Adv.* **2023**, *13*, 17420–17426.

(12) Weinstein, A. B.; Stahl, S. S. *Angew. Chem. Int. Ed.* **2012**, *51*, 11505–11509.

(13) Okano, K.; Okuyama, K. I.; Fukuyama, T.; Tokuyama, H. *Synlett.* **2008**, *13*, 1977–1980.

(14) Hazimeh, H.; Mattalia, J. M.; Marchi-Delapierre, C.; Kanoufi, F.; Combellas, C.; Chanon, M. *Eur. J. Org. Chem.* **2009**, *17*, 2775–2787.

(15) Numasawa, K.; Hanaoka, K.; Saito, N.; Yamaguchi, Y.; Ikeno, T.; Echizen, H.; Yasunaga, M.; Komatsu, T.; Ueno, T.; Miura, M.; Nagano, T.; Urano, Y. *Angew. Chem. Int. Ed.* **2020,** *132*, 6071–6076.

(16) Joussot, J.; Schoenfelder, A.; Suffert, J.; Blond, G. *C. R. Chimie.* **2017**, *20*, 665–681.

(17) Xu, R.-Q.; Gu, Q.; Wu, W.-T.; Zhao, Z.-A.; You, S.-L. *J. Am. Chem. Soc.* **2014**, *136*, 15469–15472.

(18) Zhao, Y.-M.; Gu, P.-M.; Tu, Y.-Q.; Zhang, H.-J.; Zhang, Q.-W.; Fan, C.-A. *J. Org. Chem.* **2010**, 75, 5289–5295.

(19) Johnson, K. F.; Van Zeeland, R.; Stanley, L. M. *Org. Lett.* **2013**, *15*, 2798–2801.

(20) Ly, K. U.; Boussonnière, A.; Castanet, A.-S. *Eur. J. Org. Chem.* **2022**, *e202101409*.

(21) Suzuki, Y.; Nemoto, T.; Kakugawa, K.; Hamajima, A.; Hamada, Y. *Org. Lett.* **2012**, *14*, 2350–2353.

(22) Hellal, M.; Singh, S.; Cuny, G. D. *Tetrahedron.* **2012**, *68*, 1674–1681.

(23) Chernyak, N.; Buchwald, S. L. *J. Am. Chem. Soc.* **2012**, *134*, 12466–12469.

(24) Steeds, H. G.; Knowles, J. P.; Yu, W. L.; Richardson, J.; Cooper, K. G.; Booker-Milburn, K. I. *Chem. Eur. J.* **2020**, *26*, 14330–14334.

(25) Plamondon, S. J.; Warnica, J. M.; Kaldre, D.; Gleason, J. L. *Angew. Chem. Int. Ed.* **2020**, *132,* 259–264.

(*R*)-2-Methoxy-2',3'-dihydrospiro[cyclohexane-1, 1'-indene]-2,5-dien-4-one (2a)

Chiral SFC Analysis: CHIRALPAK IG (CO_2:MeOH, 90:10, 2.5 mL min^{-1}, 40 °C, 220 nm) indicated 92% ee, t_R = 6.41 (minor), 7.74 (major) minutes.

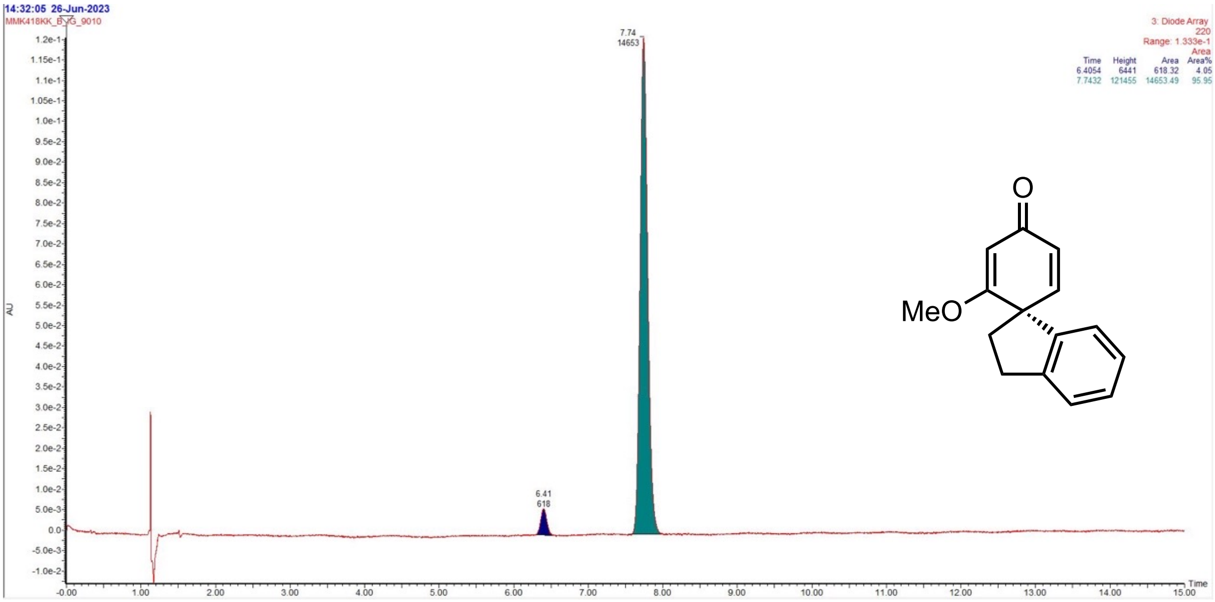

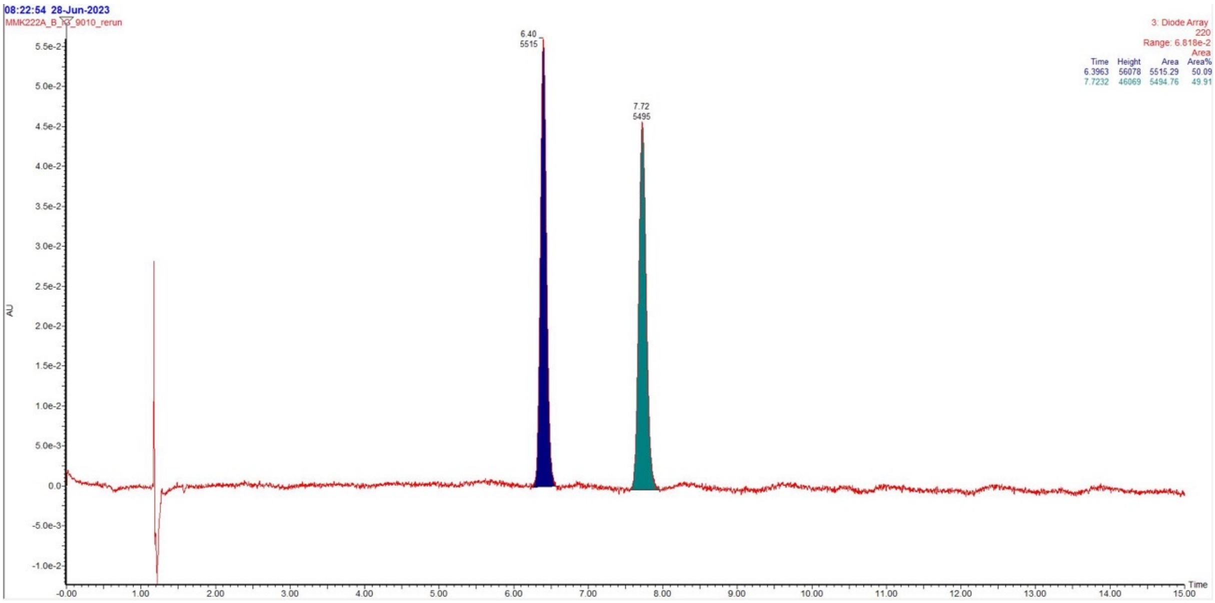

(R)-2-Methoxy-2',3'-dihydrospiro[cyclohexane-1, 1'-indene]-2,5-dien-4-one (2a)

Control reaction 5D: reaction with (R)-sSPhos-Np as the ligand

Chiral SFC Analysis: CHIRALPAK IG (CO_2:MeOH, 90:10, 2.5 mL min^{-1}, 40 °C, 220 nm) indicated -6% ee, t_R = 6.32 (major), 7.65 (minor) minutes.

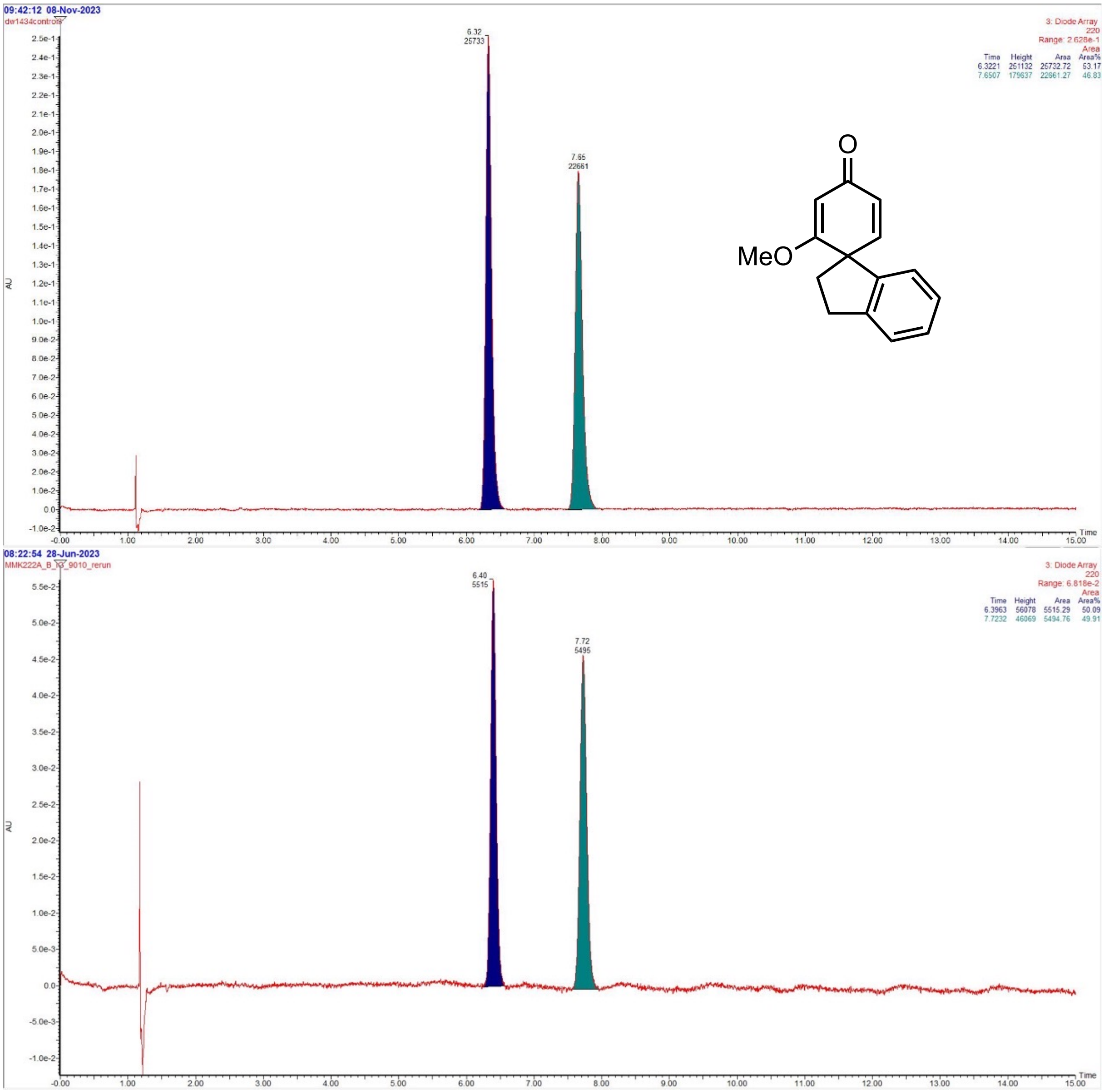

(*S*)-2-Methyl-2',3'-dihydrospiro[cyclohexane-1,1'-indene]-2,5-dien-4-one (2b)

Chiral SFC Analysis: CHIRALPAK IG (CO_2:MeOH, 90:10, 2.5 mL min^{-1}, 40 °C, 220 nm) indicated 86% ee, t_R = 7.49 (minor), 7.97 (major) minutes.

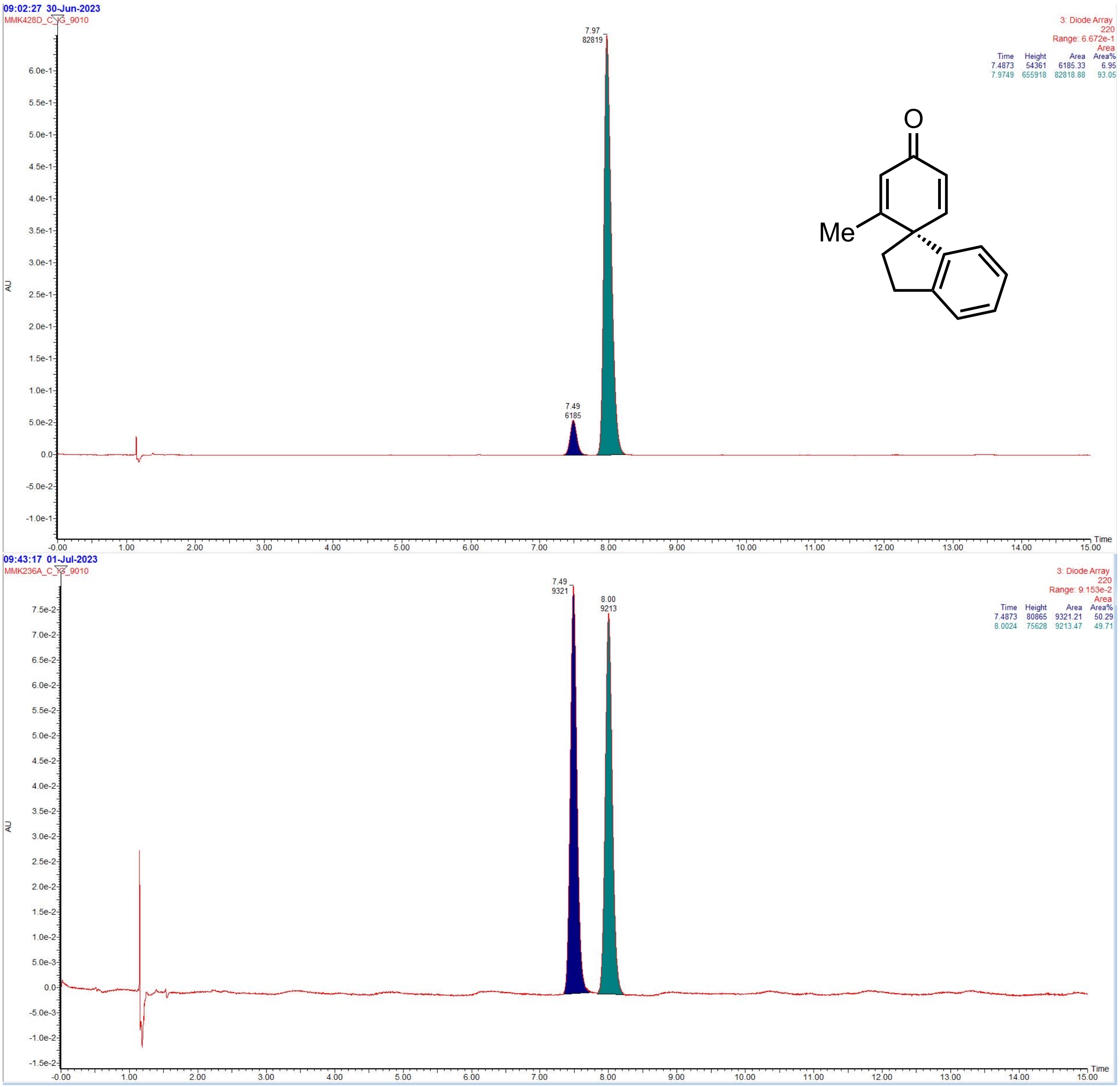

(*S*)-2-Phenyl-2',3'-dihydrospiro[cyclohexane-1,1'-indene]-2,5-dien-4-one (2c)

Chiral SFC Analysis: CHIRALPAK IG (CO_2:MeOH, 85:15, 2.5 mL min^{-1}, 40 °C, 220 nm) indicated 87% ee, t_R = 5.89 (minor), 8.44 (major) minutes.

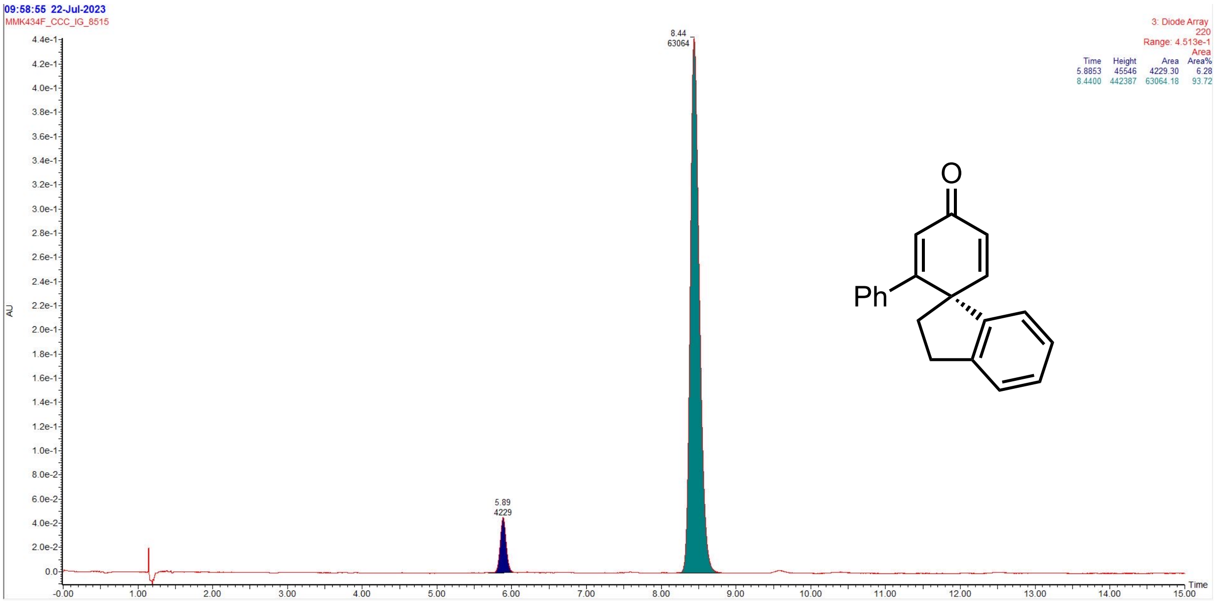

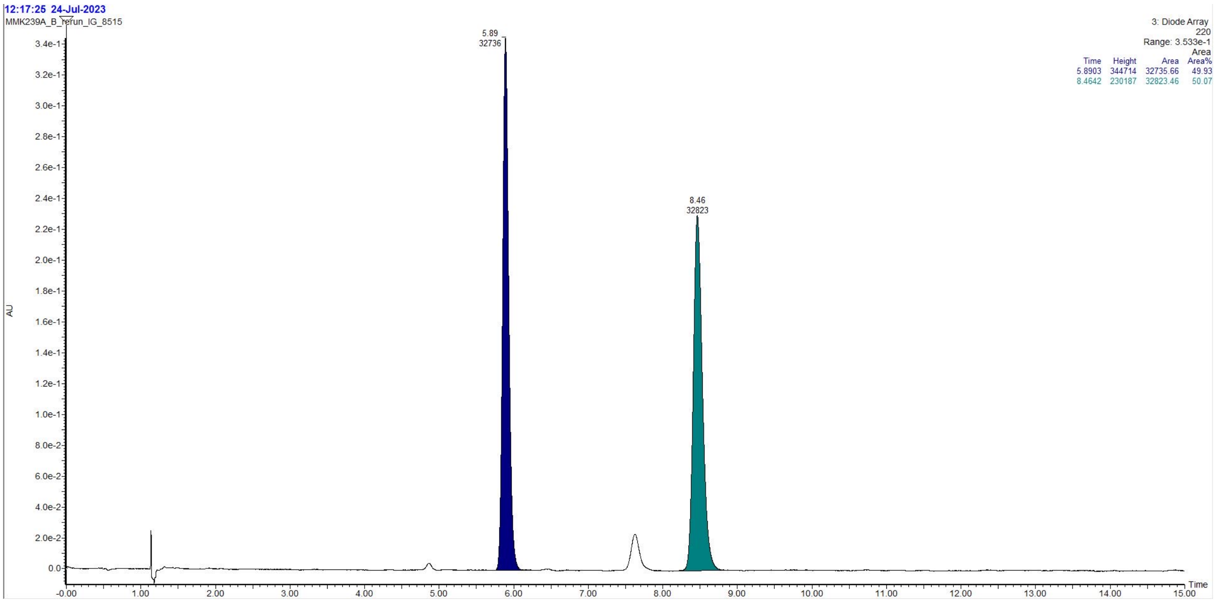

(*S*)-3-Phenyl-2',3'-dihydrospiro[cyclohexane-1,1'-indene]-2,5-dien-4-one (2d)

Chiral SFC Analysis: CHIRALPAK IE (CO_2:MeOH, 85:15, 2.5 mL min^{-1}, 40 °C, 220 nm) indicated 86% ee, t_R = 7.97 (minor), 8.60 (major) minutes.

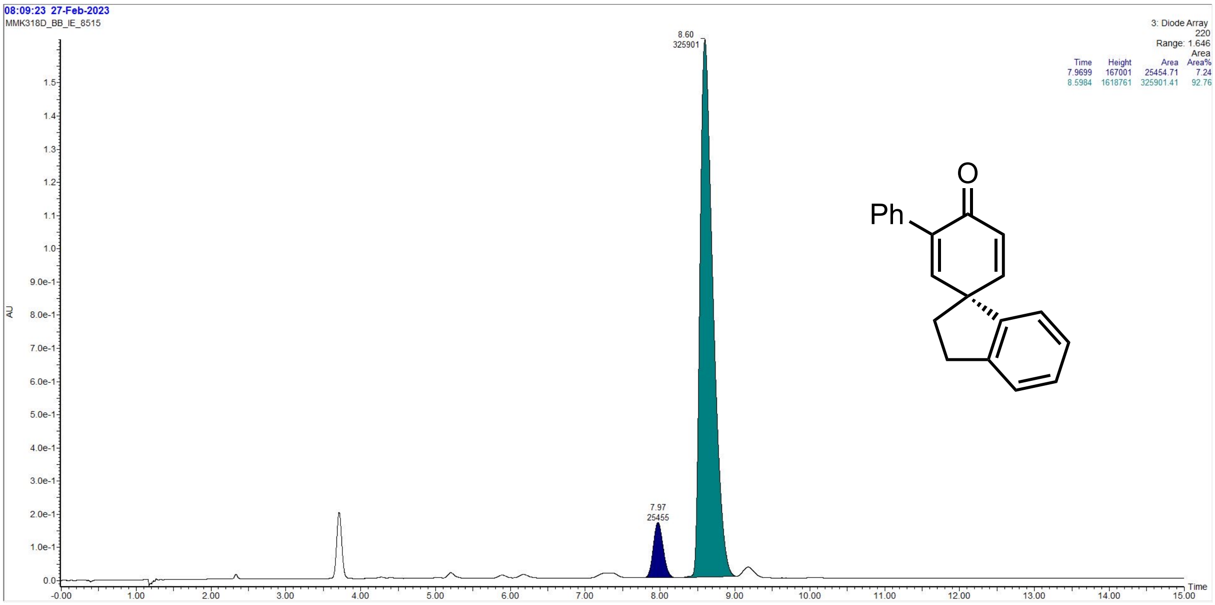

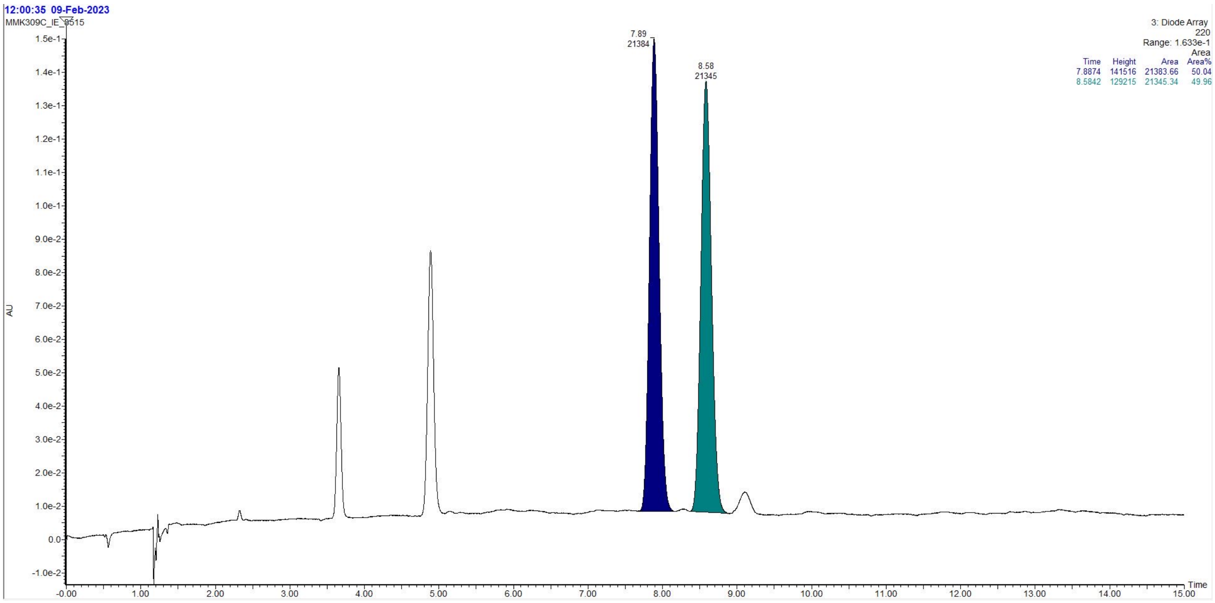

(*S*)-3-Methoxy-2',3'-dihydrospiro[cyclohexane-1,1'-indene]-2,5-dien-4-one (2e)

Chiral SFC Analysis: CHIRALPAK IG (CO_2:MeOH, 90:10, 2.5 mL min^{-1}, 40 °C, 220 nm) indicated 91% ee, t_R = 8.44 (minor), 9.41 (major) minutes.

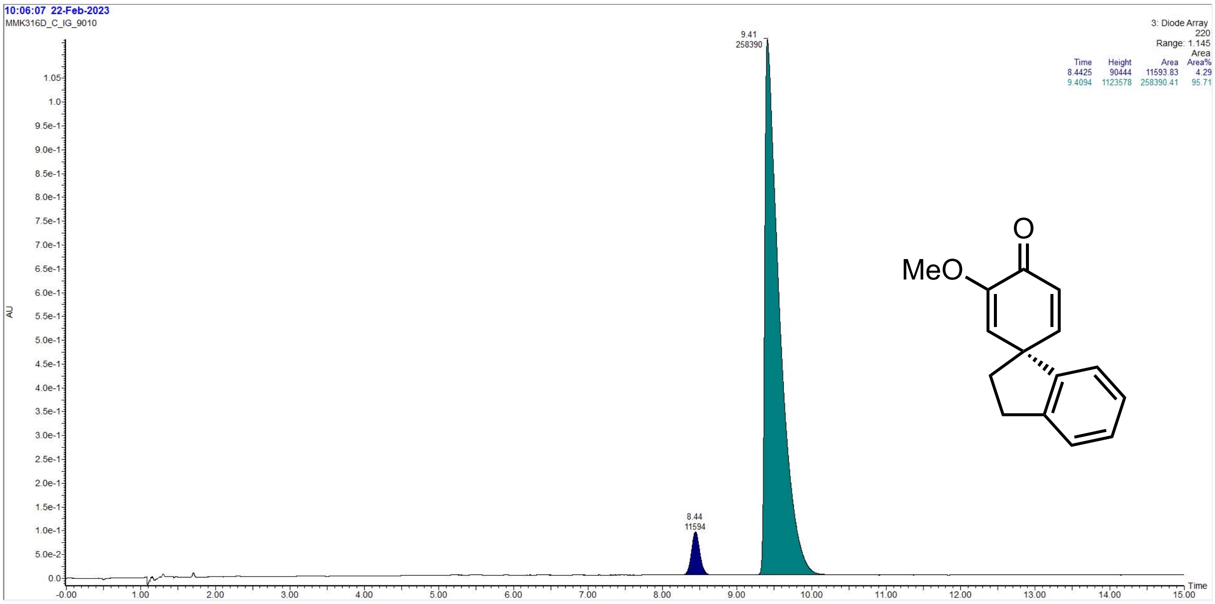

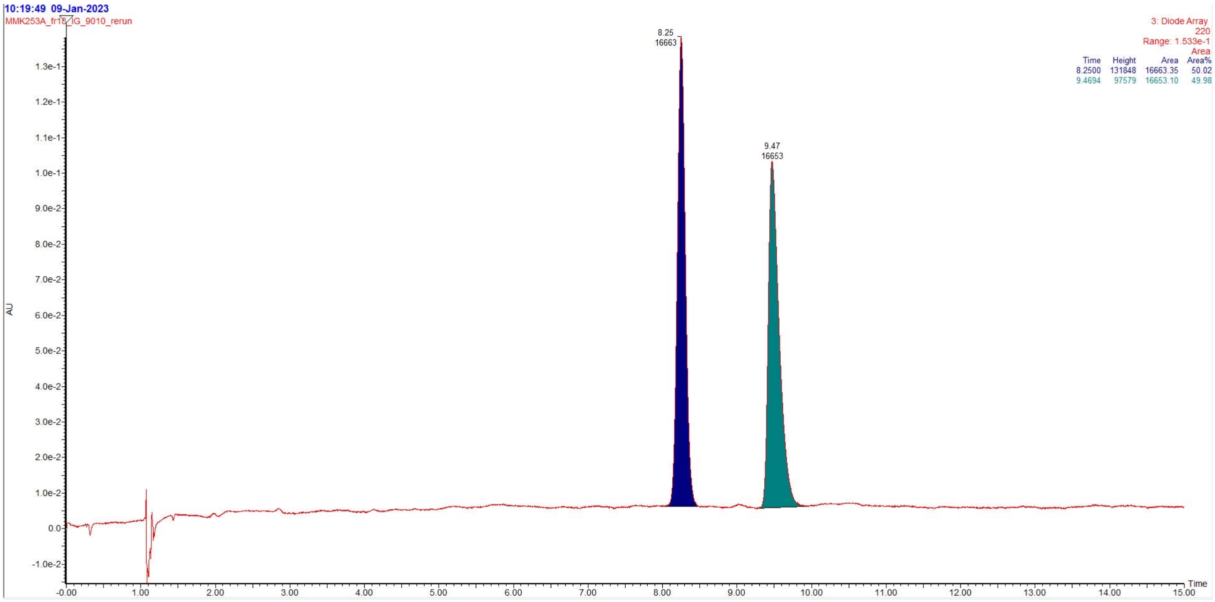

(*S*)-3-Methyl-2',3'-dihydrospiro[cyclohexane-1,1'-indene]-2,5-dien-4-one (2f)

Chiral HPLC Analysis: CHIRALPAK IG (Hexane:^{i}PrOH, 97:03, 1.25 mL min^{-1}, 40 °C, 250 nm) indicated 92% ee, t_R = 11.01 (minor), 12.53 (major) minutes.

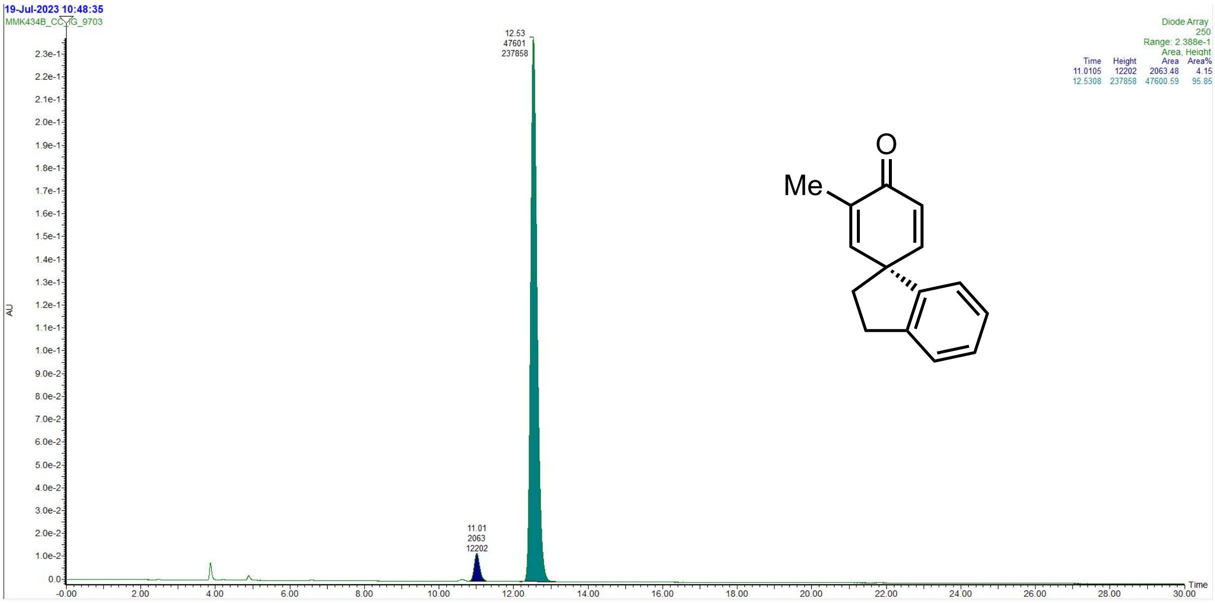

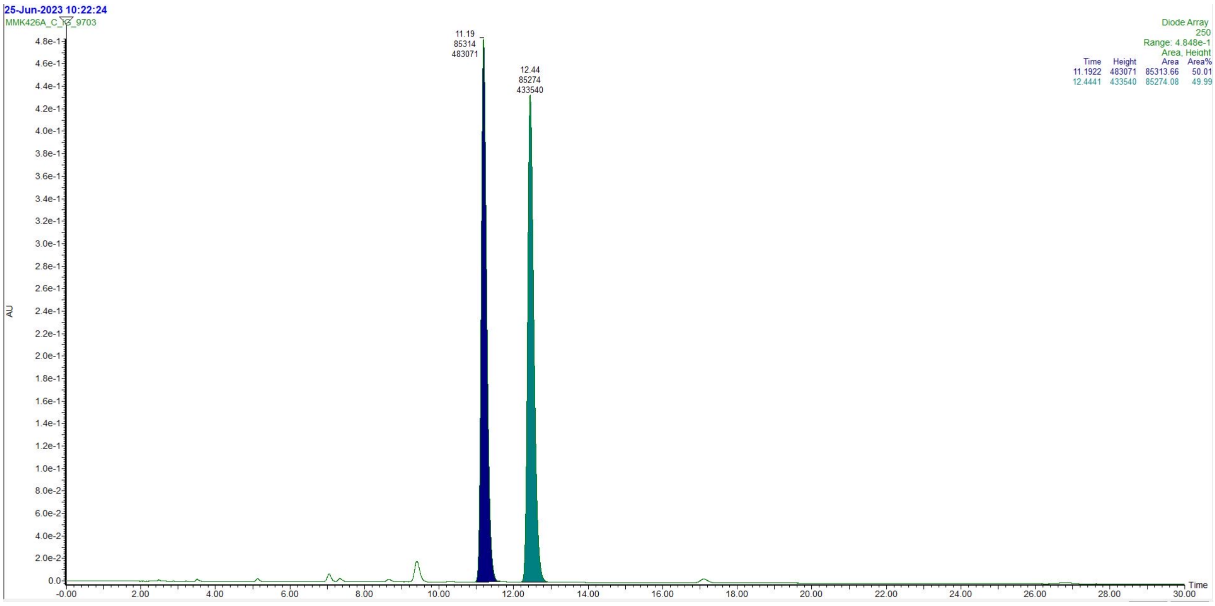

(*S*)-3-Fluoro-2',3'-dihydrospiro[cyclohexane-1,1'-indene]-2,5-dien-4-one (2g)

Chiral SFC Analysis: CHIRALPAK IA (CO_2:MeOH, 96:04, 2.5 mL min^{-1}, 40 °C, 240 nm) indicated 88% ee, t_R= 5.05 (minor), 5.39 (major) minutes.

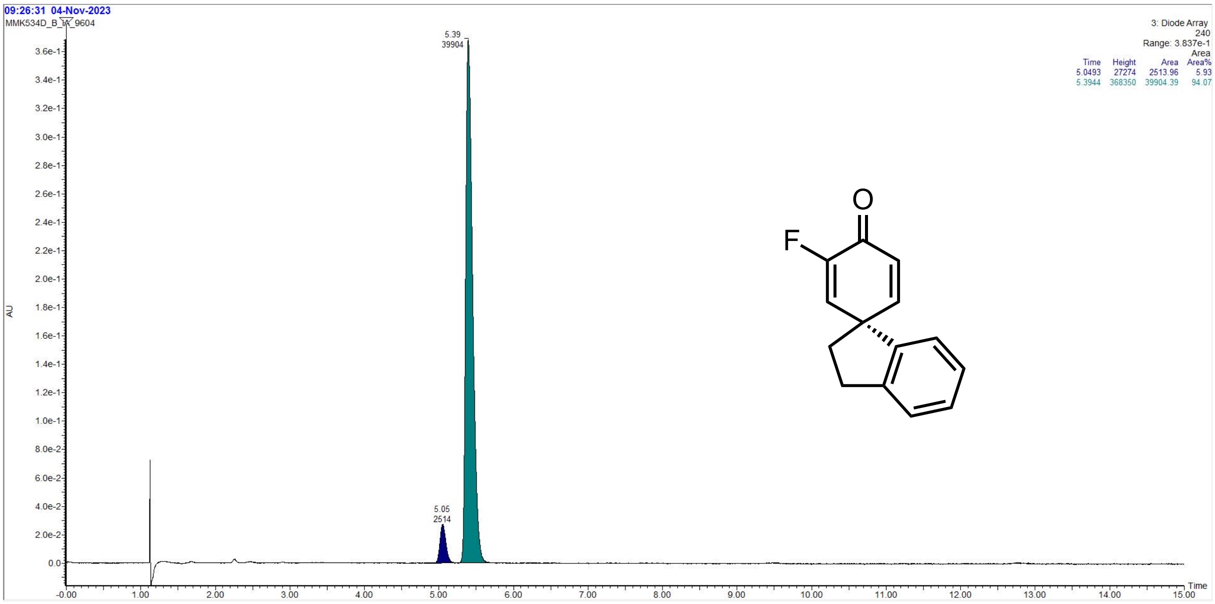

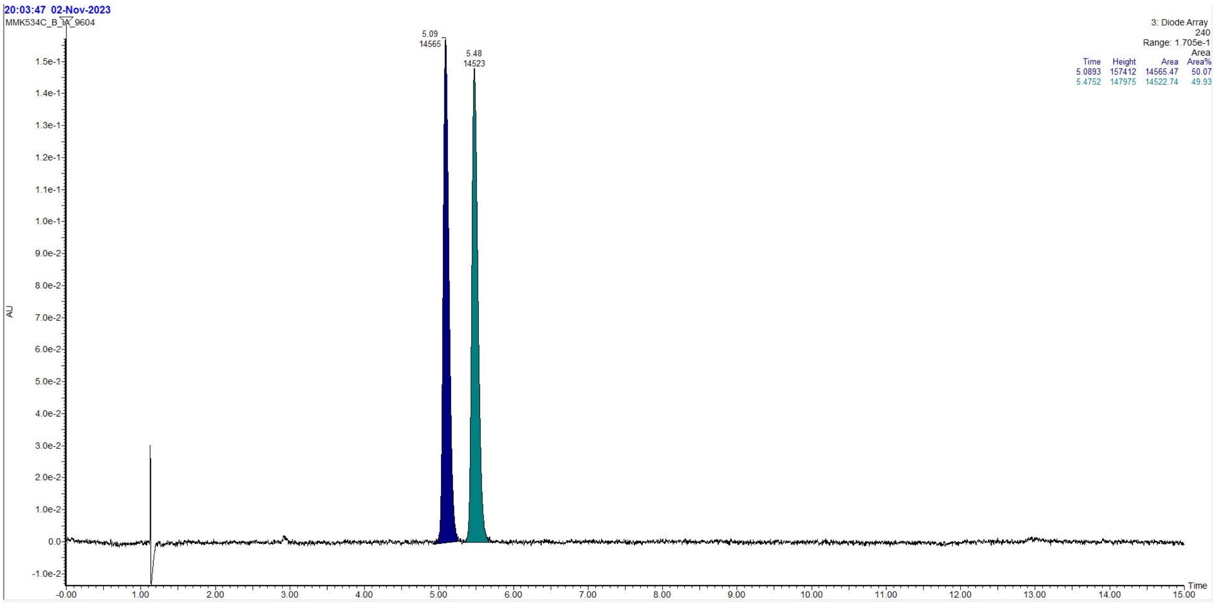

(*R*)-2,3-Dihydro-4'*H*-spiro[indene-1,1'-naphthalen]-4'-one (2h)

Chiral SFC Analysis: CHIRALPAK IG (CO_2:MeOH, 90:10, 2.5 mL min^{-1}, 40 °C, 220 nm) indicated 95% ee, t_R = 8.54 (minor), 10.48 (major) minutes.

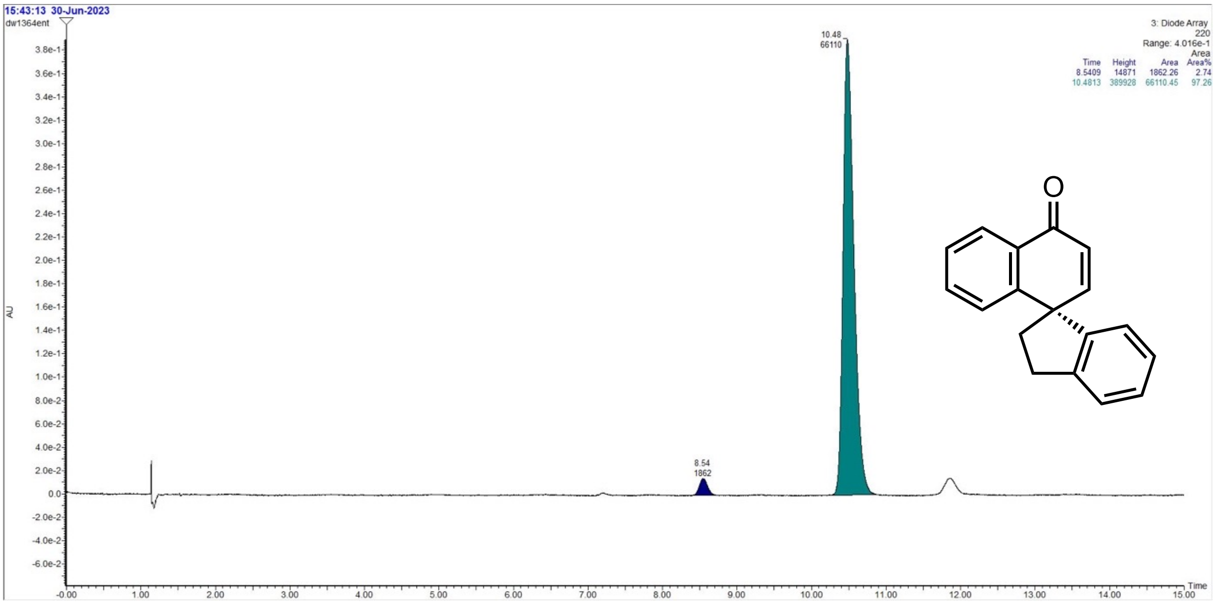

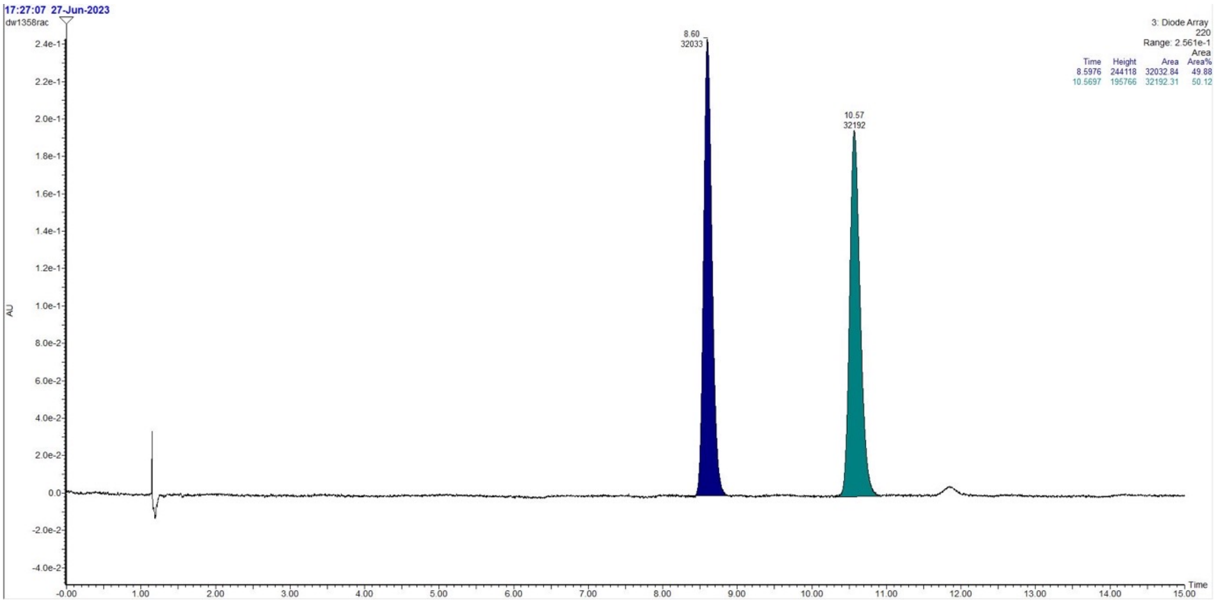

(*S*)-2,3-Dimethyl-2',3'-dihydrospiro[cyclohexane-1,1'-indene]-2,5-dien-4-one (2i)

Chiral SFC Analysis: CHIRALPAK IG (CO_2:MeOH, 90:10, 2.5 mL min^{-1}, 40 °C, 220 nm) indicated 88% ee, t_R = 5.73 (minor), 7.09 (major) minutes.

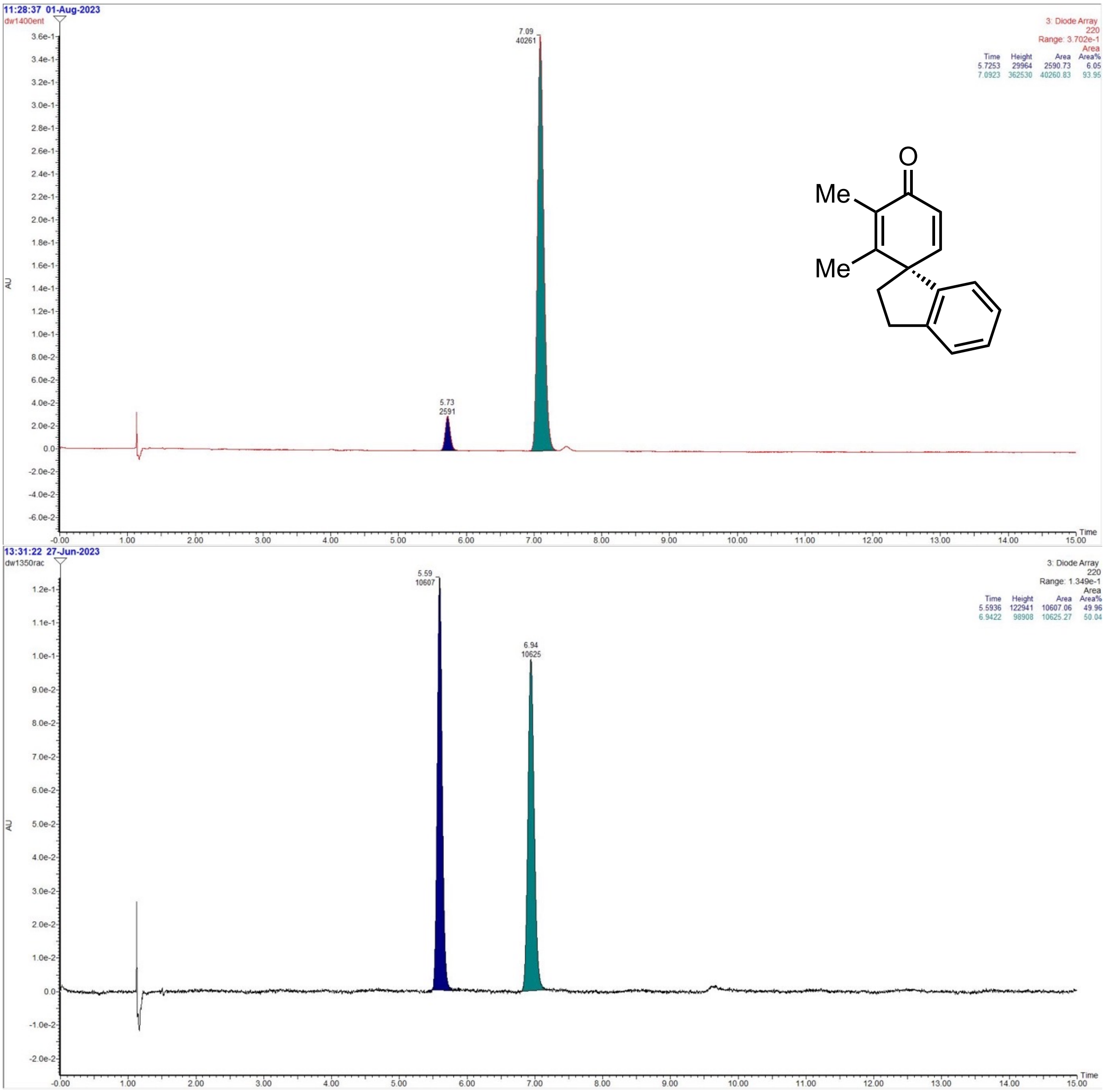

(*R*)-2-Methoxy-3',4'-dihydro-2'*H*-spiro[cyclohexane-1,1'-naphthalene]-2,5-dien-4-one (2j)

Chiral SFC Analysis: CHIRALPAK IG (CO_2:MeOH, 90:10, 2.5 mL min^{-1}, 40 °C, 220 nm) indicated 93% ee, t_R = 7.43 (minor), 9.69 (major) minutes.

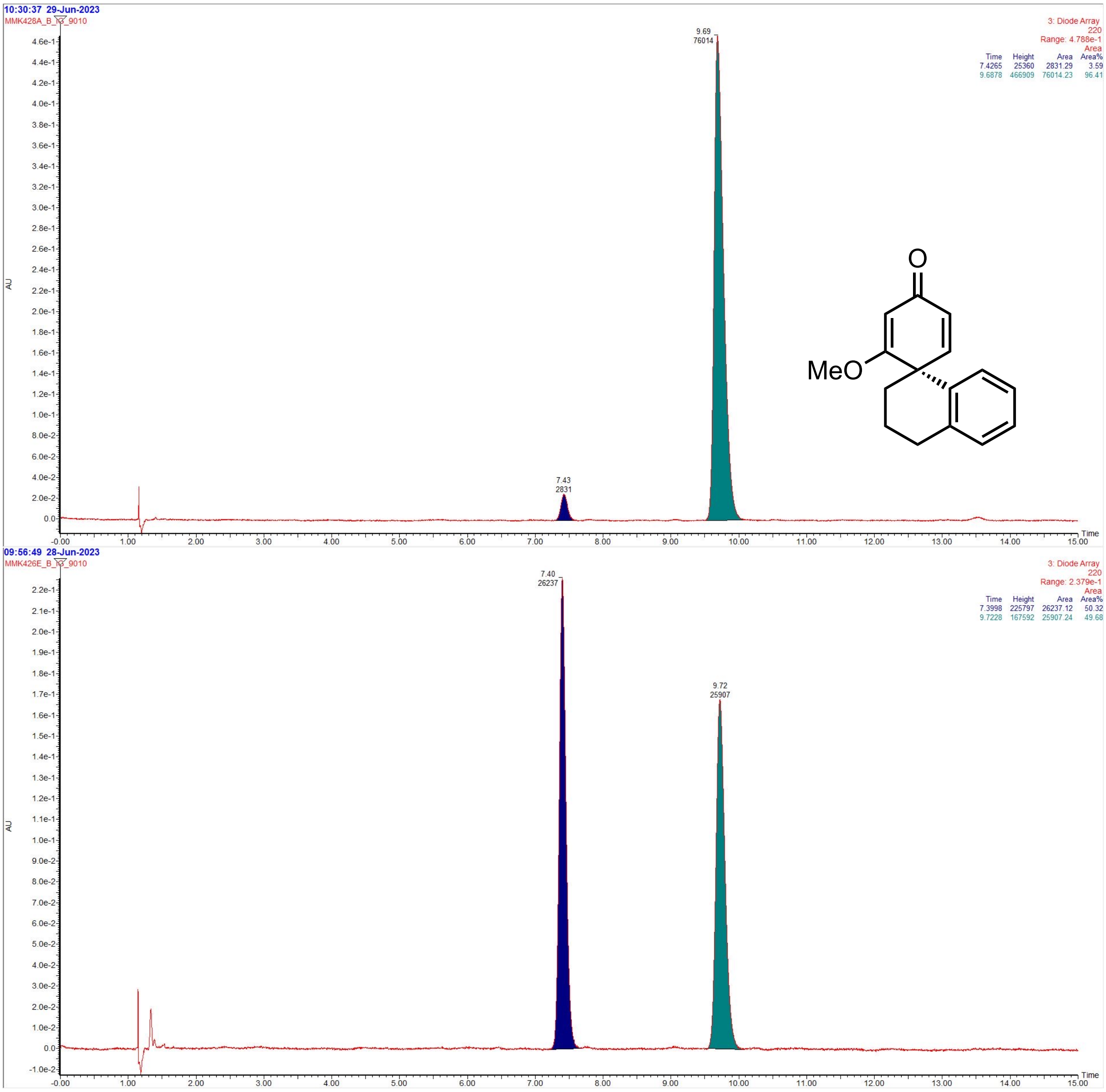

(*R*)-2'-Methoxy-6,7,8,9-tetrahydrospiro[benzo[7]annulene-5,1'-cyclohexane]-2',5'-dien-4'-one (2k)

Chiral SFC Analysis: CHIRALPAK IK (CO_2:MeOH, 90:10, 2.5 mL min^{-1}, 40 °C, 220 nm) indicated 88% ee, t_R = 8.01 (major), 8.45 (minor) minutes.

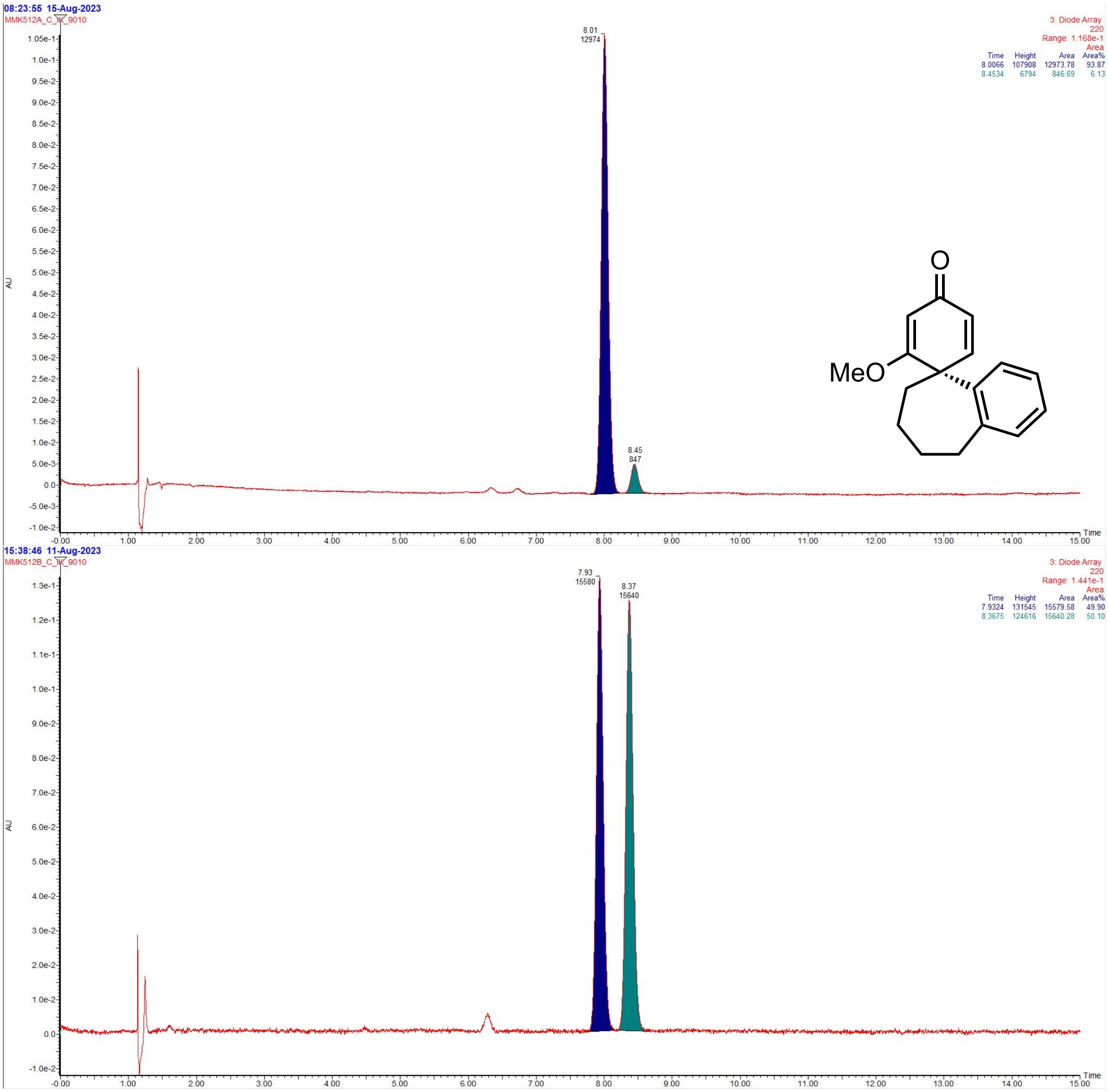

(*R*)-2-Methoxy-5'-(trifluoromethyl)-2',3'-dihydrospiro[cyclohexane-1,1'-indene]-2,5-dien-4-one (2l) (0.1 mmol scale)

Chiral SFC Analysis: CHIRALPAK IA (CO_2:MeOH, 95:05, 2.5 mL min^{-1}, 40 °C, 220 nm) indicated 88% ee, t_R = 5.13 (minor), 5.32 (major) minutes.

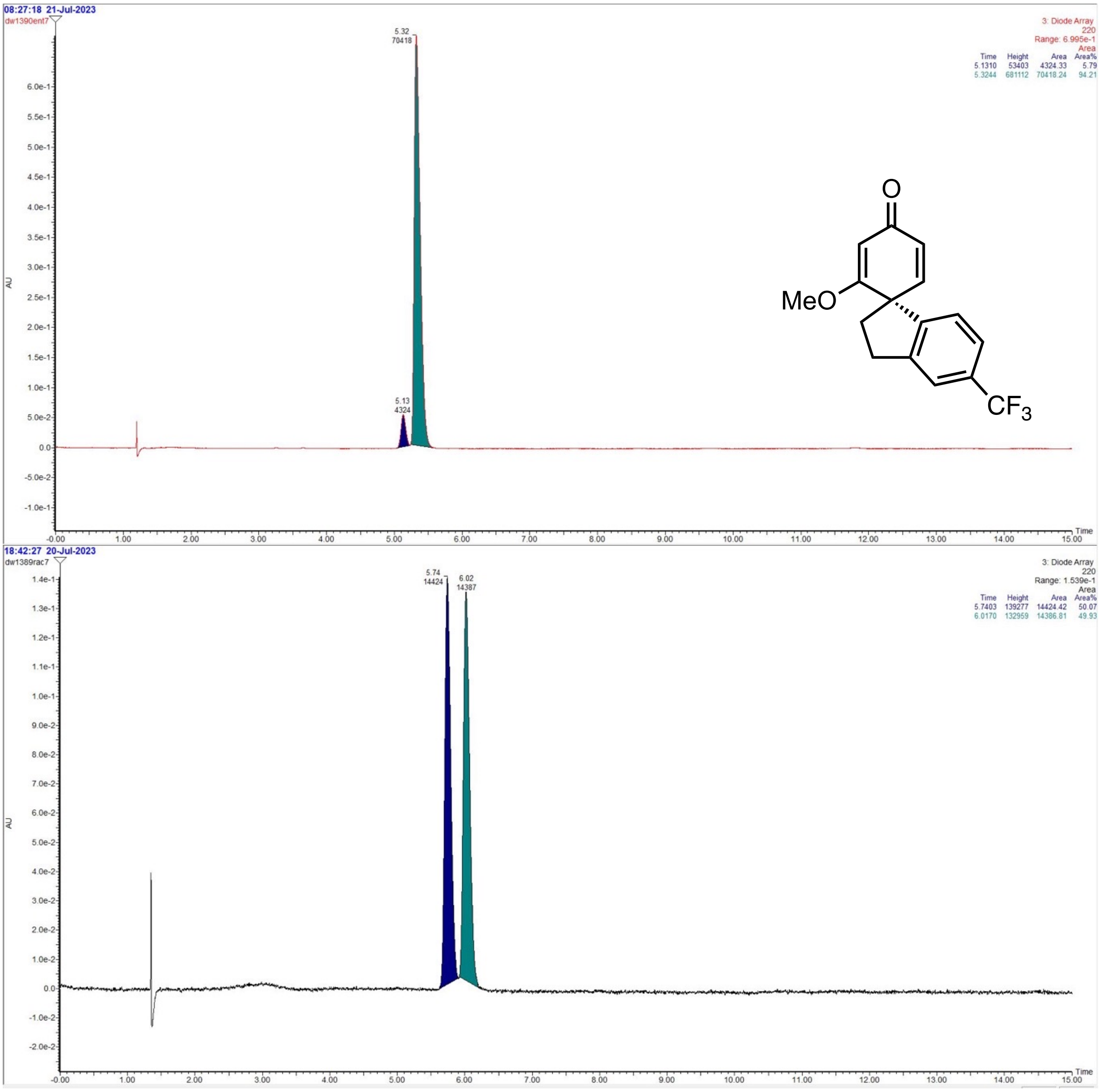

(*R*)-2-Methoxy-5'-(trifluoromethyl)-2',3'-dihydrospiro[cyclohexane-1,1'-indene]-2,5-dien-4-one (2l) (1.0 mmol scale)

Chiral SFC Analysis: CHIRALPAK IA (CO_2:MeOH, 95:05, 2.5 mL min^{-1}, 40 °C, 250 nm) indicated 90% ee, t_R = 4.89 (minor), 5.08 (major) minutes.

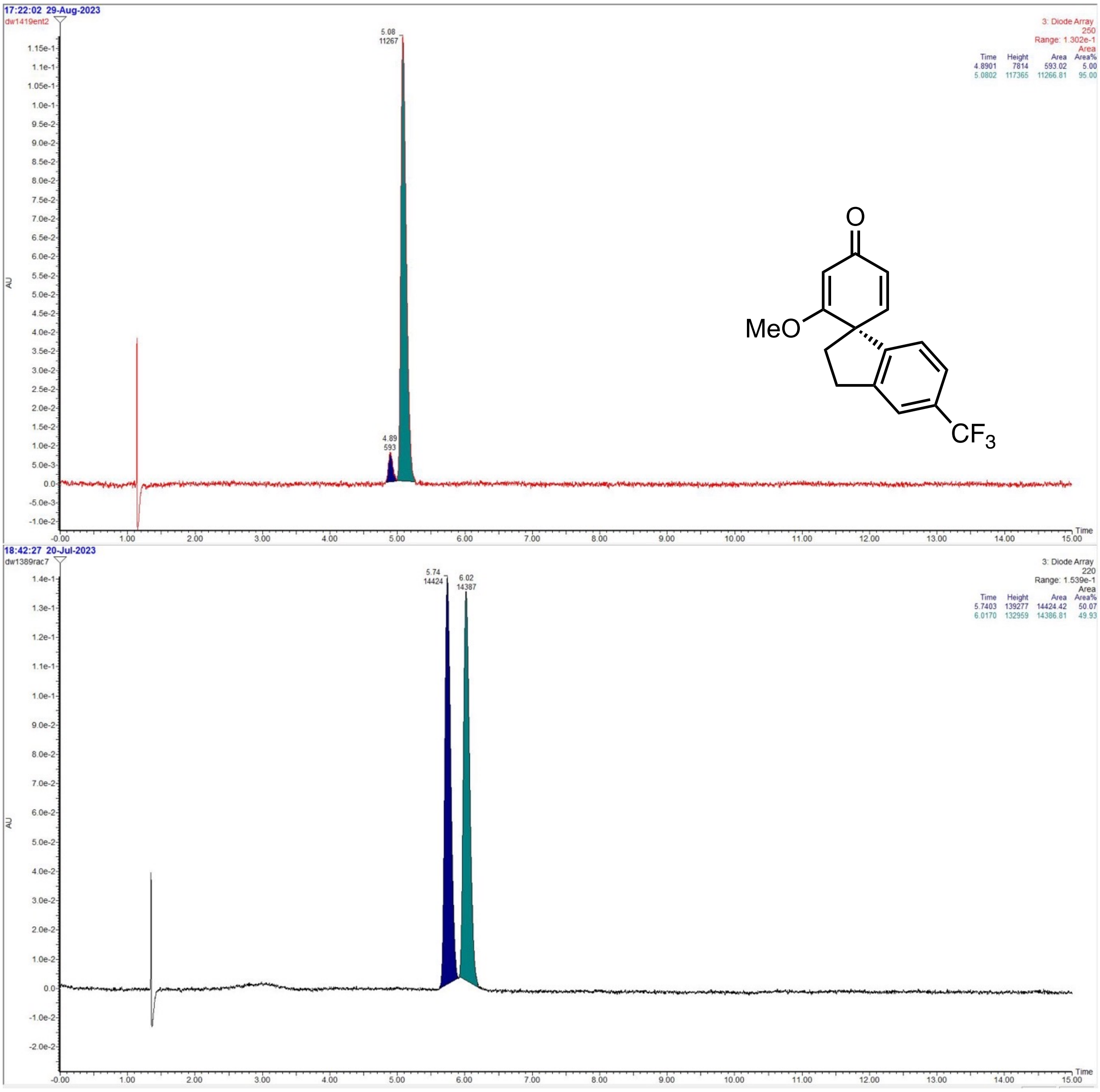

(*R*)-2,5',6'-Trimethoxy-2',3'-dihydrospiro[cyclohexane-1,1'-indene]-2,5-dien-4-one (2m)

Chiral SFC Analysis: CHIRALPAK IG (CO_2:MeOH, 90:10, 2.5 mL min^{-1}, 40 °C, 220 nm) indicated 90% ee, t_R = 6.20 (major), 12.95 (minor) minutes.

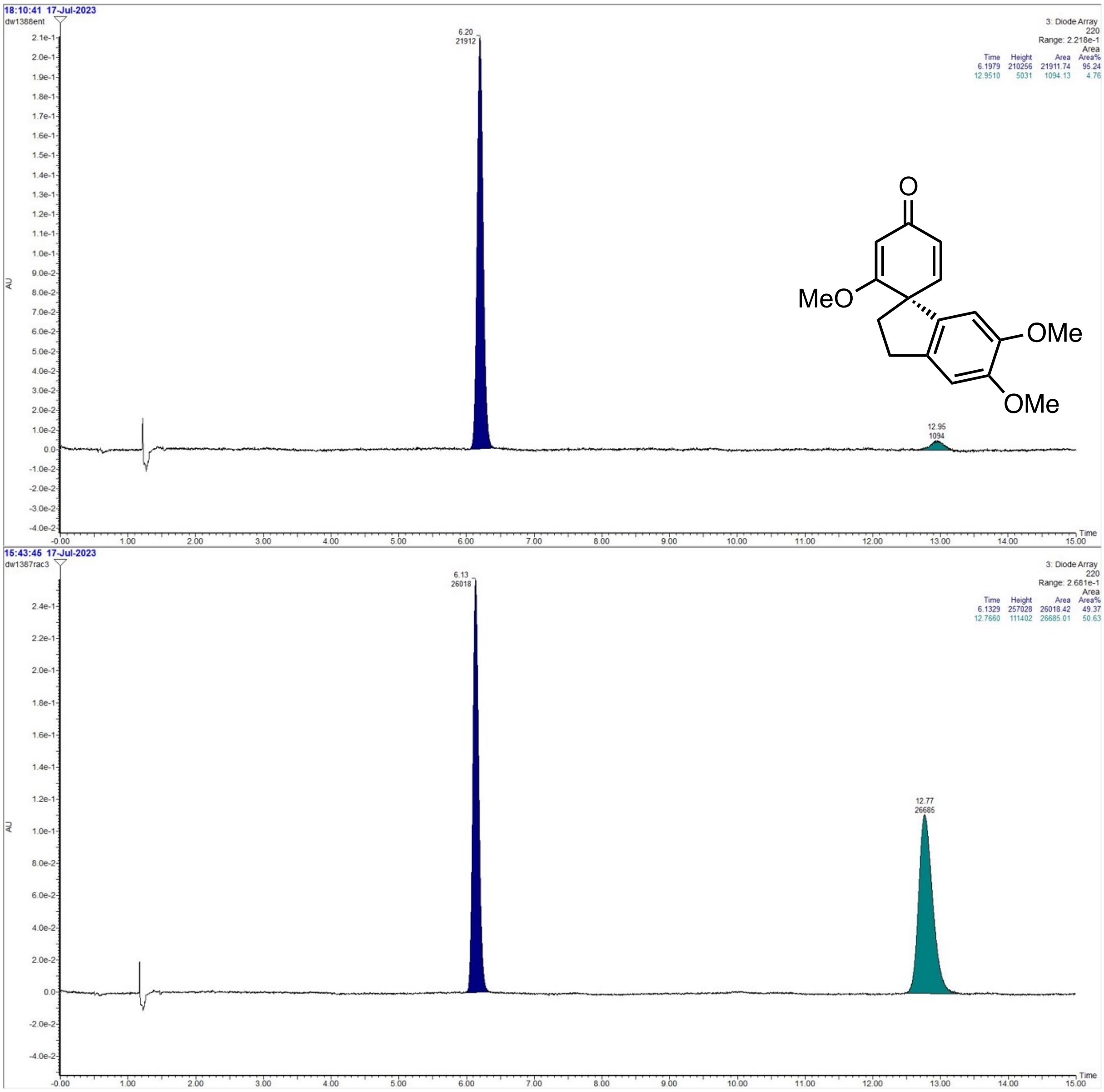

(*R*)-4'-Chloro-2-methoxy-2',3'-dihydrospiro[cyclohexane-1,1'-indene]-2,5-dien-4-one (2n)

Chiral SFC Analysis: CHIRALPAK IG (CO_2:MeOH, 90:10, 2.5 mL min^{-1}, 40 °C, 220 nm) indicated 96% ee, t_R = 7.15 (minor), 9.20 (major) minutes.

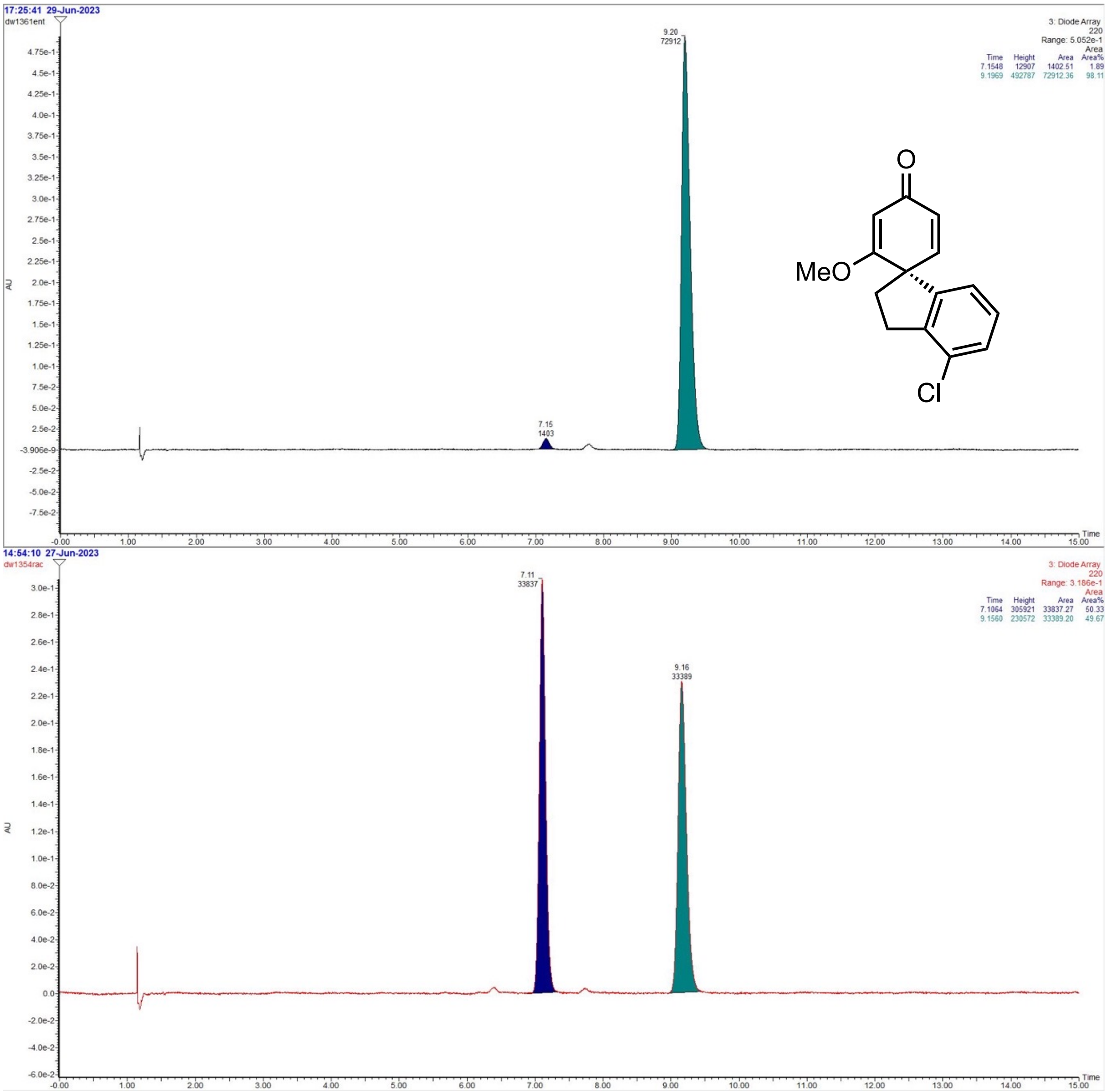

***tert*-Butyl (*R*)-(2-methoxy-4-oxo-2',3'-dihydrospiro[cyclohexane-1,1'-indene]-2,5-dien-6'-yl)carbamate (2o)**

Chiral SFC Analysis: CHIRALPAK IG (CO_2:MeOH, 85:15, 2.5 mL min^{-1}, 40 °C, 206 nm) indicated 86% ee, t_R = 8.60 (major), 9.86 (minor) minutes.

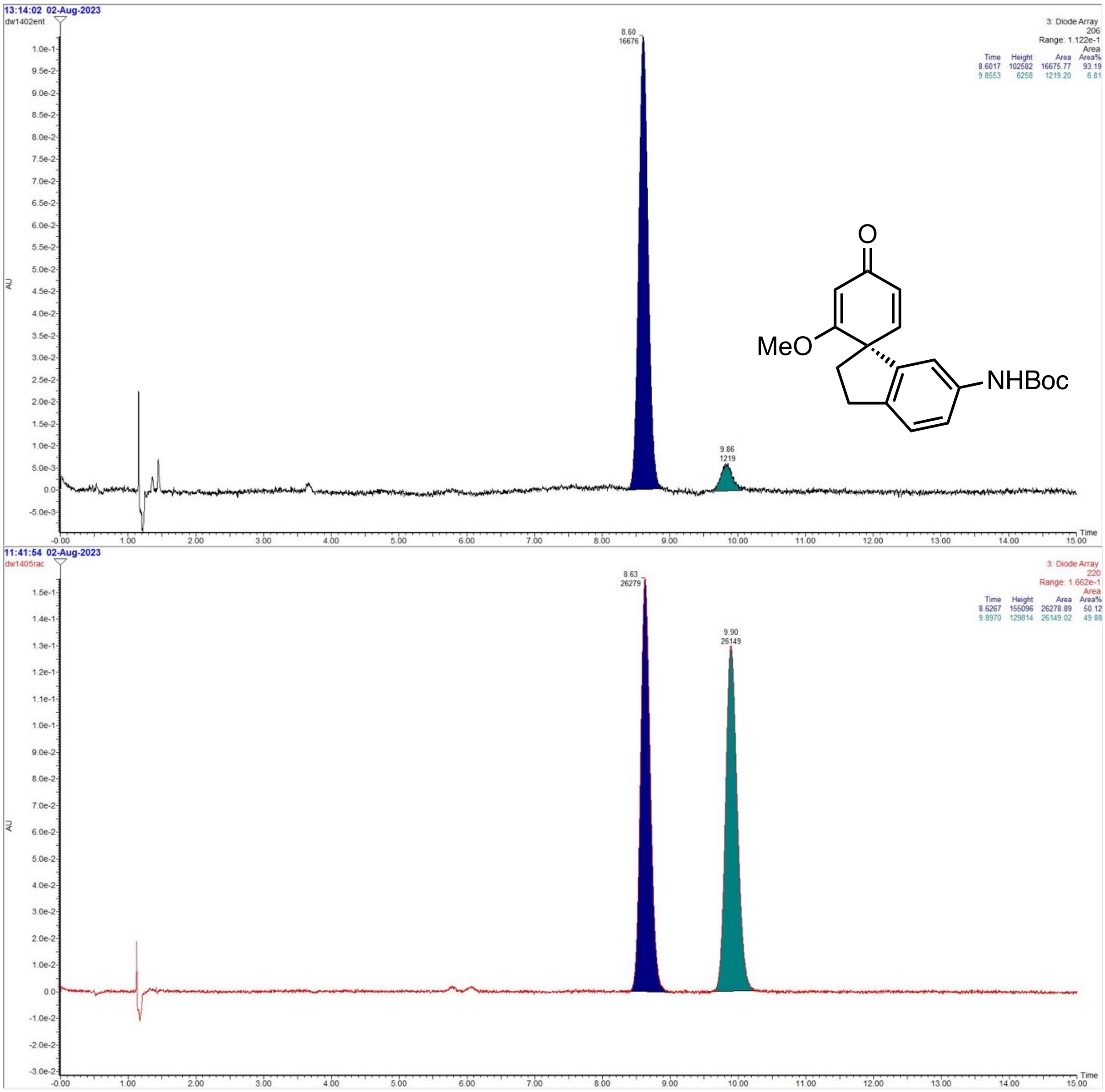

***tert*-Butyl (*R*)-2-methoxy-4-oxo-2',3'-dihydrospiro[cyclohexane-1,1'-indene]-2,5-diene-6'-carboxylate (2p)**

Chiral SFC Analysis: CHIRALPAK IG (CO_2:MeOH, 87:13, 2.5 mL min^{-1}, 40 °C, 230 nm) indicated 91% ee, t_R = 5.02 (major), 5.39 (minor) minutes.

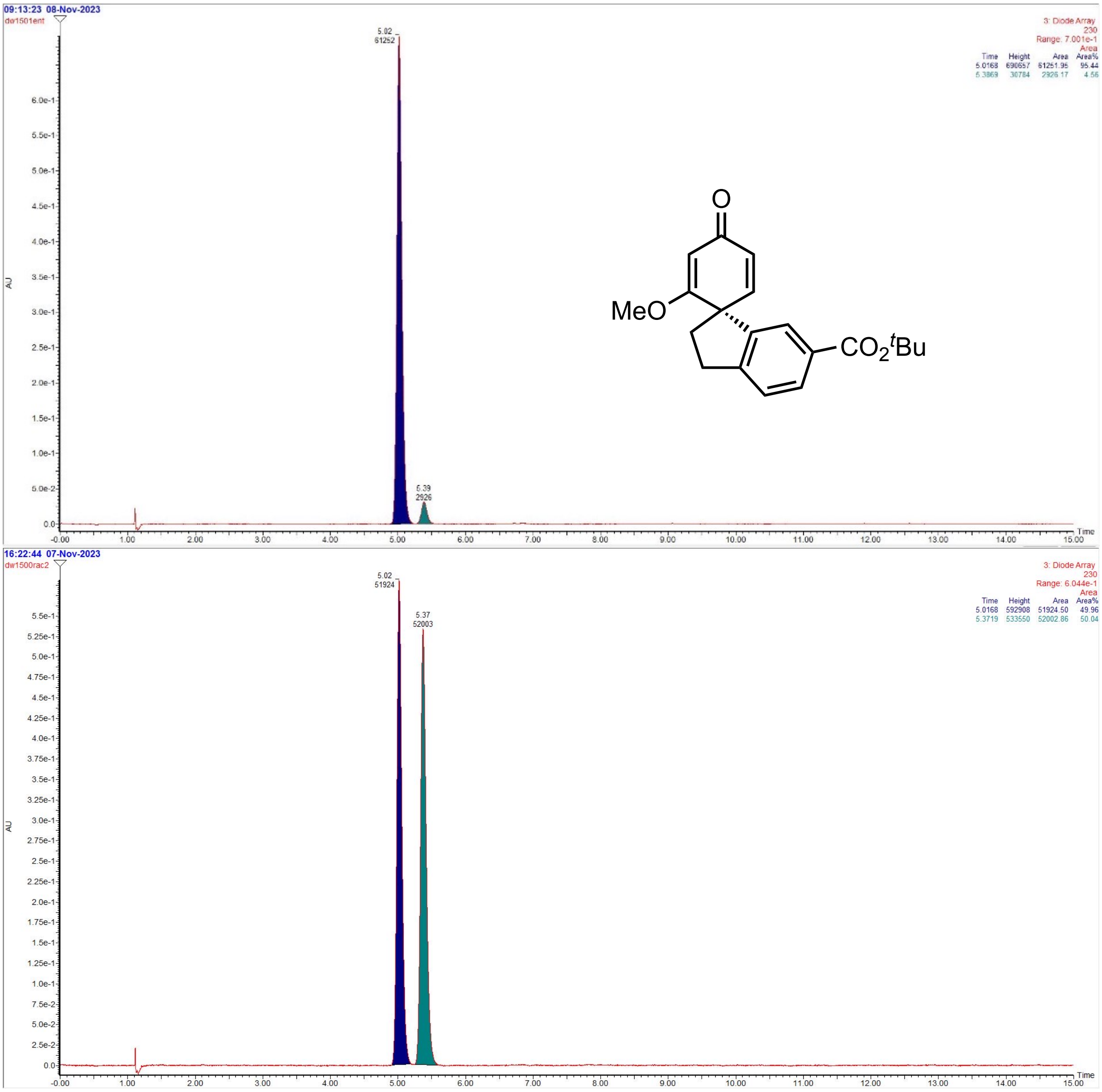

(*R*)-2-Methoxy-7'-methyl-2',3'-dihydrospiro[cyclohexane-1,1'-indene]-2,5-dien-4-one (2q)

Chiral SFC Analysis: CHIRALPAK IG (CO_2:MeOH, 90:10, 2.5 mL min^{-1}, 40 °C, 220 nm) indicated 74% ee, t_R = 6.62 (minor), 7.52 (major) minutes.

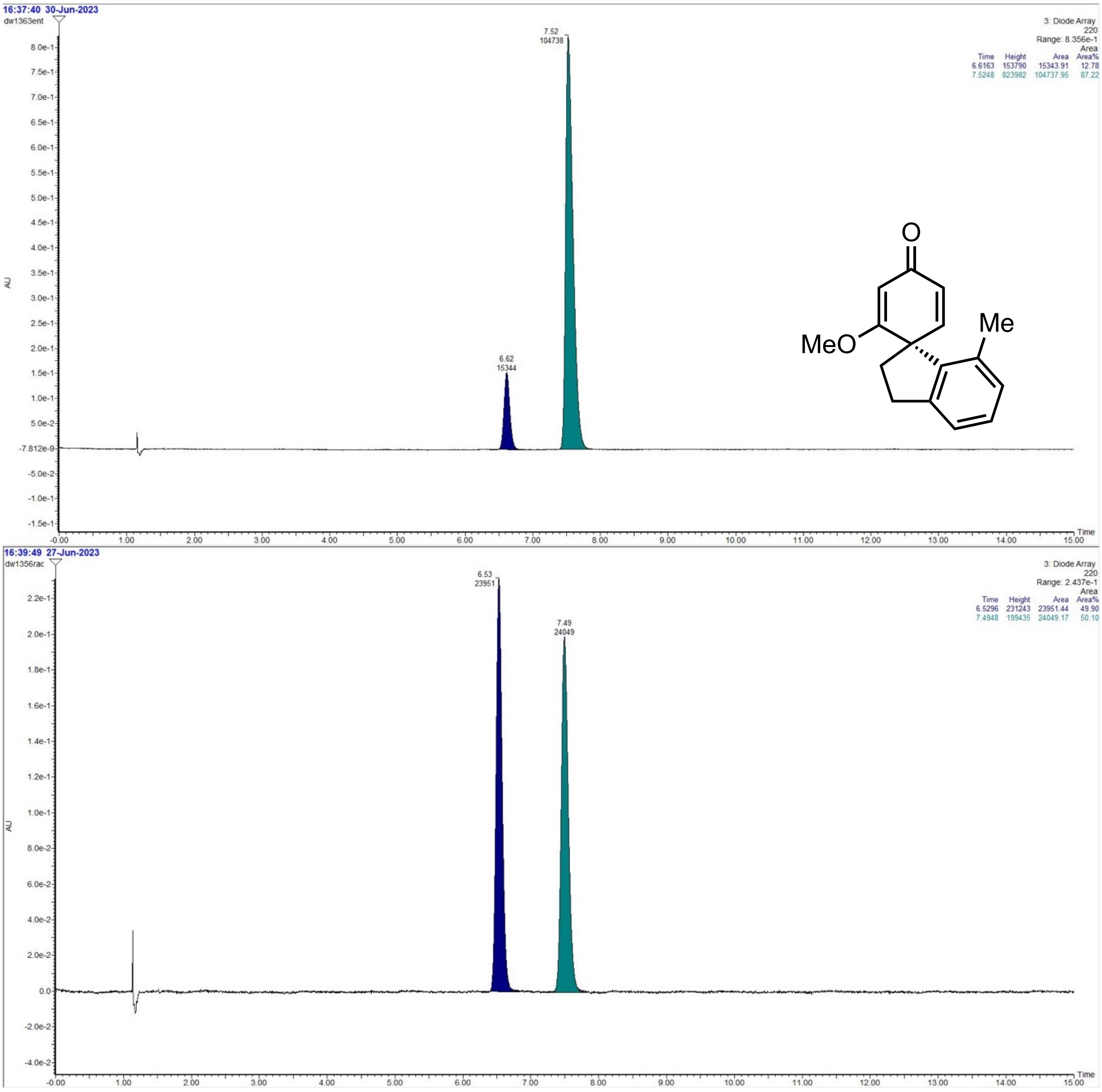

(*R*)-5'-Fluoro-2-methoxy-2',3'-dihydrospiro[cyclohexane-1,1'-indene]-2,5-dien-4-one (2r) (0.1 mmol scale)

Chiral SFC Analysis: CHIRALPAK IG (CO_2:MeOH, 90:10, 2.5 mL min^{-1}, 40 °C, 220 nm) indicated 91% ee, t_R = 5.58 (minor), 6.30 (major) minutes.

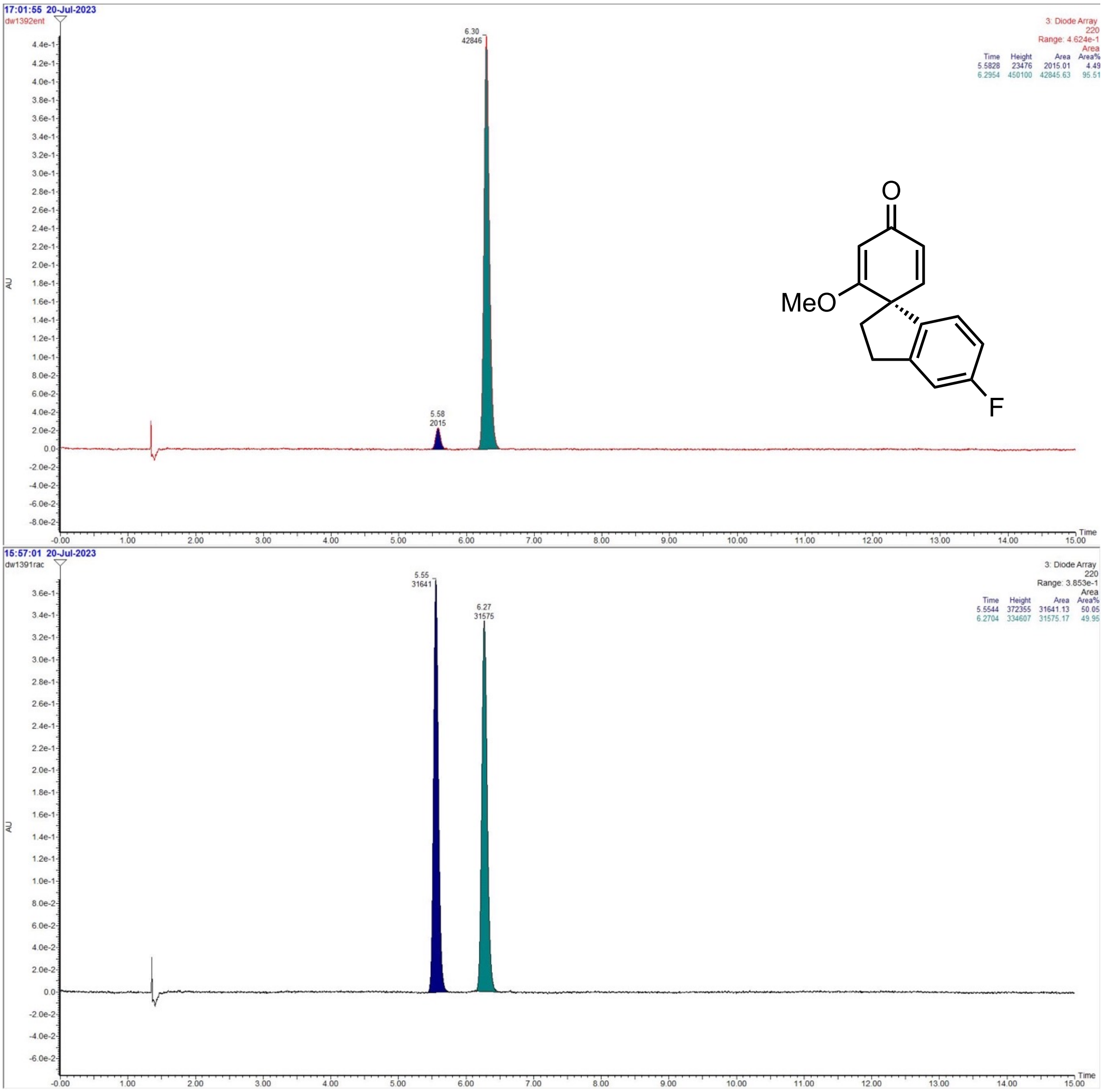

(*R*)-5'-Fluoro-2-methoxy-2',3'-dihydrospiro[cyclohexane-1,1'-indene]-2,5-dien-4-one (2r) (1.0 mmol scale)

Chiral SFC Analysis: CHIRALPAK IG (CO_2:MeOH, 90:10, 2.5 mL min^{-1}, 40 °C, 220 nm) indicated 95% ee, t_R = 5.20 (minor), 5.90 (major) minutes.

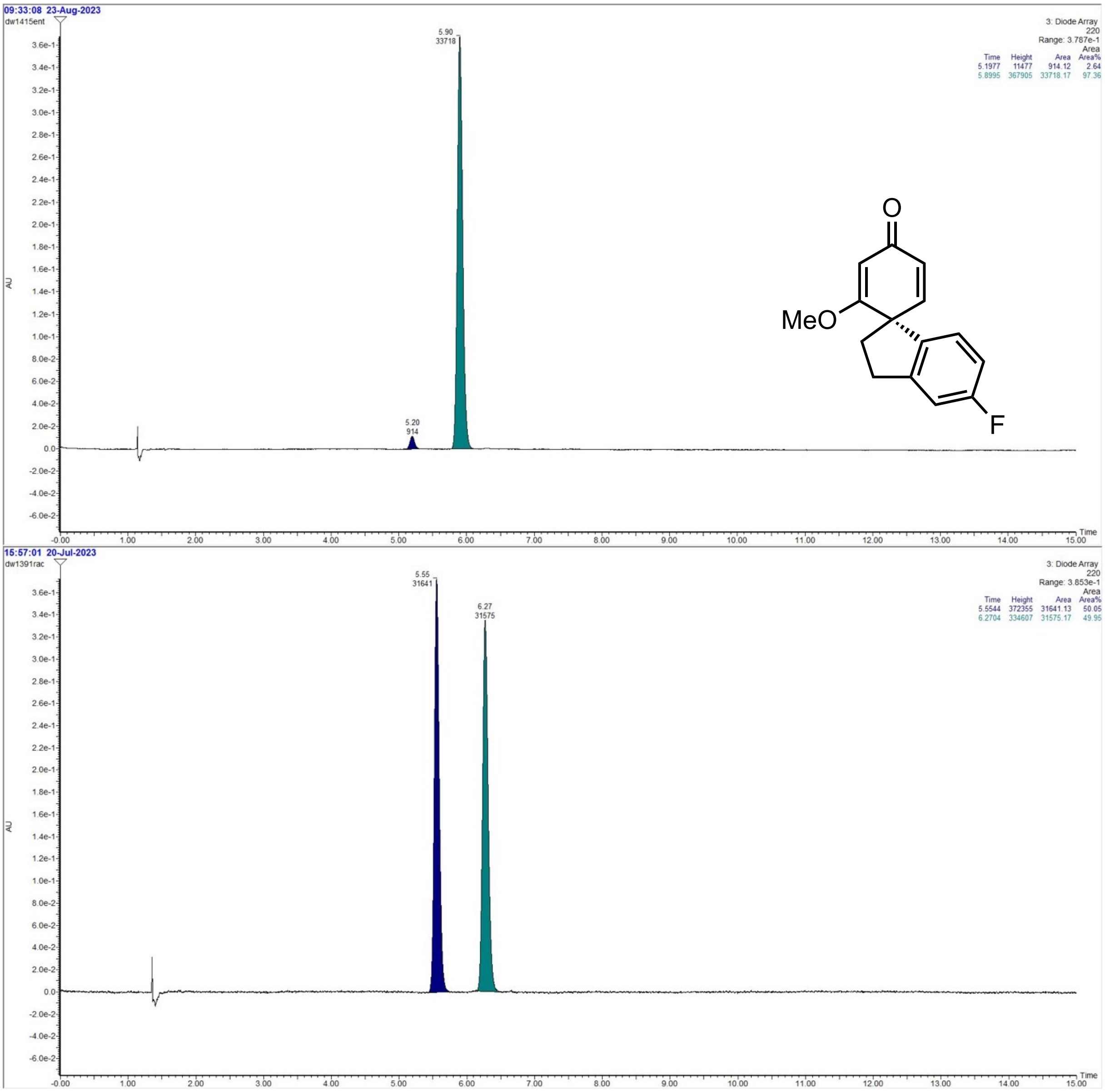

(*R*)-6’-Fluoro-2-methoxy-2',3'-dihydrospiro[cyclohexane-1,1'-indene]-2,5-dien-4-one (2s)

Chiral SFC Analysis: CHIRALPAK IG (CO_2:MeOH, 90:10, 2.5 mL min^{-1}, 40 °C, 220 nm) indicated 88% ee, t_R = 5.74 (minor), 6.24 (major) minutes.

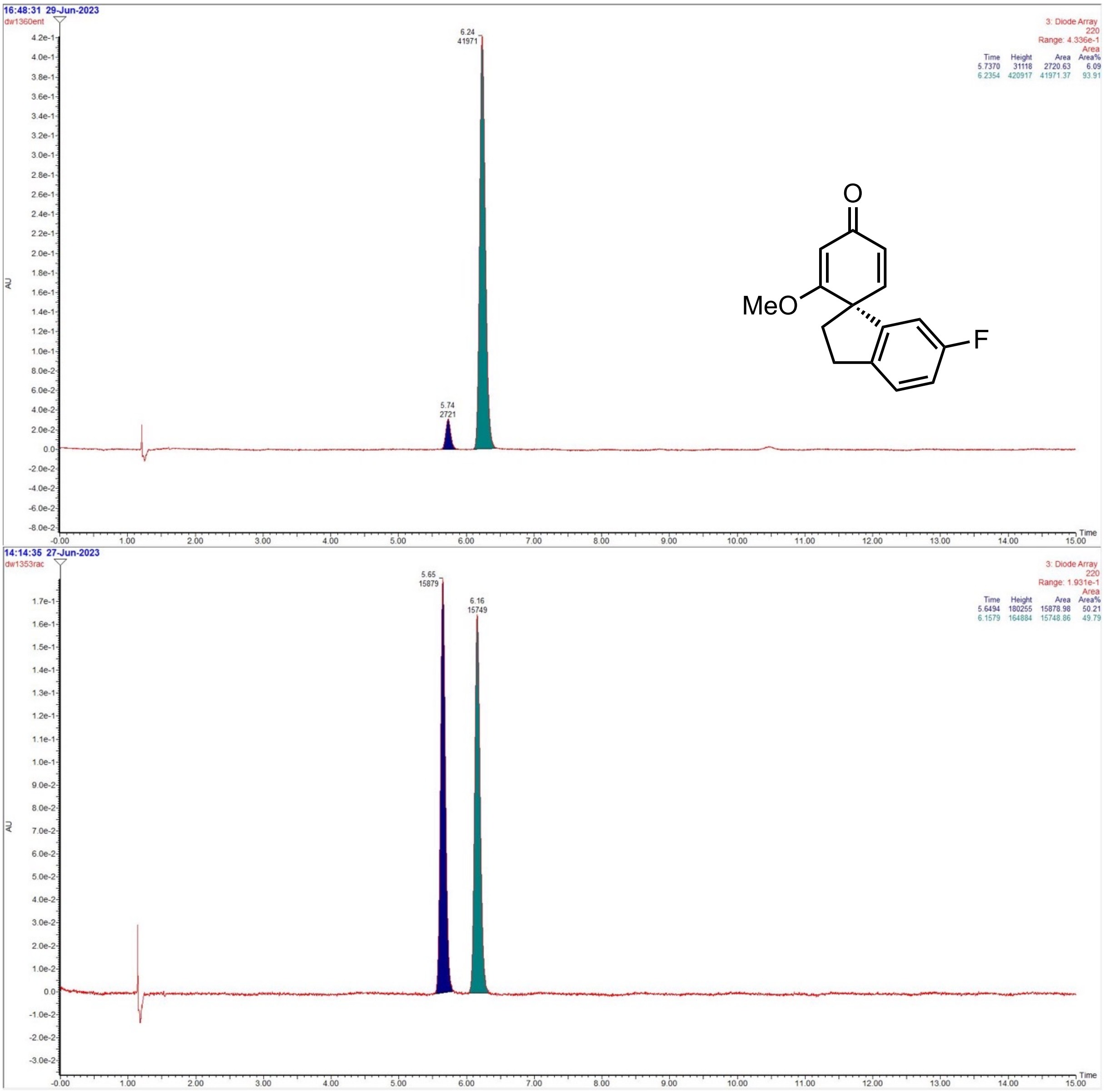

(*R*)-5,6,8,9-Tetrahydro-3*H*-indolo[7a,1-*a*]isoquinolin-3-one (4a) (from 3a)

Chiral SFC Analysis: CHIRALPAK IH (CO_2:MeOH, 90:10, 2.5 mL min^{-1}, 40 °C, 220 nm) indicated 93% ee, t_R = 7.56 (minor), 9.03 (major) minutes.

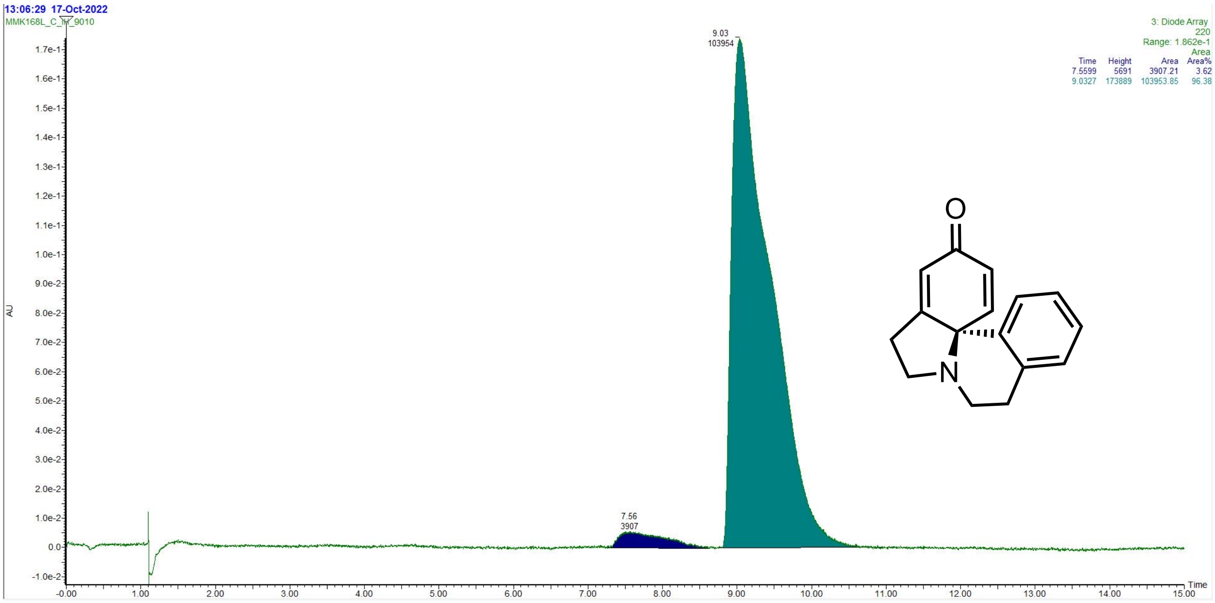

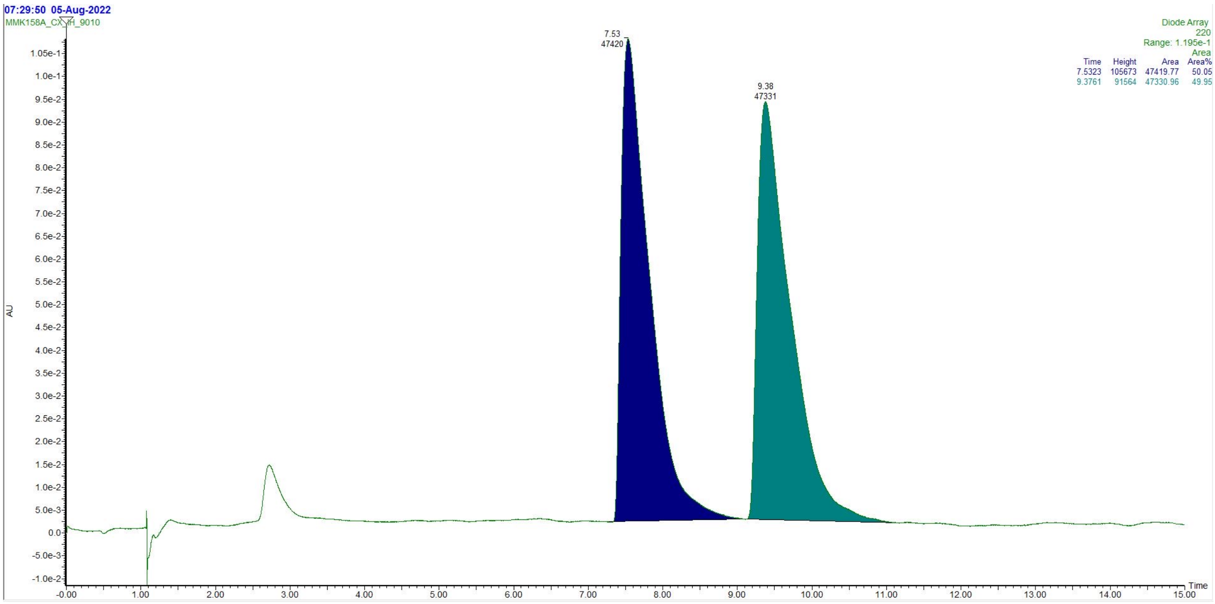

(*R*)-5,6,8,9-Tetrahydro-3*H*-indolo[7a,1-*a*]isoquinolin-3-one (4a) (from 3a')

Chiral SFC Analysis: CHIRALPAK IH (CO_2:MeOH, 90:10, 2.5 mL min^{-1}, 40 °C, 220 nm) indicated 89% ee, t_R = 7.70 (minor), 9.49 (major) minutes.

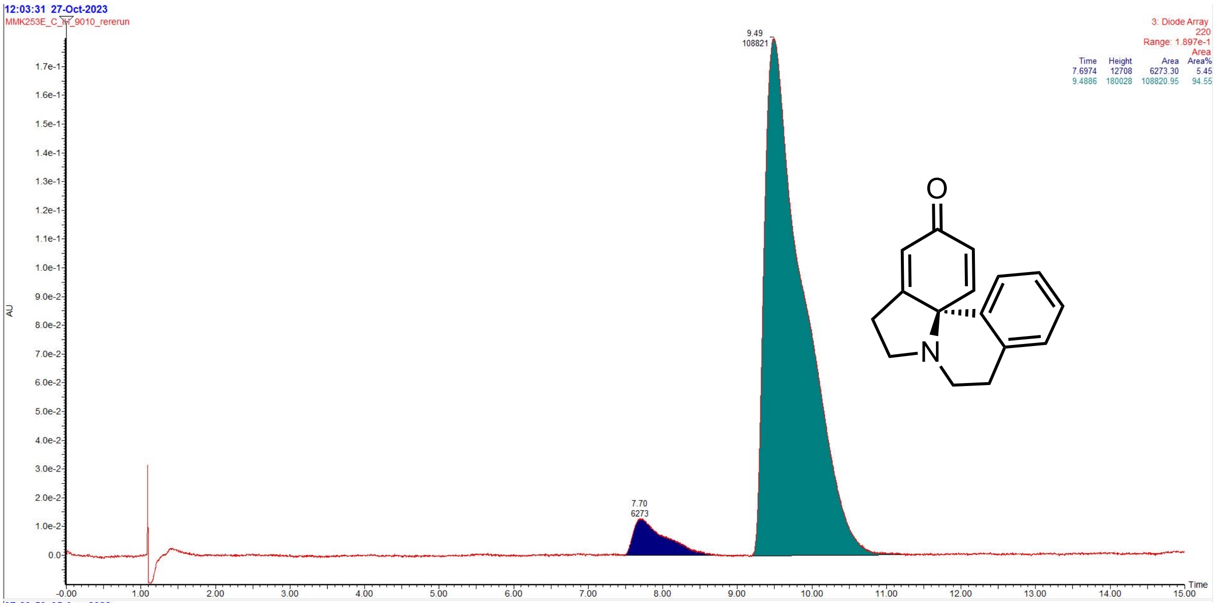

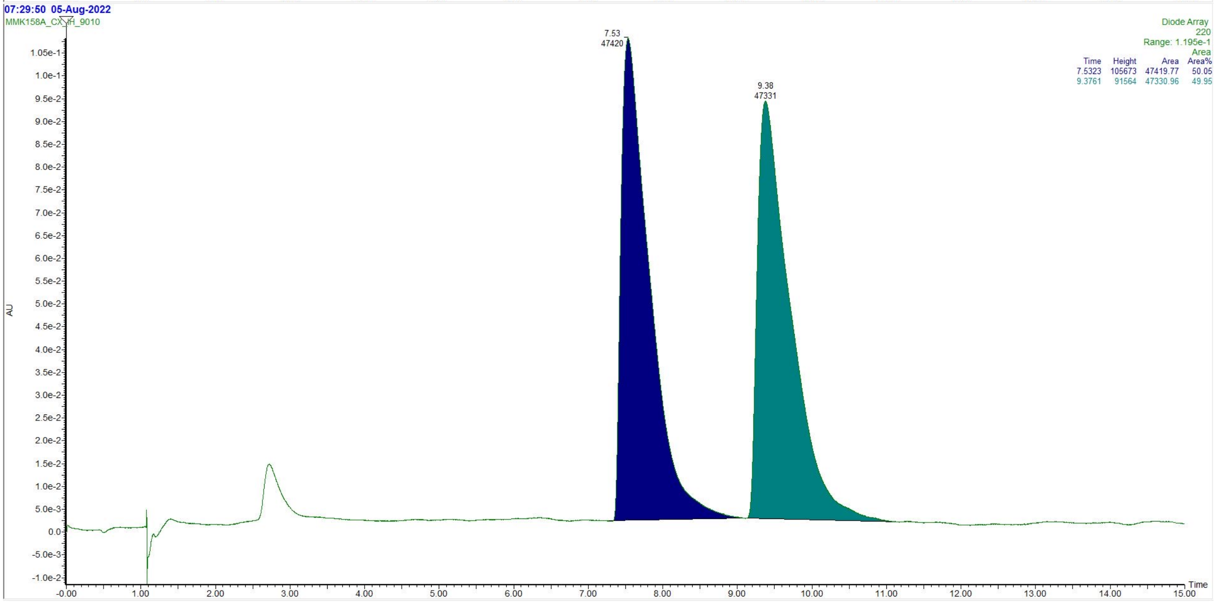

(*R*)-6,7,9,10-Tetrahydroisoquinolino[1,2-*j*]quinolin-3(5*H*)-one (4b)

Chiral HPLC Analysis: CHIRALPAK IG (Hexane:^{i}PrOH, 85:15, 1.25 mL min^{-1}, 40 °C, 220 nm) indicated 97% ee, t_R = 11.82 (minor), 13.60 (major) minutes.

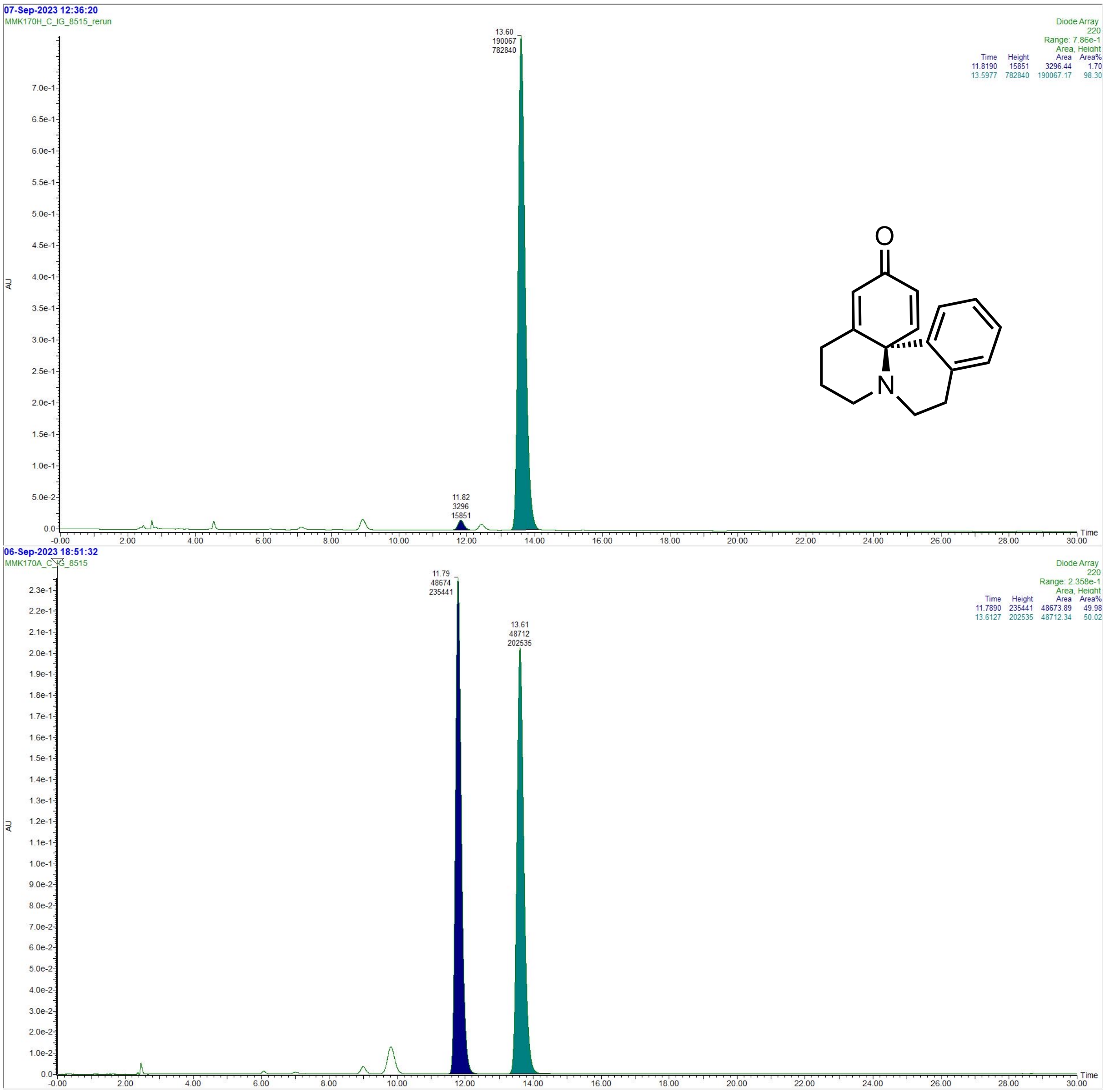

(*R*)-11-Methoxy-5,6,8,9-tetrahydro-3*H*-indolo[7a,1-*a*]isoquinolin-3-one (4c)

Chiral SFC Analysis: CHIRALPAK IG (CO_2:MeOH, 85:15, 2.5 mL min^{-1}, 40 °C, 220 nm) indicated 88% ee, t_R = 10.53 (major), 13.54 (minor) minutes.

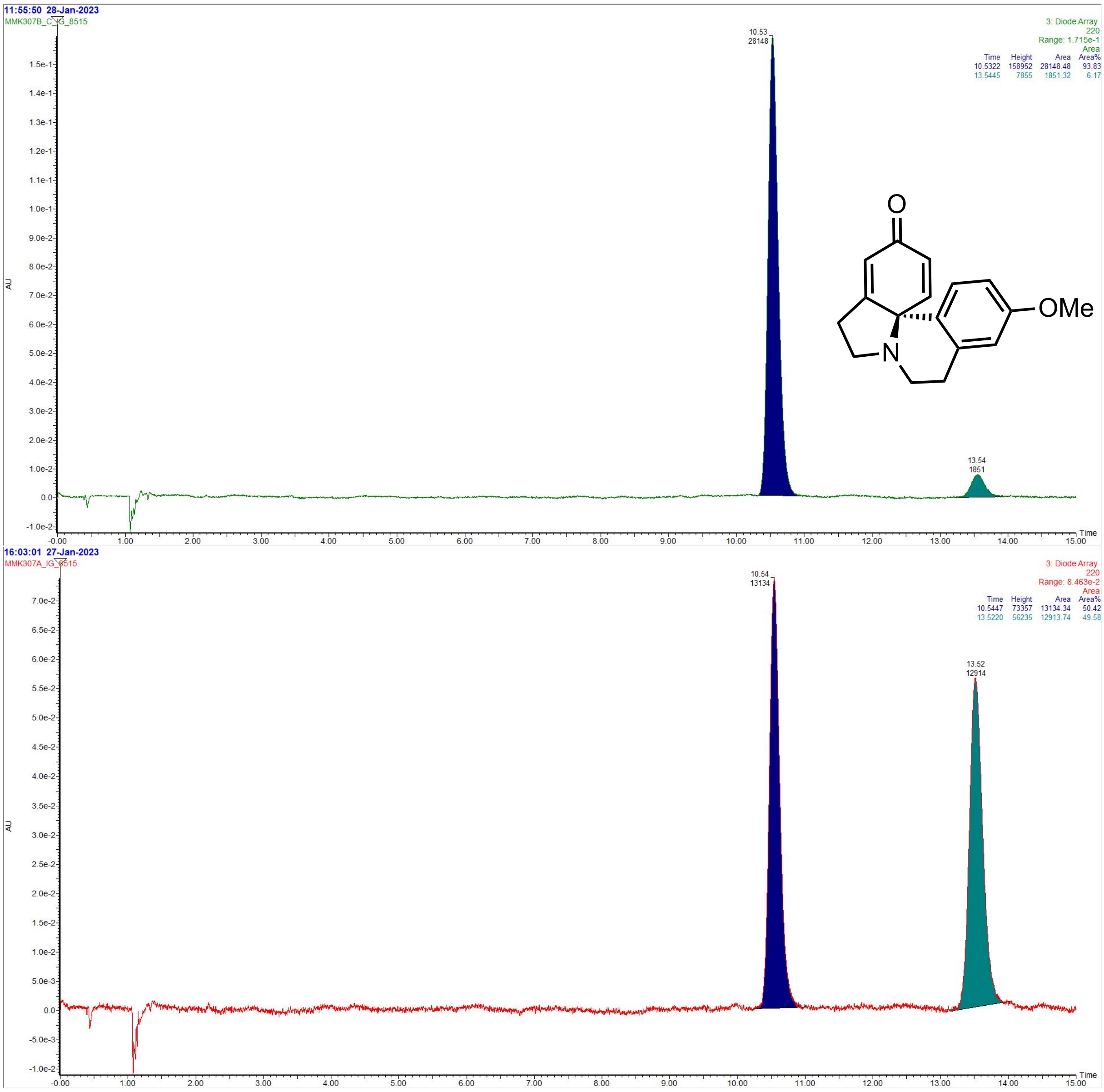

(*R*)-11-Chloro-5,6,8,9-tetrahydro-3*H*-indolo[7a,1-*a*]isoquinolin-3-one (4d)

Chiral SFC Analysis: CHIRALPAK IH (CO_2:MeOH, 90:10, 2.5 mL min^{-1}, 40 °C, 220 nm) indicated 94% ee, t_R = 8.21 (minor), 10.77 (major) minutes.

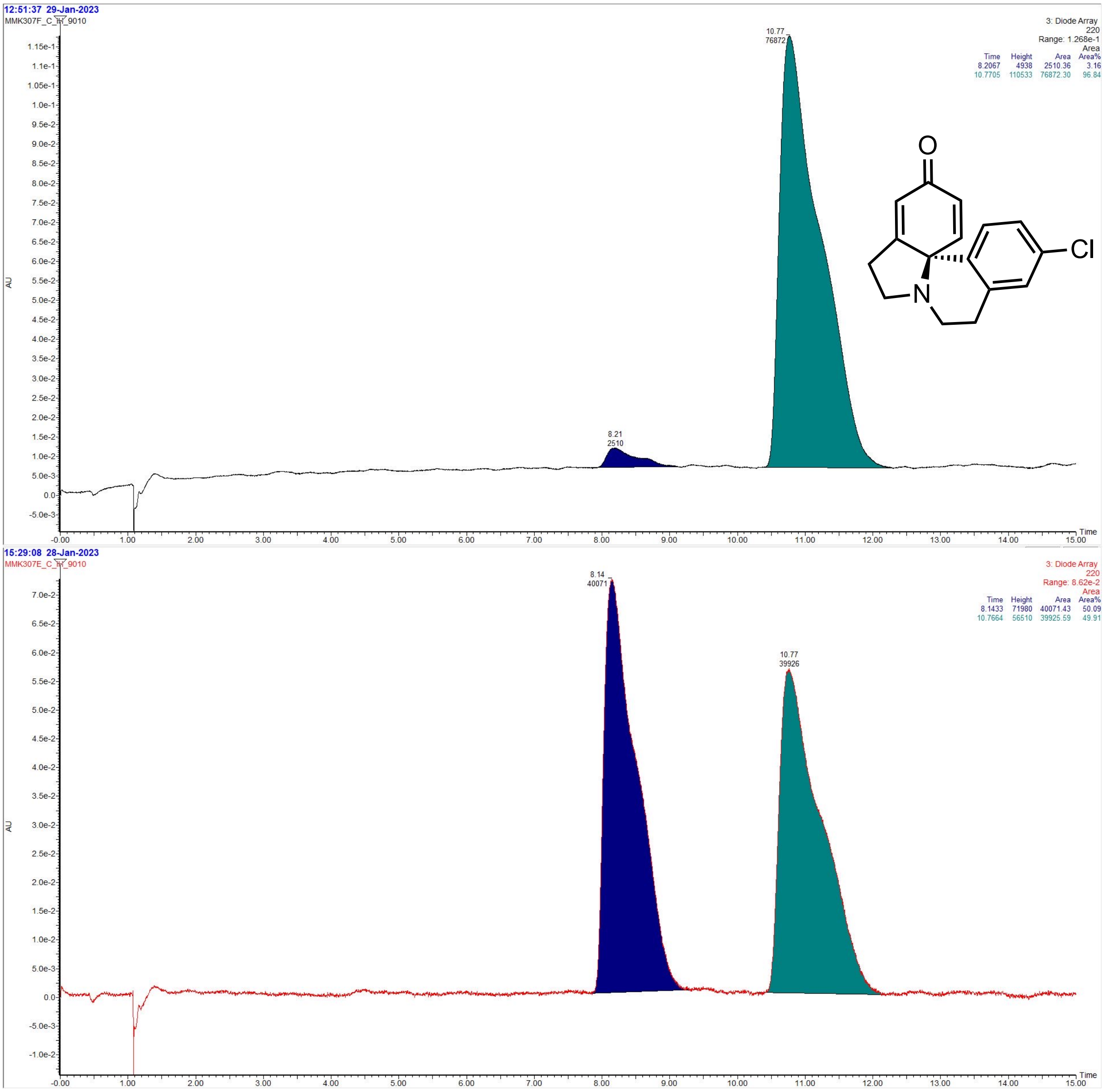

(*R*)-11-(trifluoromethyl)-5,6,8,9-Tetrahydro-3*H*-indolo[7a,1-*a*]isoquinolin-3-one (4e)

Chiral SFC Analysis: CHIRALPAK IG (CO_2:MeOH, 85:15, 2.5 mL min^{-1}, 40 °C, 220 nm) indicated 92% ee, t_R = 3.35 (minor), 3.73 (major) minutes.

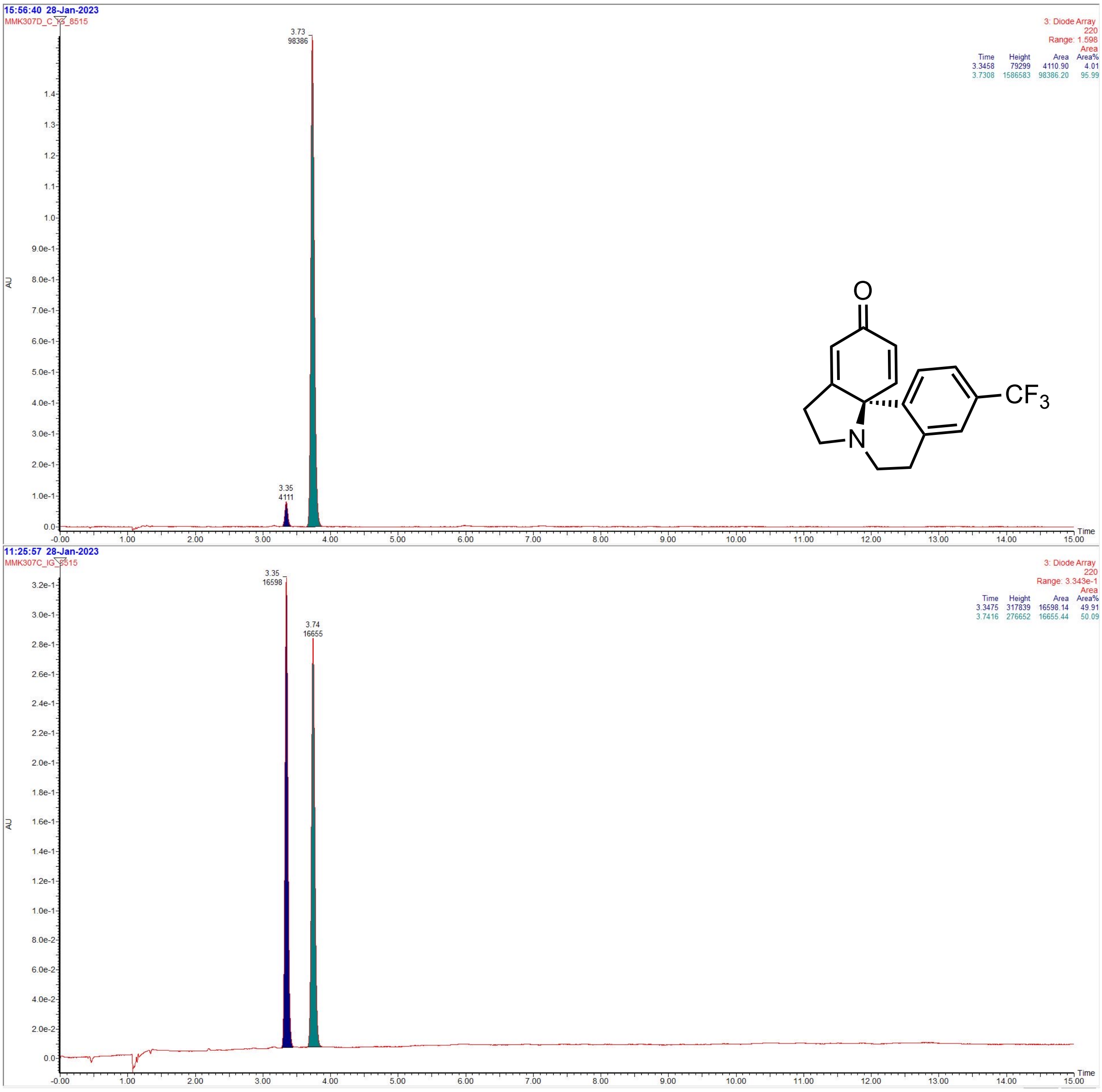

(*R*)-12,13-Dimethoxy-6,7,9,10-tetrahydroisoquinolino[1,2-*j*]quinolin-3(5*H*)-one (4f)

Chiral HPLC Analysis: CHIRALPAK IG (Hexane:^{i}PrOH, 75:25, 1.25 mL min^{-1}, 40 °C, 220 nm) indicated 91% ee, t_R = 11.31 (major), 16.80 (minor) minutes.

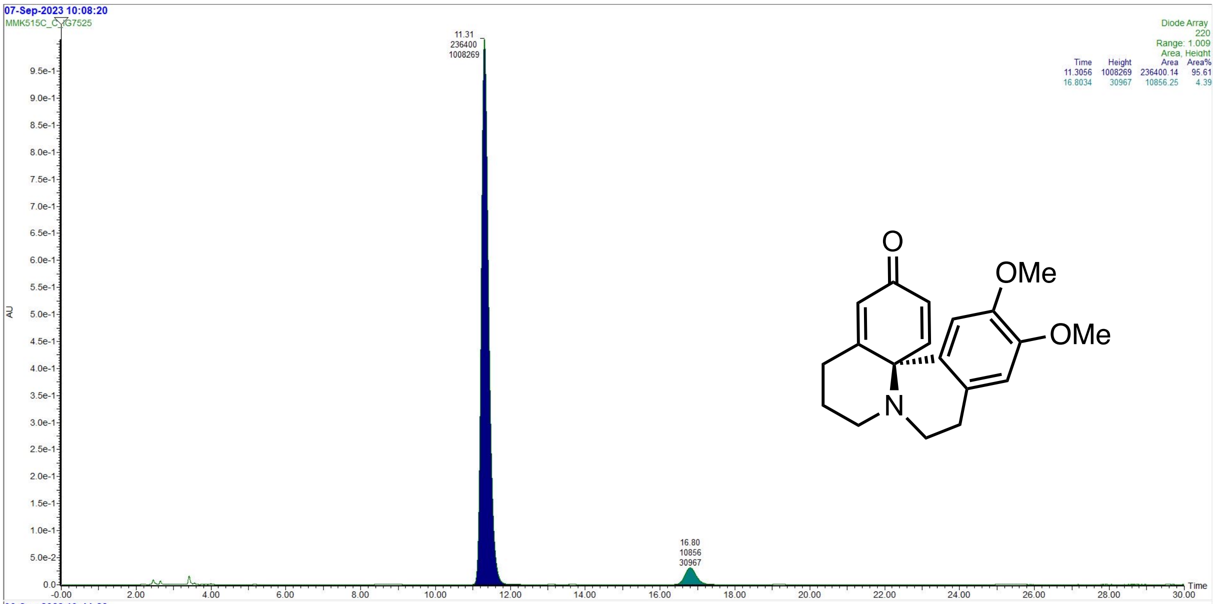

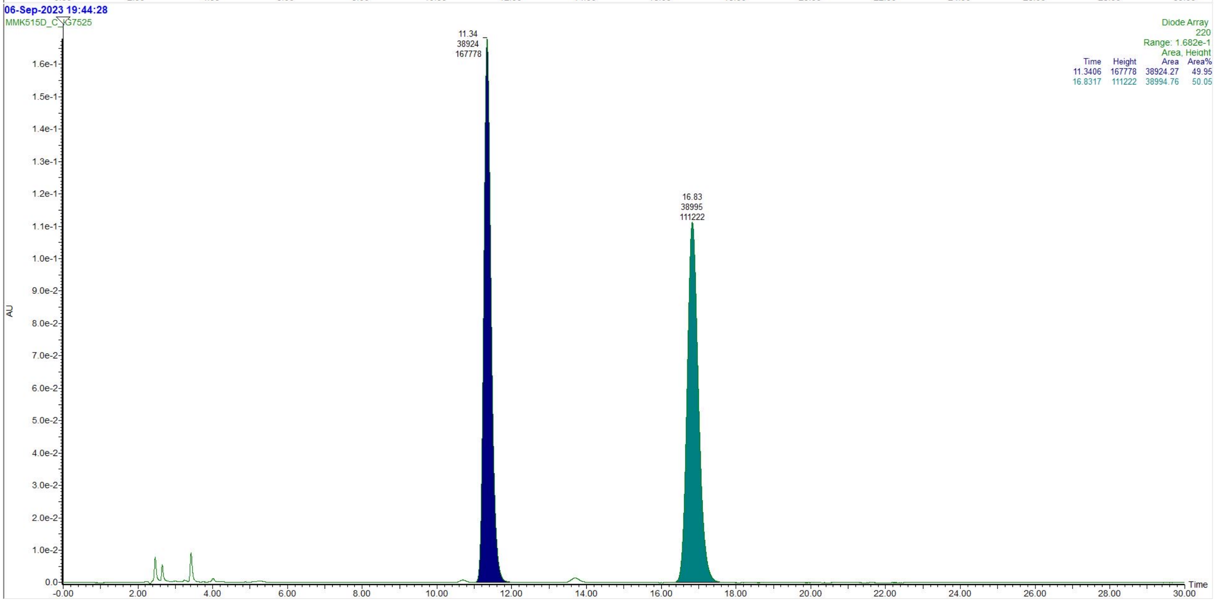

(*R*)-11,12-Dimethoxy-5,6,8,9-tetrahydro-3*H*-indolo[7a,1-*a*]isoquinolin-3-one (4g)

Chiral SFC Analysis: CHIRALPAK IK (CO_2:MeOH, 85:15, 2.5 mL min^{-1}, 40 °C, 220 nm) indicated 88% ee, t_R = 9.75 (major), 11.66 (minor) minutes.

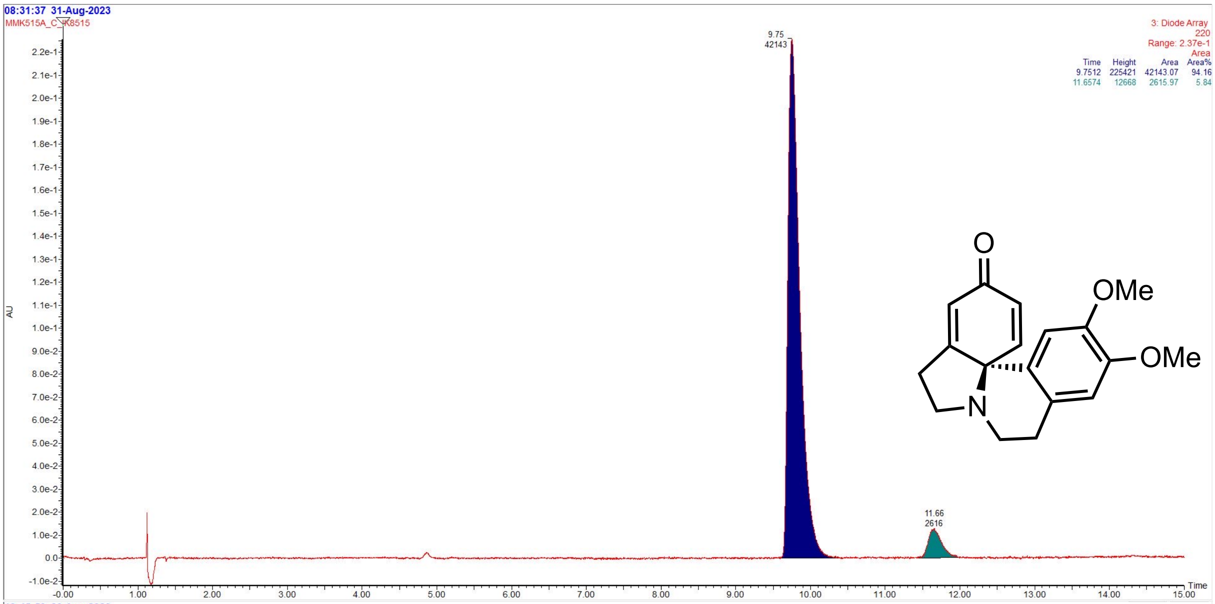

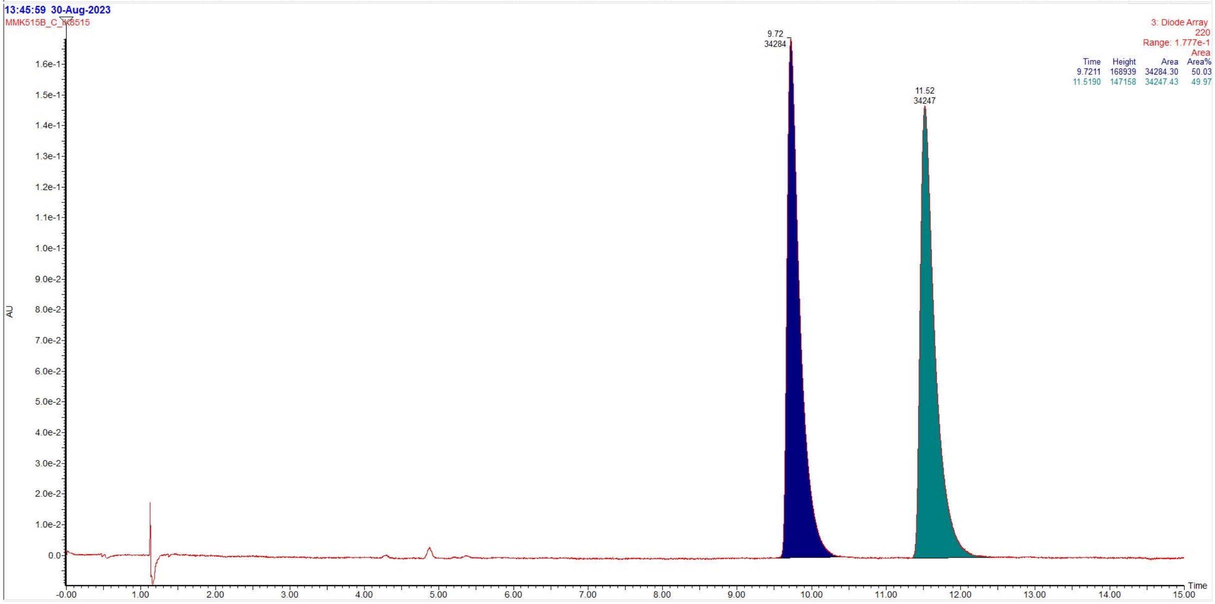

(*S*)-4a-Methyl-9,10-dihydrophenanthren-2(4aH)-one (7a)

Chiral SFC Analysis: CHIRALPAK IG (CO_2:MeOH, 85:15, 1.25 mL min^{-1}, 40 °C, 240 nm) indicated 93% ee, t_R = 6.60 (major), 8.31 (minor) minutes.

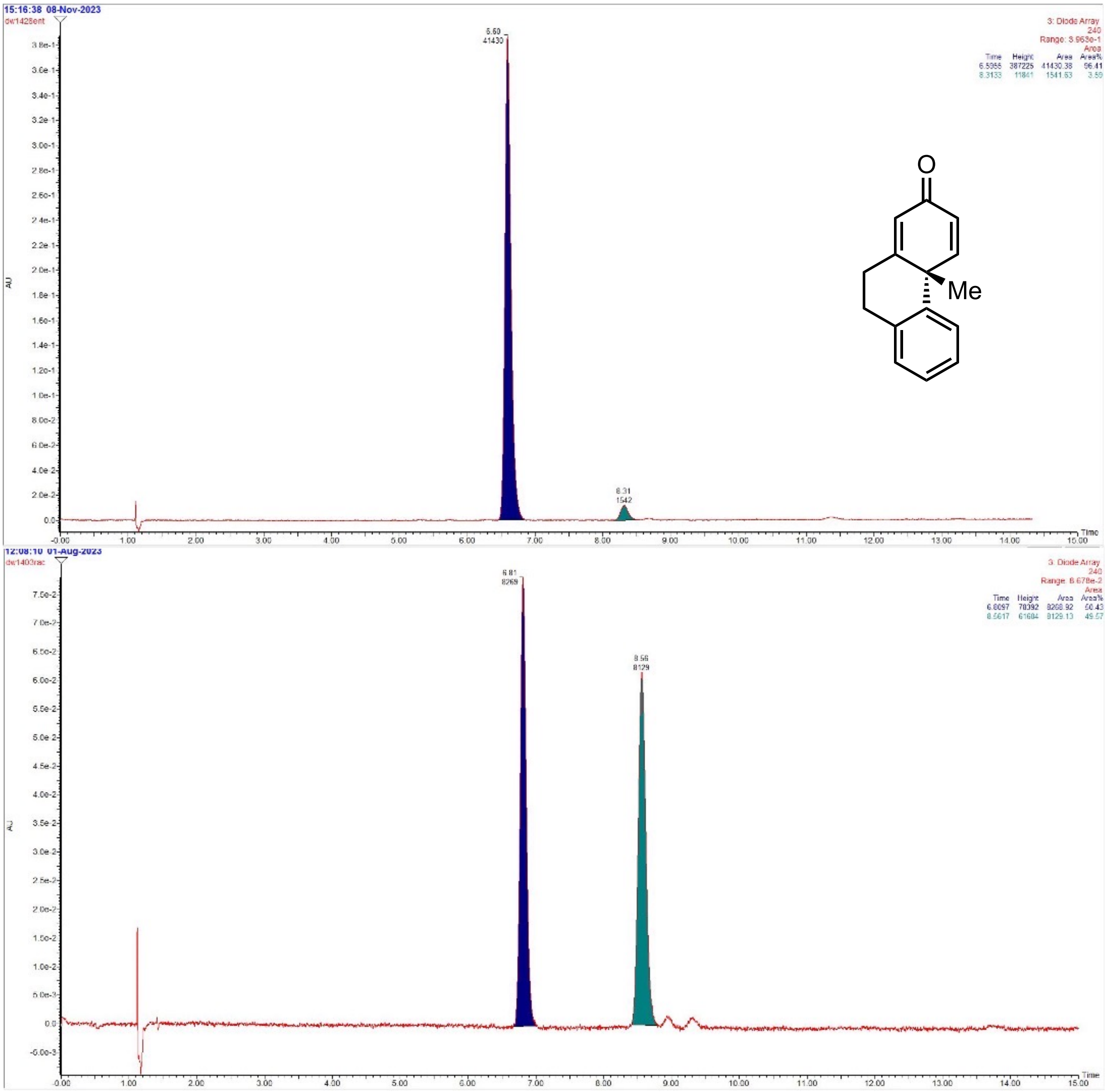

(*S*)-4a-Ethyl-9,10-dihydrophenanthren-2(4a*H*)-one (7b)

Chiral SFC Analysis: CHIRALPAK IE (CO_2:MeOH, 85:15, 2.5 mL min^{-1}, 40 °C, 220 nm) indicated 91% ee, t_R = 7.45 (major), 7.98 (minor) minutes.

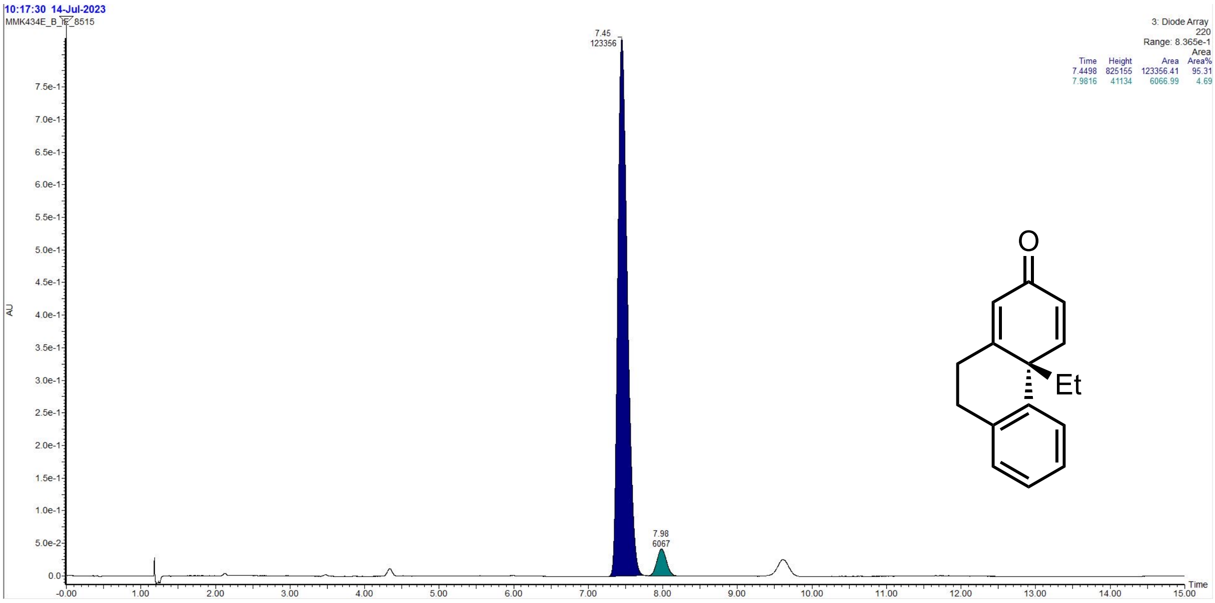

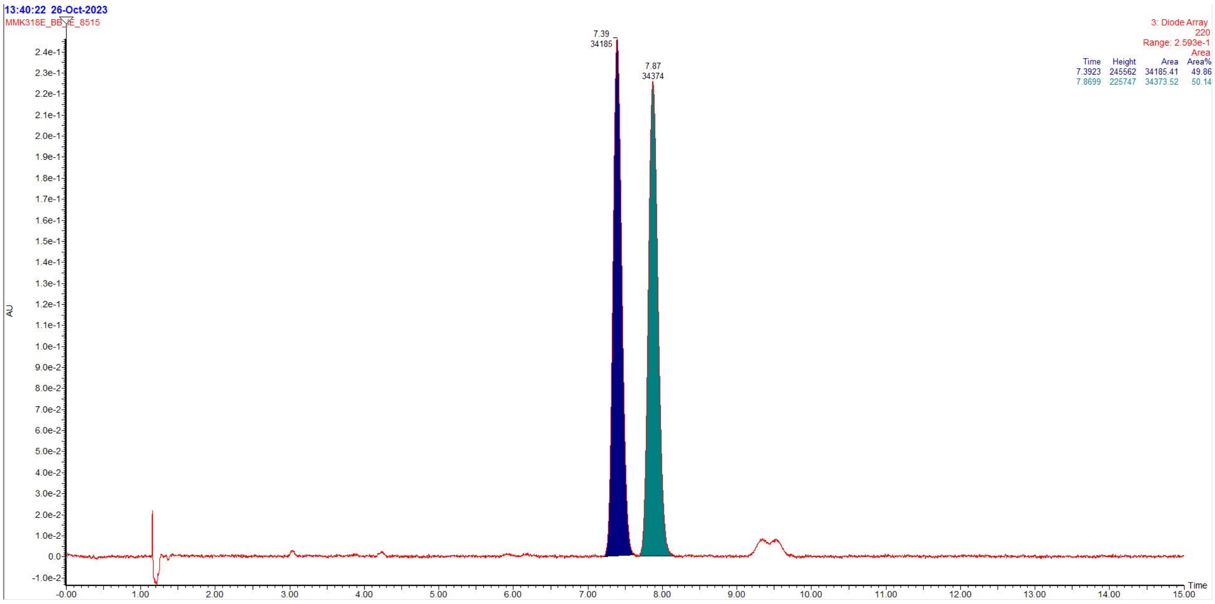

(*S*)-8-Chloro-4a-methyl-9,10-dihydrophenanthren-2(4aH)-one (7c)

Chiral SFC Analysis: CHIRALPAK IA (CO_2:MeOH, 85:15, 2.5 mL min^{-1}, 40 ℃, 224 nm) indicated 92% ee, t_R= 4.52 (minor), 4.96 (major) minutes.

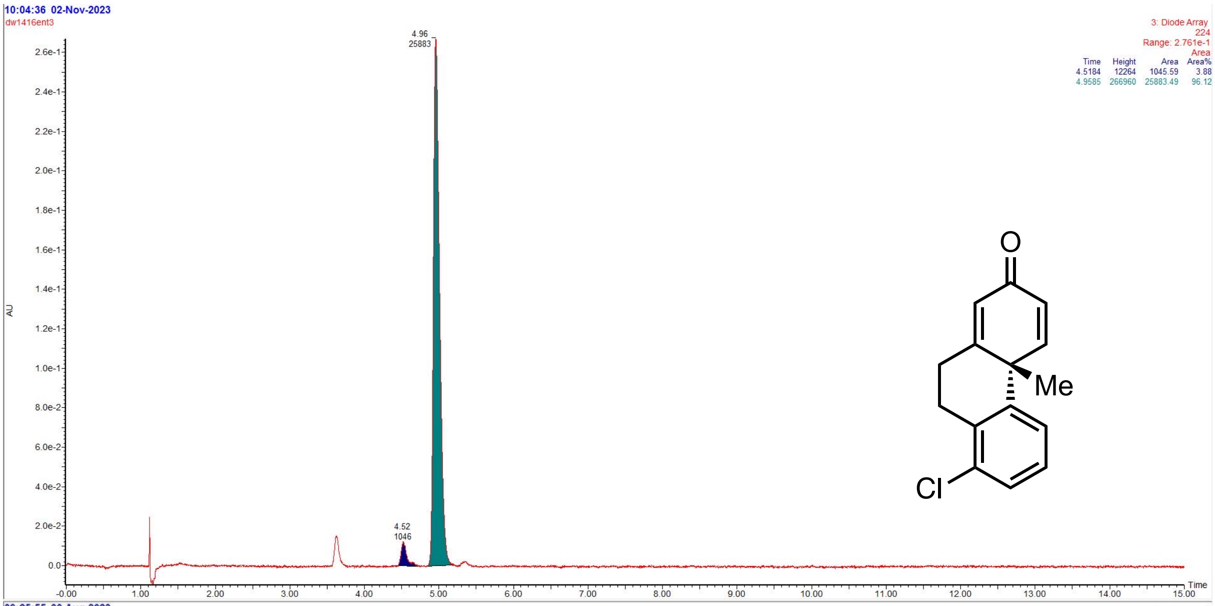

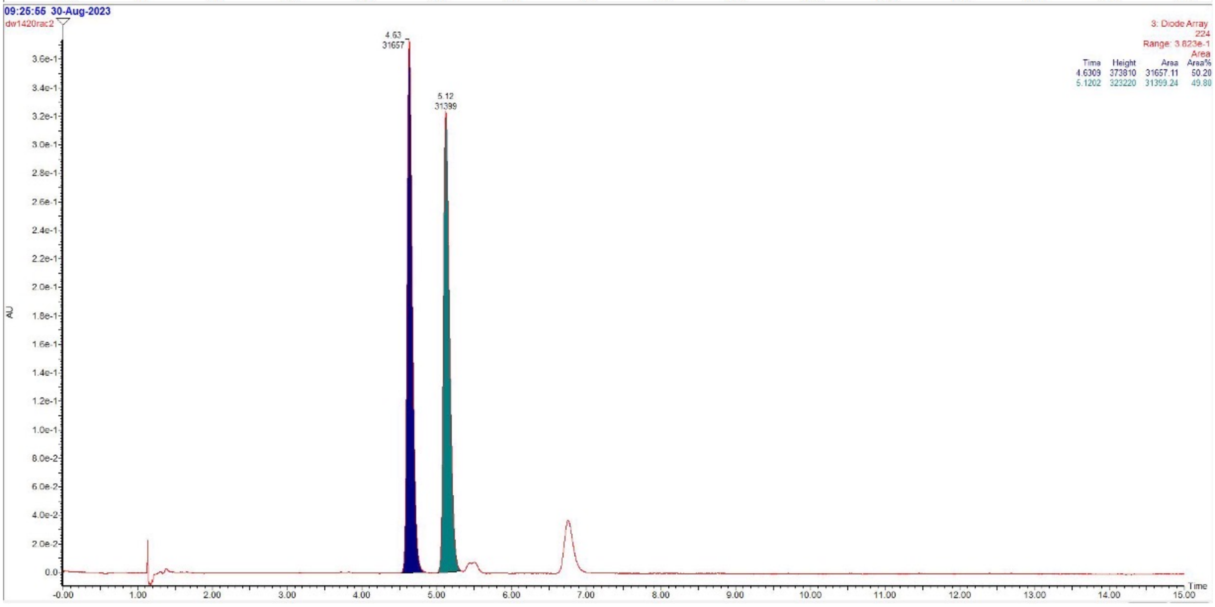

(*S*)-7-Fluoro-4a-methyl-9,10-dihydrophenanthren-2(4aH)-one (7d)

Chiral HPLC analysis: CHIRALPACK AD-H (Hexane: ^{i}PrOH, 90:10, 1.25 mL min^{-1}, 40 °C, 232 nm) indicated 90% ee t_R= 7.33 (major), 8.45 (minor) minutes.

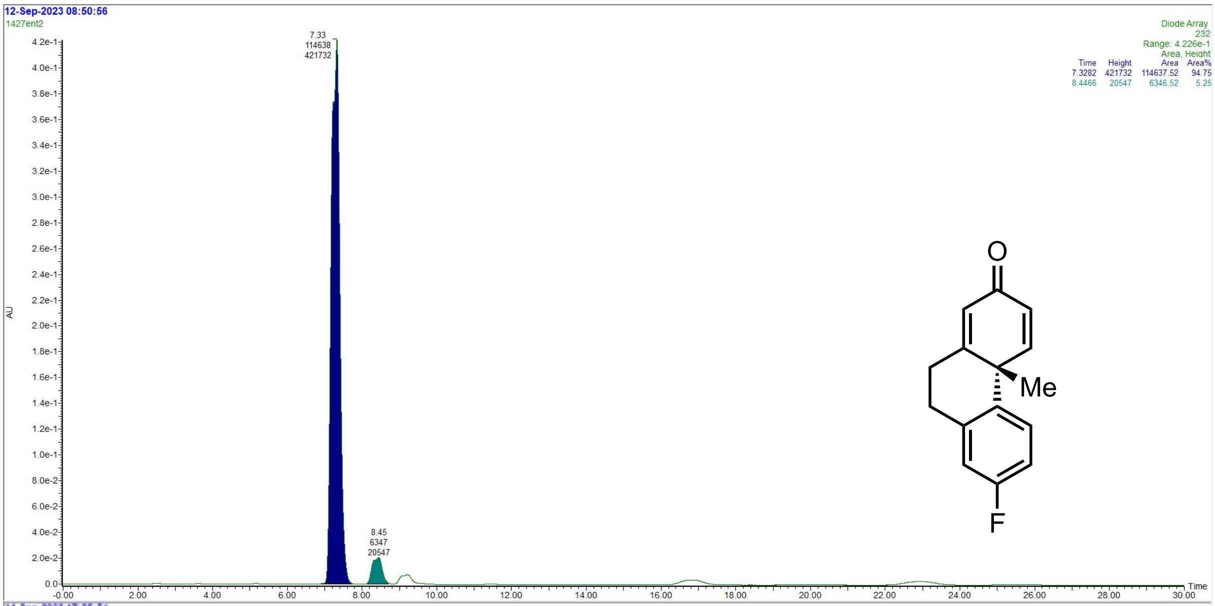

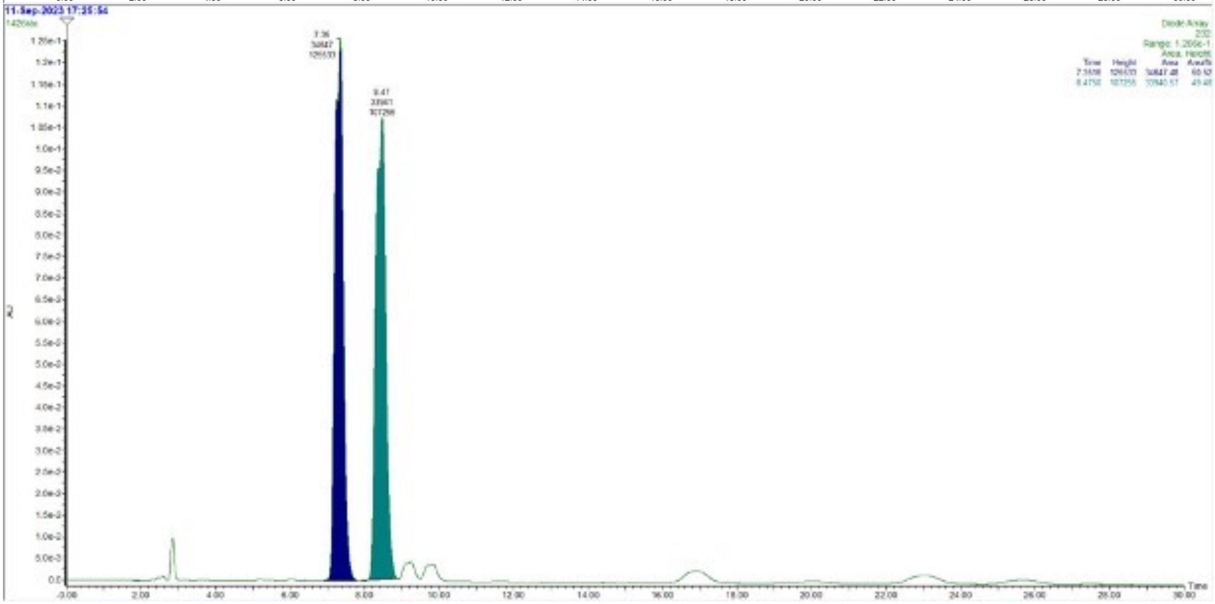

(*S*)-9,9,11b-Trimethyl-6,11b-dihydrophenanthro[2,3-d][1,3]dioxol-3(5H)-one (7e)

Chiral SFC Analysis: CHIRALPAK IG (CO_2:MeOH, 85:15, 2.5 mL min^{-1}, 40 °C, 220 nm) indicated 90% ee, t_R= 6.52 (major), 11.79 (minor) minutes.

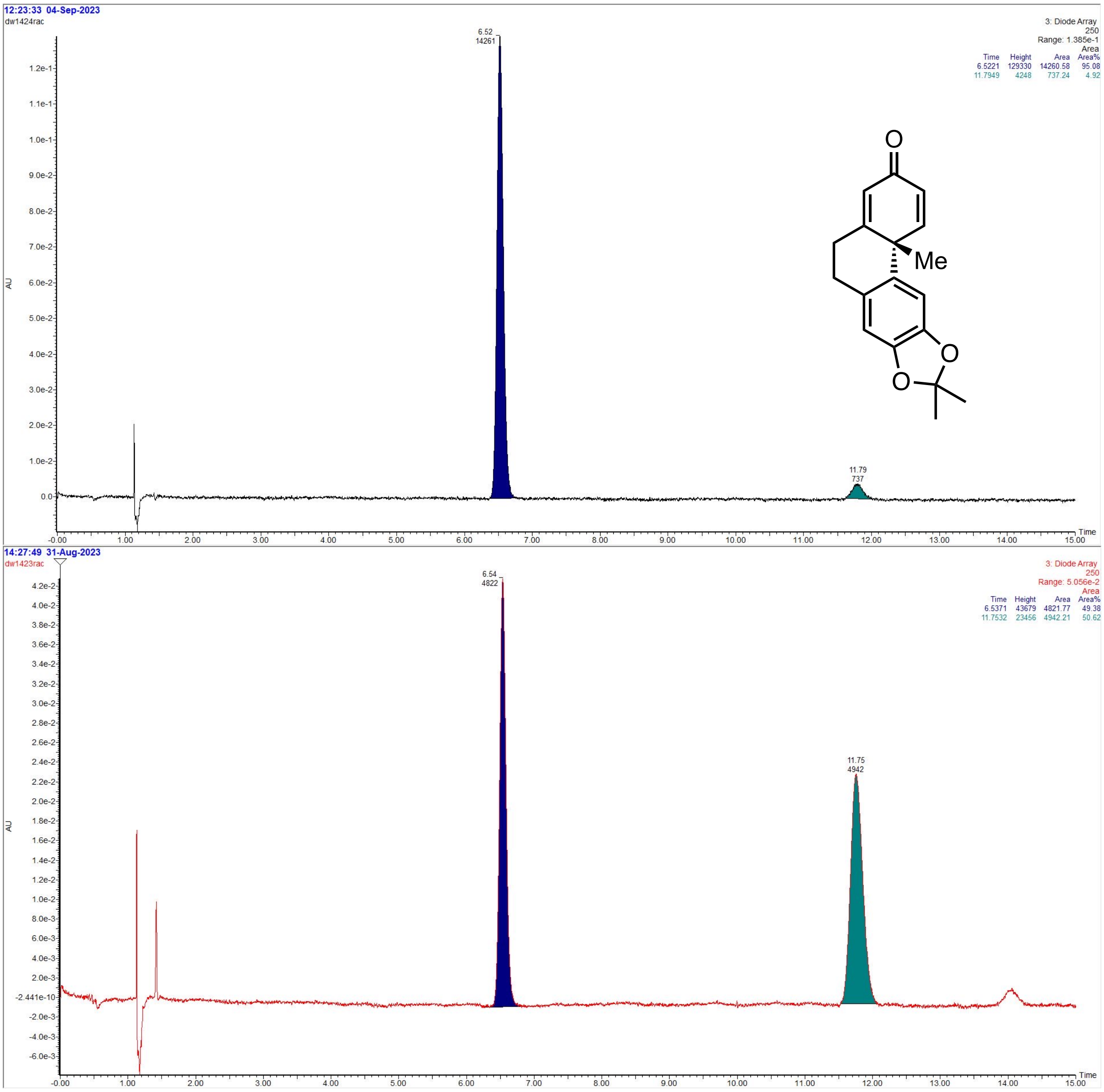

(*R*)-2-Methyl-3'*H*-spiro[cyclohexane-1,1'-isobenzofuran]-2,5-dien-4-one (9a)

Chiral SFC Analysis: CHIRALPAK IK (CO_2:MeOH, 90:10, 2.5 mL min^{-1}, 40 °C, 250 nm) indicated 83% ee, t_R = 3.74 (major), 4.12 (minor) minutes.

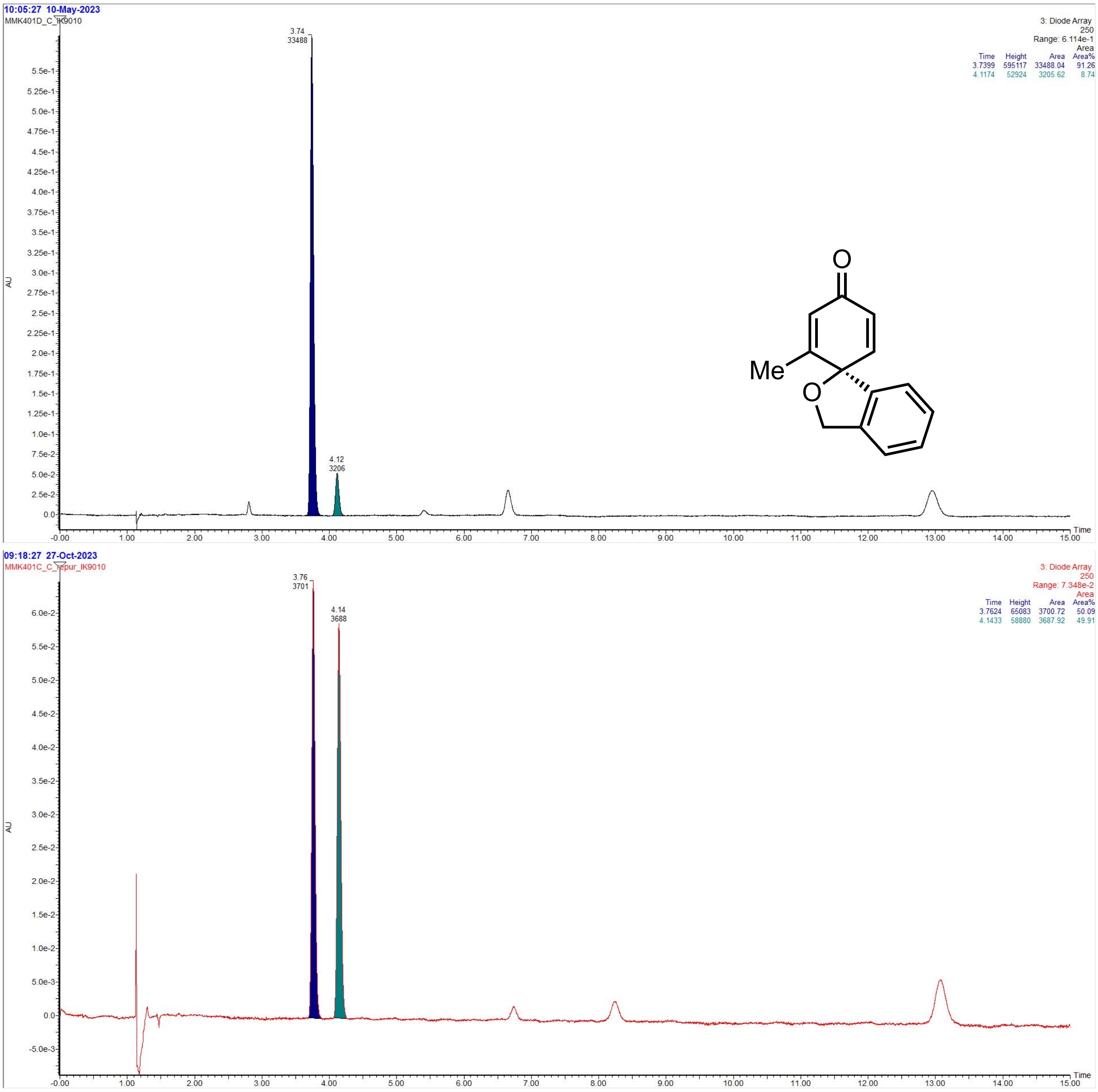

(*S*)-2-Methoxy-3'*H*-spiro[cyclohexane-1,1'-isobenzofuran]-2,5-dien-4-one (9b)

Chiral SFC Analysis: CHIRALPAK IG (CO_2:MeOH, 95:05, 2.5 mL min^{-1}, 40 °C, 220 nm) indicated 82% ee, t_R = 10.86 (minor), 13.25 (major) minutes.

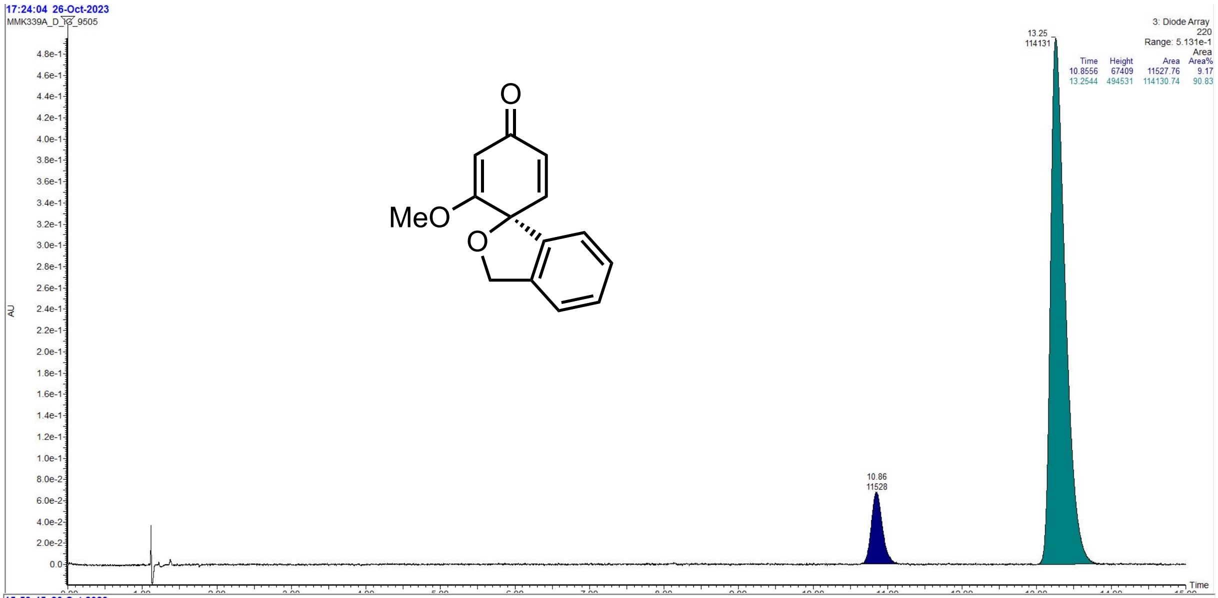

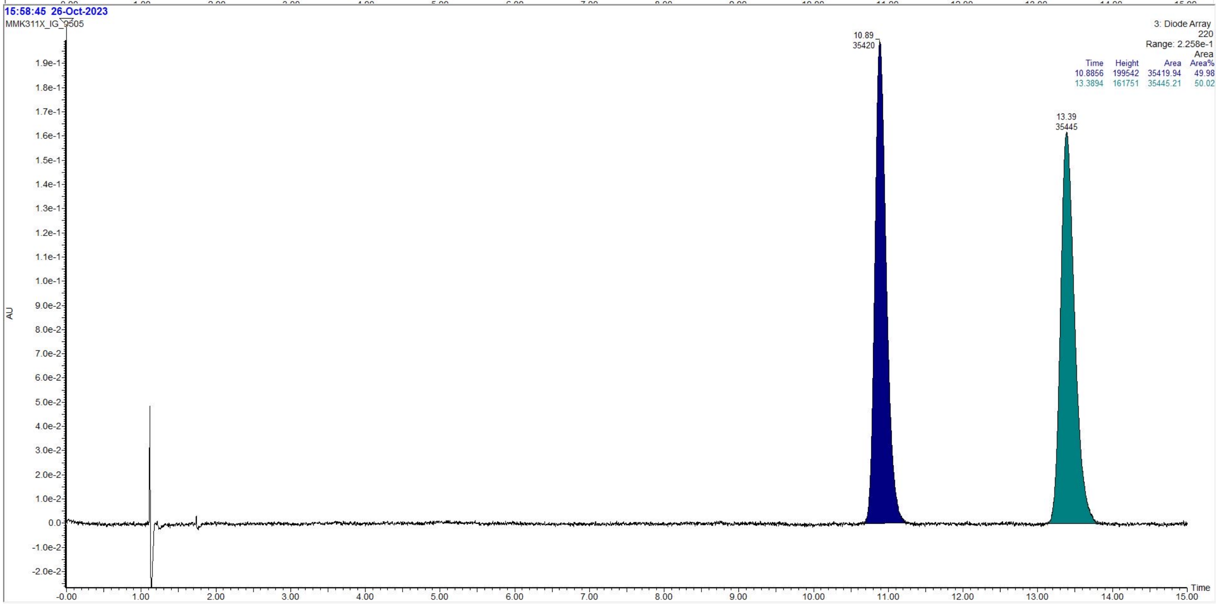

(*R*)-4'-Chloro-2,3-dimethyl-3'*H*-spiro[cyclohexane-1,1'-isobenzofuran]-2,5-dien-4-one (9c)

Chiral SFC Analysis: CHIRALPAK IK (CO_2:MeOH, 90:10, 2.5 mL min^{-1}, 40 °C, 220 nm) indicated 93% ee, t_R = 3.69 (major), 4.61 (minor) minutes.

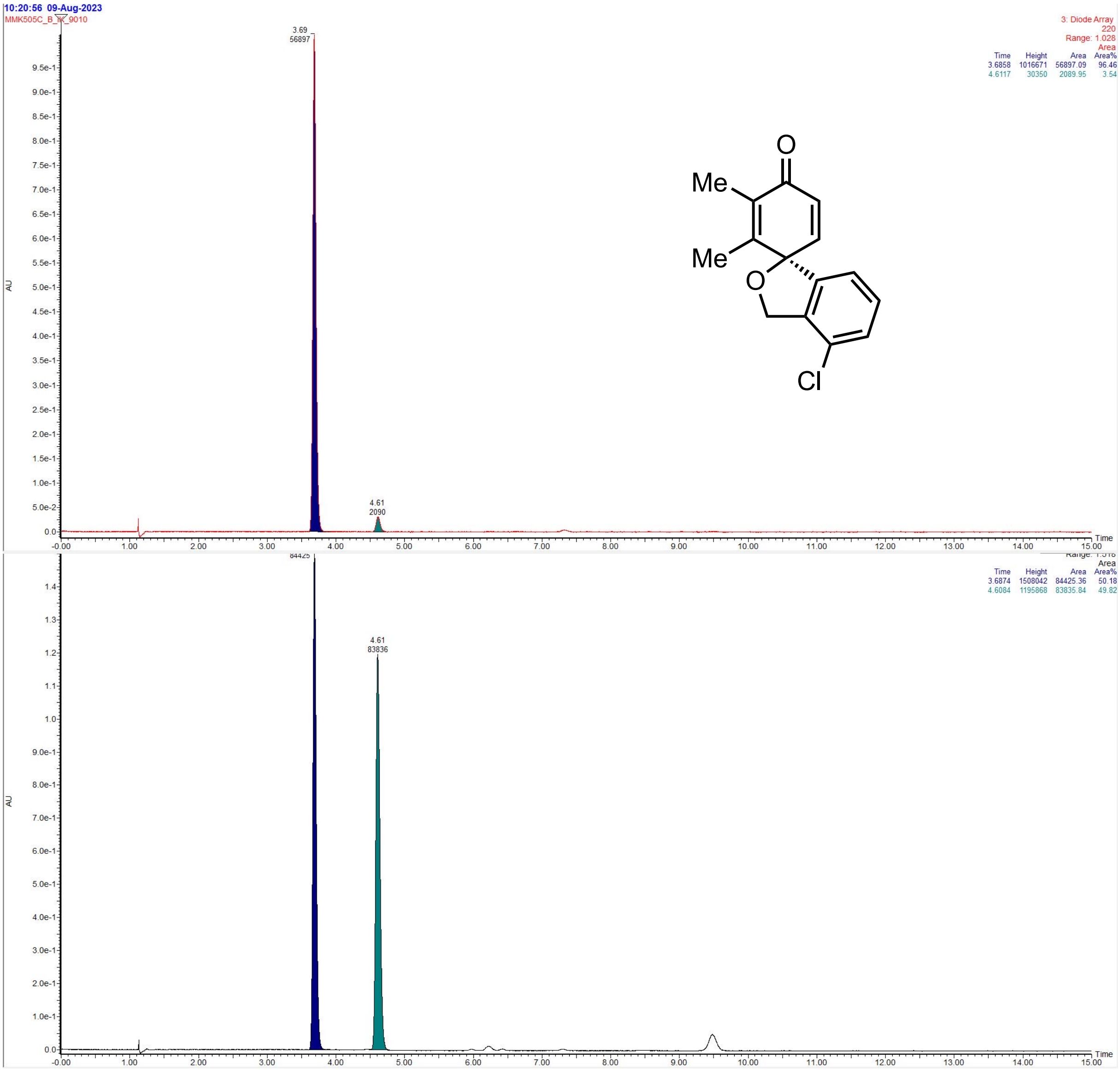

(*R*)-Neopentyl 2'-(dicyclohexylphosphoryl)-2,6-dimethoxy-[1,1'-biphenyl]-3-sulfonate (S39)

Chiral HPLC Analysis: CHIRALPAK IG (CO_2: ^{i}PrOH, 85:15, 1.25 mL min^{-1}, 40 °C, 210 nm) indicated > 99% ee, t_R = 18.15 (minor), 20.24 (major) minutes.

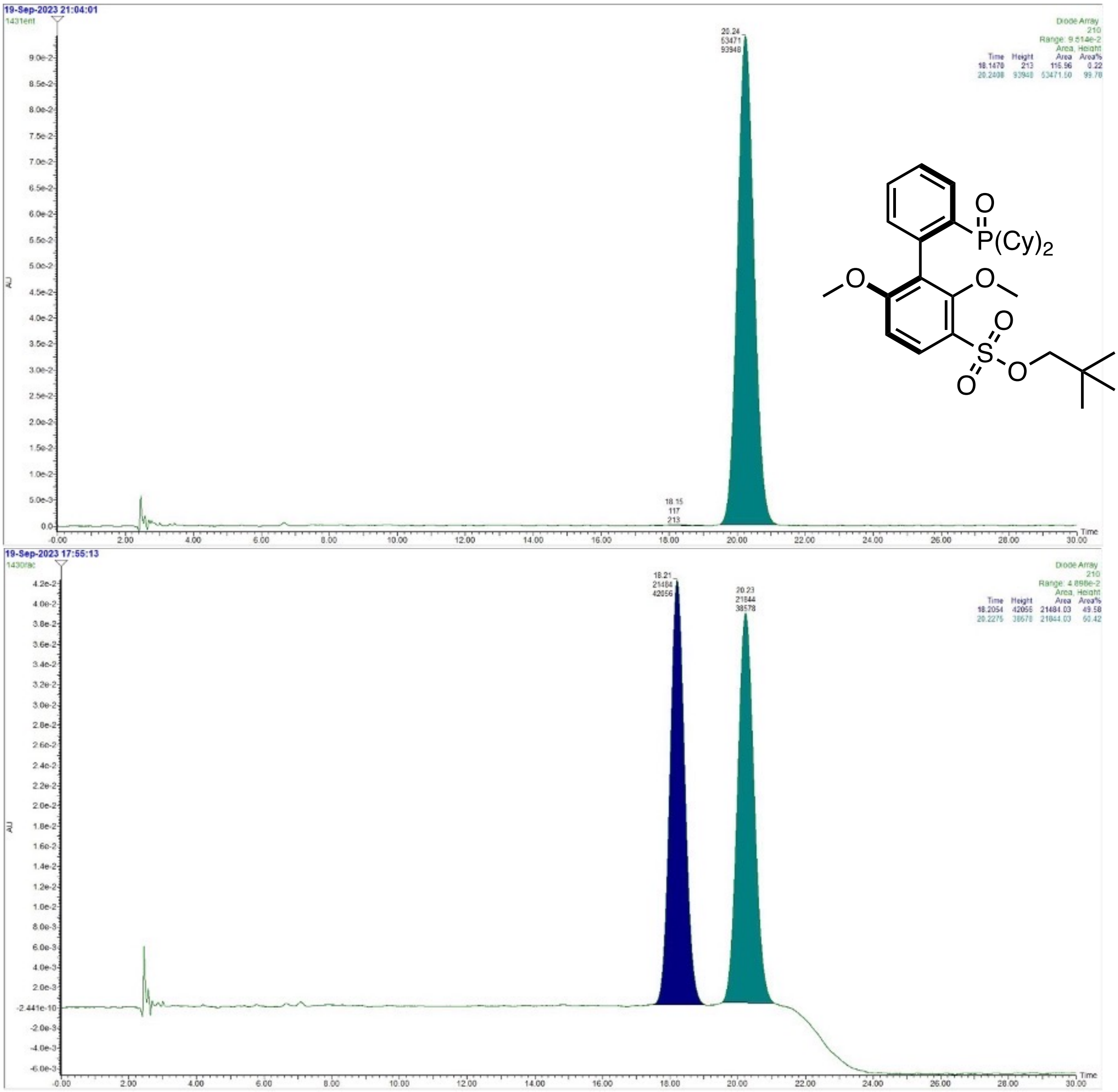

(*R*)-Neopentyl 2'-(dicyclohexylphosphaneyl)-2,6-dimethoxy-[1,1'-biphenyl]-3-sulfonate (*R*)-sSPhos-Np

Chiral HPLC Analysis: CHIRALPAK IG (CO_2: ^{i}PrOH, 95:05, 1.25 mL min^{-1}, 40 °C, 210 nm) indicated > 99% ee, t_R = 5.29 (minor), 5.84 (major) minutes.

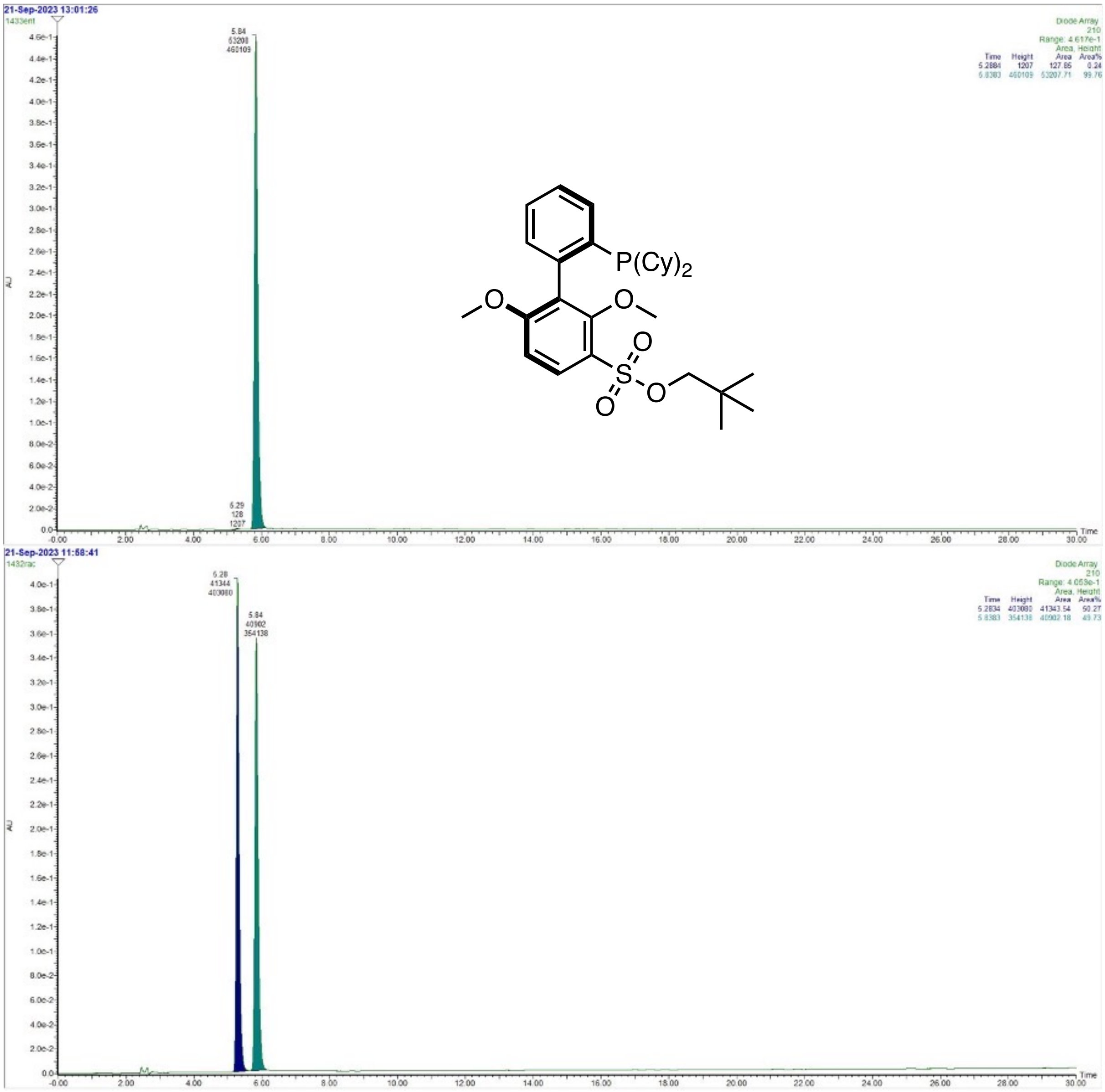

^{1}H NMR ($CDCl_3$): 4-(benzyloxy)-2-Methoxybenzaldehyde **(S4)**

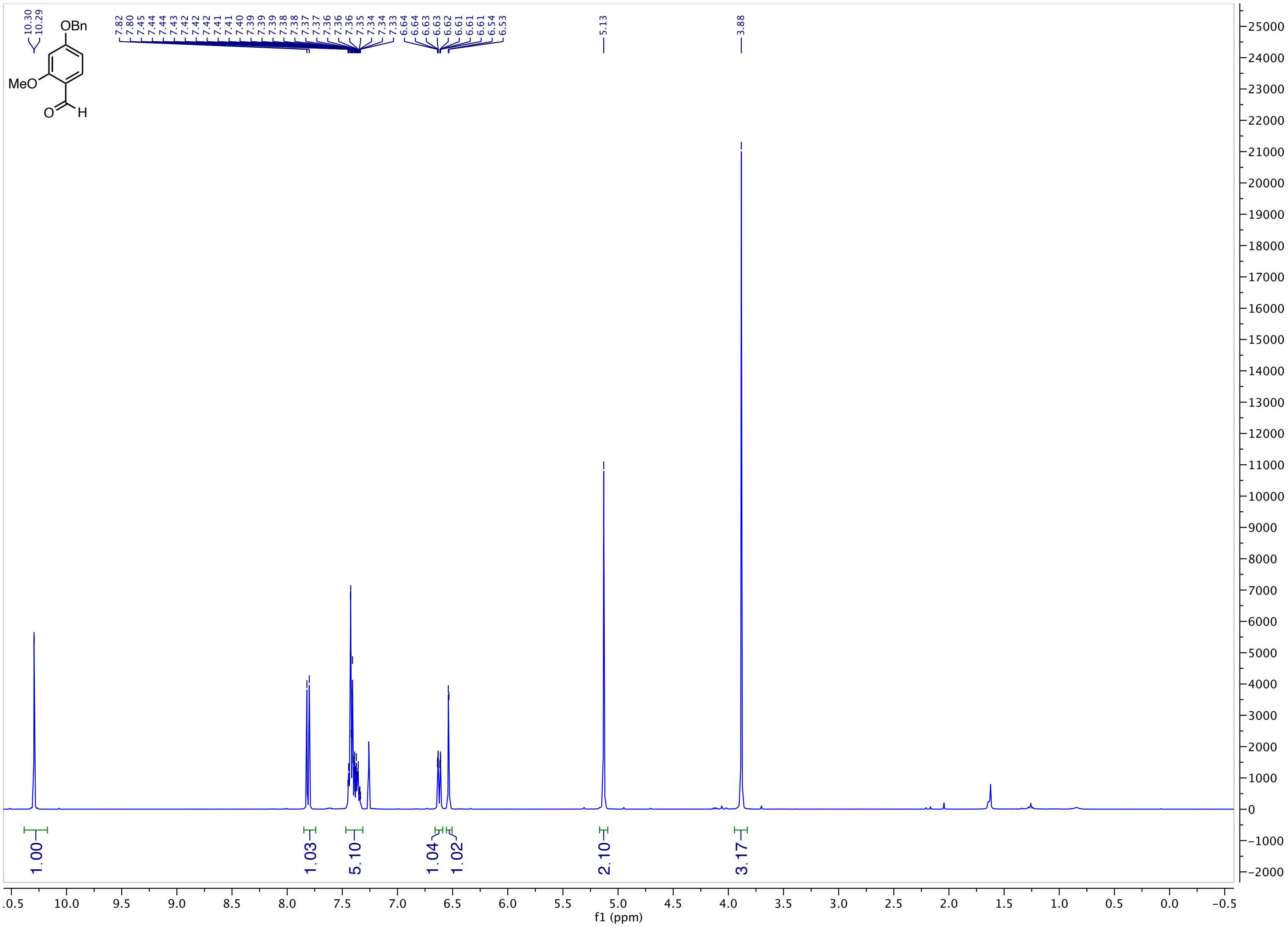

^{13}C NMR ($CDCl_3$): 4-(benzyloxy)-2-Methoxybenzaldehyde **(S4)**

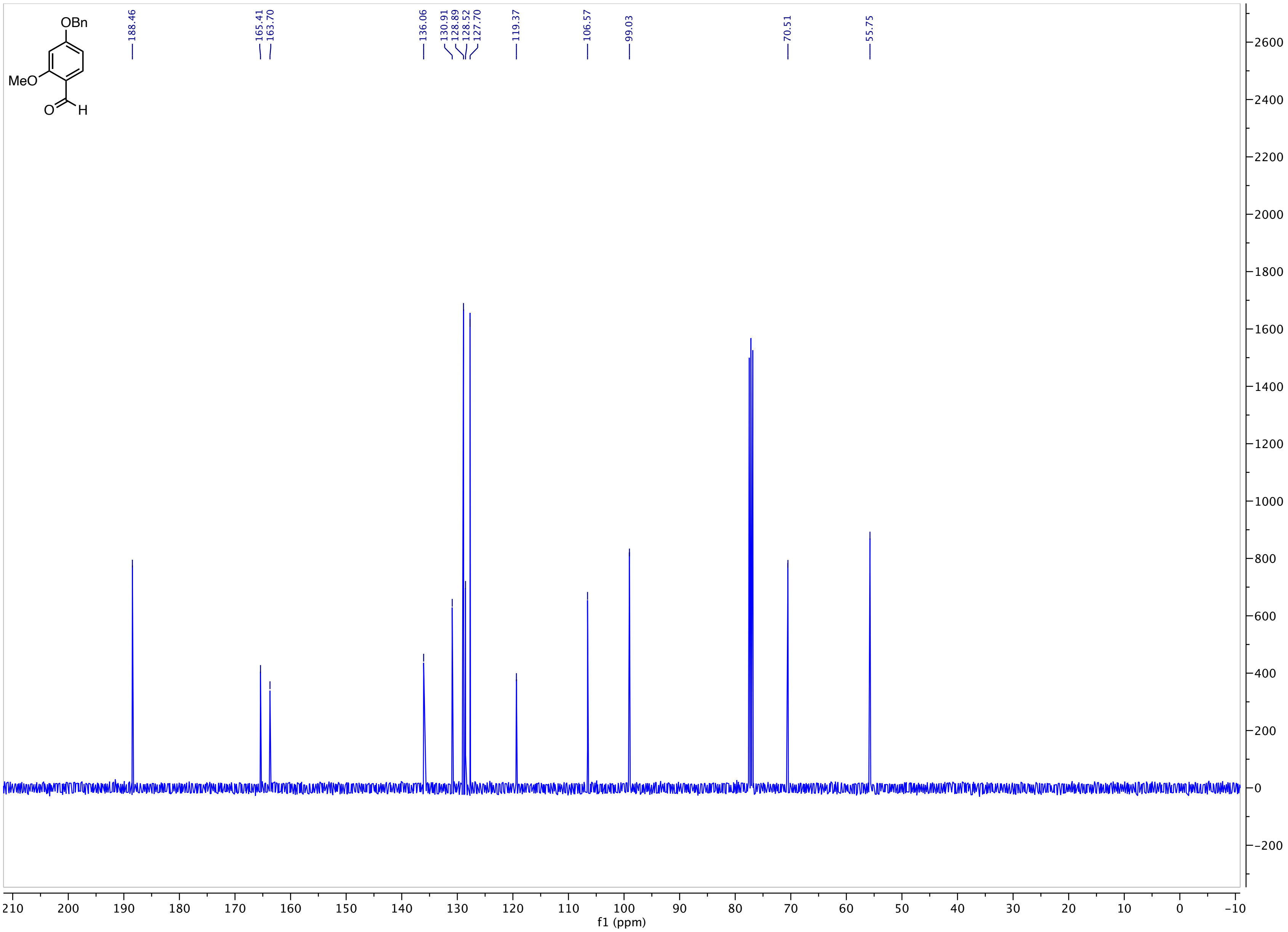

^{1}H NMR ($CDCl_3$): (2-bromobenzyl)Triphenylphosphonium bromide **(S5)**

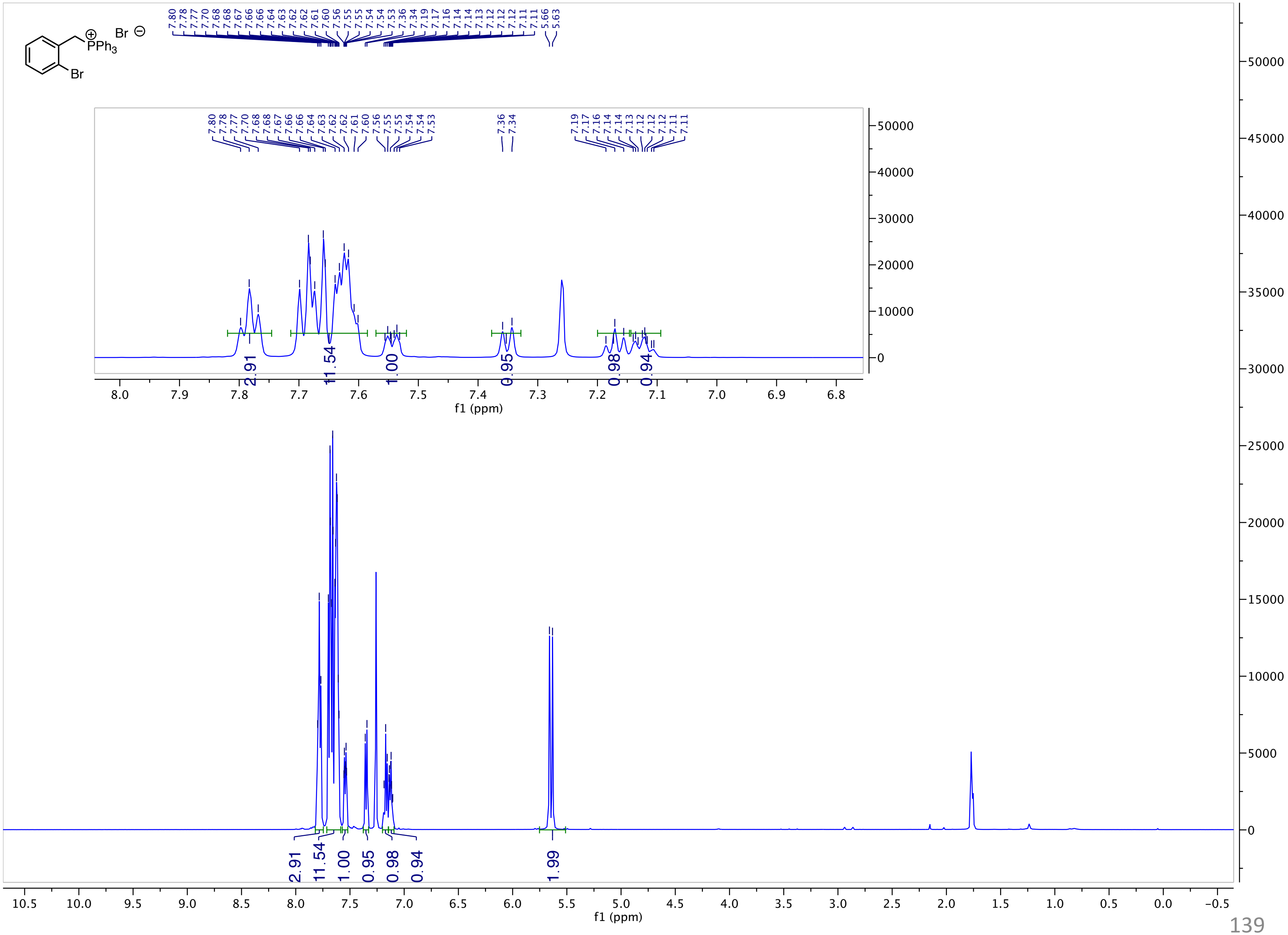

^{13}C NMR ($CDCl_3$): (2-bromobenzyl)Triphenylphosphonium bromide **(S5)**

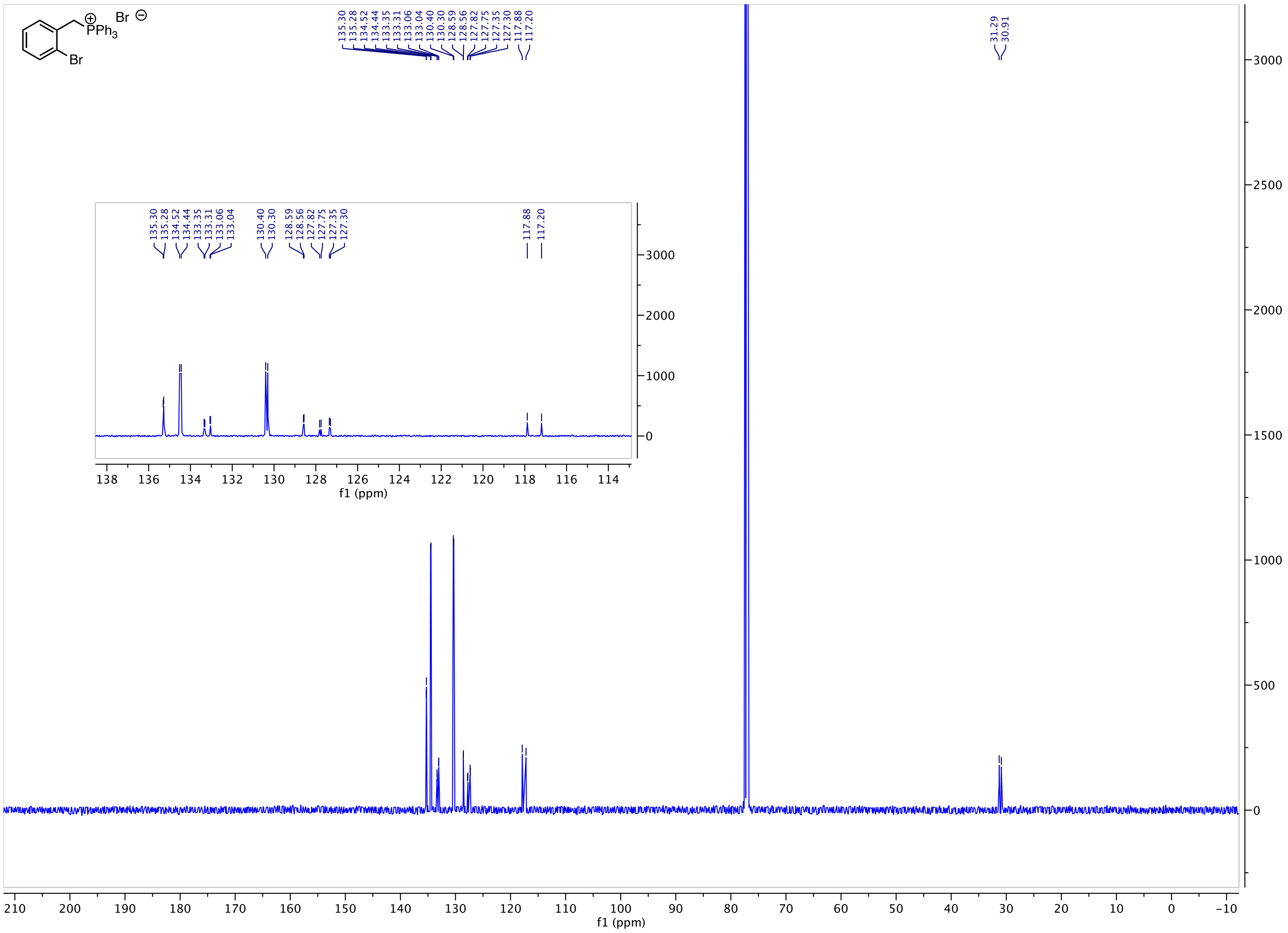

^{1}H NMR ($CDCl_3$): 4-(2-bromophenethyl)-3-Methoxyphenol **(1a)**

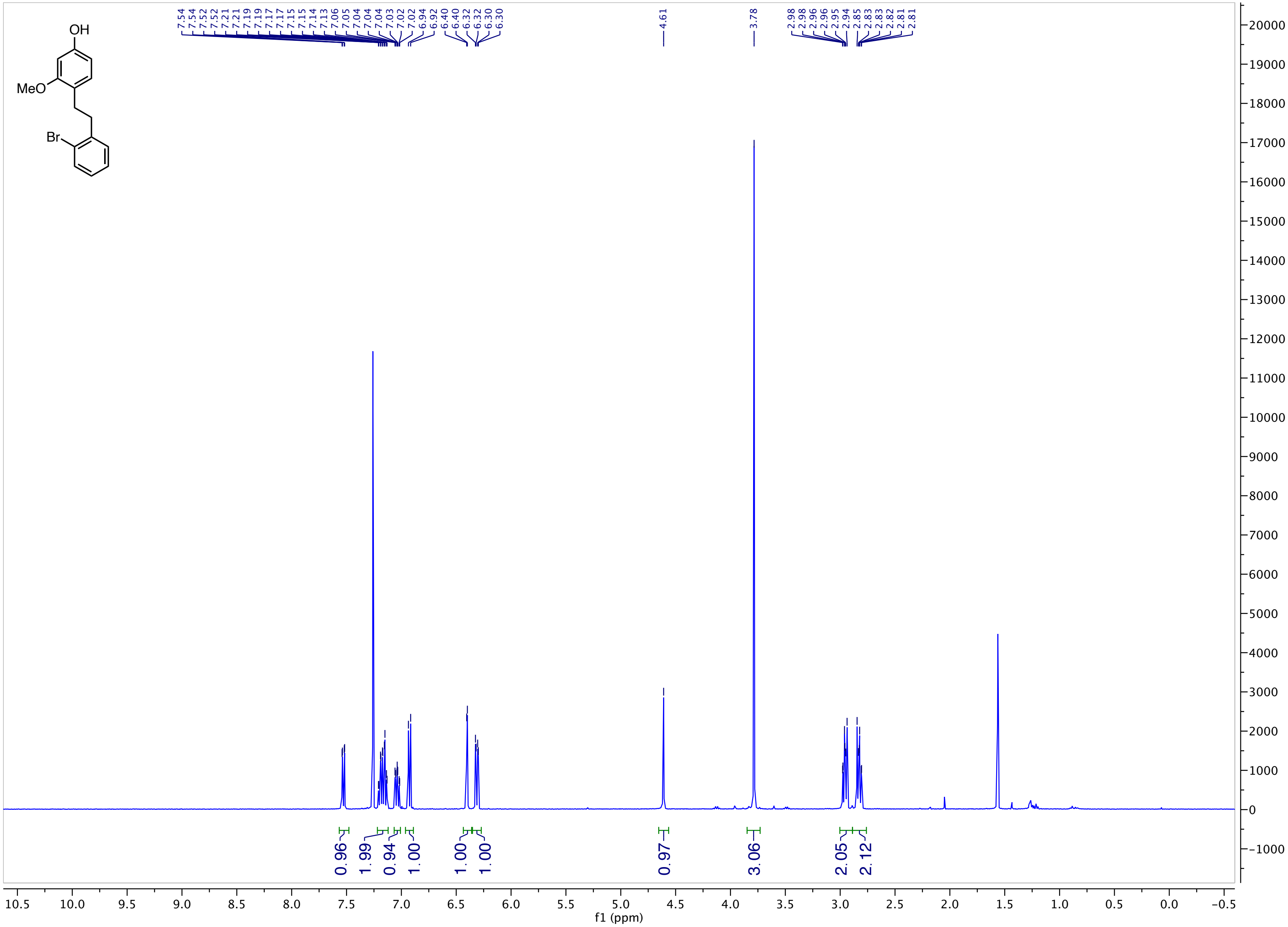

^{13}C NMR ($CDCl_3$): 4-(2-bromophenethyl)-3-Methoxyphenol **(1a)**

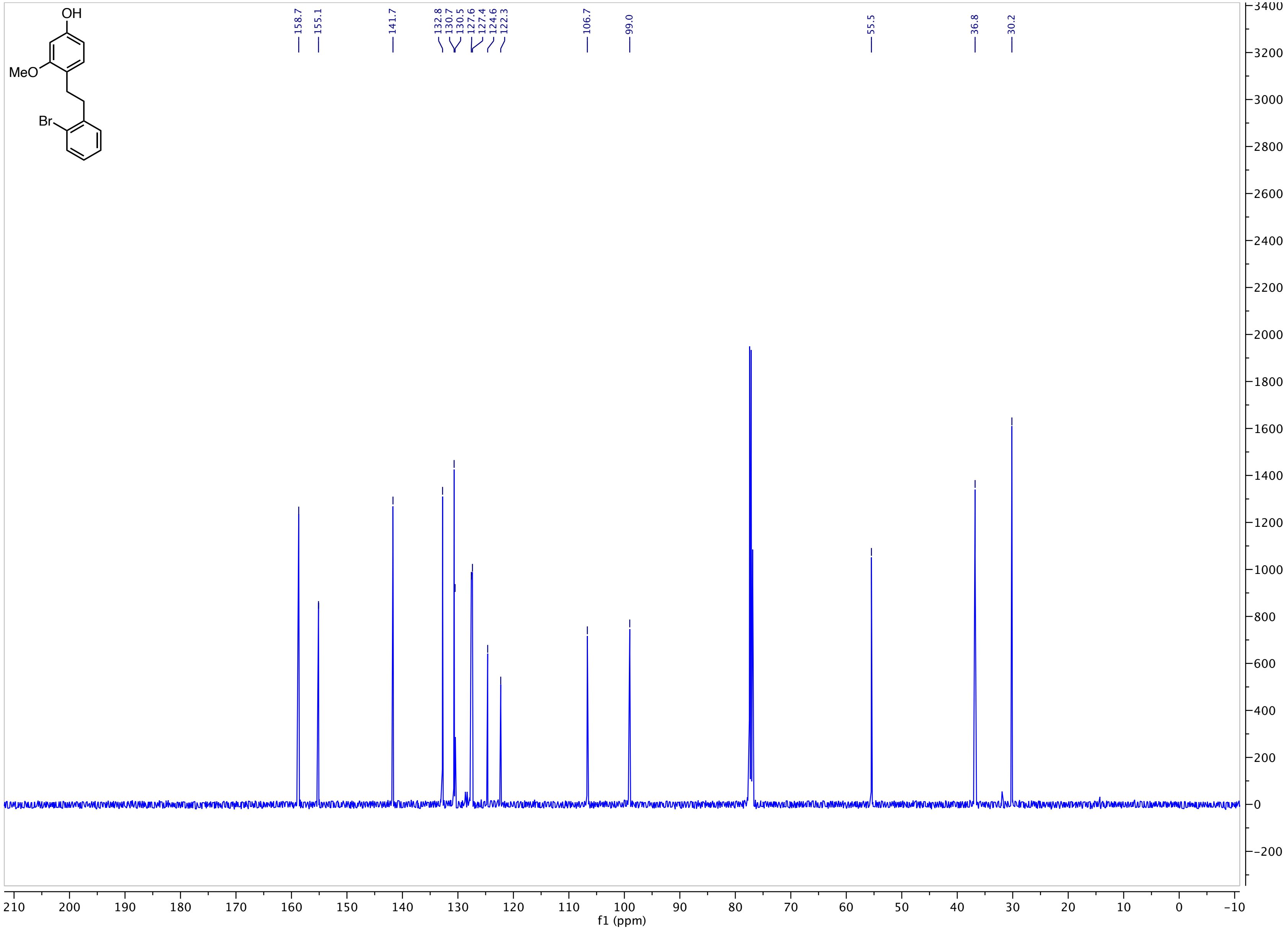

^{1}H NMR ($CDCl_3$): 4-(2-bromophenethyl)-3-Methylphenol **(1b)**

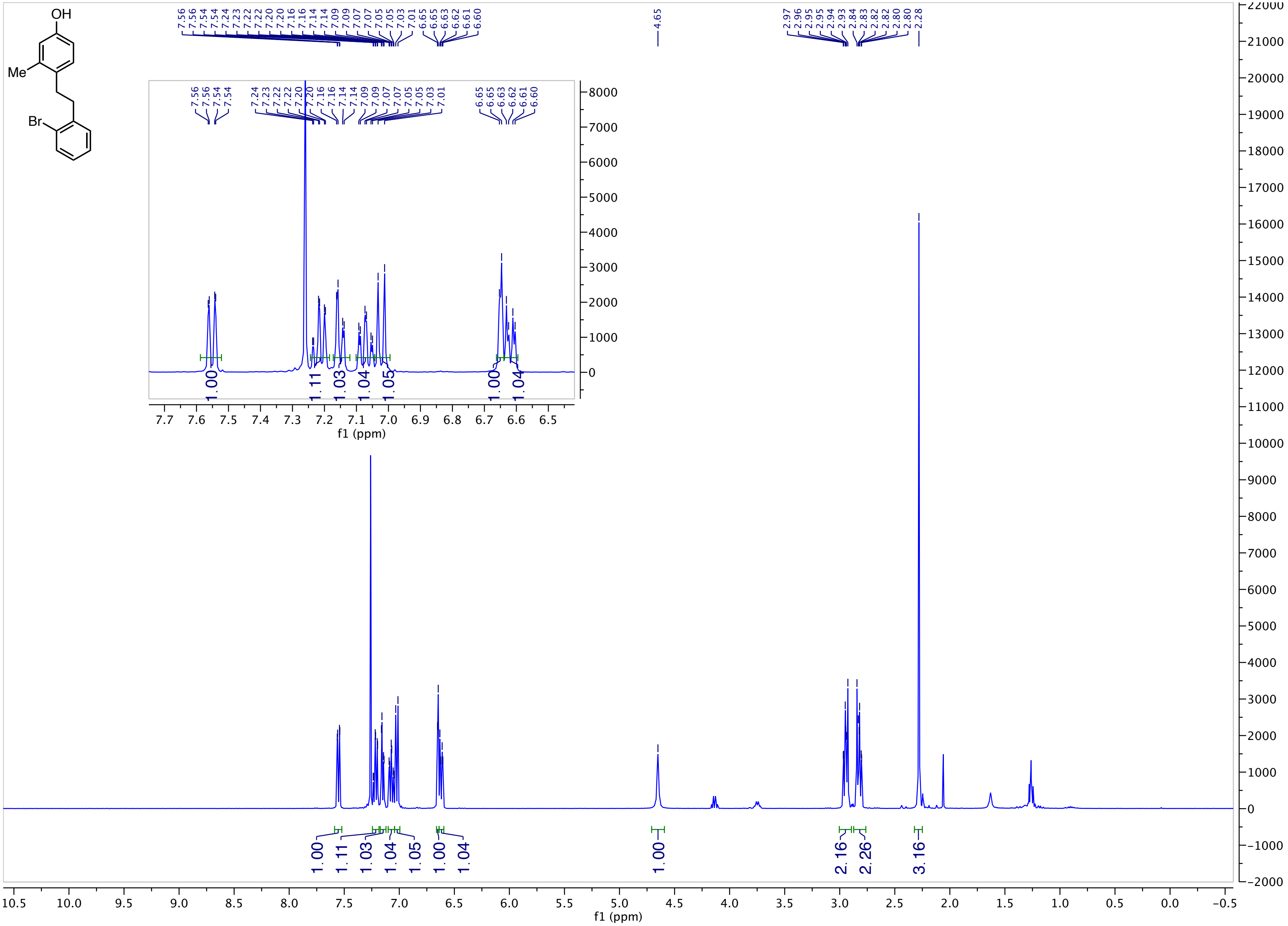

^{13}C NMR ($CDCl_3$): 4-(2-bromophenethyl)-3-Methylphenol (1b)

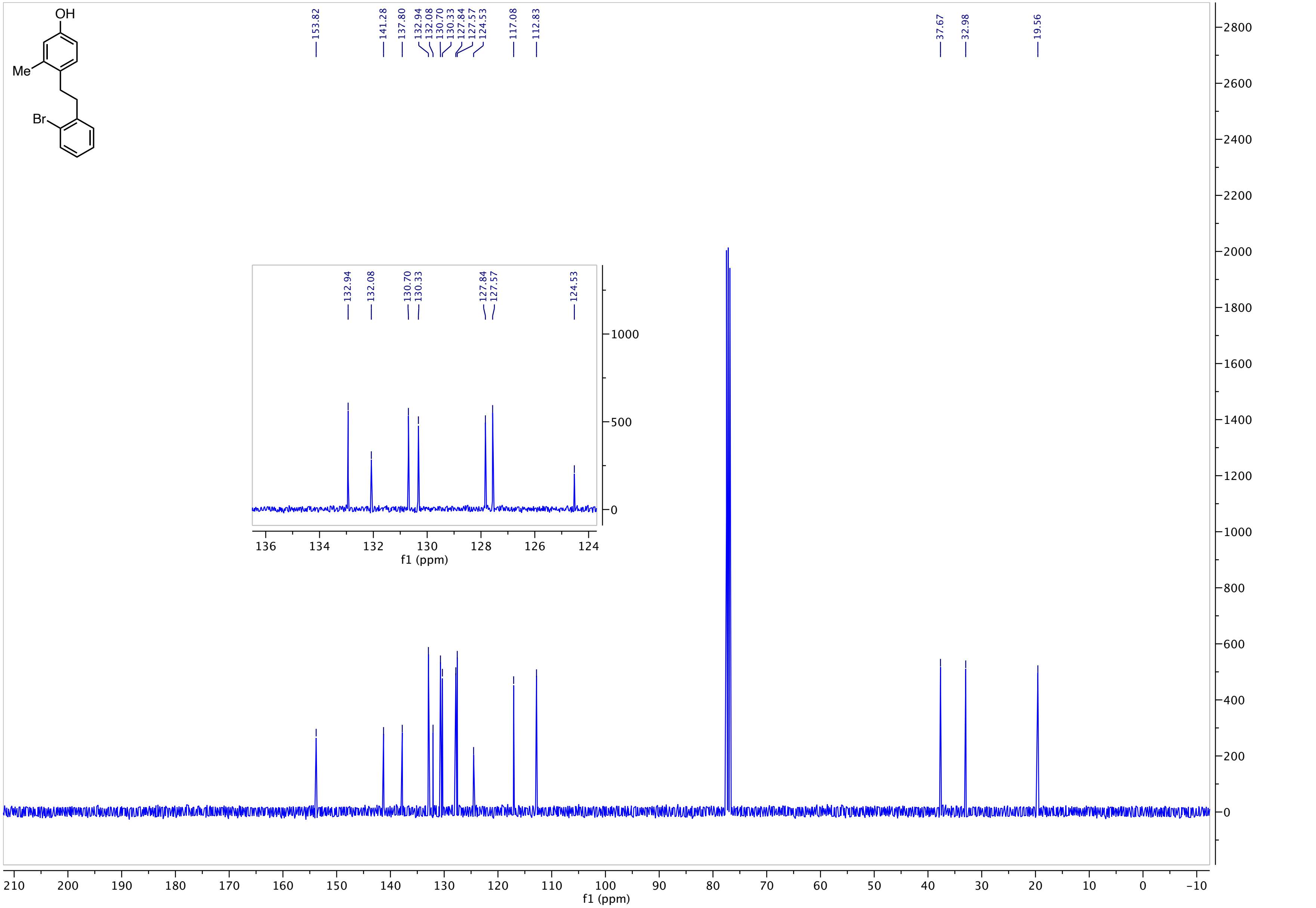

^{1}H NMR ($CDCl_3$): 5-Methoxy-[1,1'-biphenyl]-2-carbaldehyde (S6)

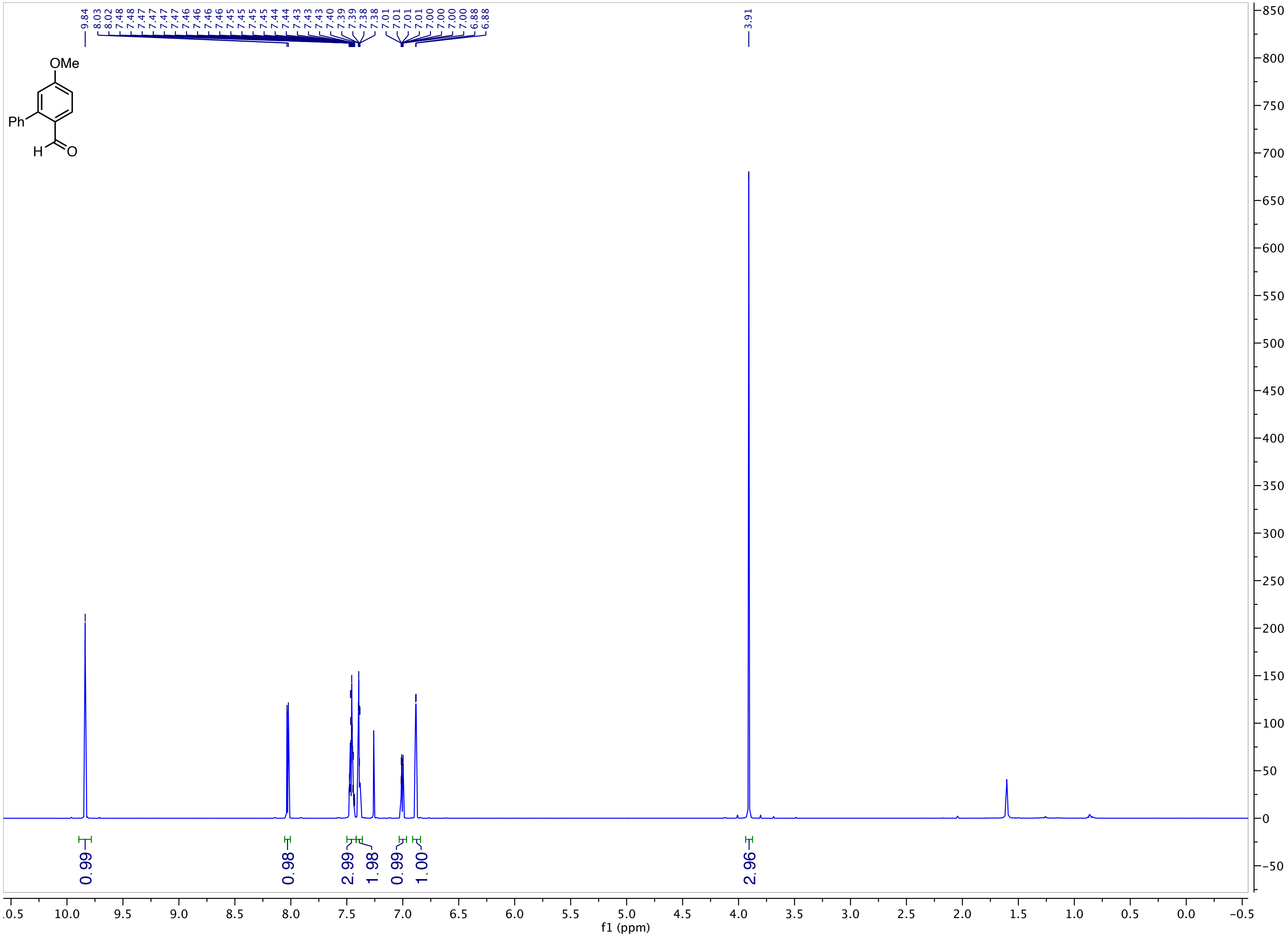

^{13}C NMR ($CDCl_3$): 5-Methoxy-[1,1'-biphenyl]-2-carbaldehyde (S6)

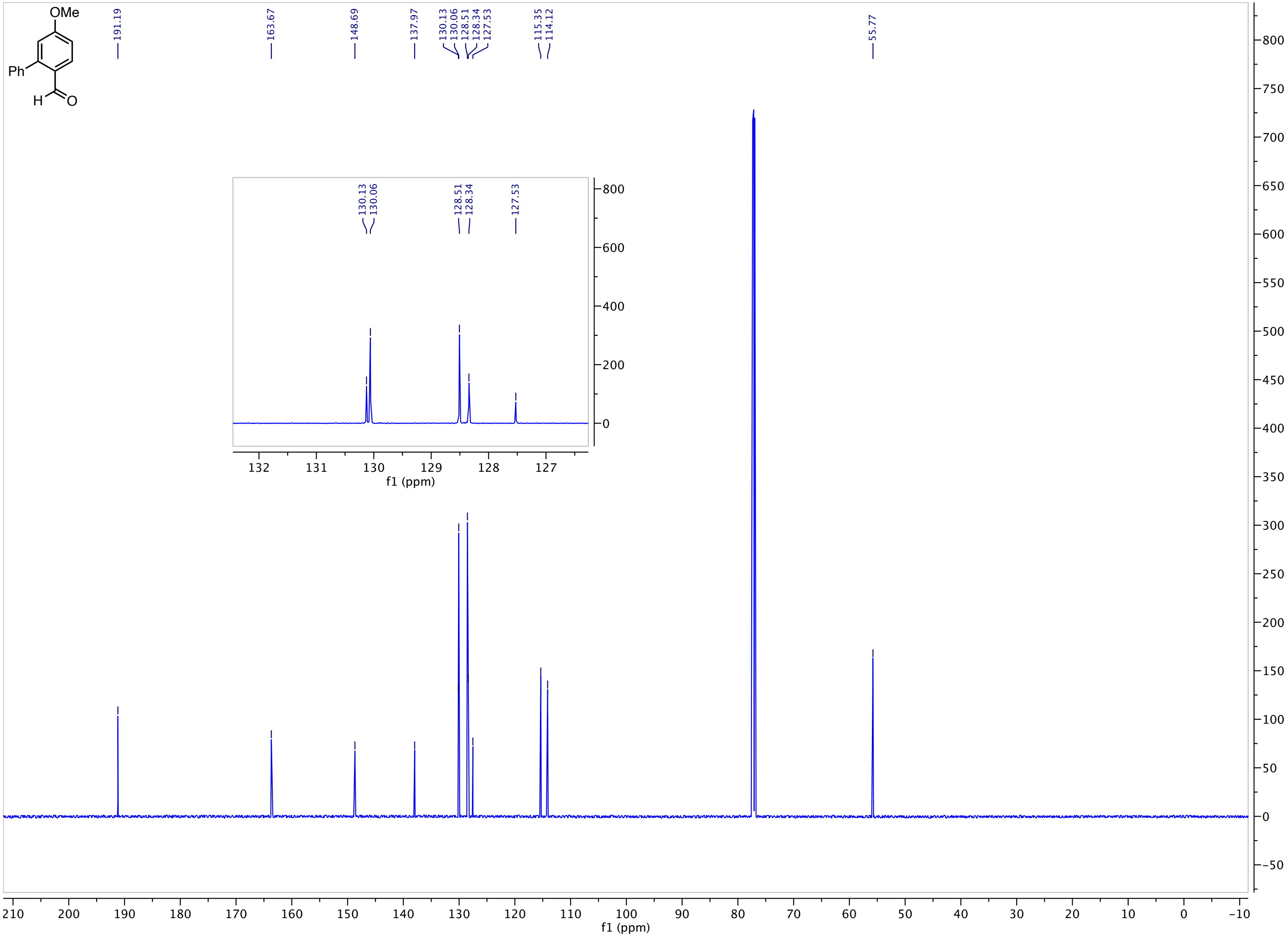

^{1}H NMR ($CDCl_3$): 6-(2-bromophenethyl)-[1,1'-biphenyl]-3-ol (1c)

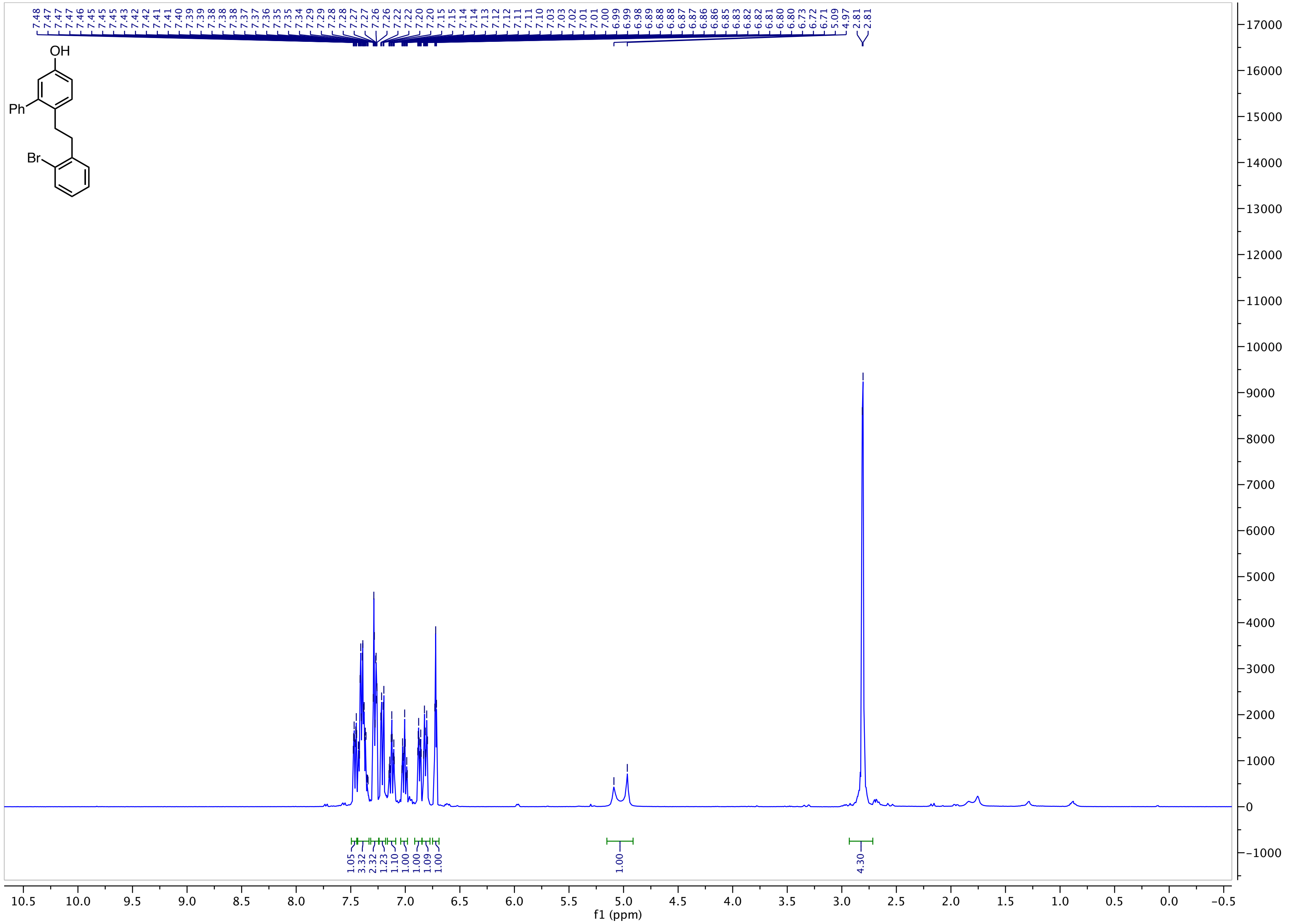

^{13}C NMR ($CDCl_3$): 6-(2-bromophenethyl)-[1,1'-biphenyl]-3-ol **(1c)**

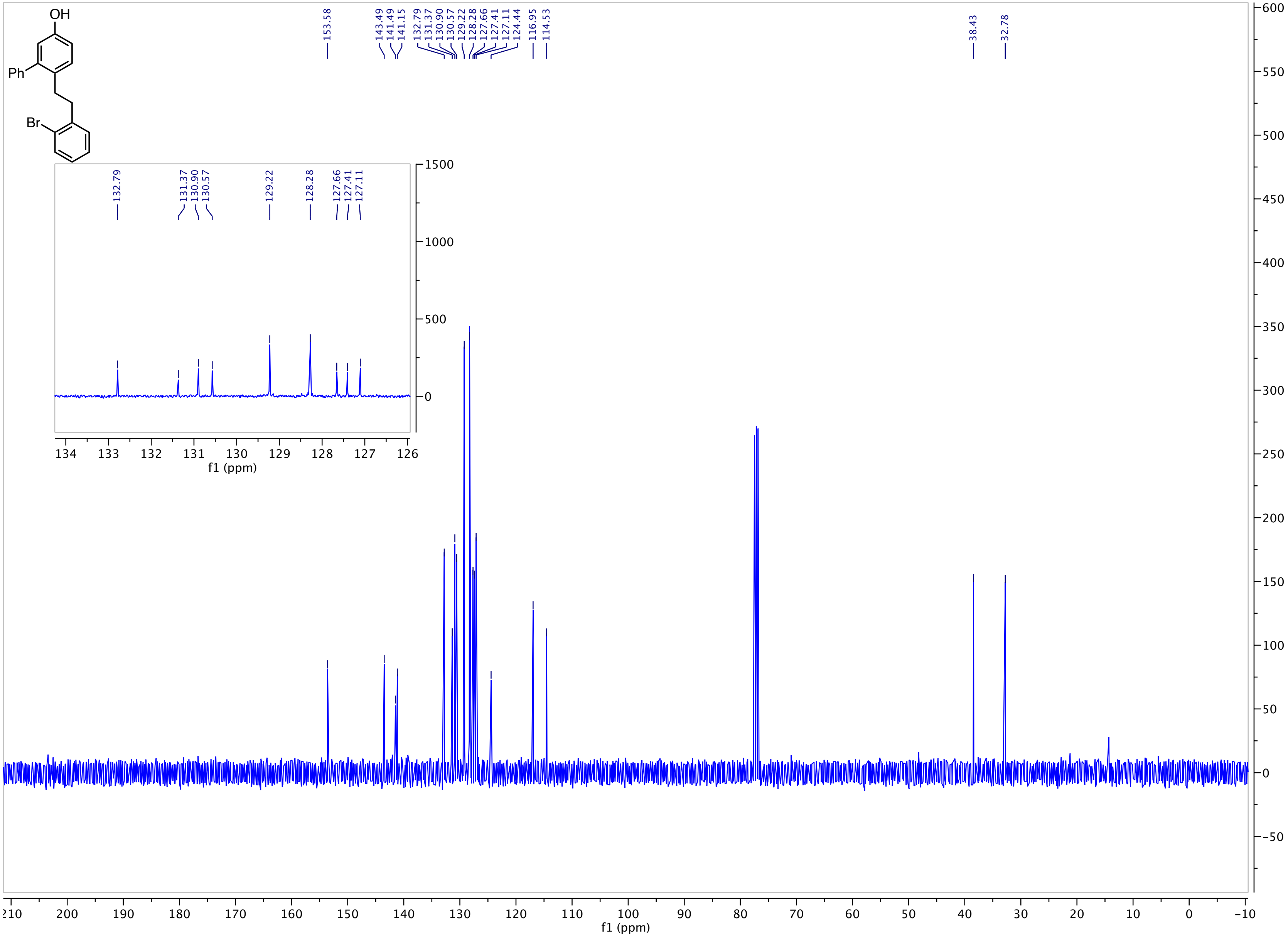

^{1}H NMR ($CDCl_3$): 6-Methoxy-[1,1'-biphenyl]-3-carbaldehyde (S7)

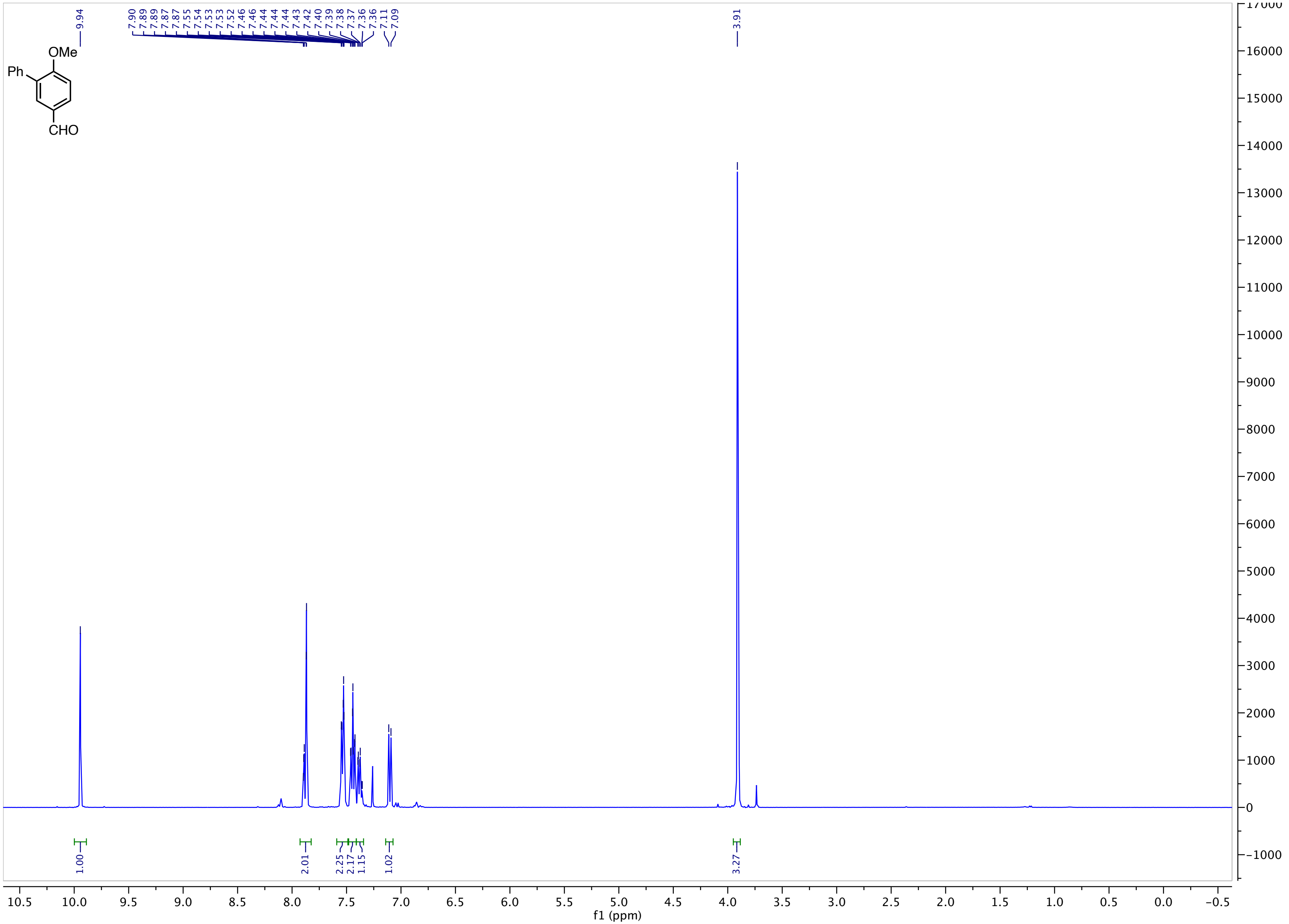

^{13}**C NMR** ($CDCl_3$): 6-Methoxy-[1,1'-biphenyl]-3-carbaldehyde **(S7)**

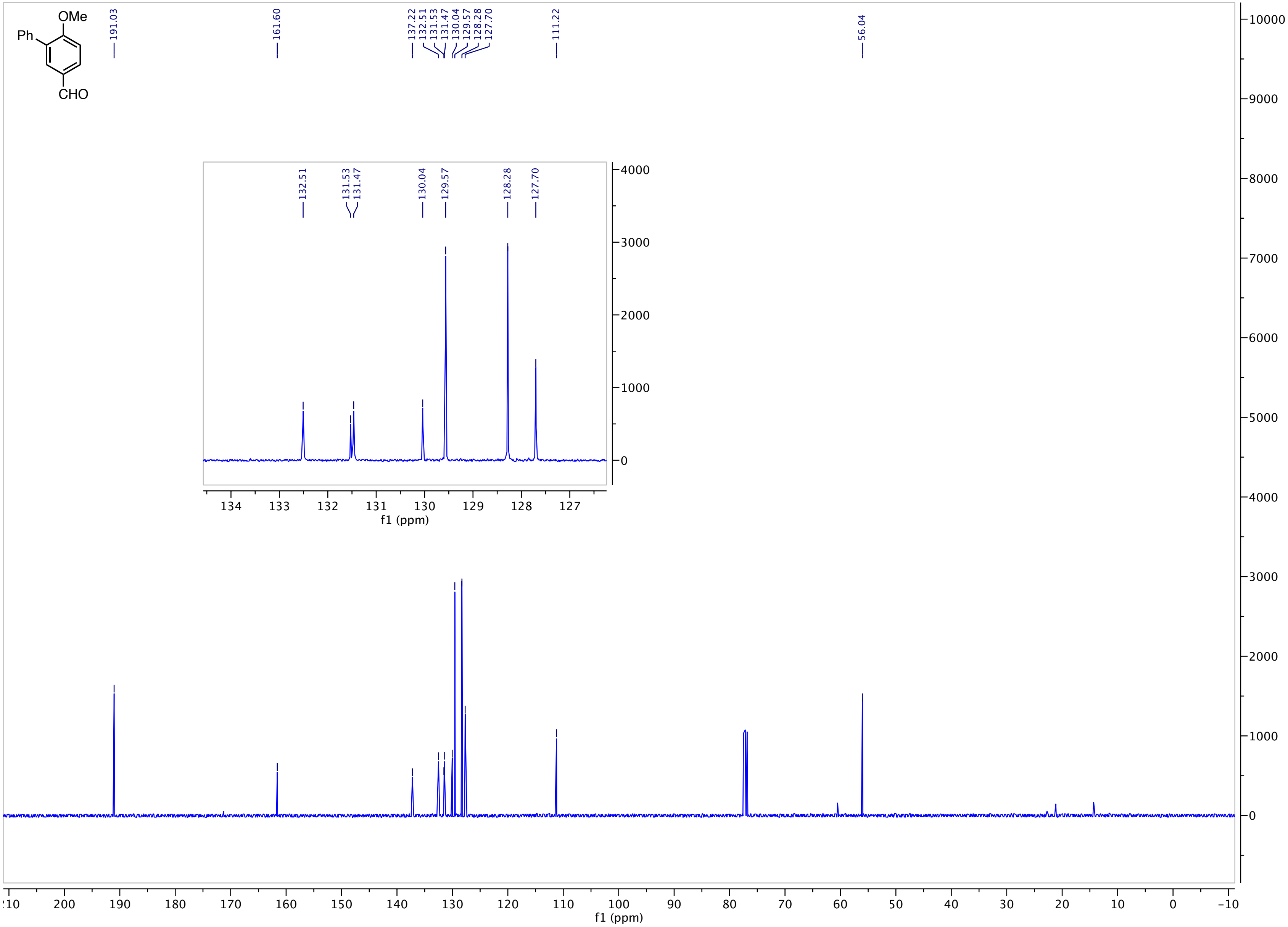

^{1}H NMR ($CDCl_3$): 5-(2-bromophenethyl)-[1,1'-biphenyl]-2-ol (1d)

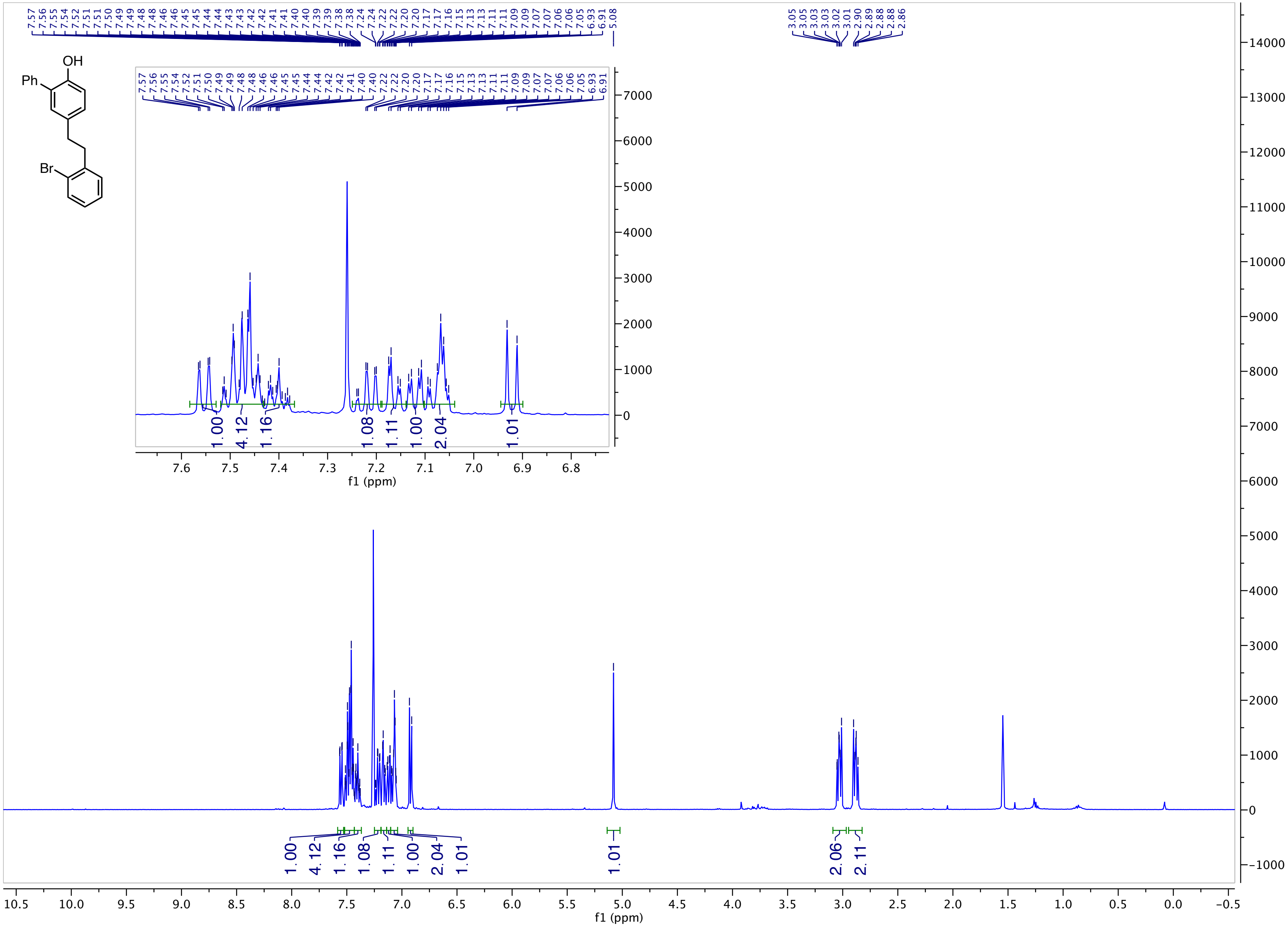

^{13}C NMR ($CDCl_3$): 5-(2-bromophenethyl)-[1,1'-biphenyl]-2-ol **(1d)**

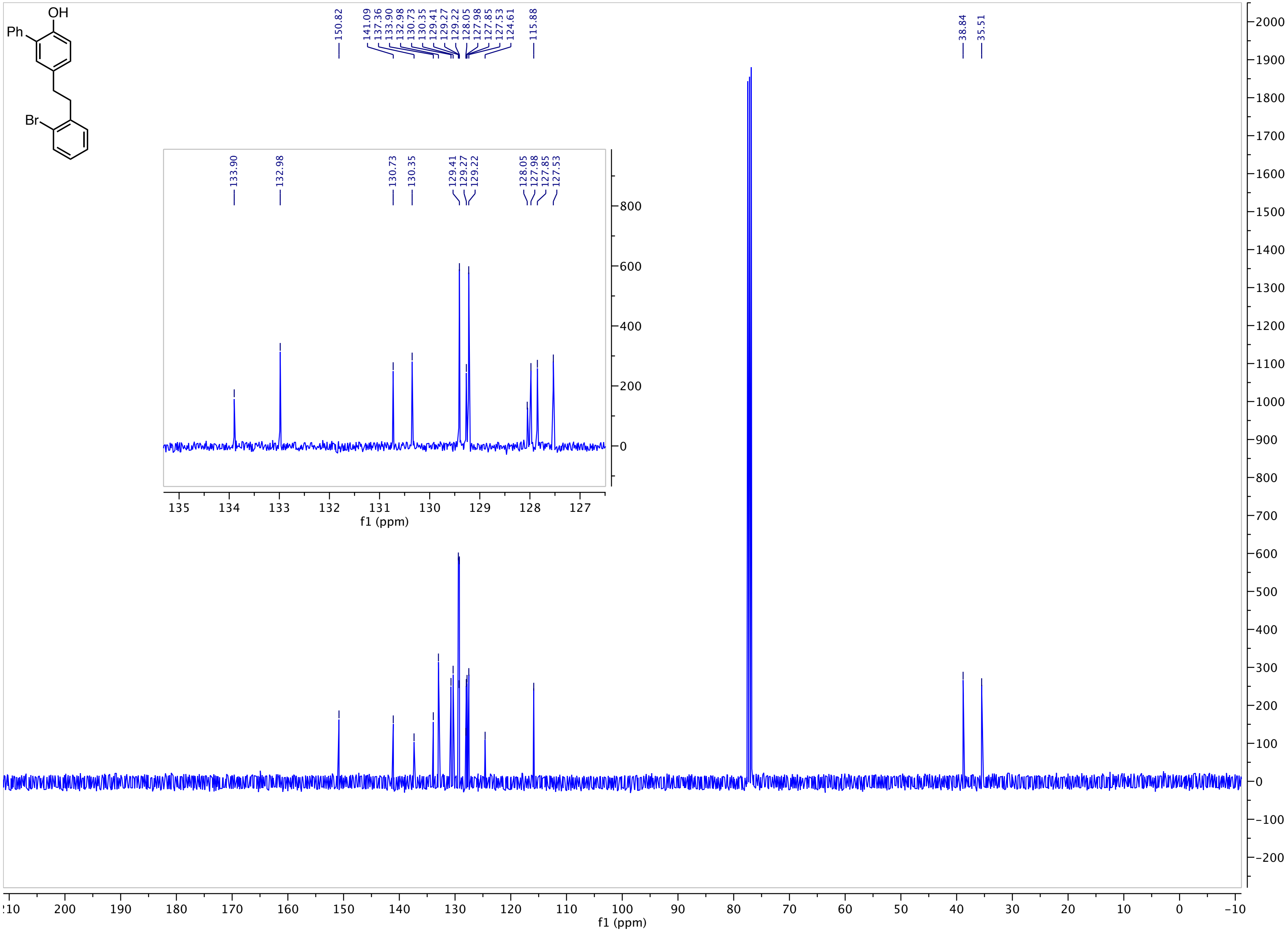

^{1}H NMR ($CDCl_3$): 4-(2-bromophenethyl)-2-Methoxyphenol (1e)

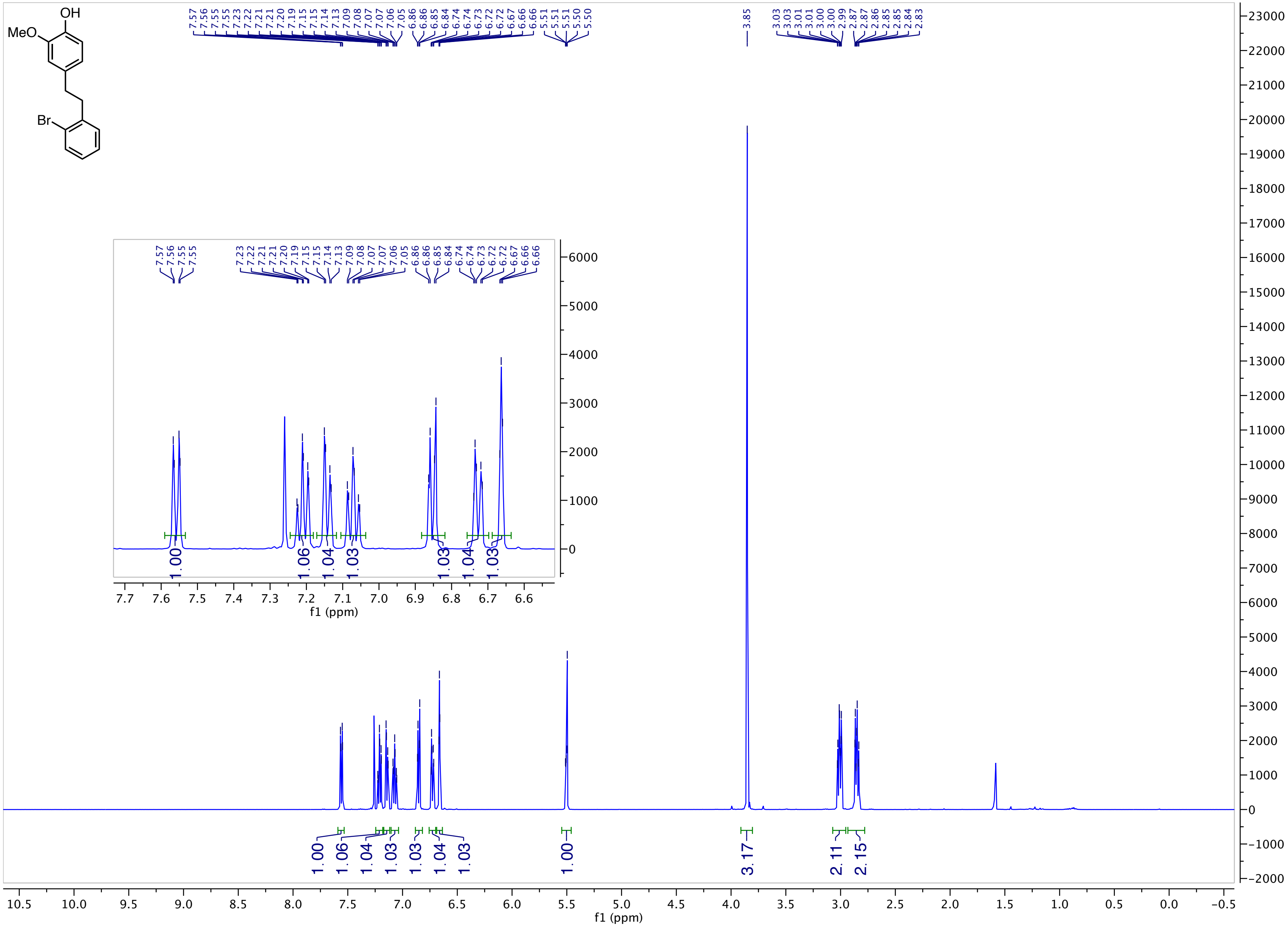

^{13}C NMR ($CDCl_3$): 4-(2-bromophenethyl)-2-Methoxyphenol (1e)

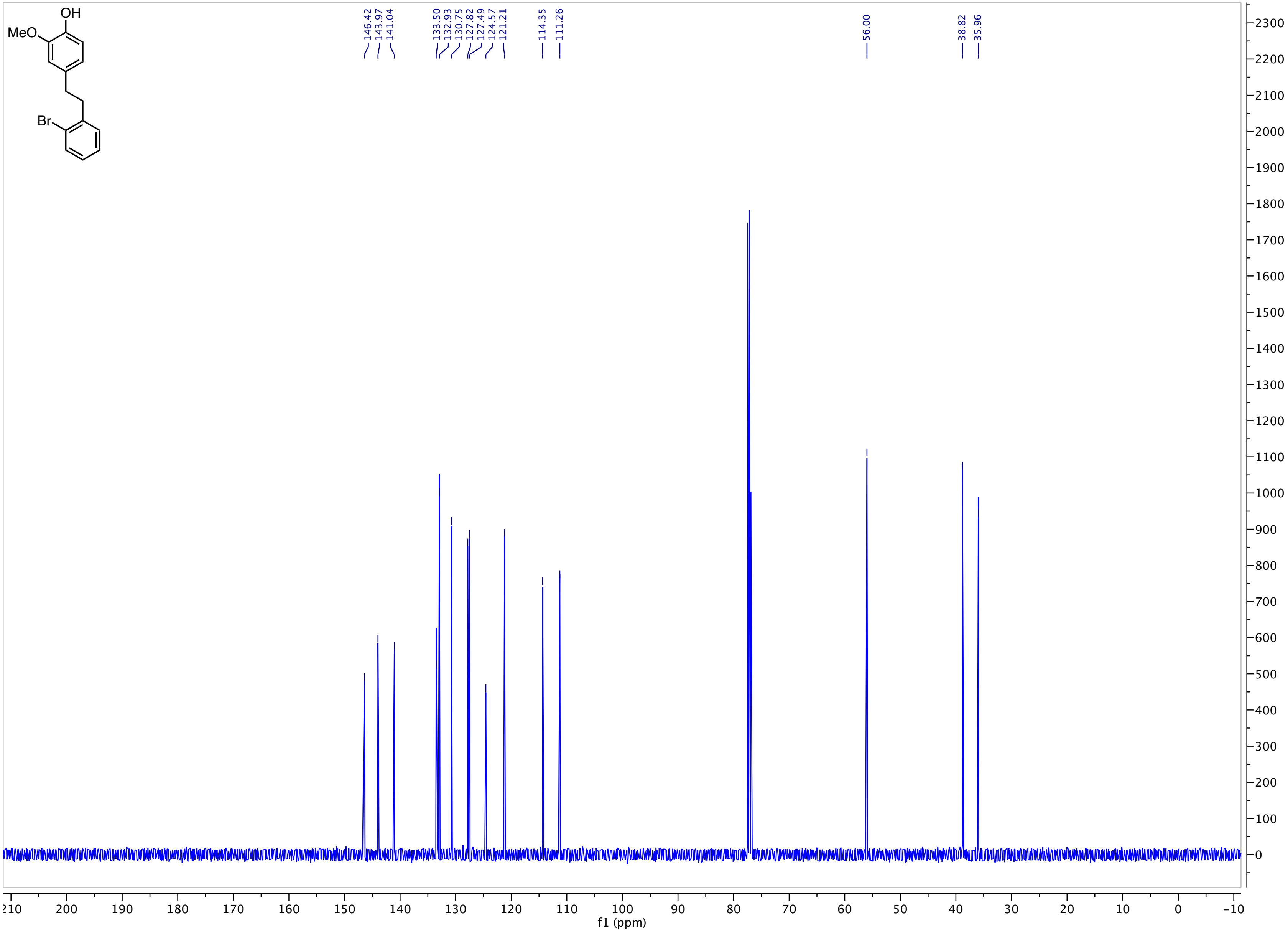

^{1}H NMR ($CDCl_3$): 4-(2-bromophenethyl)-2-Methylphenol (1f)

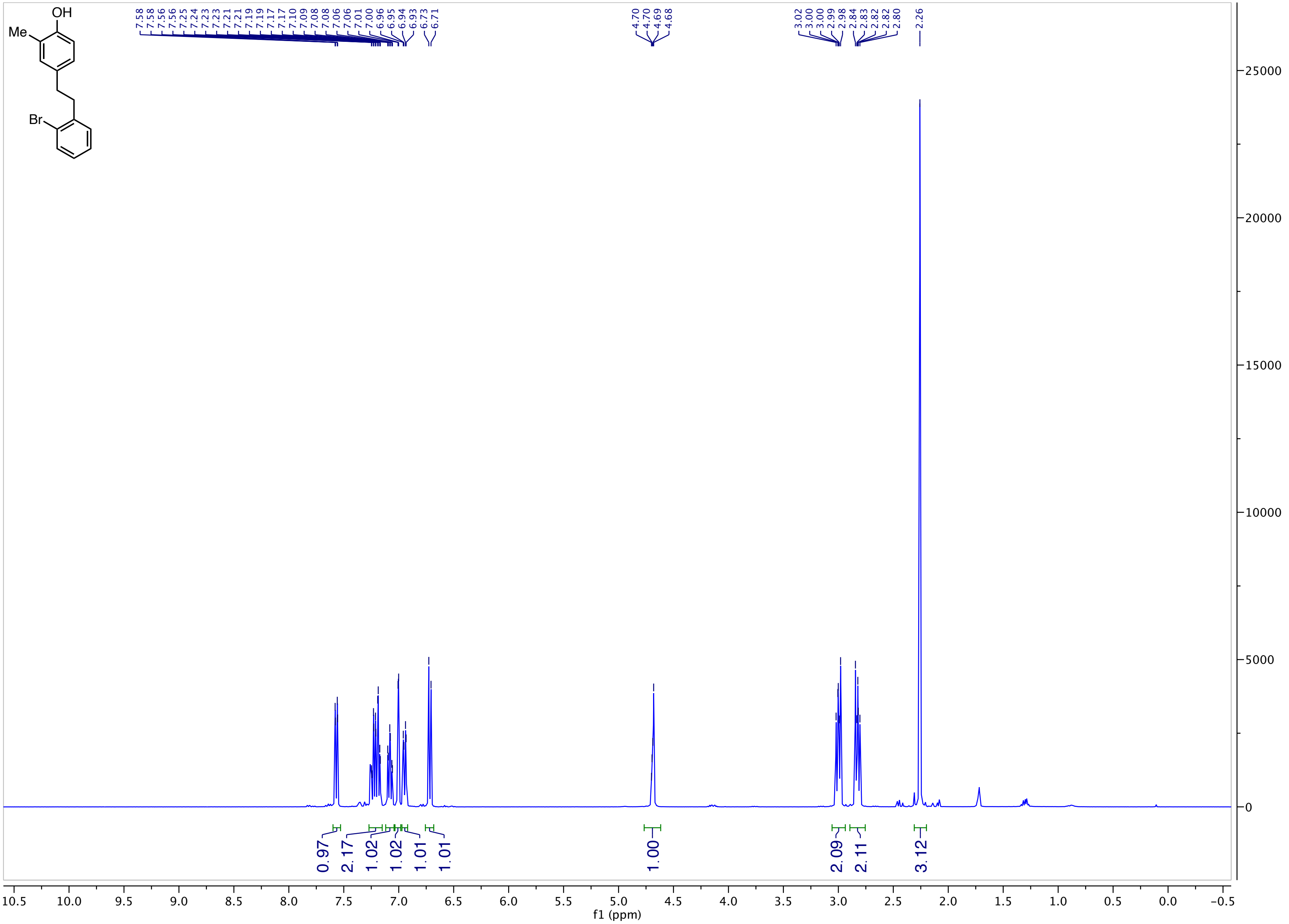

^{13}**C NMR** ($CDCl_3$): 4-(2-bromophenethyl)-2-Methylphenol **(1f)**

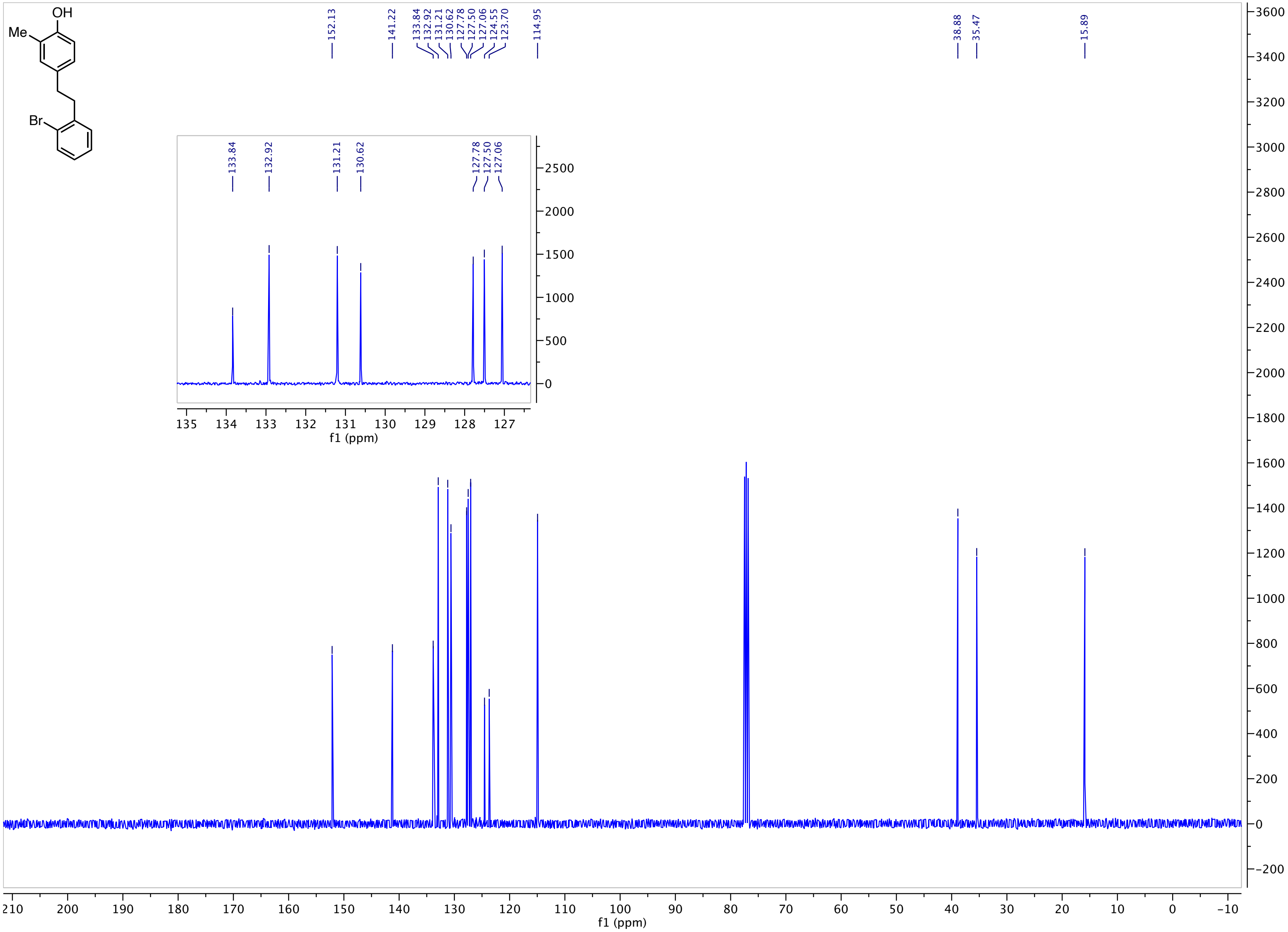

^{1}H NMR ($CDCl_3$): 4-(2-bromophenethyl)-2-Fluorophenol (1g)

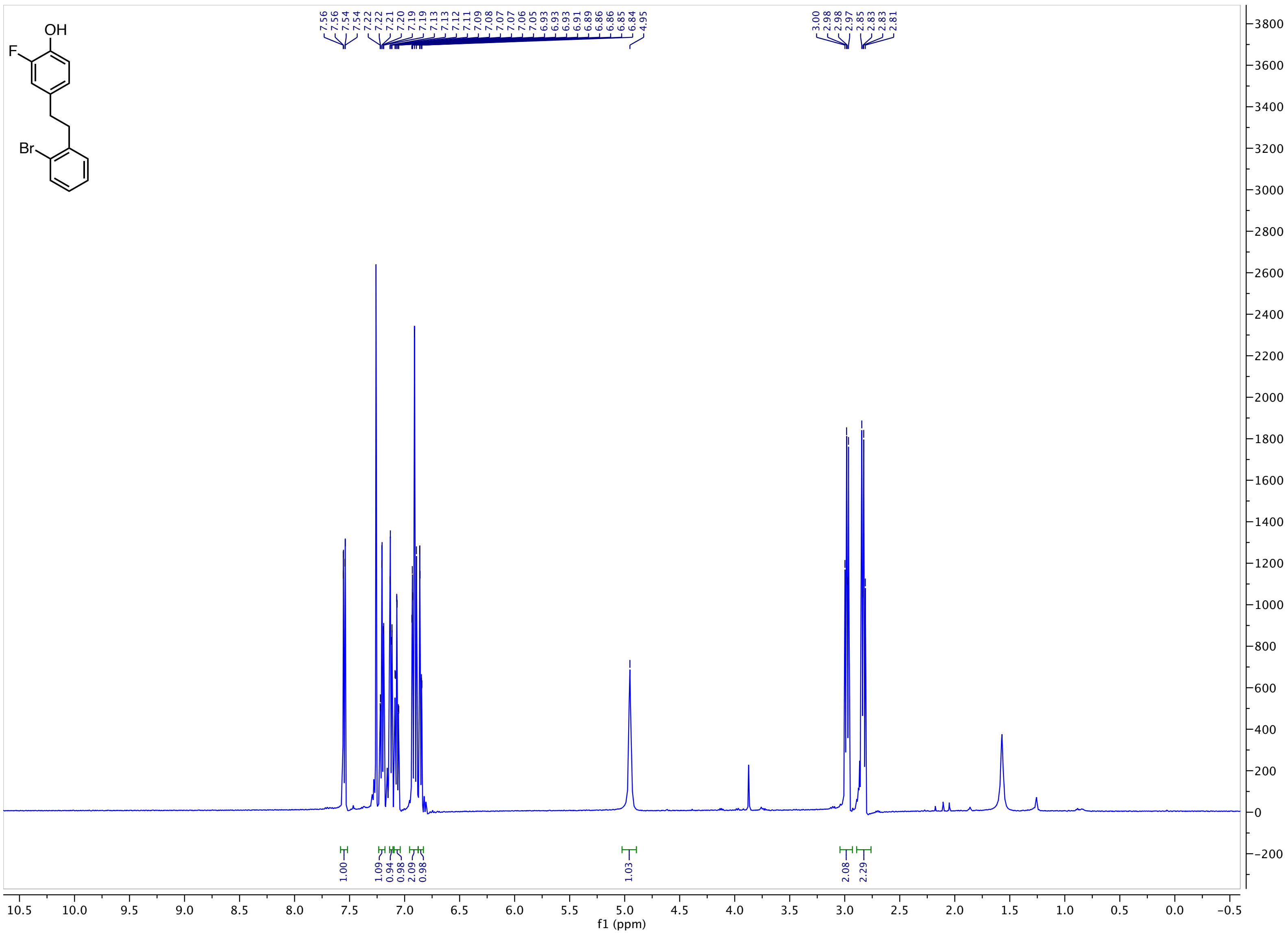

^{13}C NMR ($CDCl_3$): 4-(2-bromophenethyl)-2-Fluorophenol (1g)

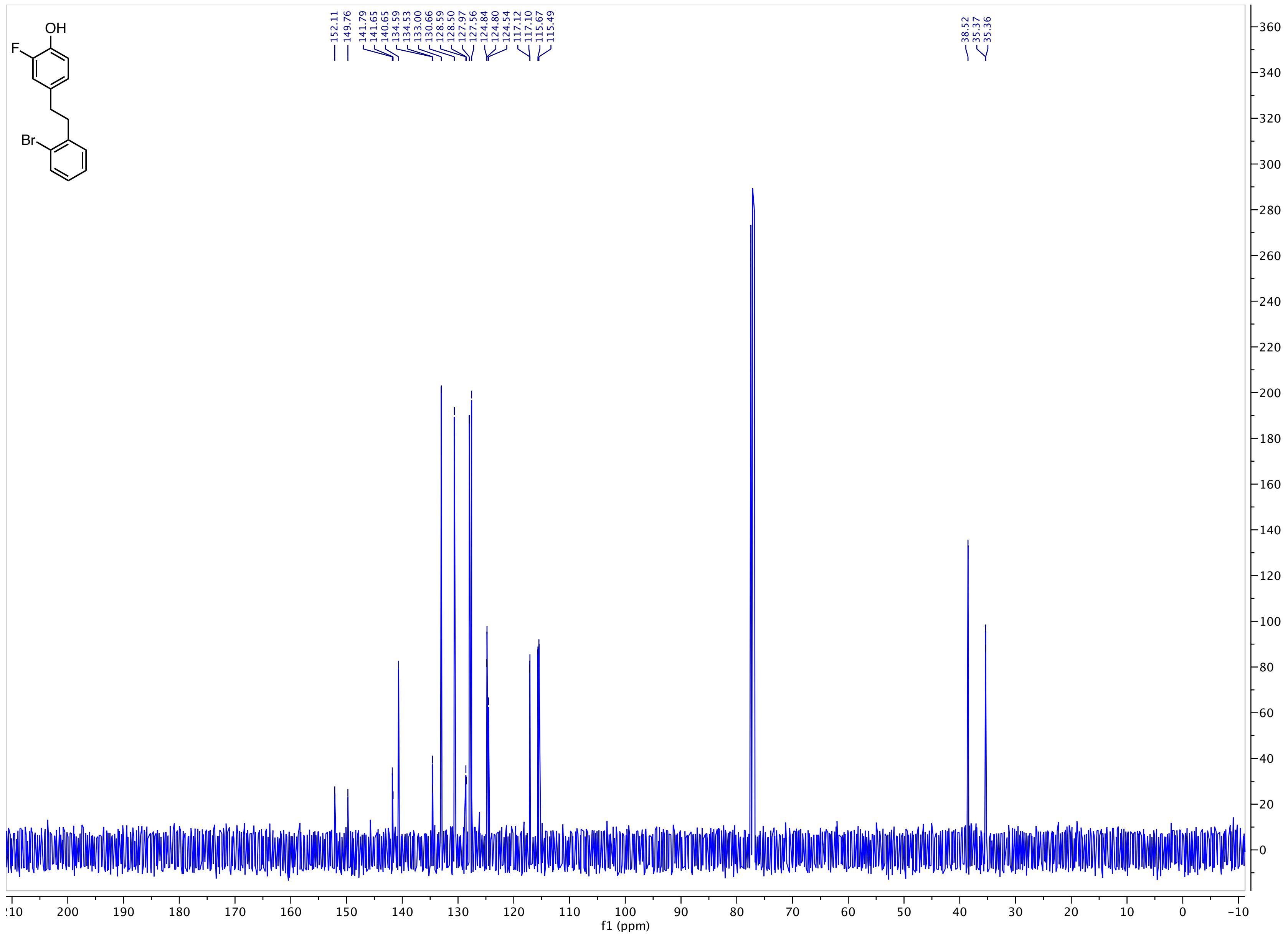

^{19}**F NMR** ($CDCl_3$): 4-(2-bromophenethyl)-2-Fluorophenol (**1g**)

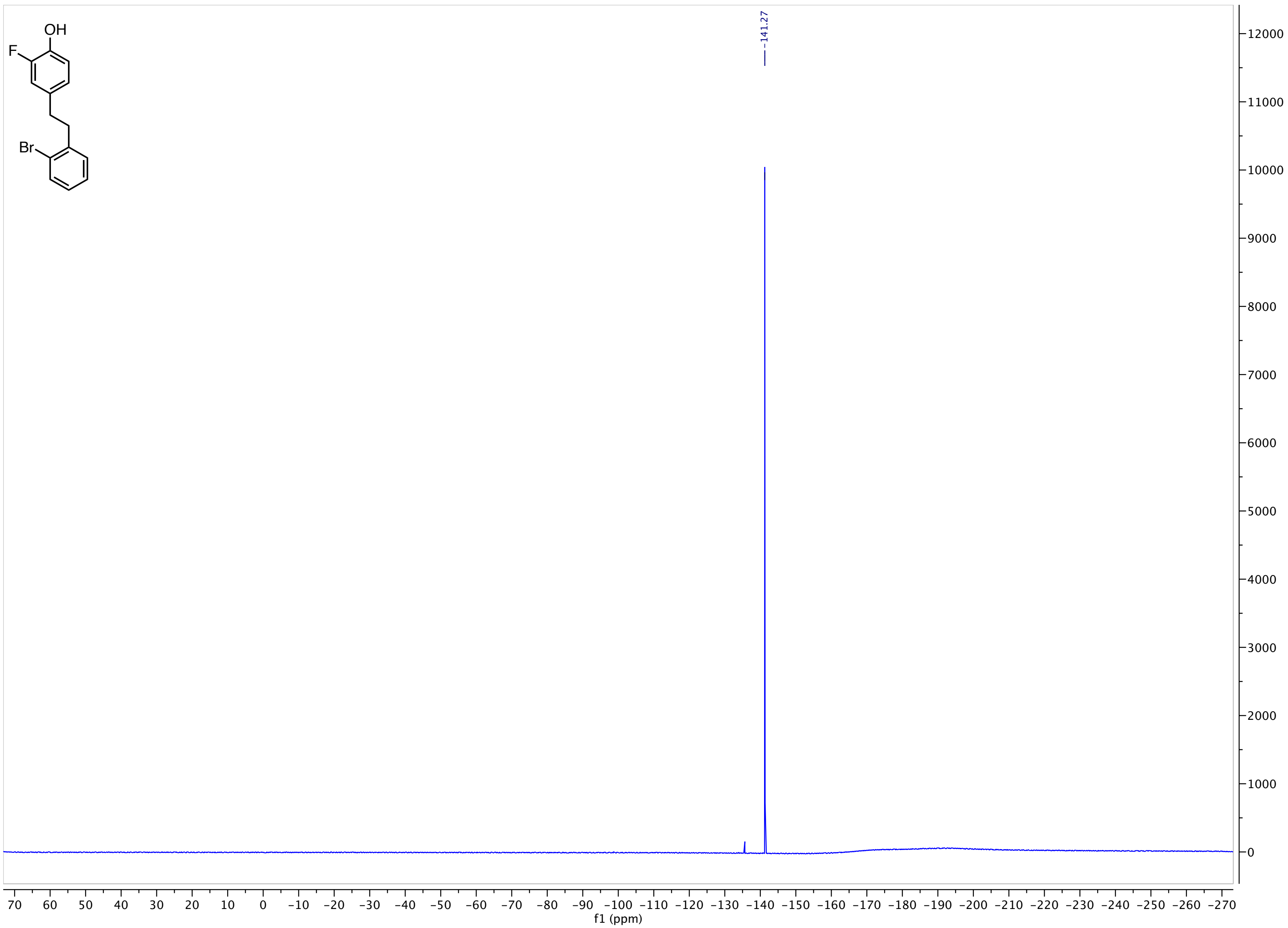

^{1}**H NMR** ($CDCl_3$): 4-(benzyloxy)-1-Naphthaldehyde **(S8)**

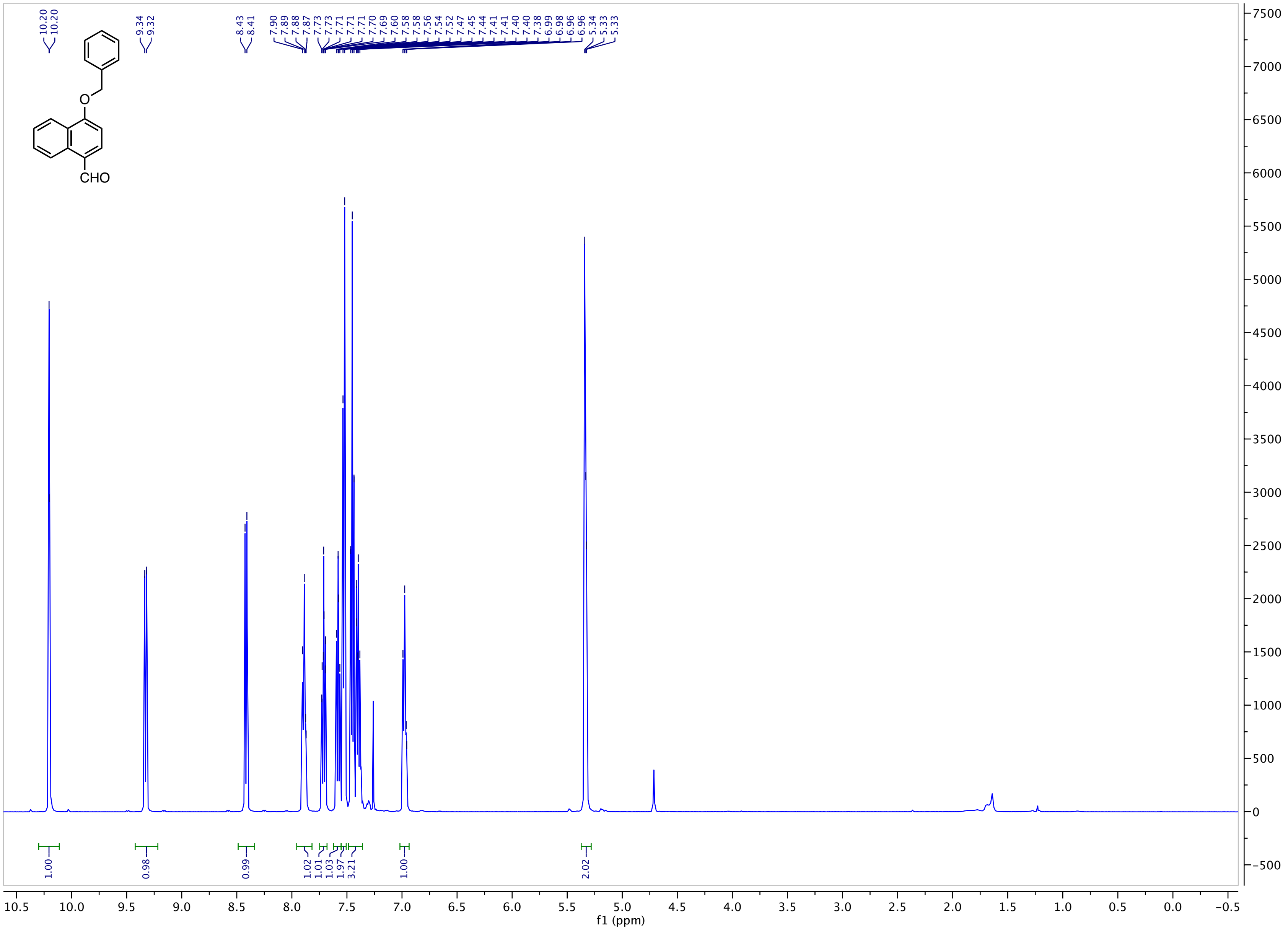

^{13}C NMR ($CDCl_3$): 4-(benzyloxy)-1-Naphthaldehyde (S8)

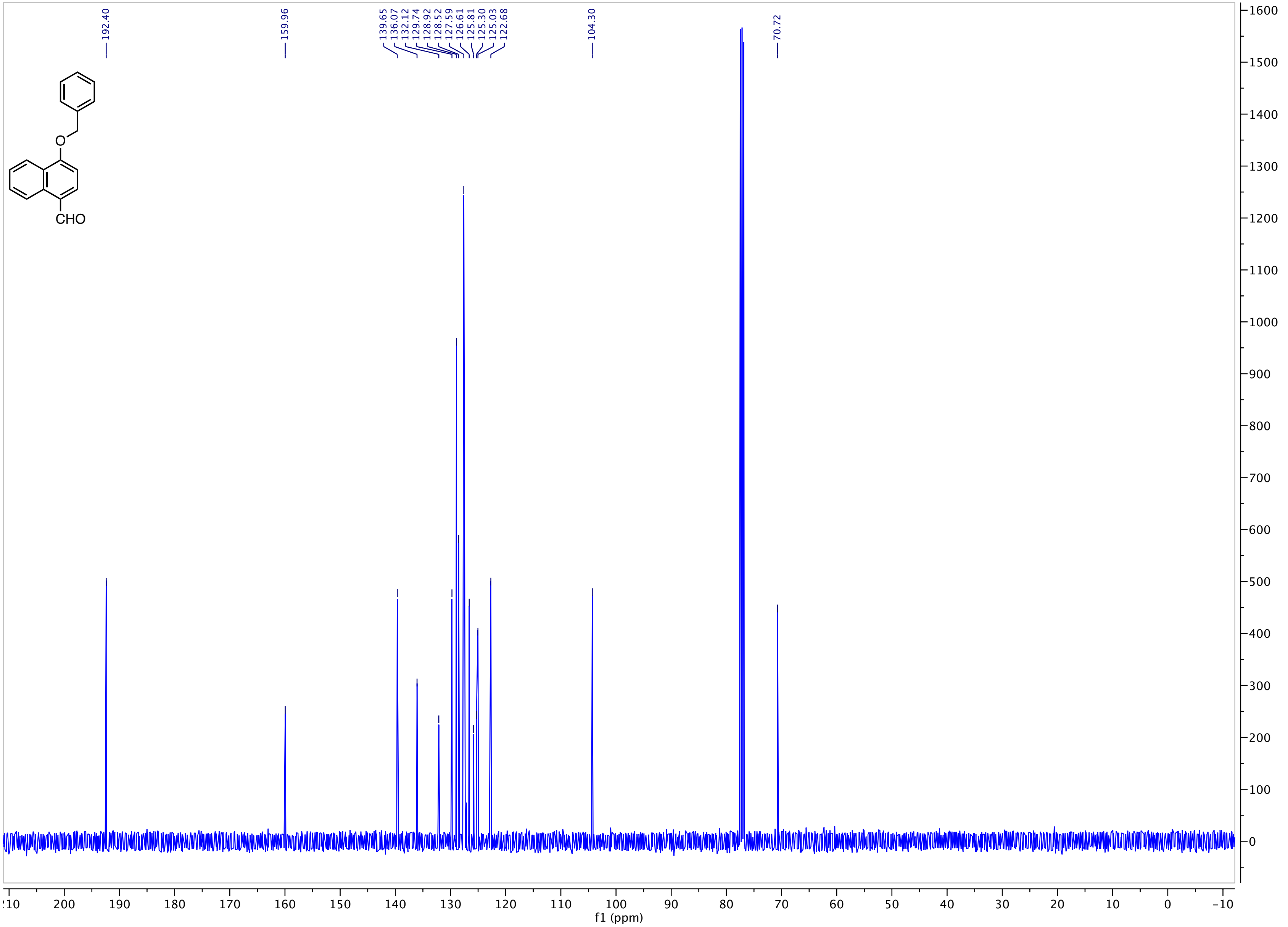

^{13}H NMR ($CDCl_3$): 4-(2-bromophenethyl)Naphthalen-1-ol **(1h)**

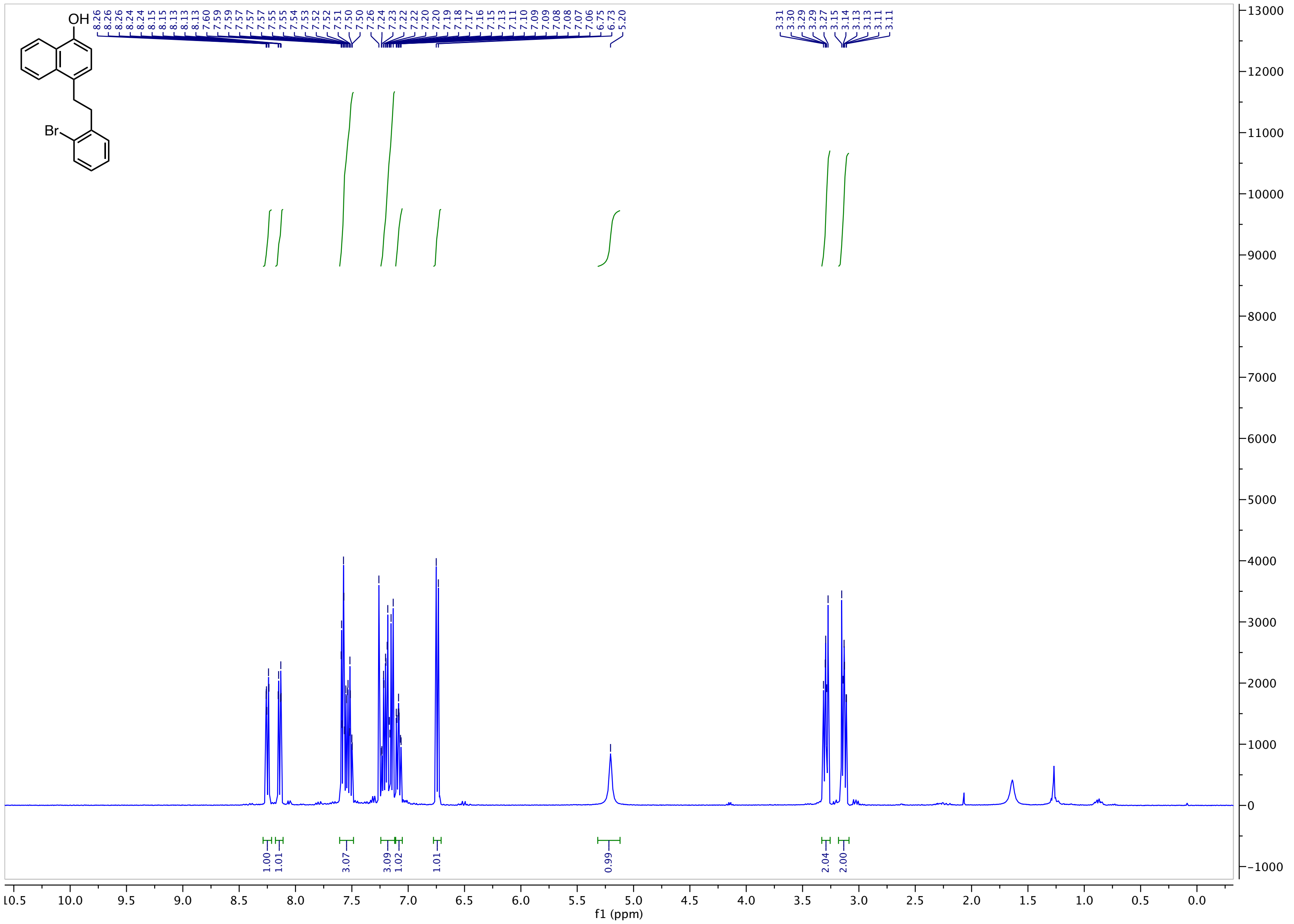

^{13}C NMR ($CDCl_3$): 4-(2-bromophenethyl)Naphthalen-1-ol **(1h)**

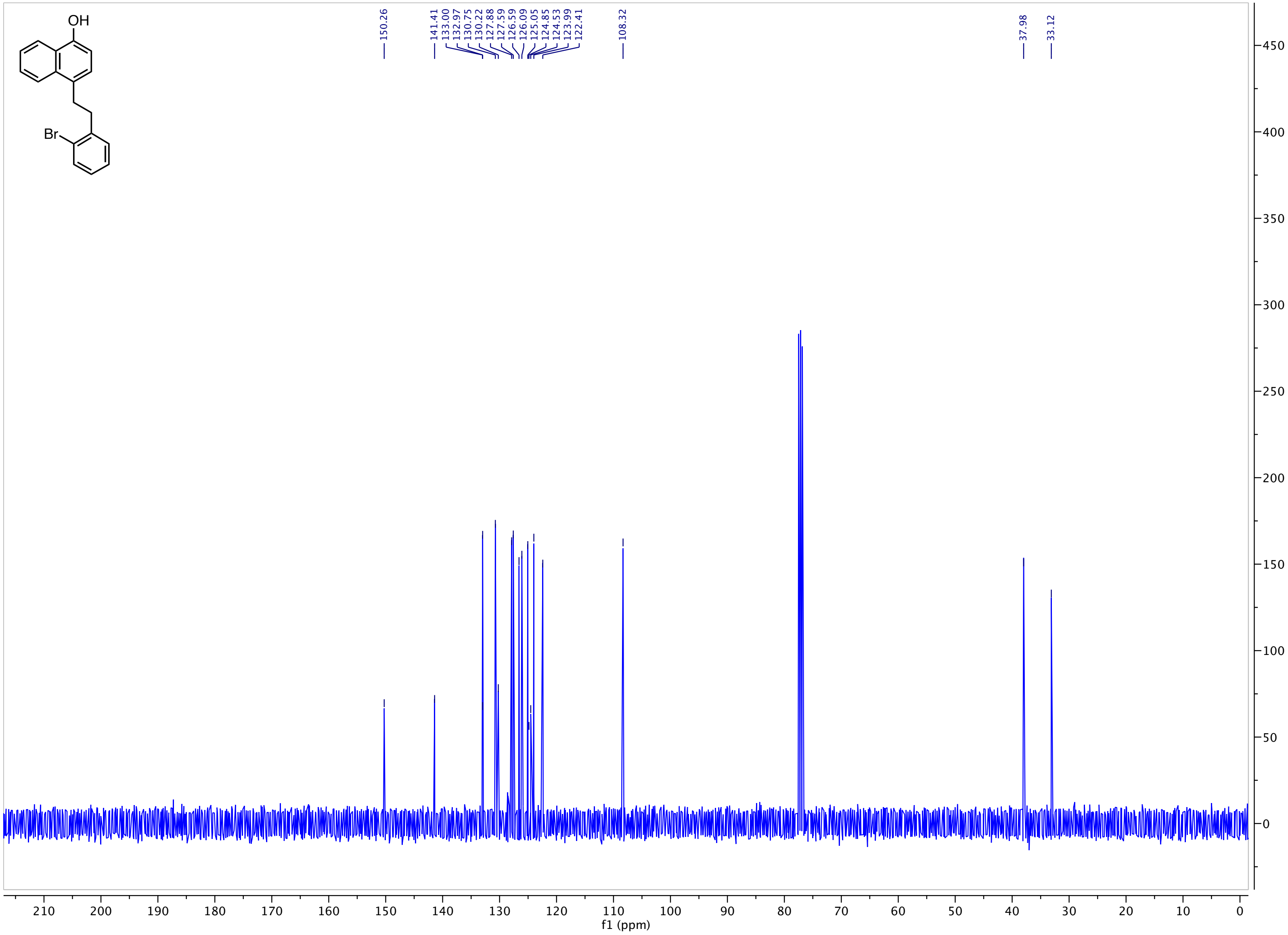

^{1}H NMR (CDCl$_3$): 4-(2-bromophenethyl)-2,3-Dimethylphenol **(1i)**

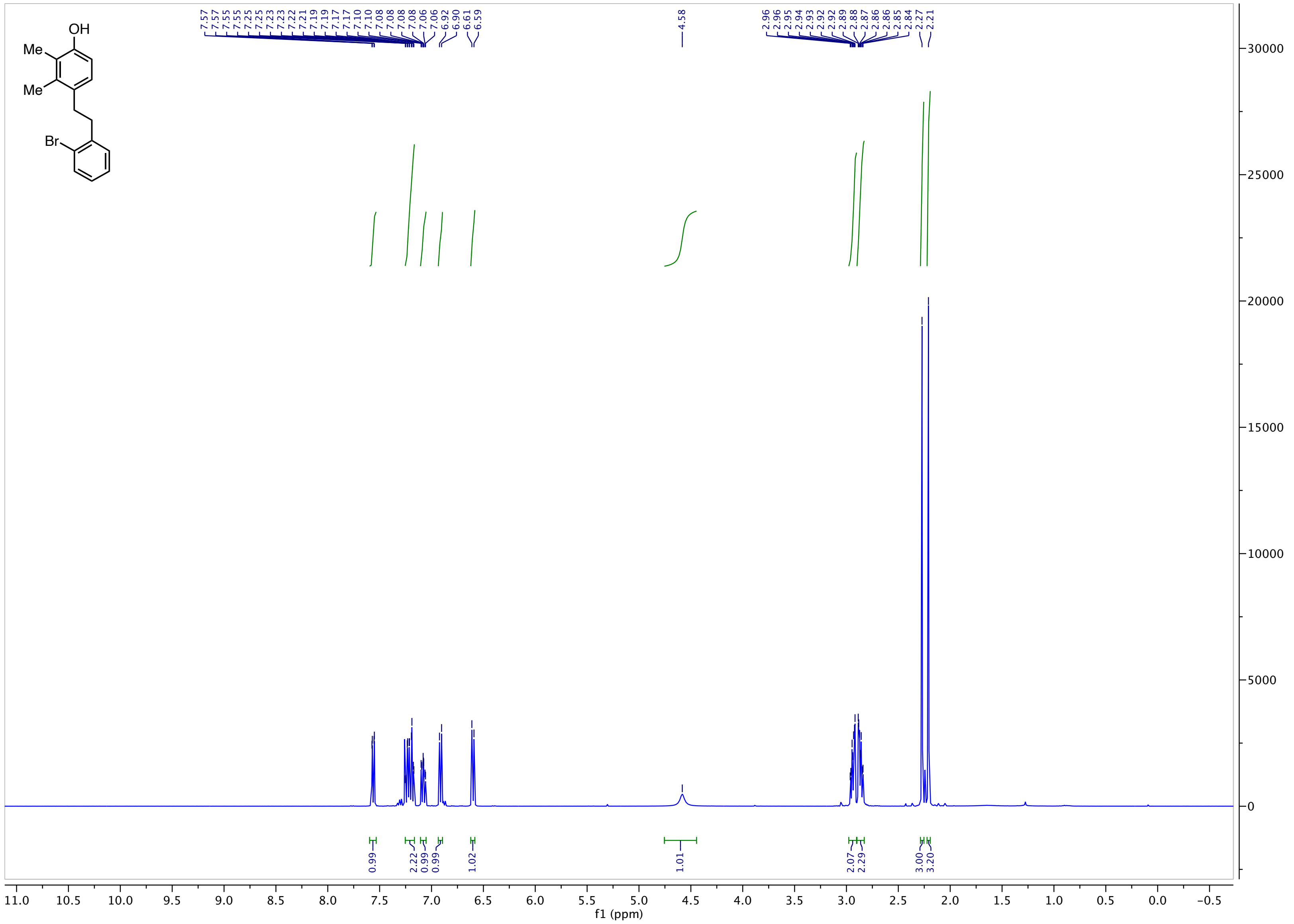

^{13}C NMR ($CDCl_3$): 4-(2-bromophenethyl)-2,3-Dimethylphenol **(1i)**

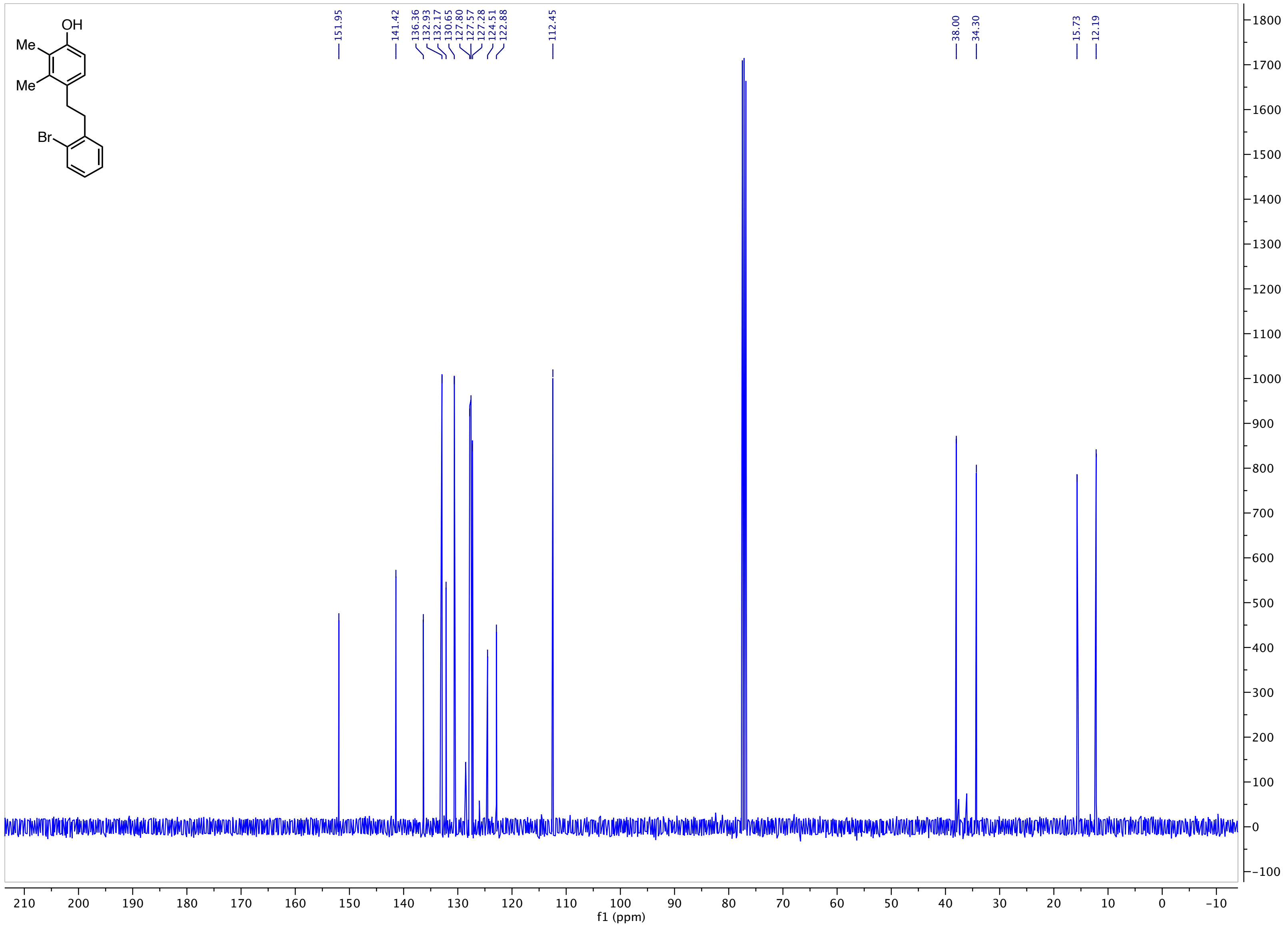

^{1}H NMR ($CDCl_3$): 1-Bromo-2-(2-bromoethyl)benzene **(S10)**

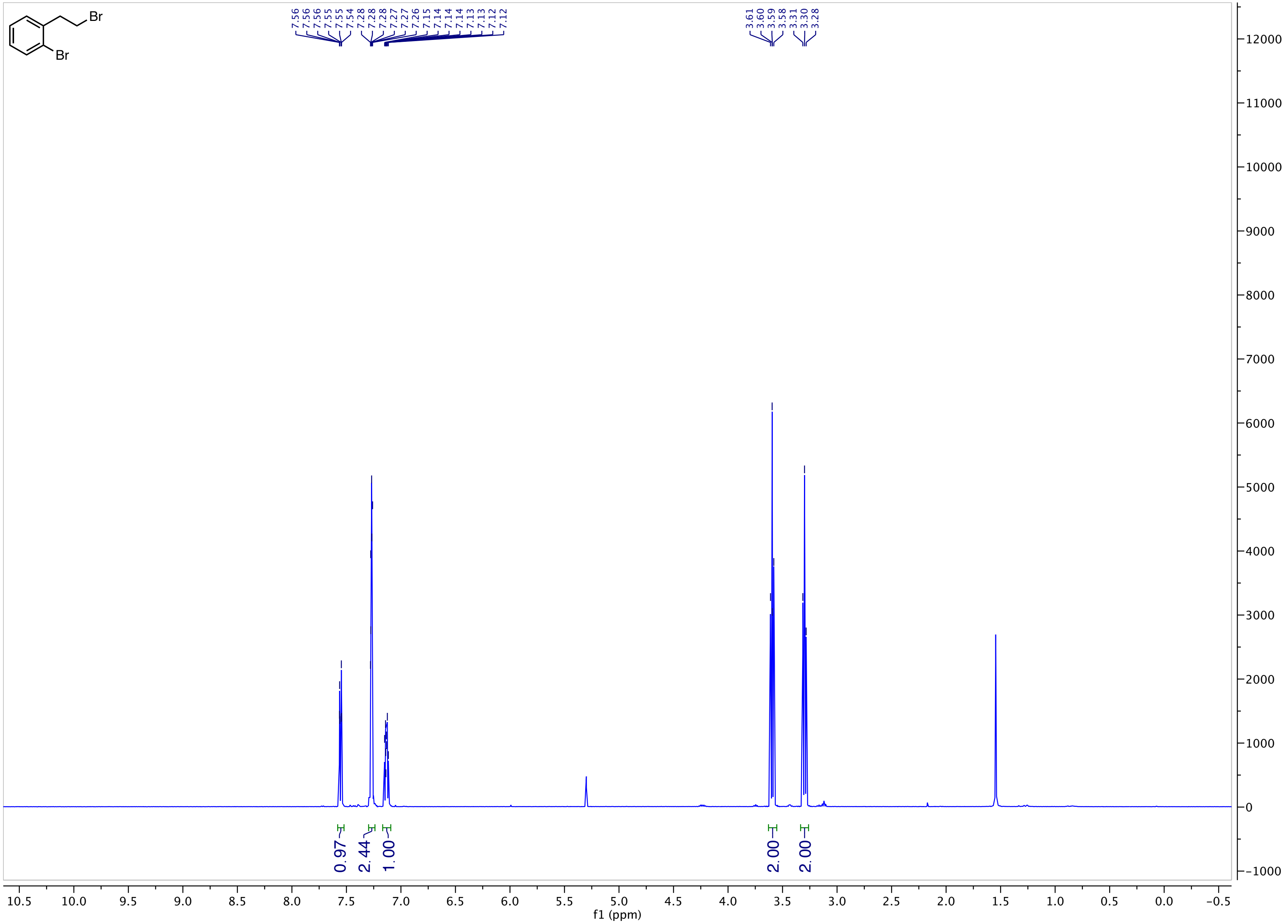

^{13}C NMR (CDCl$_3$): 1-Bromo-2-(2-bromoethyl)benzene (S10)

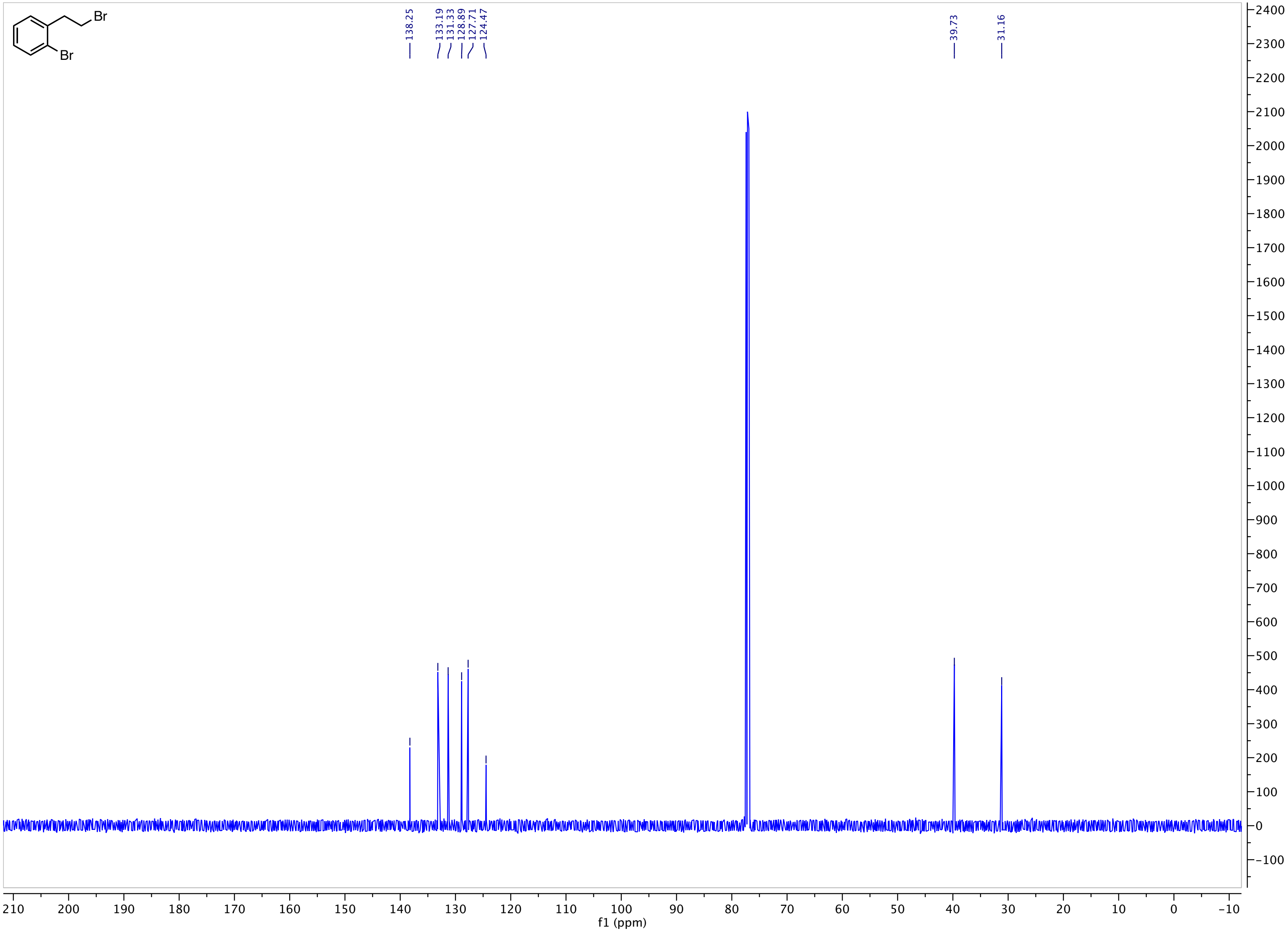

^{1}H NMR (CDCl$_3$): (2-bromophenethyl)Triphenylphosphonium bromide **(S11)**

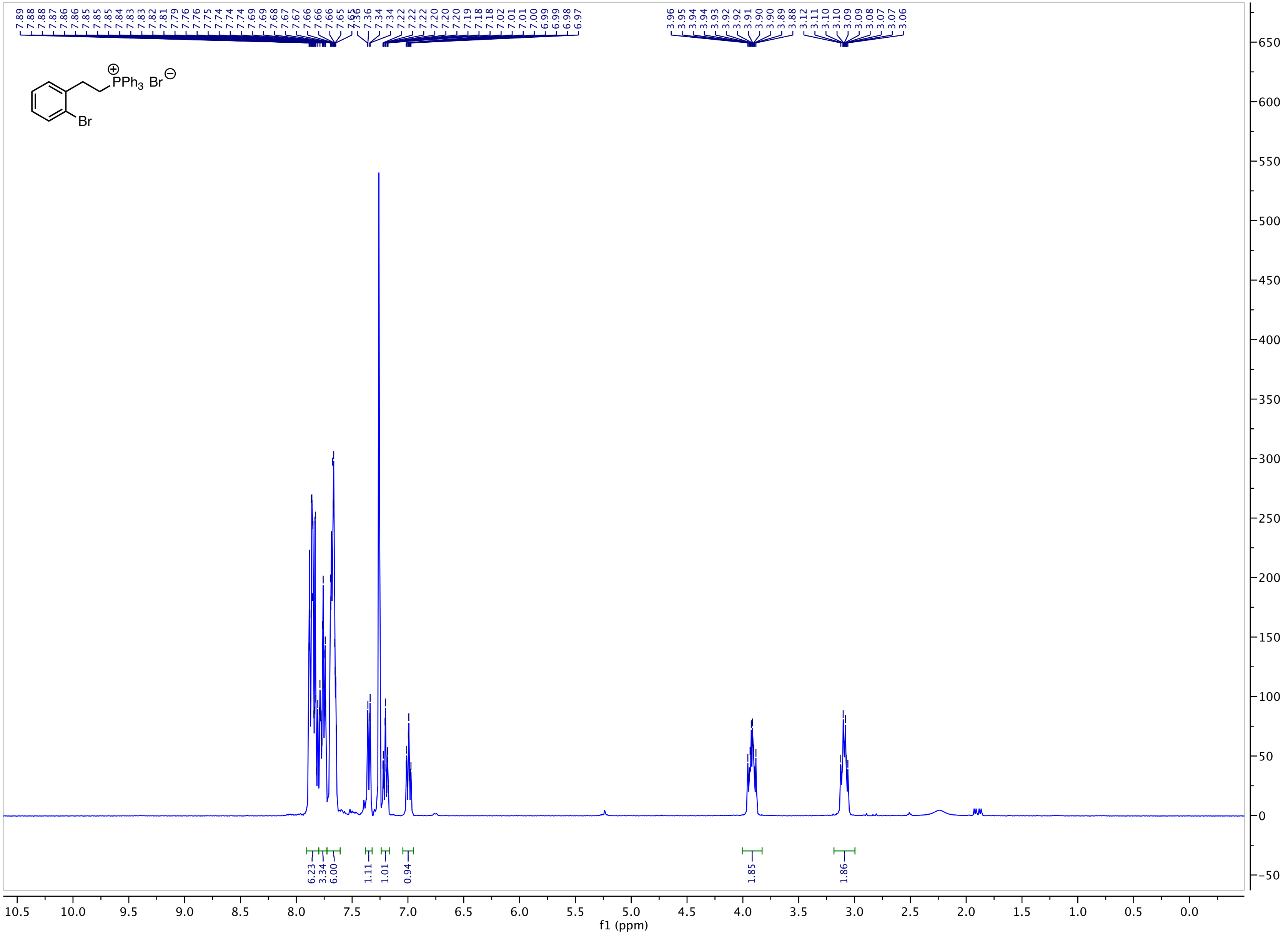

^{13}C NMR ($CDCl_3$): (2-bromophenethyl)Triphenylphosphonium bromide (S11)

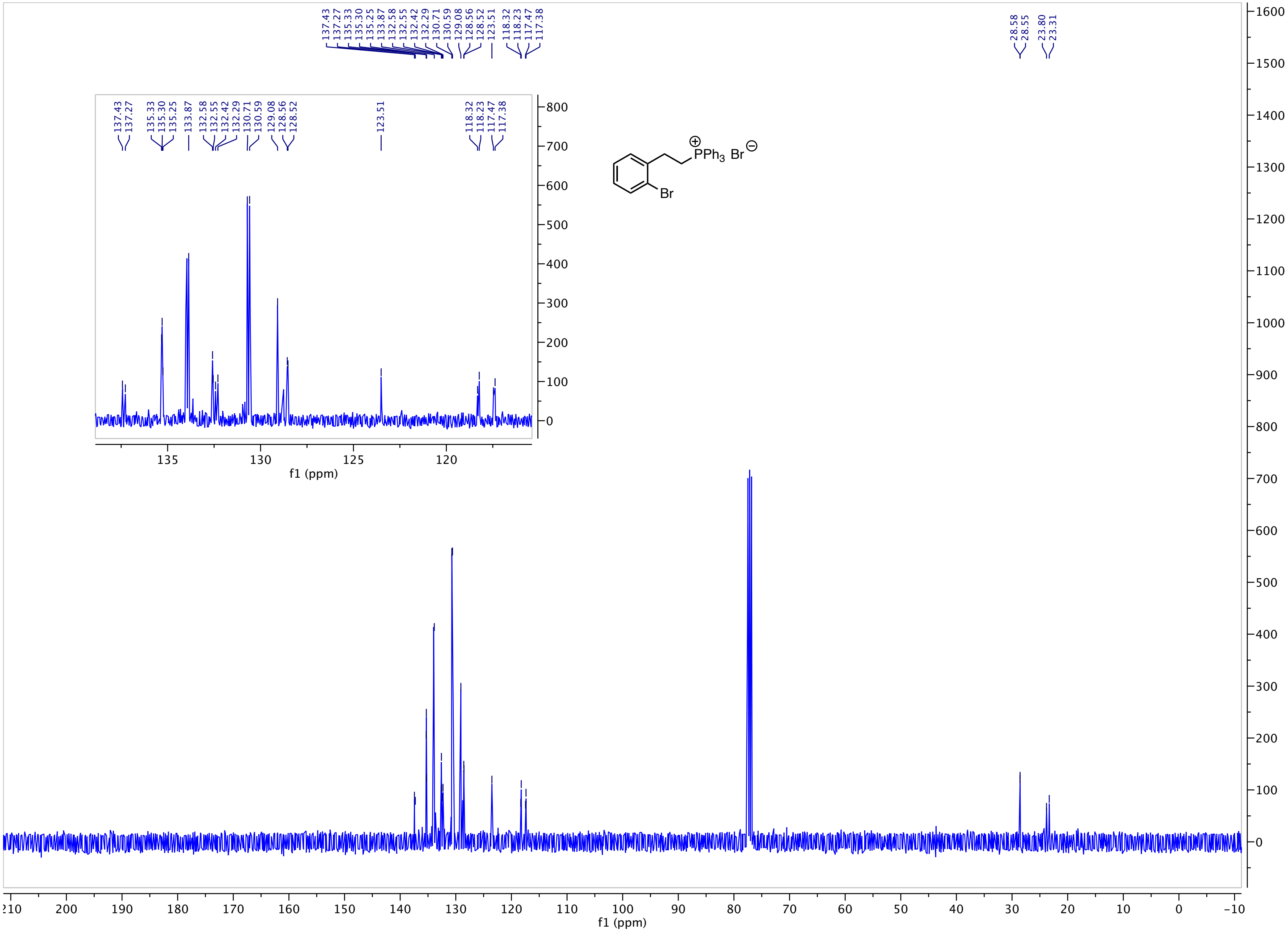

^{31}P NMR (CDCl$_3$): (2-bromophenethyl)Triphenylphosphonium bromide **(S11)**

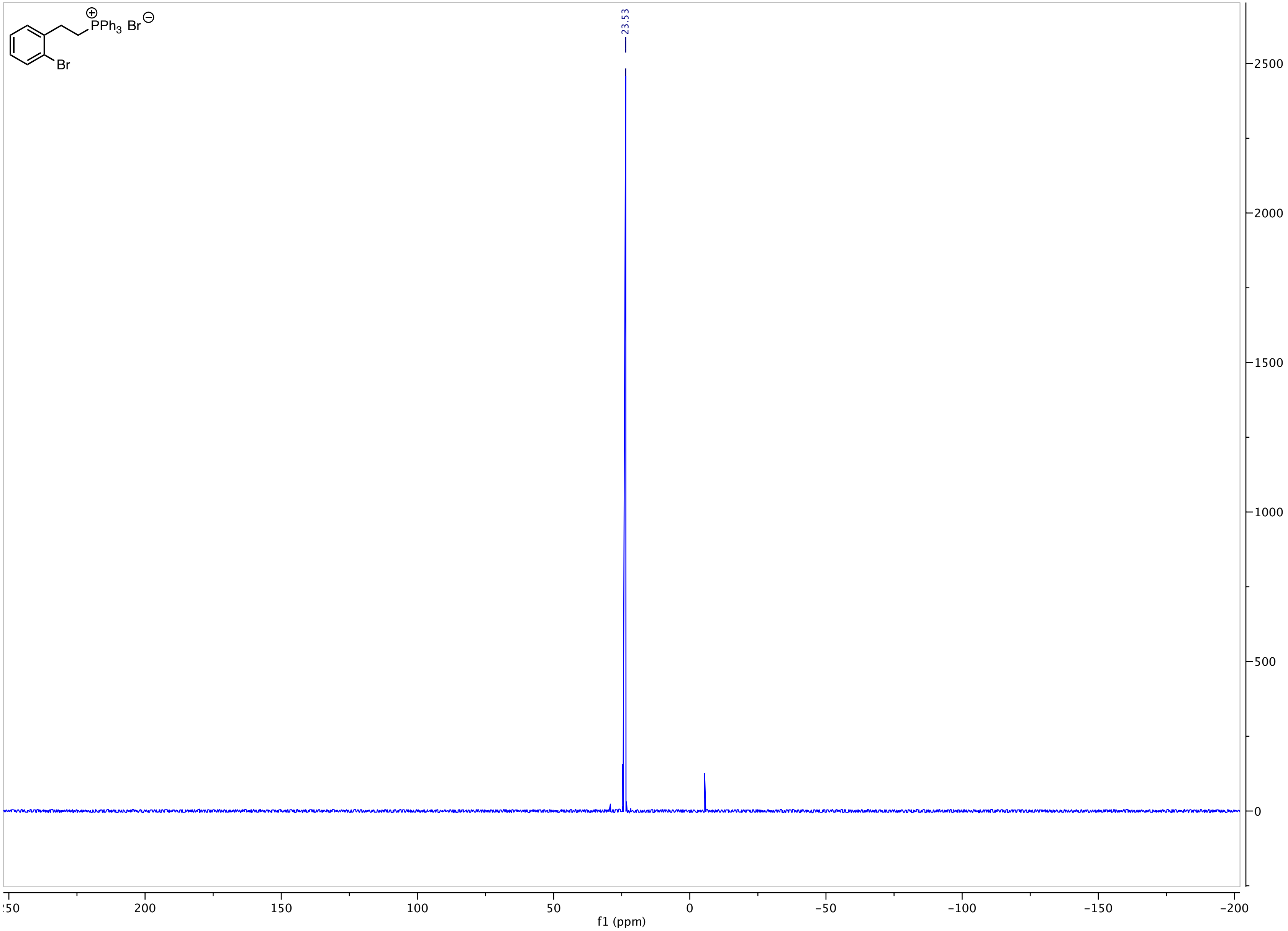

^{1}H NMR ($CDCl_3$): 4-(benzyloxy)-1-(3-(2-bromophenyl)propyl)-2-Methoxybenzene **(S12)**

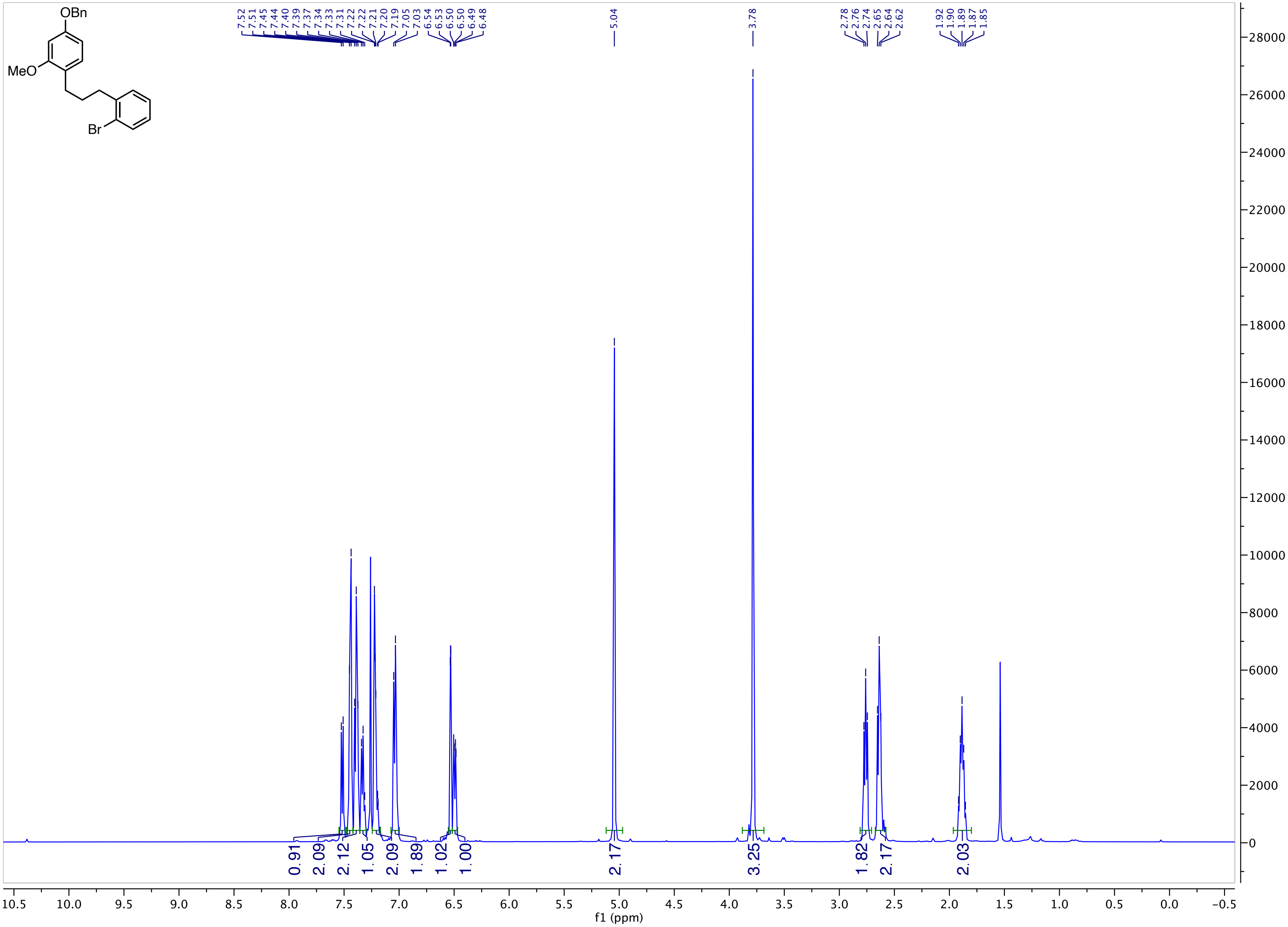

^{13}**C NMR** ($CDCl_3$): 4-(benzyloxy)-1-(3-(2-bromophenyl)propyl)-2-Methoxybenzene **(S12)**

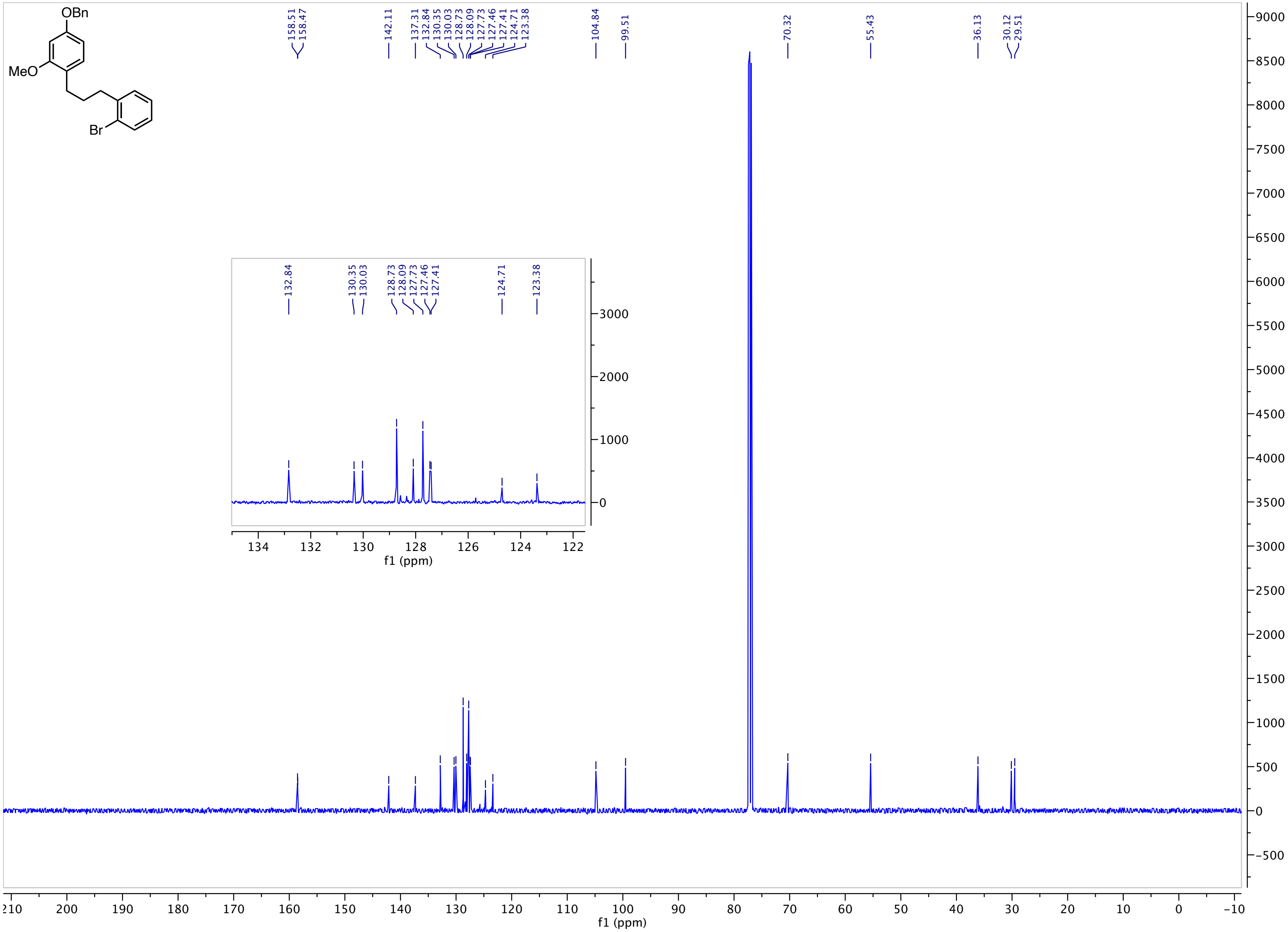

^{1}H NMR ($CDCl_3$): 4-(3-(2-bromophenyl)propyl)-3-Methoxyphenol (1j)

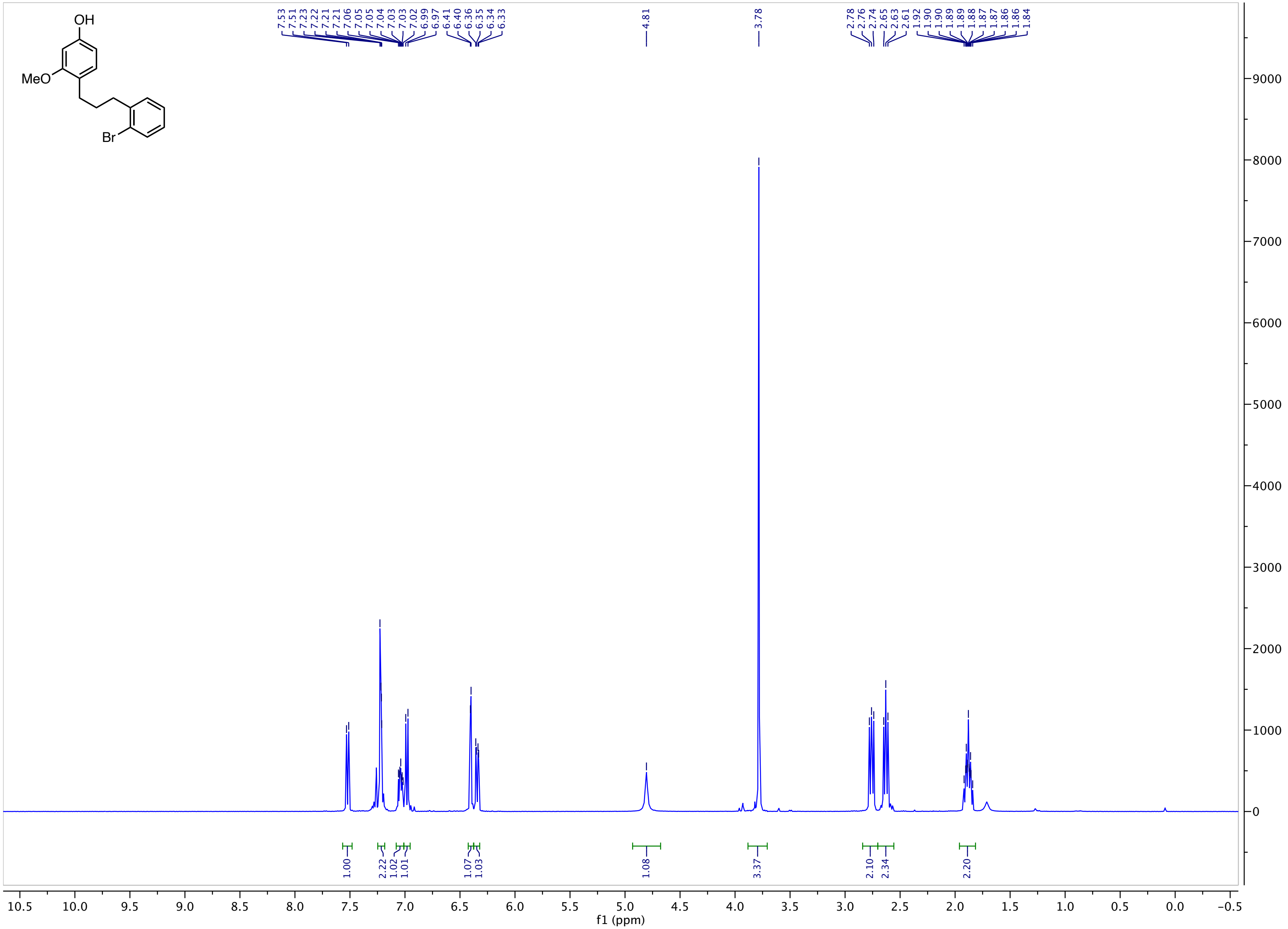

^{13}C NMR ($CDCl_3$): 4-(3-(2-bromophenyl)propyl)-3-Methoxyphenol (1j)

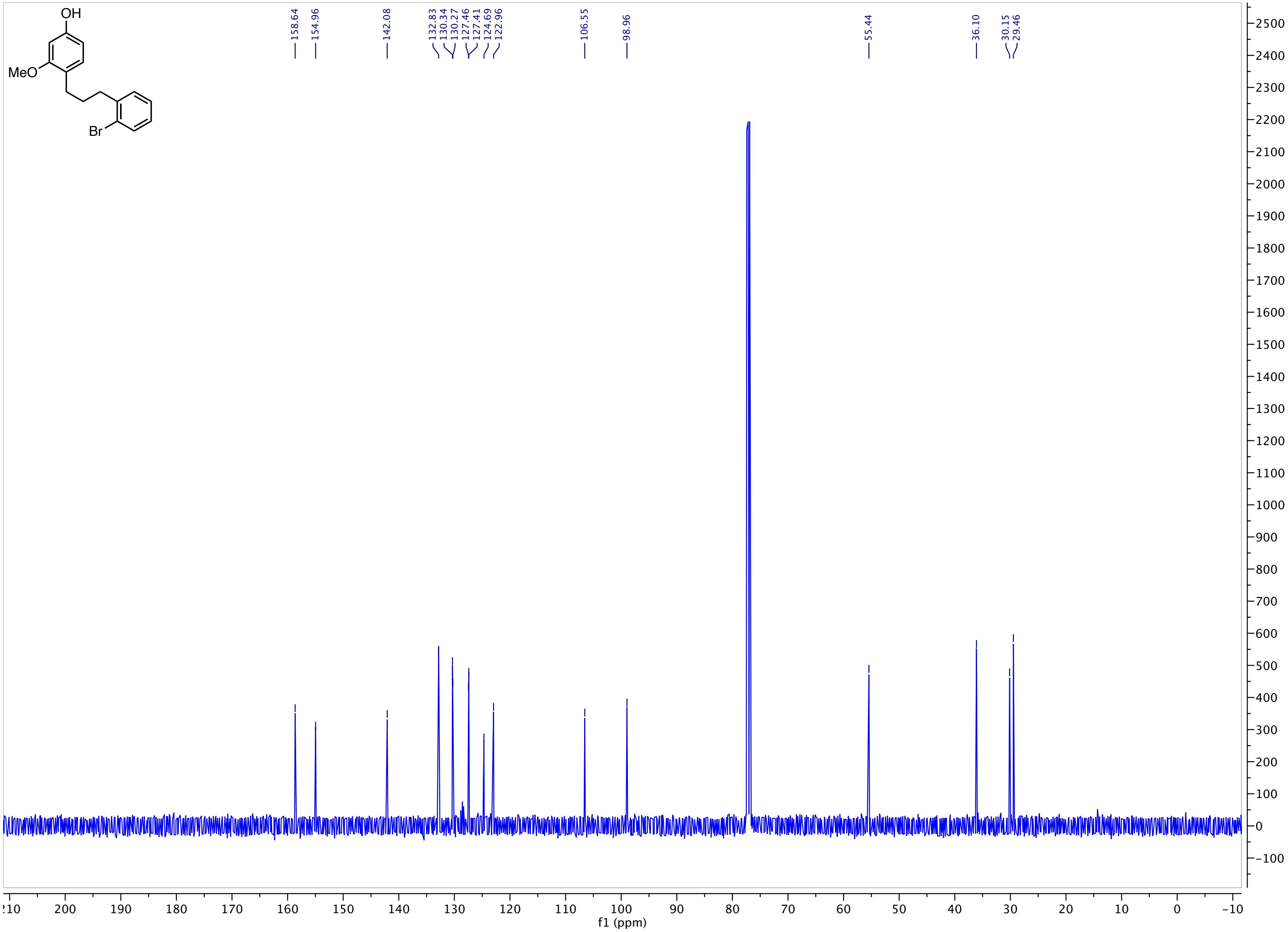

^{1}H NMR (CDCl$_3$): 1-Bromo-2-(3-bromopropyl)benzene (S13)

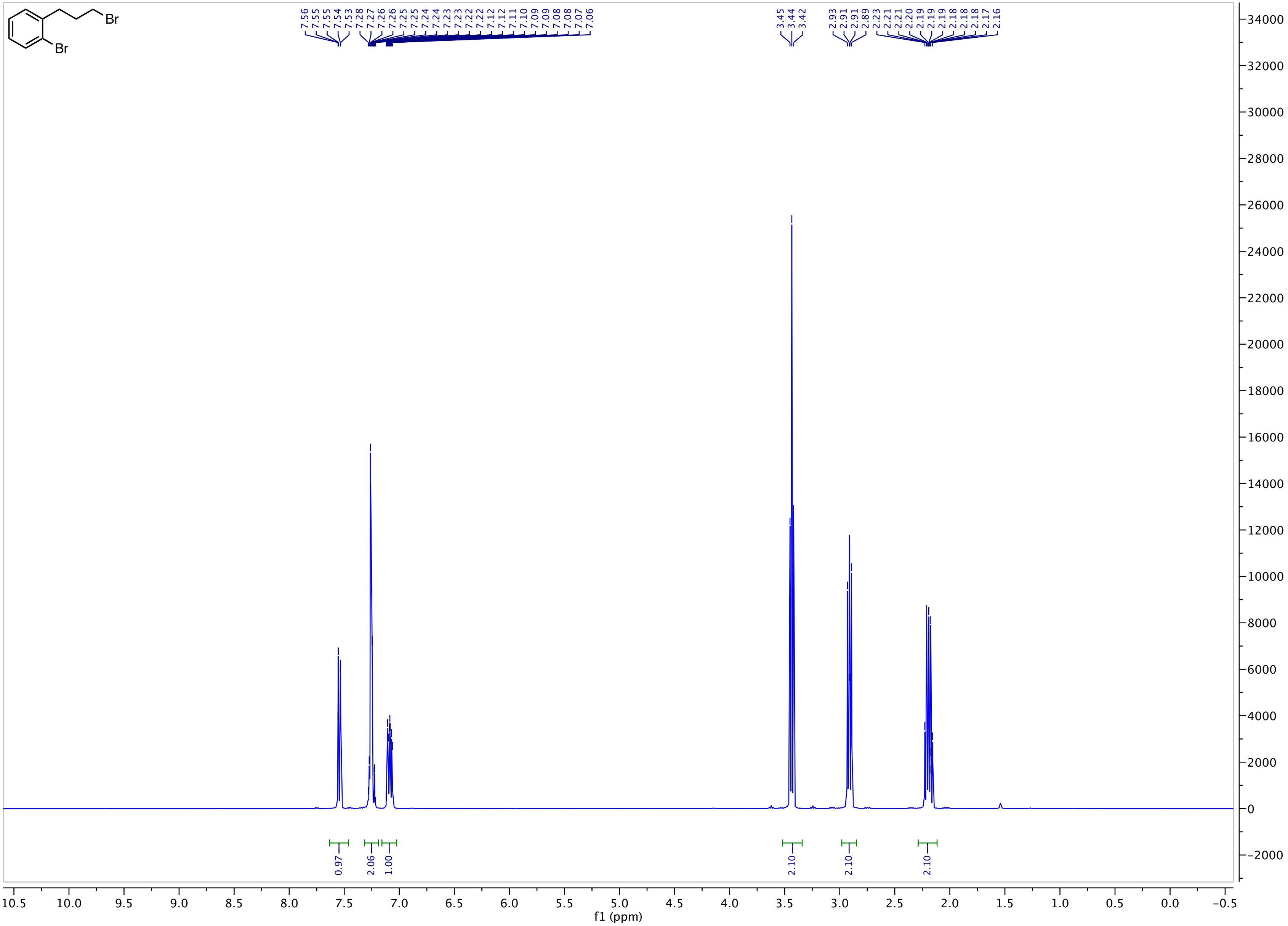

^{13}C NMR ($CDCl_3$): 1-Bromo-2-(3-bromopropyl)benzene **(S13)**

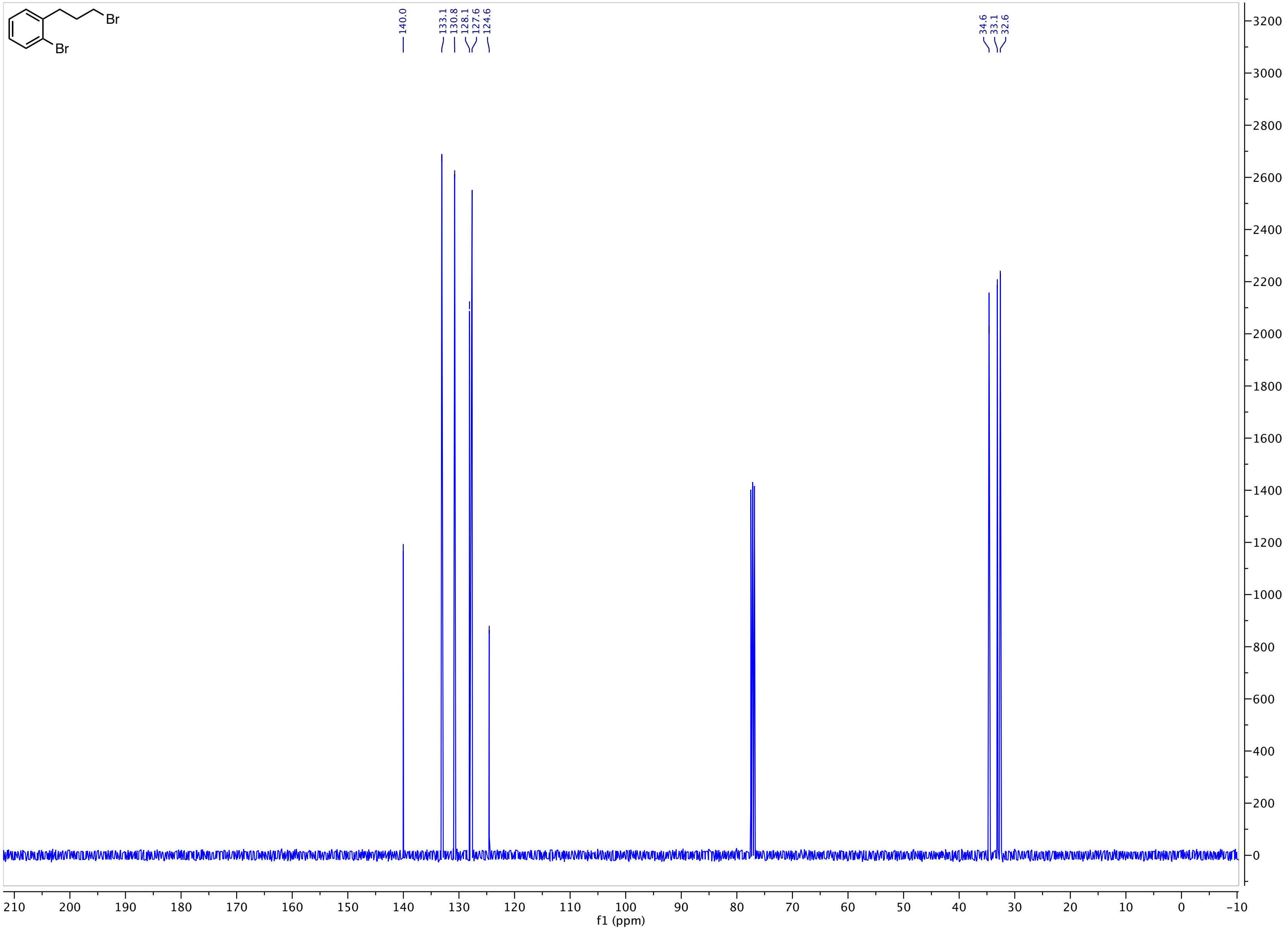

^{1}H NMR (CDCl$_3$): (3-(2-bromophenyl)propyl)Triphenylphosphonium bromide **(S14)**

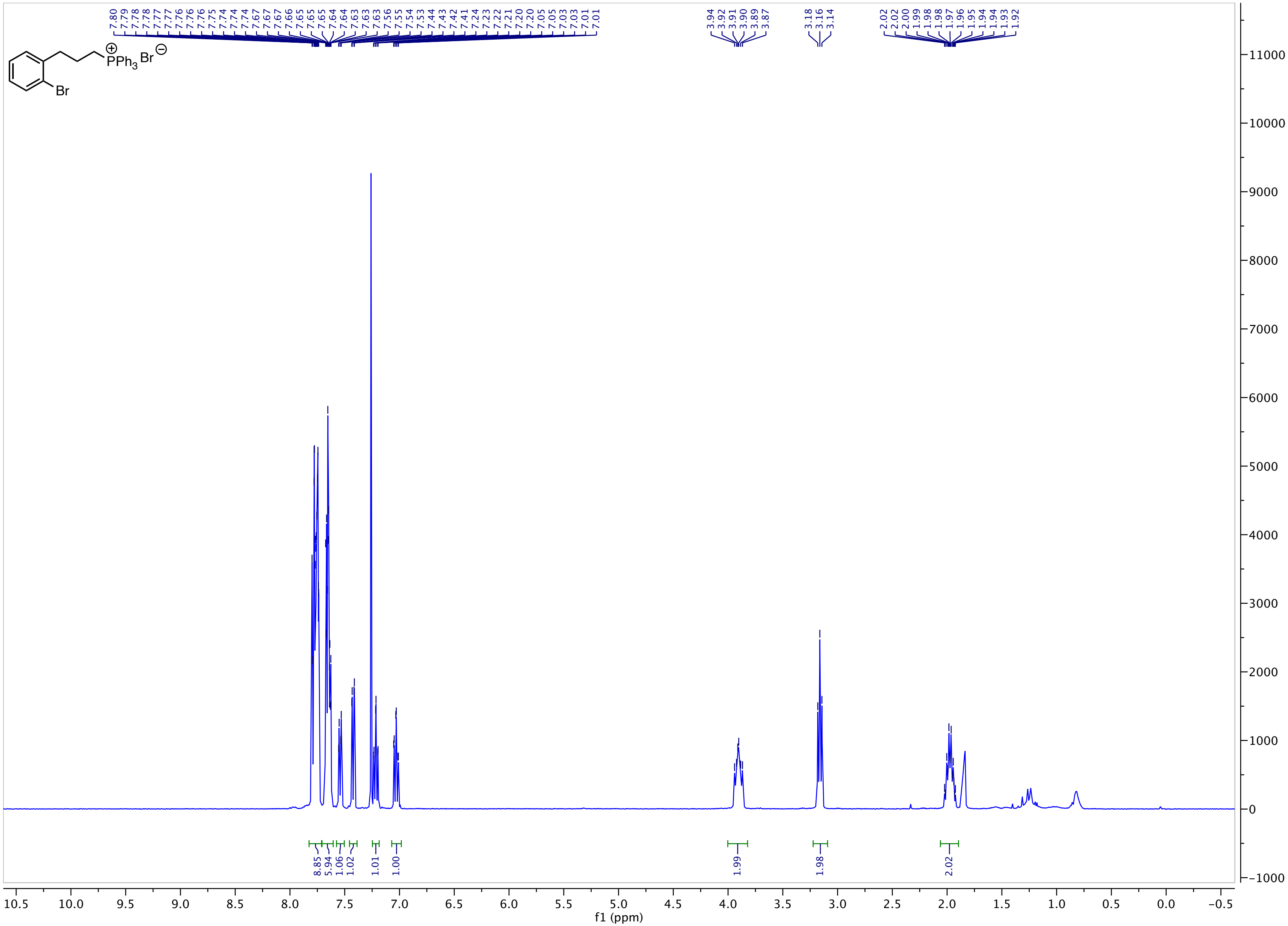

^{13}C NMR ($CDCl_3$): (3-(2-bromophenyl)propyl)Triphenylphosphonium bromide **(S14)**

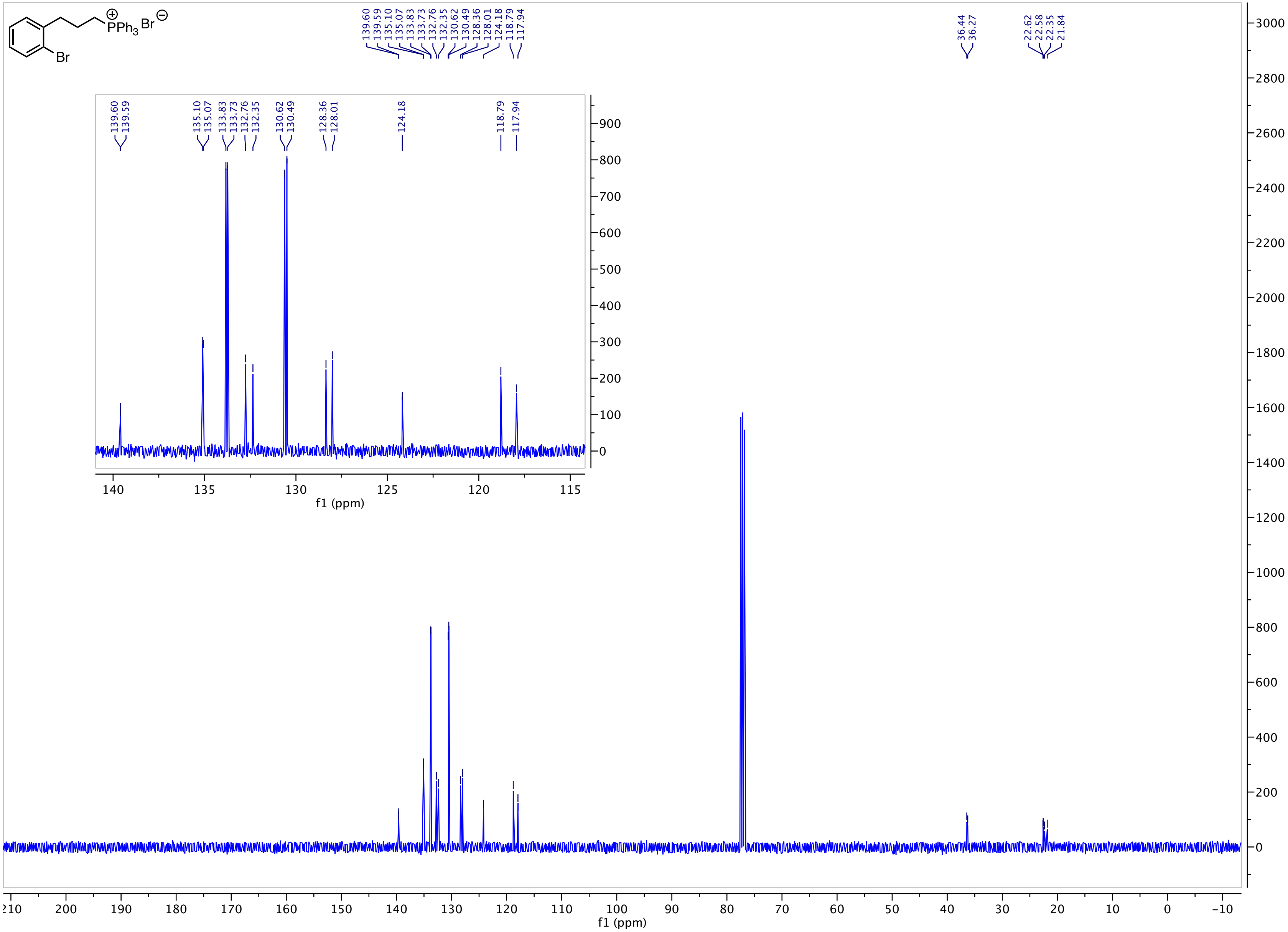

^{1}H NMR ($CDCl_3$): 4-(4-(2-bromophenyl)butyl)-3-Methoxyphenol (1k)

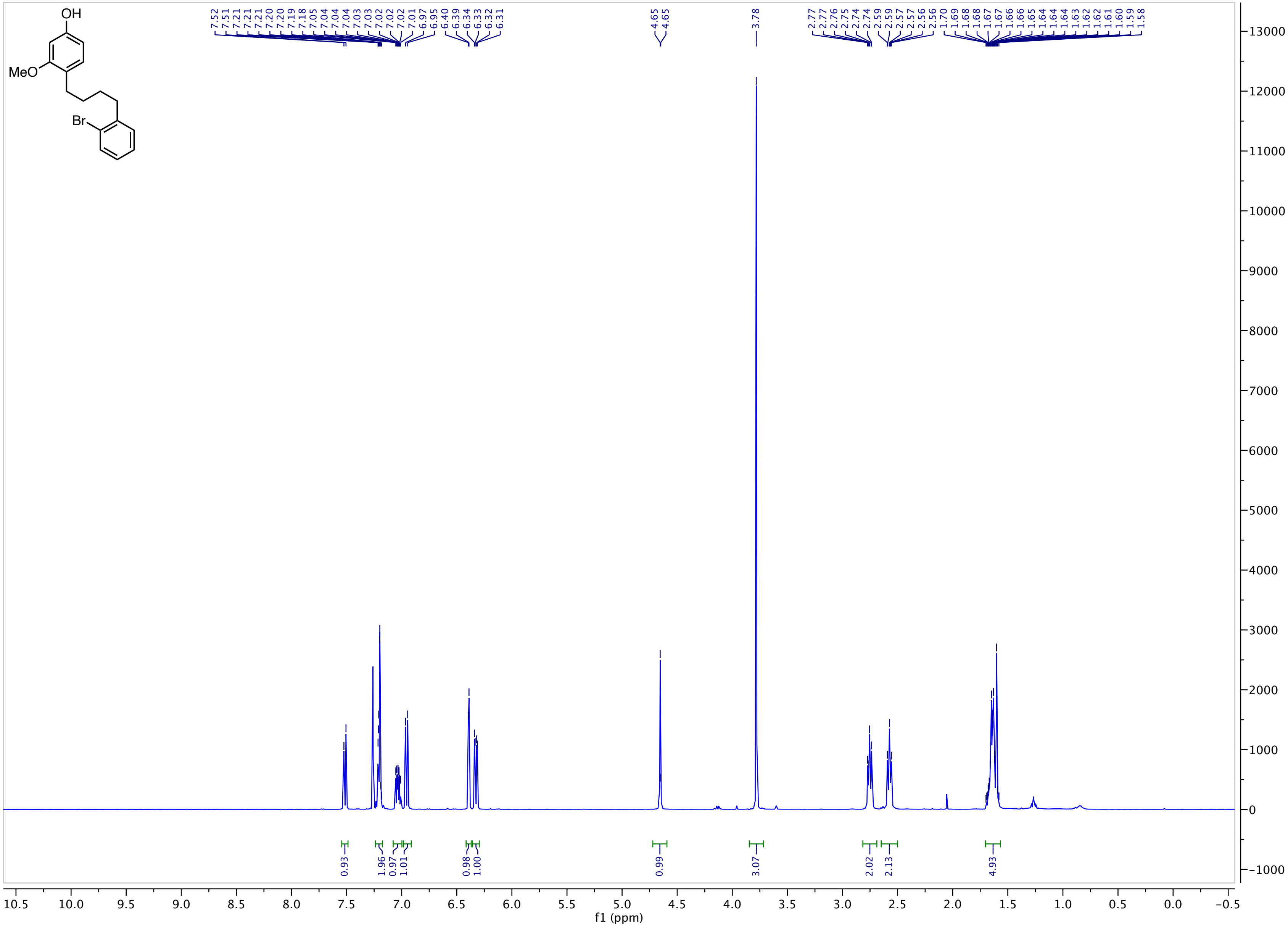

^{13}C NMR ($CDCl_3$): 4-(4-(2-bromophenyl)butyl)-3-Methoxyphenol **(1k)**

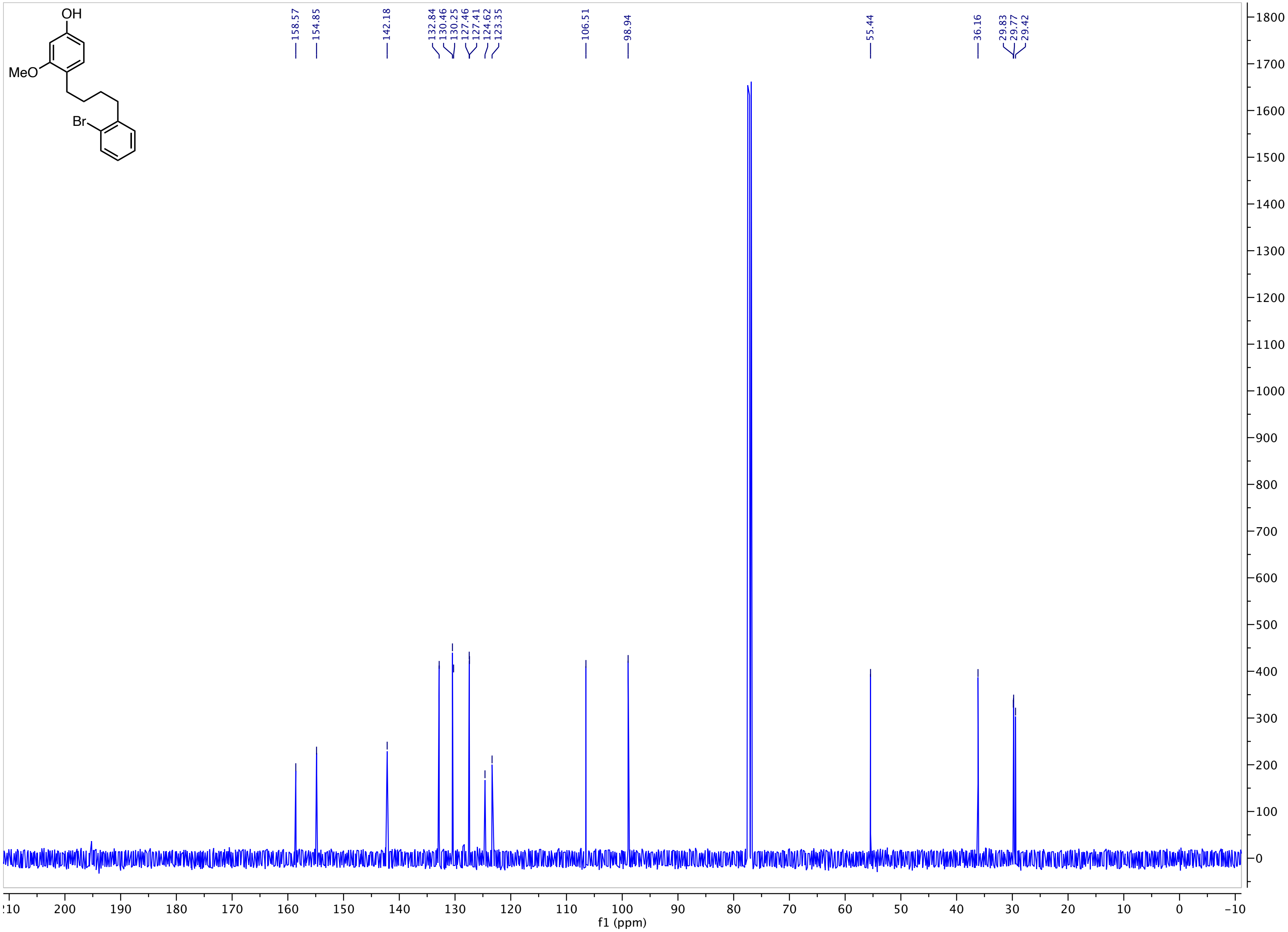

^{1}H NMR (MeCN-d_3): (2-bromo-5-(trifluoromethyl)benzyl)Triphenylphosphonium bromide **(S15)**

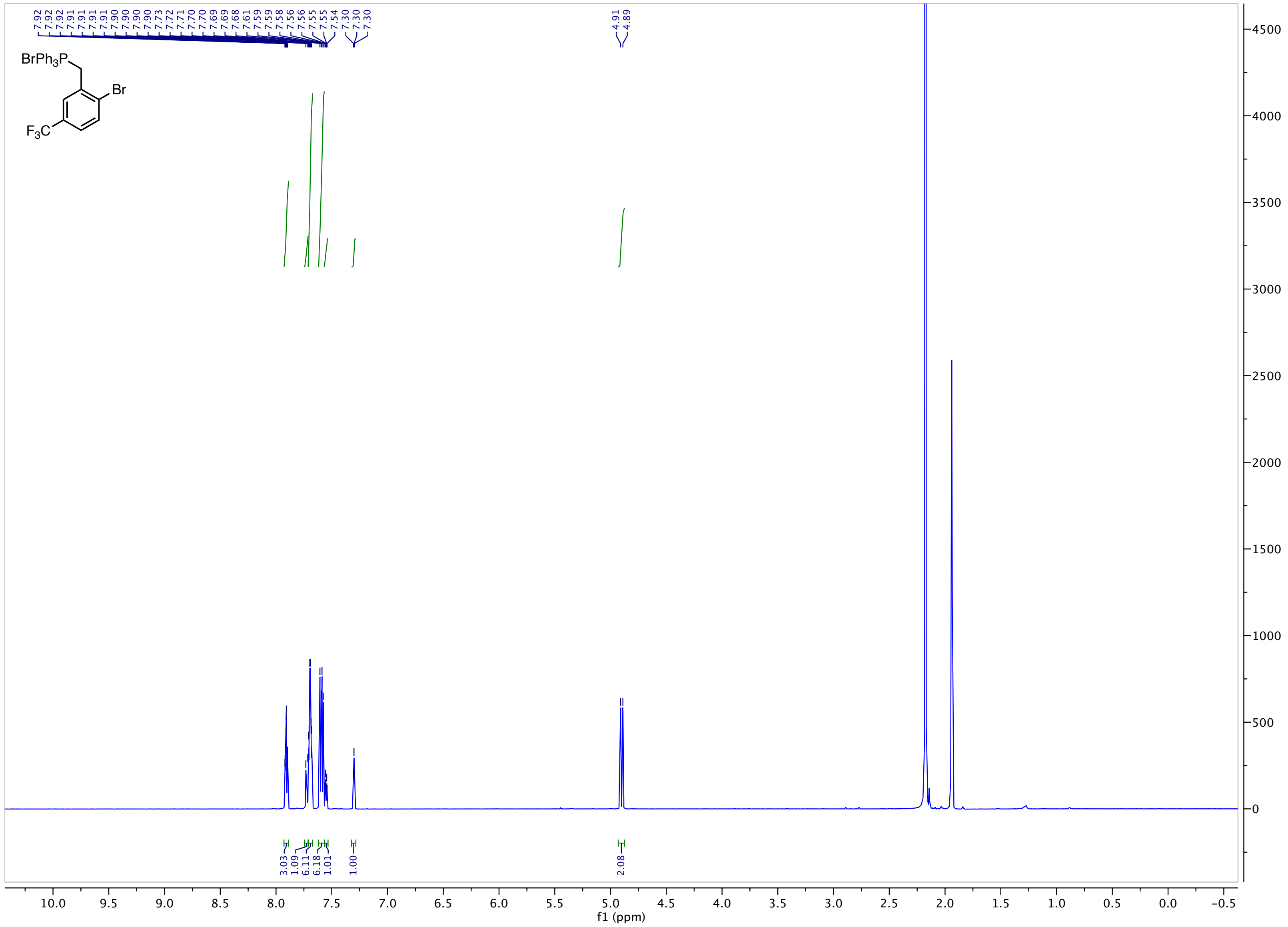

^{13}C NMR (MeCN-d_3): (2-bromo-5-(trifluoromethyl)benzyl)Triphenylphosphonium bromide **(S15)**

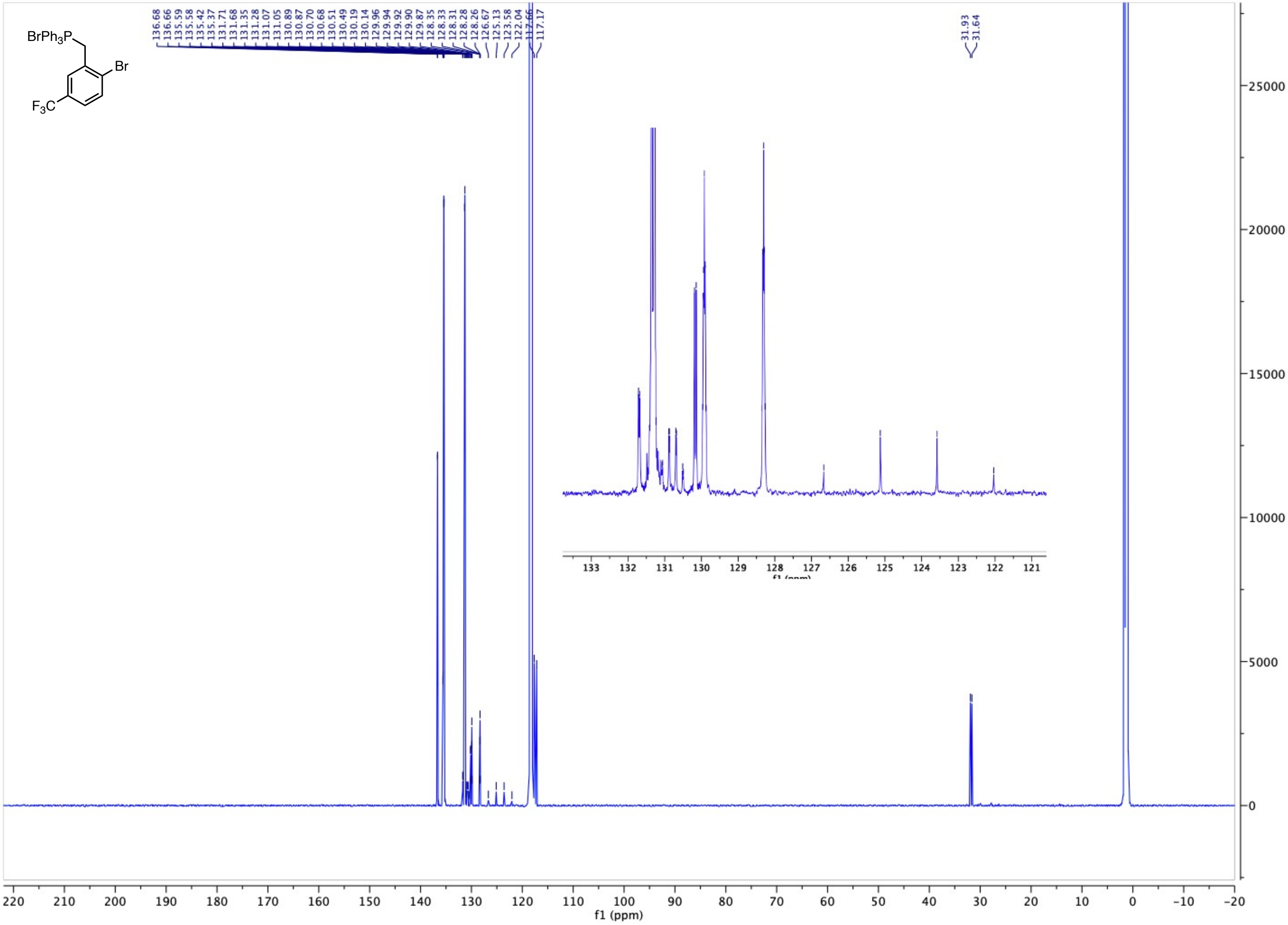

^{19}**F NMR** (MeCN-d_3): (2-bromo-5-(trifluoromethyl)benzyl)Triphenylphosphonium bromide **(S15)**

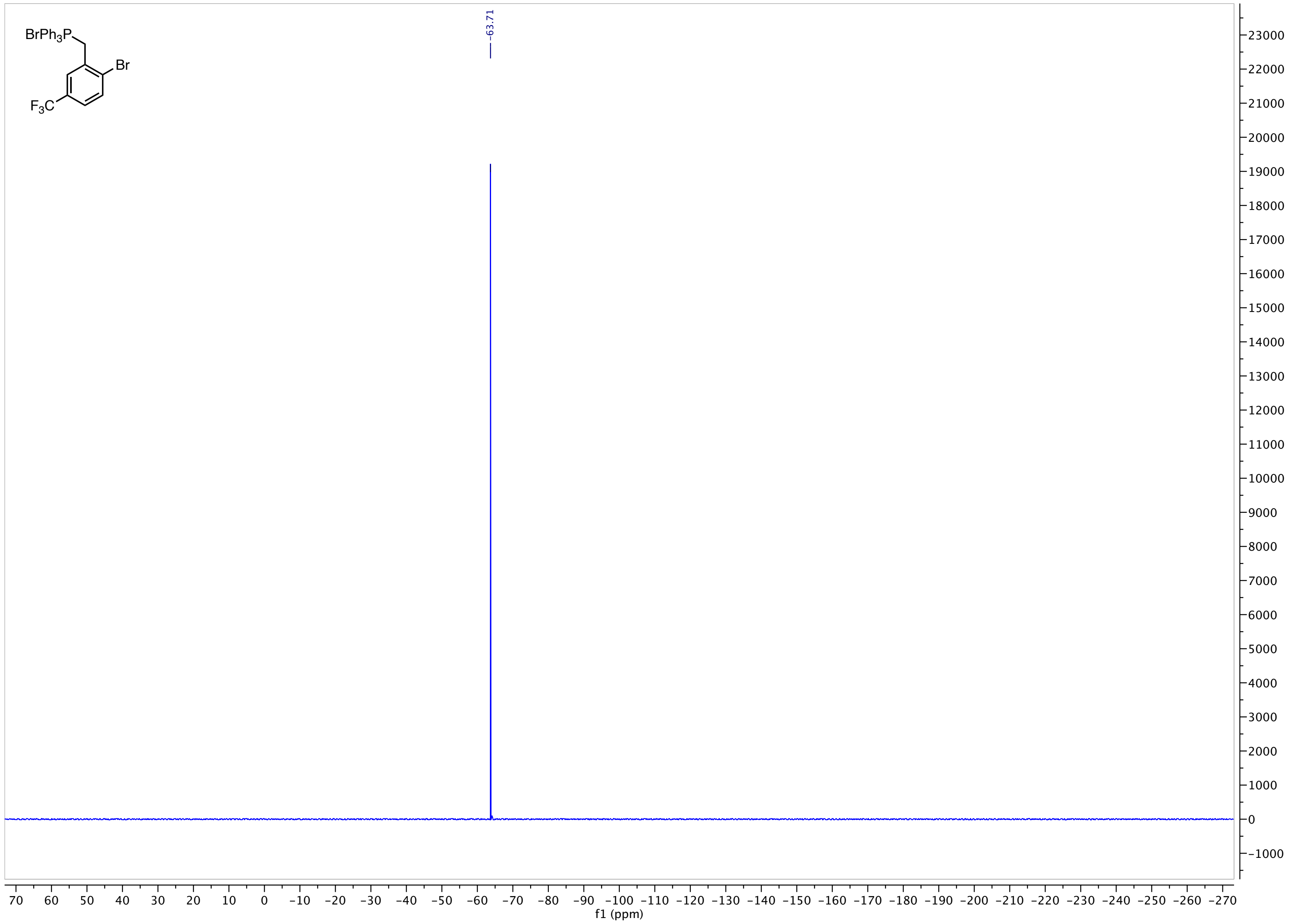

^{31}P NMR (MeCN-d_3): (2-bromo-5-(trifluoromethyl)benzyl)Triphenylphosphonium bromide **(S15)**

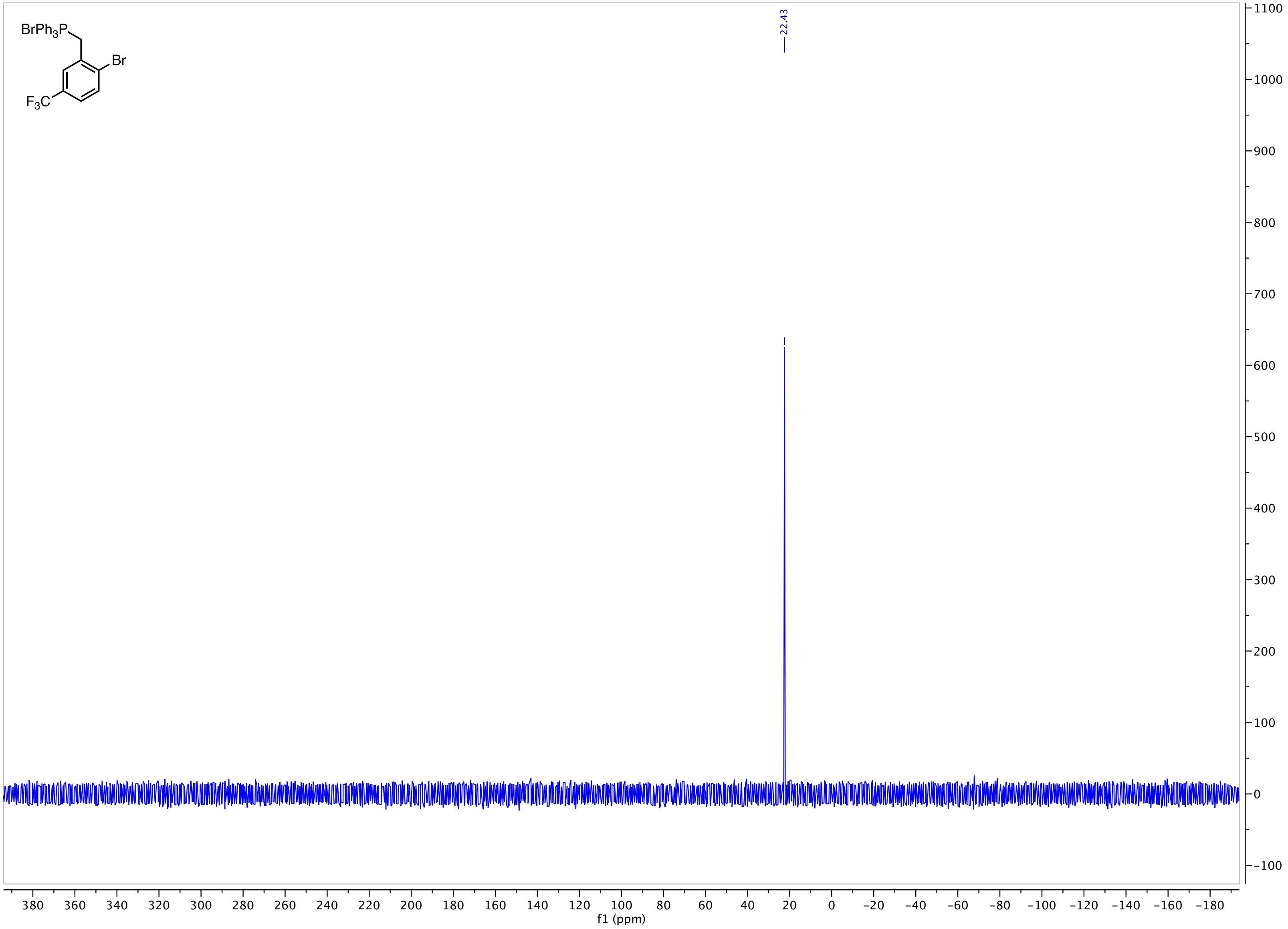

^{1}H NMR ($CDCl_3$): 4-(2-bromo-5-(trifluoromethyl)phenethyl)-3-Methoxyphenol **(1l)**

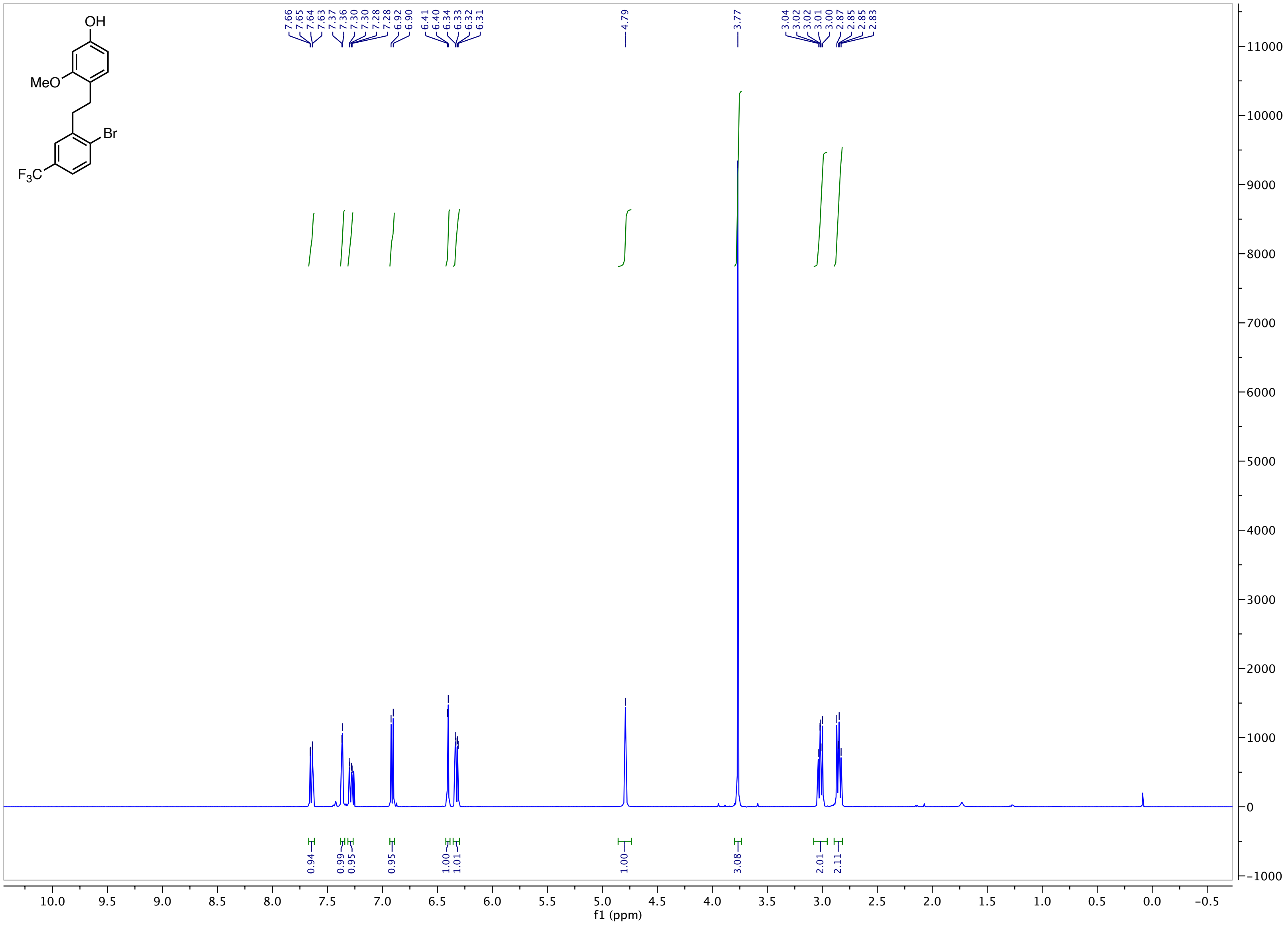

^{13}C NMR ($CDCl_3$): 4-(2-bromo-5-(trifluoromethyl)phenethyl)-3-Methoxyphenol (1l)

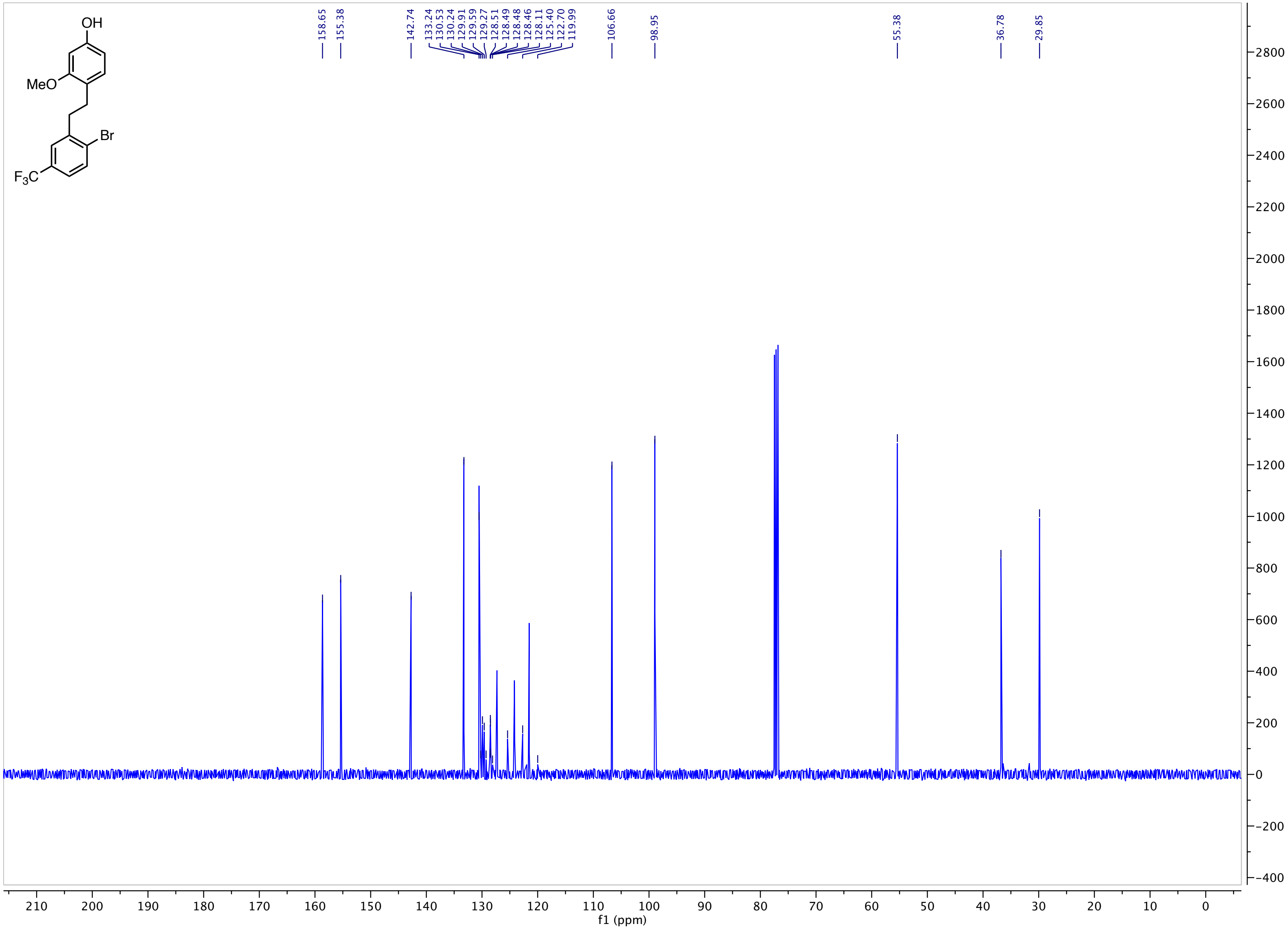

^{19}**F NMR** (CDCl$_3$): 4-(2-bromo-5-(trifluoromethyl)phenethyl)-3-Methoxyphenol **(1l)**

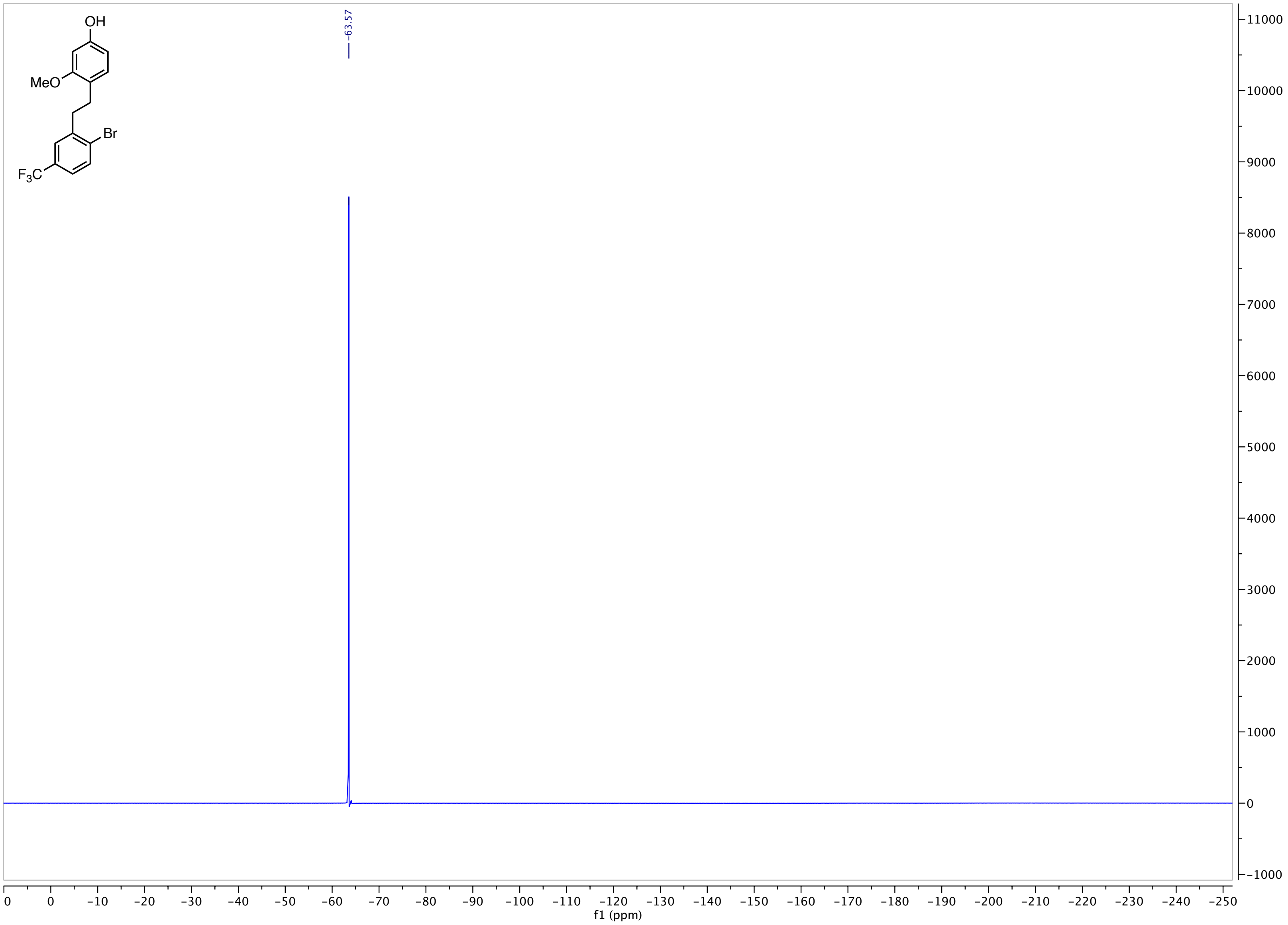

^{1}H NMR (MeCN-d_3): (2-bromo-4,5-dimethoxybenzyl)Triphenylphosphonium bromide **(S16)**

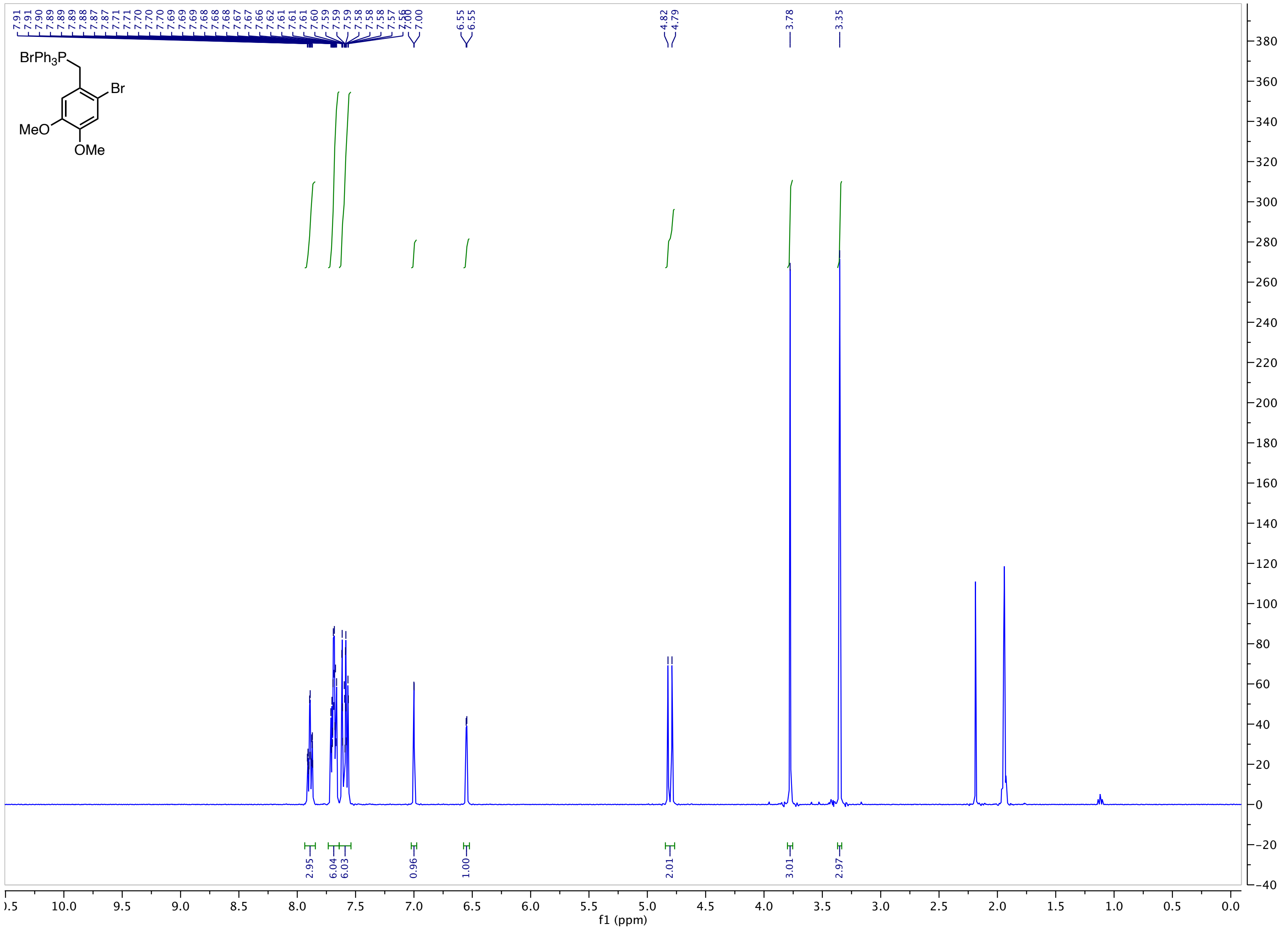

^{13}C NMR (MeCN-d_3): (2-bromo-4,5-dimethoxybenzyl)Triphenylphosphonium bromide **(S16)**

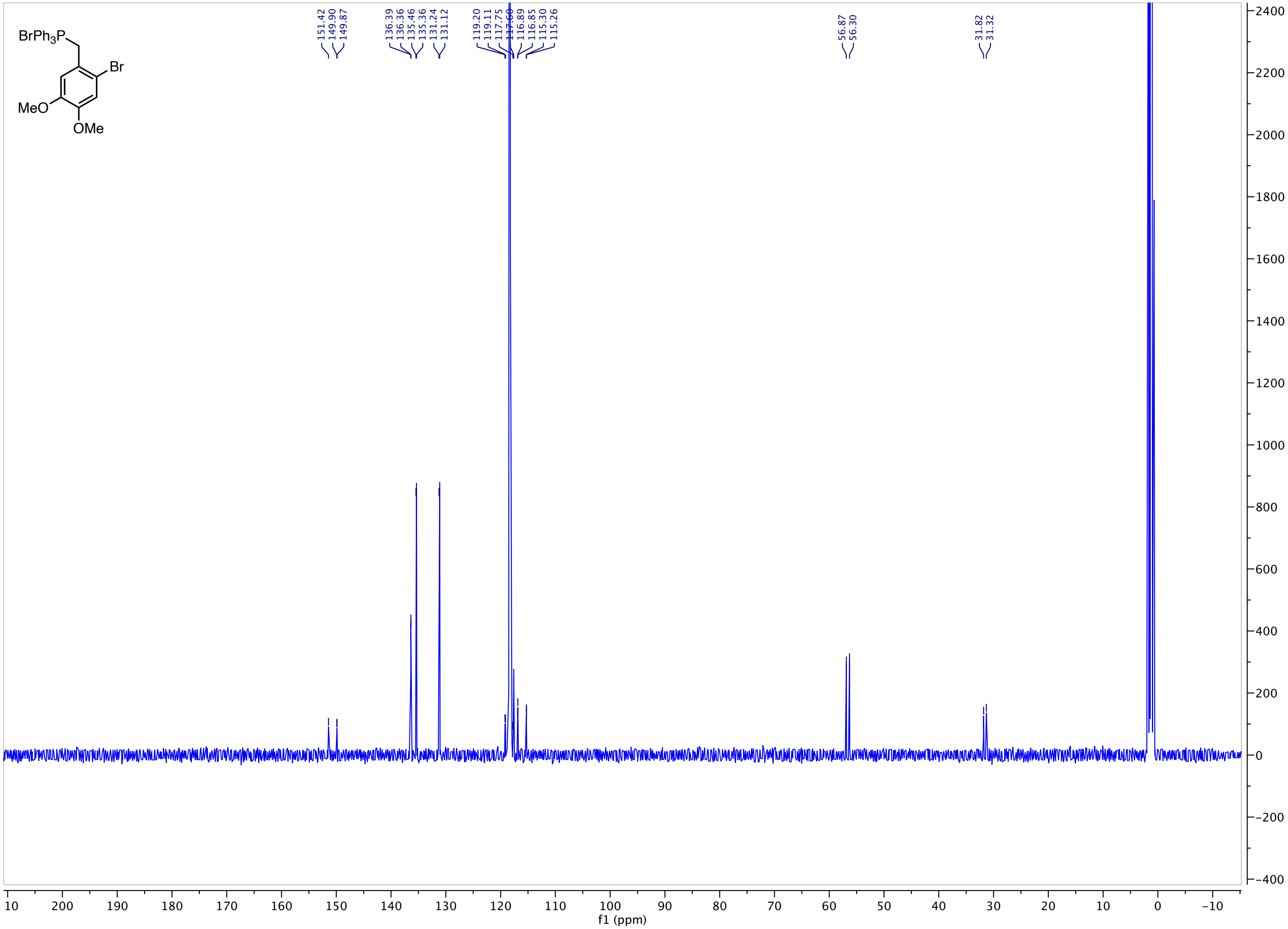

^{31}P NMR (MeCN-d_3): (2-bromo-4,5-dimethoxybenzyl)Triphenylphosphonium bromide **(S16)**

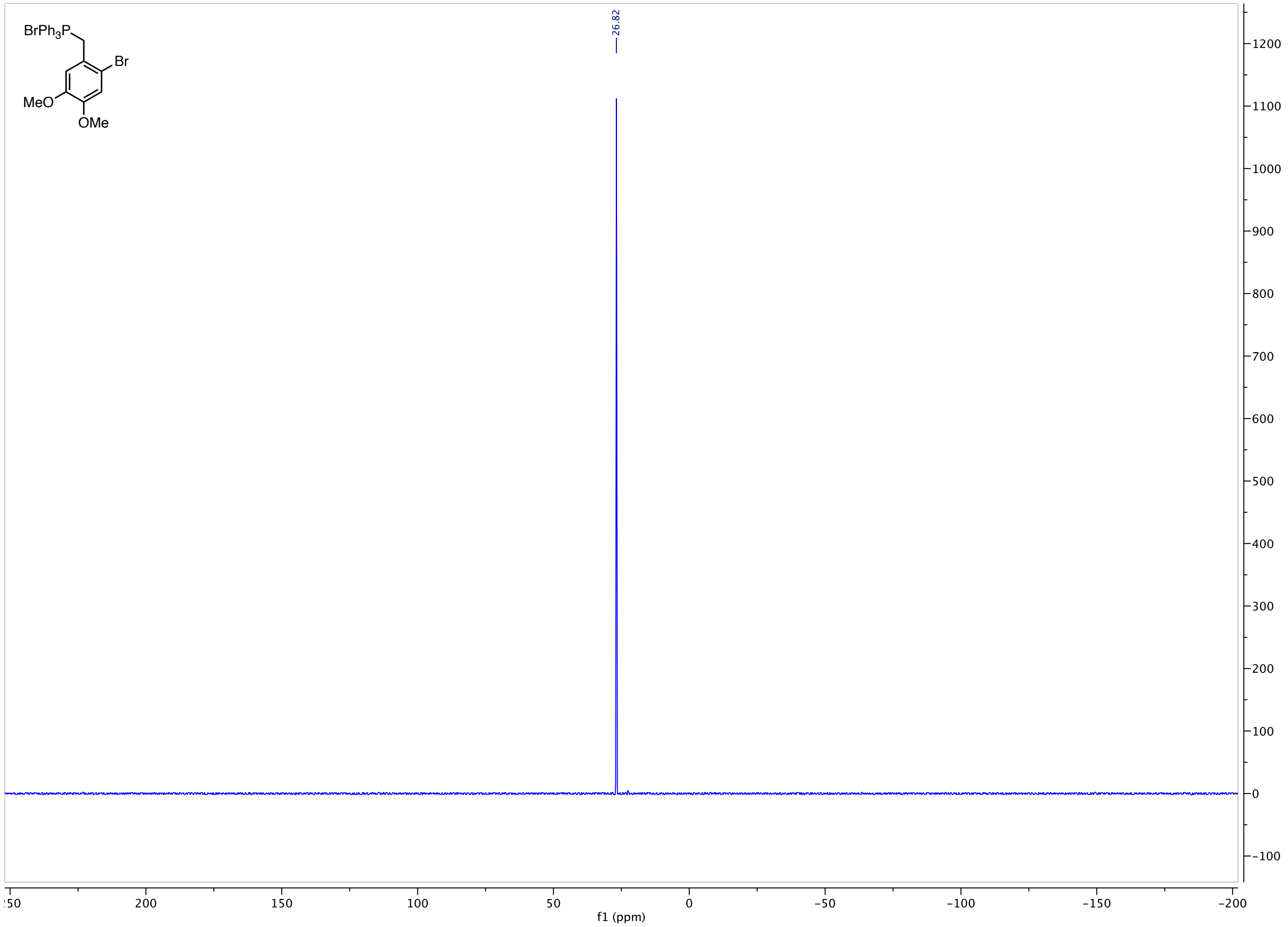

^{1}H NMR (Acetone-d_6): 4-(2-bromo-4,5-dimethoxyphenethyl)-3-Methoxyphenol **(1m)**

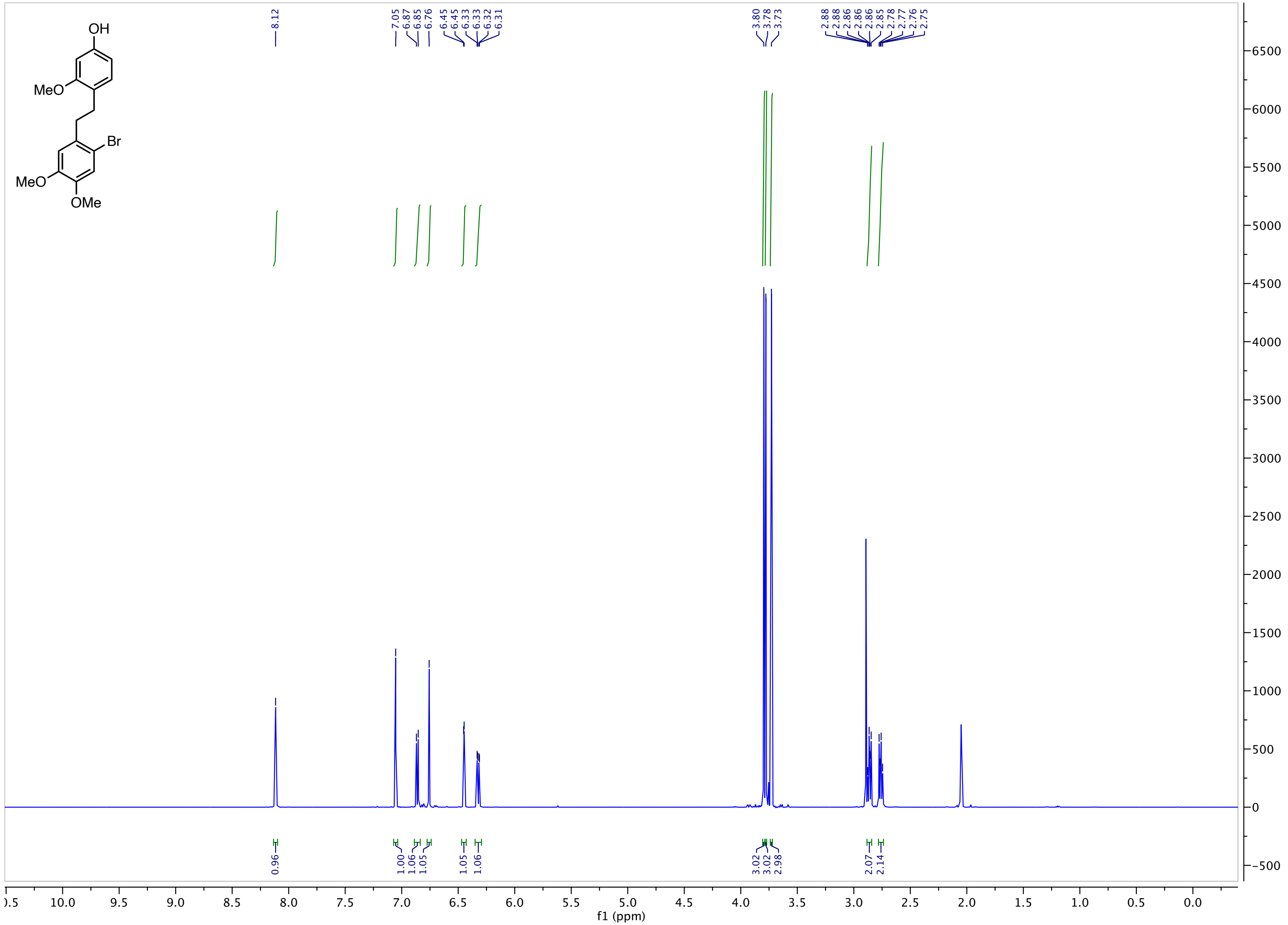

^{13}C NMR (Acetone-d_6): 4-(2-bromo-4,5-dimethoxyphenethyl)-3-Methoxyphenol **(1m)**

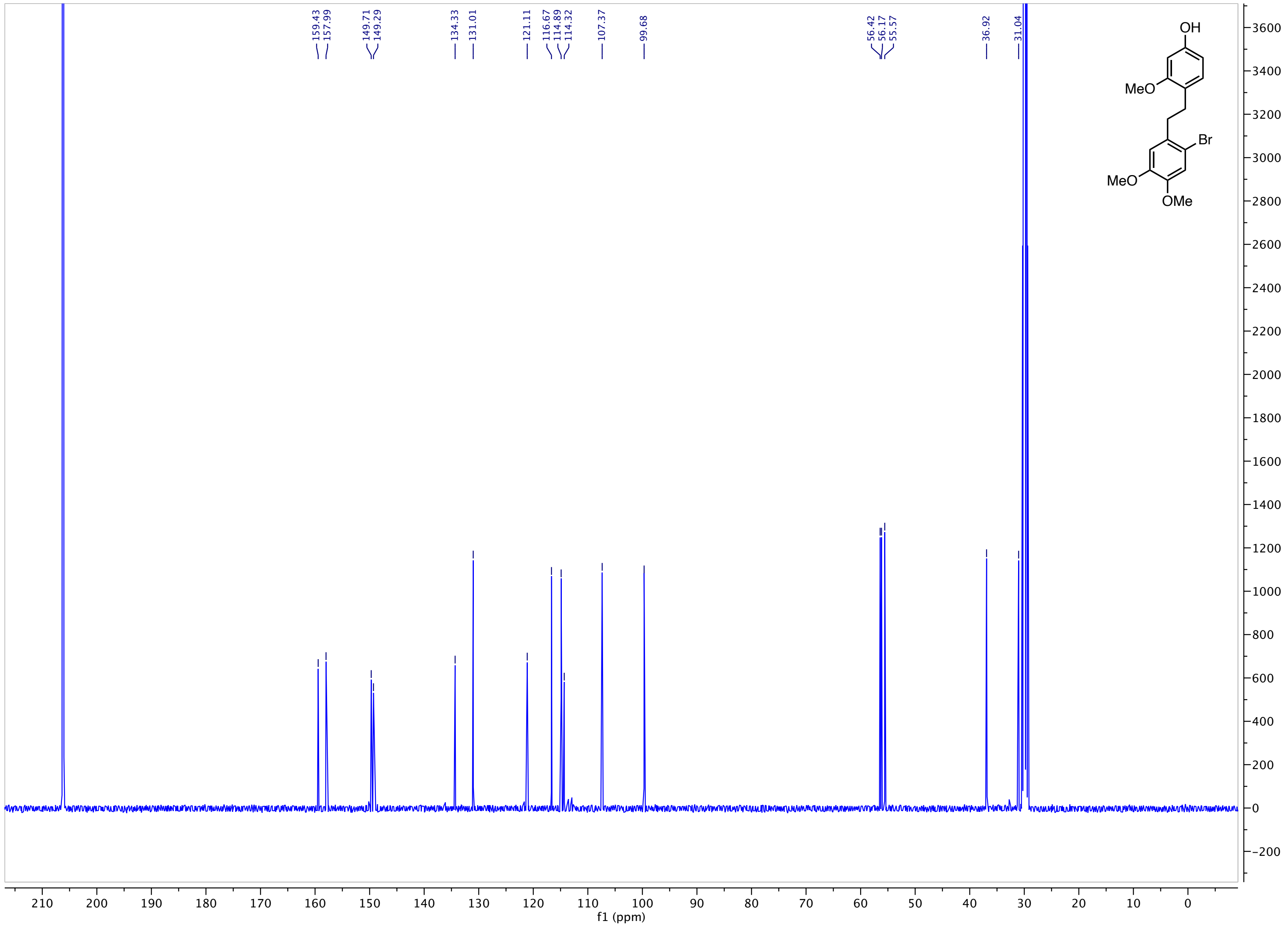

^{1}H NMR (MeCN-d_3): Bromo(2-bromo-6-chlorobenzyl)triphenylphosphonium bromide **(S17)**

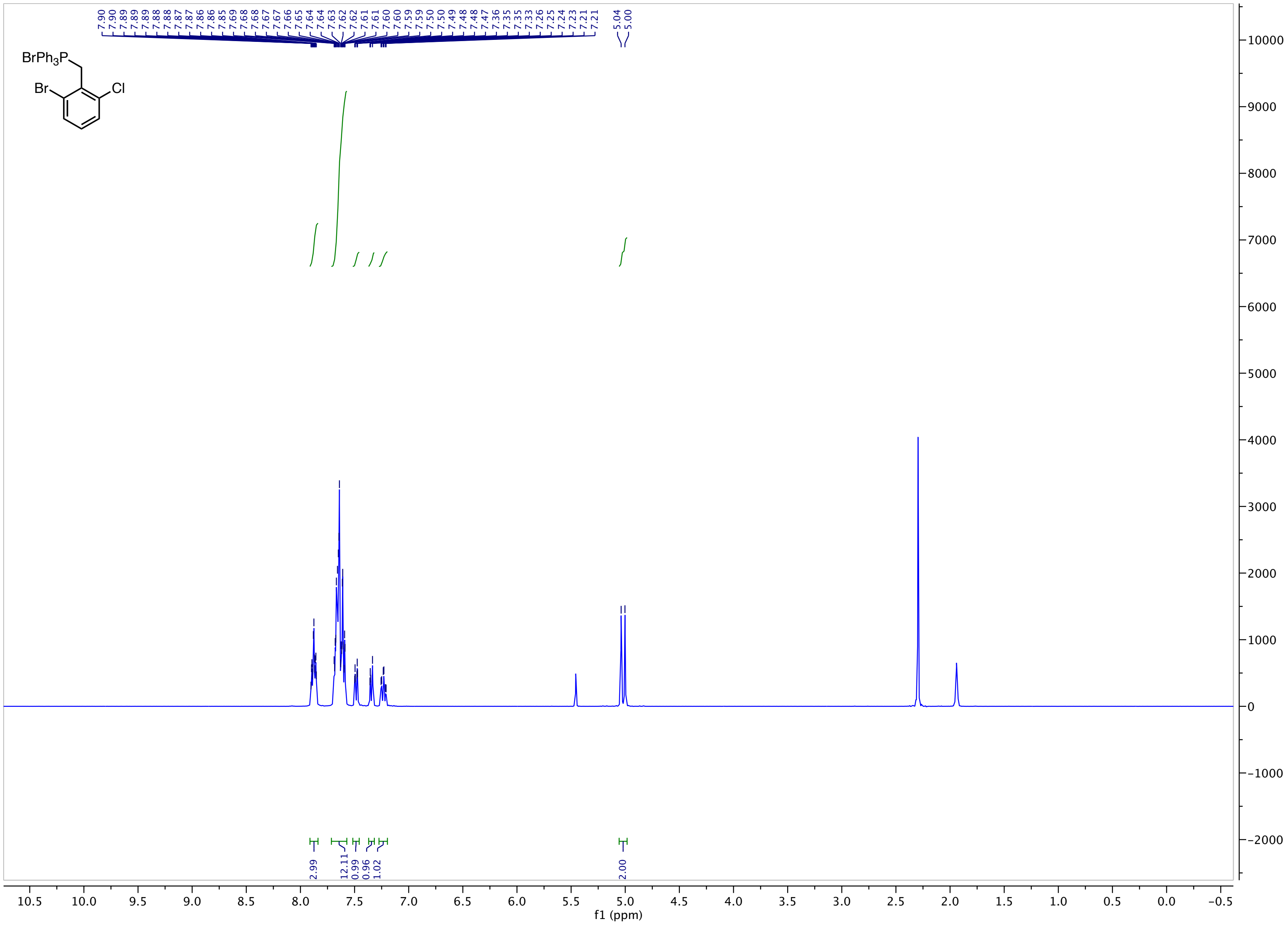

^{13}C NMR (MeCN-d_3): Bromo(2-bromo-6-chlorobenzyl)triphenylphosphonium bromide **(S17)**

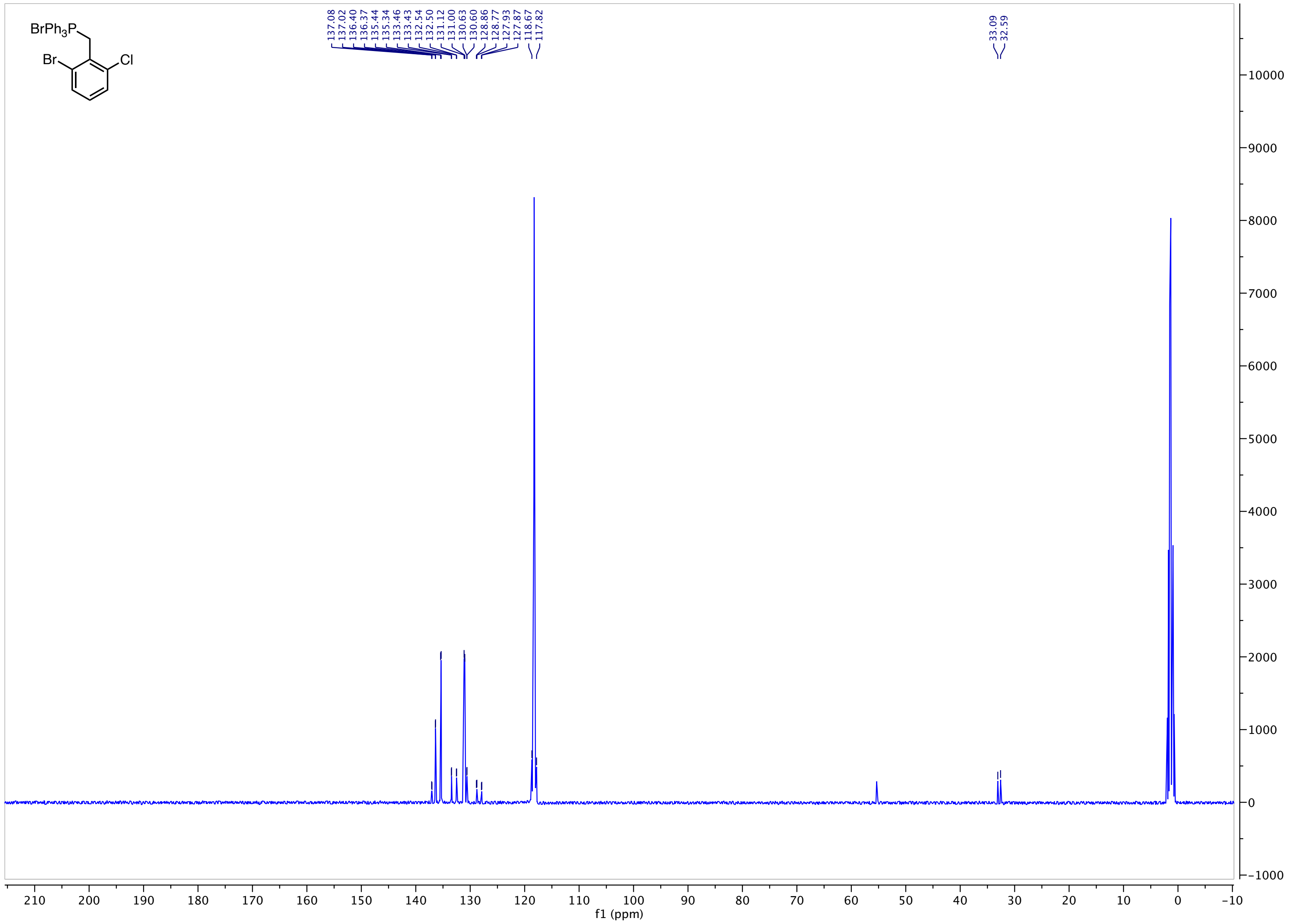

^{31}P NMR (MeCN-d_3): Bromo(2-bromo-6-chlorobenzyl)triphenylphosphonium bromide **(S17)**

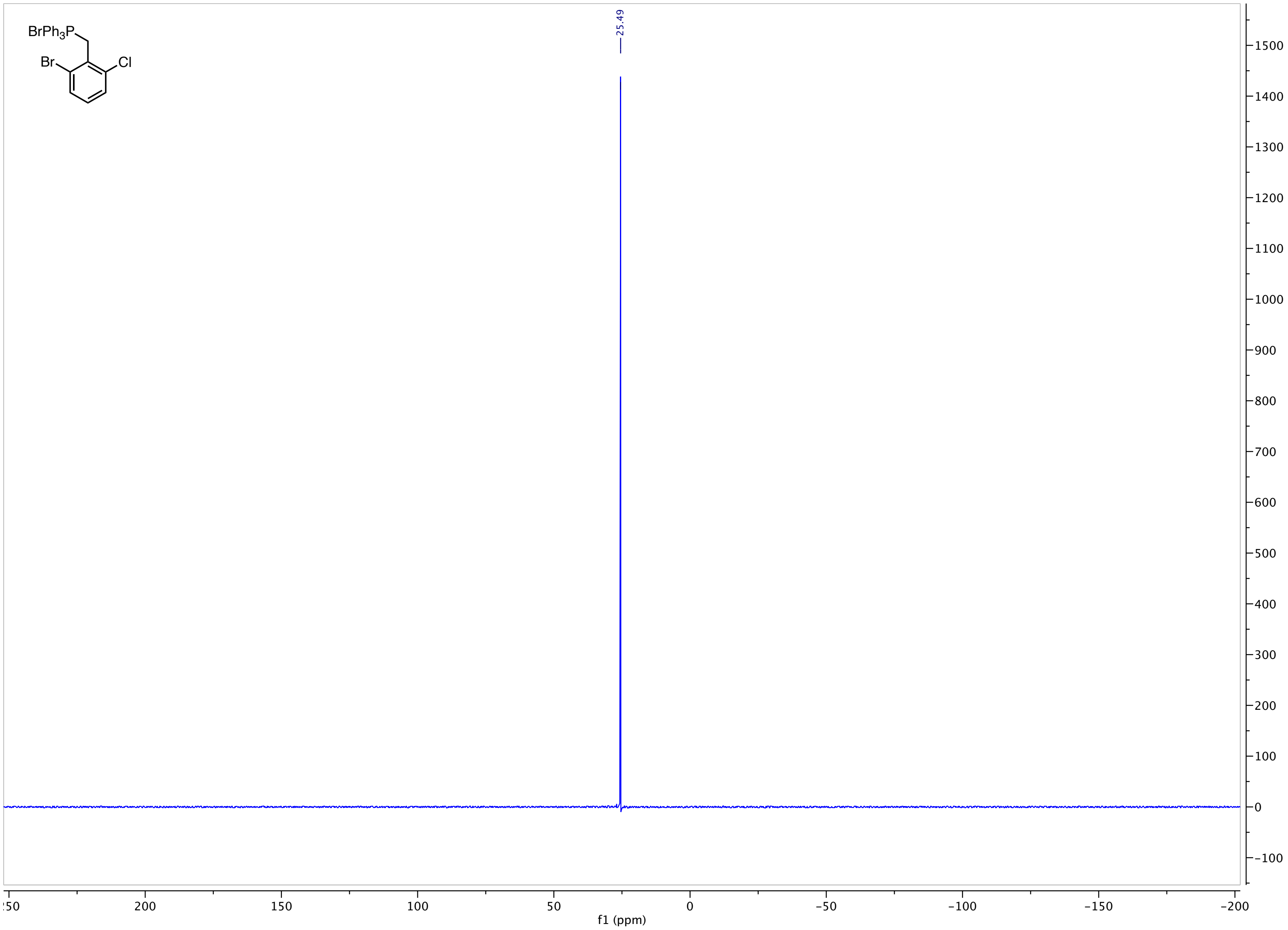

^{1}**H NMR** ($CDCl_3$): 4-(2-bromo-6-chlorophenethyl)-3-Methoxyphenol **(1n)**

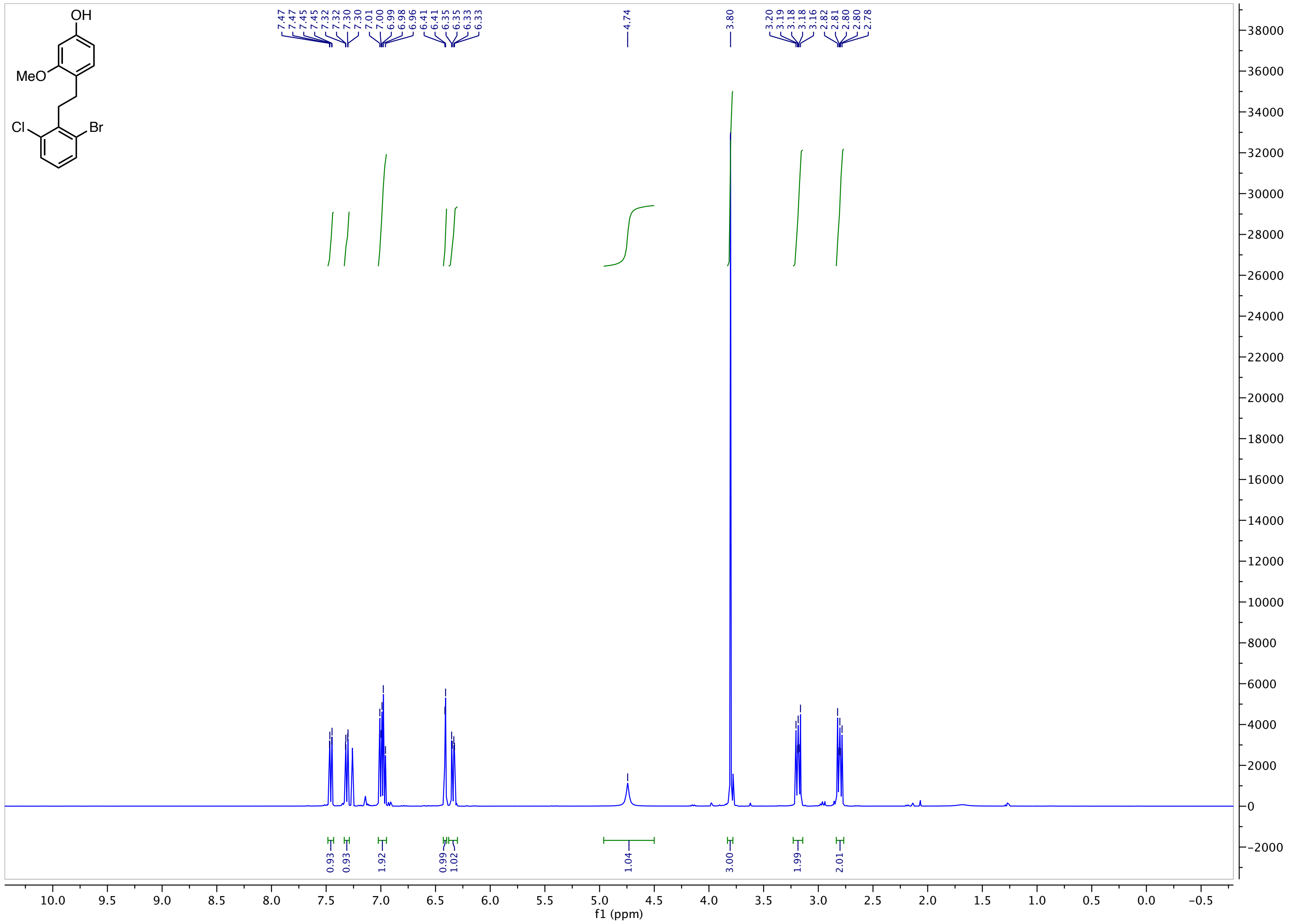

^{13}C NMR ($CDCl_3$): 4-(2-bromo-6-chlorophenethyl)-3-Methoxyphenol **(1n)**

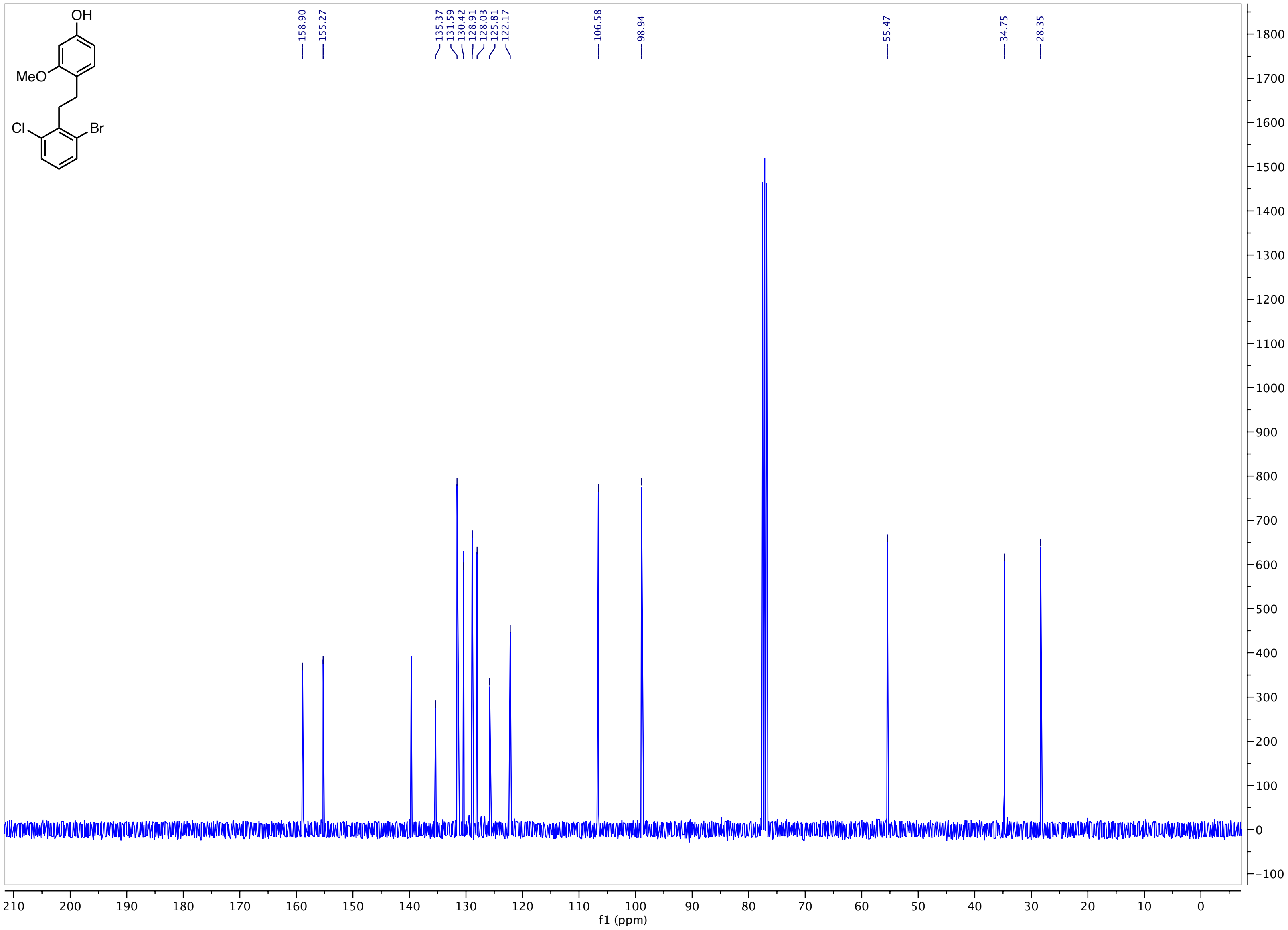

^{1}H NMR ($CDCl_3$): *tert*-Butyl (3-bromo-4-(bromomethyl)phenyl)carbamate **(S18)**

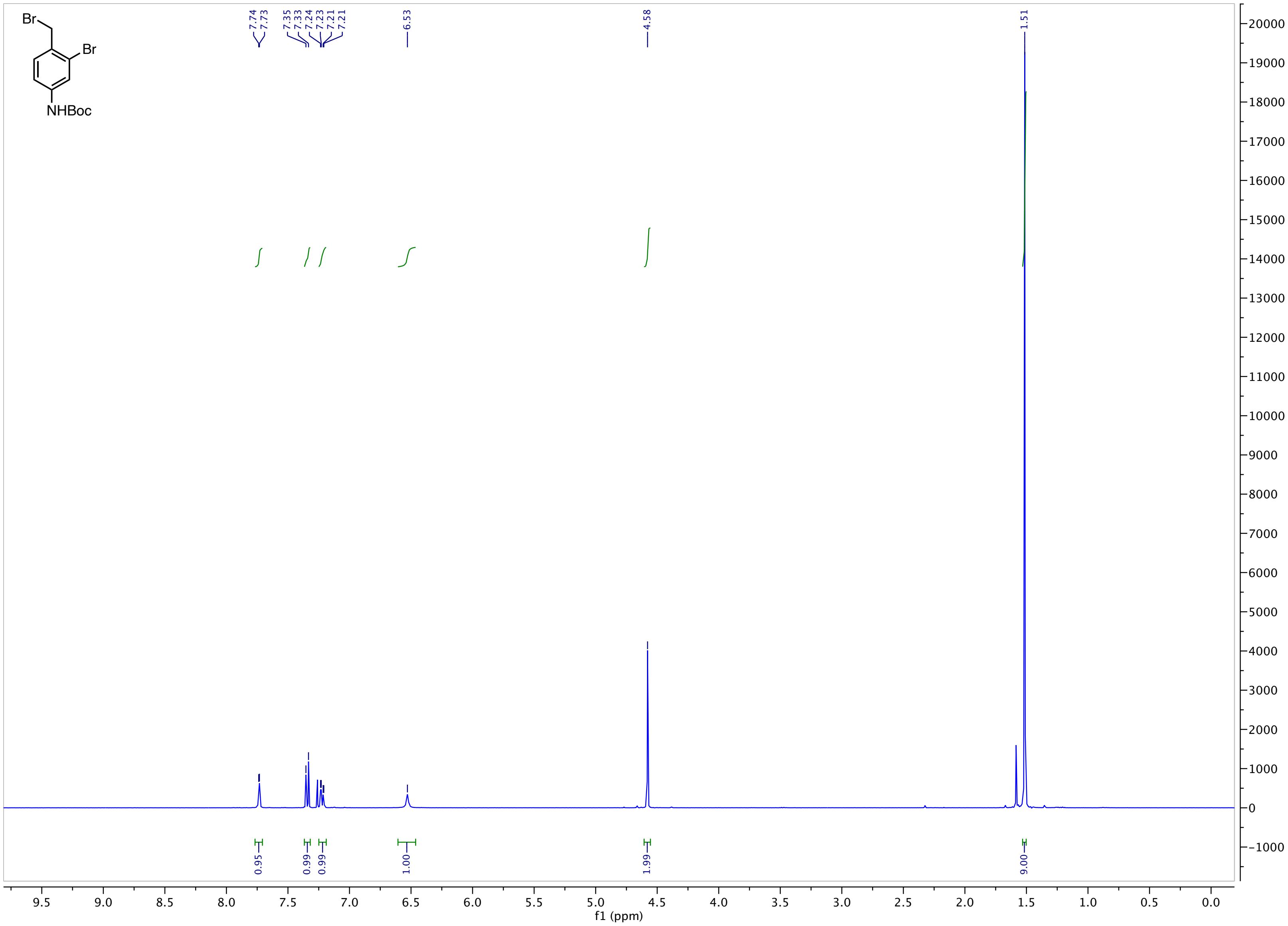

^{13}C NMR ($CDCl_3$): *tert*-Butyl (3-bromo-4-(bromomethyl)phenyl)carbamate **(S18)**

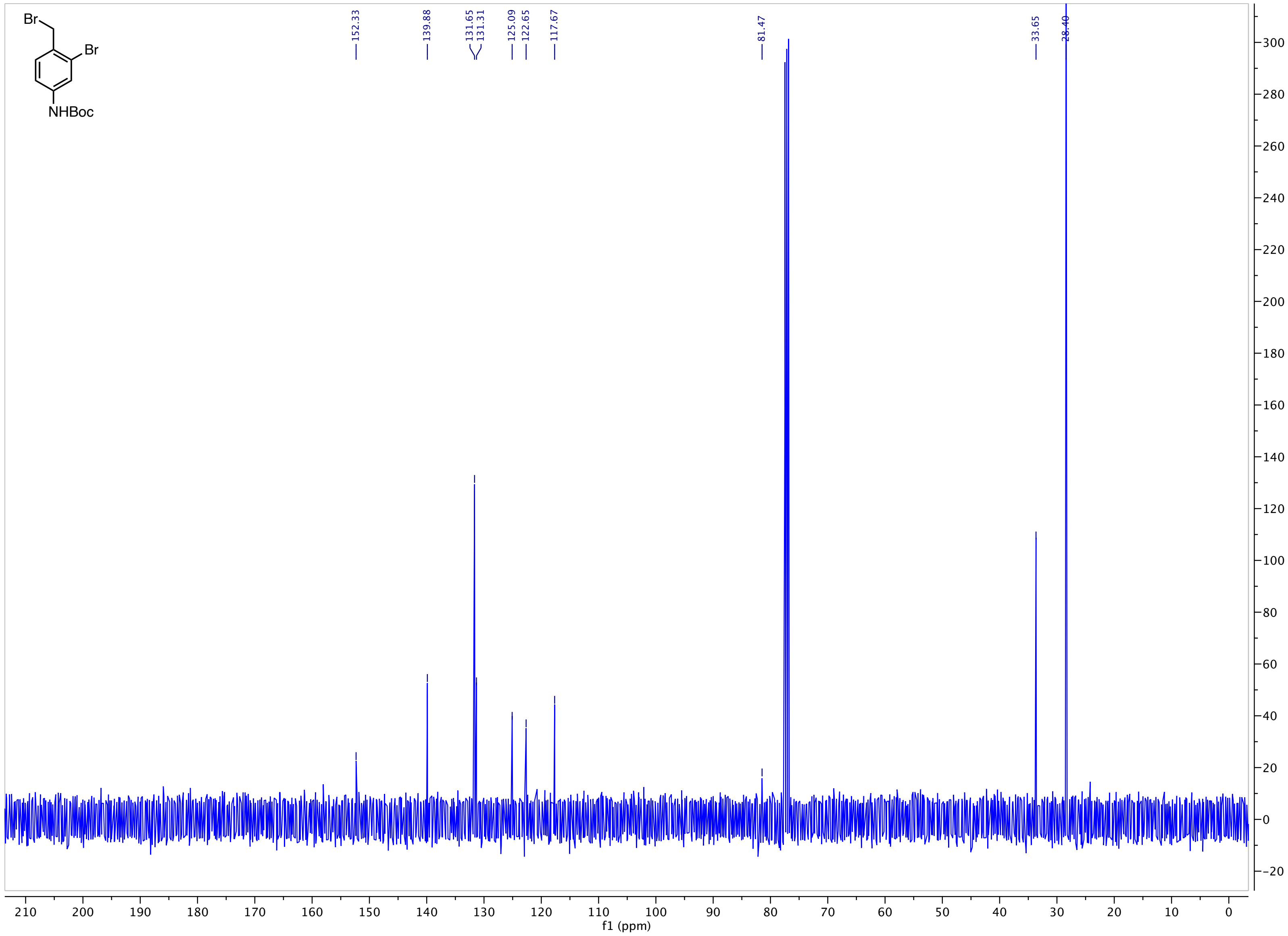

^{1}**H NMR** ($CDCl_3$): *tert*-Butyl (3-bromo-4-(4-hydroxy-2-methoxyphenethyl)phenyl)carbamate **(1o)**

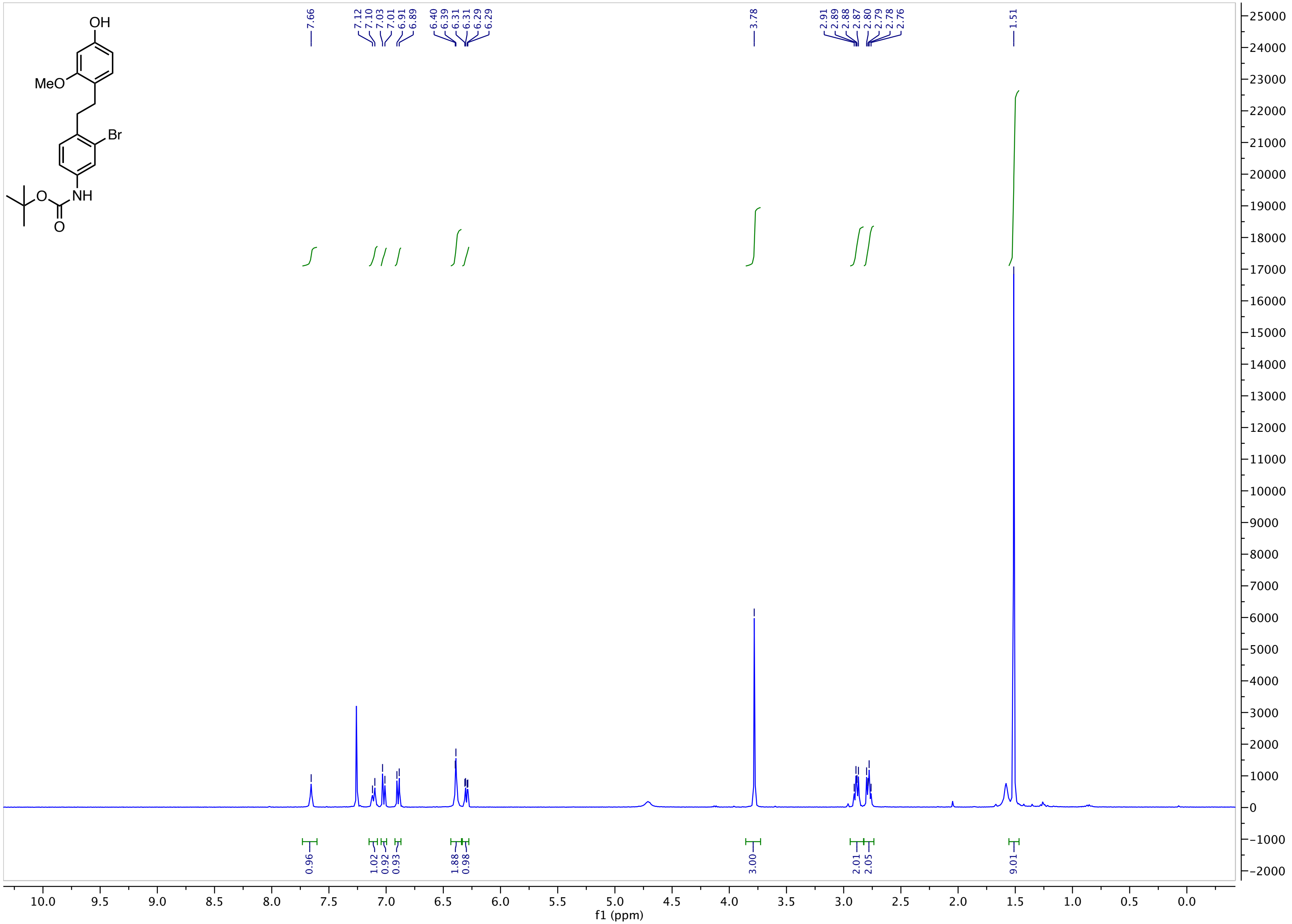

^{13}C NMR (Acetone-d_6): *tert*-Butyl (3-bromo-4-(4-hydroxy-2-methoxyphenethyl)phenyl)carbamate **(1o)**

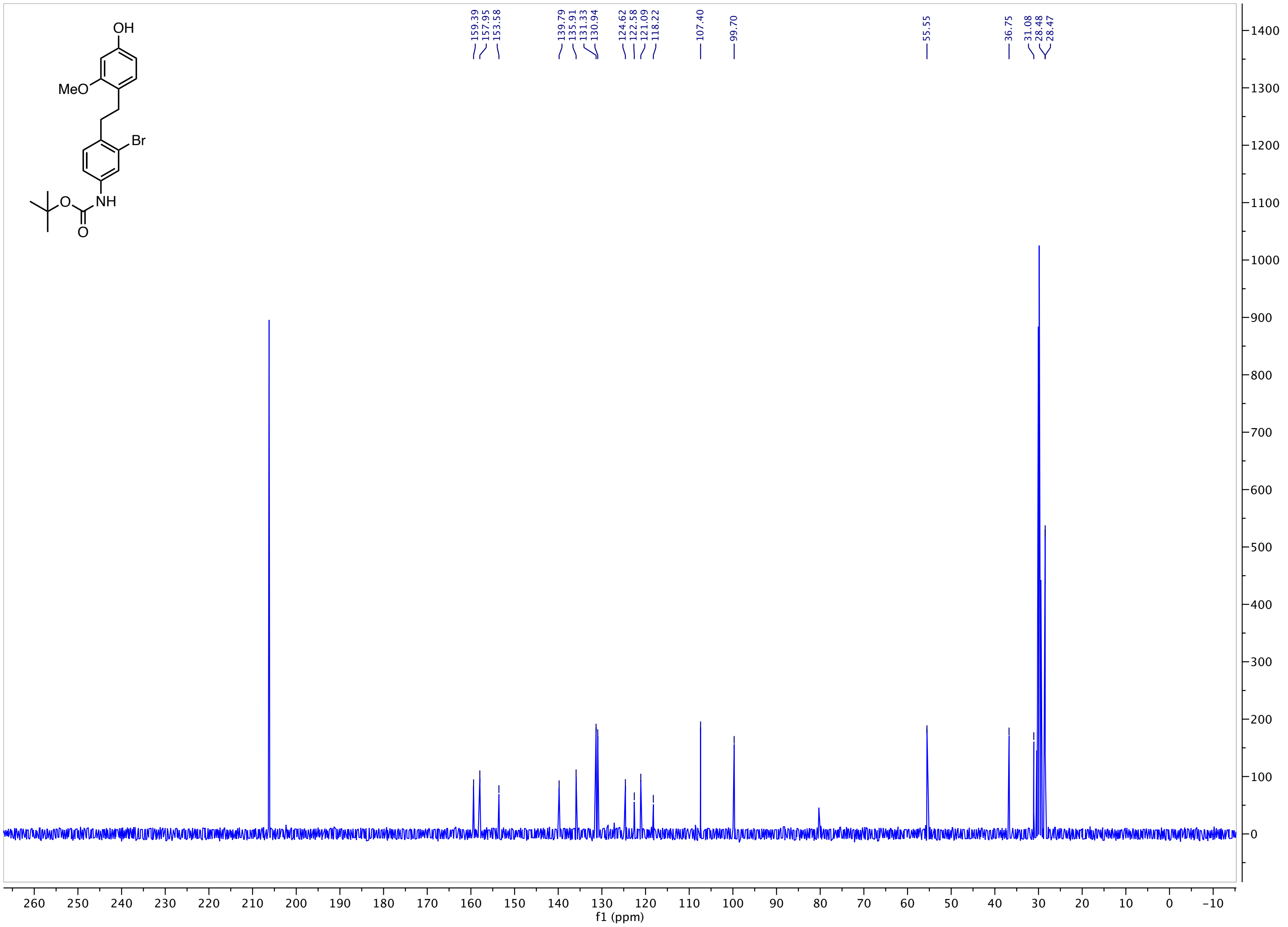

^{1}H NMR ($CDCl_3$): *tert*-Butyl 3-bromo-4-methylbenzoate (S19)

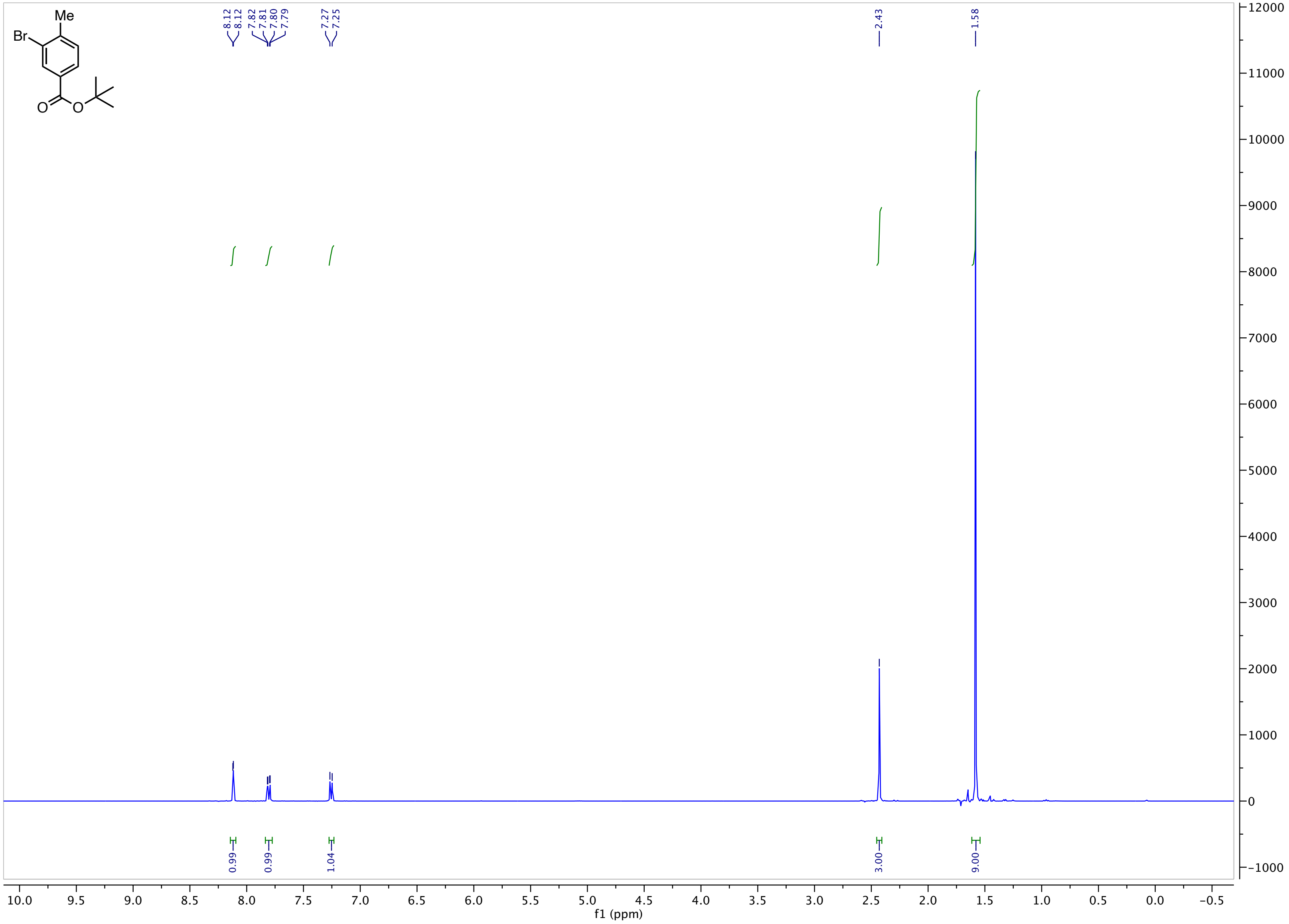

^{13}C NMR ($CDCl_3$): *tert*-Butyl 3-bromo-4-methylbenzoate (**S19**)

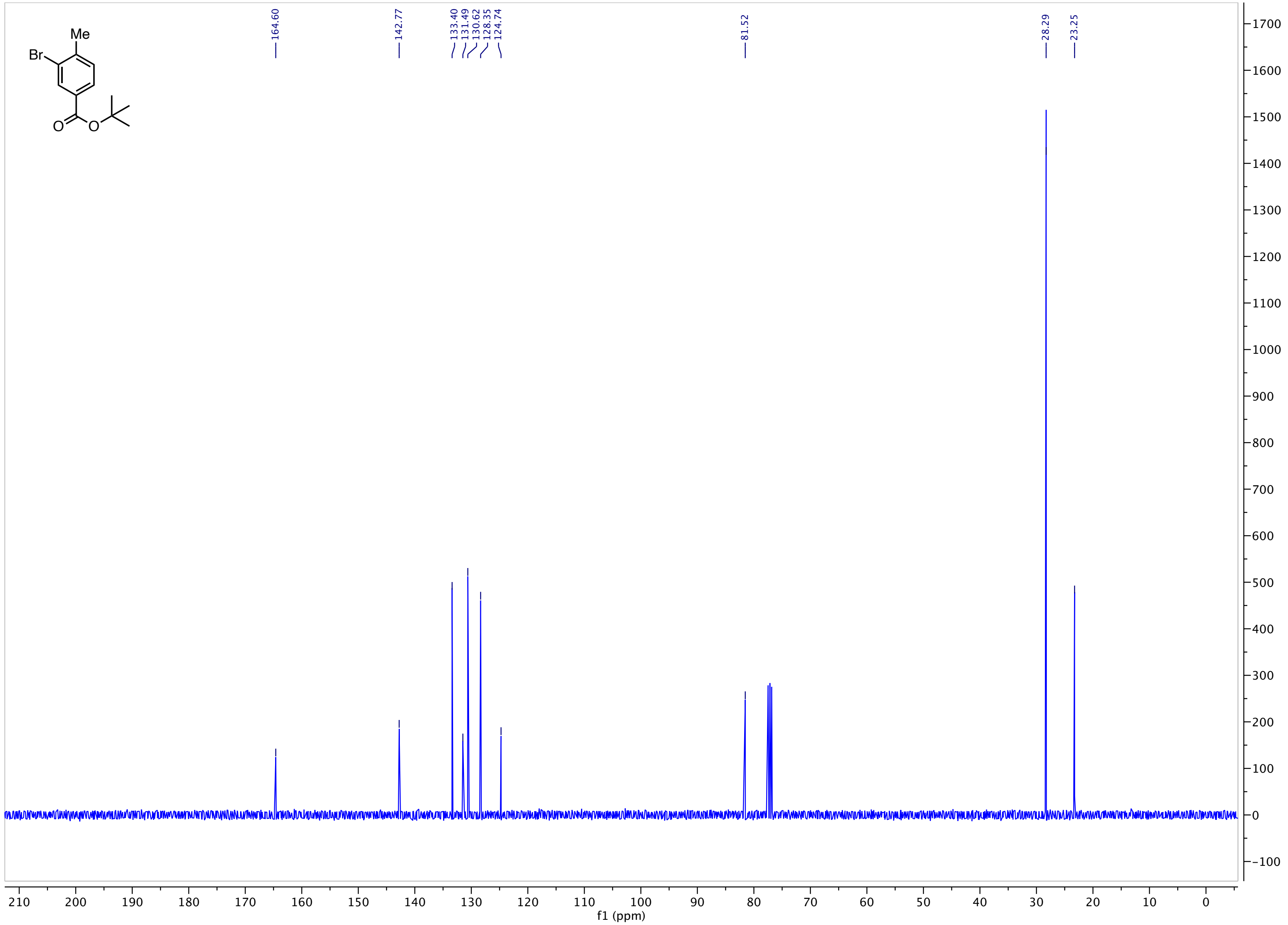

^{1}H NMR ($CDCl_3$): (2-bromo-4-(*tert*-butoxycarbonyl)benzyl)Triphenylphosphonium bromide (**S20**)

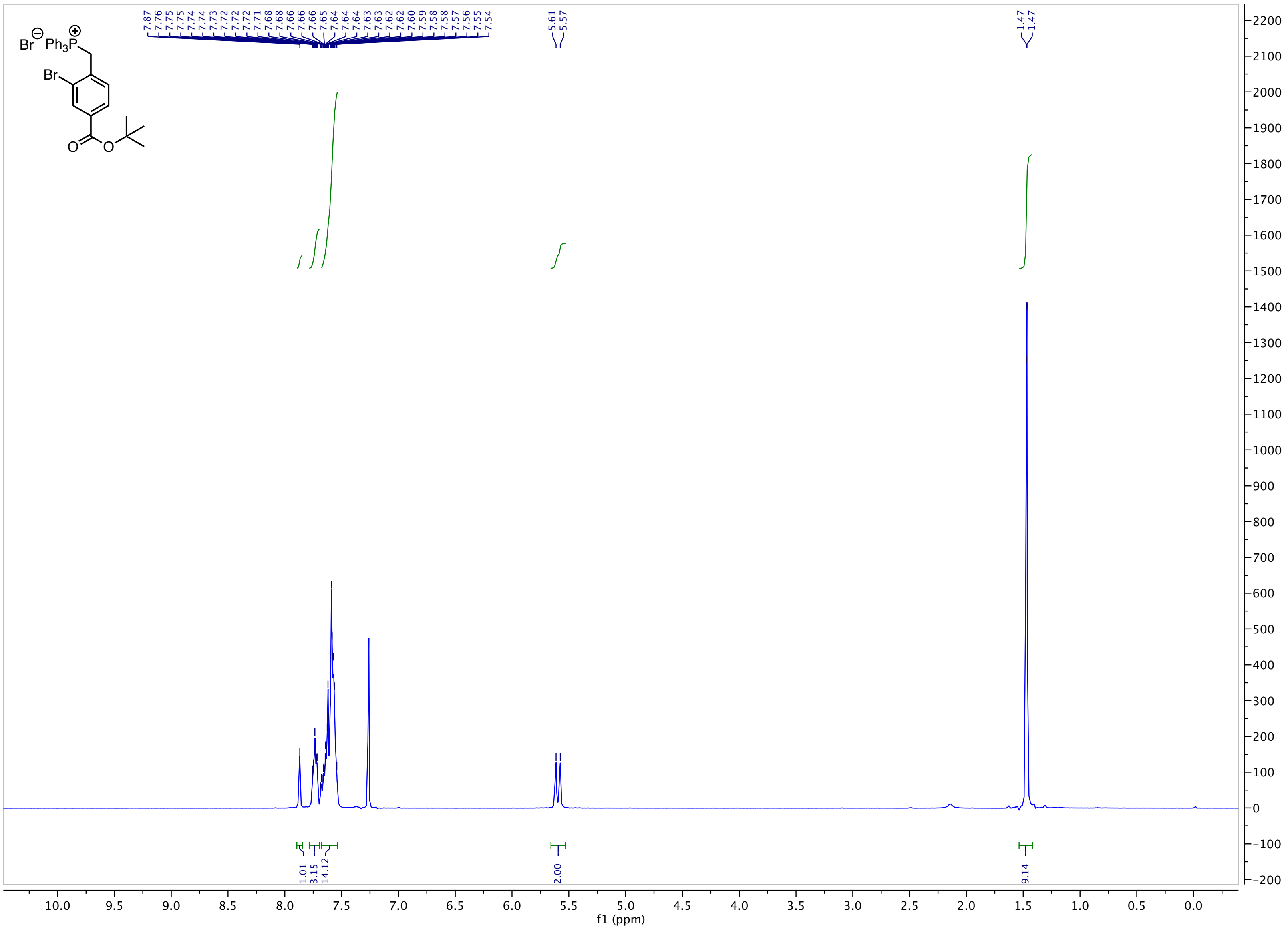

^{13}C NMR ($CDCl_3$): (2-bromo-4-(*tert*-butoxycarbonyl)benzyl)Triphenylphosphonium bromide (**S20**)

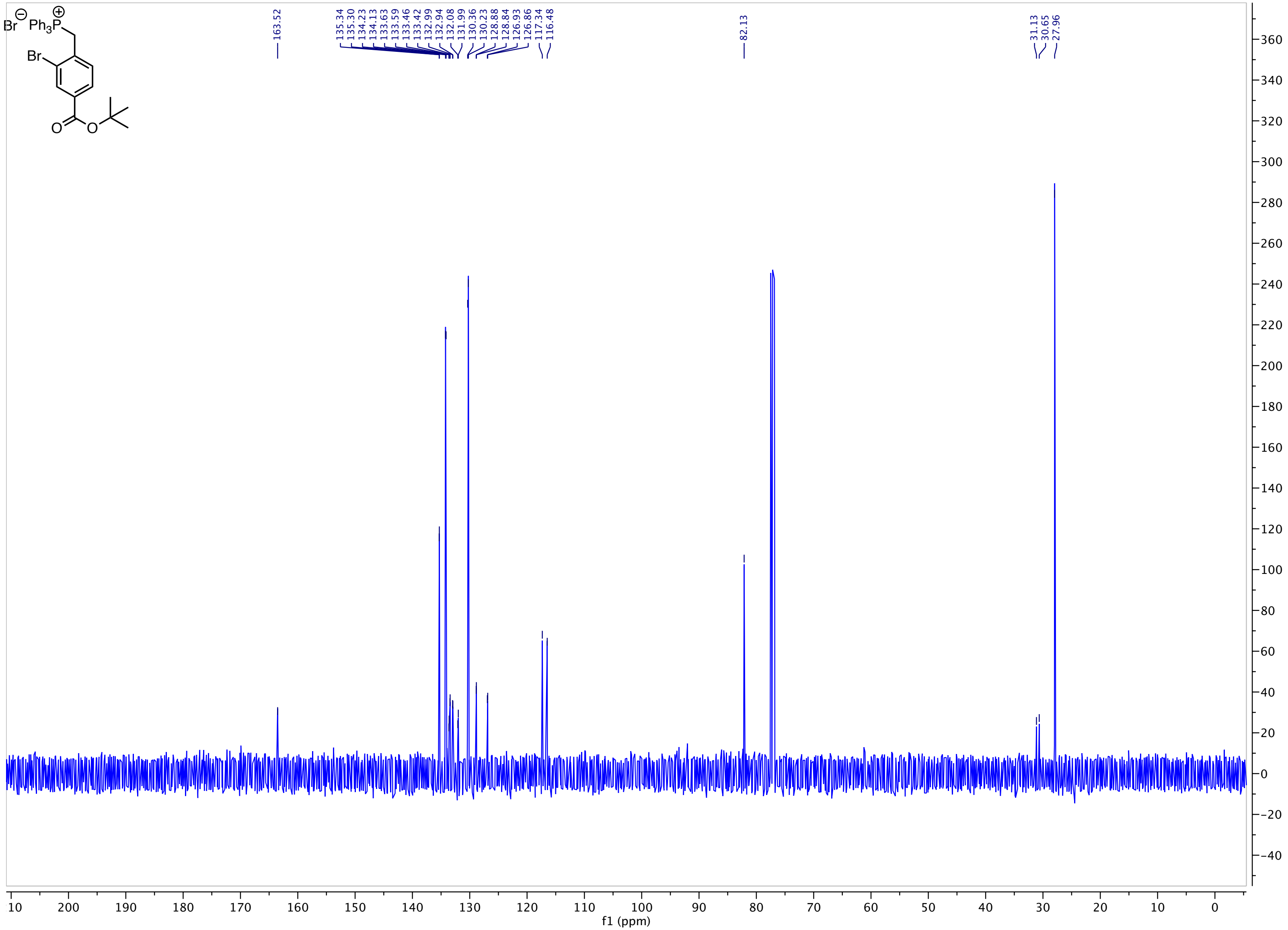

^{31}**P NMR** (CDCl$_3$): (2-bromo-4-(*tert*-butoxycarbonyl)benzyl)Triphenylphosphonium bromide (**S20**)

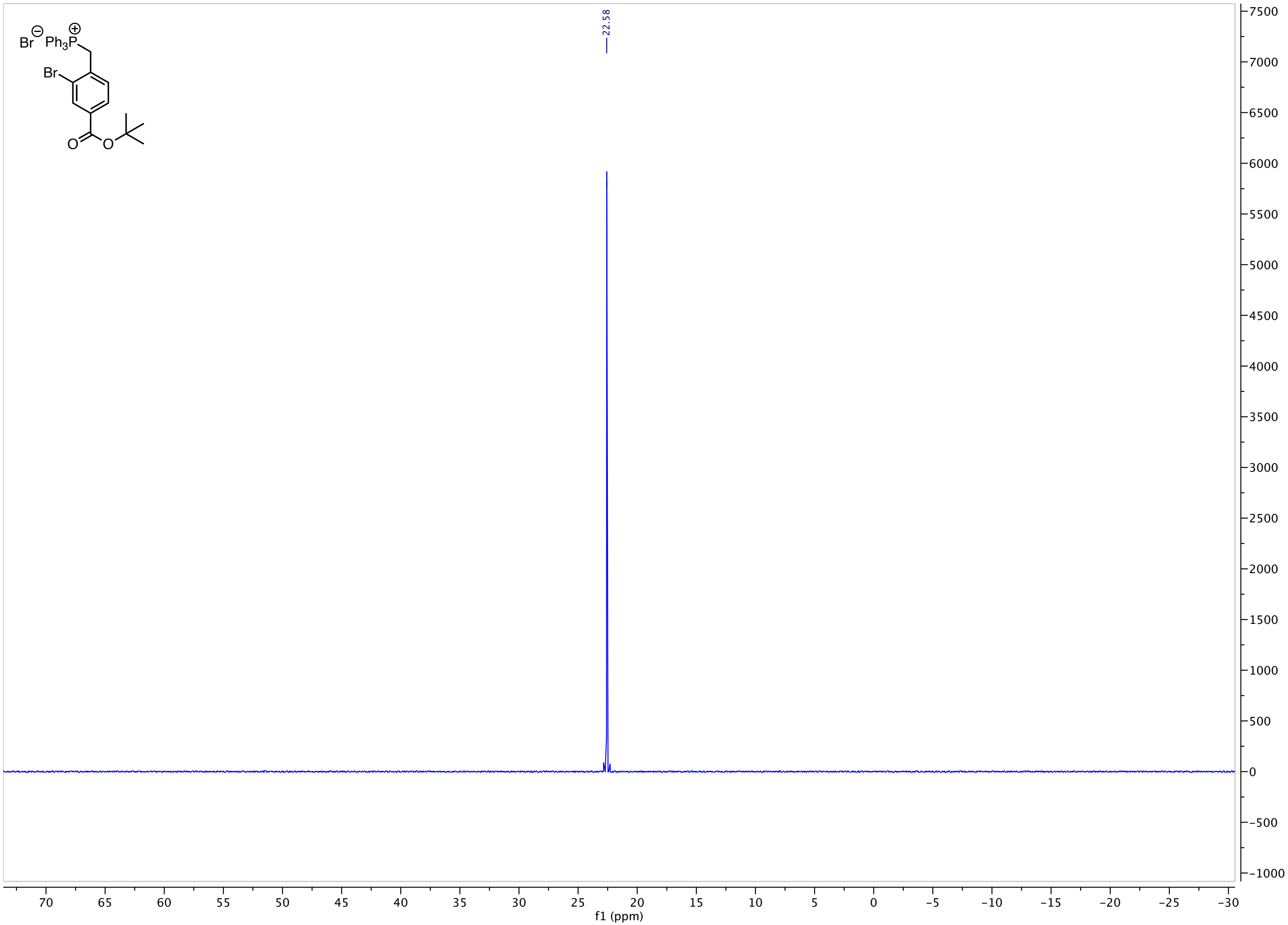

^{1}H NMR ($CDCl_3$): *tert*-Butyl 3-bromo-4-(4-hydroxy-2-methoxyphenethyl)benzoate (**1p**)

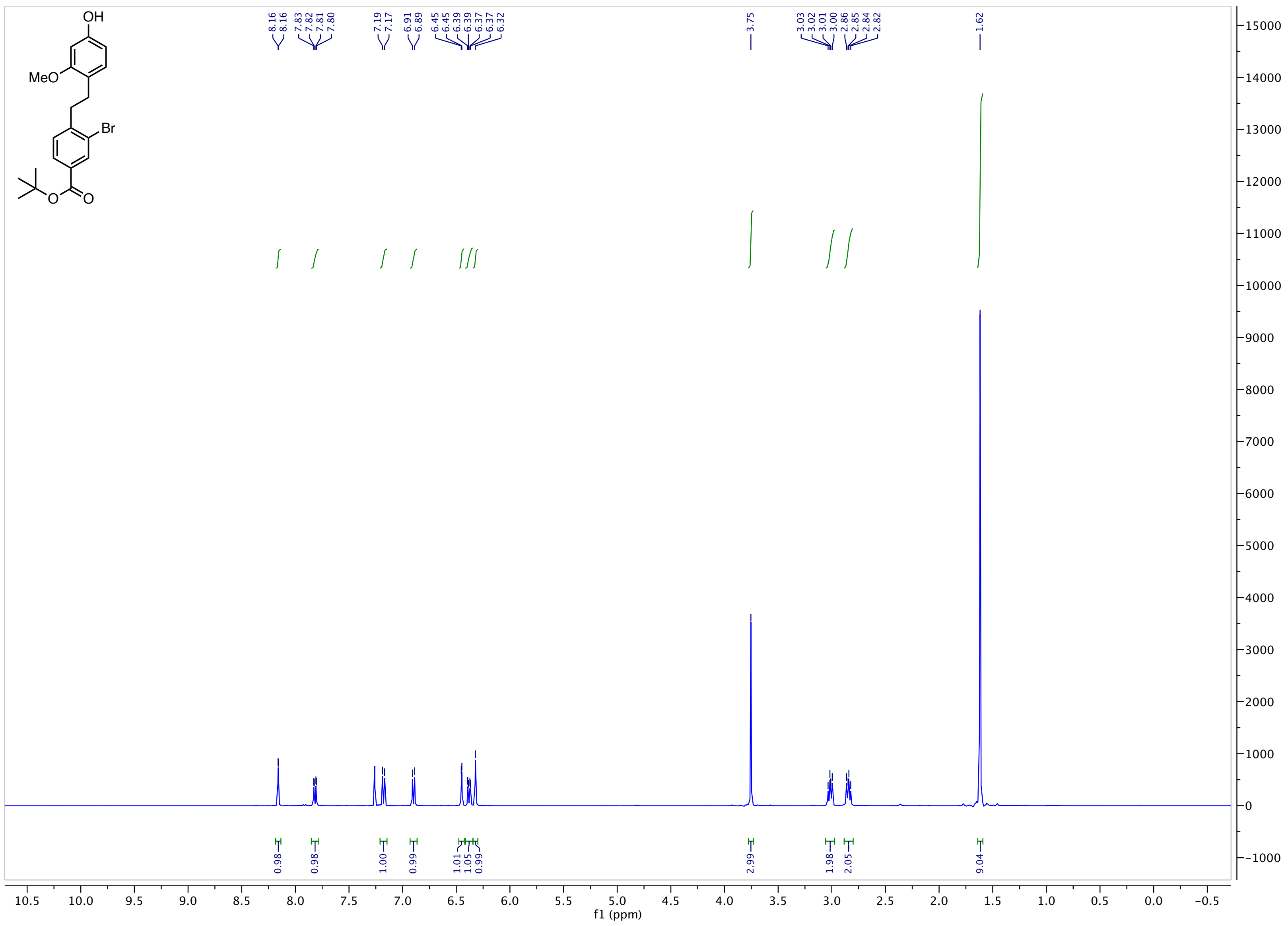

^{13}C NMR ($CDCl_3$): *tert*-Butyl 3-bromo-4-(4-hydroxy-2-methoxyphenethyl)benzoate (**1p**)

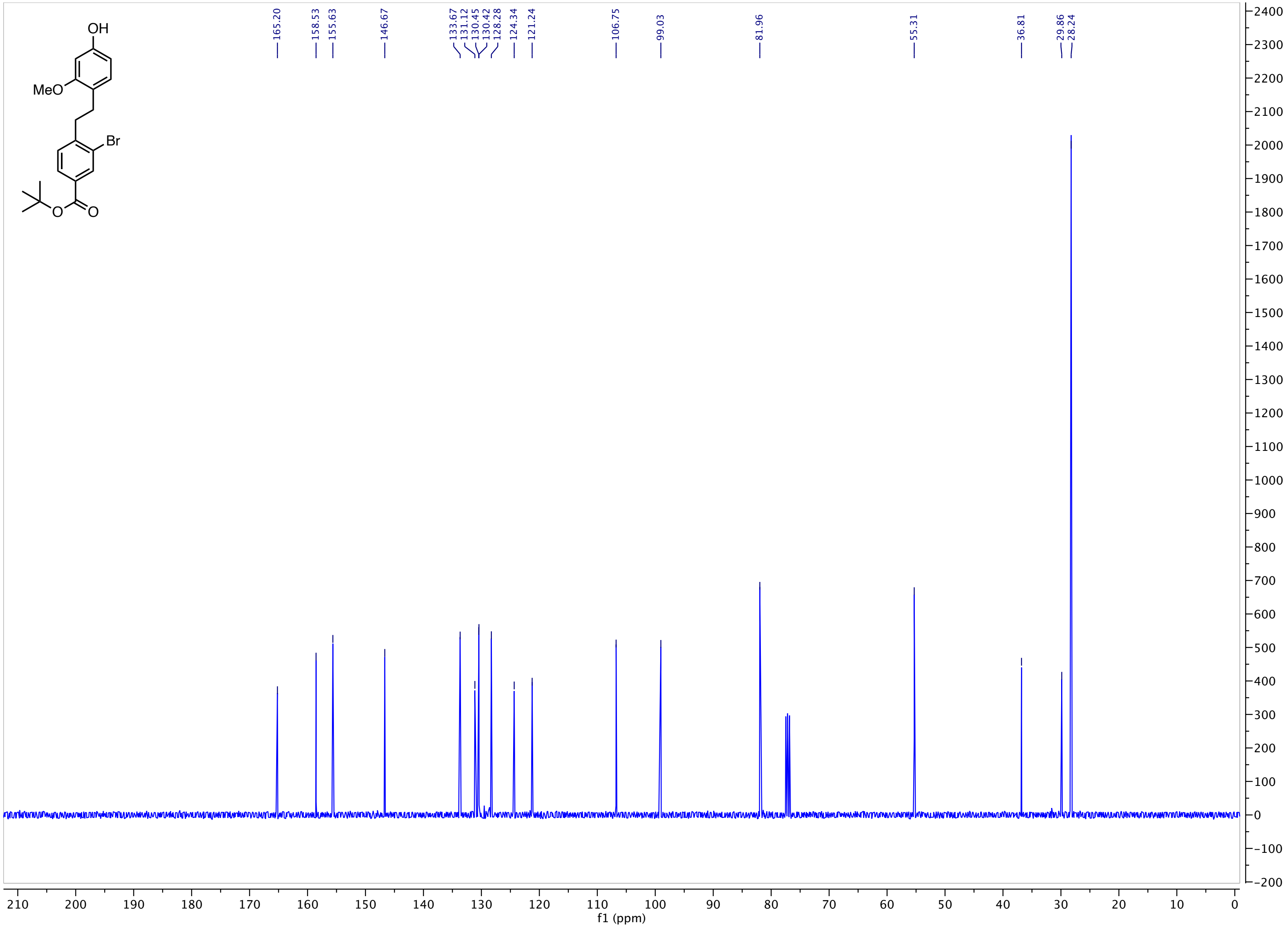

^{1}H NMR ($CDCl_3$): (2-bromo-3-methylphenyl)Methanol **(S21)**

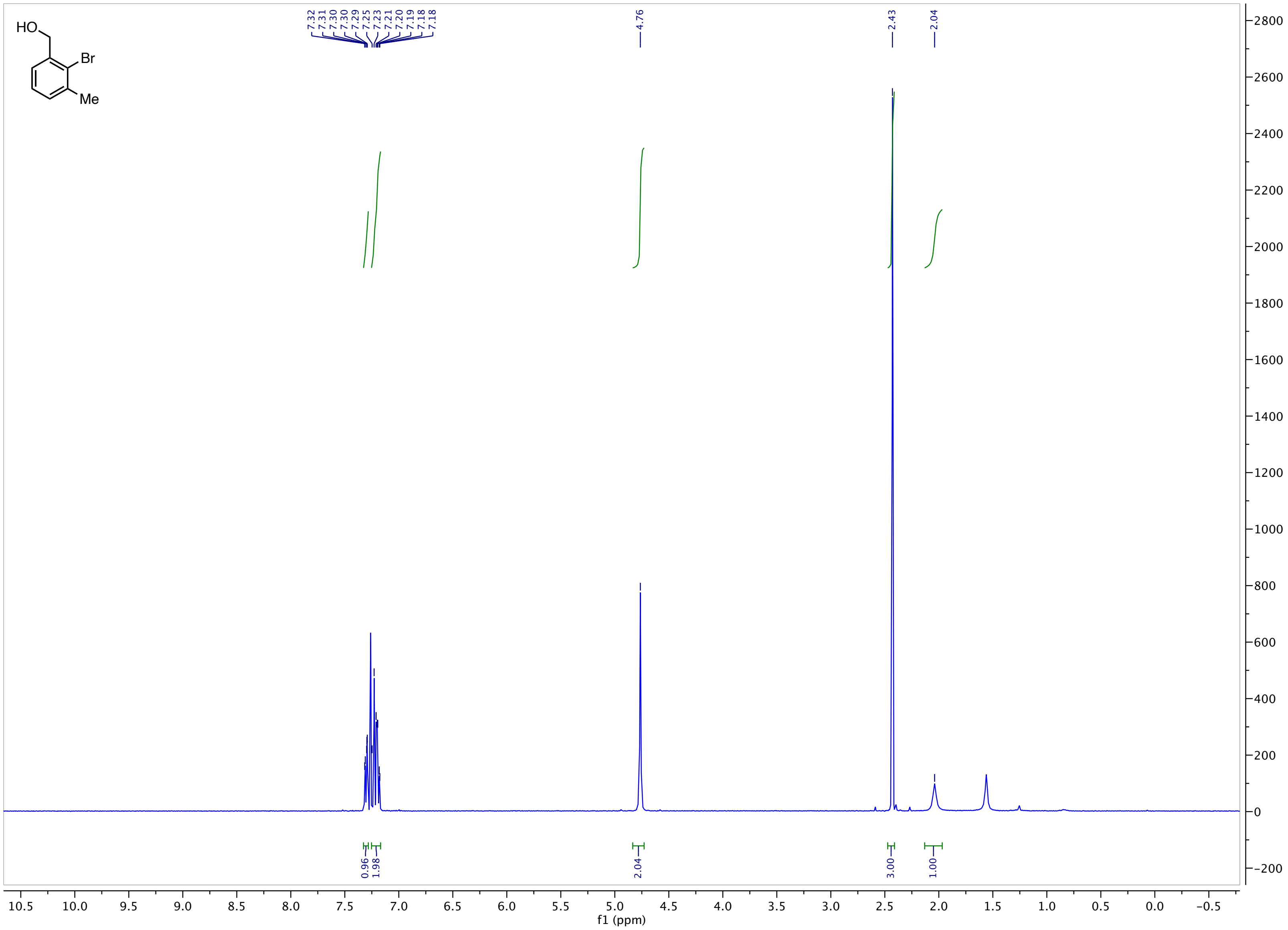

^{13}C NMR ($CDCl_3$): (2-bromo-3-methylphenyl)Methanol **(S21)**

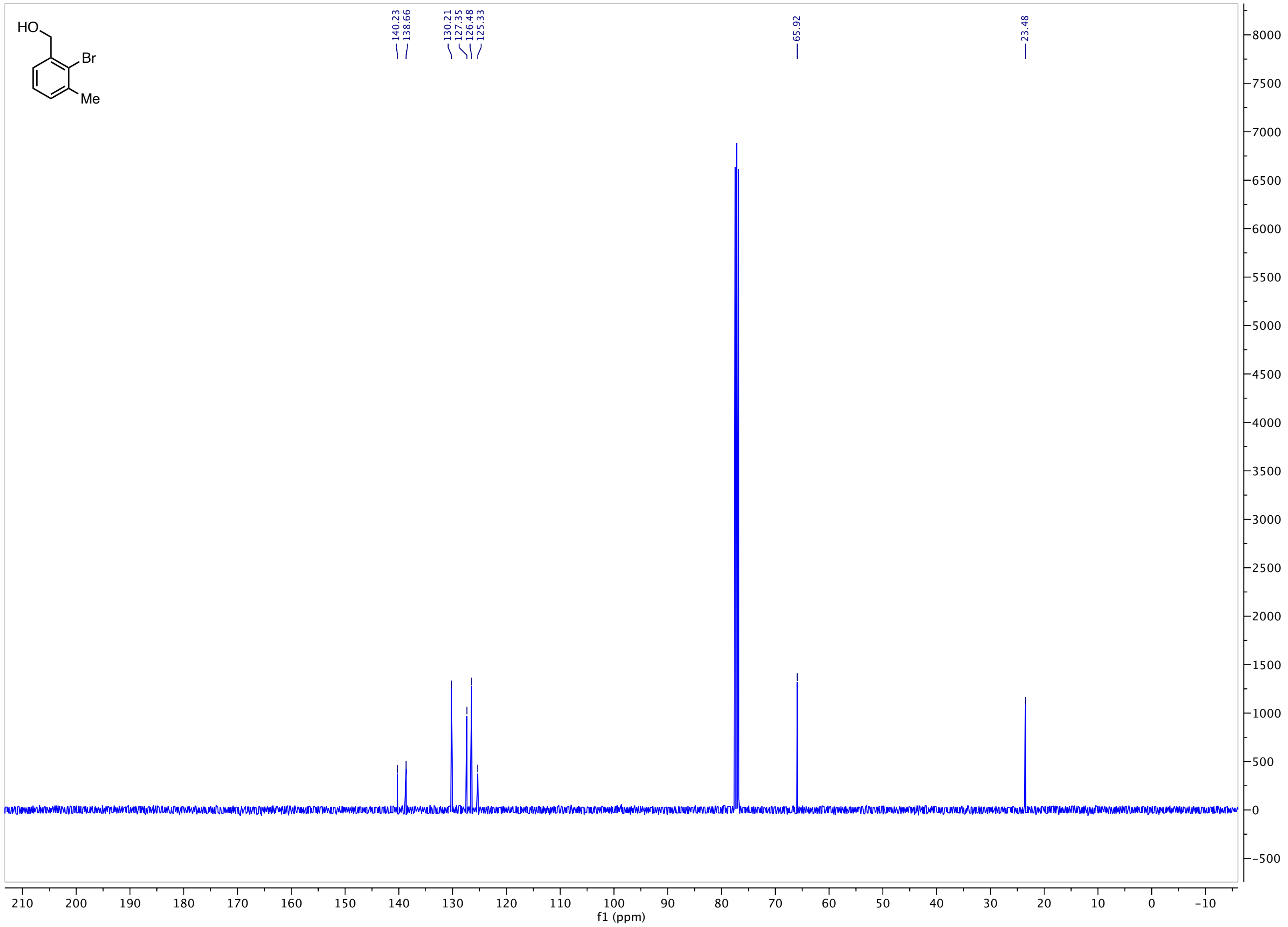

^{1}H NMR ($CDCl_3$): (2-bromo-3-methylbenzyl)Triphenylphosphonium bromide (S22)

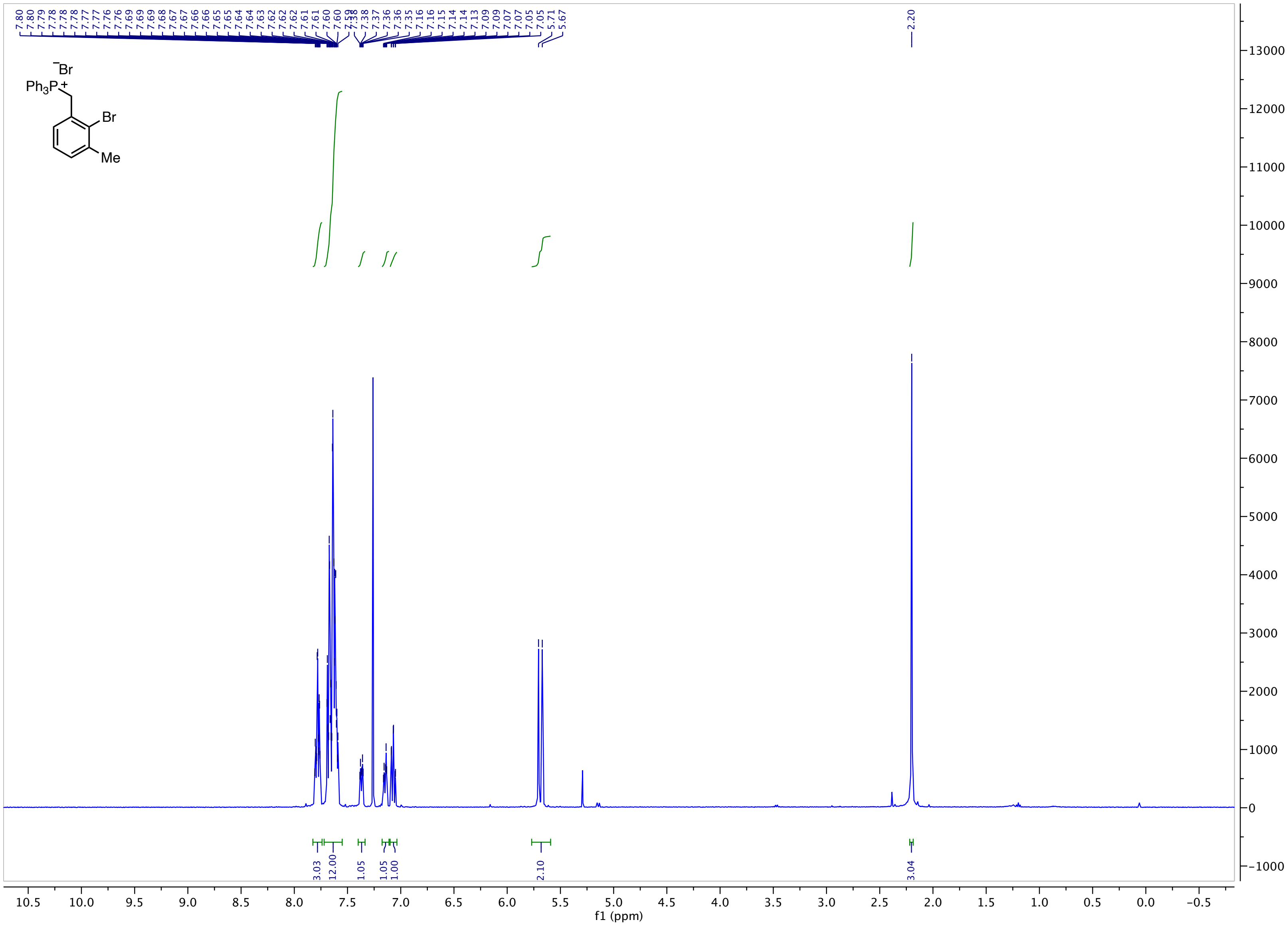

^{13}C NMR ($CDCl_3$): (2-bromo-3-methylbenzyl)Triphenylphosphonium bromide (S22)

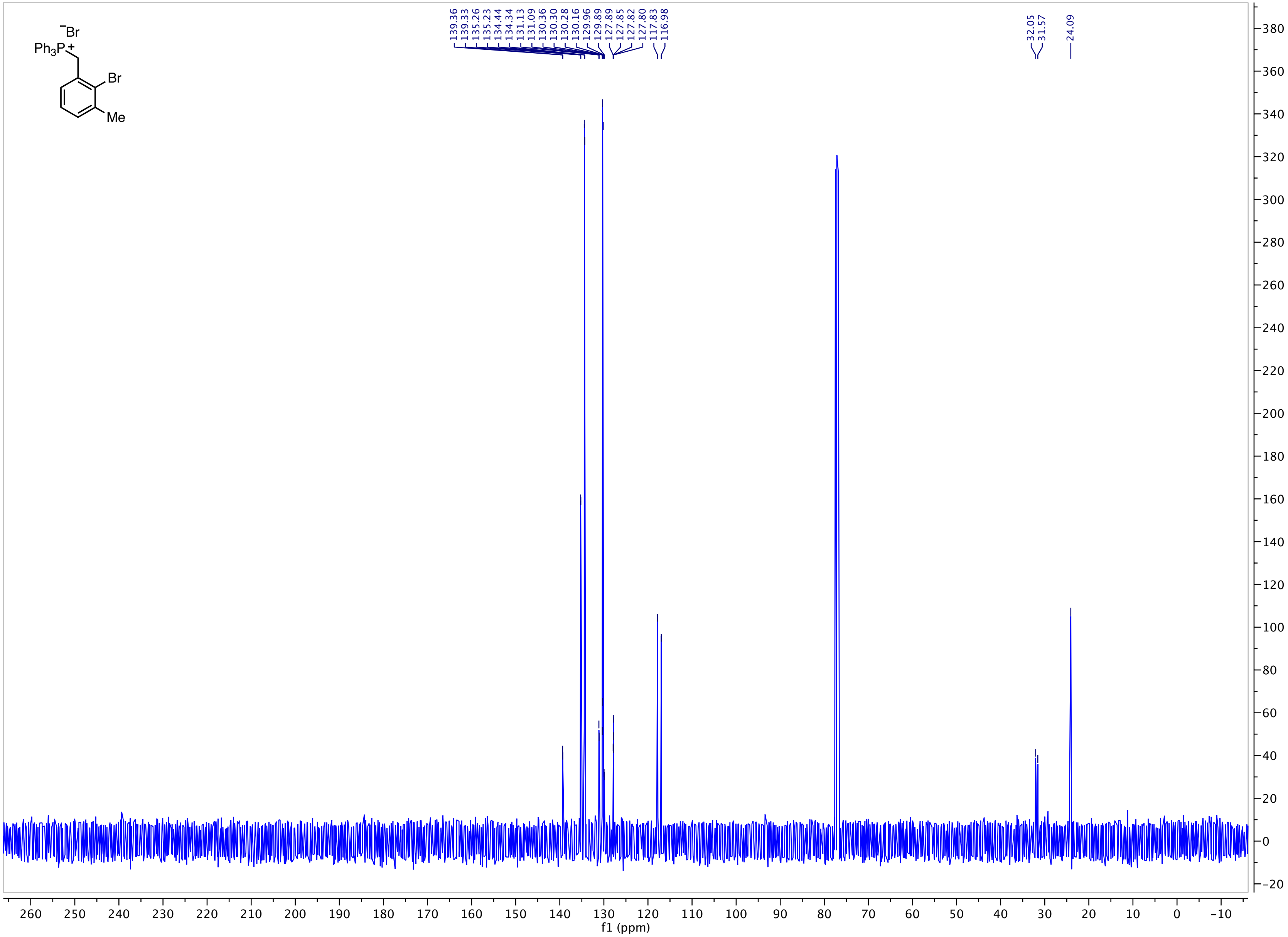

^{31}**P NMR** (CDCl$_3$): (2-bromo-3-methylbenzyl)Triphenylphosphonium bromide **(S22)**

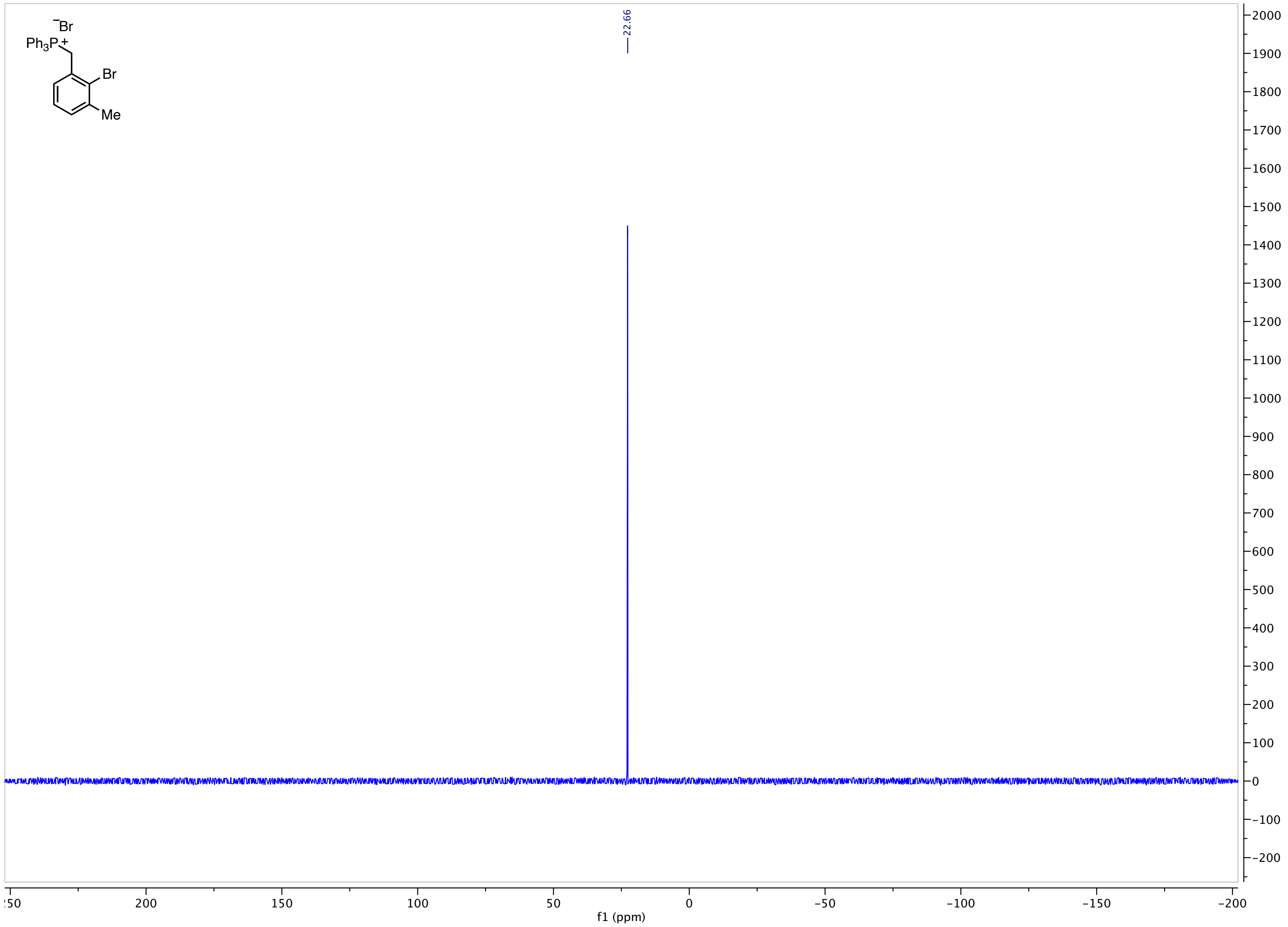

^{1}H NMR ($CDCl_3$): 1-(4-(benzyloxy)-2-methoxyphenethyl)-2-Bromo-3-methylbenzene (1q)

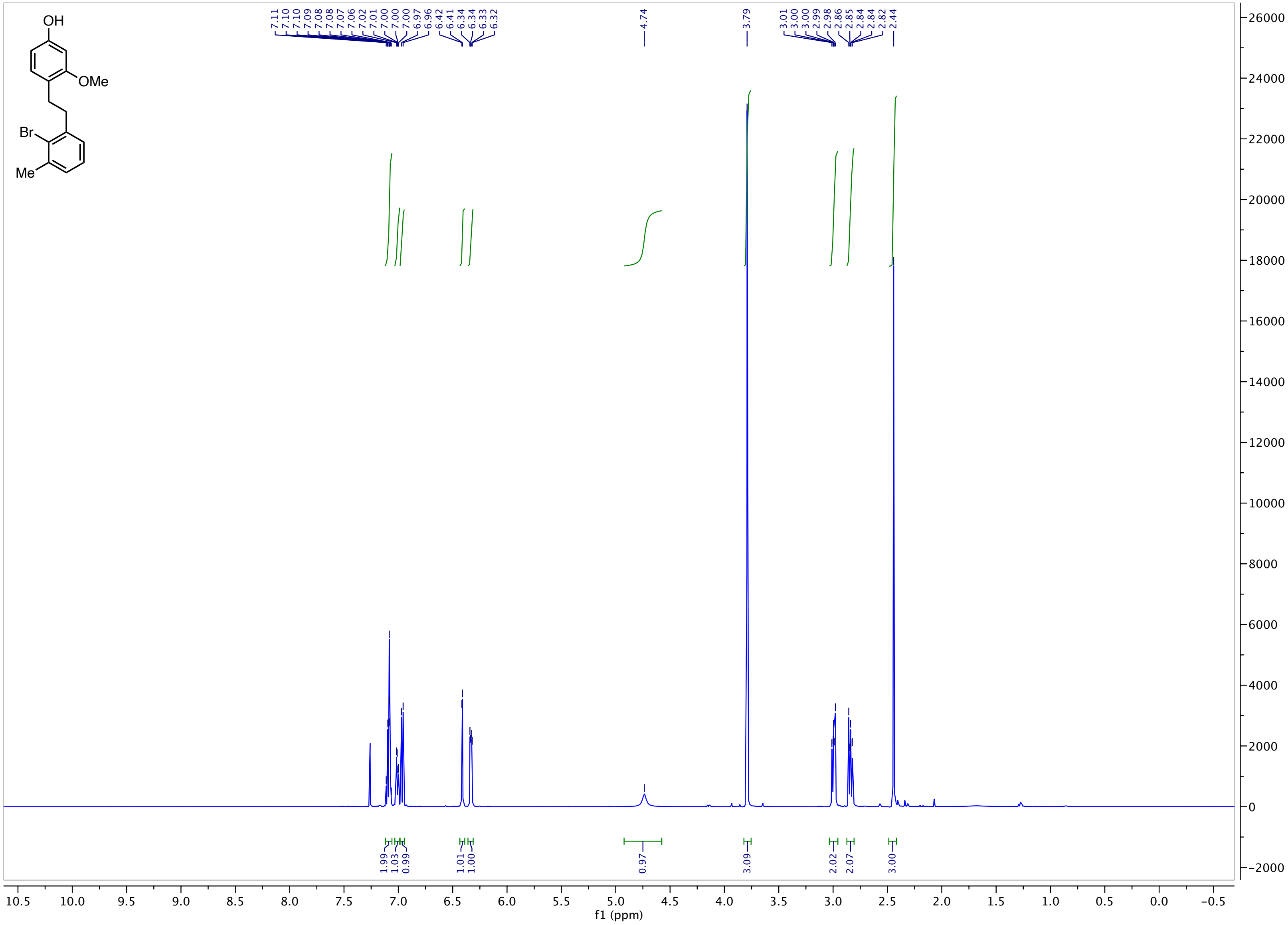

^{13}C NMR ($CDCl_3$): 1-(4-(benzyloxy)-2-methoxyphenethyl)-2-Bromo-3-methylbenzene **(1q)**

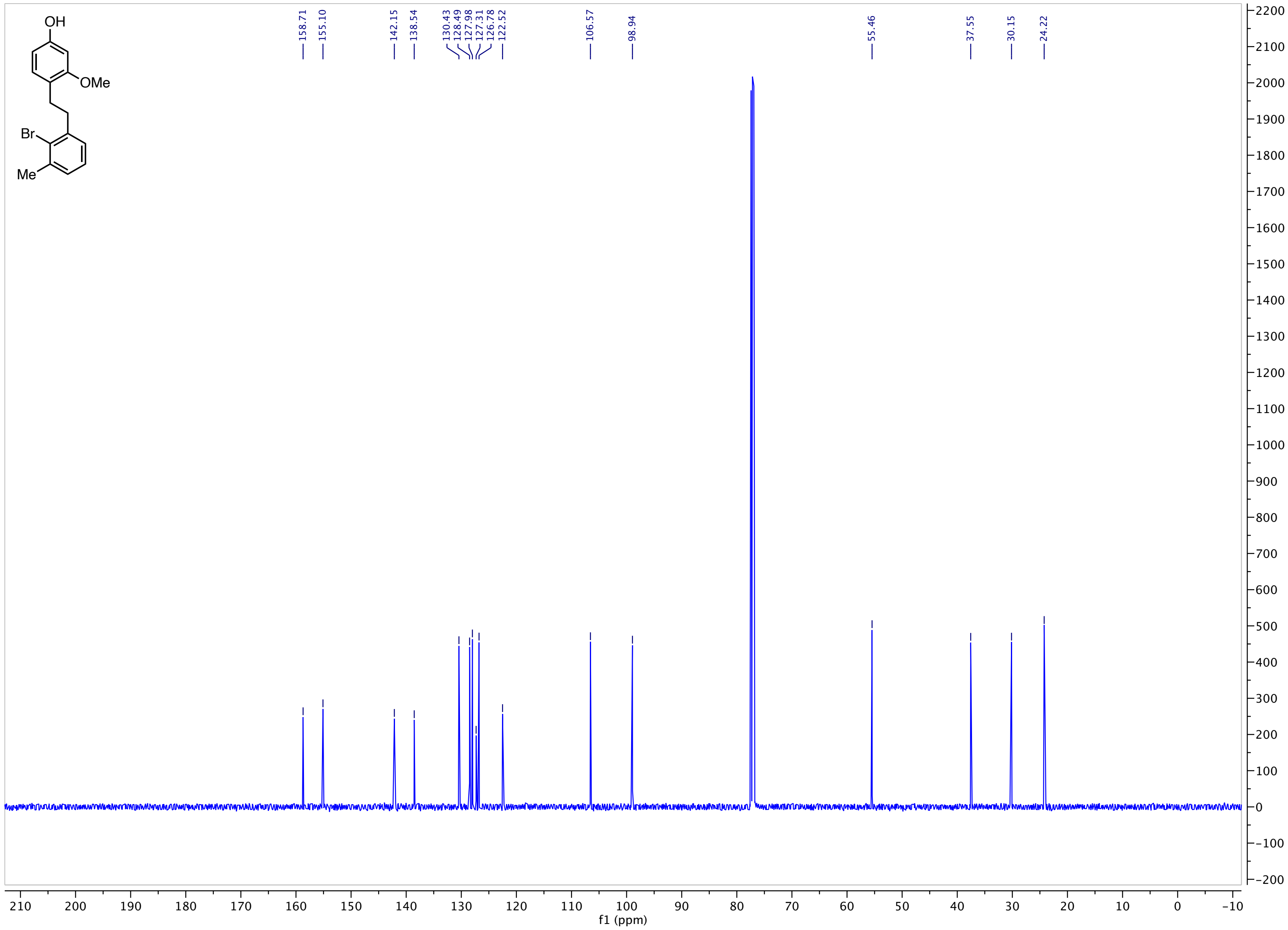

^{1}H NMR (MeCN-d_3): (2-bromo-5-fluorobenzyl)Triphenylphosphonium bromide **(S23)**

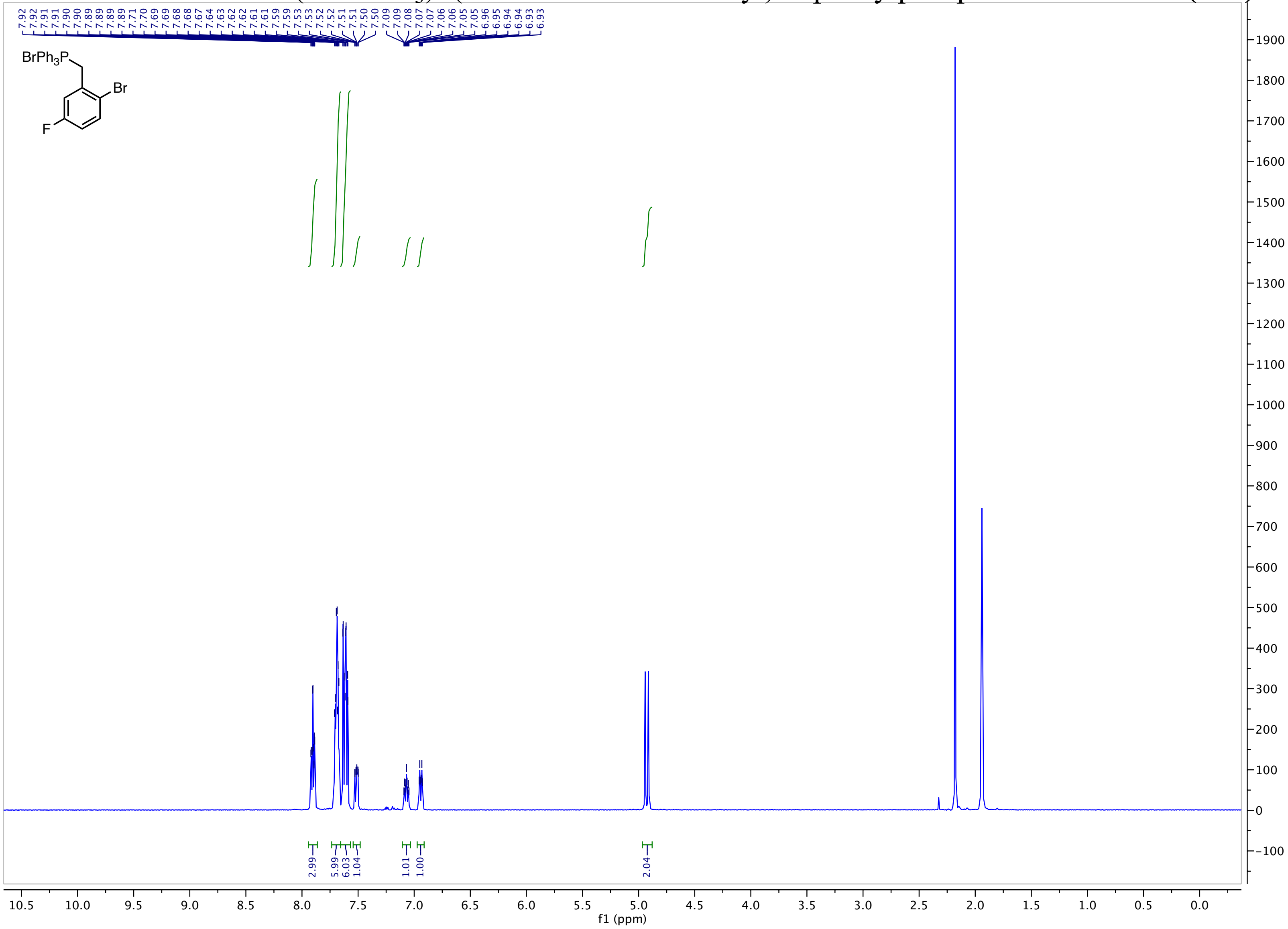

^{13}C NMR (DMSO-d6): (2-bromo-5-fluorobenzyl)Triphenylphosphonium bromide **(S23)**

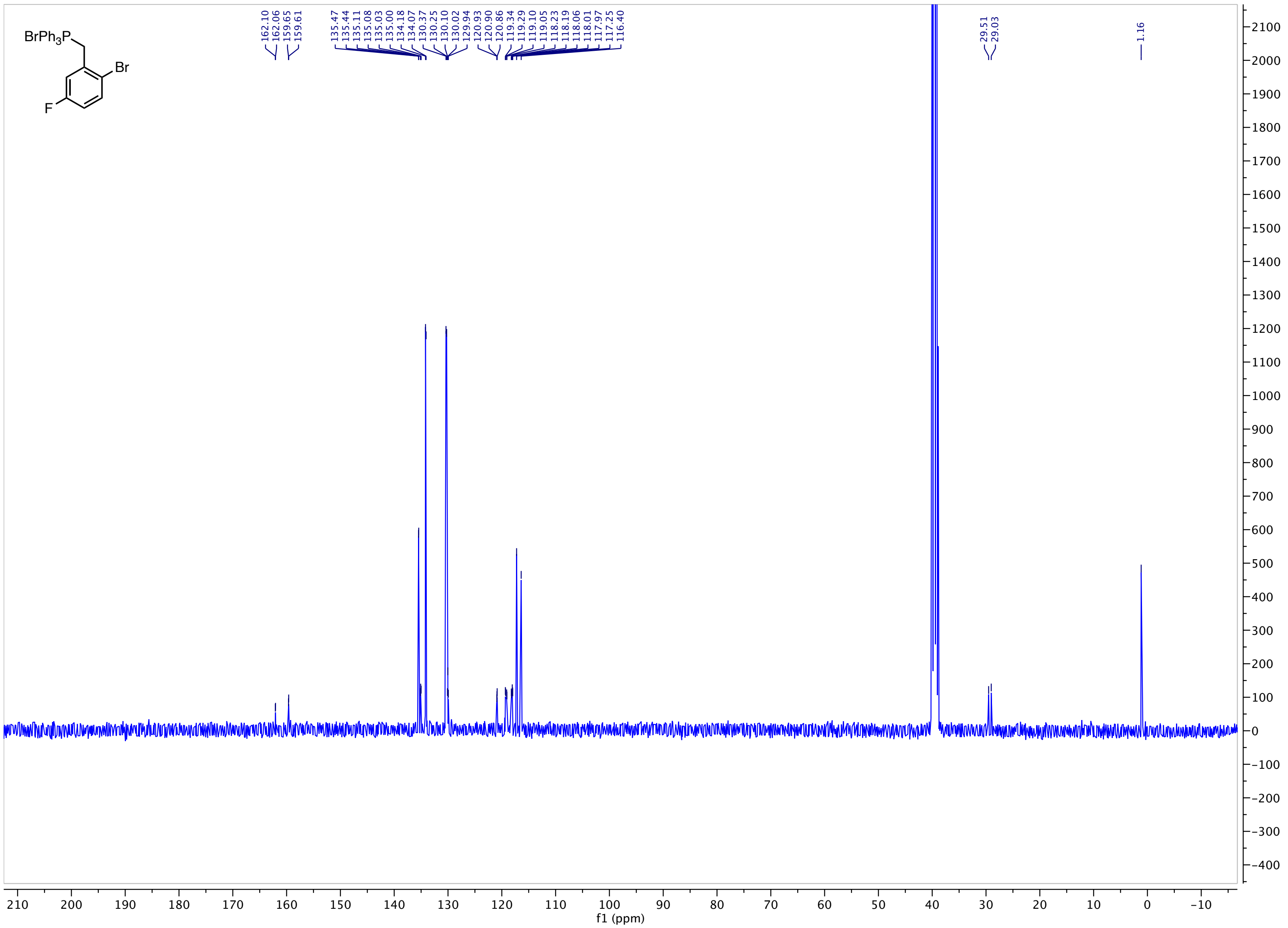

^{19}**F NMR** (MeCN-d$_3$): (2-bromo-5-fluorobenzyl)Triphenylphosphonium bromide **(S23)**

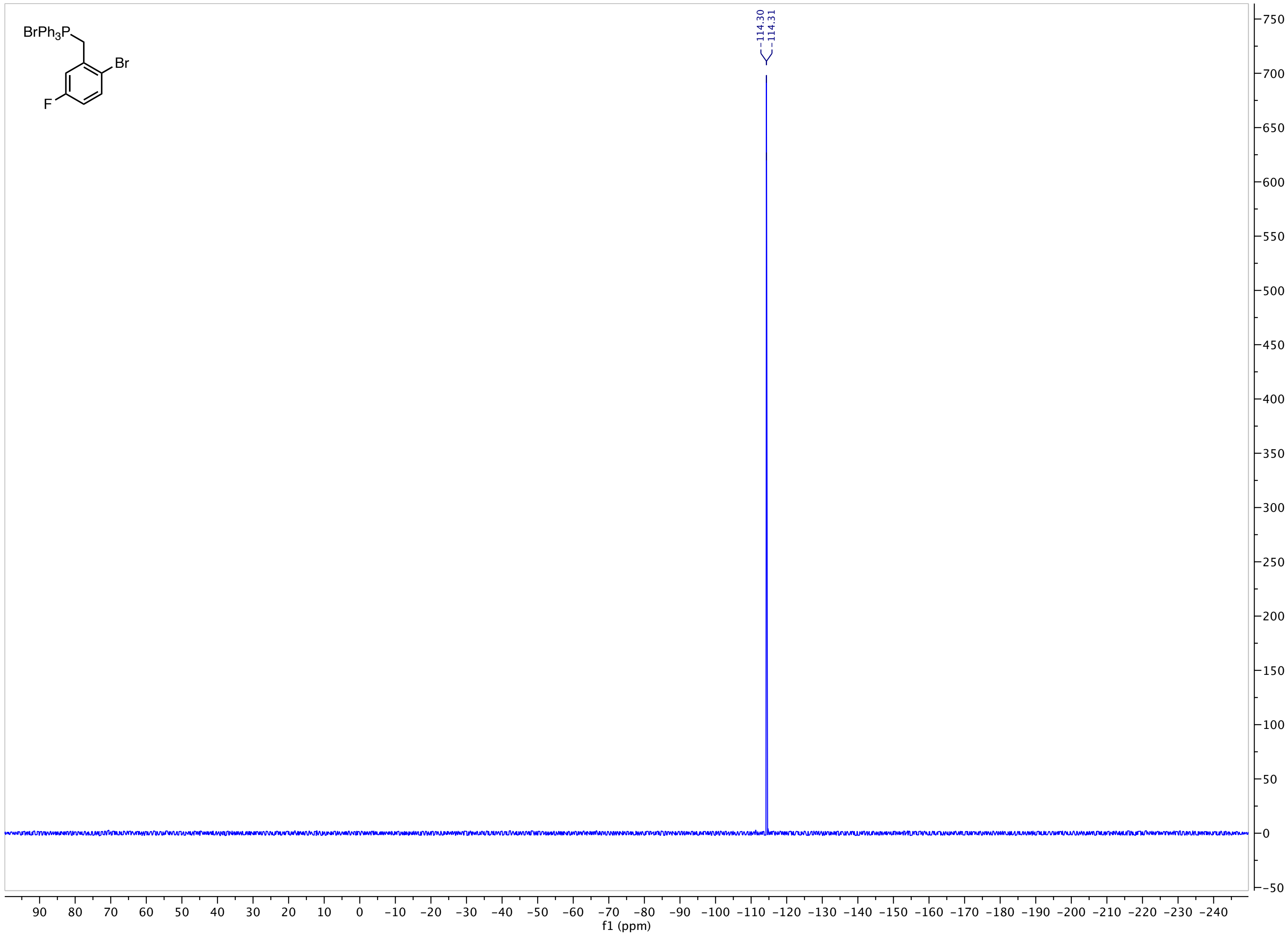

^{31}**P NMR** (MeCN-d_3): (2-bromo-5-fluorobenzyl)Triphenylphosphonium bromide **(S23)**

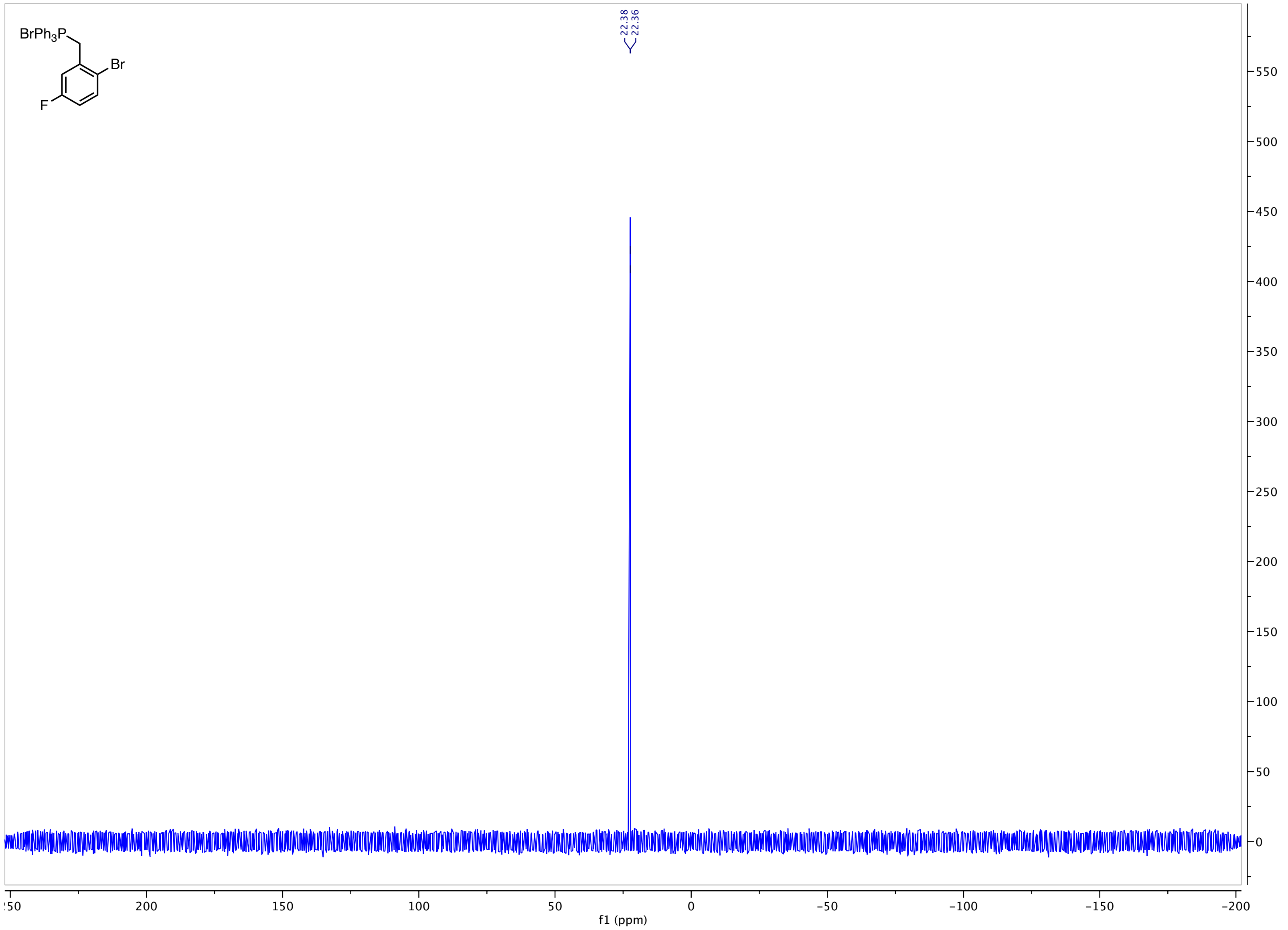

^{1}H NMR ($CDCl_3$): 4-(2-bromo-5-fluorophenethyl)-3-Methoxyphenol (1r)

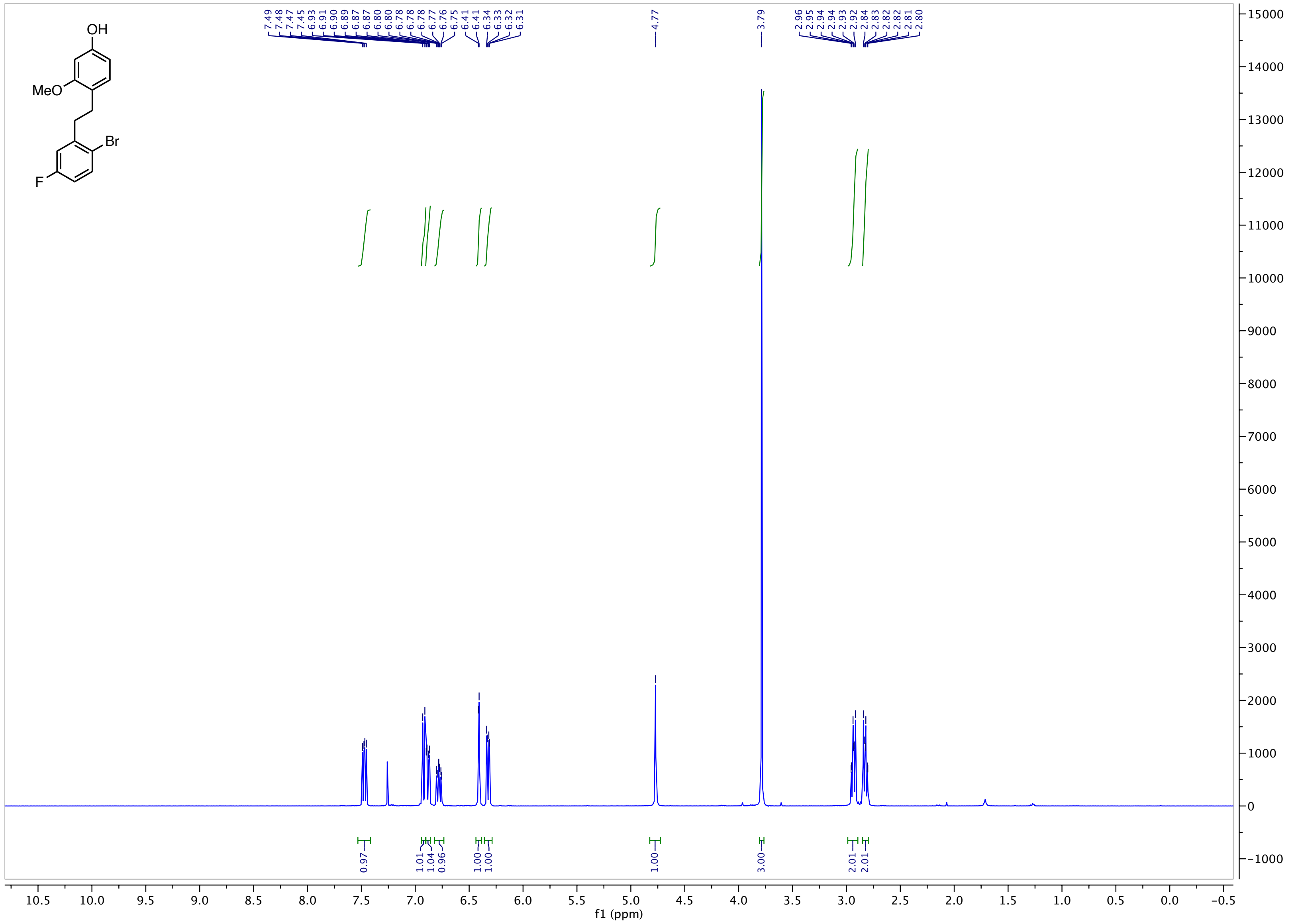

^{13}C NMR ($CDCl_3$): 4-(2-bromo-5-fluorophenethyl)-3-Methoxyphenol (1r)

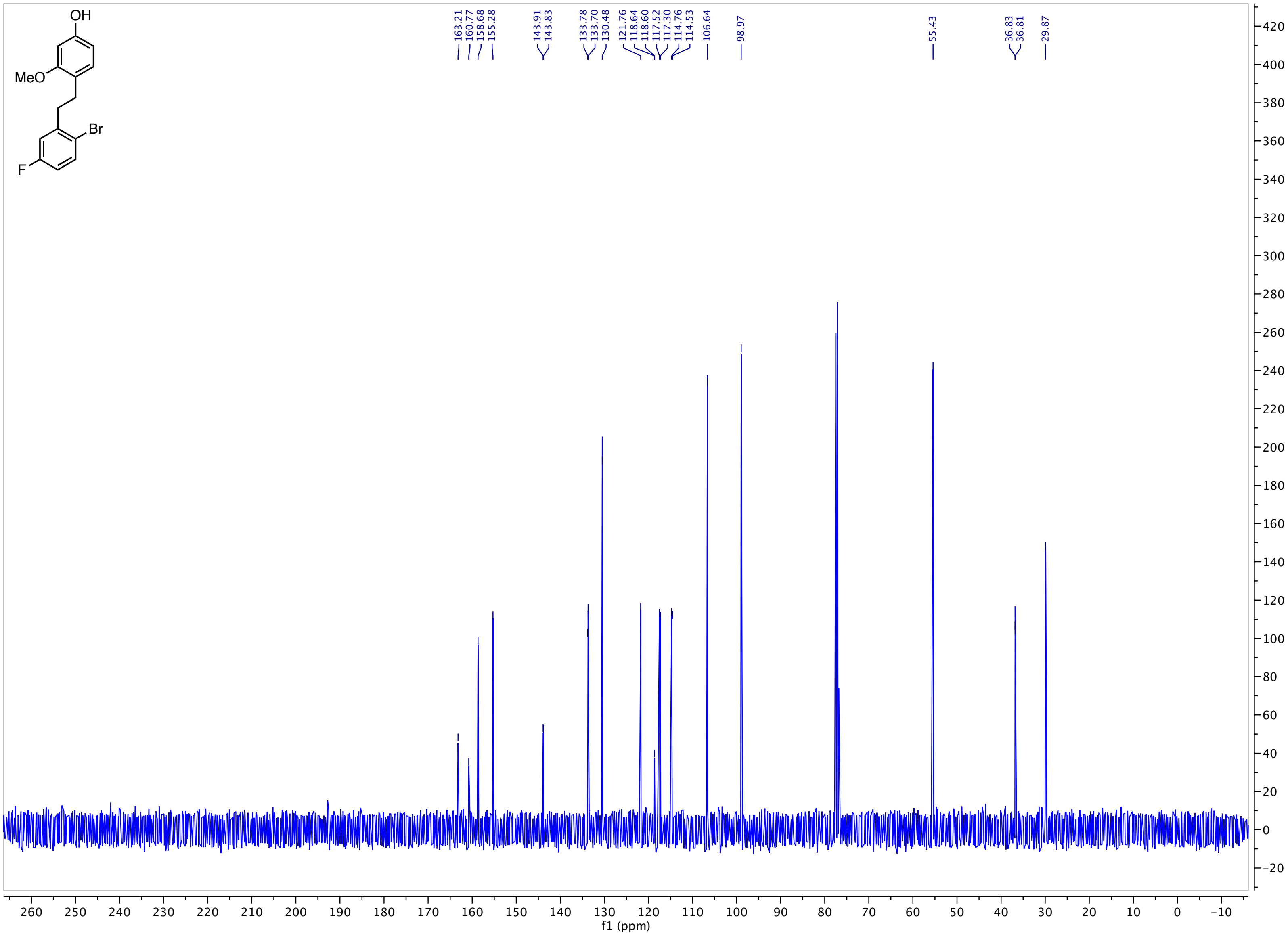

^{19}F NMR ($CDCl_3$): 4-(2-bromo-5-fluorophenethyl)-3-Methoxyphenol **(1r)**

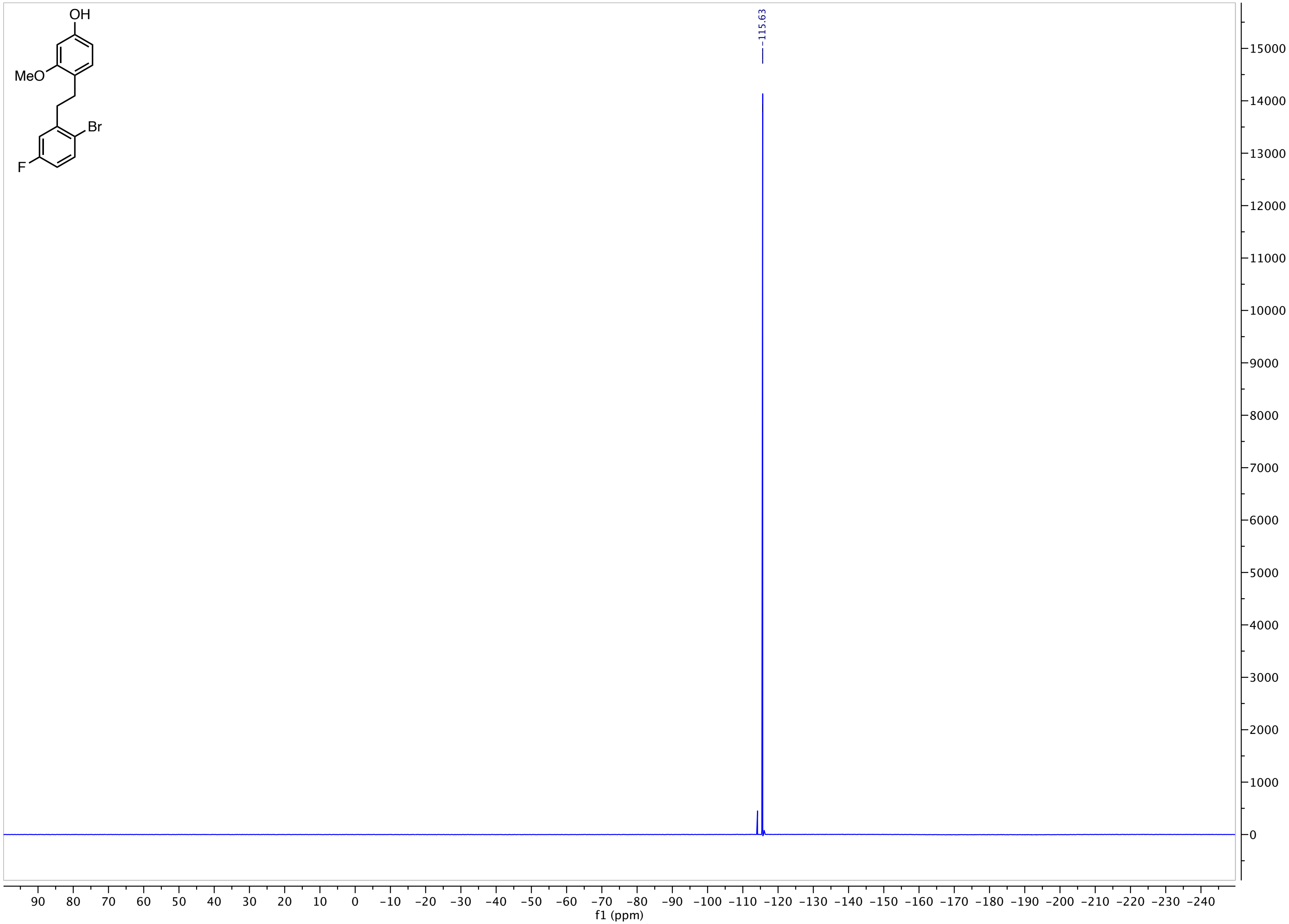

^{1}H NMR (MeCN-d_3): 2-Bromo-4-fluorobenzyl triphenylphosphonium bromide **(S24)**

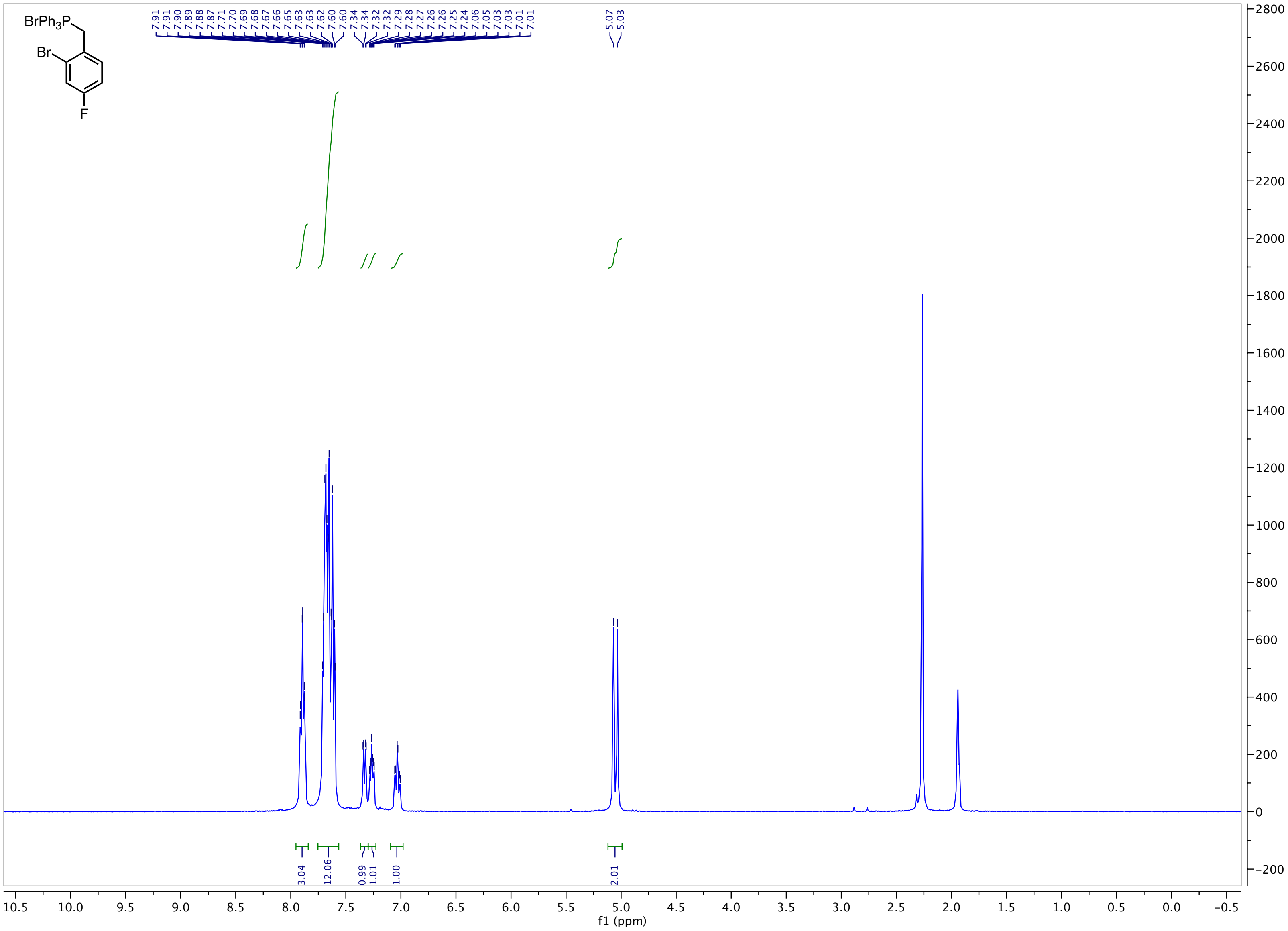

^{13}C NMR (MeCN-d_3): 2-Bromo-4-fluorobenzyl triphenylphosphonium bromide (S24)

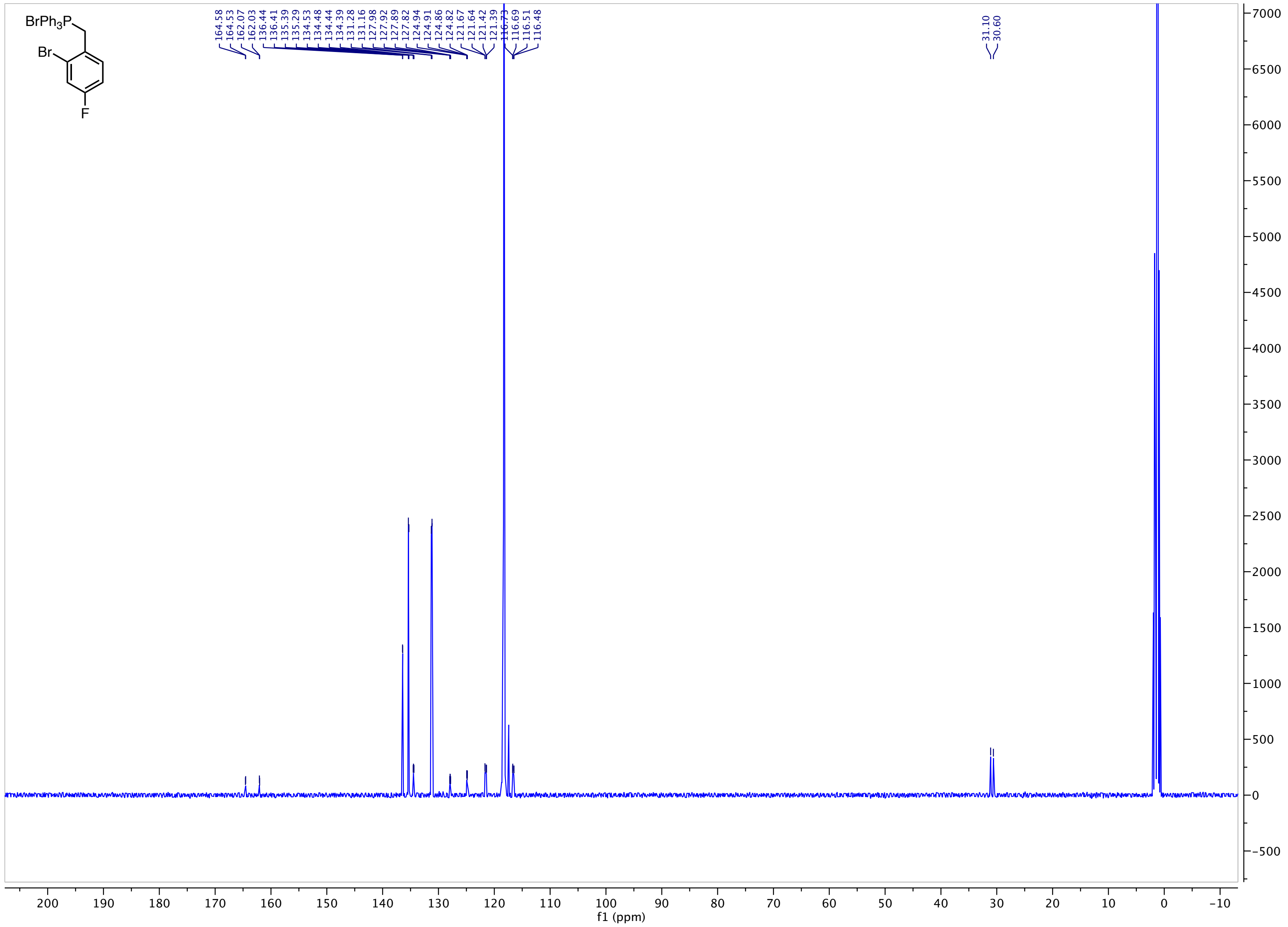

^{19}F NMR (MeCN-d_3): 2-Bromo-4-fluorobenzyl triphenylphosphonium bromide **(S24)**

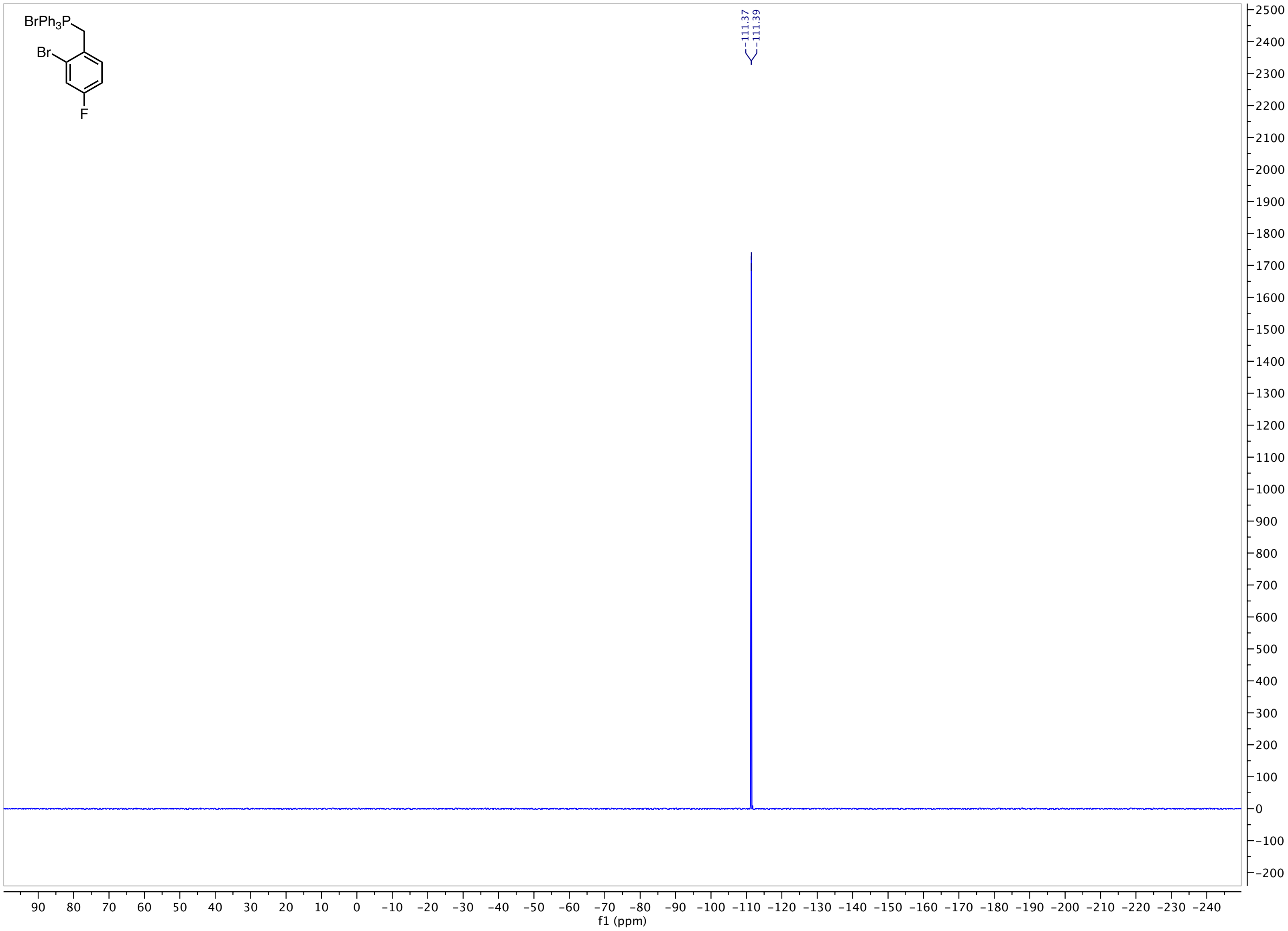

^{31}P NMR (MeCN-d_3): 2-Bromo-4-fluorobenzyl triphenylphosphonium bromide (S24)

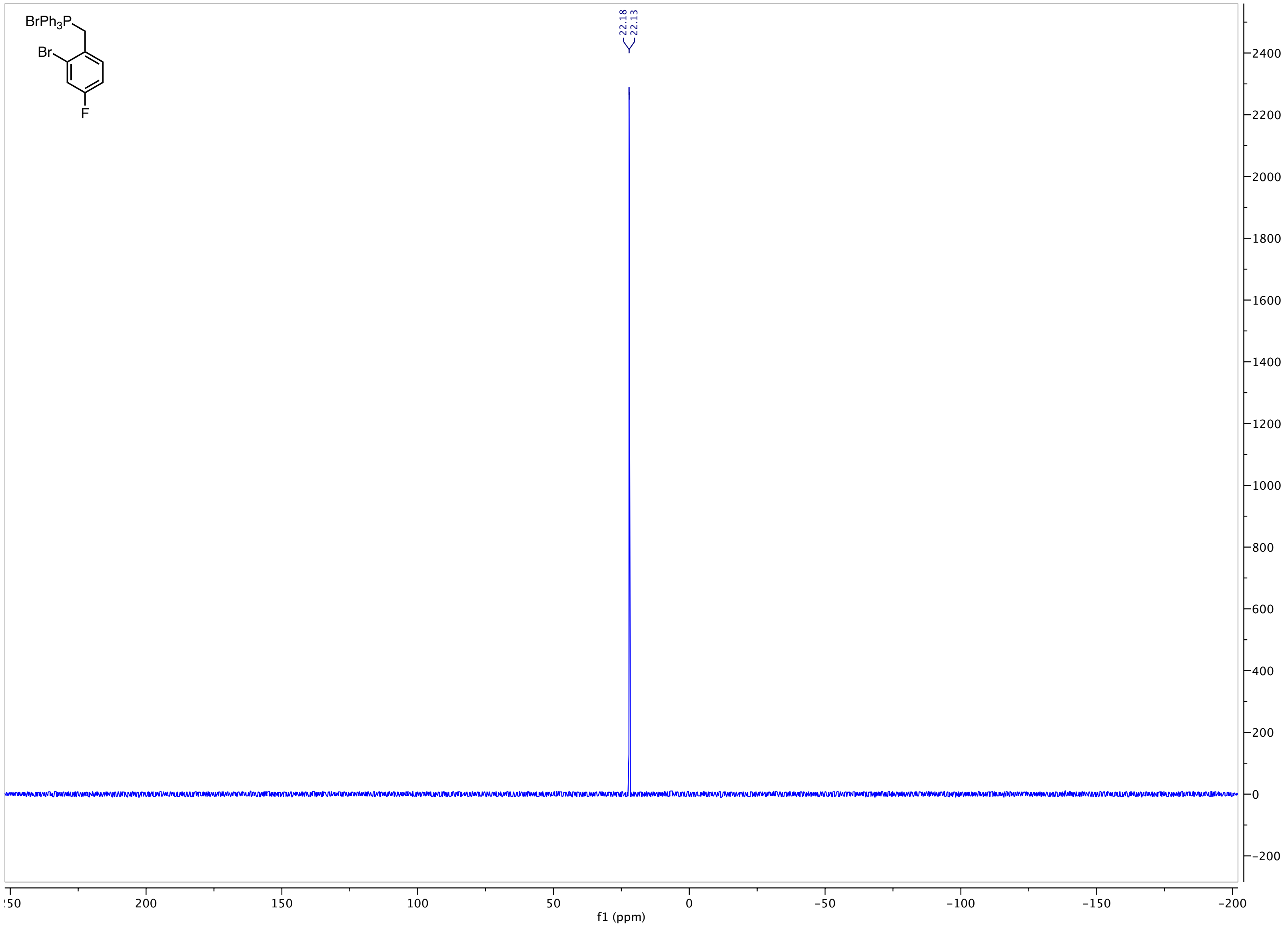

^{1}H NMR ($CDCl_3$): 4-(2-bromo-4-fluorophenethyl)-3-Methoxyphenol (1s)

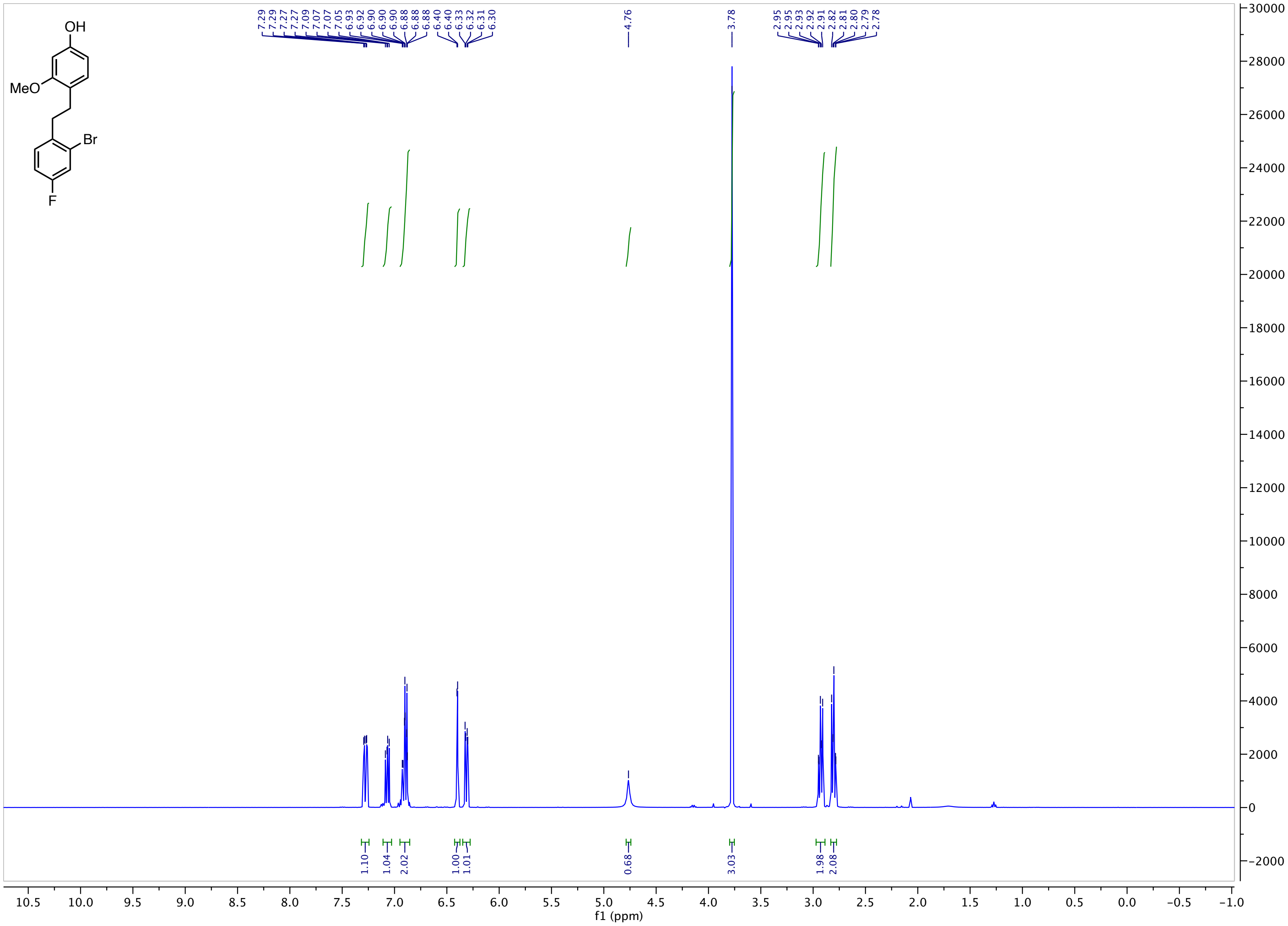

^{13}C NMR ($CDCl_3$): 4-(2-bromo-4-fluorophenethyl)-3-Methoxyphenol **(1s)**

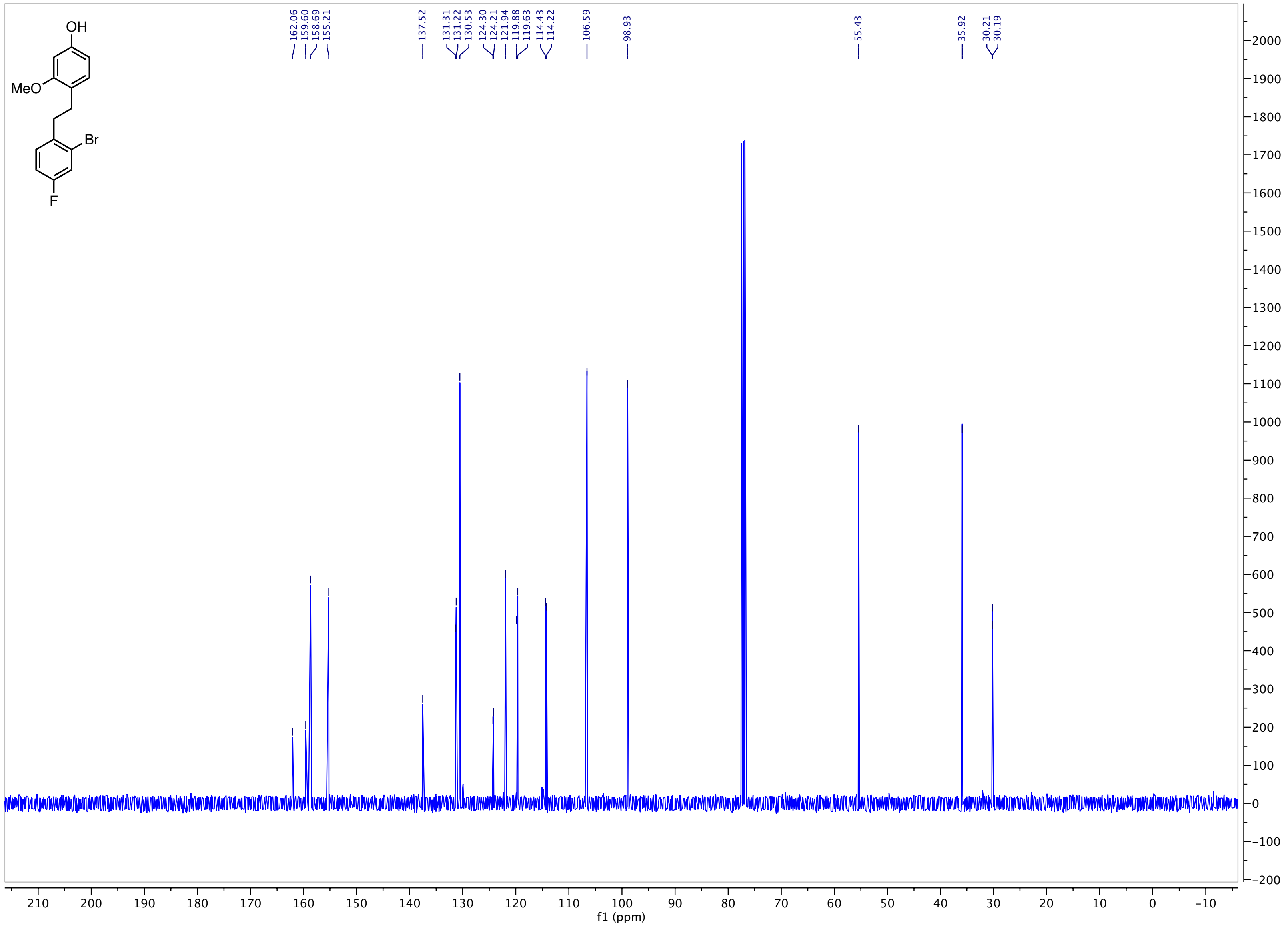

^{19}**F NMR** (CDCl$_3$): 4-(2-bromo-4-fluorophenethyl)-3-Methoxyphenol **(1s)**

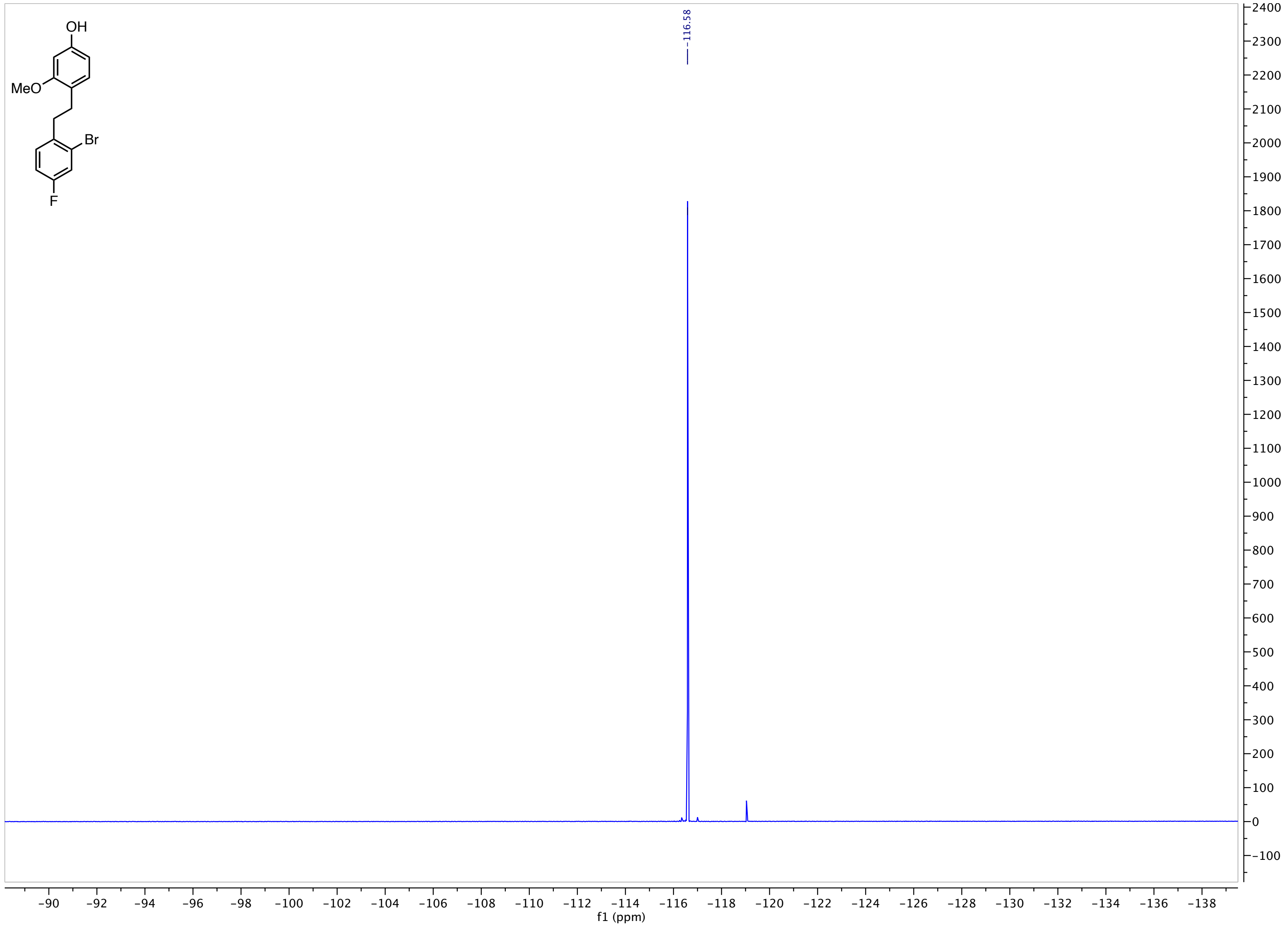

^{1}H NMR (MeOD): Indolin-5-ol (S25)

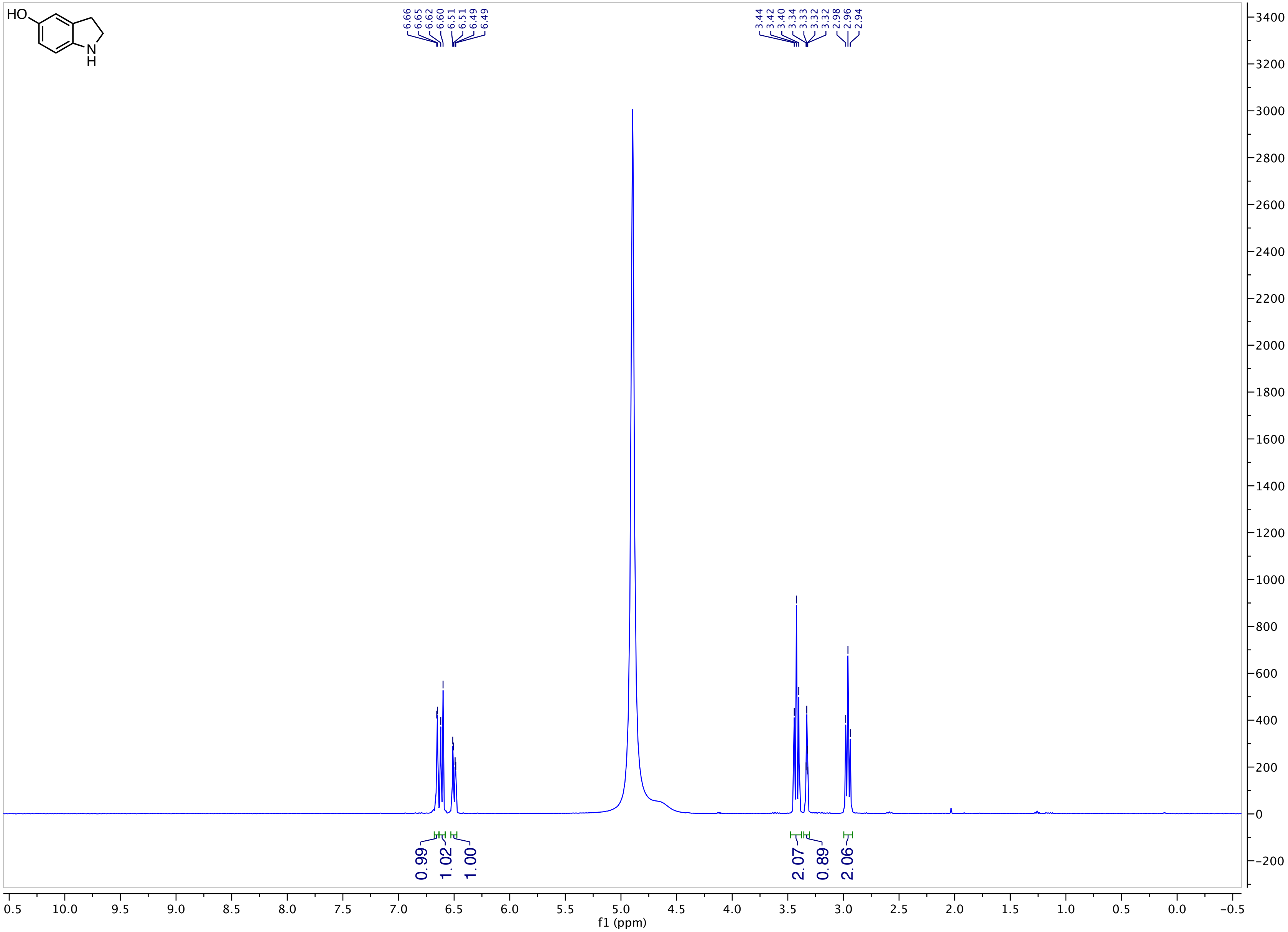

^{13}C NMR (MeOD): Indolin-5-ol **(S25)**

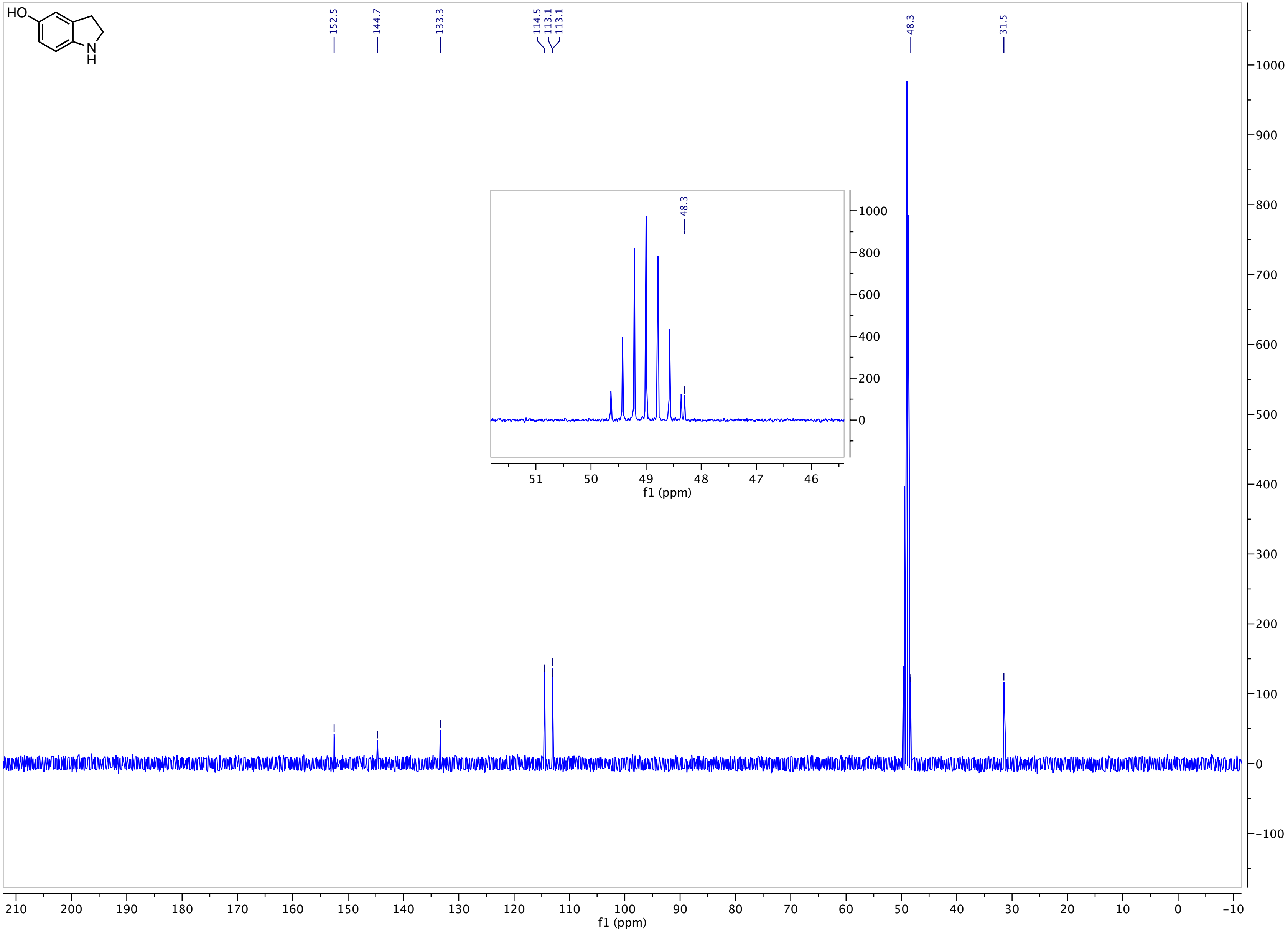

^{1}H NMR ($CDCl_3$): 2-(2-bromophenyl)Acetaldehyde (S26)

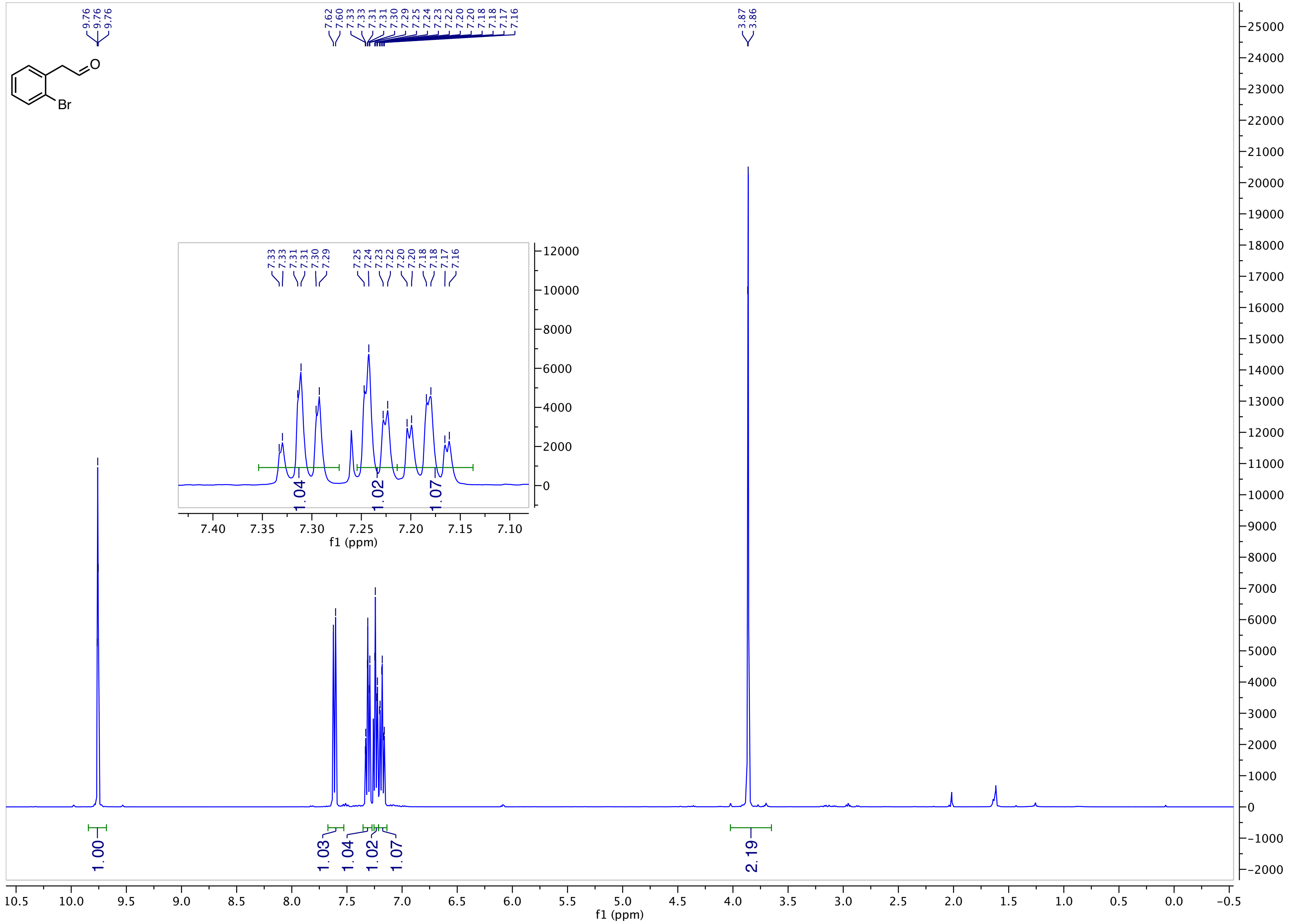

^{13}C NMR (CDCl$_3$): 2-(2-bromophenyl)Acetaldehyde **(S26)**

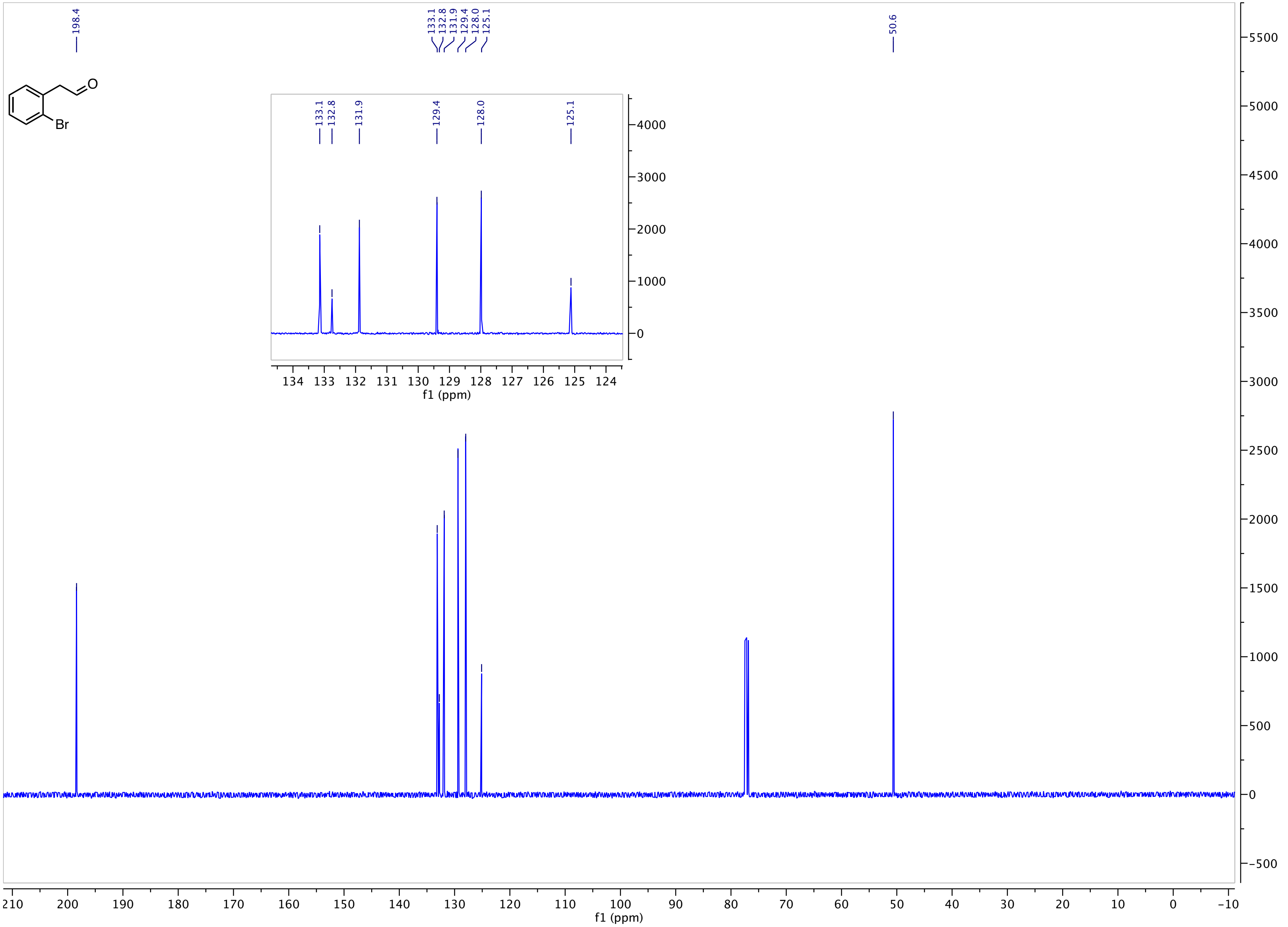

^{1}H NMR (Acetone-d$_6$): 1-(2-bromophenethyl)Indolin-5-ol (3a)

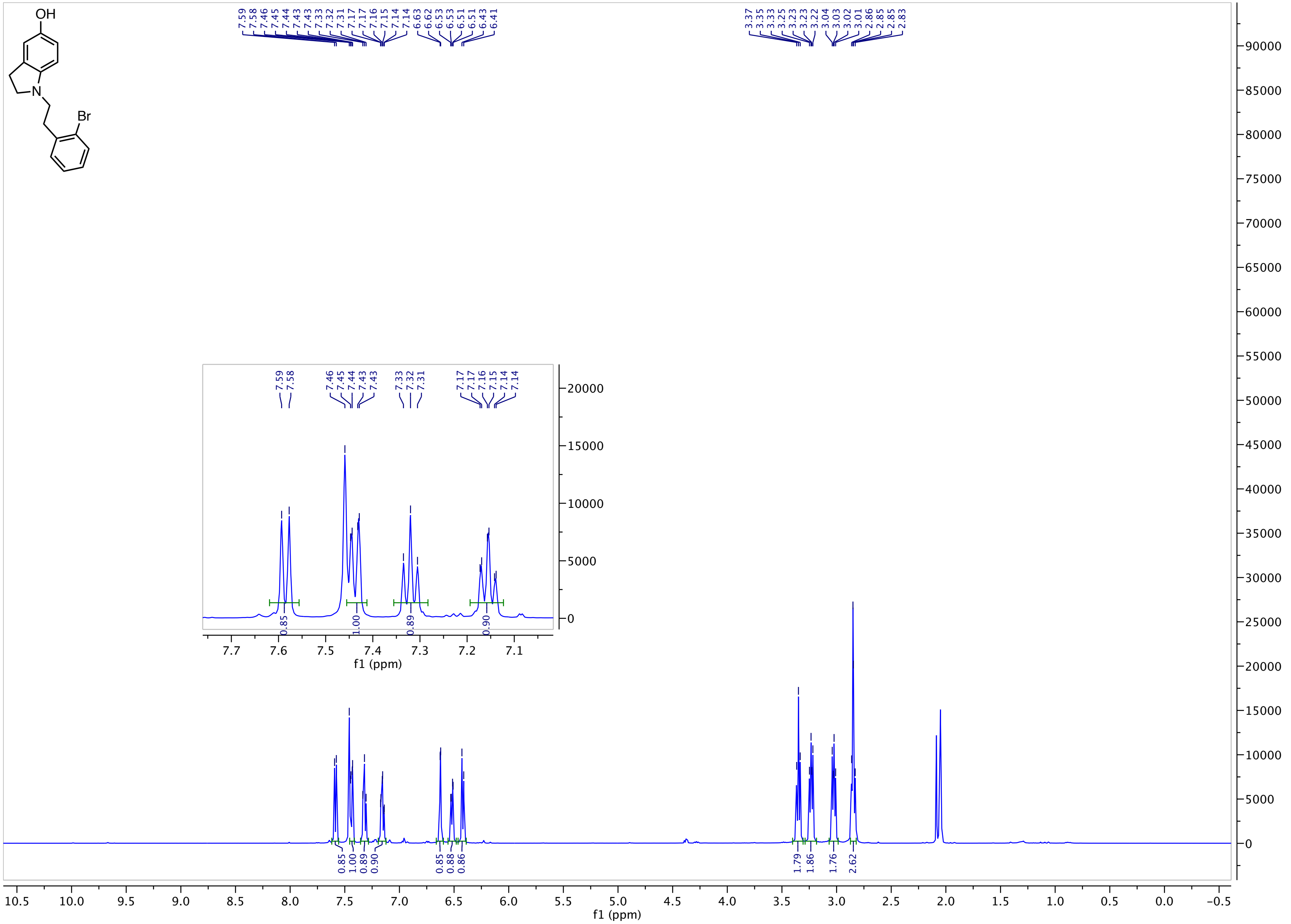

^{13}C NMR (Acetone-d_6): 1-(2-bromophenethyl)Indolin-5-ol **(3a)**

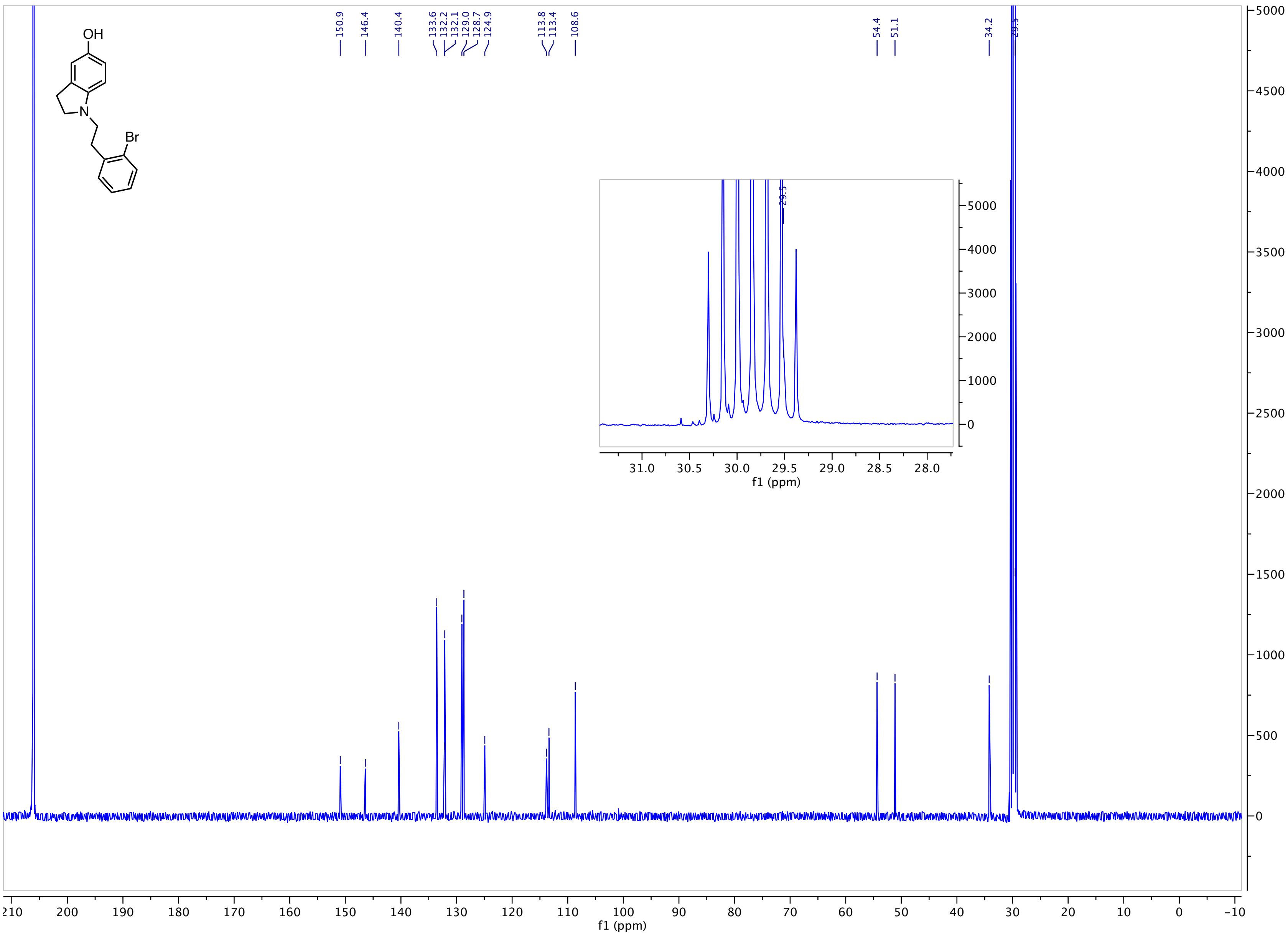

^{1}H NMR ($CDCl_3$): 2-(2-chlorophenyl)Acetaldehyde **(S27)**

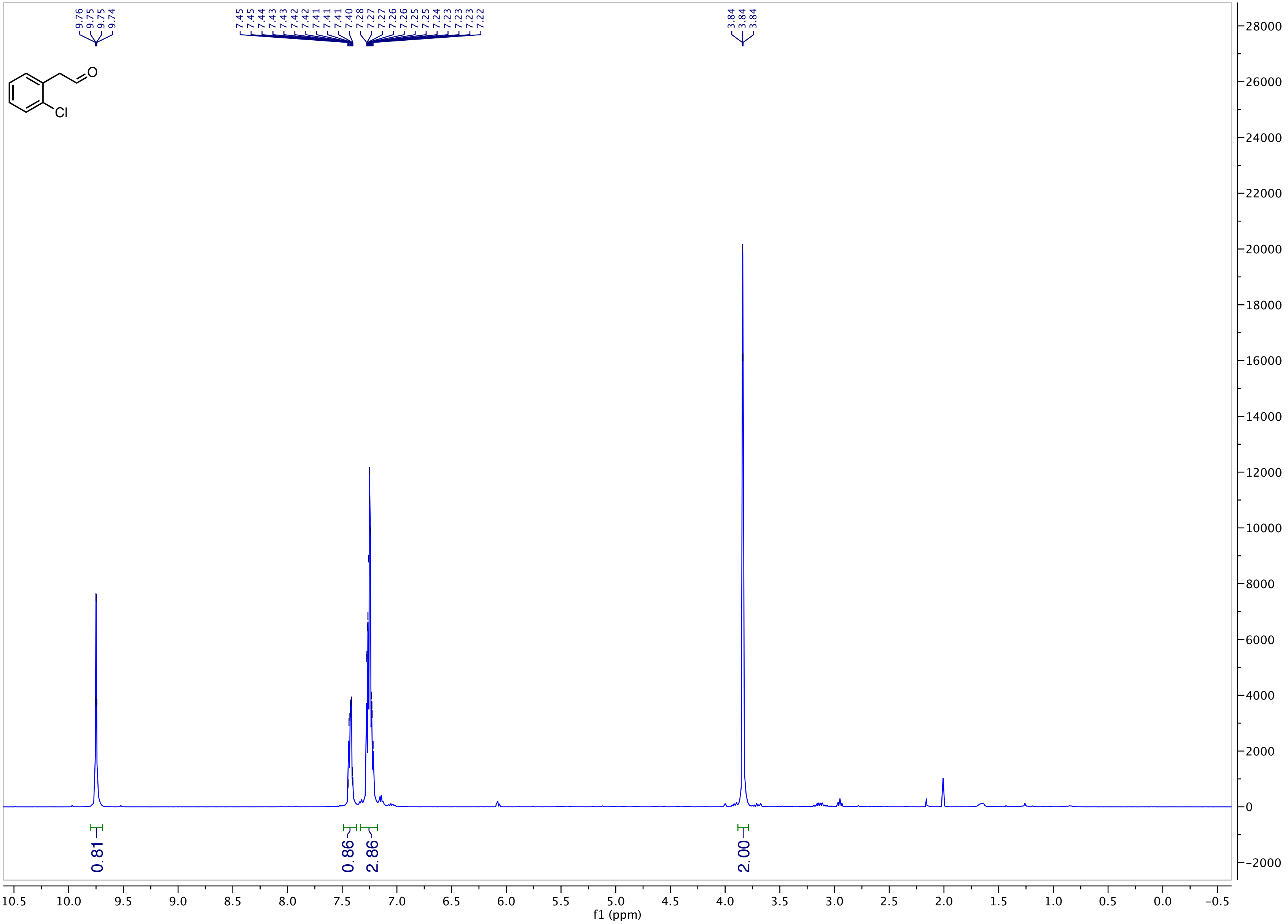

^{13}C NMR ($CDCl_3$): 2-(2-chlorophenyl)Acetaldehyde (S27)

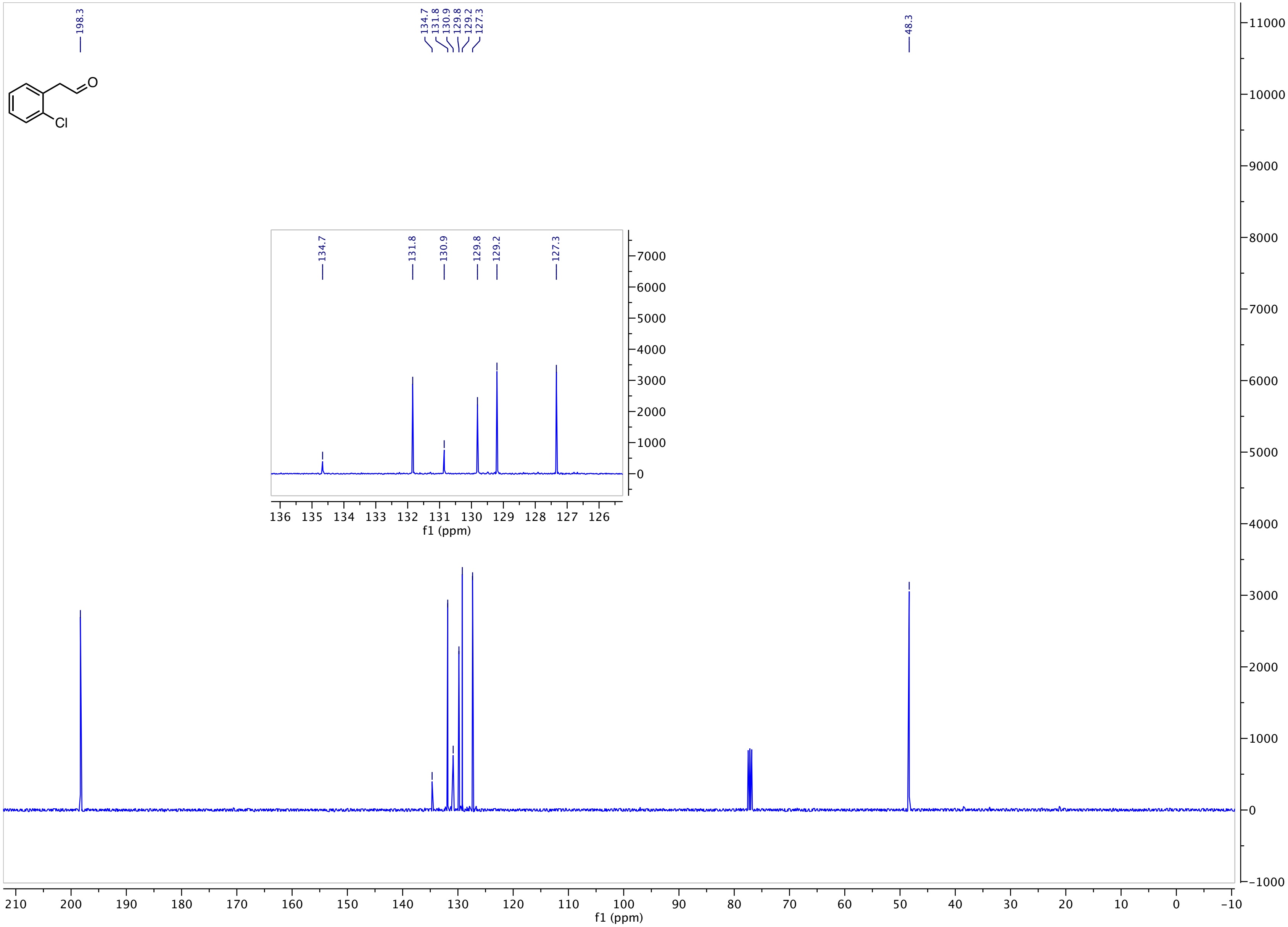

^{1}**H NMR** (Acetone-d_6): 1-(2-chlorophenethyl)Indolin-5-ol **(3a')**

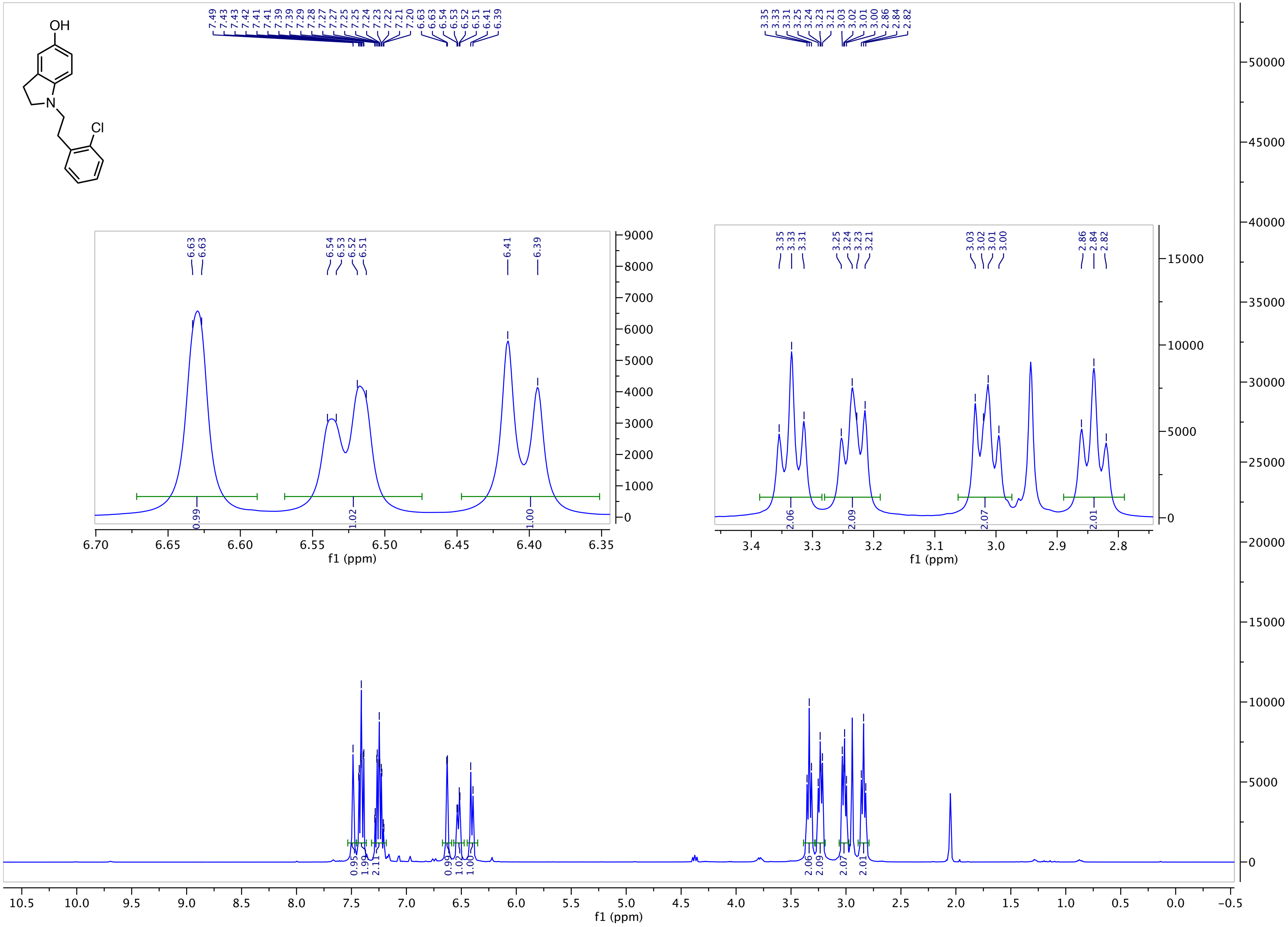

^{13}C NMR (Acetone-d_6): 1-(2-chlorophenethyl)Indolin-5-ol **(3a')**

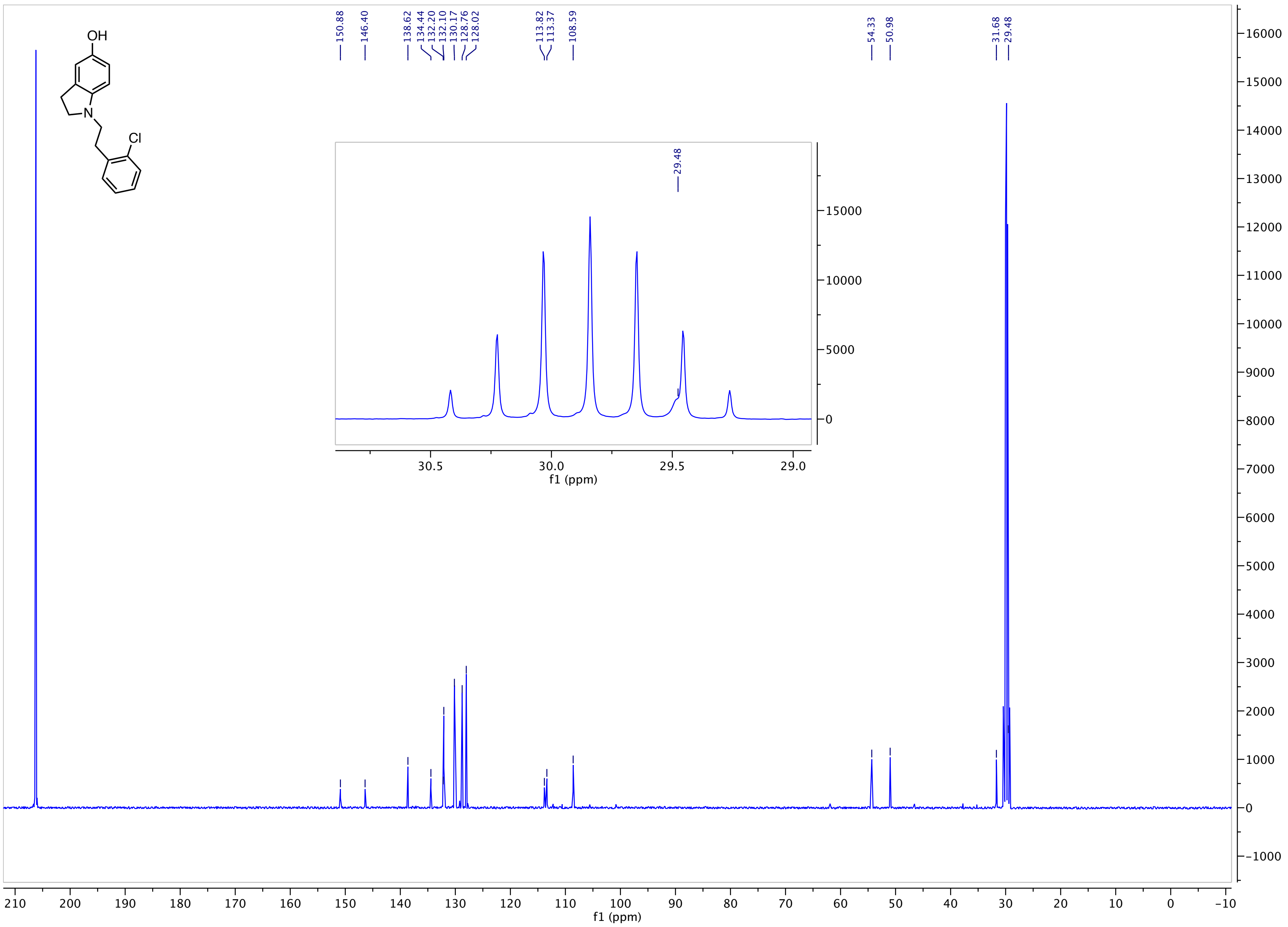

^{1}H NMR (Acetone-d_6): 1-(2-bromophenethyl)-1,2,3,4-Tetrahydroquinolin-6-ol **(3b)**

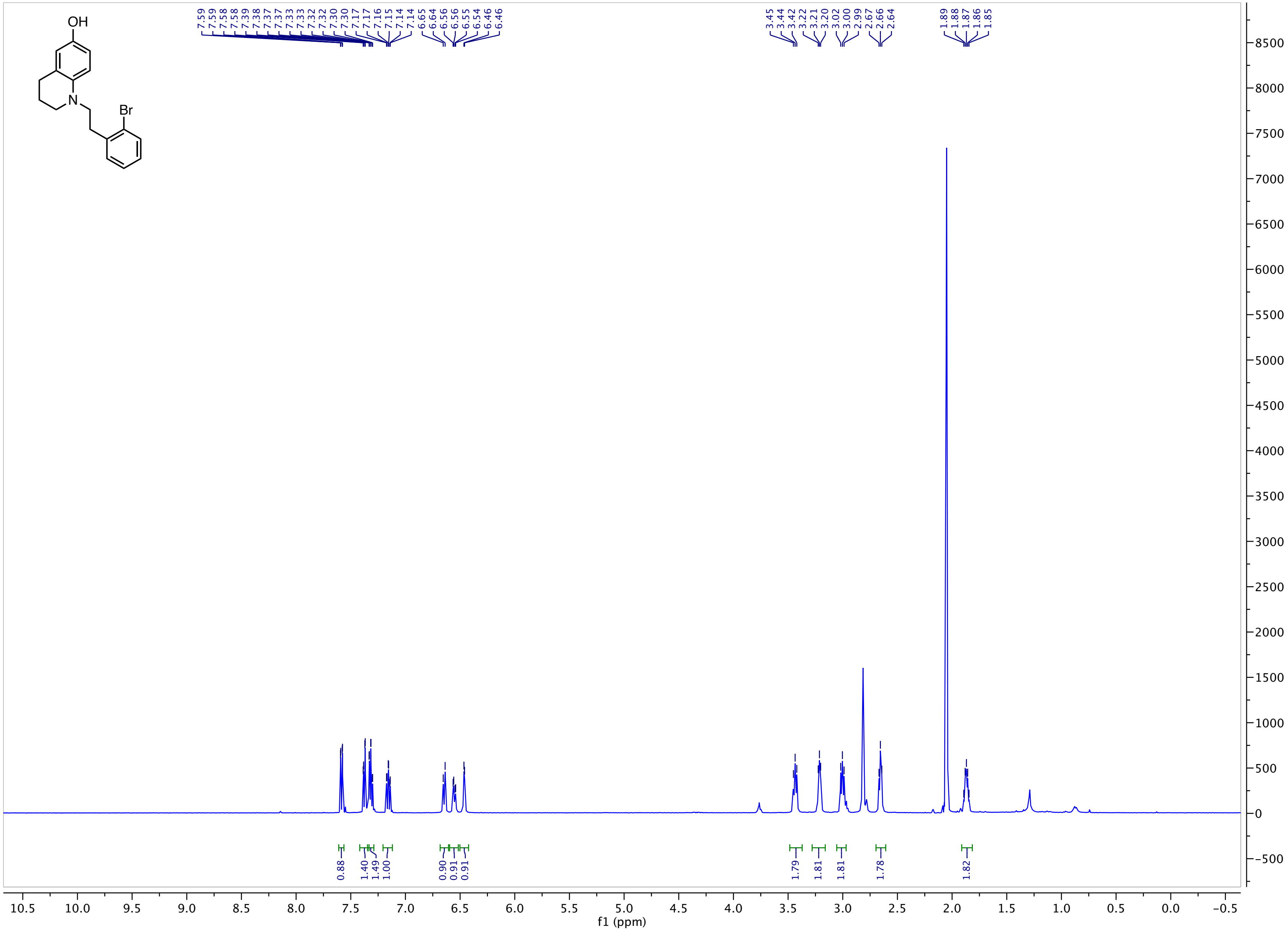

^{13}C NMR (Acetone-d_6): 1-(2-bromophenethyl)-1,2,3,4-Tetrahydroquinolin-6-ol **(3b)**

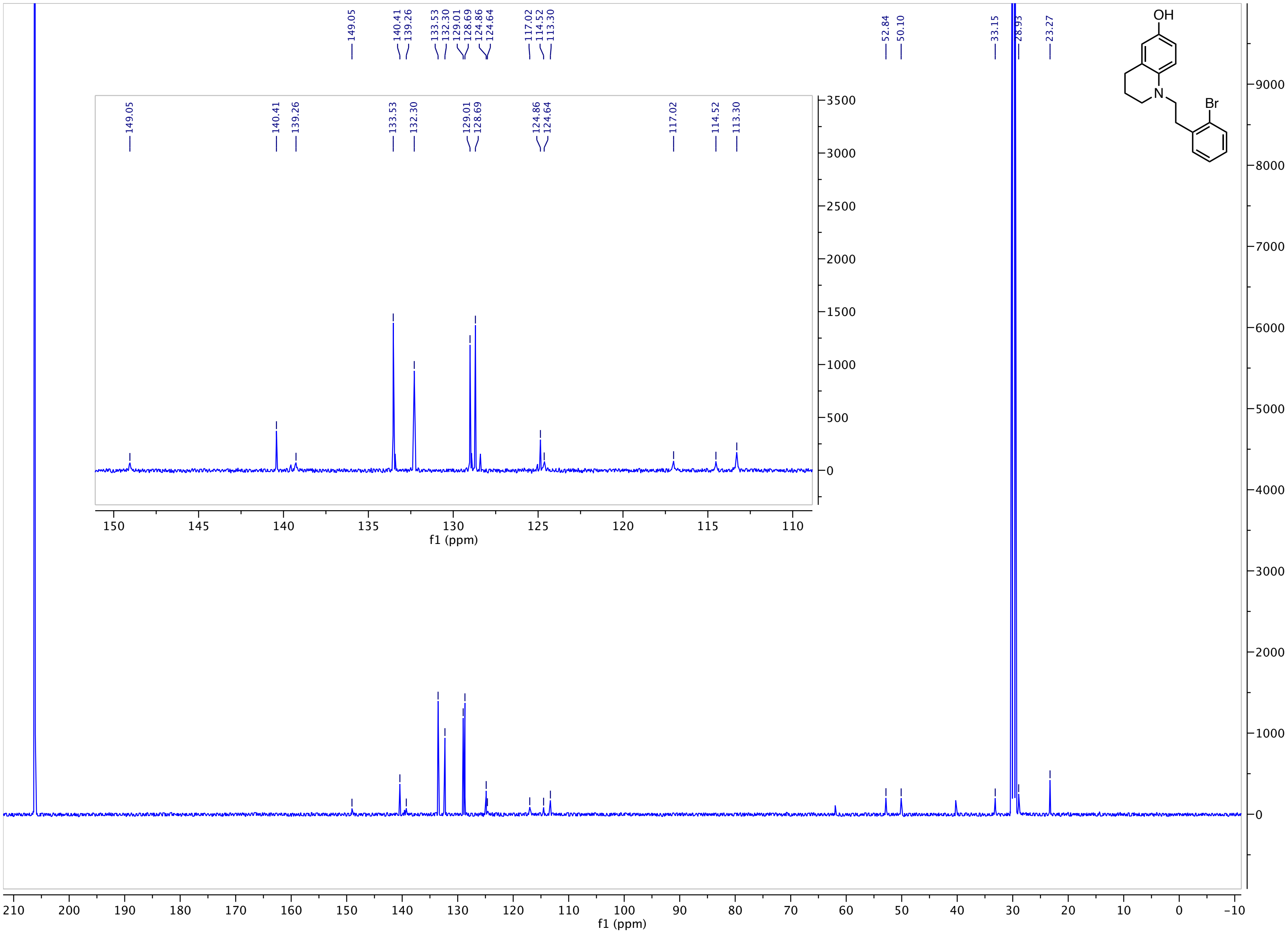

^{1}H NMR (CDCl$_3$): 2-(2-bromo-5-methoxyphenyl)acetaldehyde **(S28)**

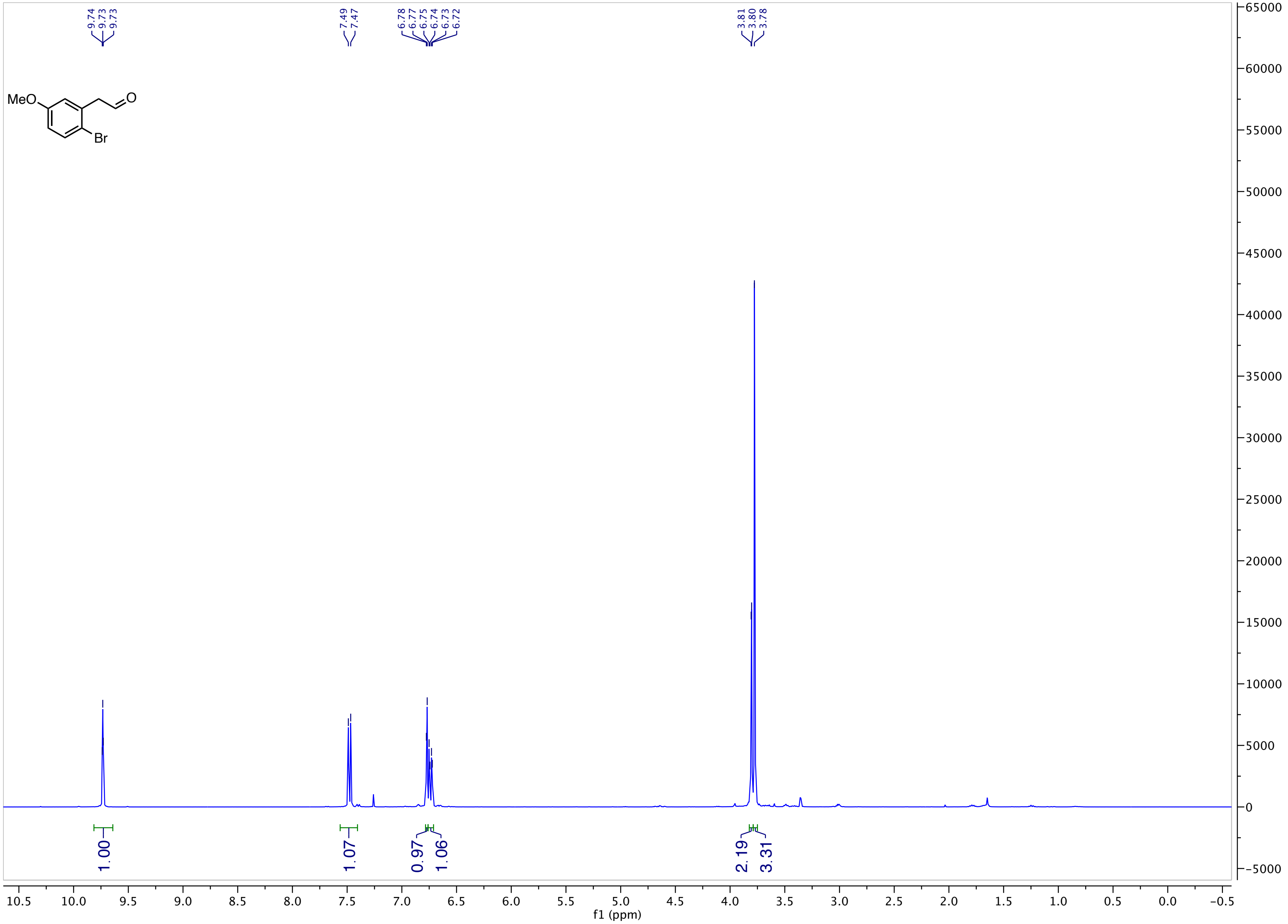

^{13}C NMR ($CDCl_3$): 2-(2-bromo-5-methoxyphenyl)Acetaldehyde (S28)

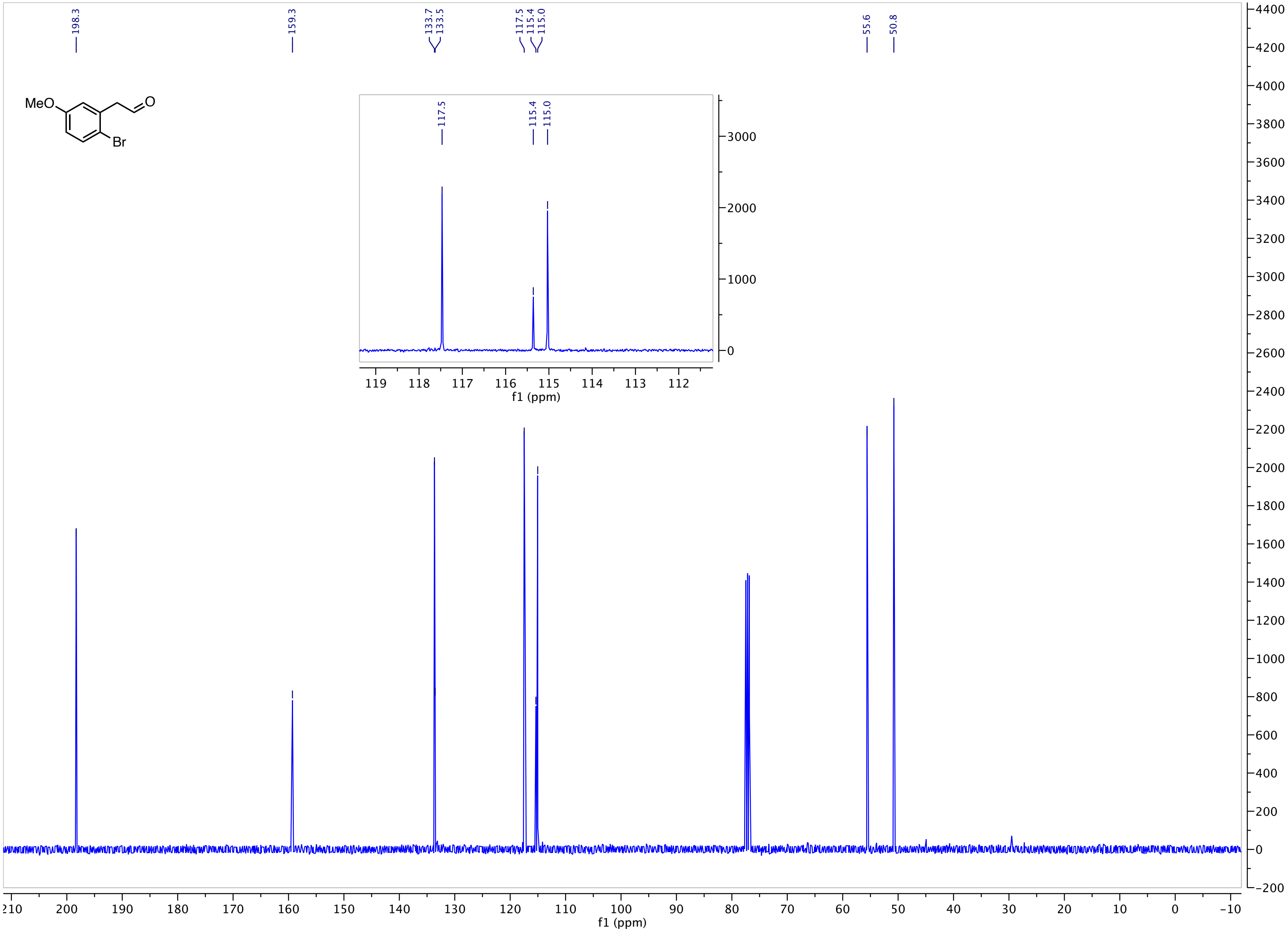

^{1}H NMR (Acetone-d_6): 1-(2-bromo-5-methoxyphenethyl)Indolin-5-ol **(3c)**

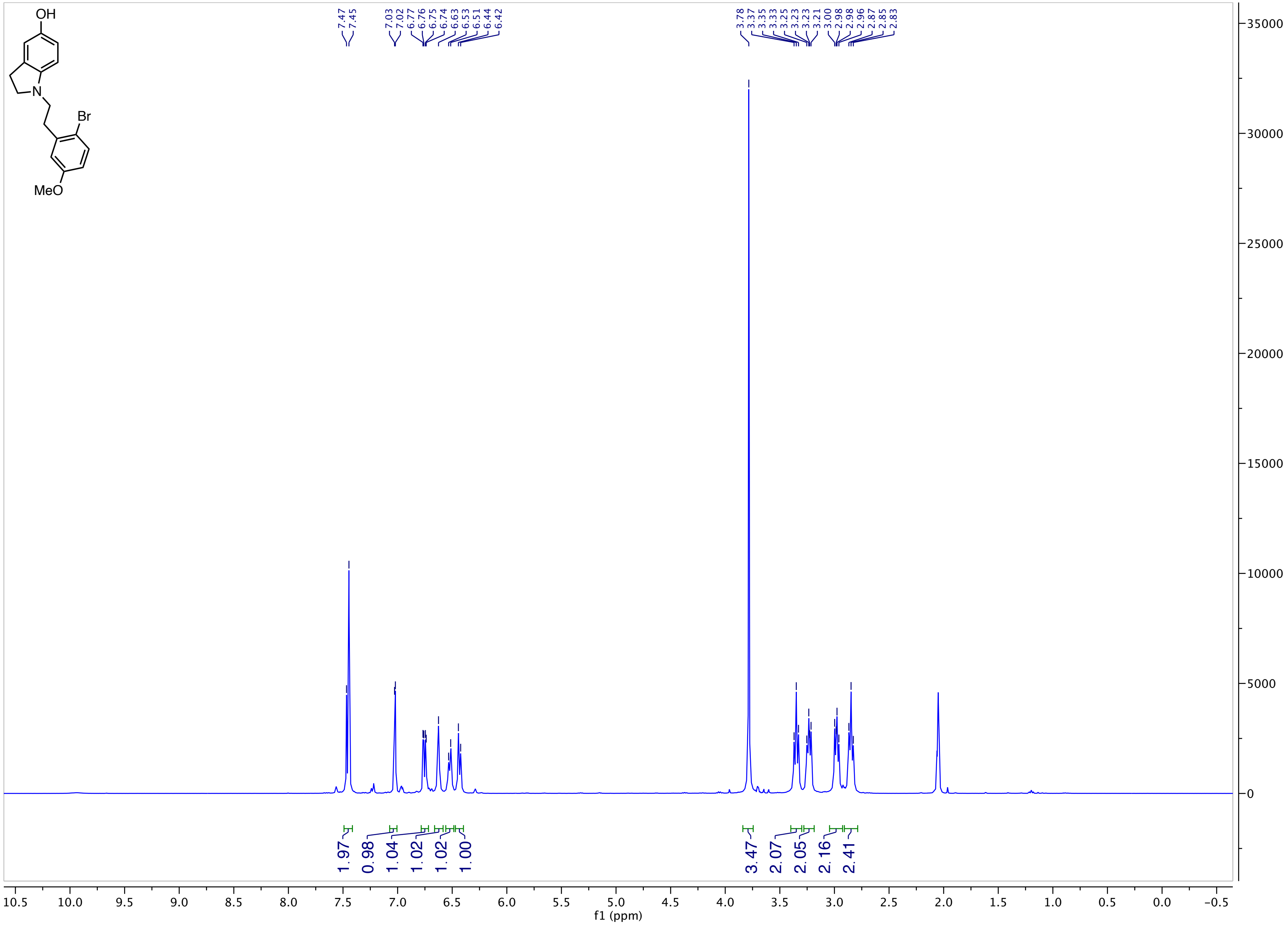

^{13}C NMR (Acetone-d_6): 1-(2-bromo-5-methoxyphenethyl)Indolin-5-ol **(3c)**

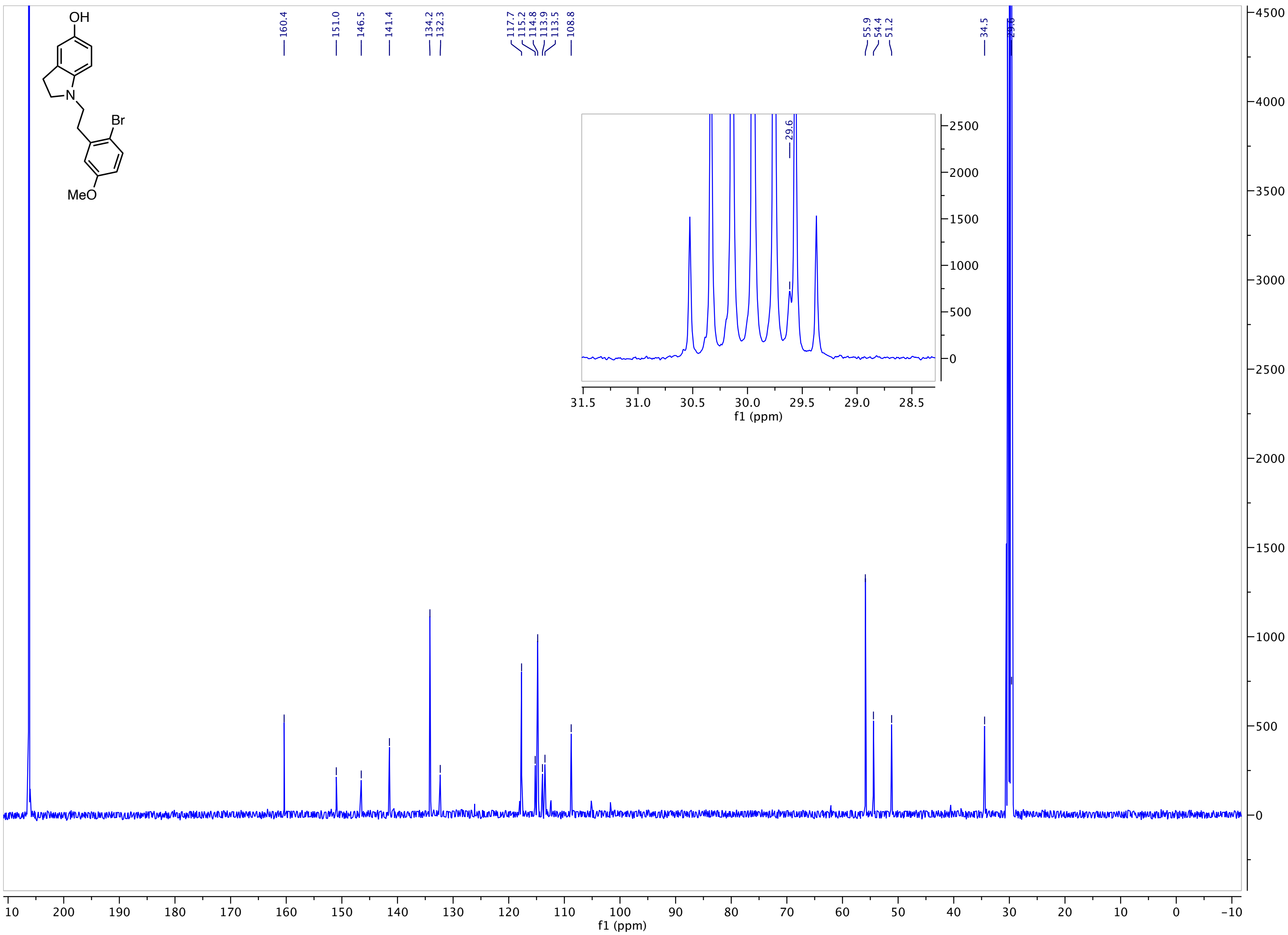

^{1}H NMR ($CDCl_3$): 2-(2-bromo-5-chlorophenyl)Acetaldehyde **(S29)**

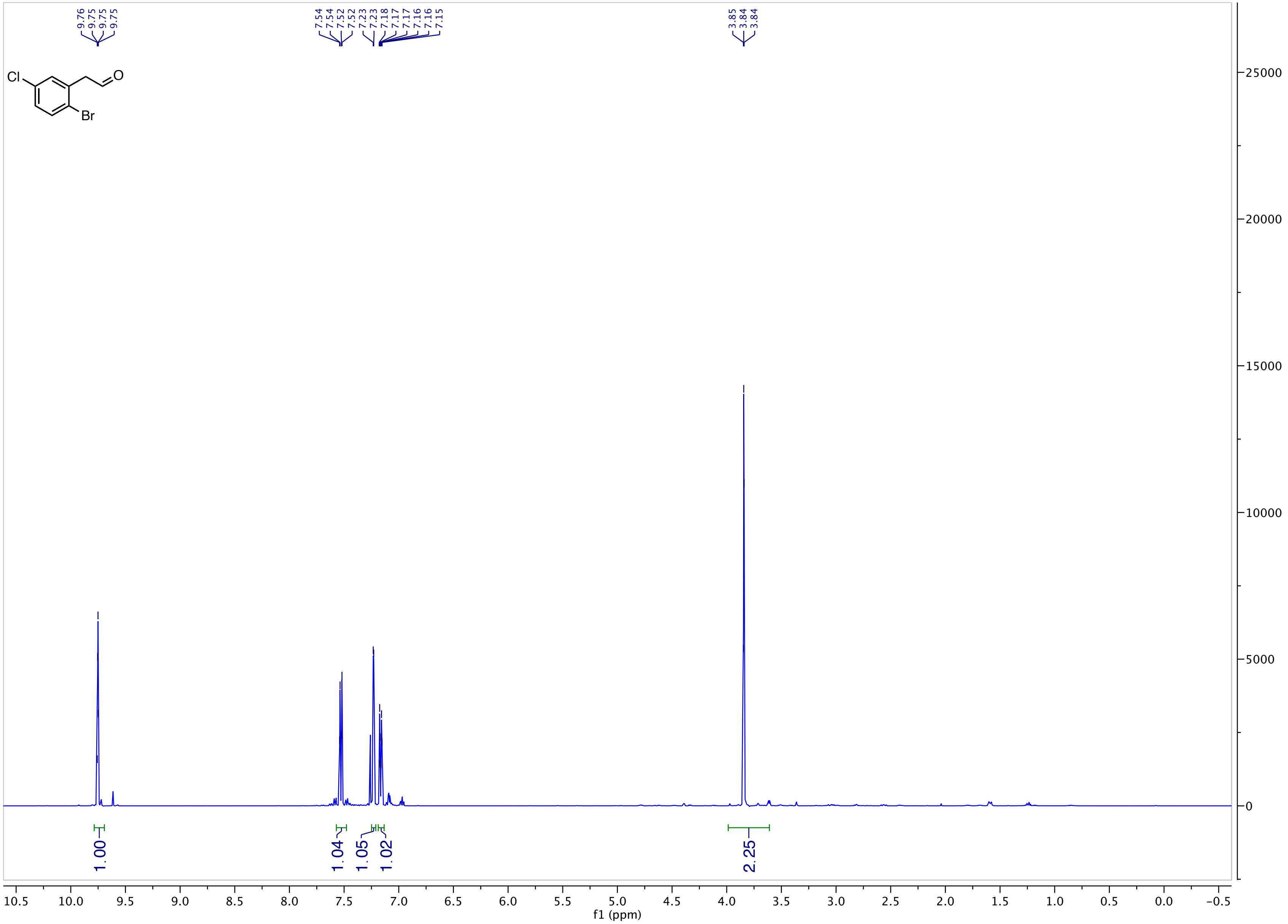

^{13}C NMR ($CDCl_3$): 2-(2-bromo-5-chlorophenyl)Acetaldehyde **(S29)**

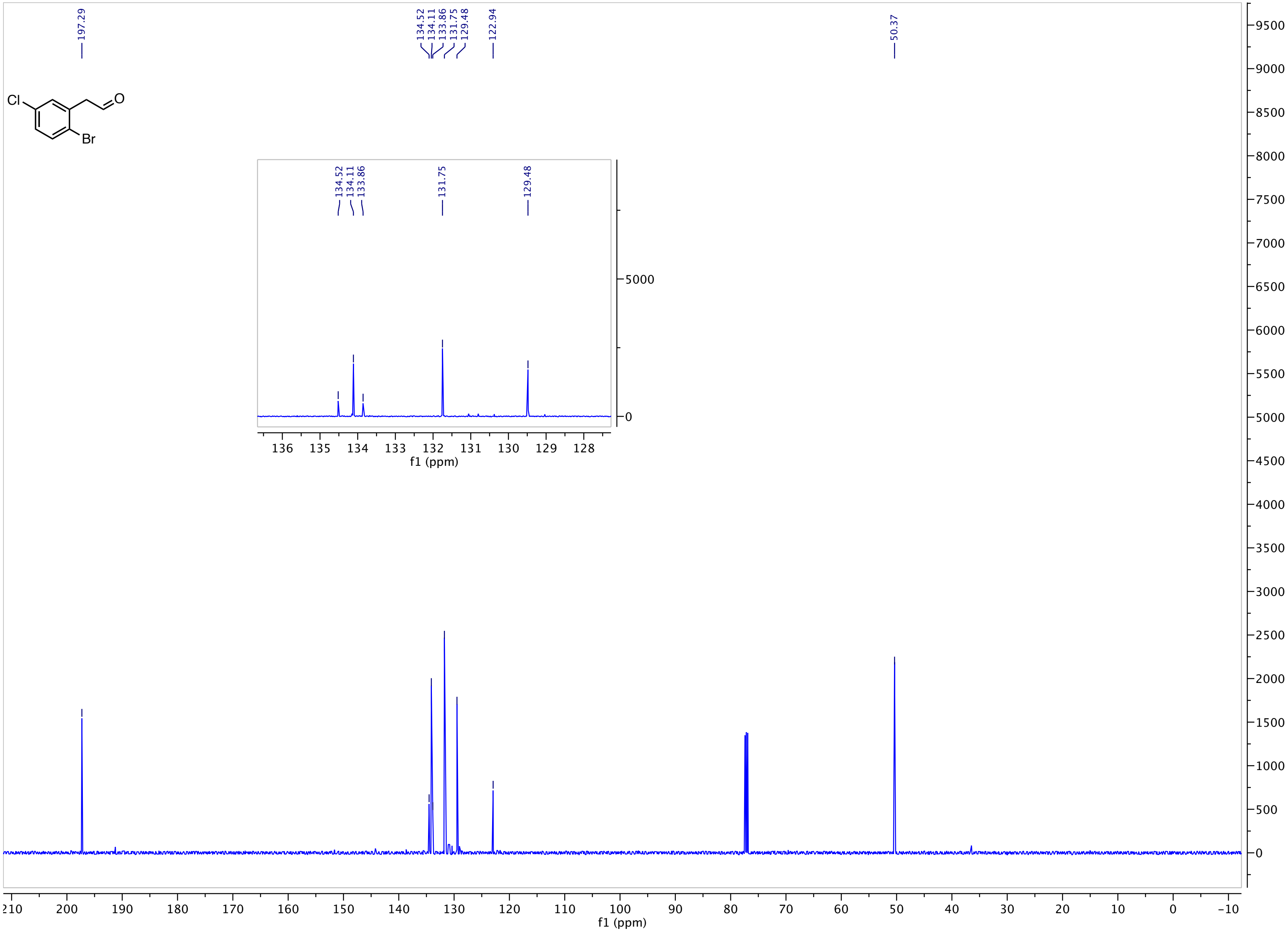

^{1}**H NMR** (Acetone-d_6): 1-(2-bromo-5-chlorophenethyl)Indolin-5-ol **(3d)**

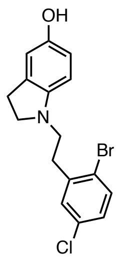

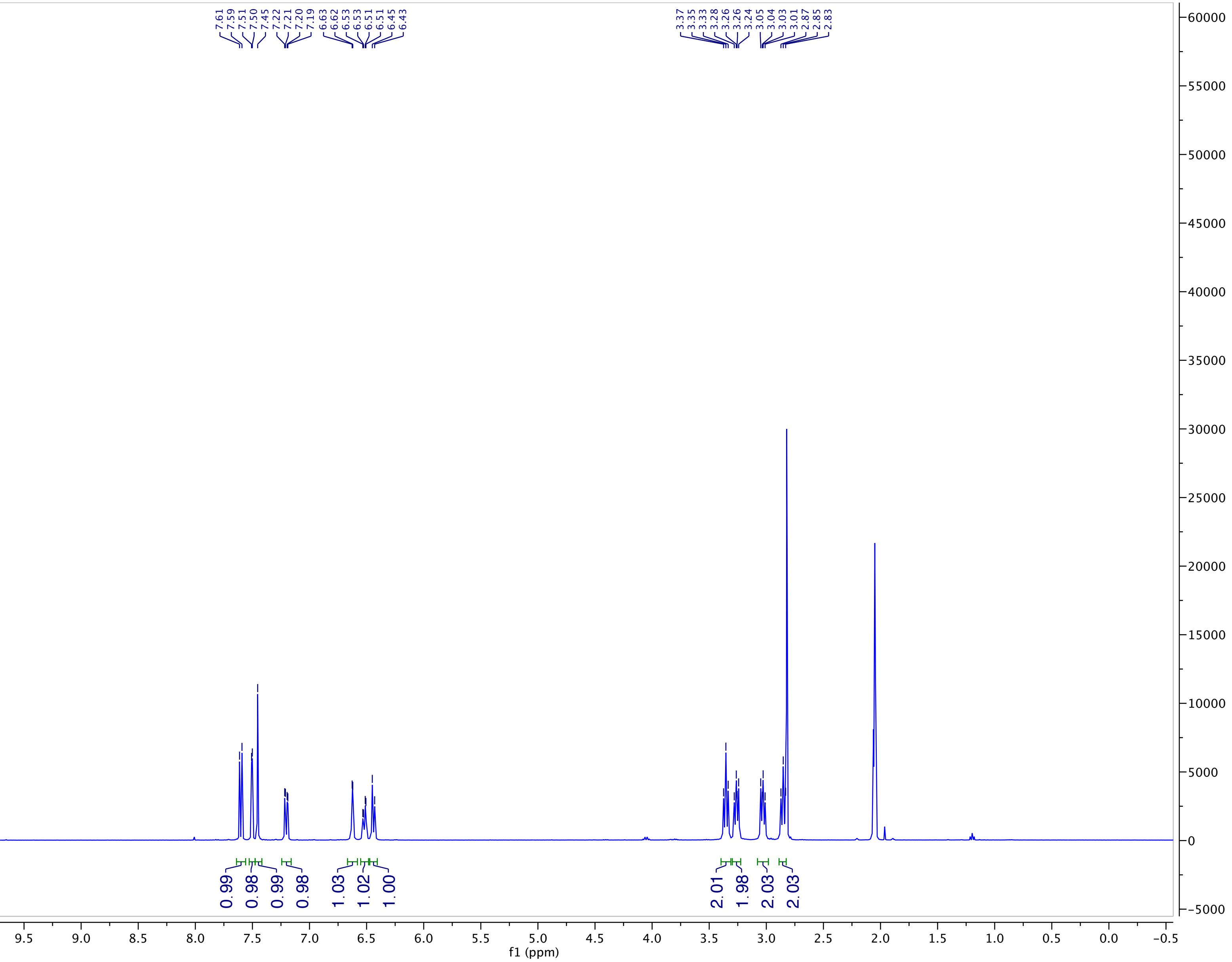

^{13}C NMR (Acetone-d_6): 1-(2-bromo-5-chlorophenethyl)Indolin-5-ol **(3d)**

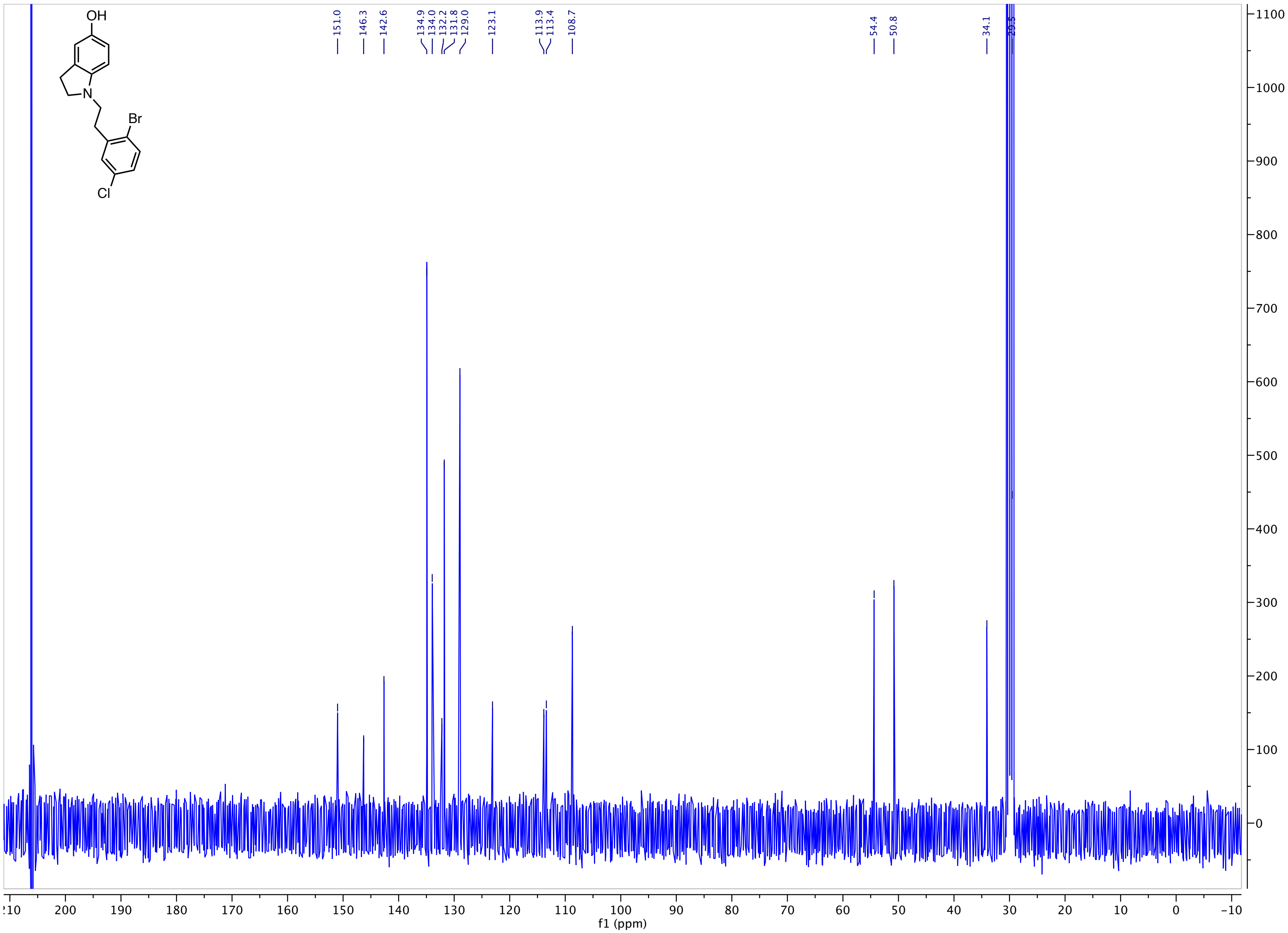

^{1}H NMR ($CDCl_3$): 2-(2-bromo-5-(trifluoromethyl)phenyl)Acetaldehyde **(S30)**

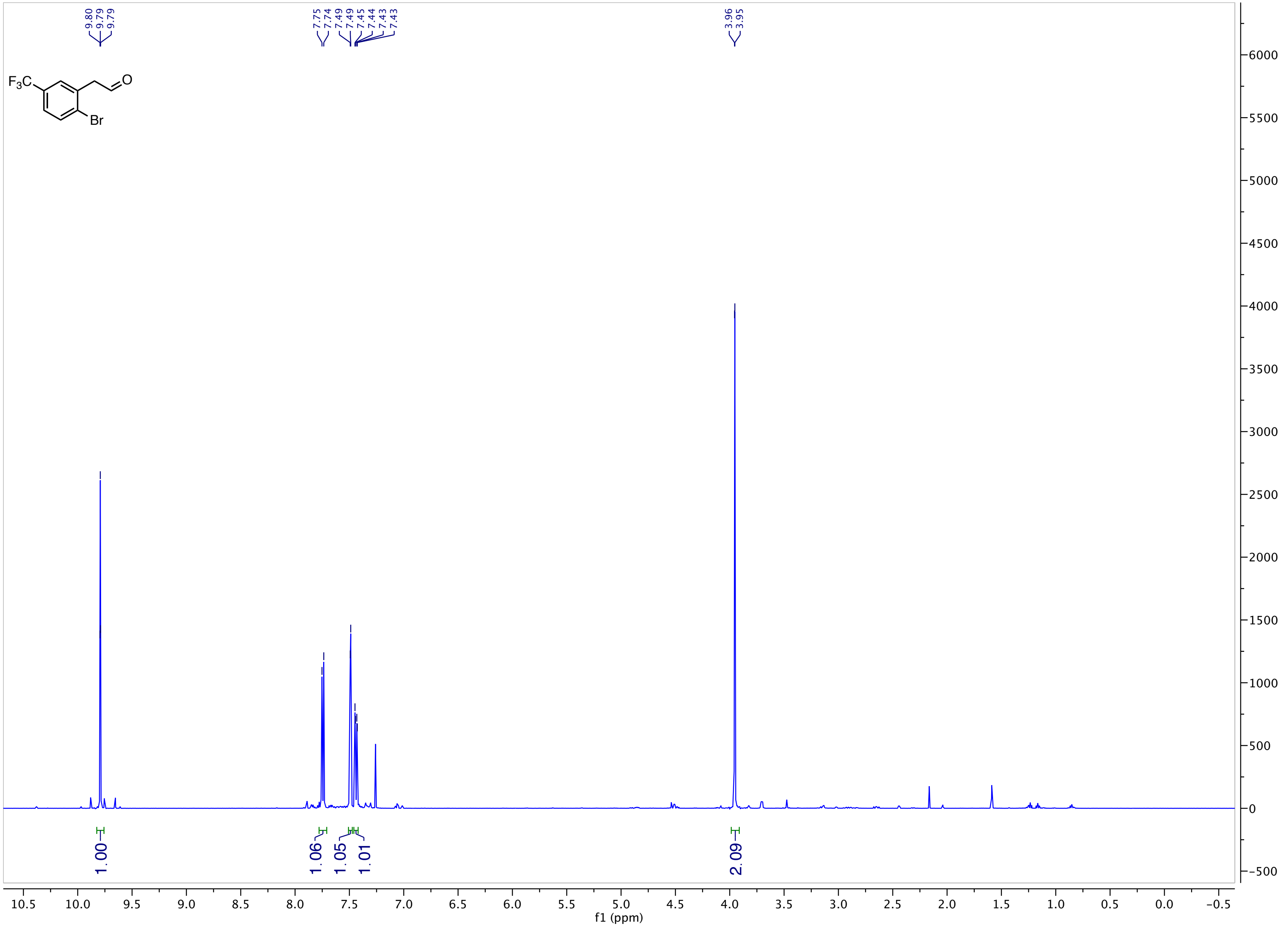

^{13}C NMR ($CDCl_3$): 2-(2-bromo-5-(trifluoromethyl)phenyl)Acetaldehyde **(S30)**

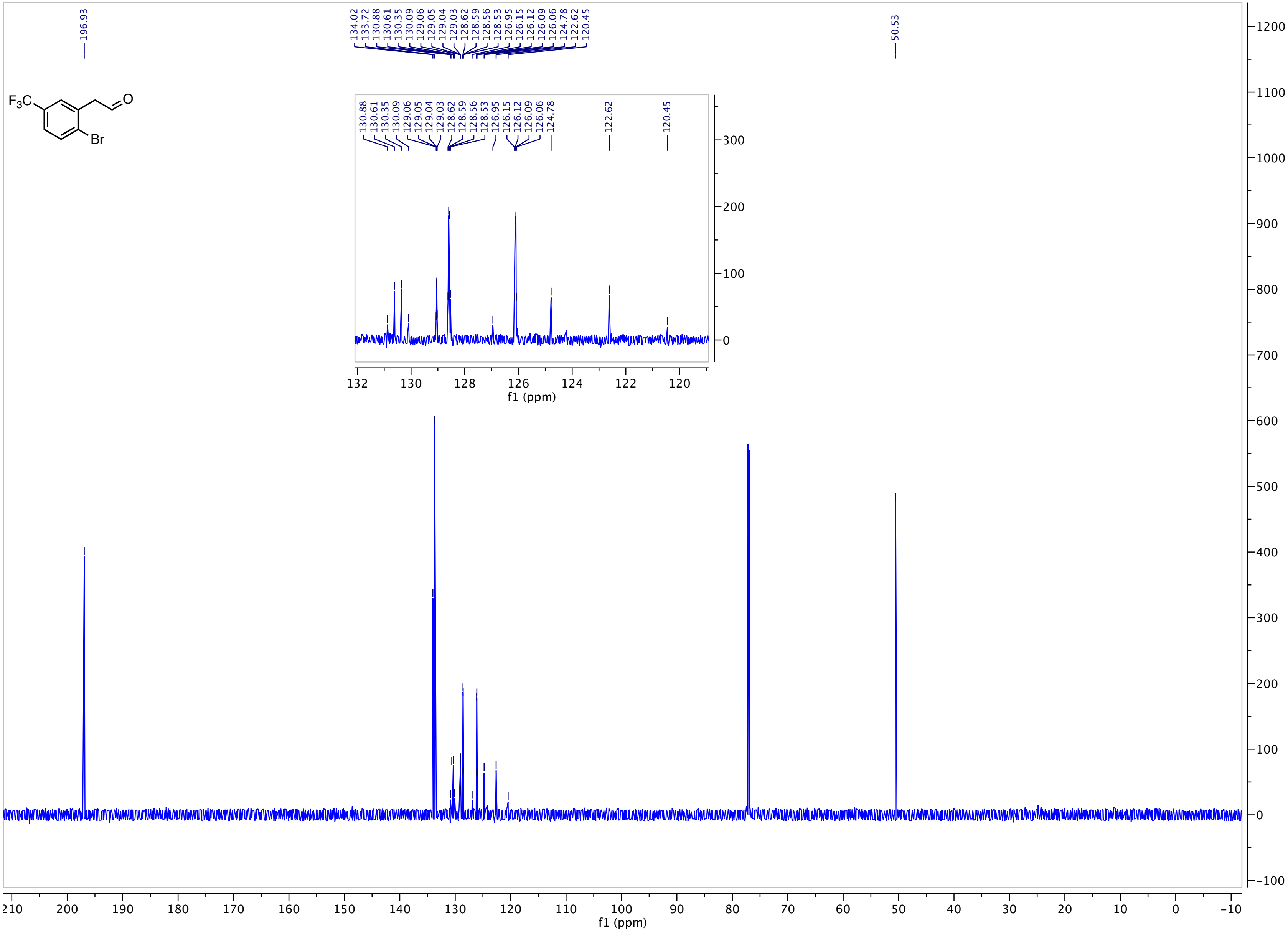

^{1}H NMR (Acetone-d_6): 1-(2-bromo-5-(trifluoromethyl)phenethyl)Indolin-5-ol **(3e)**

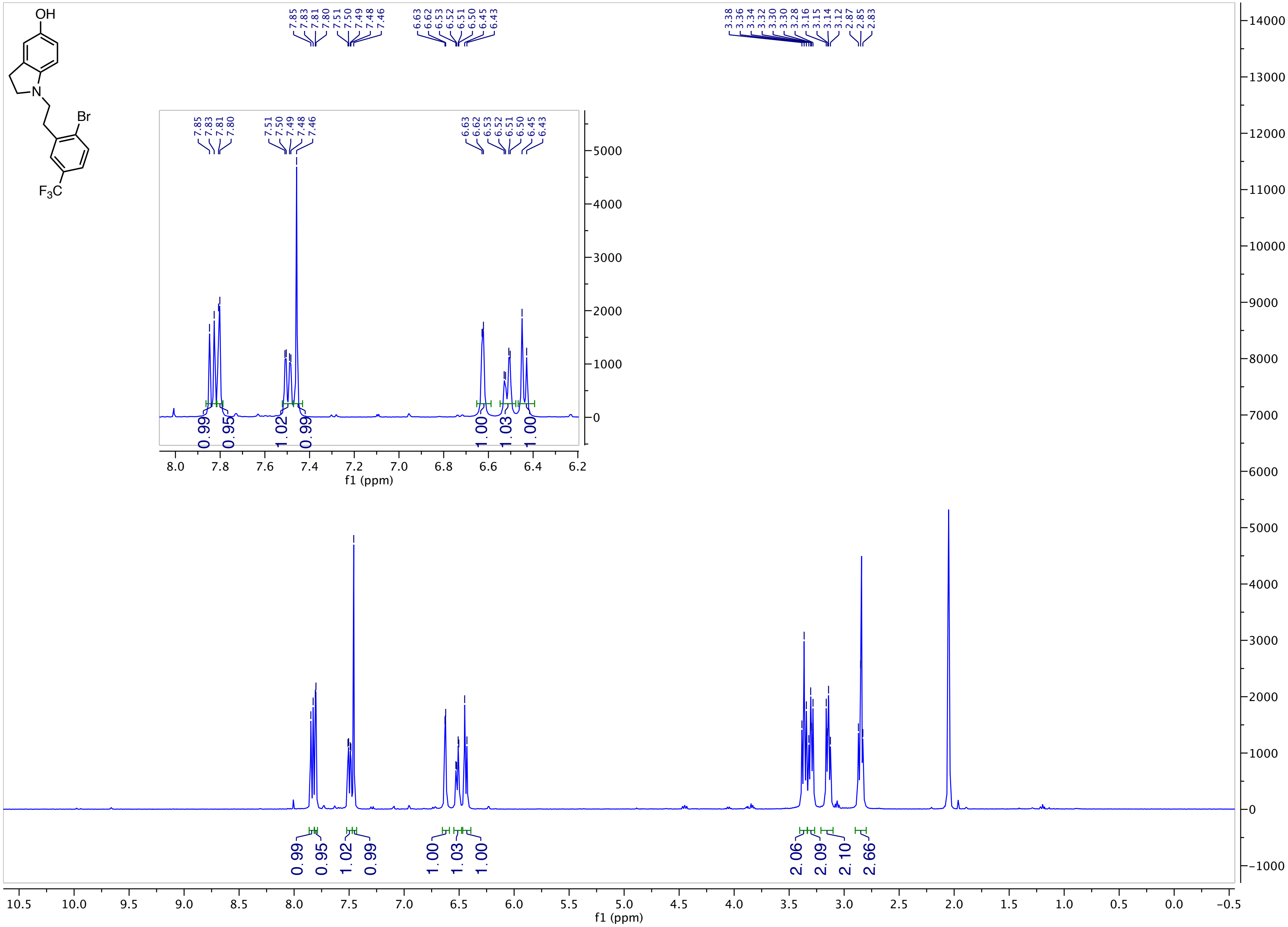

^{13}C NMR (Acetone-d_6): 1-(2-bromo-5-(trifluoromethyl)phenethyl)Indolin-5-ol **(3e)**

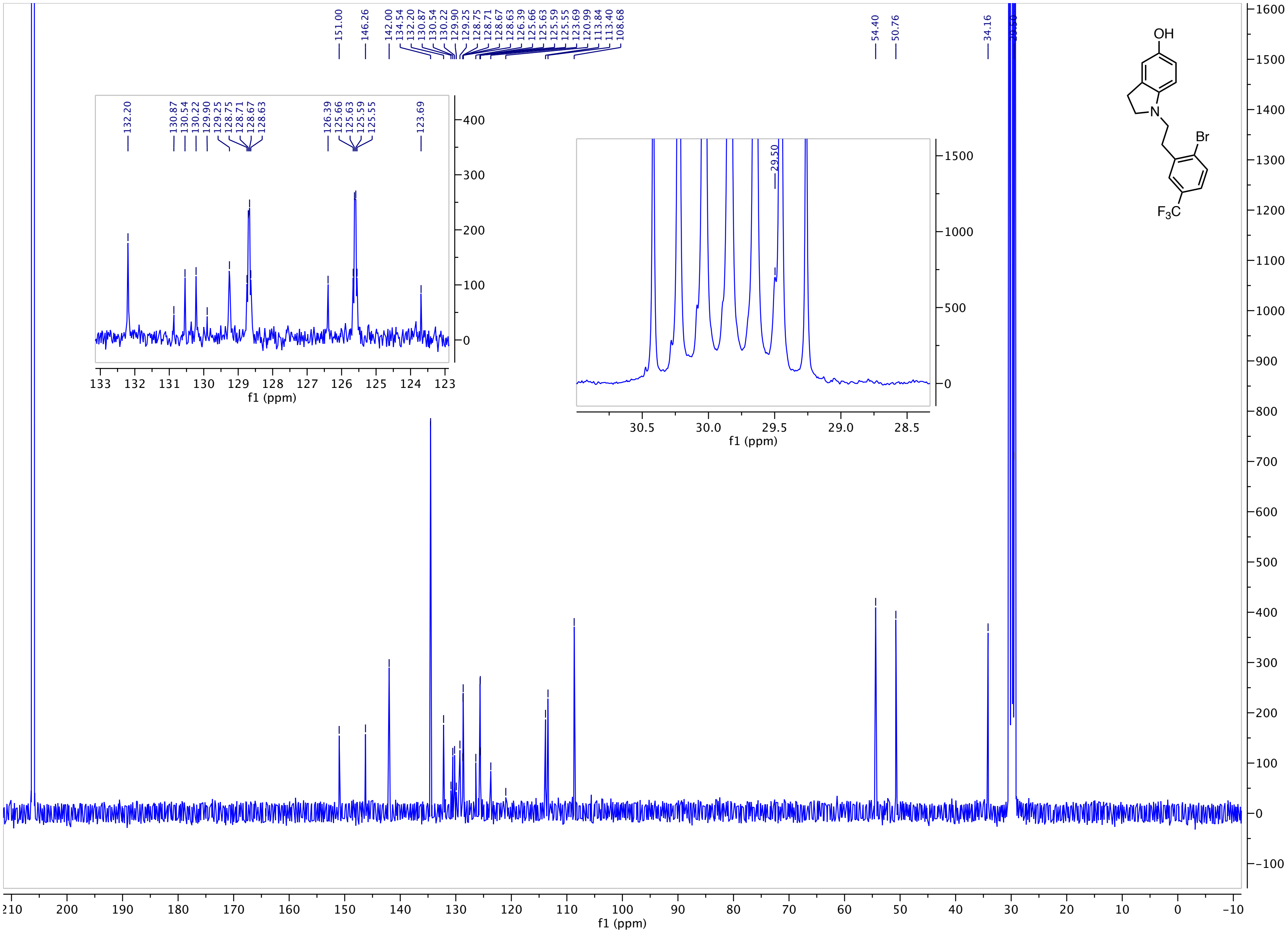

^{1}H NMR ($CDCl_3$): 2-(2-bromo-4,5-dimethoxyphenyl)Acetaldehyde (S31)

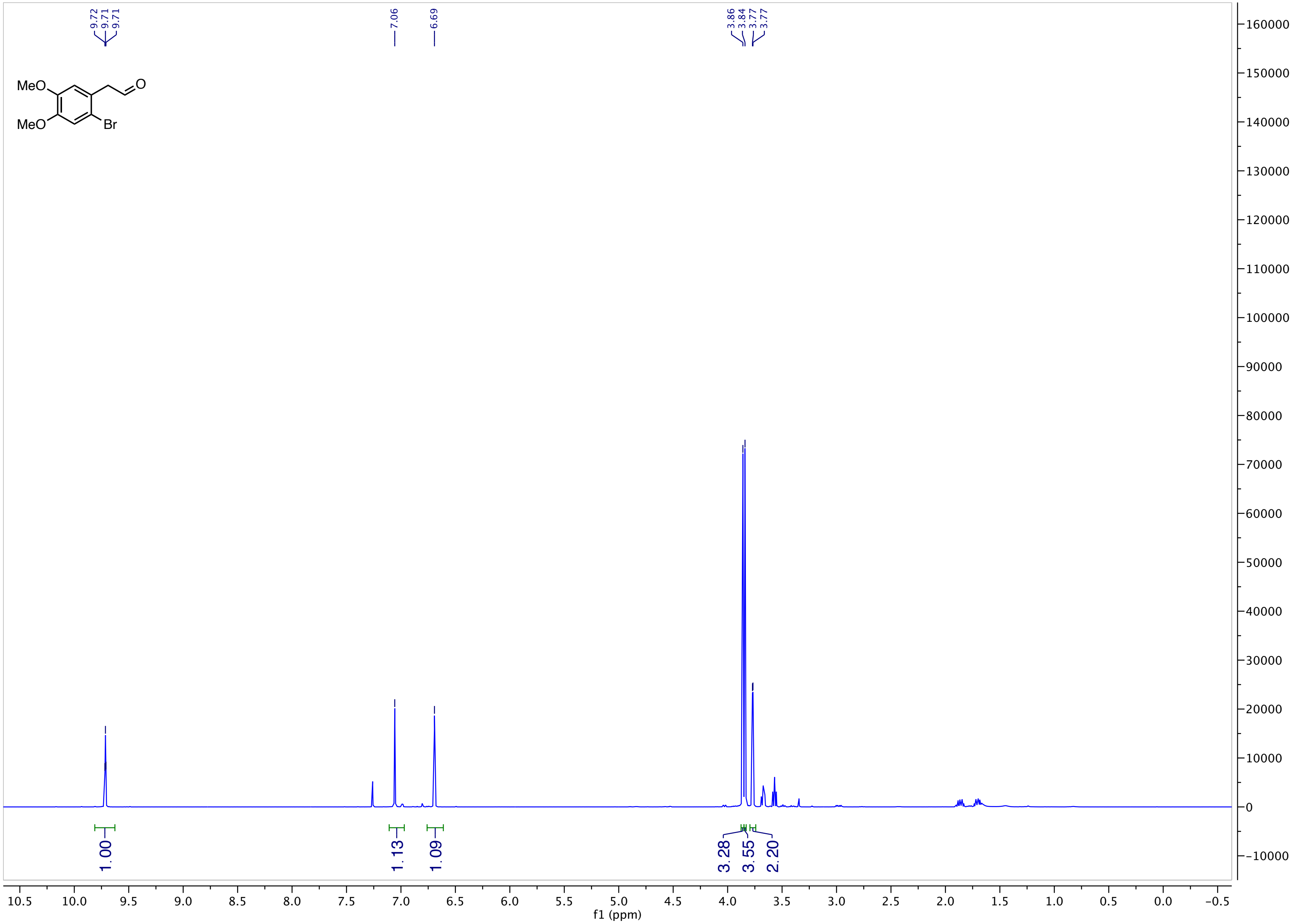

^{13}C NMR ($CDCl_3$): 2-(2-bromo-4,5-dimethoxyphenyl)Acetaldehyde **(S31)**

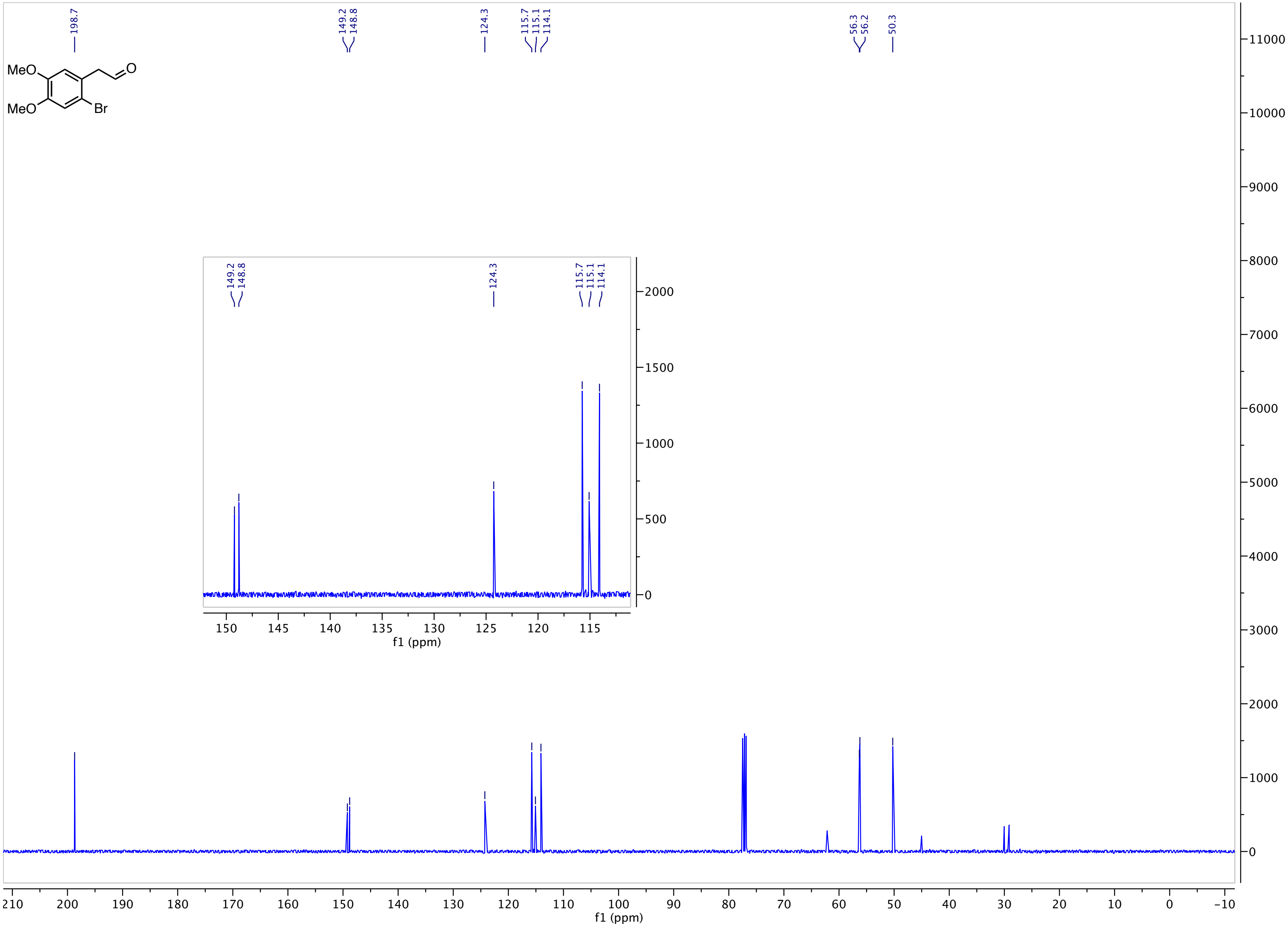

^{1}H NMR (Acetone-d_6): 1-(2-bromo-4,5-dimethoxyphenethyl)-1,2,3,4-Tetrahydroquinolin-6-ol **(3f)**

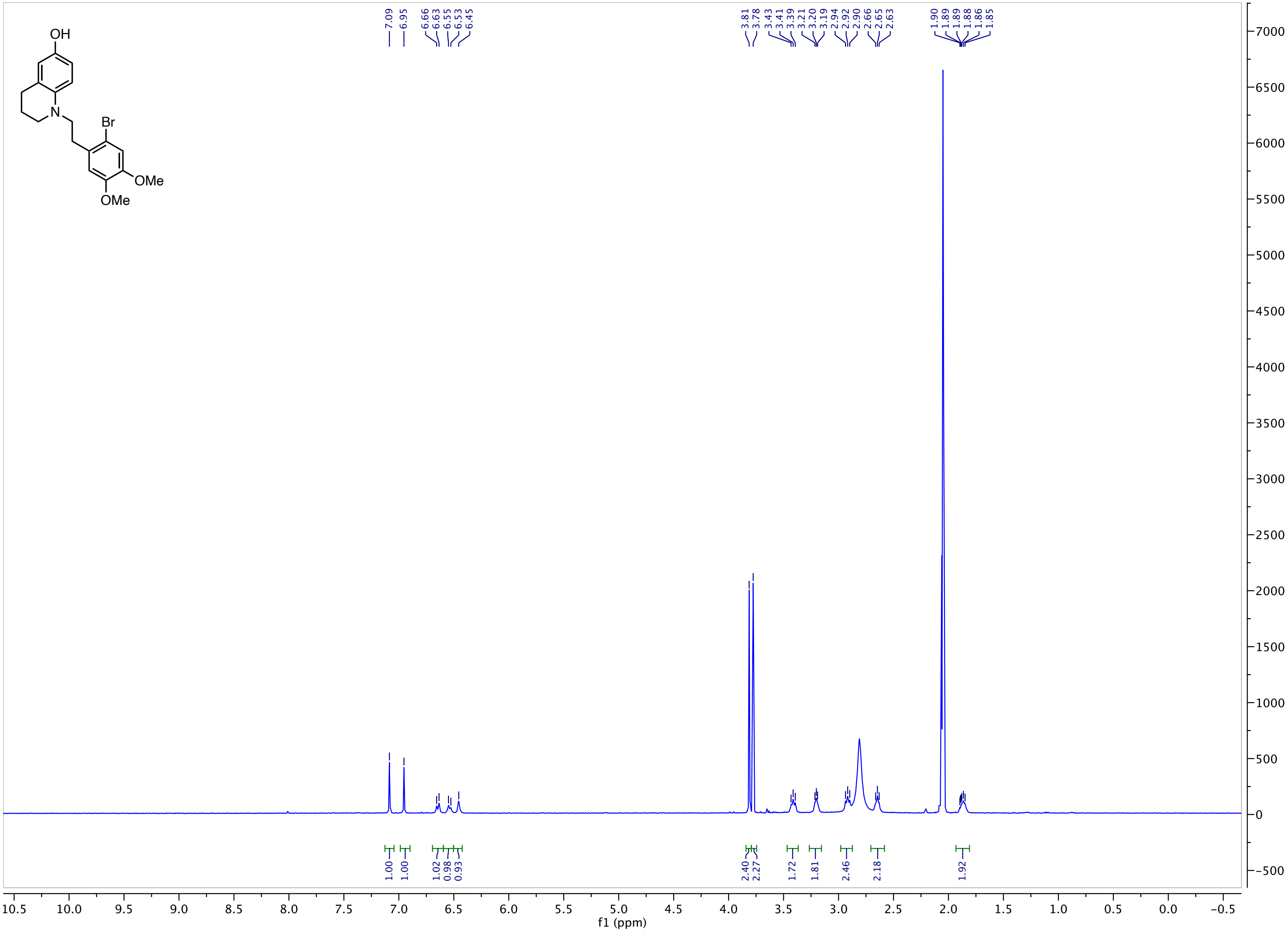

^{13}C NMR (Acetone-d_6): 1-(2-bromo-4,5-dimethoxyphenethyl)-1,2,3,4-Tetrahydroquinolin-6-ol **(3f)**

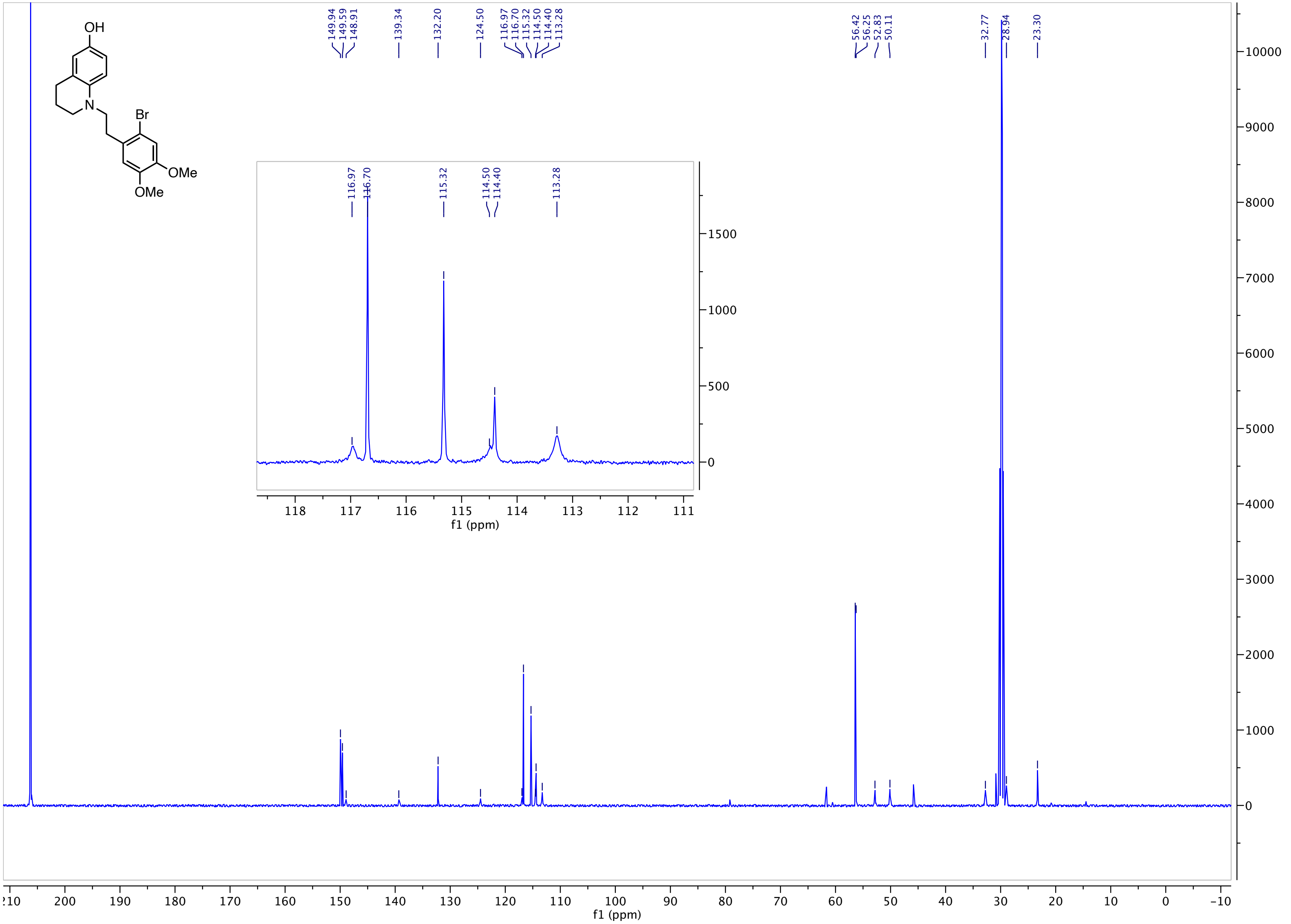

^{1}H NMR (Acetone-d_6): 1-(2-bromo-4,5-dimethoxyphenethyl)Indolin-5-ol **(3g)**

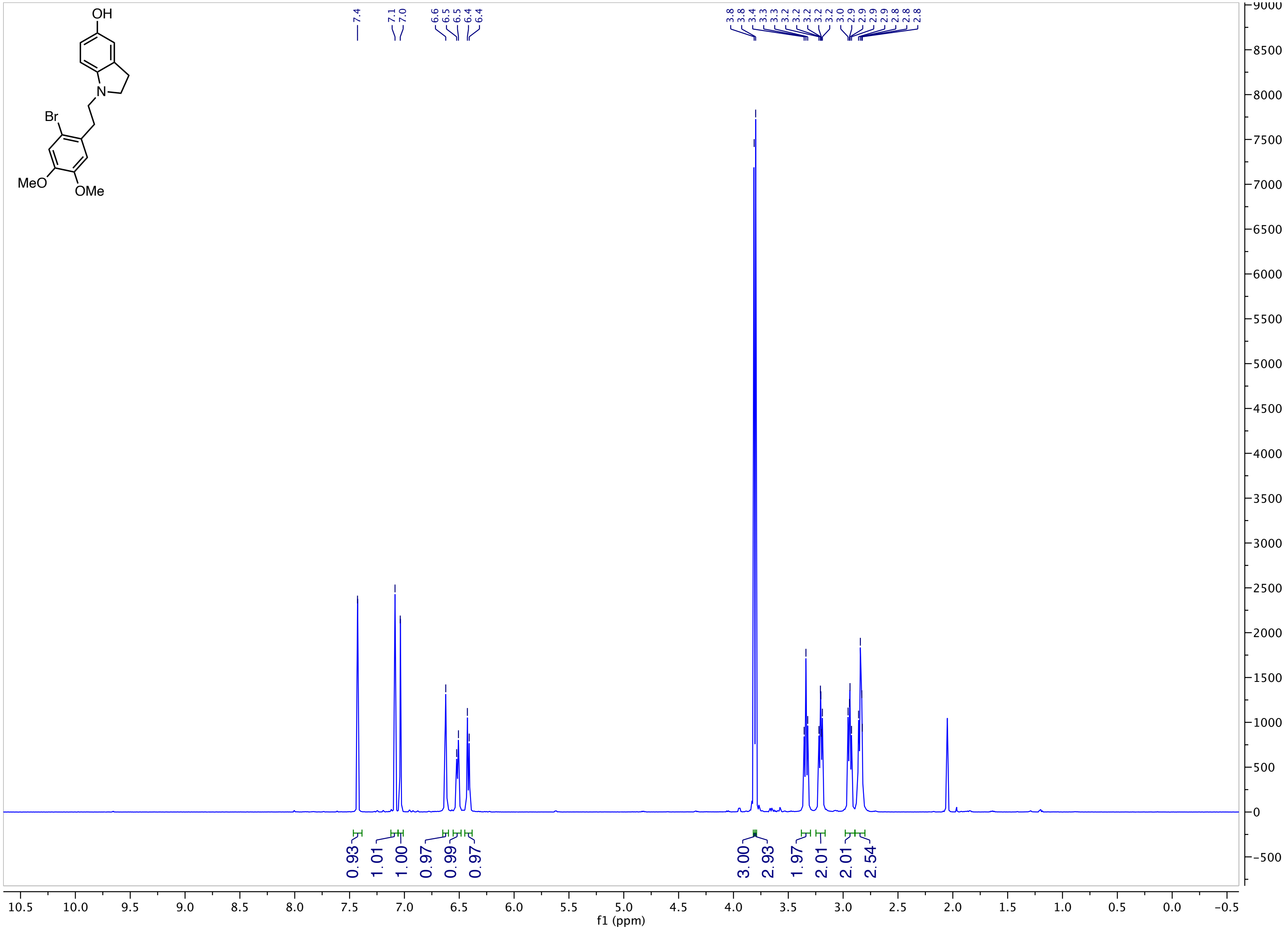

^{13}**C NMR** (Acetone-d_6): 1-(2-bromo-4,5-dimethoxyphenethyl)Indolin-5-ol **(3g)**

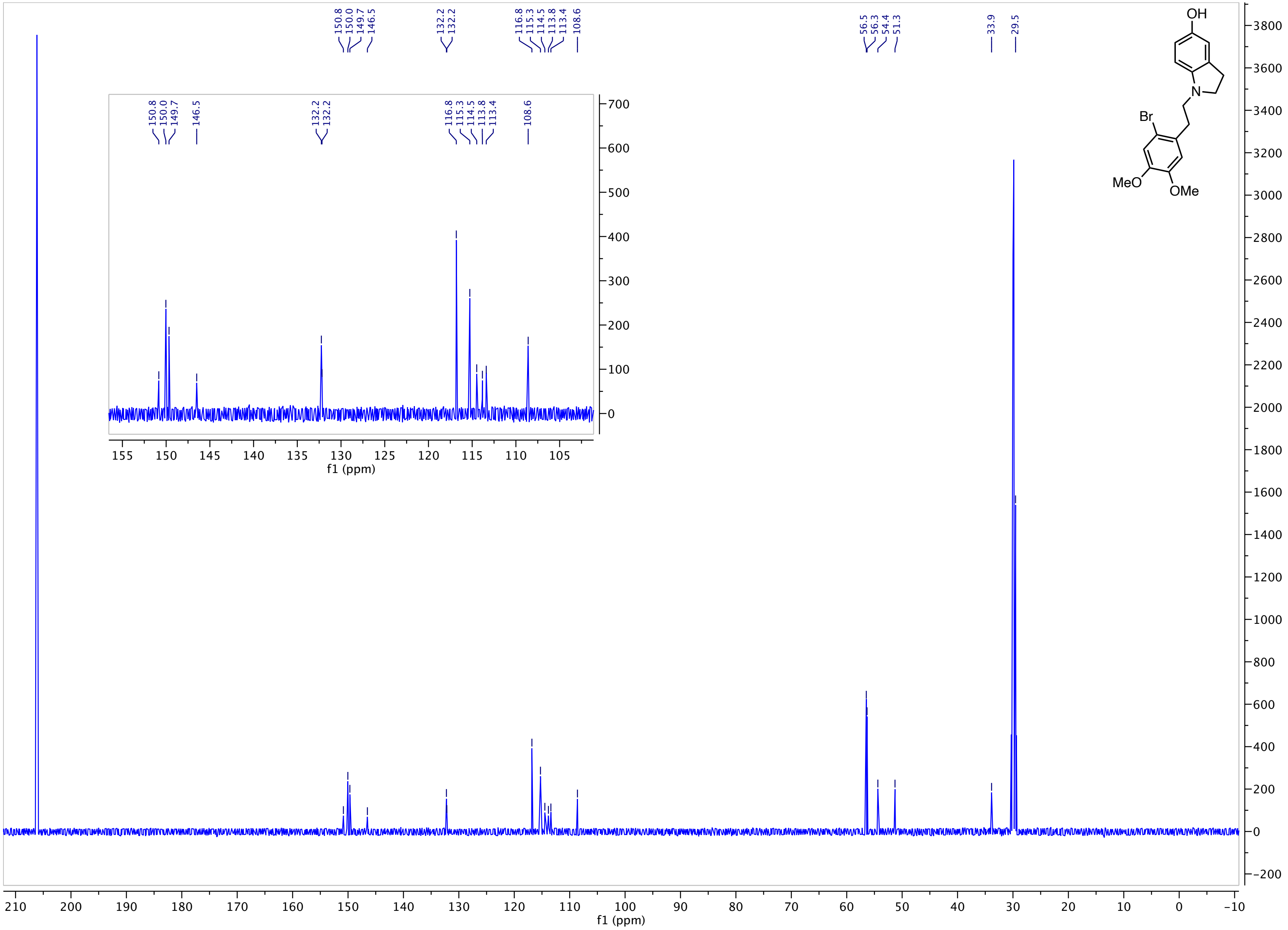

^{1}H NMR (CDCl$_3$): 5-Methoxy-2-methylbenzaldehyde **(S32)**

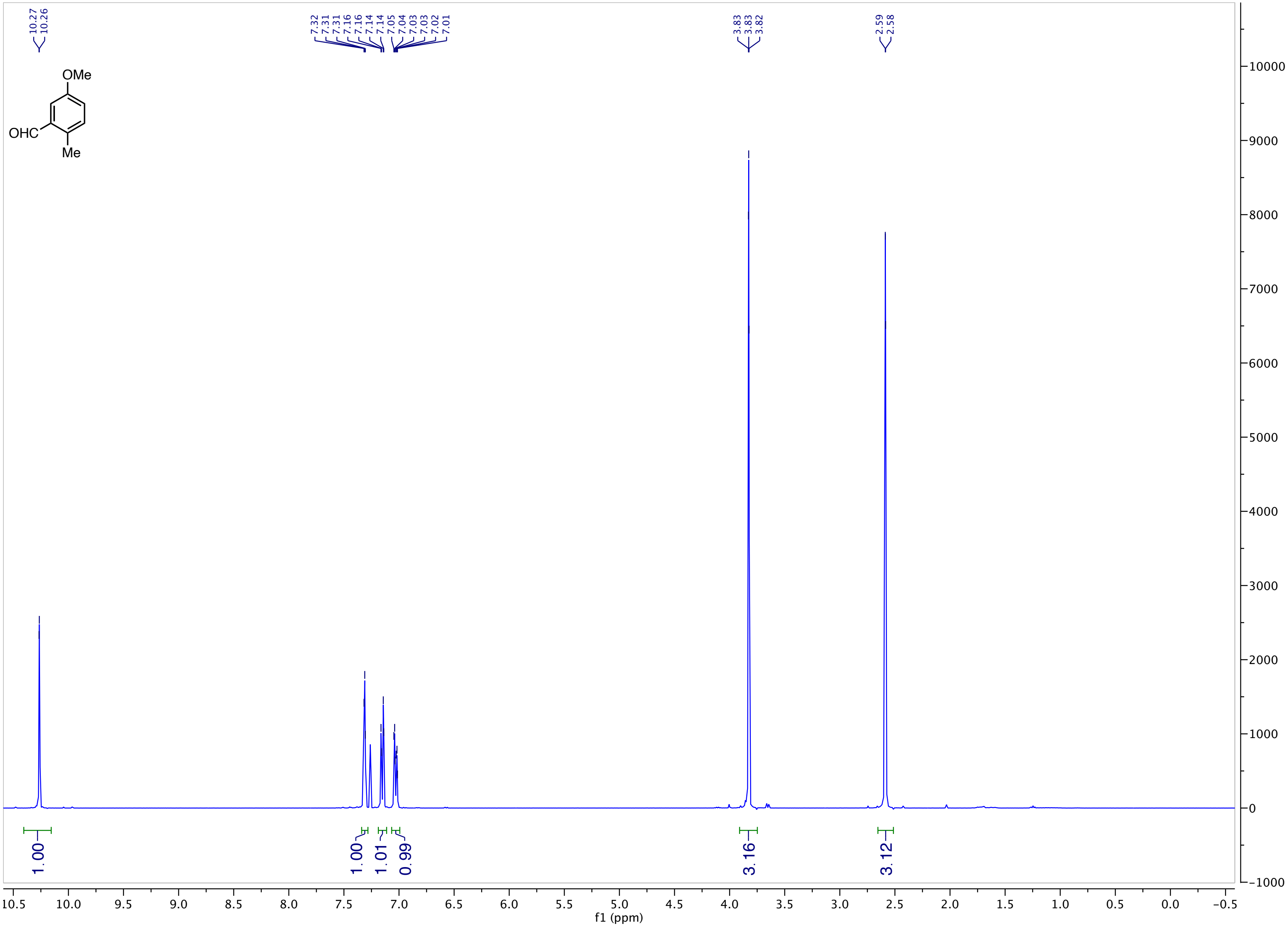

^{13}C NMR (CDCl$_3$): 5-Methoxy-2-methylbenzaldehyde **(S32)**

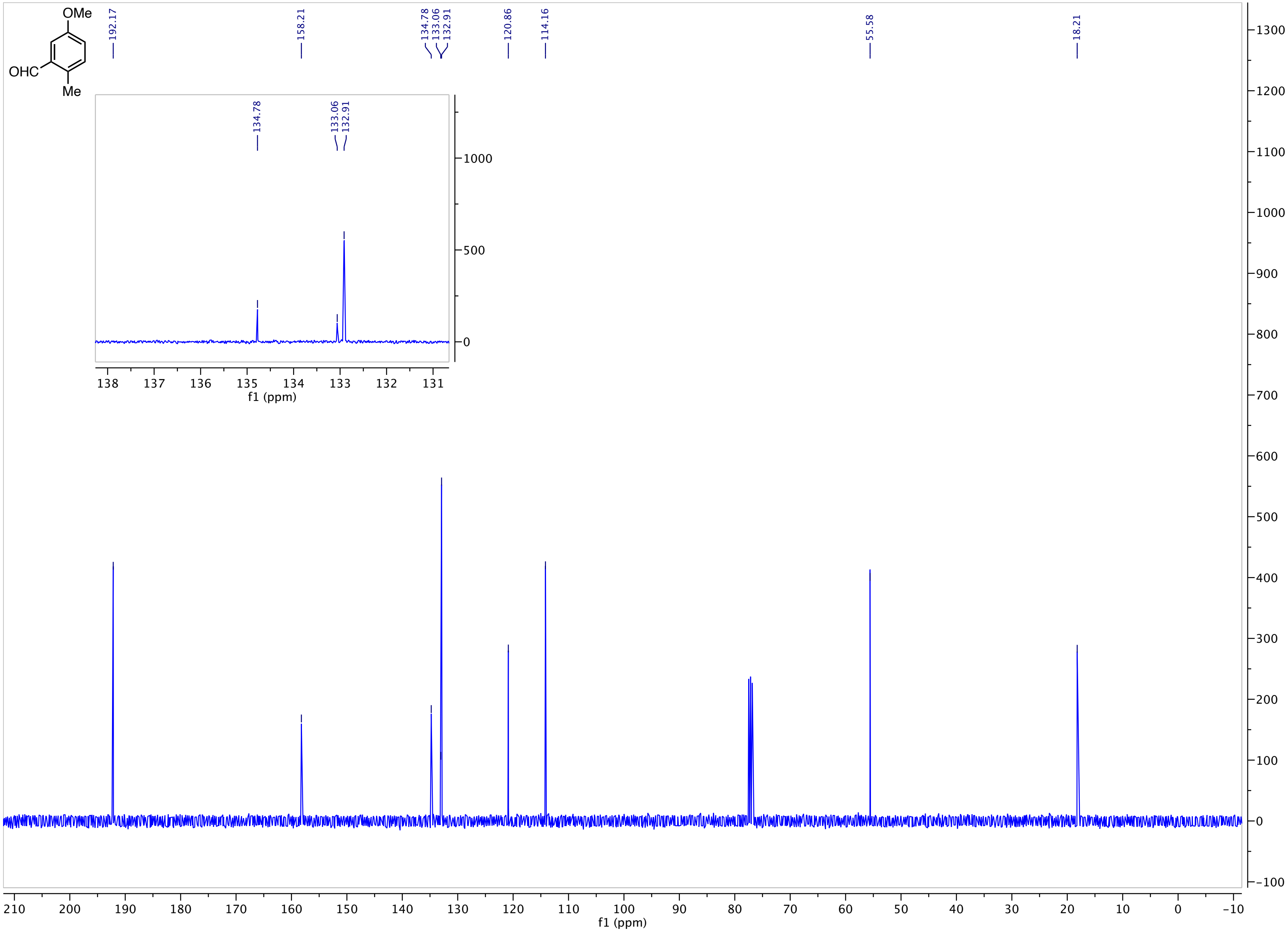

^{1}H NMR ($CDCl_3$): 3-(2-bromophenethyl)-4-Methylphenol **(6a)**

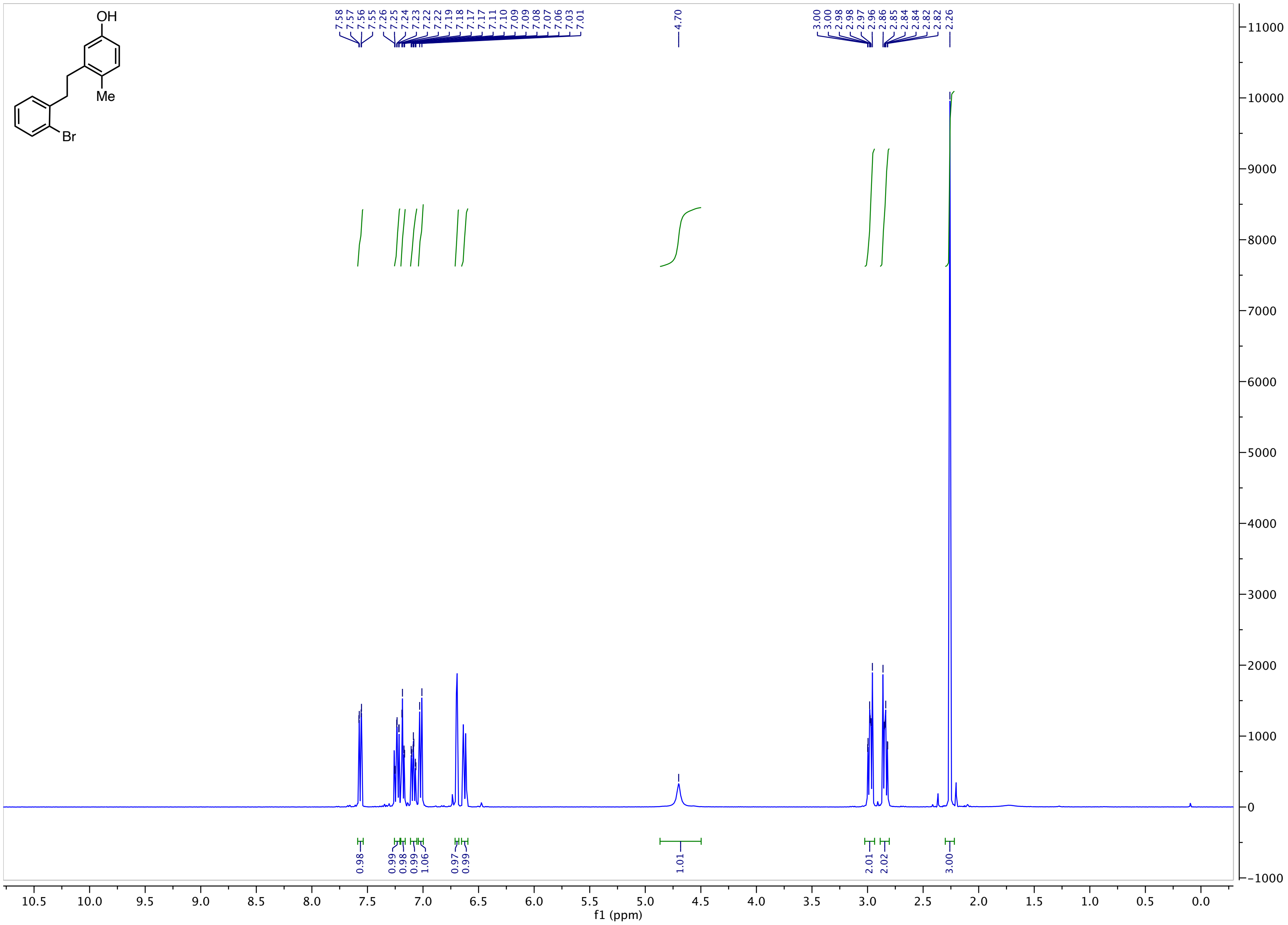

^{13}C **NMR** ($CDCl_3$): 3-(2-bromophenethyl)-4-Methylphenol **(6a)**

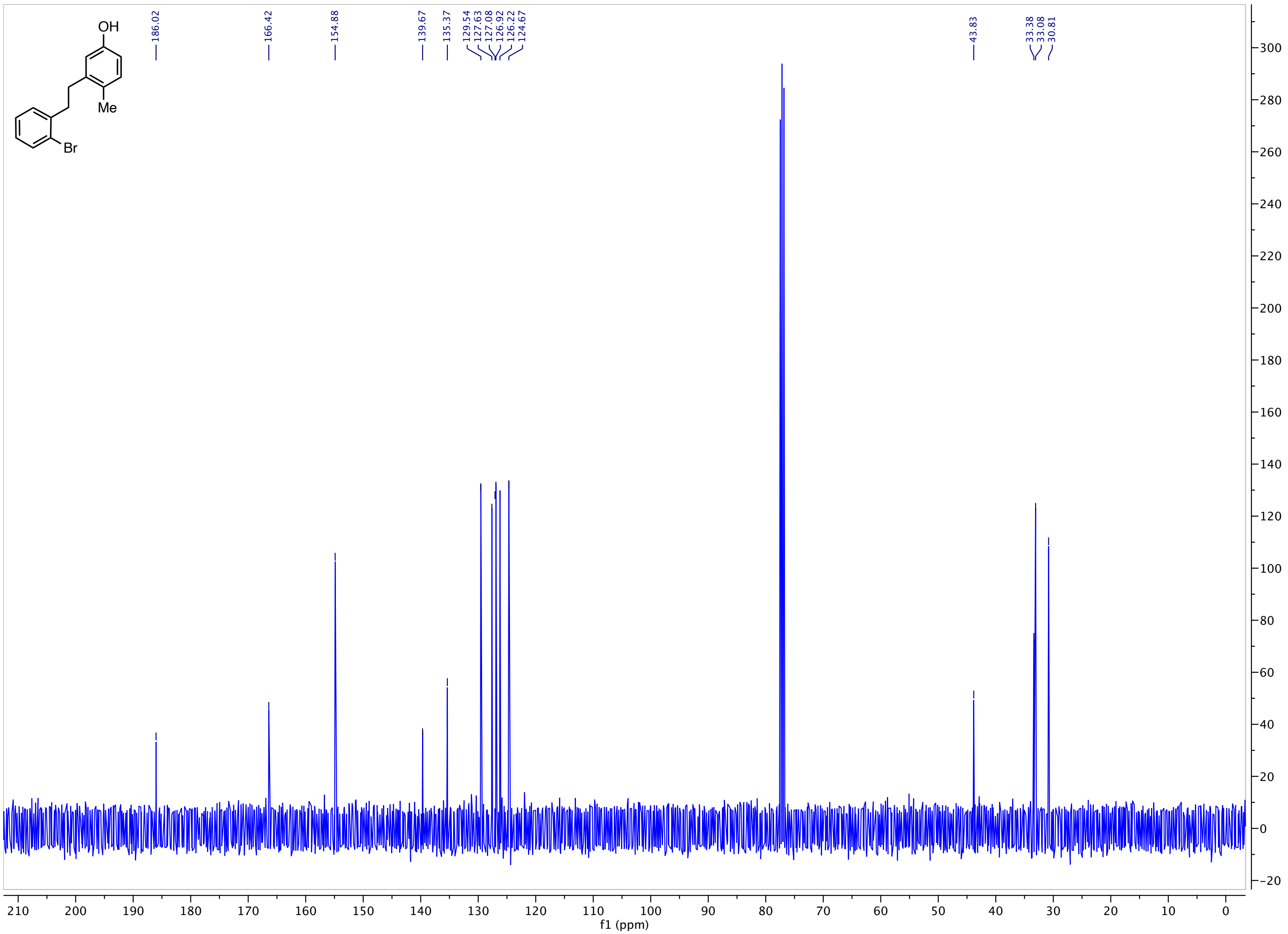

^{1}H NMR ($CDCl_3$): 2-Ethyl-5-methoxybenzaldehyde **(S33)**

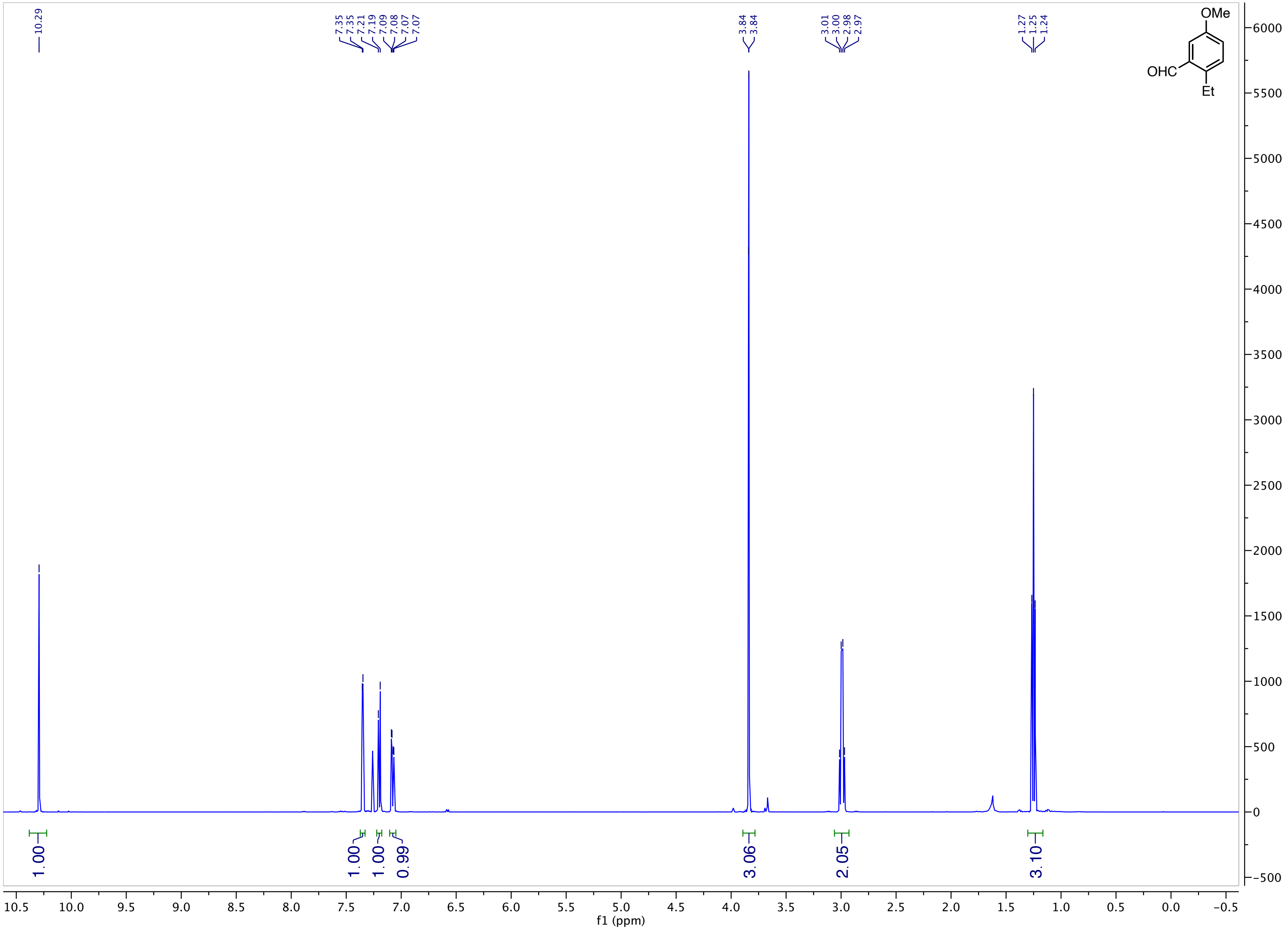

^{13}**C NMR** ($CDCl_3$): 2-Ethyl-5-methoxybenzaldehyde **(S33)**

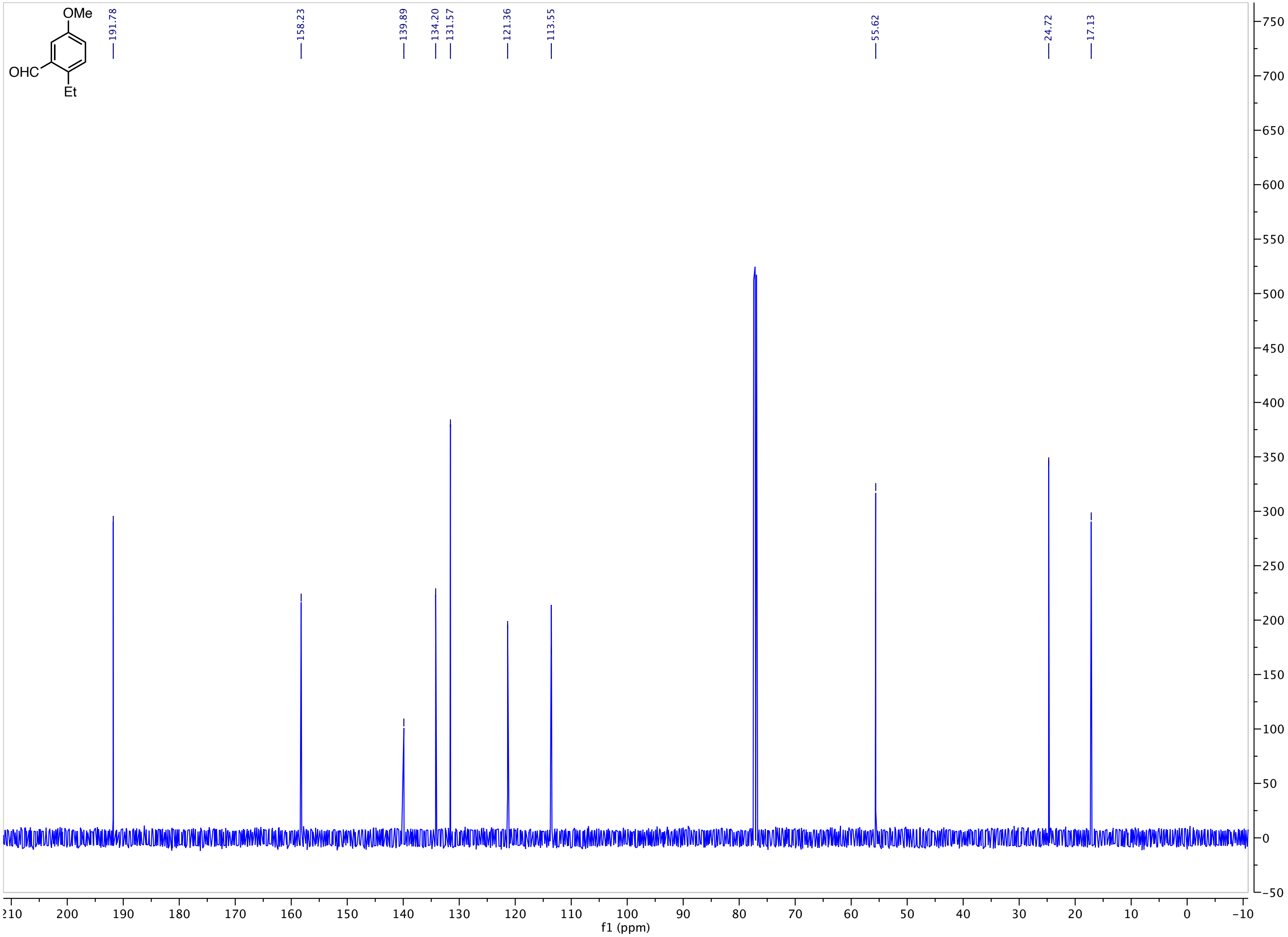

^{1}H NMR ($CDCl_3$): 3-(2-bromophenethyl)-4-Ethylphenol **(6b)**

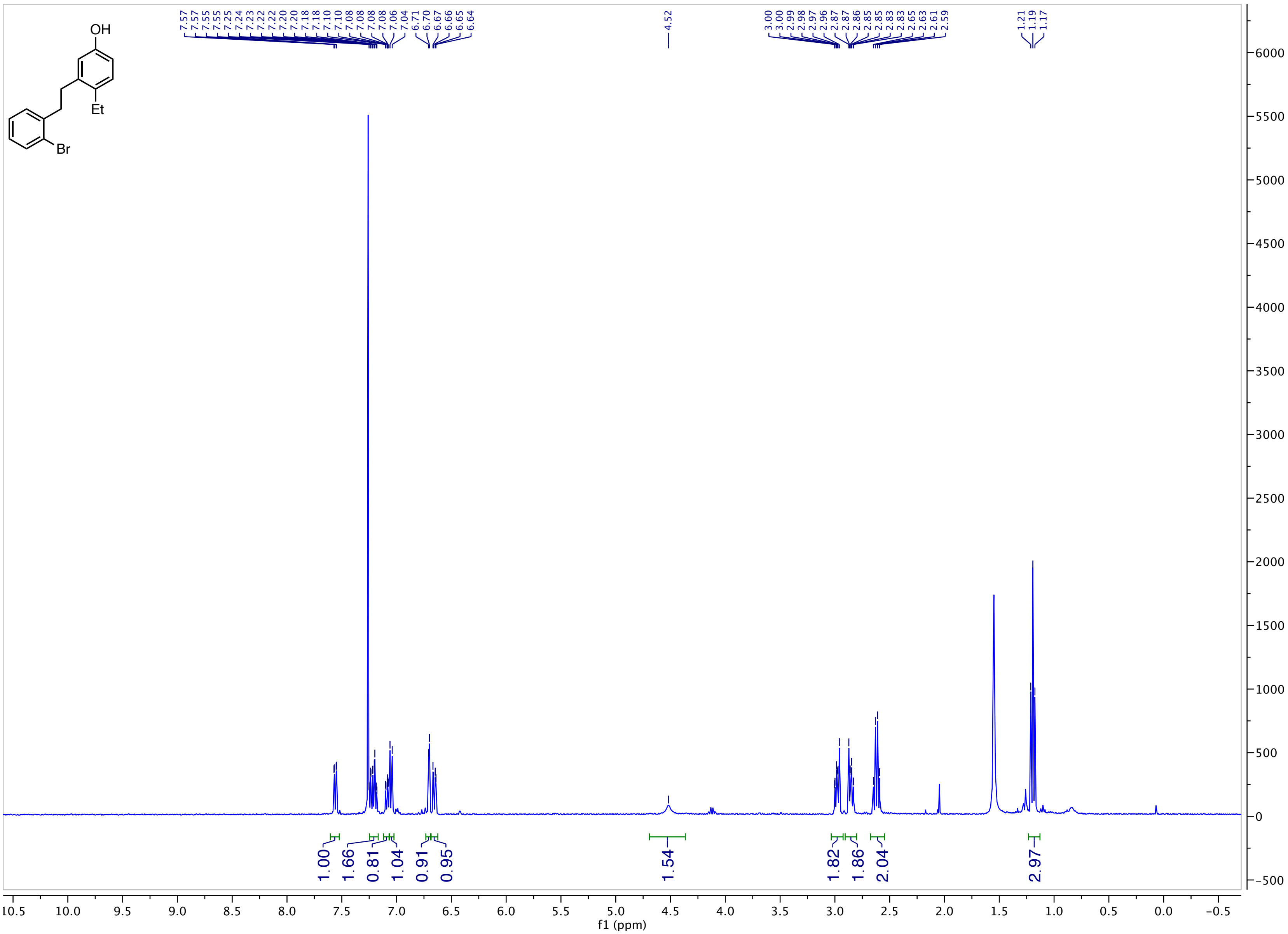

^{13}C NMR ($CDCl_3$): 3-(2-bromophenethyl)-4-Ethylphenol (6b)

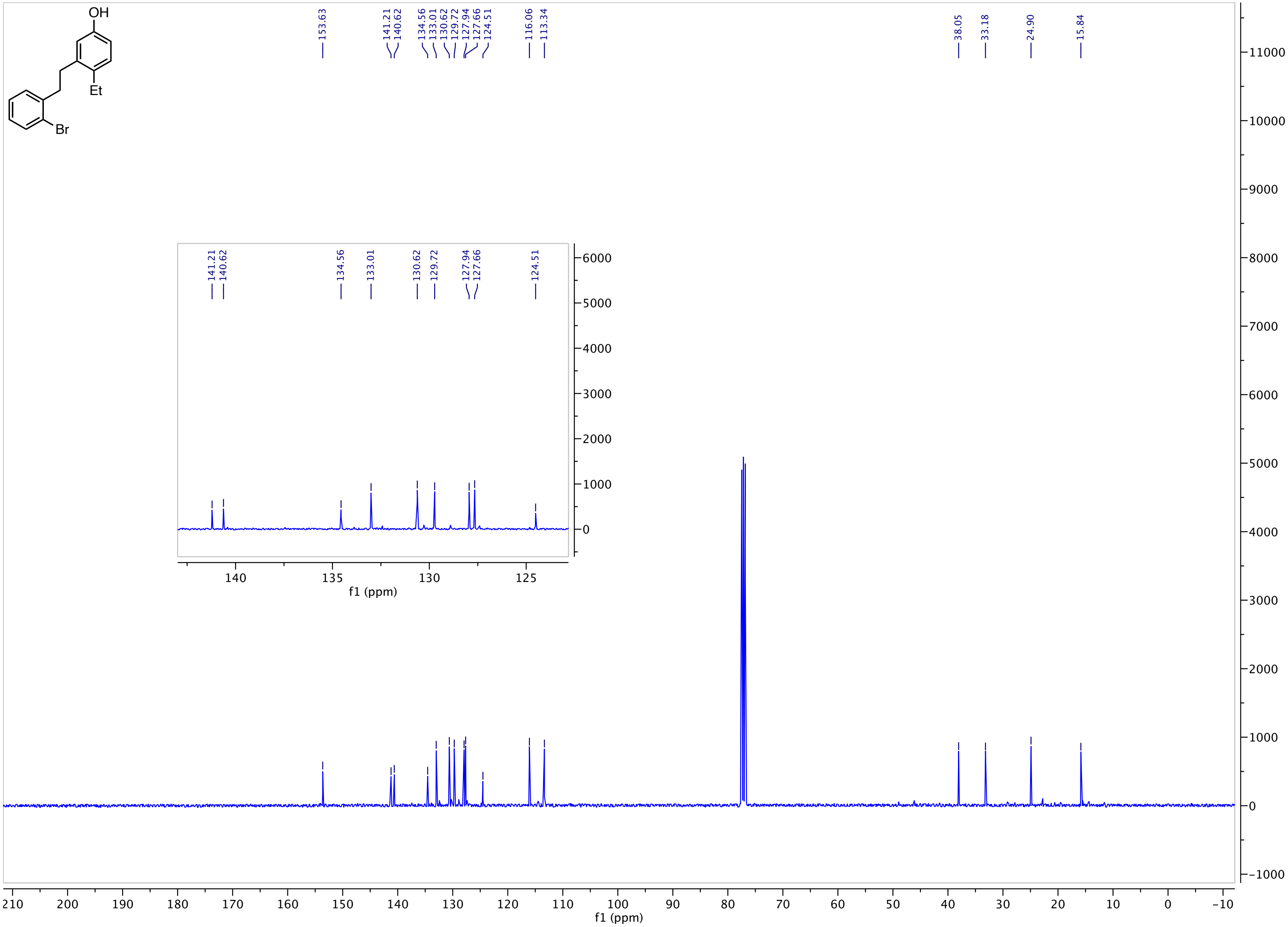

^{1}H NMR ($CDCl_3$): 3-(2-bromo-6-chlorophenethyl)-4-Methylphenol **(6c)**

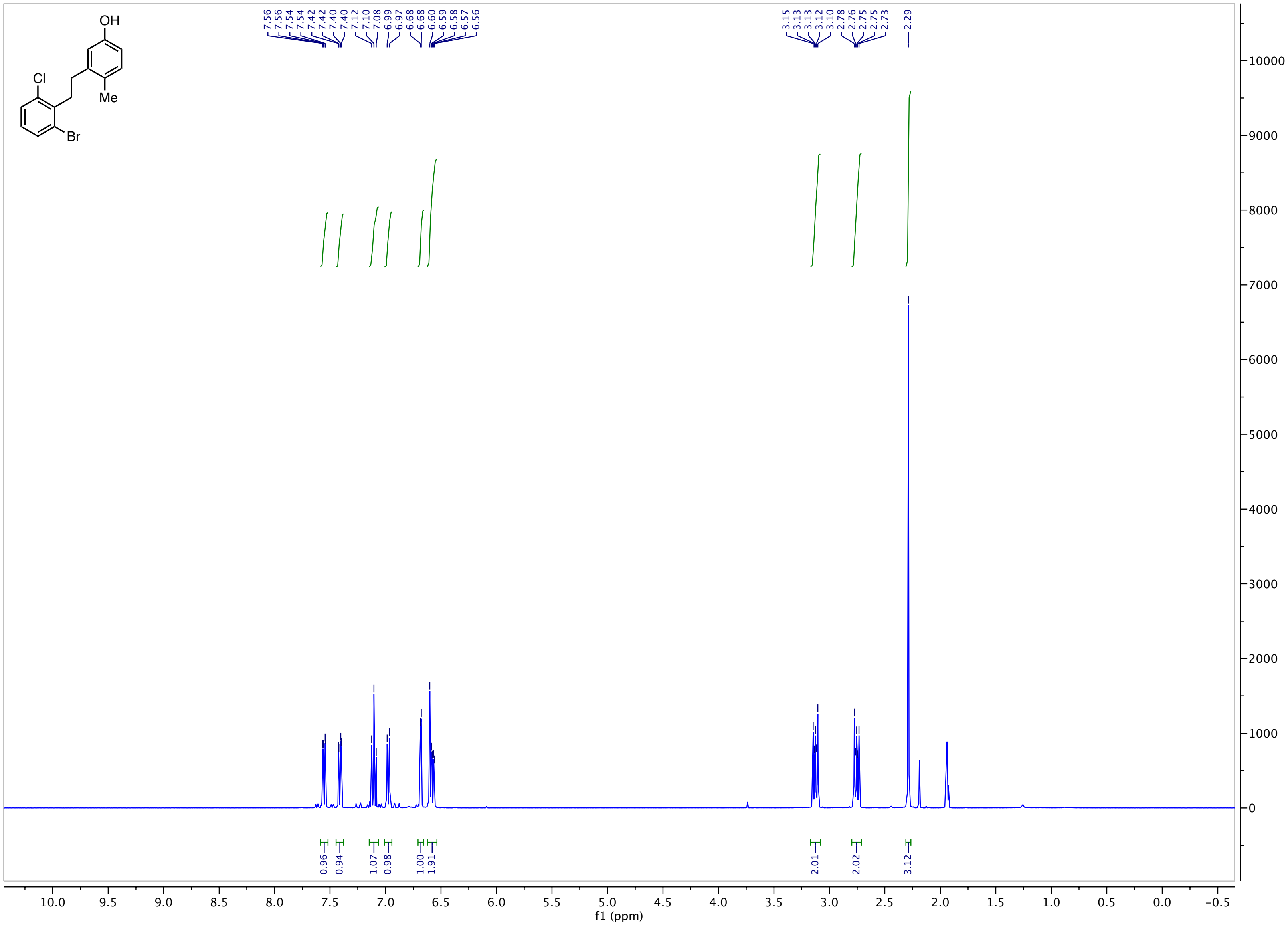

^{13}C NMR ($CDCl_3$): 3-(2-bromo-6-chlorophenethyl)-4-Methylphenol **(6c)**

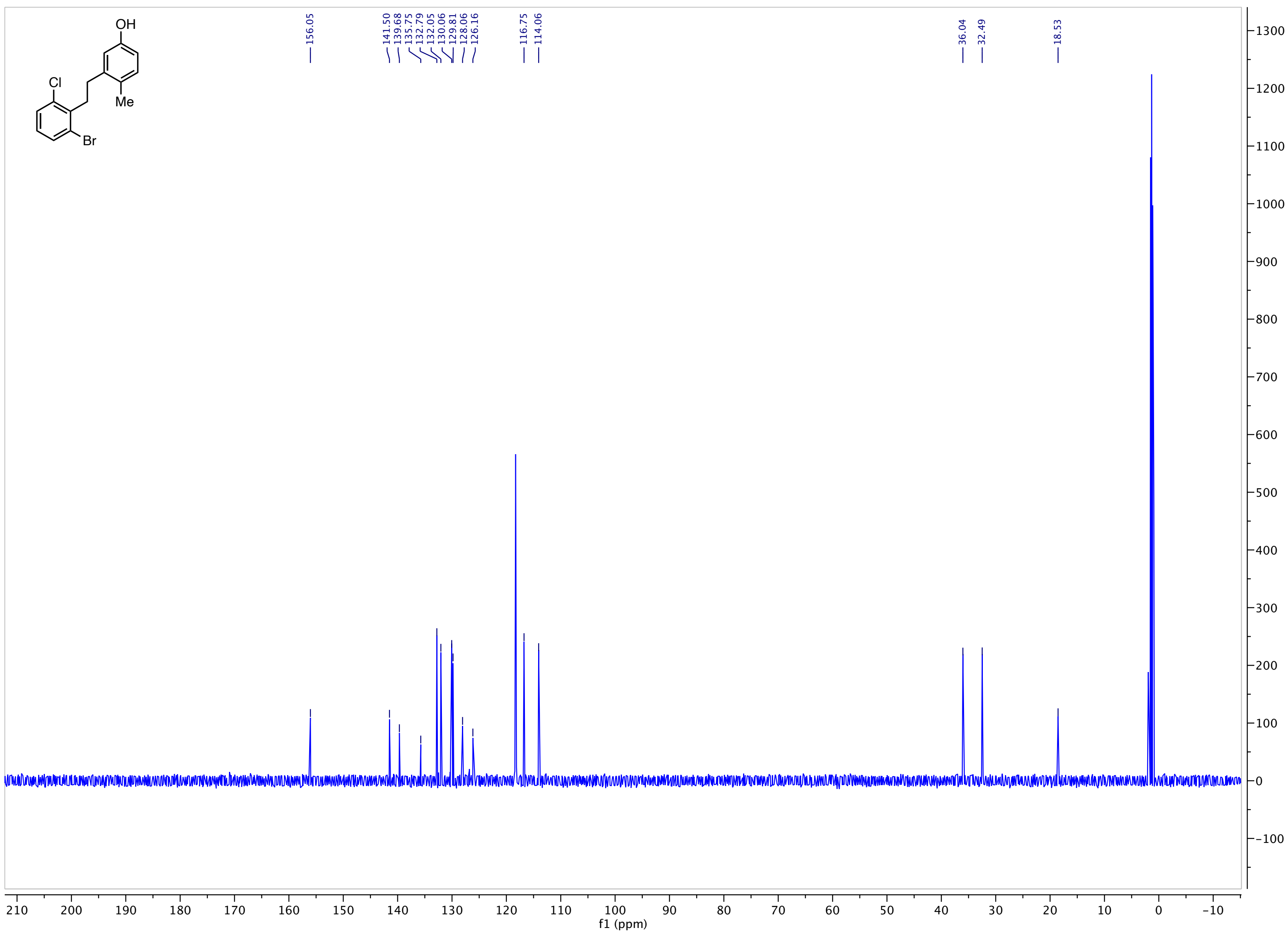

^{1}**H NMR** (CDCl$_3$): 3-(2-bromo-5-fluorophenethyl)-4-Methylphenol **(6d)**

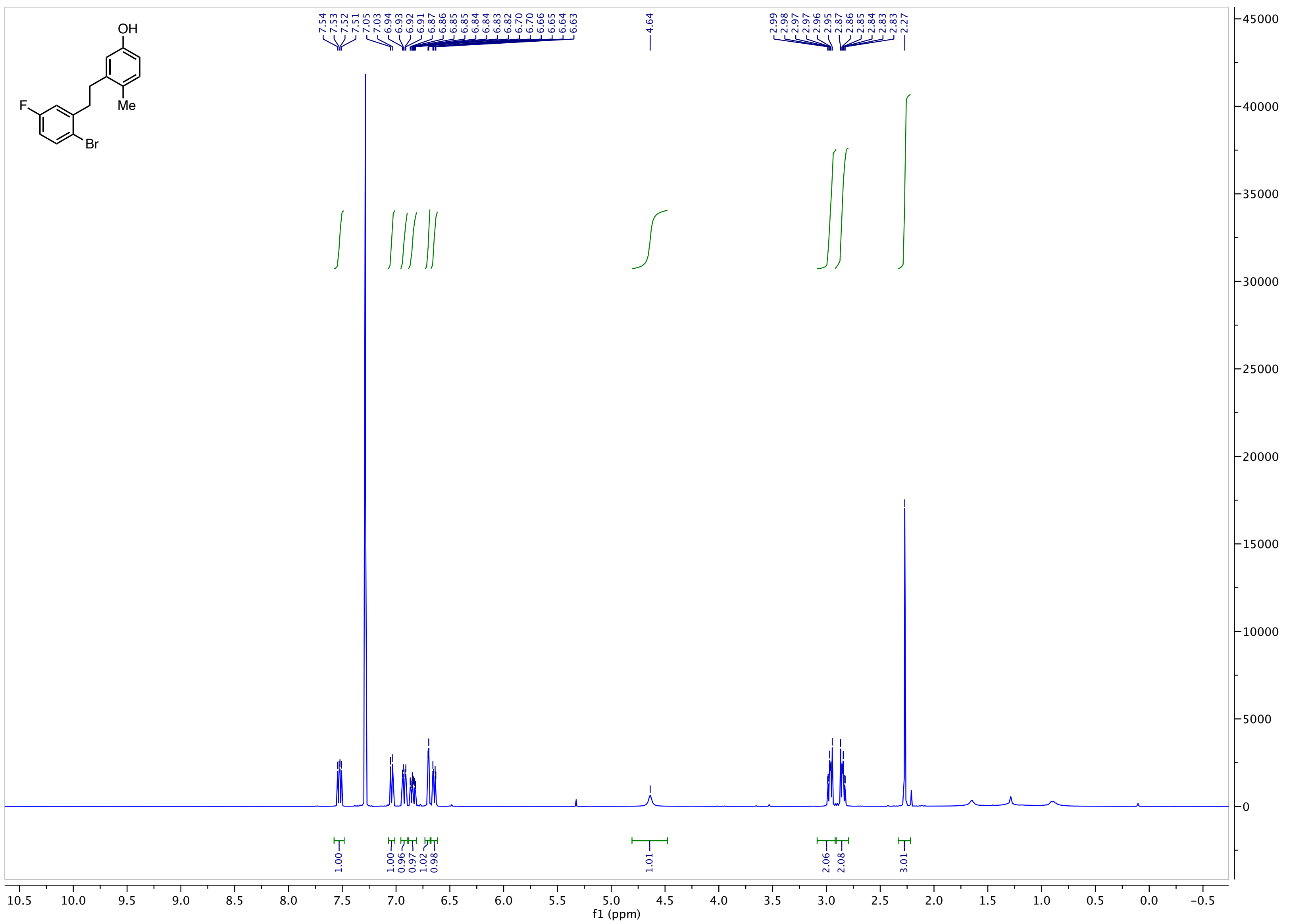

^{13}C NMR ($CDCl_3$): 3-(2-bromo-5-fluorophenethyl)-4-Methylphenol **(6d)**

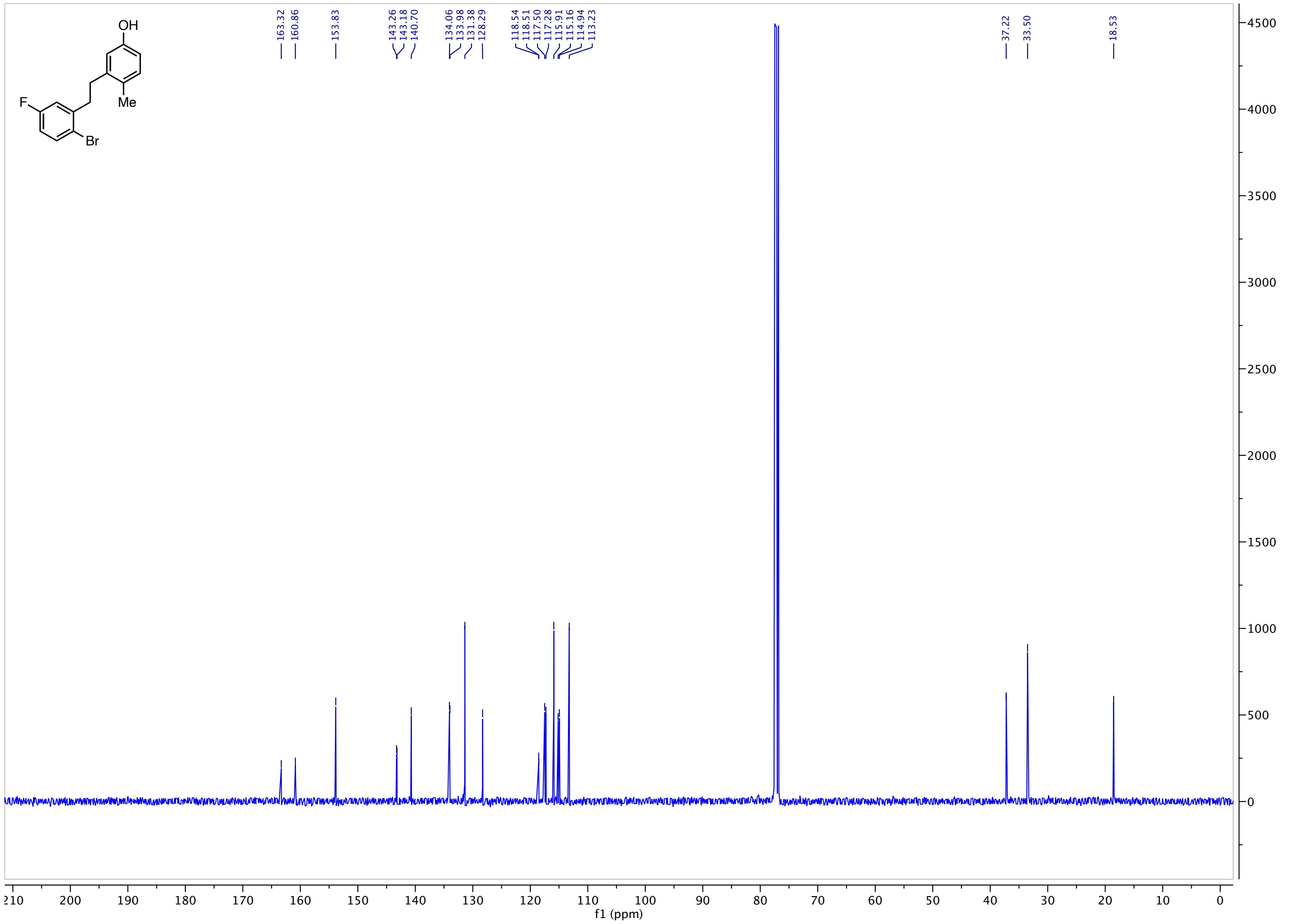

^{13}C NMR ($CDCl_3$): 3-(2-bromo-5-fluorophenethyl)-4-methylphenol **(6d)**

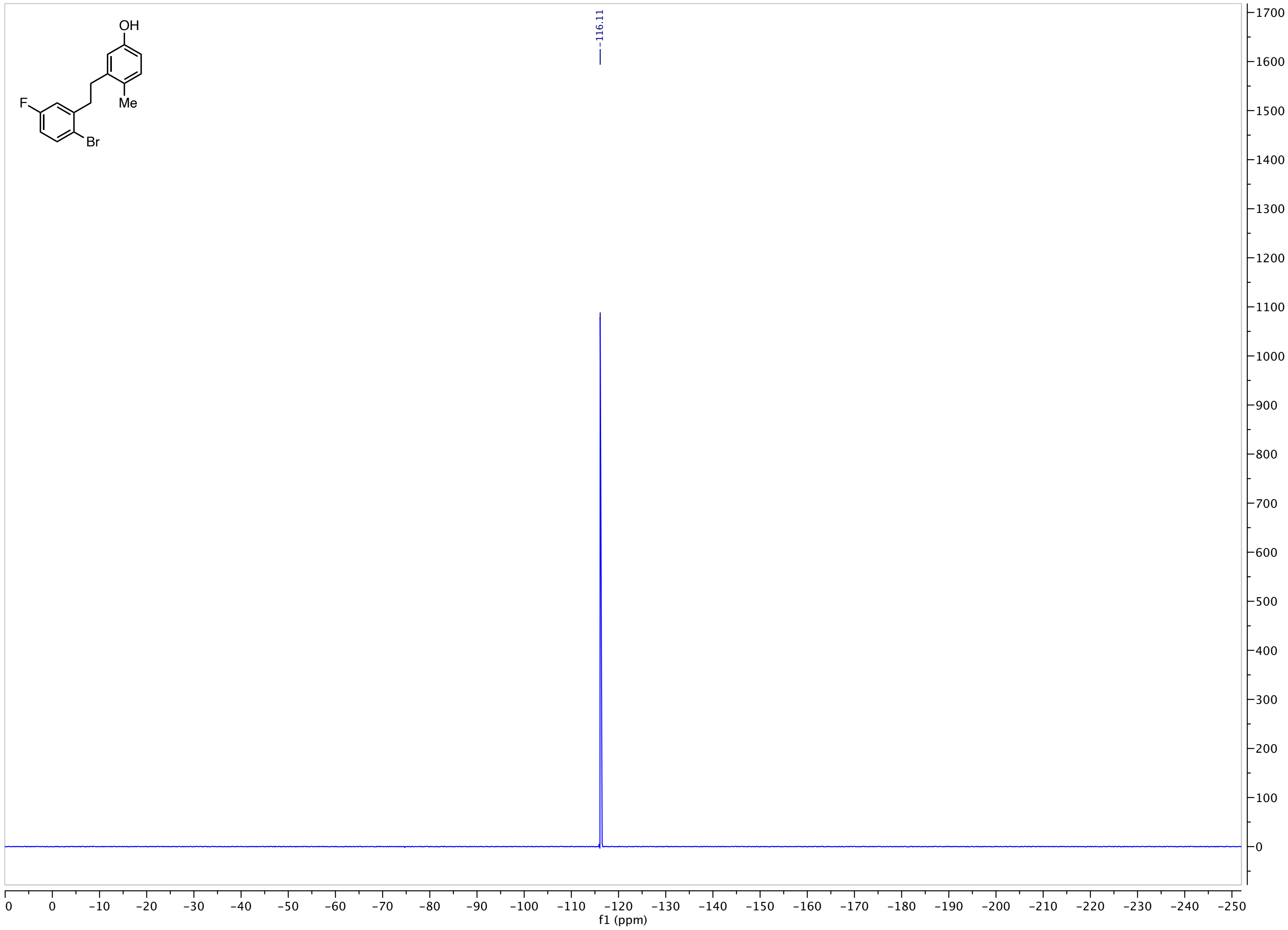

¹H NMR ($CDCl_3$): 1-Bromo-4,5-dimethoxy-2-(5-methoxy-2-methylphenethyl)benzene **(S34)**

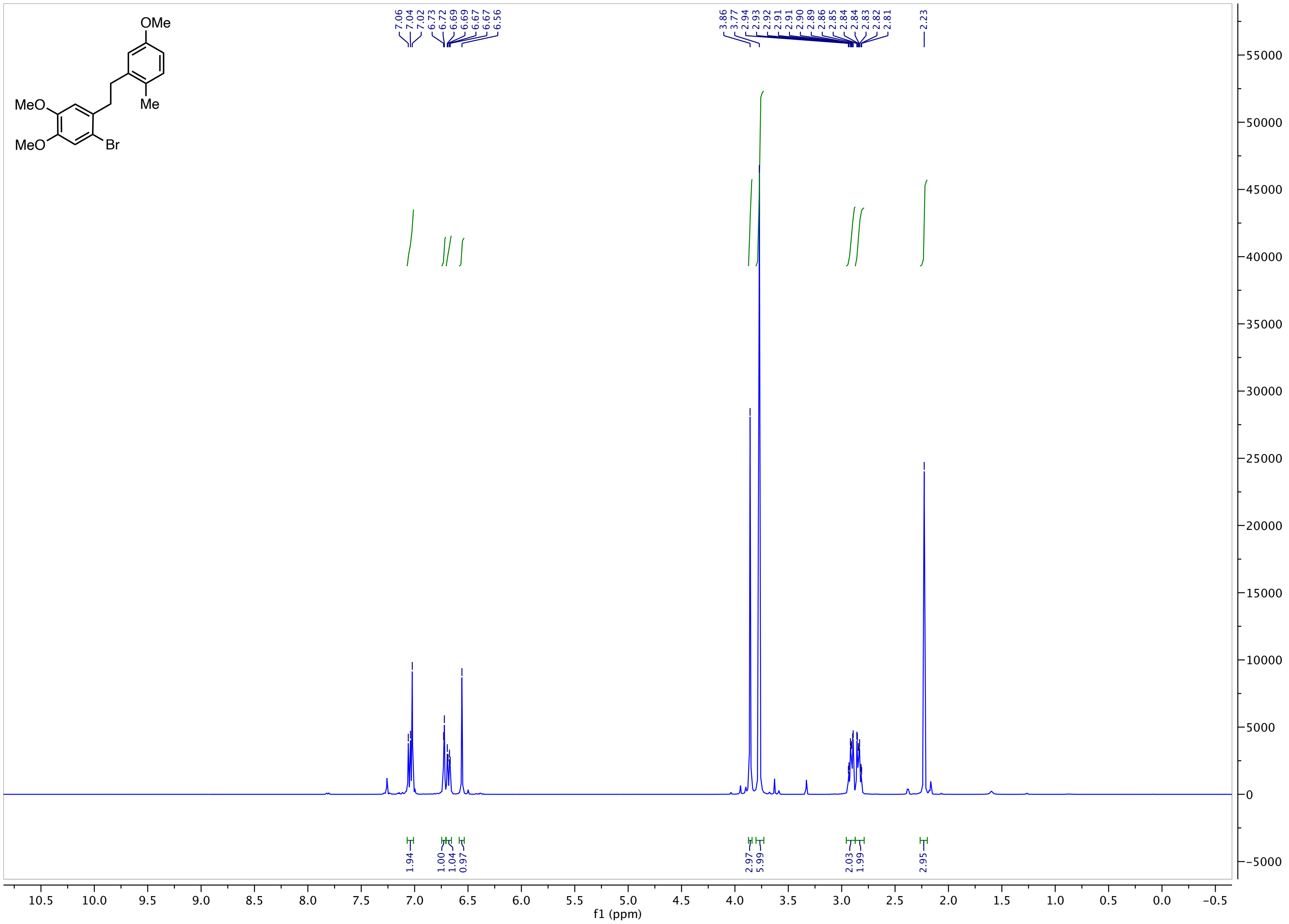

^{13}C NMR (CDCl$_3$): 1-Bromo-4,5-dimethoxy-2-(5-methoxy-2-methylphenethyl)benzene **(S34)**

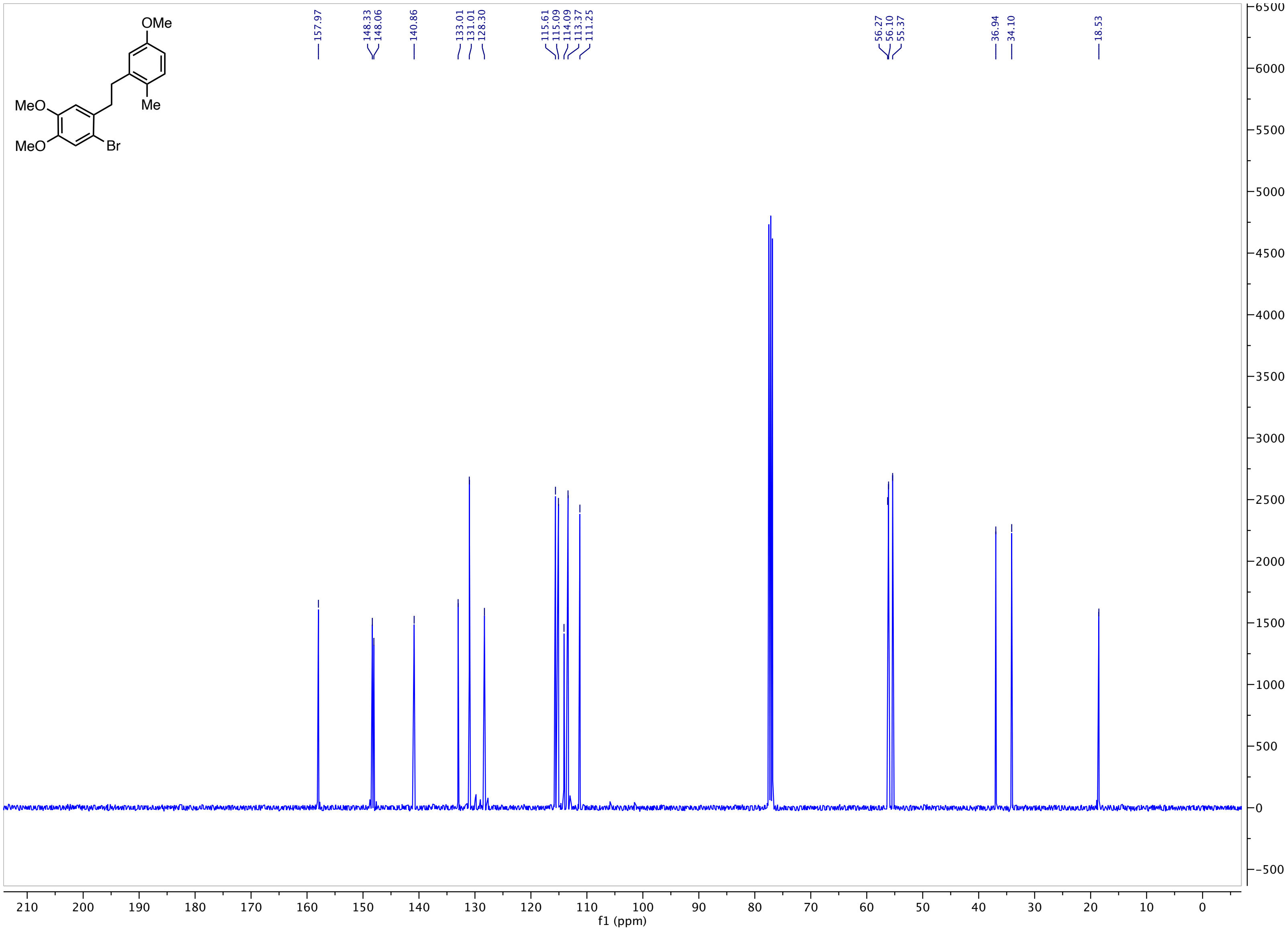

^{1}H NMR ($CDCl_3$): 4-Bromo-5-(5-hydroxy-2-methylphenethyl)benzene-1,2-diol **(S35)**

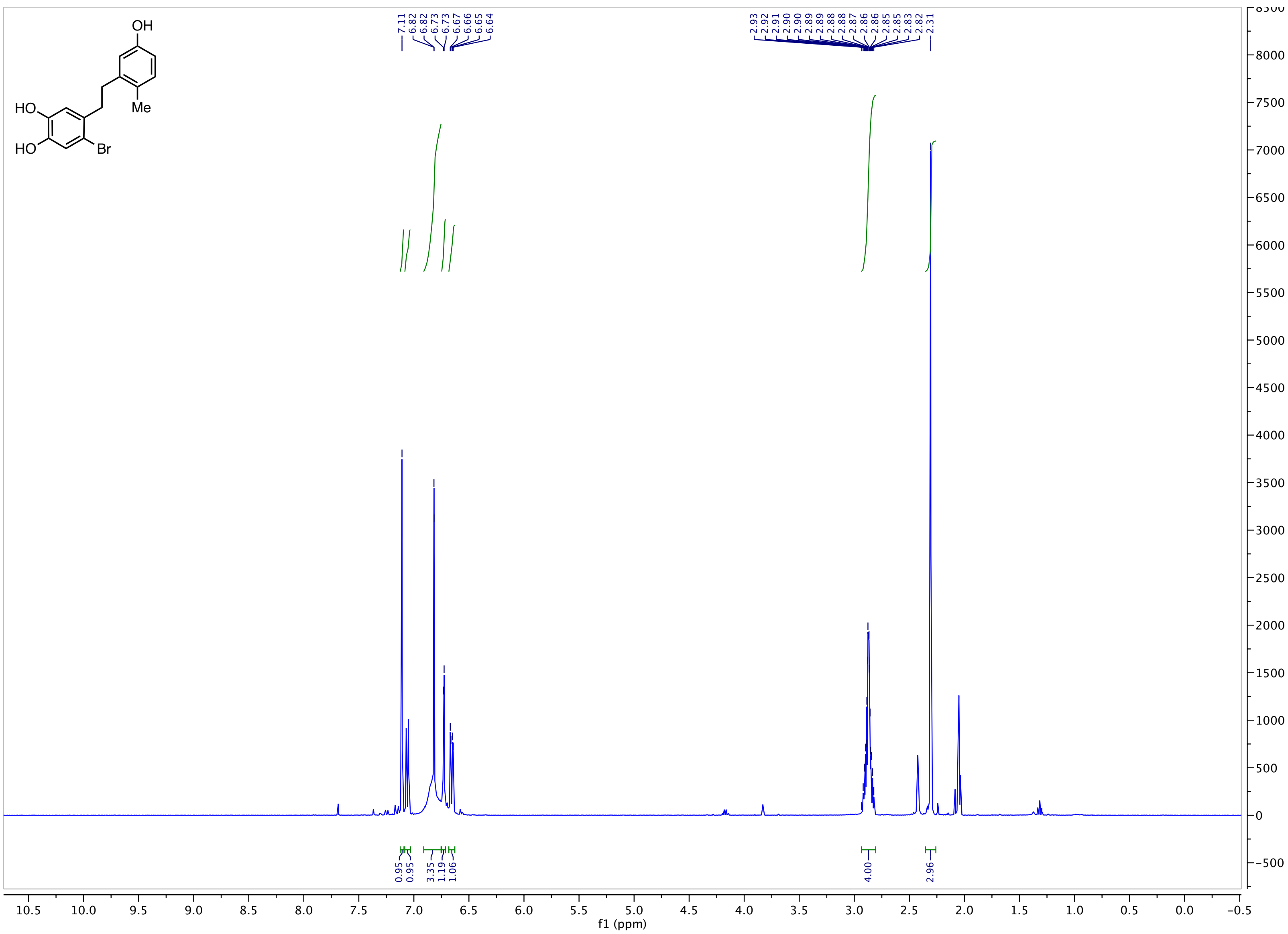

^{13}**C NMR** ($CDCl_3$): 4-Bromo-5-(5-hydroxy-2-methylphenethyl)benzene-1,2-diol **(S35)**

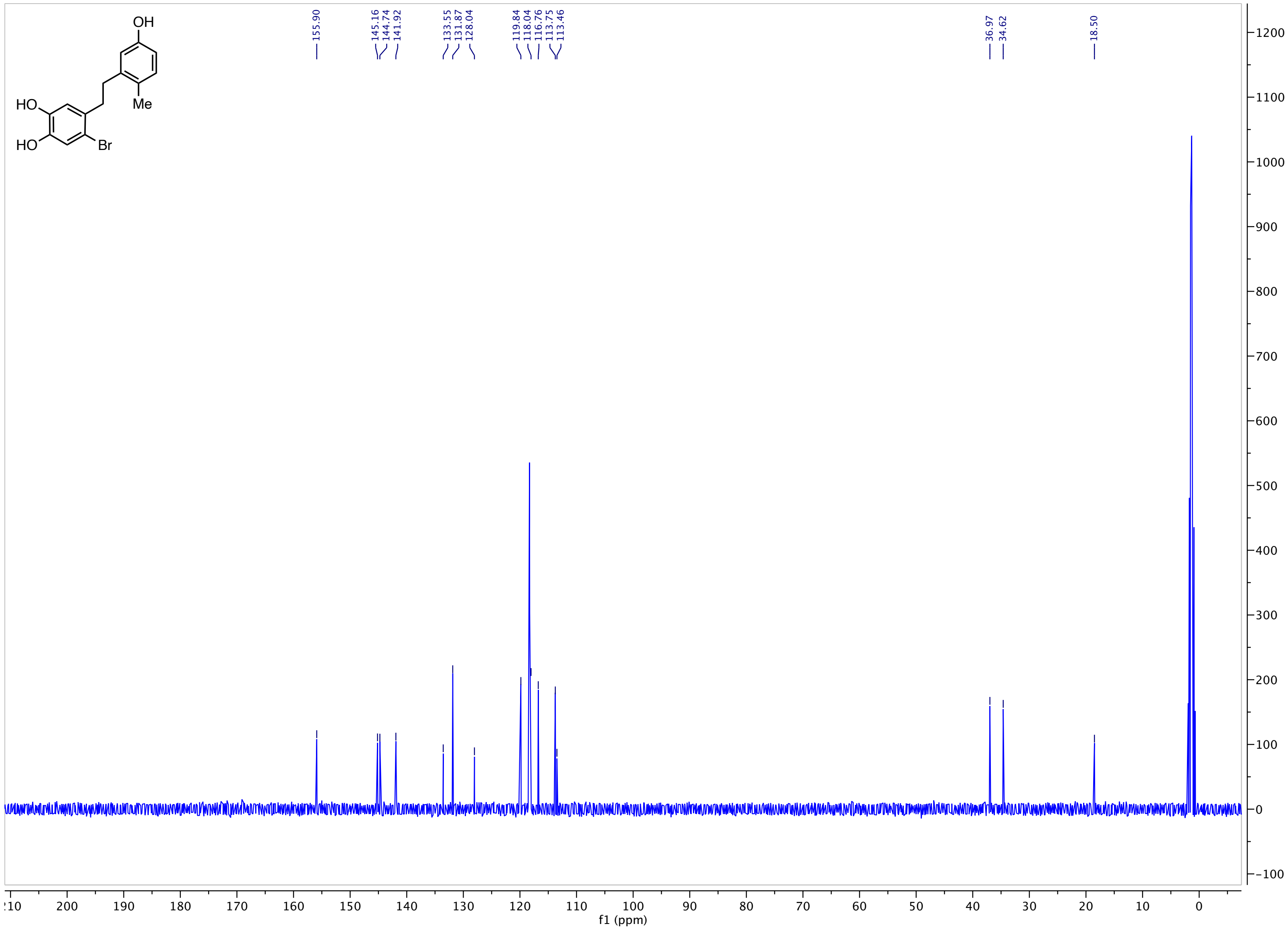

^{1}H NMR ($CDCl_3$): 3-(2-(6-bromo-2,2-dimethylbenzo[*d*][1,3]dioxol-5-yl)ethyl)-4-Methylphenol **(6e)**

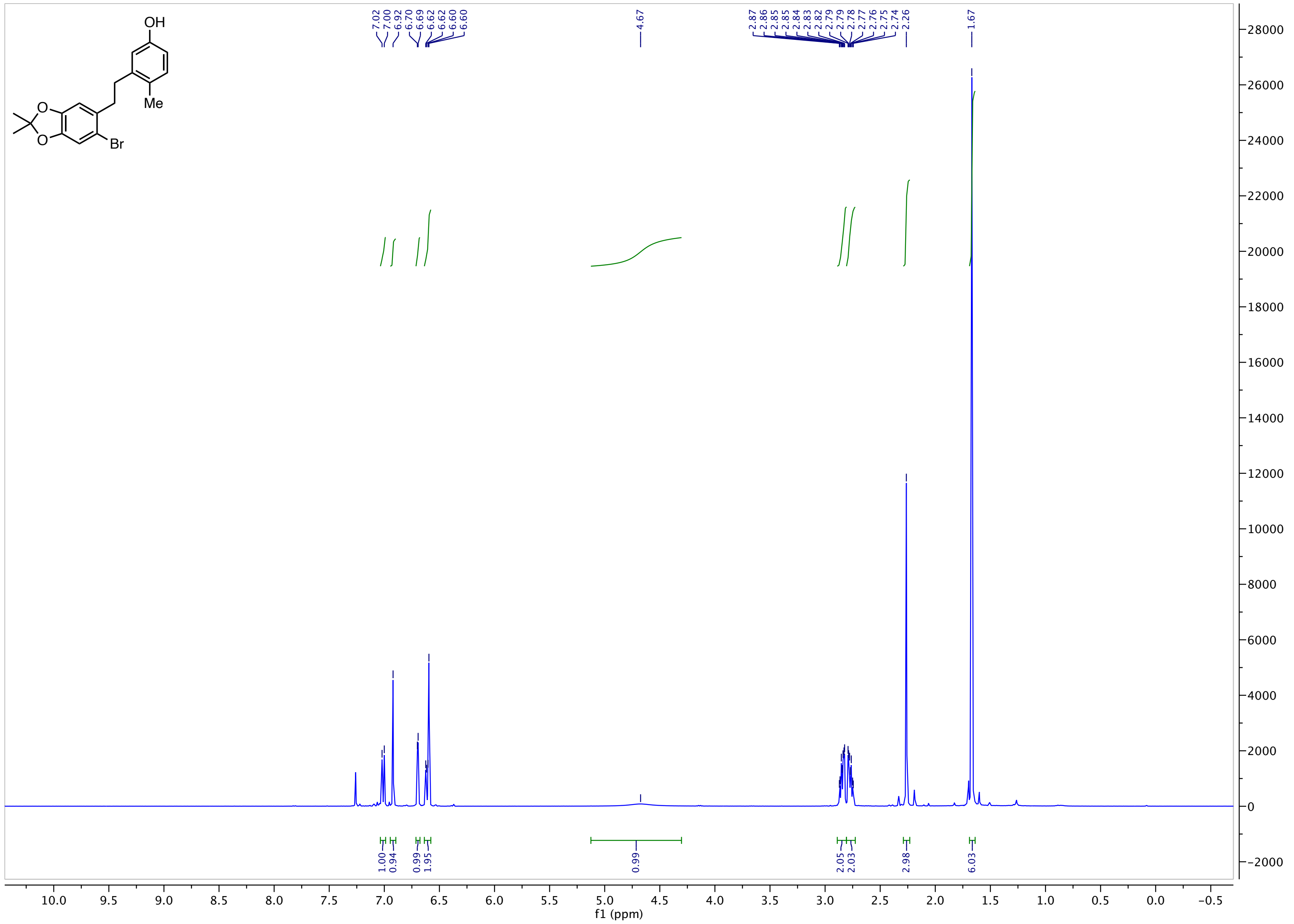

^{13}C **NMR** (CDCl$_3$): 3-(2-(6-bromo-2,2-dimethylbenzo[*d*][1,3]dioxol-5-yl)ethyl)-4-Methylphenol **(6e)**

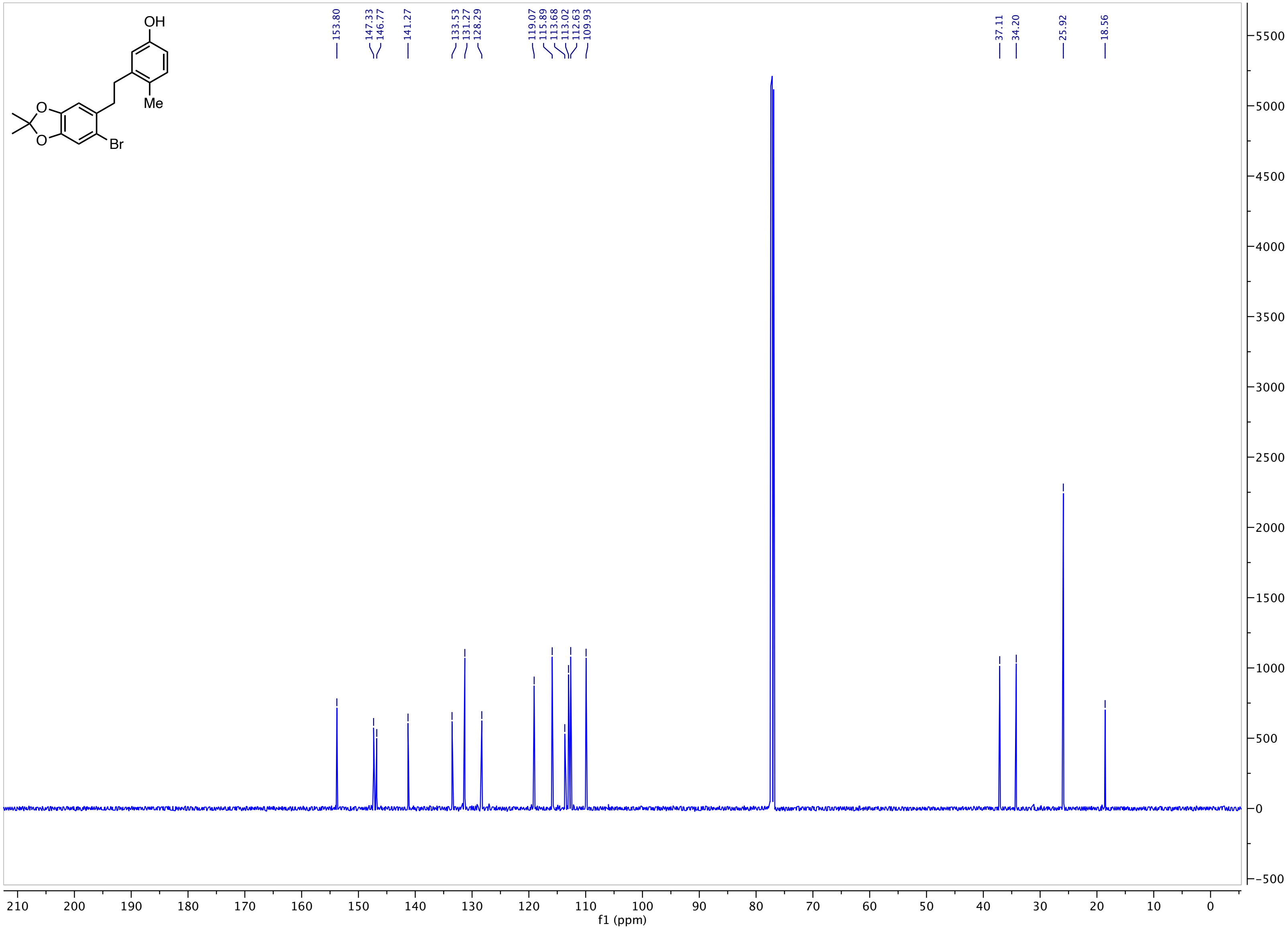

^{1}H NMR (CDCl$_3$): 4-((2-bromobenzyl)oxy)-3-Methylphenol **(8a)**

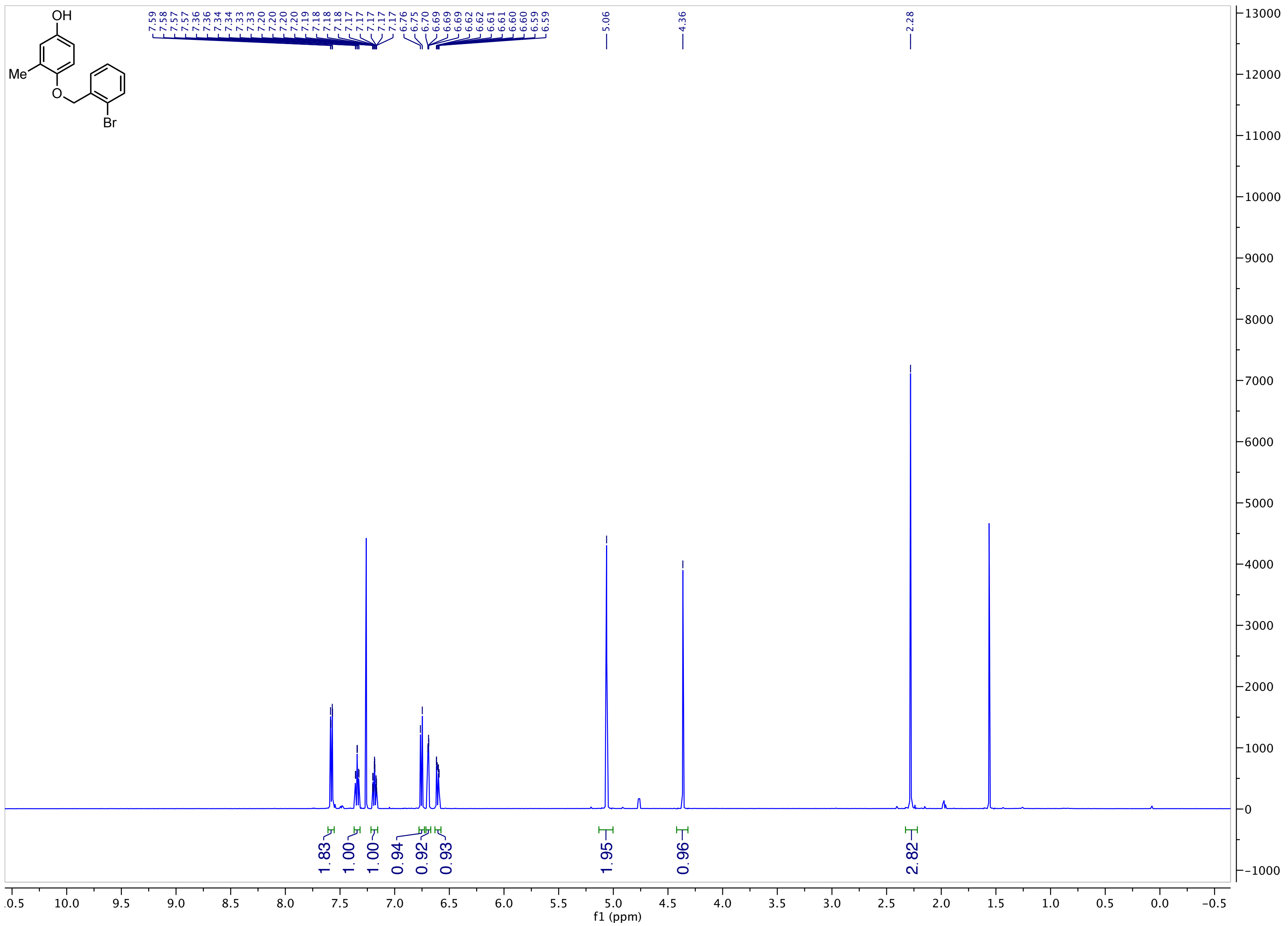

^{13}**C NMR** ($CDCl_3$): 4-((2-bromobenzyl)oxy)-3-Methylphenol **(8a)**

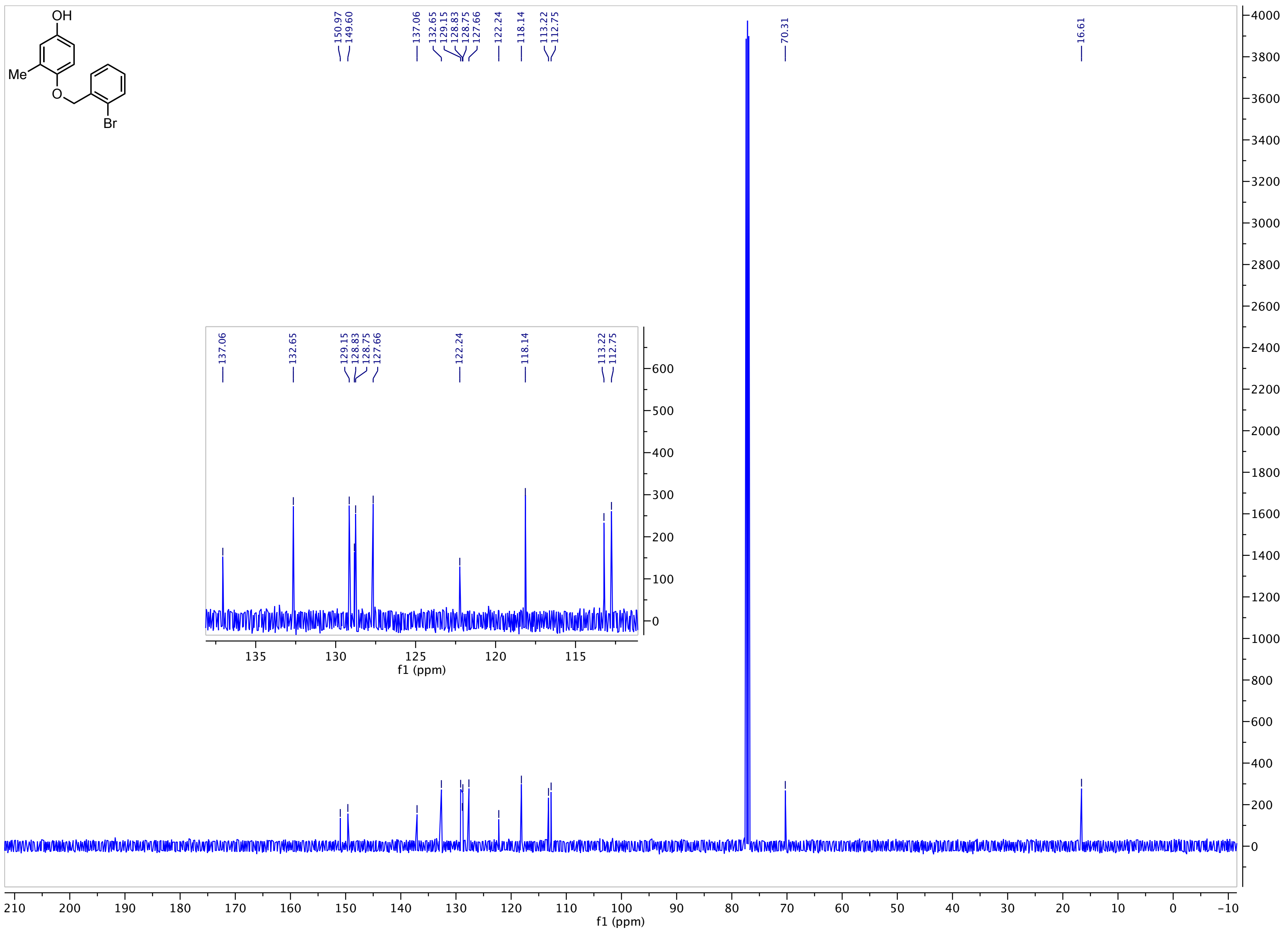

^{1}H NMR ($CDCl_3$): 4-((2-bromobenzyl)oxy)-3-Methoxyphenol (8b)

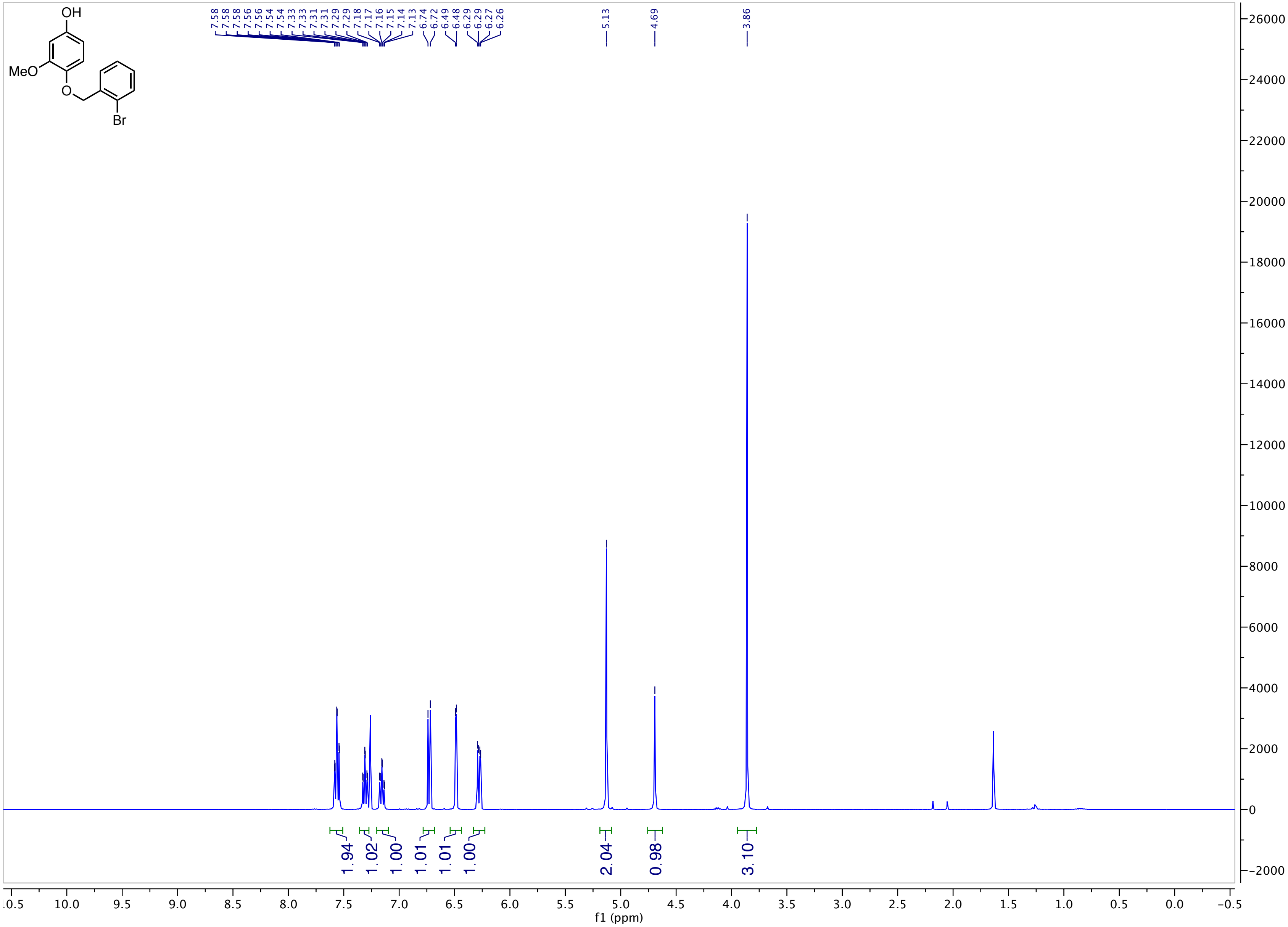

^{13}C NMR ($CDCl_3$): 4-((2-bromobenzyl)oxy)-3-Methoxyphenol **(8b)**

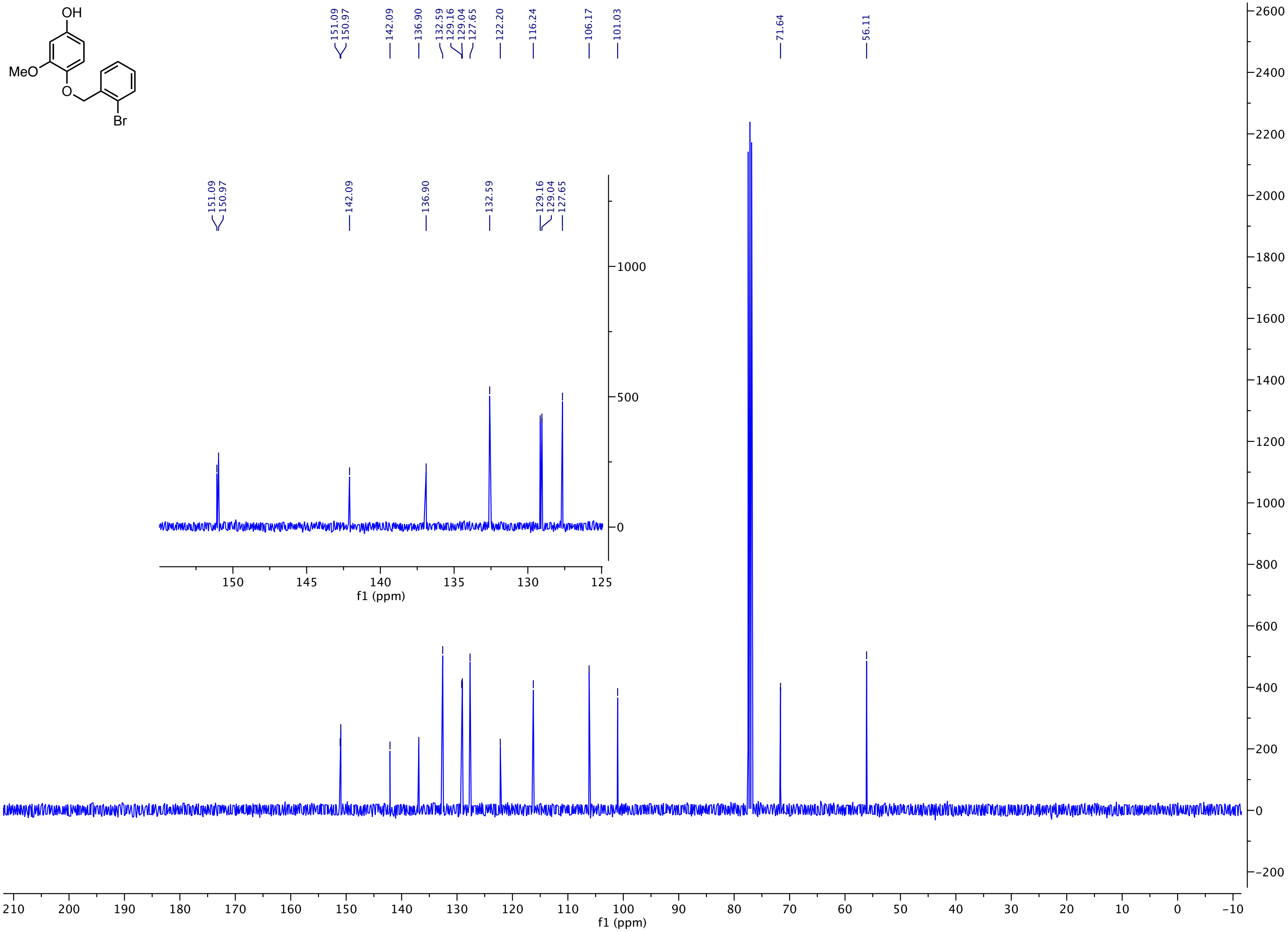

^{1}H NMR ($CDCl_3$): 4-((2-bromo-6-chlorobenzyl)oxy)-2,3-Dimethylphenol **(8c)**

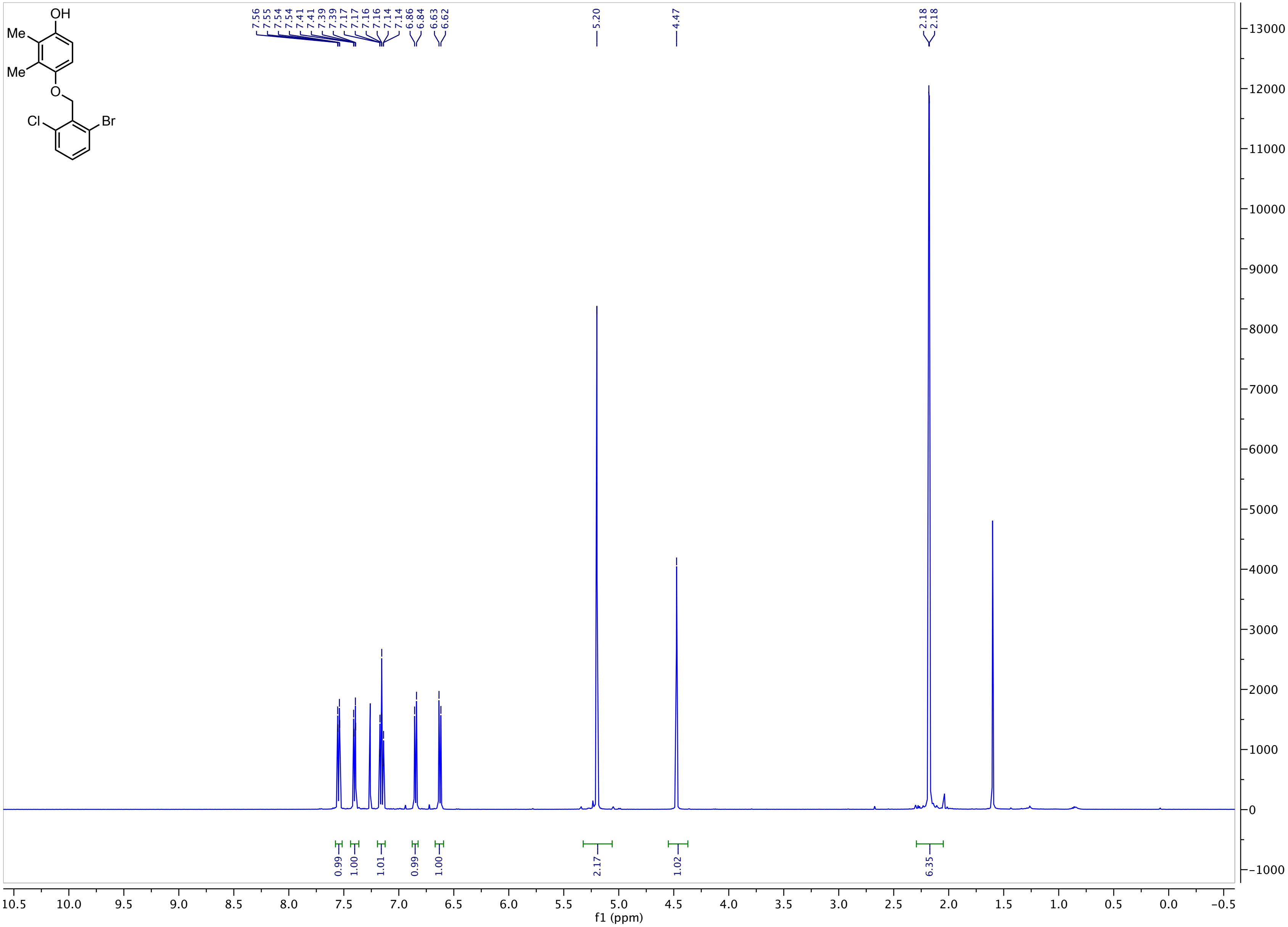

^{13}C NMR ($CDCl_3$): 4-((2-bromo-6-chlorobenzyl)oxy)-2,3-Dimethylphenol **(8c)**

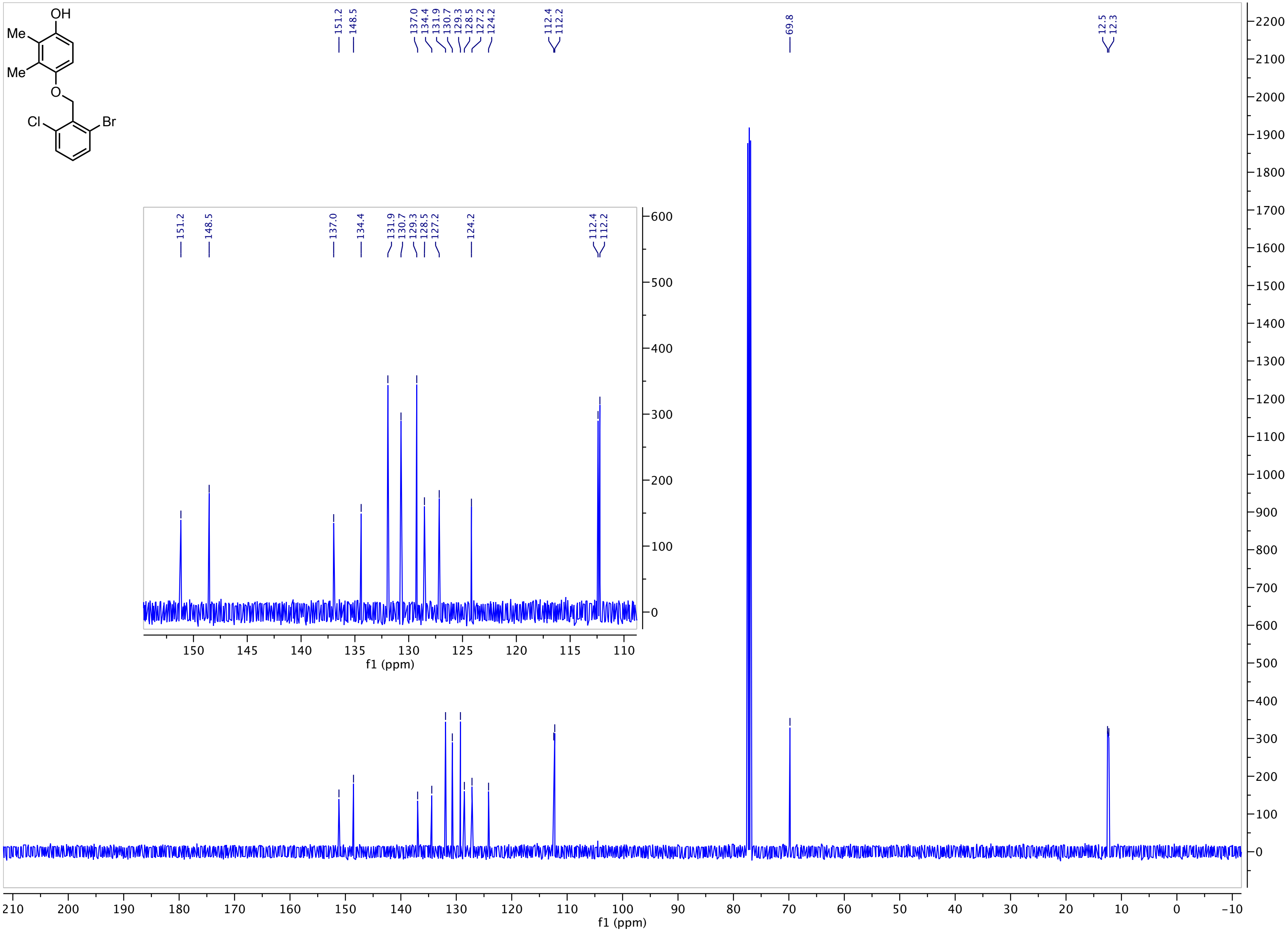

^{1}H NMR ($CDCl_3$): (4-(2-bromophenethyl)-3-methoxyphenoxy)Trimethylsilane **(S37)**

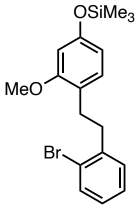

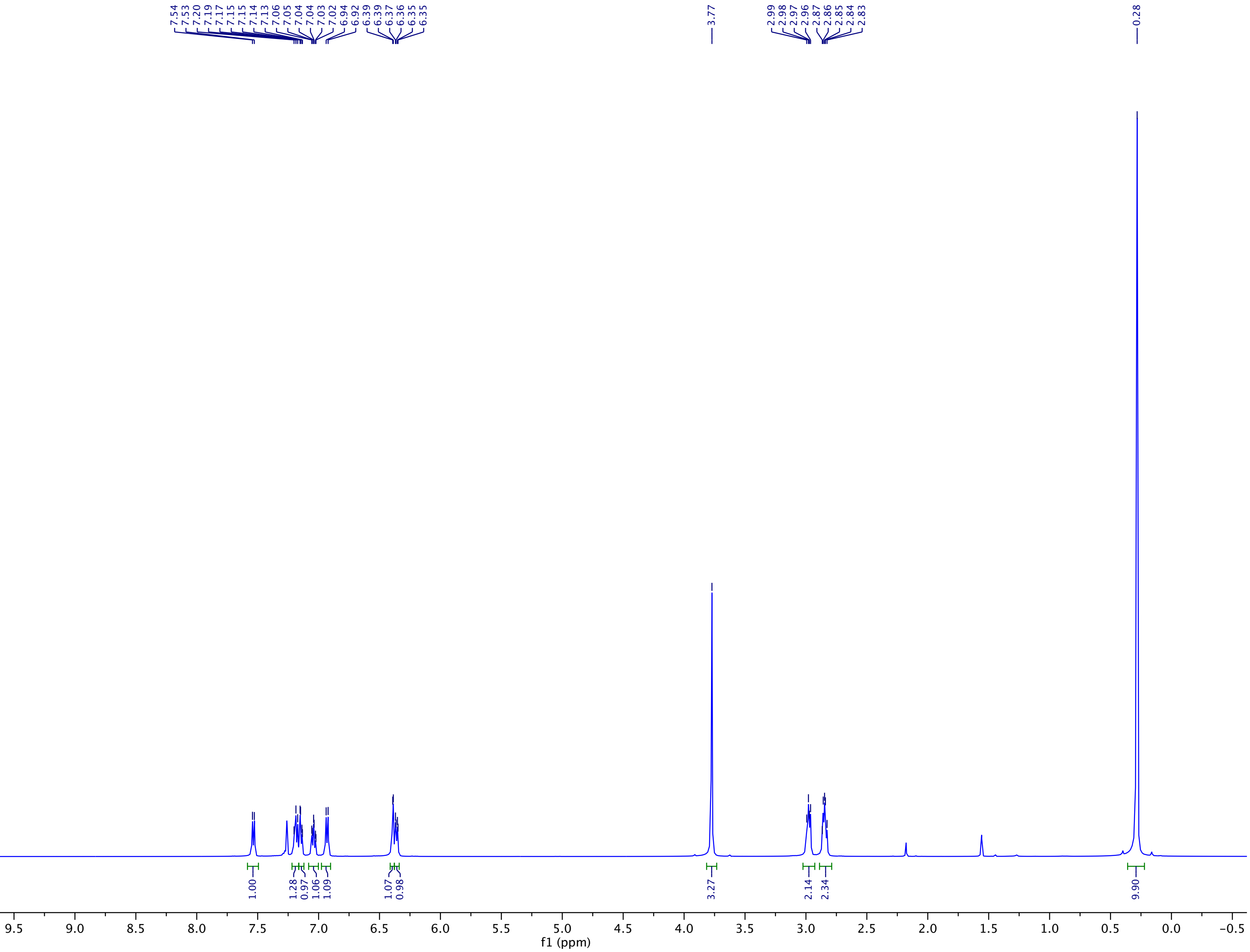

^{13}**C NMR** ($CDCl_3$): (4-(2-bromophenethyl)-3-methoxyphenoxy)Trimethylsilane **(S37)**

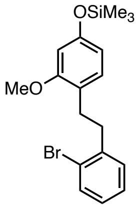

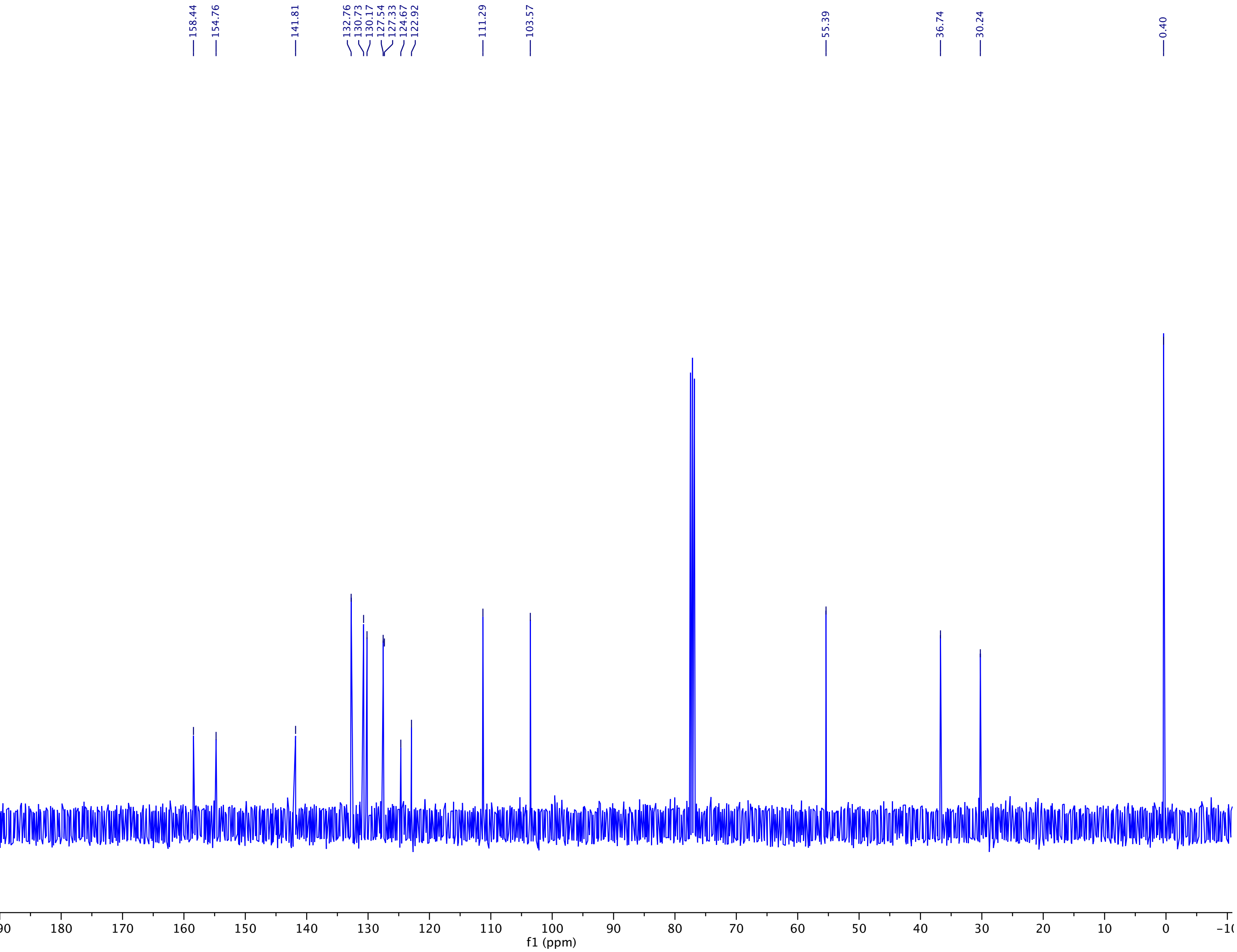

^{1}H NMR (MeOD): Potassium 4-(2-bromophenethyl)-3-methoxyphenolate (**S38**)

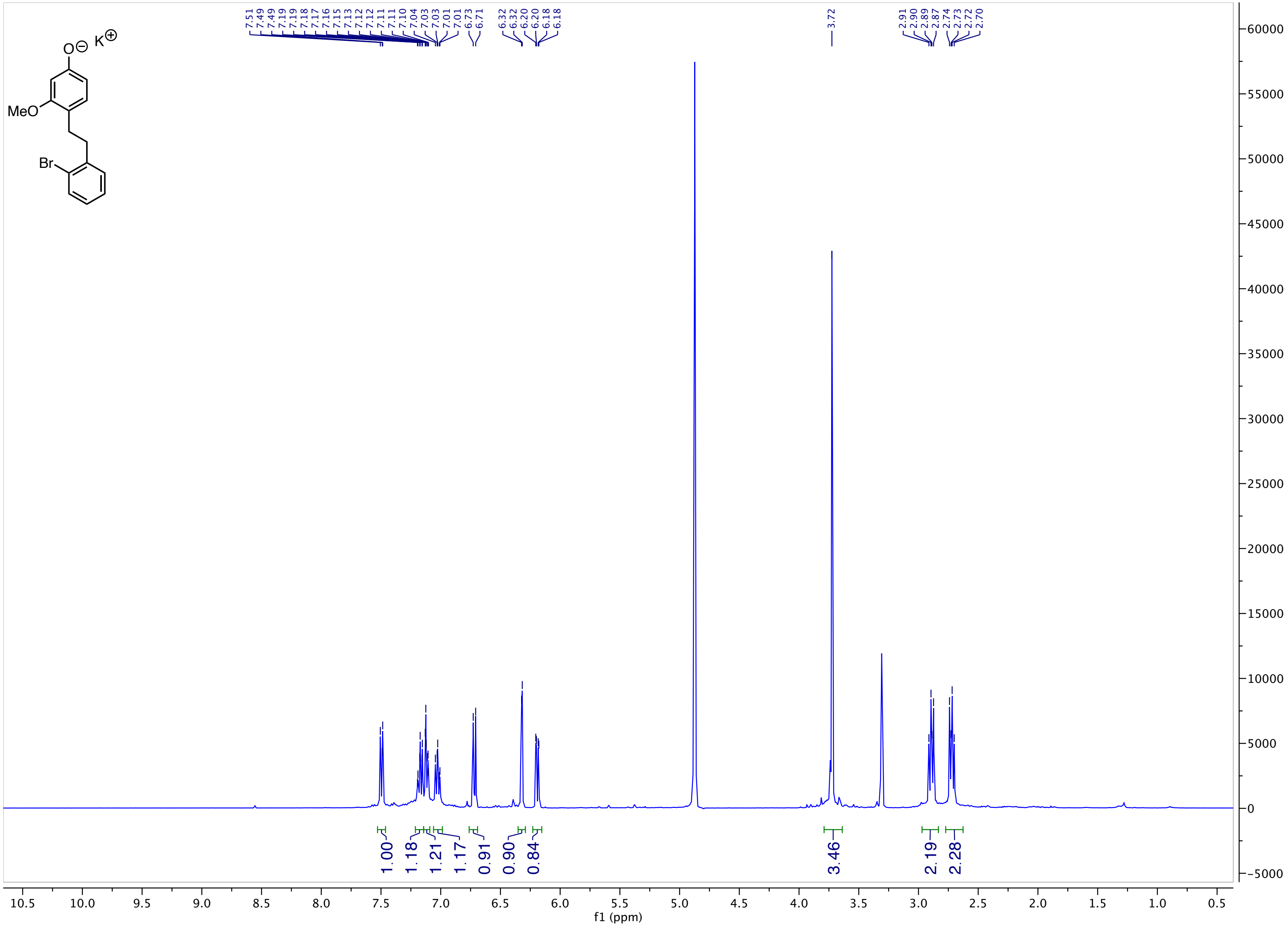

^{13}C NMR (MeOD): Potassium 4-(2-bromophenethyl)-3-methoxyphenolate (**S38**)

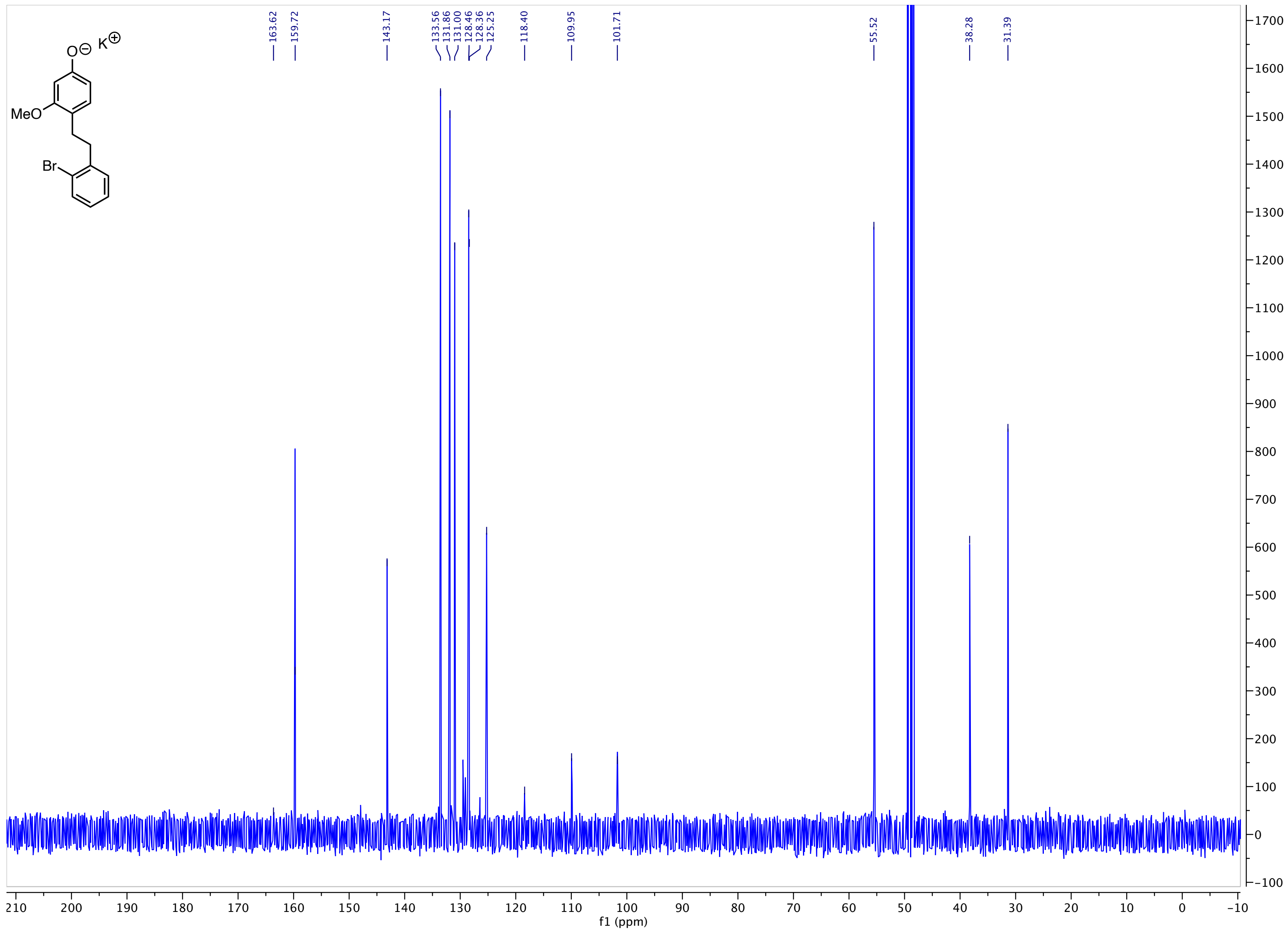

^{1}H NMR ($CDCl_3$): (*R*)-2-Methoxy-2',3'-dihydrospiro[cyclohexane-1, 1'-indene]-2,5-dien-4-one **(2a)**

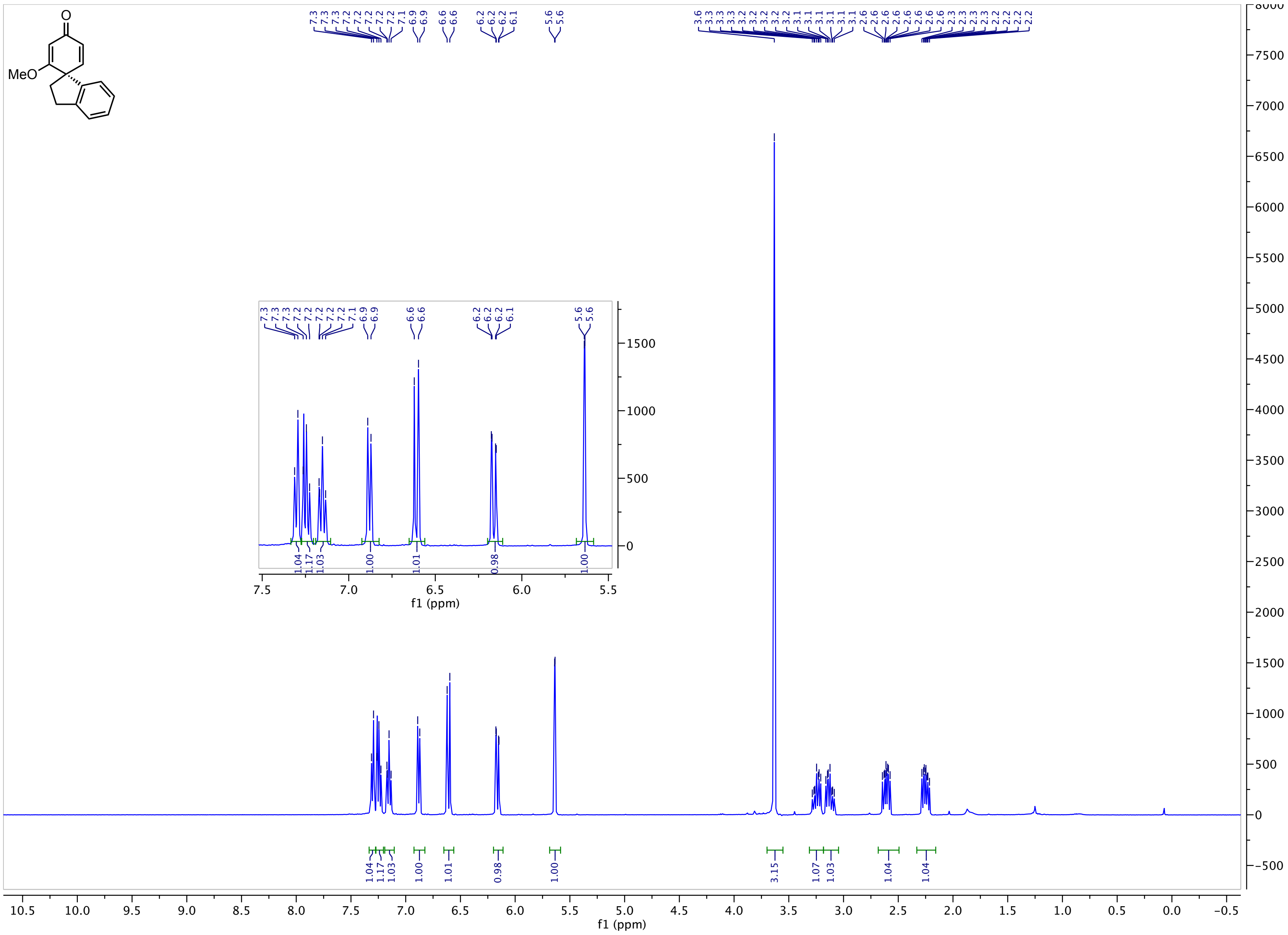

^{13}**C NMR** ($CDCl_3$): (*R*)-2-Methoxy-2',3'-dihydrospiro[cyclohexane-1, 1'-indene]-2,5-dien-4-one **(2a)**

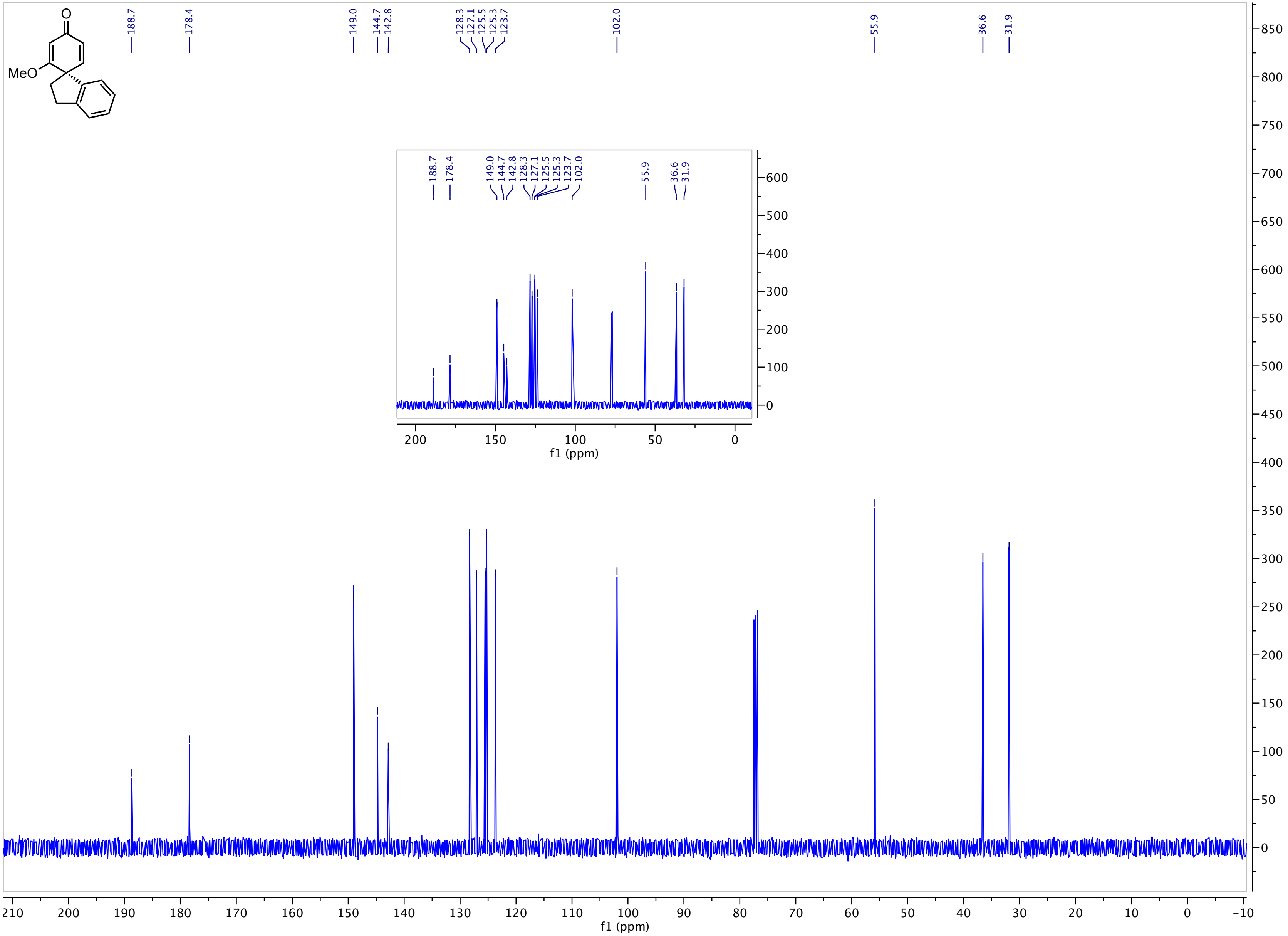

^{1}H NMR ($CDCl_3$): (*S*)-2-Methyl-2',3'-dihydrospiro[cyclohexane-1,1'-indene]-2,5-dien-4-one **(2b)**

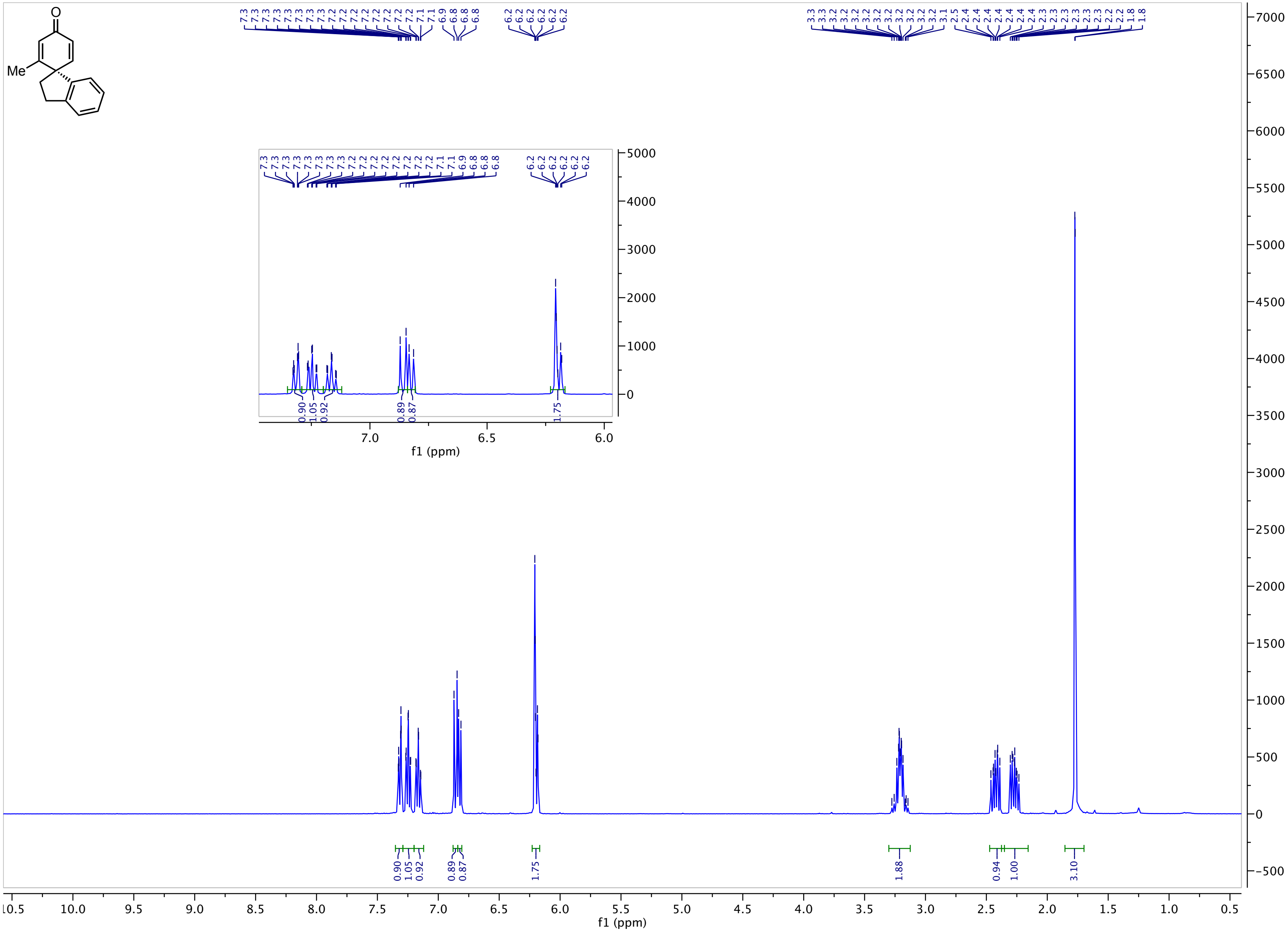

^{13}C NMR (CDCl$_3$): (*S*)-2-Methyl-2',3'-dihydrospiro[cyclohexane-1,1'-indene]-2,5-dien-4-one **(2b)**

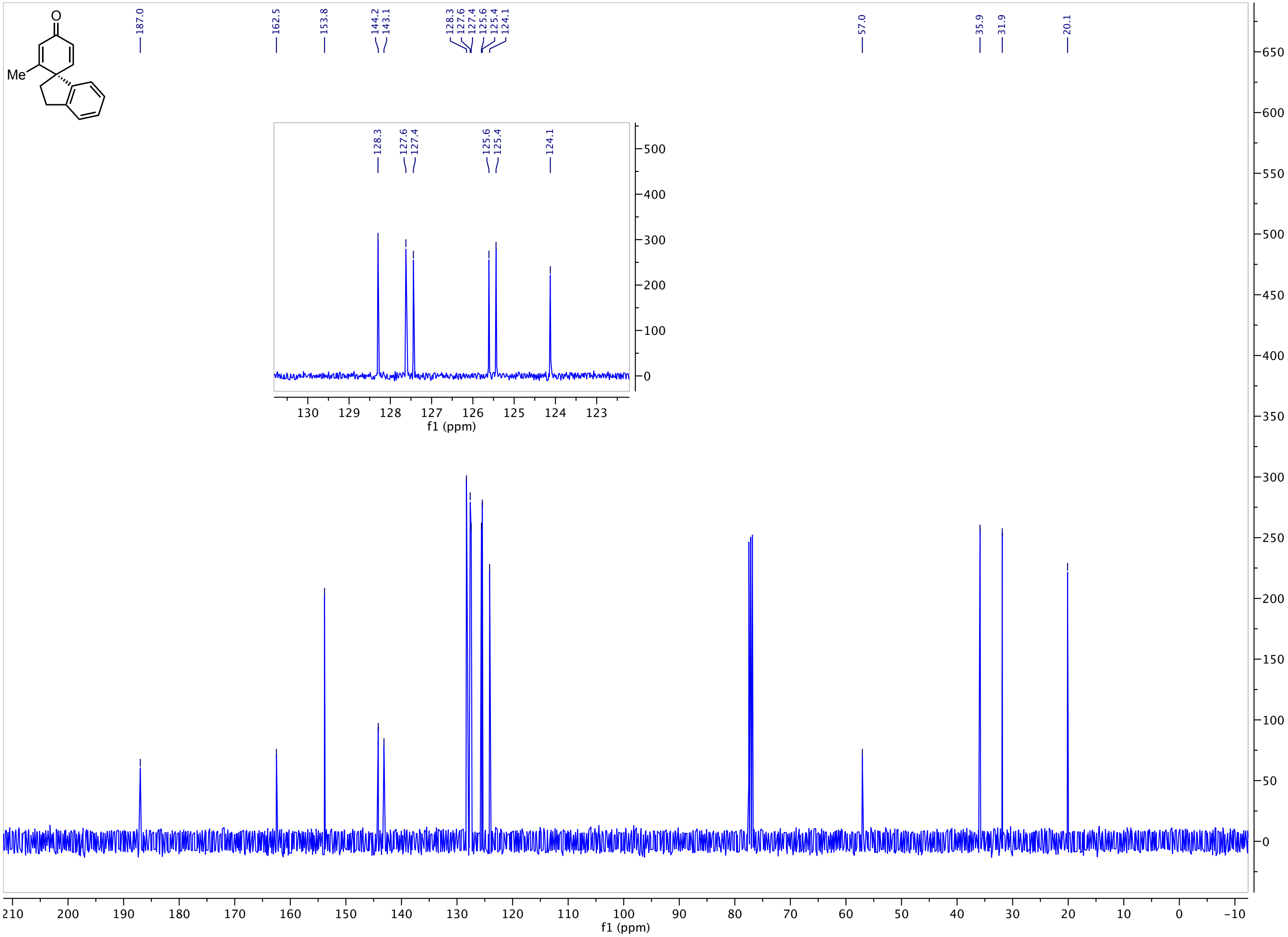

^{1}H NMR (CDCl$_3$): (*S*)-2-Phenyl-2',3'-dihydrospiro[cyclohexane-1,1'-indene]-2,5-dien-4-one **(2c)**

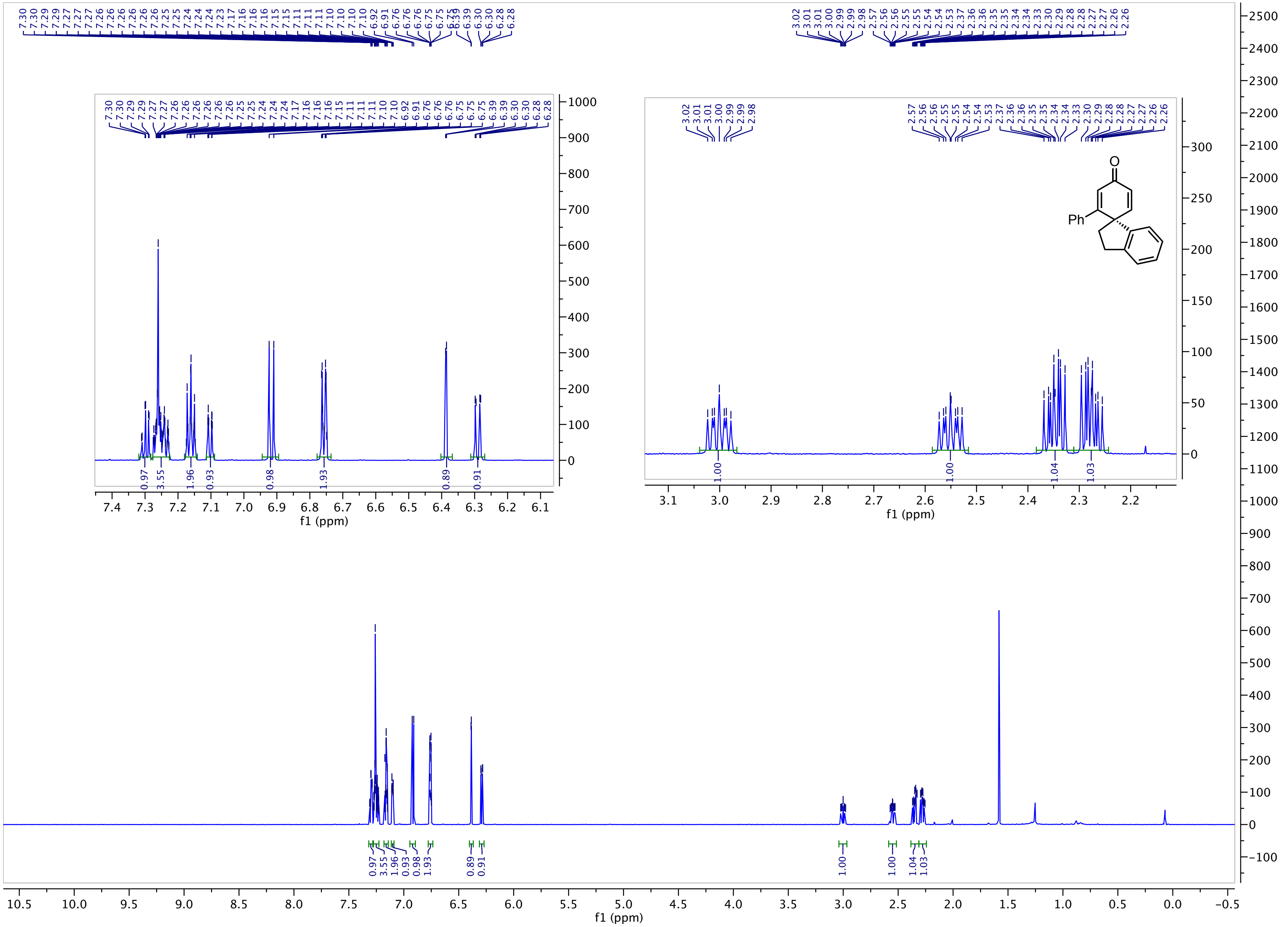

^{13}**C NMR** ($CDCl_3$): (*S*)-2-Phenyl-2',3'-dihydrospiro[cyclohexane-1,1'-indene]-2,5-dien-4-one **(2c)**

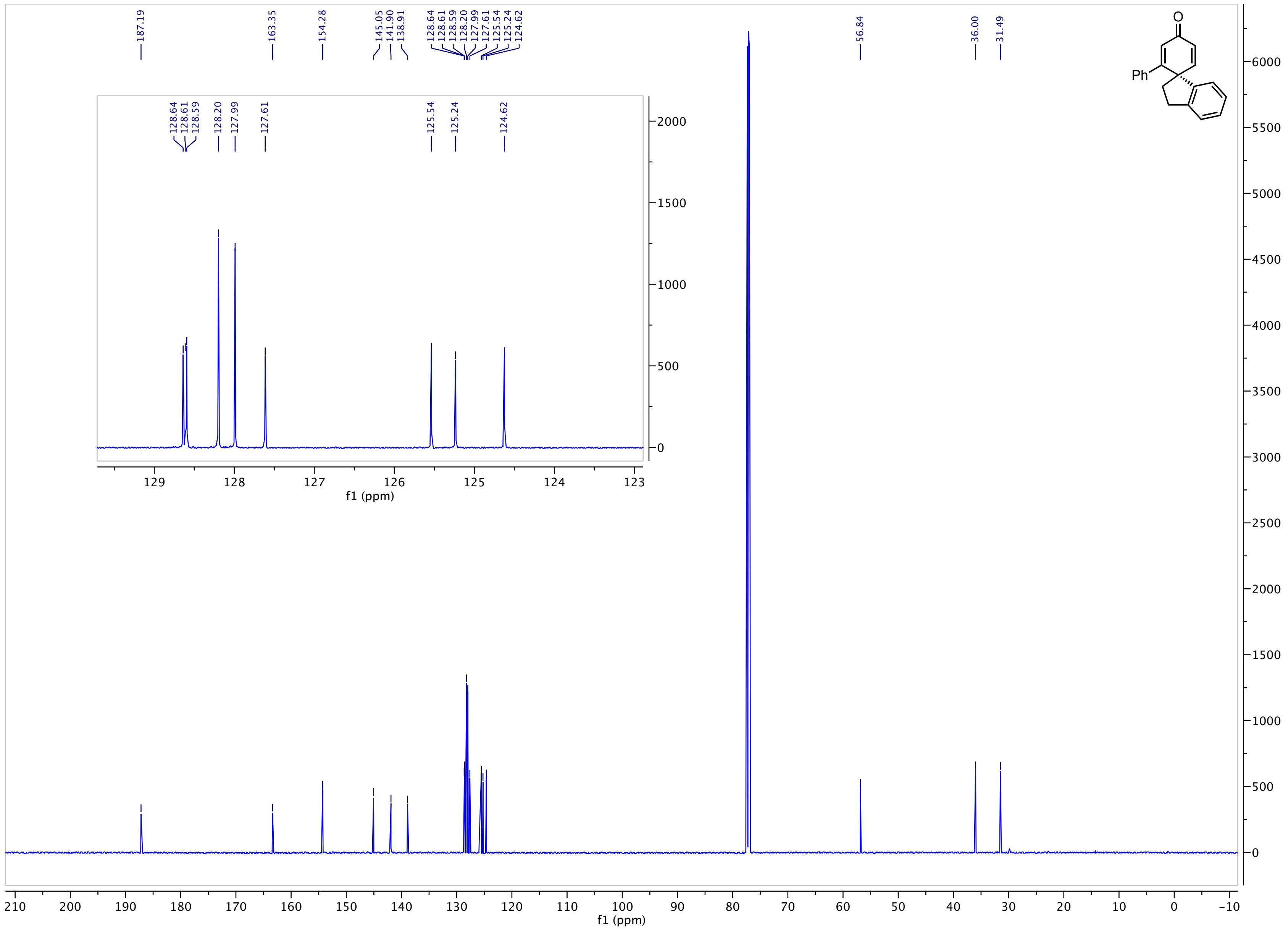

^{1}H NMR ($CDCl_3$): (*S*)-3-Phenyl-2',3'-dihydrospiro[cyclohexane-1,1'-indene]-2,5-dien-4-one (2d)

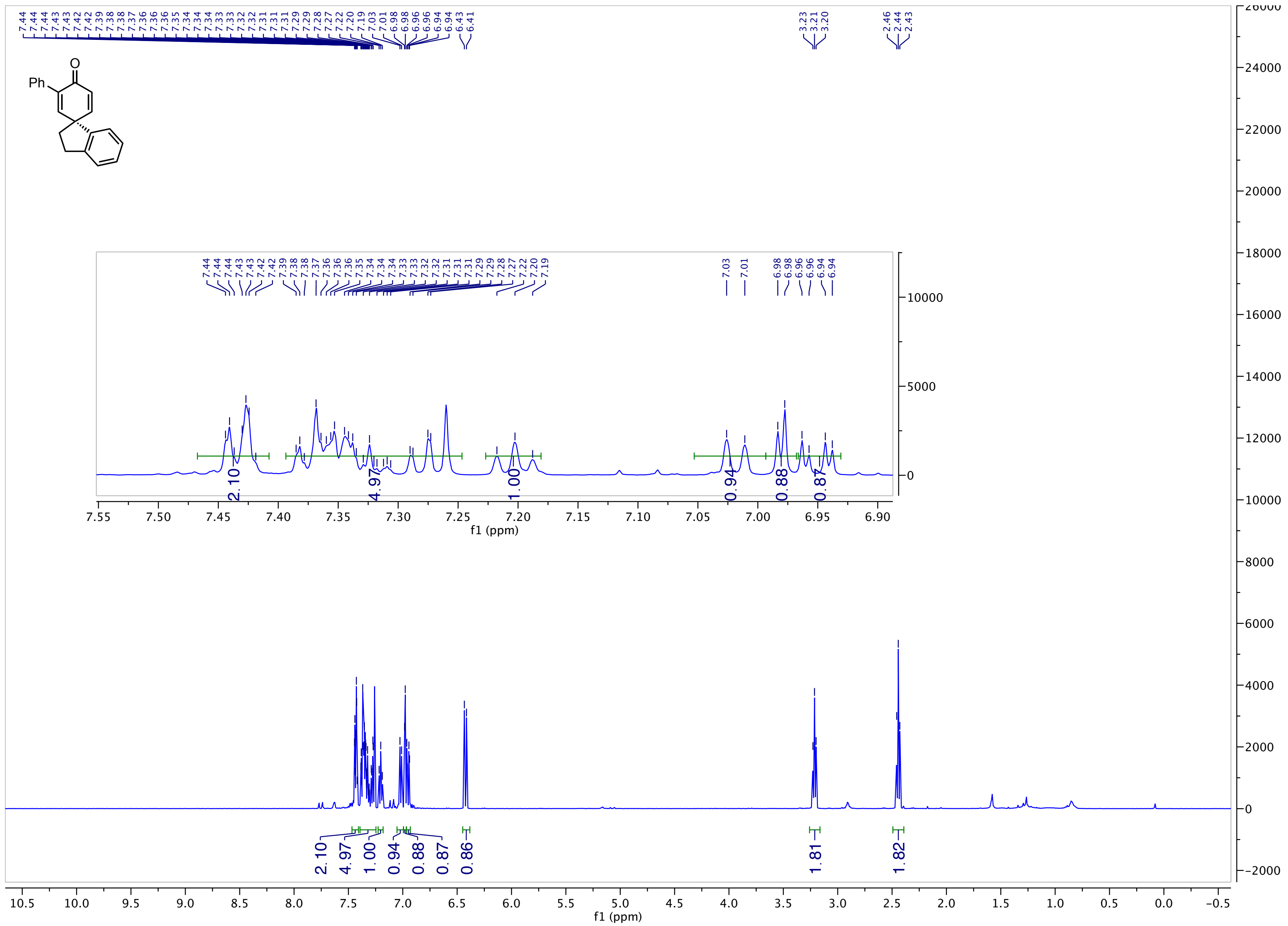

^{13}C NMR (CDCl$_3$): (*S*)-3-Phenyl-2',3'-dihydrospiro[cyclohexane-1,1'-indene]-2,5-dien-4-one **(2d)**

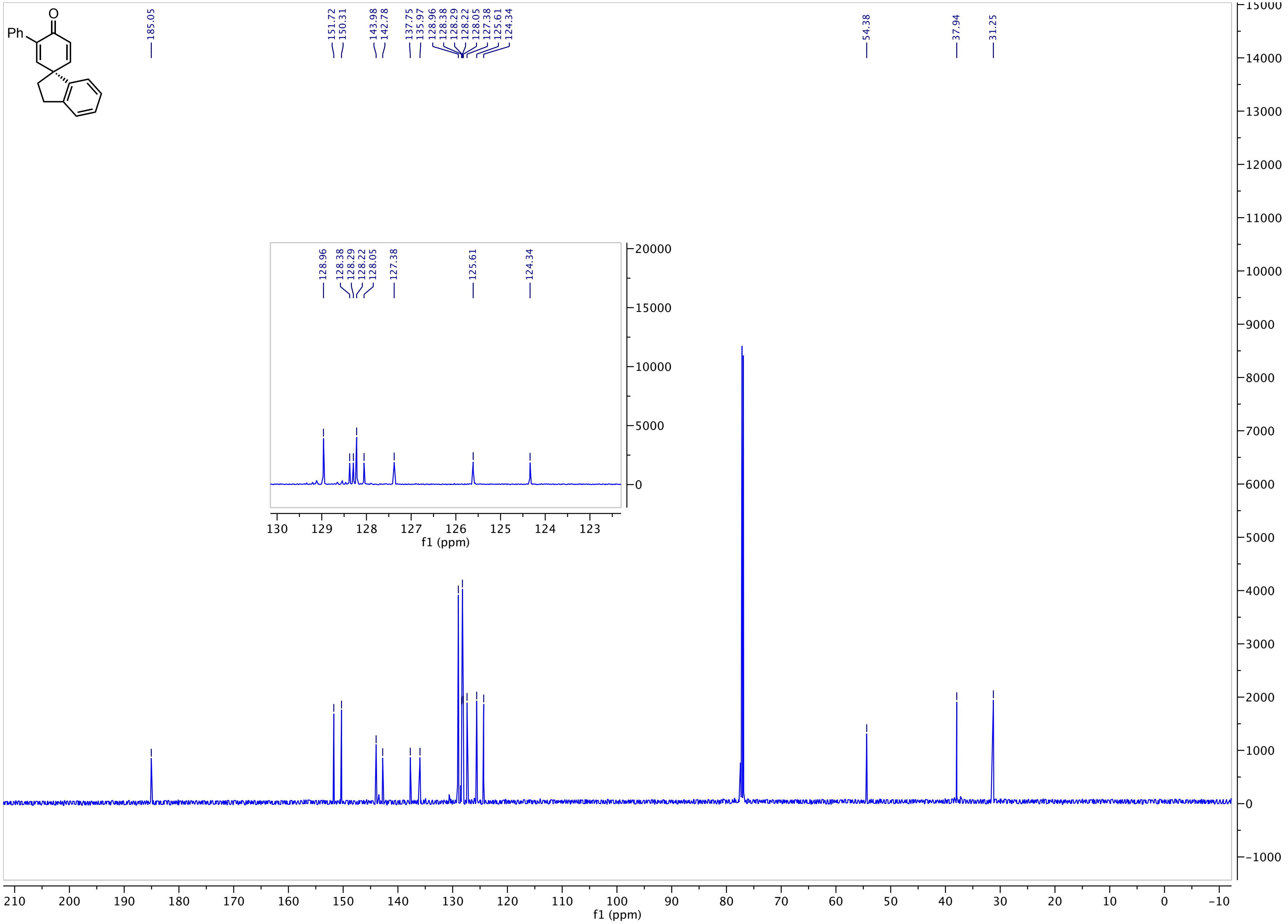

^{1}H NMR ($CDCl_3$): (*S*)-3-Methoxy-2',3'-dihydrospiro[cyclohexane-1,1'-indene]-2,5-dien-4-one **(2e)**

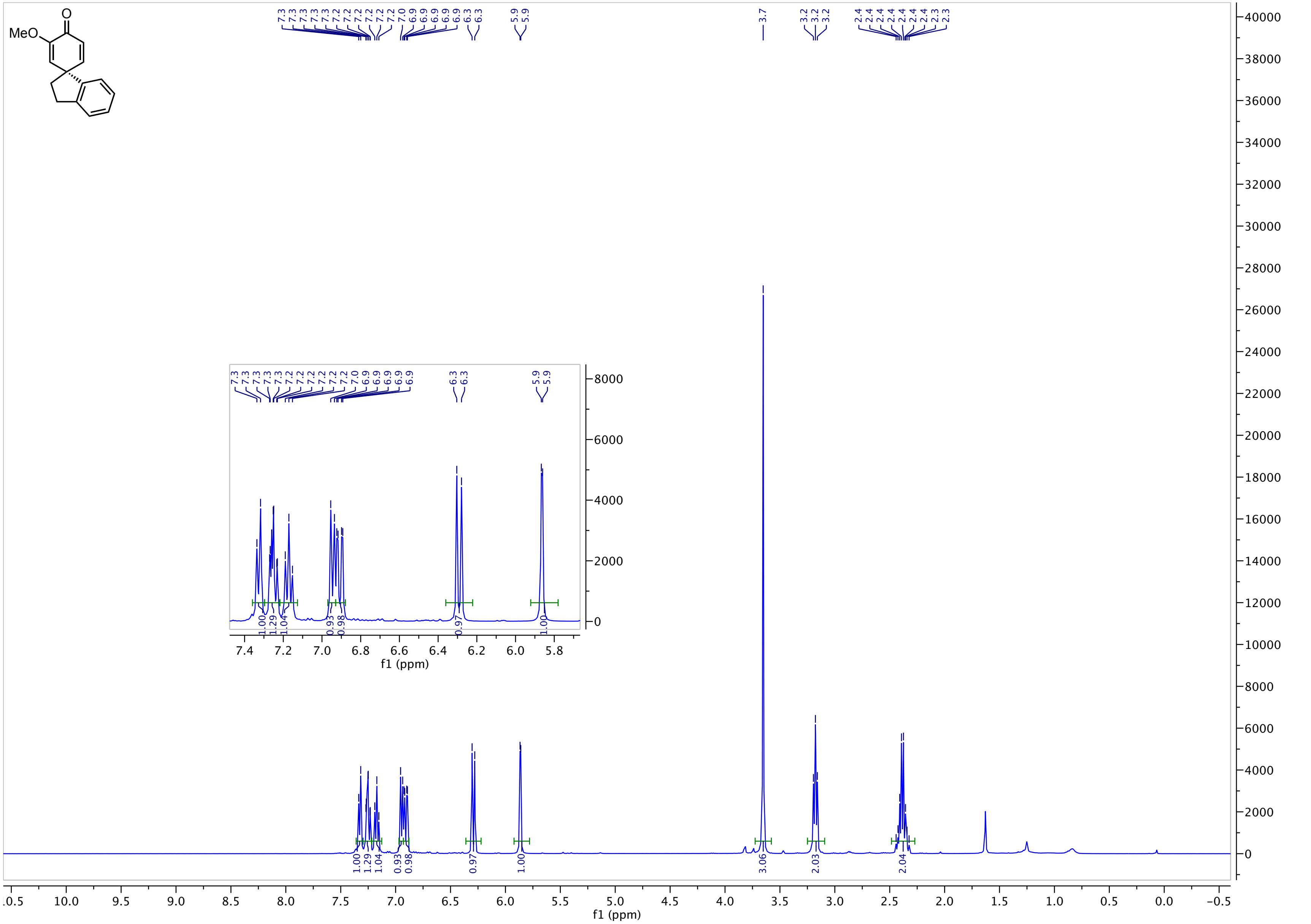

^{13}C NMR ($CDCl_3$): (*S*)-3-Methoxy-2',3'-dihydrospiro[cyclohexane-1,1'-indene]-2,5-dien-4-one (2e)

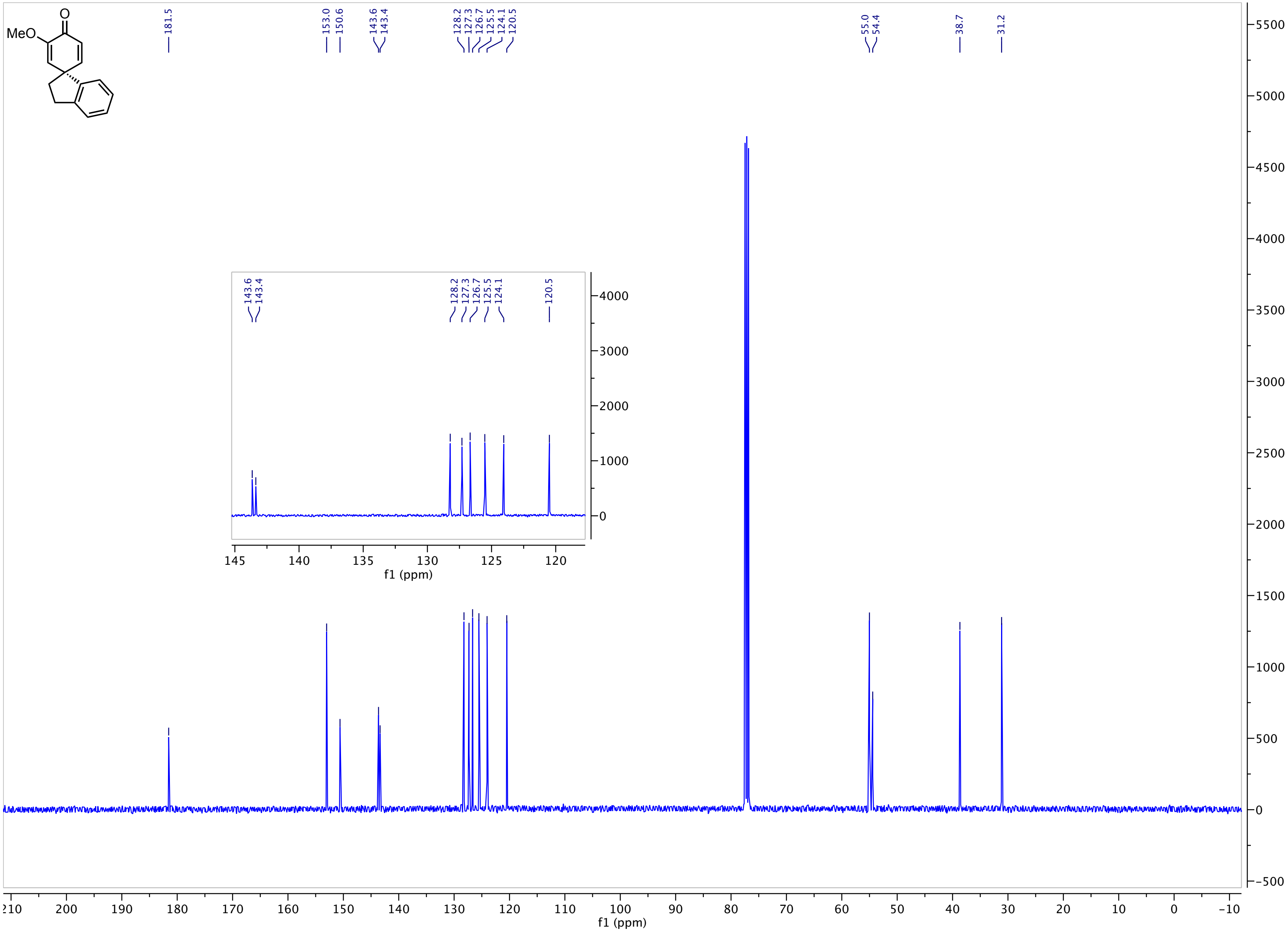

^{1}H NMR ($CDCl_3$): (*S*)-3-Methyl-2',3'-dihydrospiro[cyclohexane-1,1'-indene]-2,5-dien-4-one **(2f)**

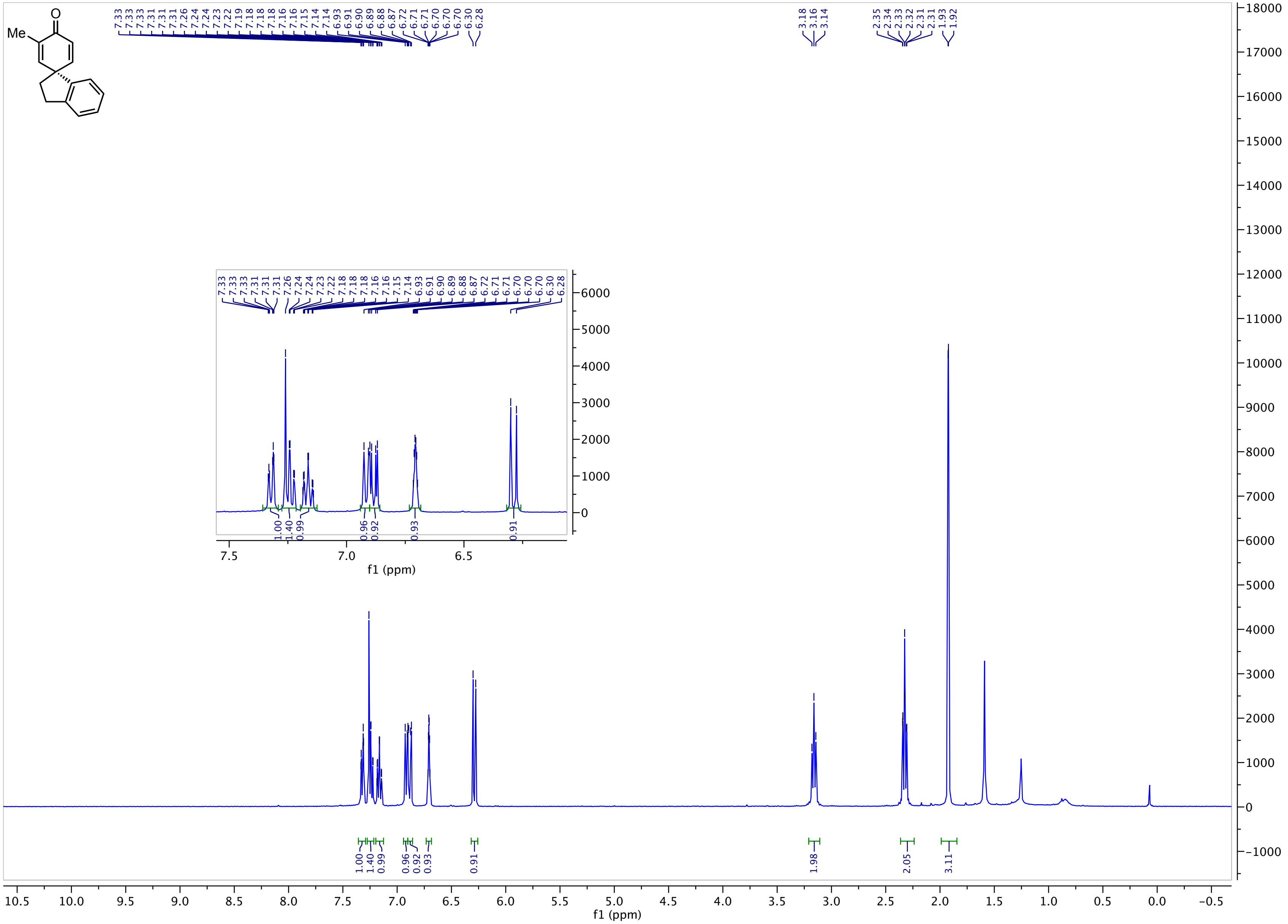

^{13}C NMR ($CDCl_3$): (*S*)-3-Methyl-2',3'-dihydrospiro[cyclohexane-1,1'-indene]-2,5-dien-4-one **(2f)**

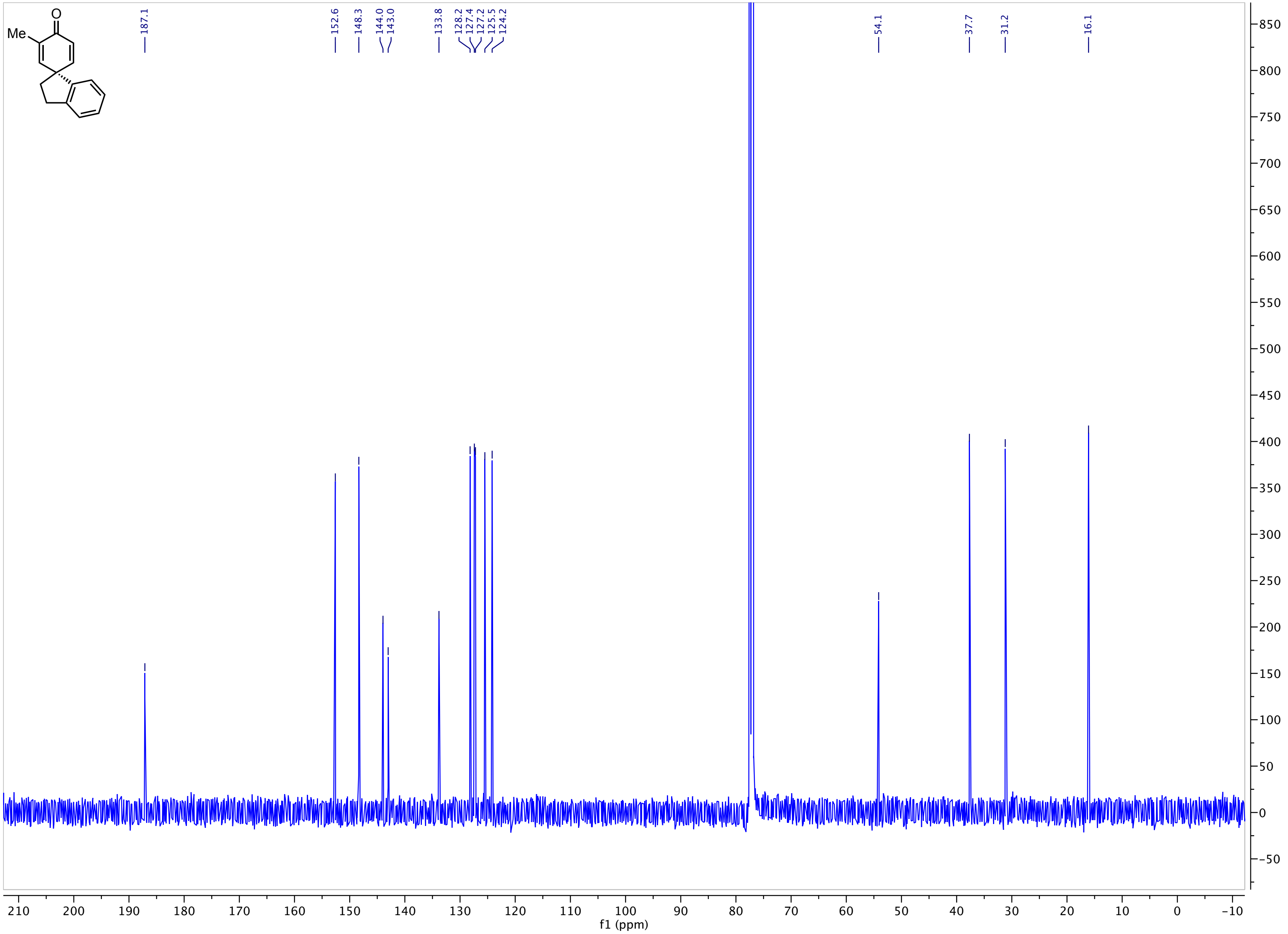

^{1}H NMR ($CDCl_3$): (*S*)-3-Fluoro-2',3'-dihydrospiro[cyclohexane-1,1'-indene]-2,5-dien-4-one **(2g)**

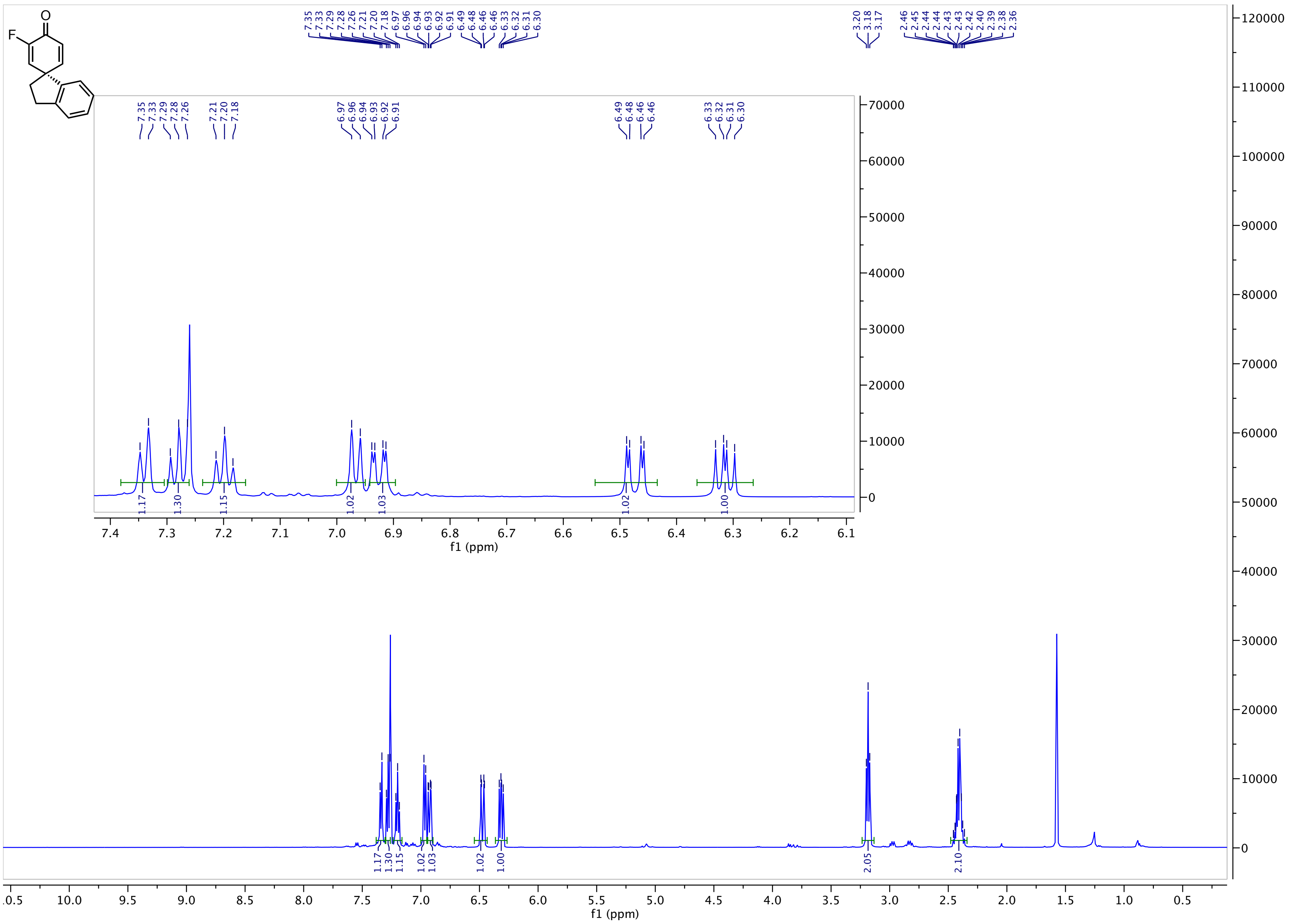

^{13}C NMR (CDCl$_3$): (*S*)-3-Fluoro-2',3'-dihydrospiro[cyclohexane-1,1'-indene]-2,5-dien-4-one **(2g)**

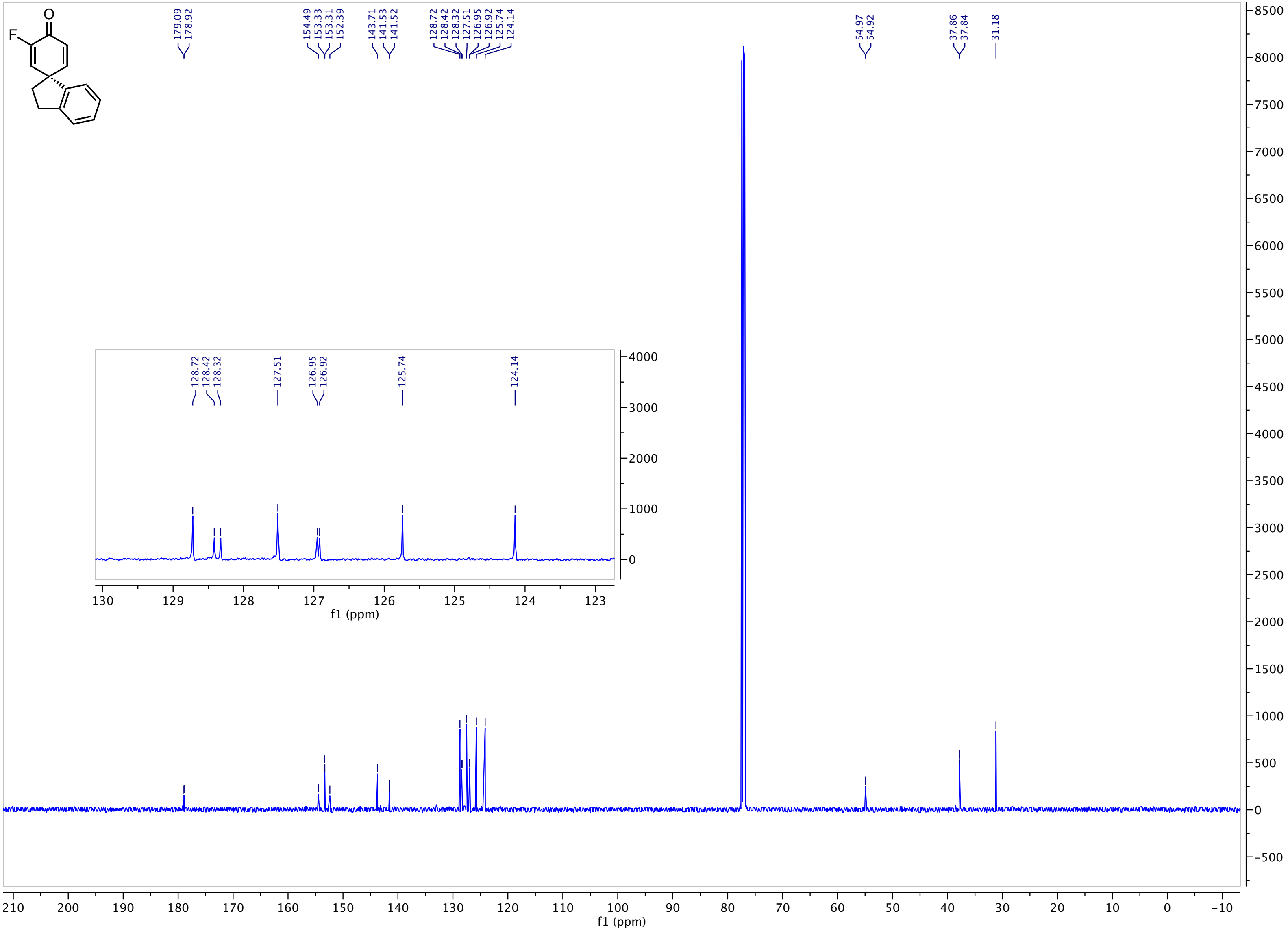

^{19}F NMR ($CDCl_3$): (*S*)-3-Fluoro-2',3'-dihydrospiro[cyclohexane-1,1'-indene]-2,5-dien-4-one **(2g)**

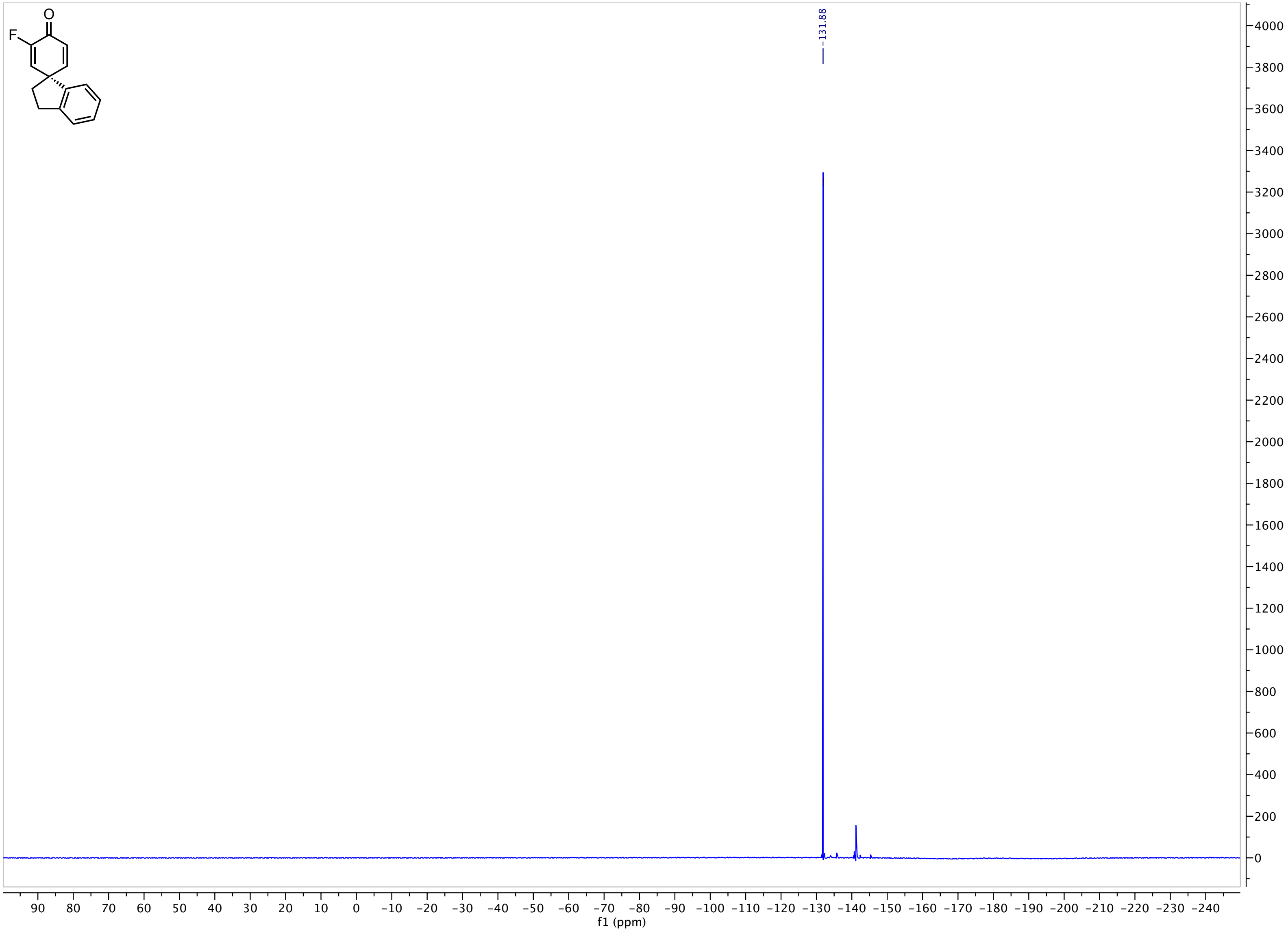

^{1}H NMR ($CDCl_3$): (*R*)-2,3-Dihydro-4'*H*-spiro[indene-1,1'-naphthalen]-4'-one **(2h)**

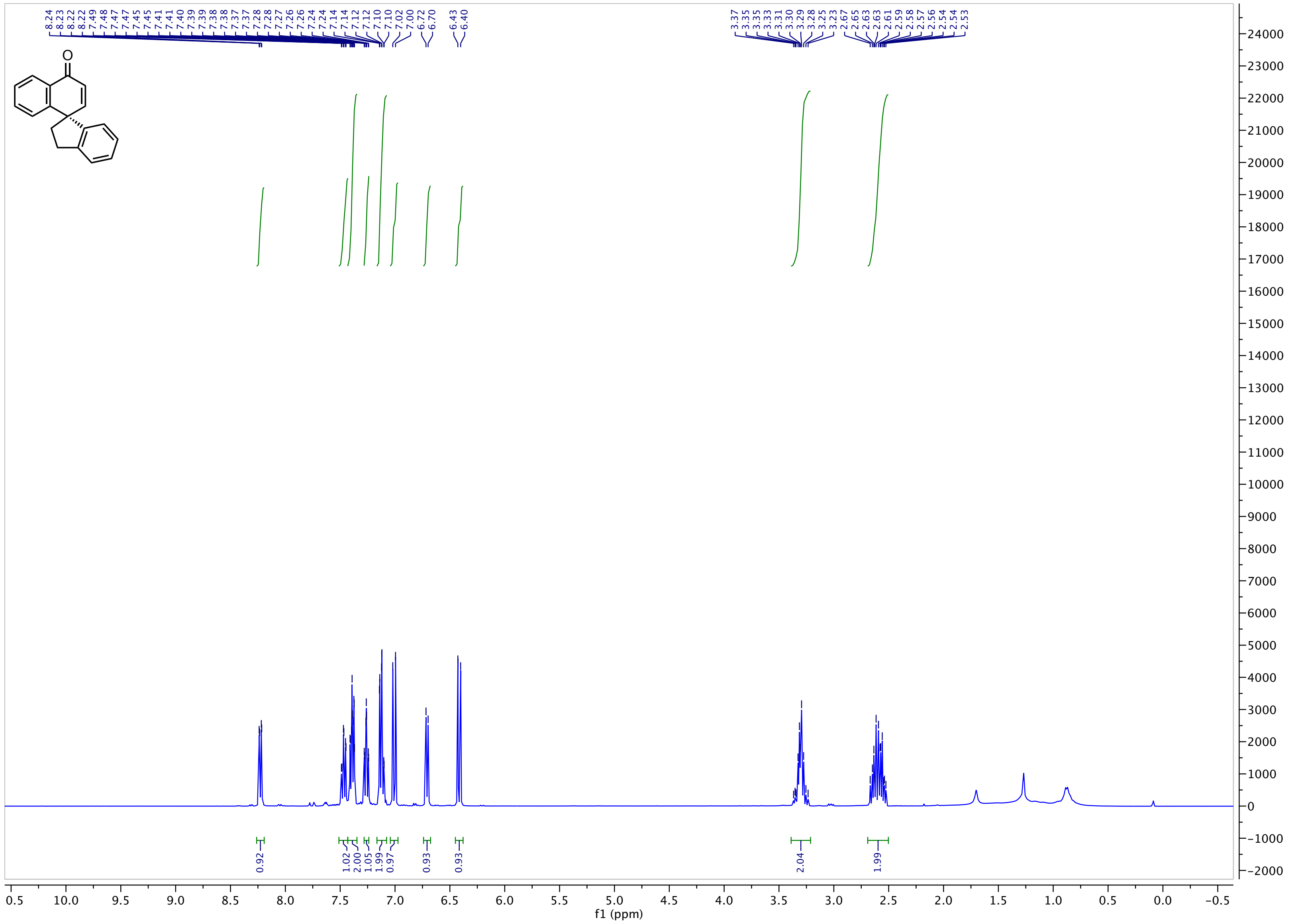

^{13}**C NMR** ($CDCl_3$): (*R*)-2,3-Dihydro-4'*H*-spiro[indene-1,1'-naphthalen]-4'-one **(2h)**

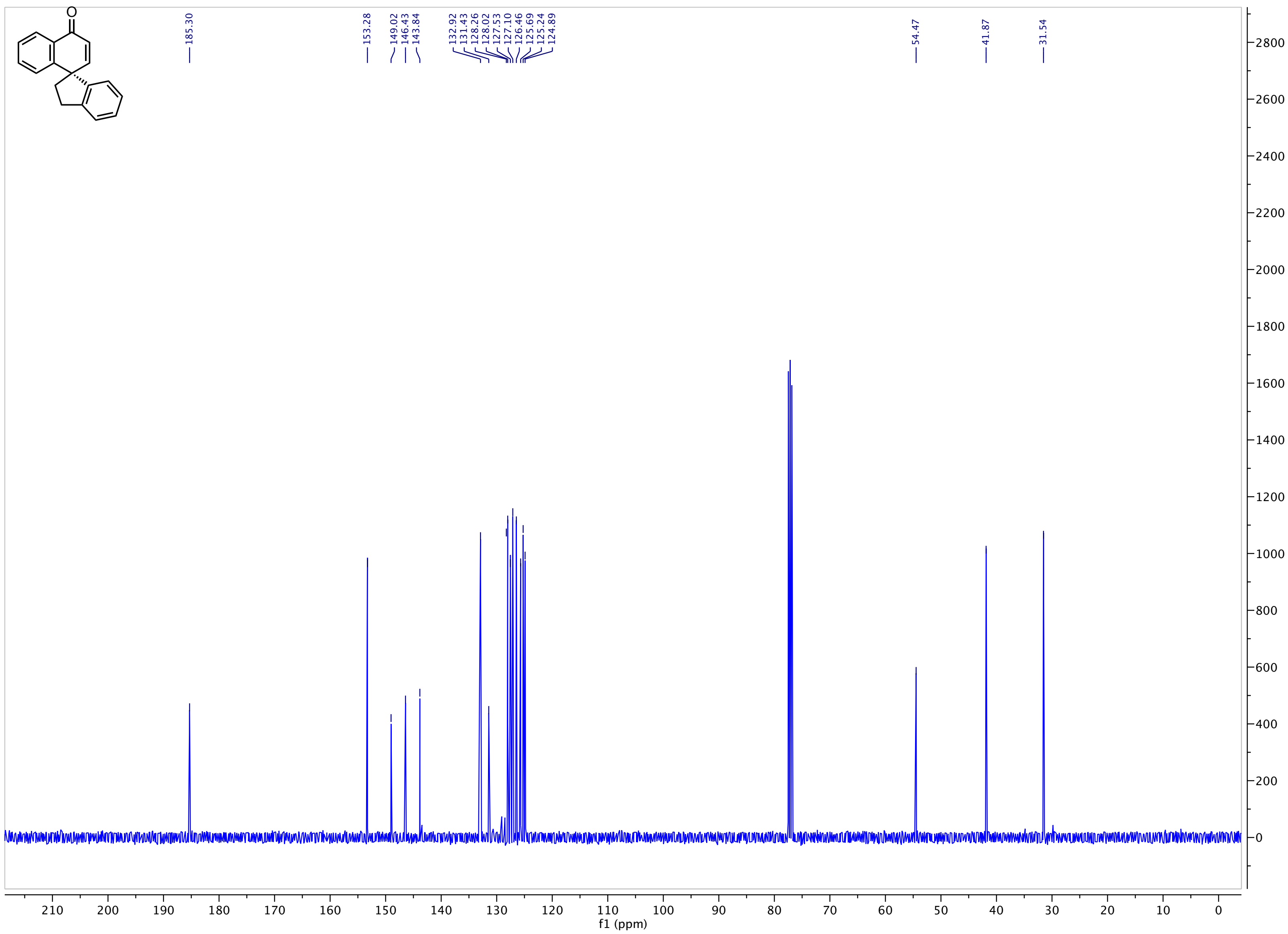

^{1}H NMR ($CDCl_3$): (*S*)-2,3-Dimethyl-2',3'-dihydrospiro[cyclohexane-1,1'-indene]-2,5-dien-4-one **(2i)**

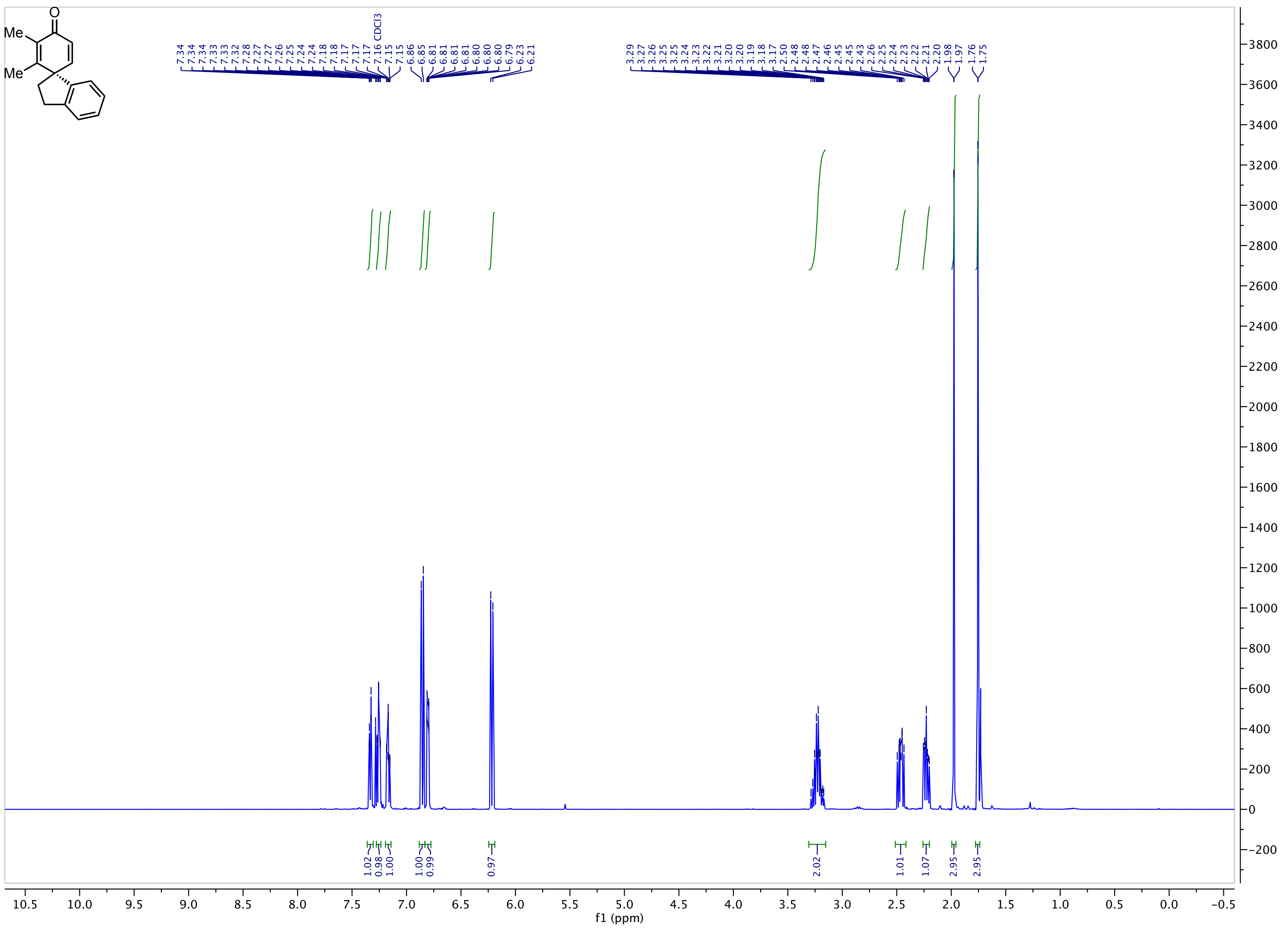

^{13}C NMR (CDCl$_3$): (*S*)-2,3-Dimethyl-2',3'-dihydrospiro[cyclohexane-1,1'-indene]-2,5-dien-4-one **(2i)**

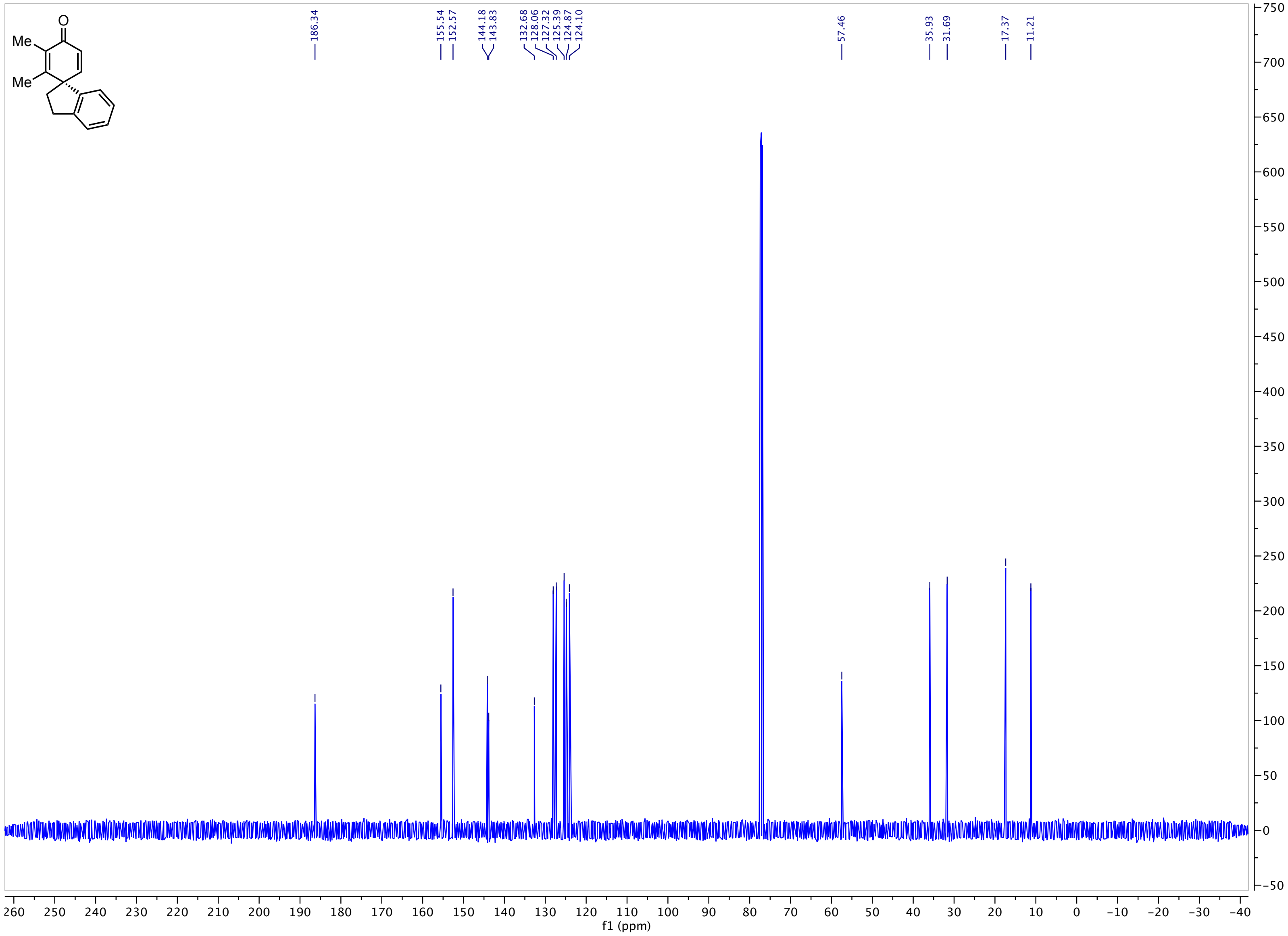

^{1}H NMR (CDCl$_3$): (*R*)-2-Methoxy-3',4'-dihydro-2'*H*-spiro[cyclohexane-1,1'-naphthalene]-2,5-dien-4-one **(2j)**

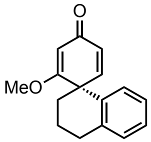

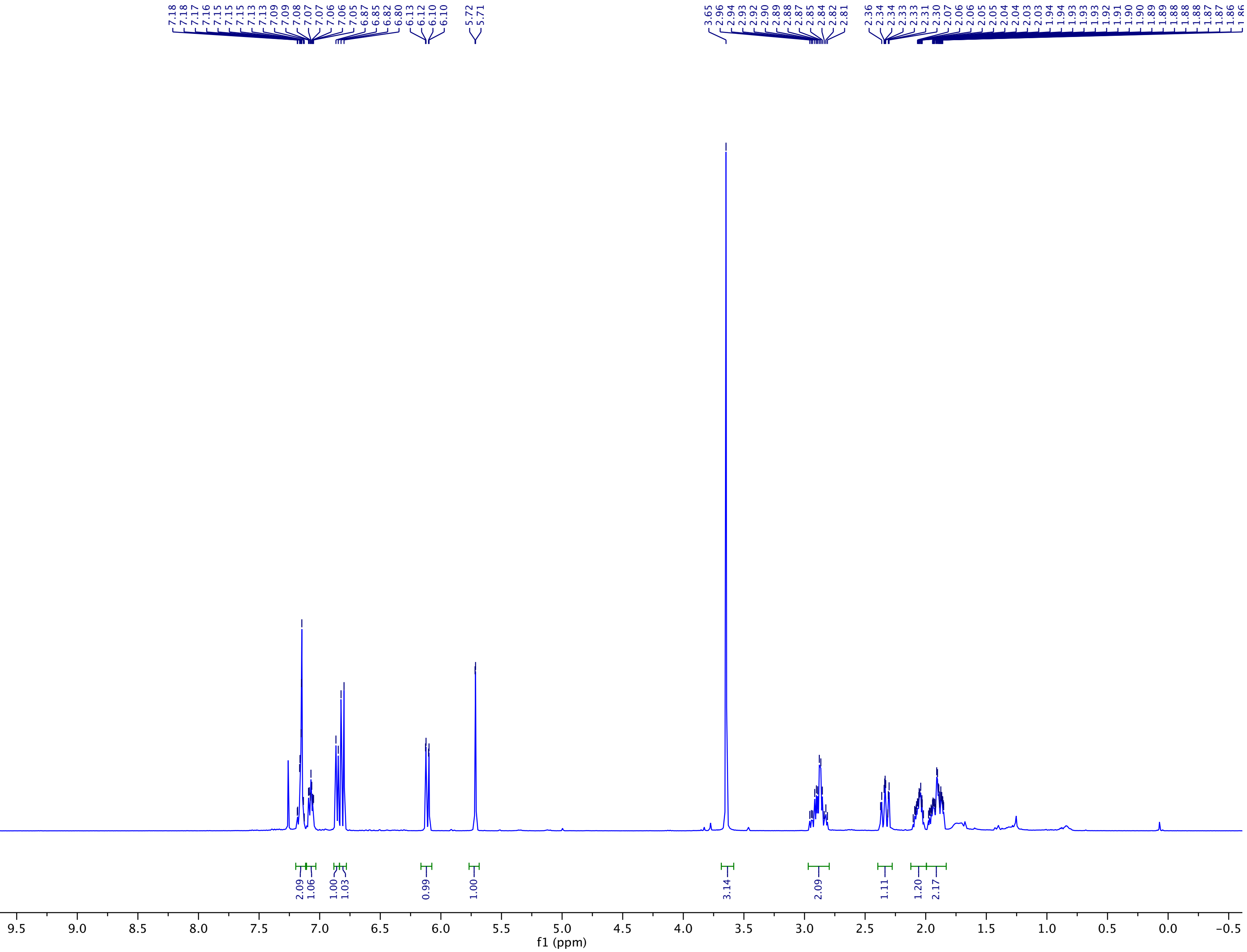

^{13}C NMR ($CDCl_3$): (*R*)-2-Methoxy-3',4'-dihydro-2'*H*-spiro[cyclohexane-1,1'-naphthalene]-2,5-dien-4-one **(2j)**

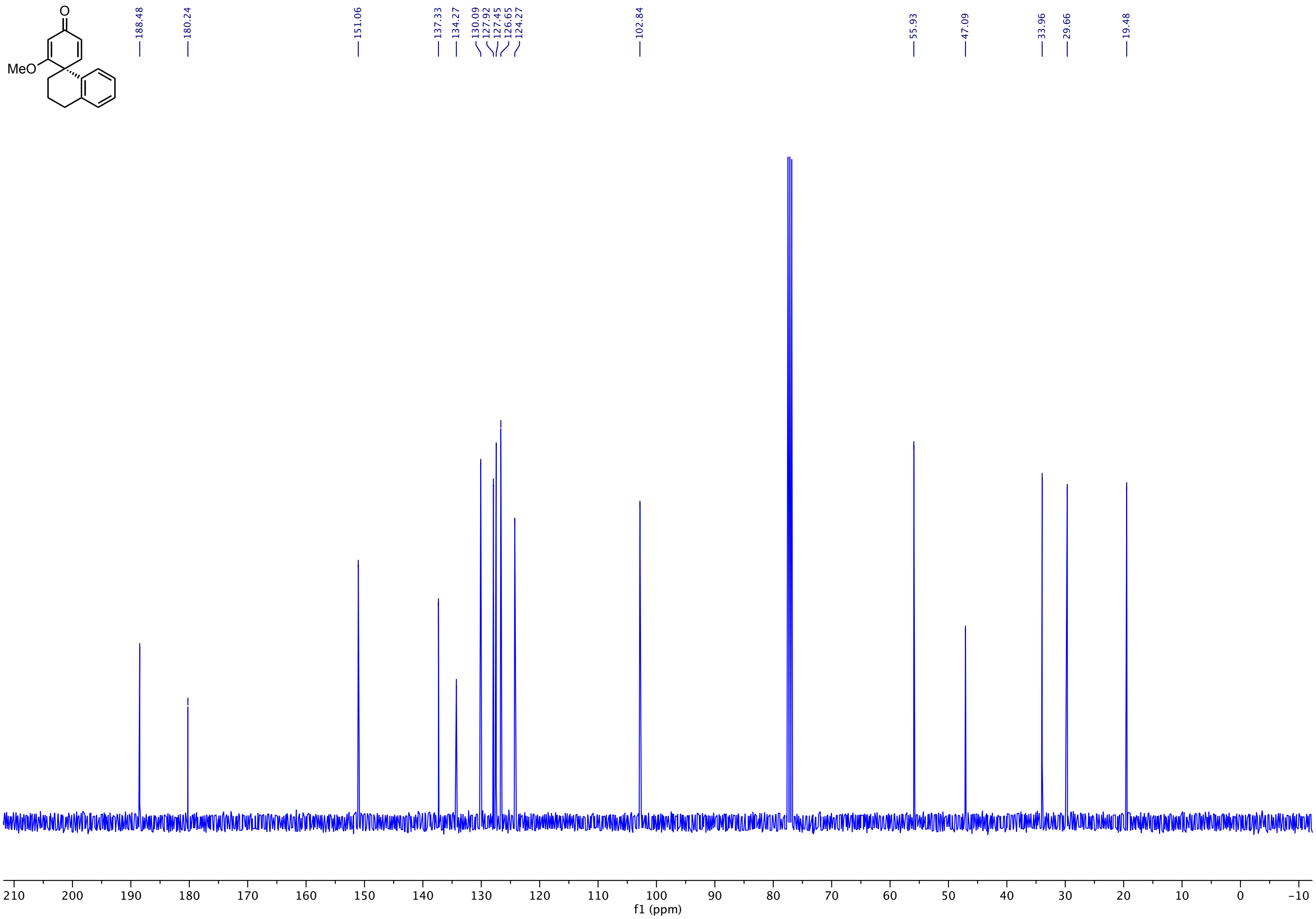

^{1}H NMR (CDCl$_3$): (*R*)-2'-Methoxy-6,7,8,9-tetrahydrospiro[benzo[7]annulene-5,1'-cyclohexane]-2',5'-dien-4'-one **(2k)**

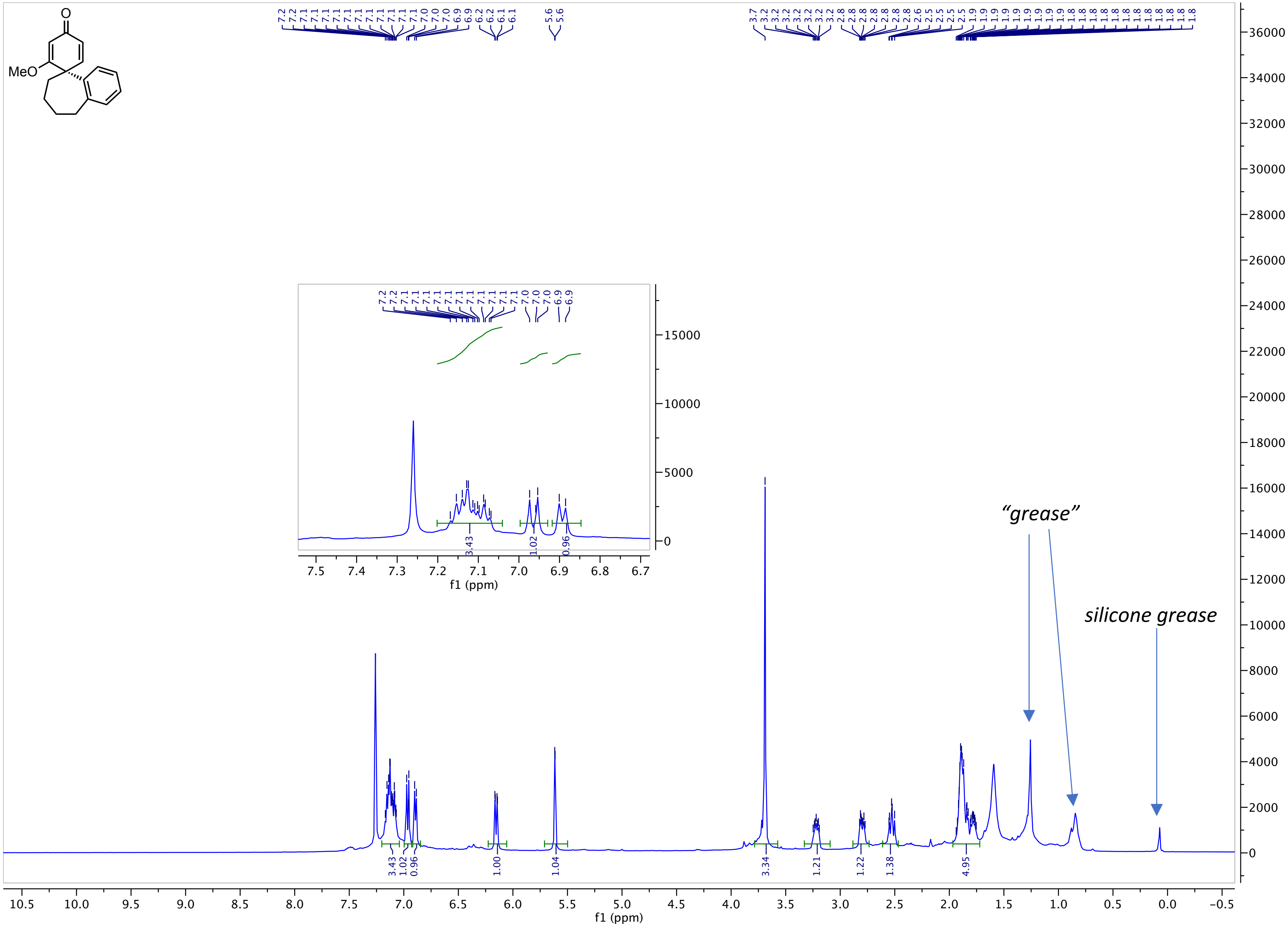

^{13}C NMR ($CDCl_3$): (*R*)-2'-Methoxy-6,7,8,9-tetrahydrospiro[benzo[7]annulene-5,1'-cyclohexane]-2',5'-dien-4'-one **(2k)**

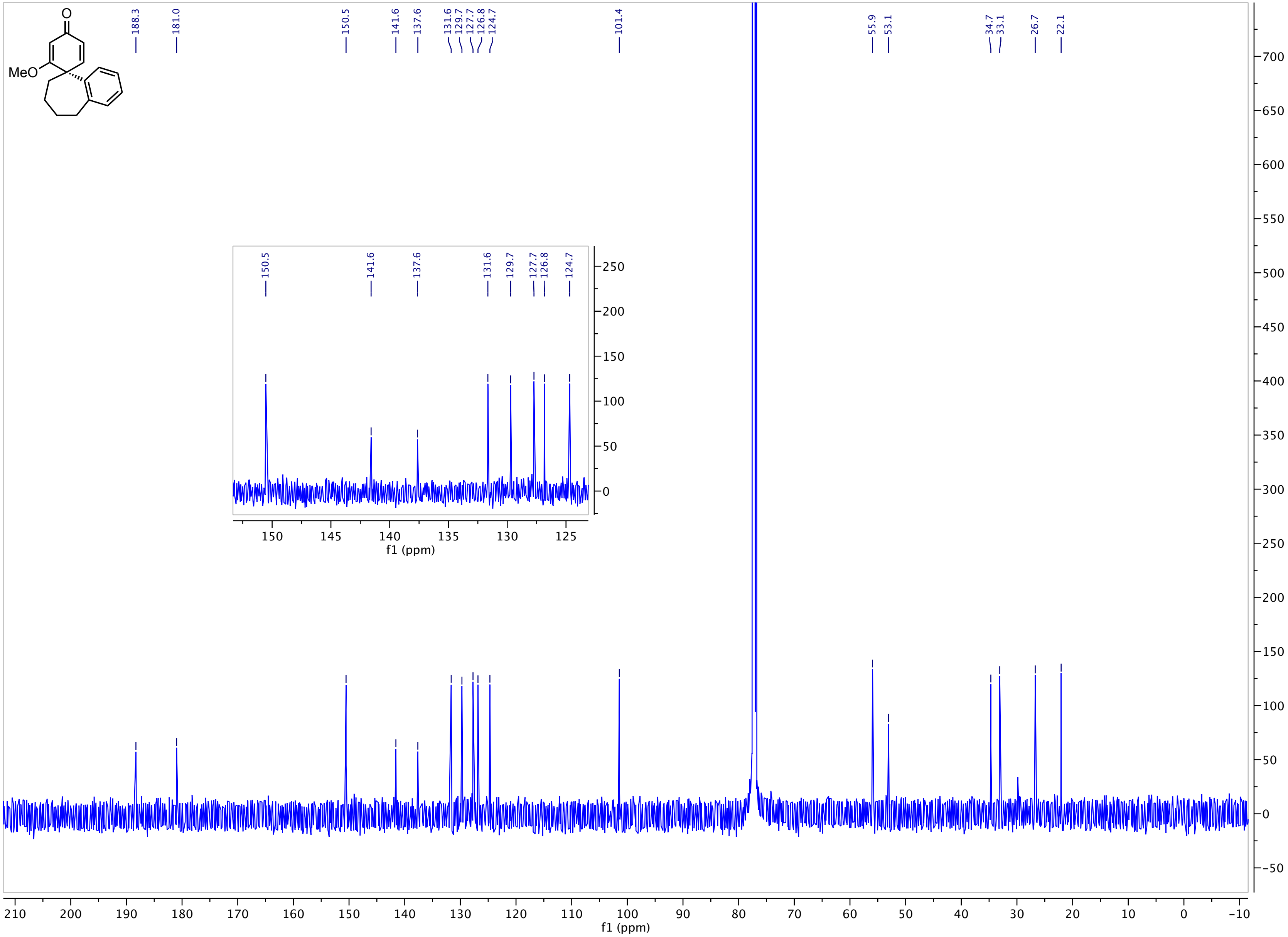

^{1}H NMR ($CDCl_3$): (*R*)-2-Methoxy-5'-(trifluoromethyl)-2',3'-dihydrospiro[cyclohexane-1,1'-indene]-2,5-dien-4-one **(2l)**

(0.1 mmol)

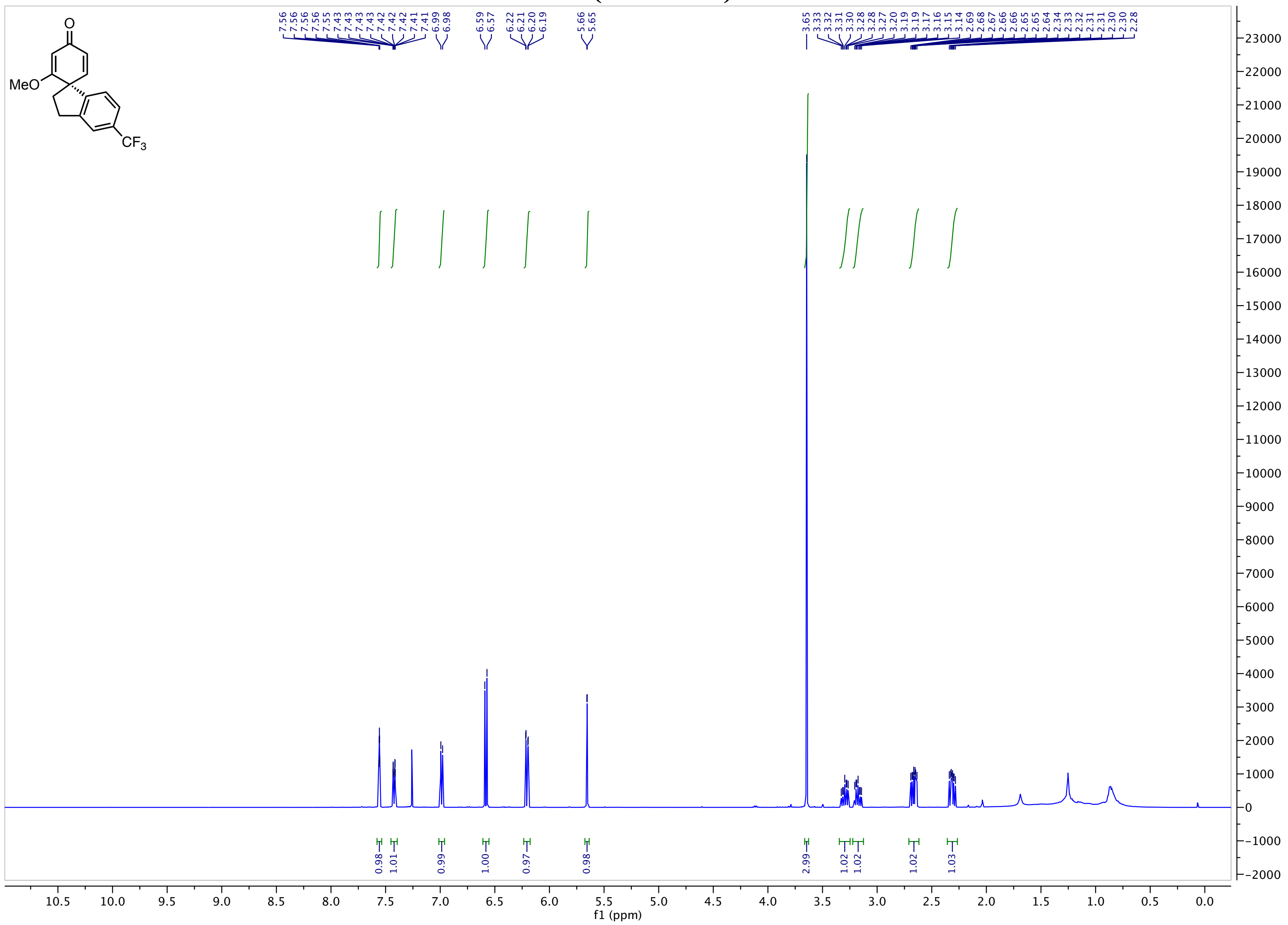

^{13}C NMR ($CDCl_3$): (*R*)-2-Methoxy-5'-(trifluoromethyl)-2',3'-dihydrospiro[cyclohexane-1,1'-indene]-2,5-dien-4-one **(2l)**

0.1 mmol

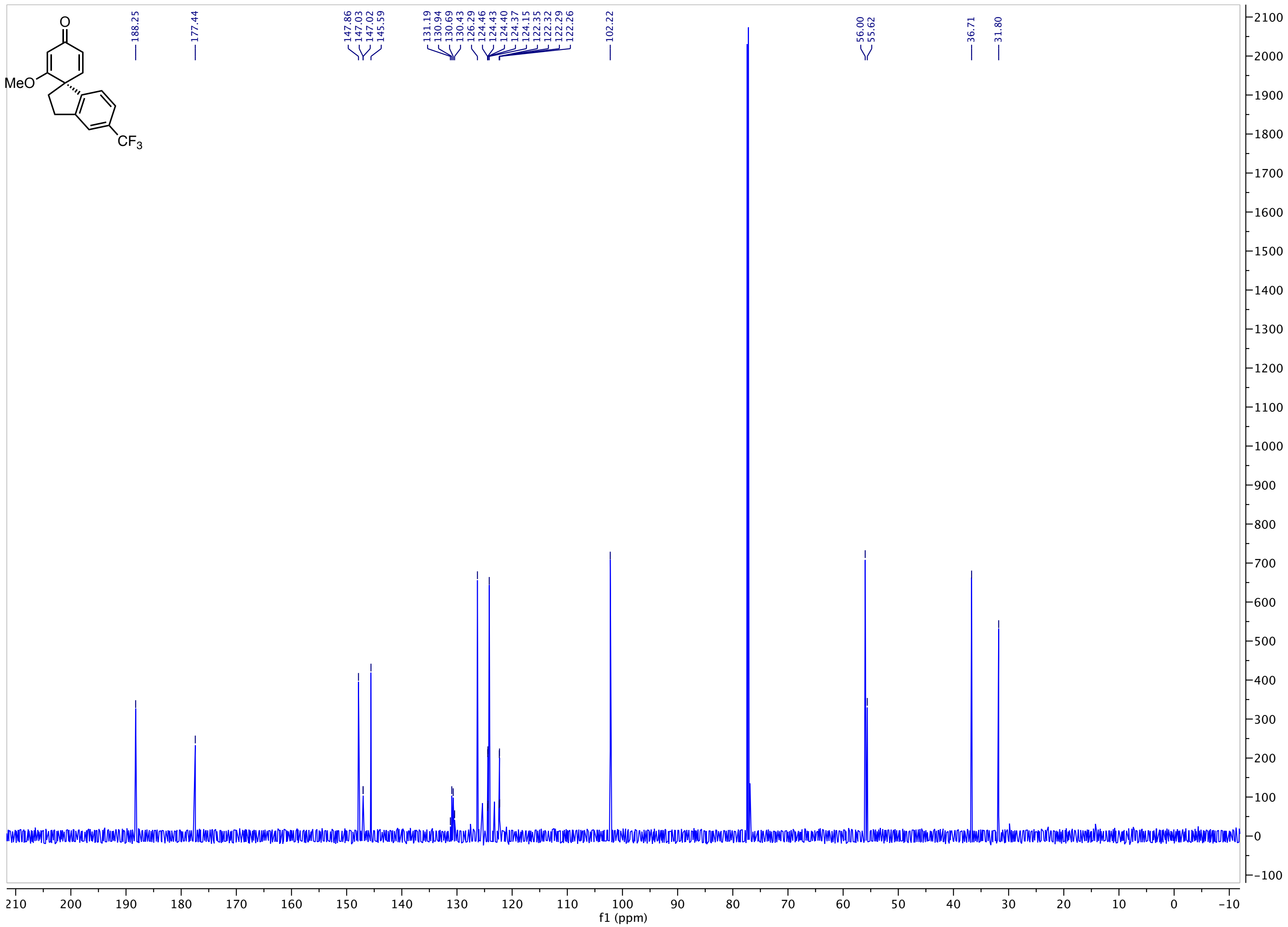

^{19}F NMR (CDCl$_3$): (*R*)-2-Methoxy-5'-(trifluoromethyl)-2',3'-dihydrospiro[cyclohexane-1,1'-indene]-2,5-dien-4-one **(2l) 0.1 mmol**

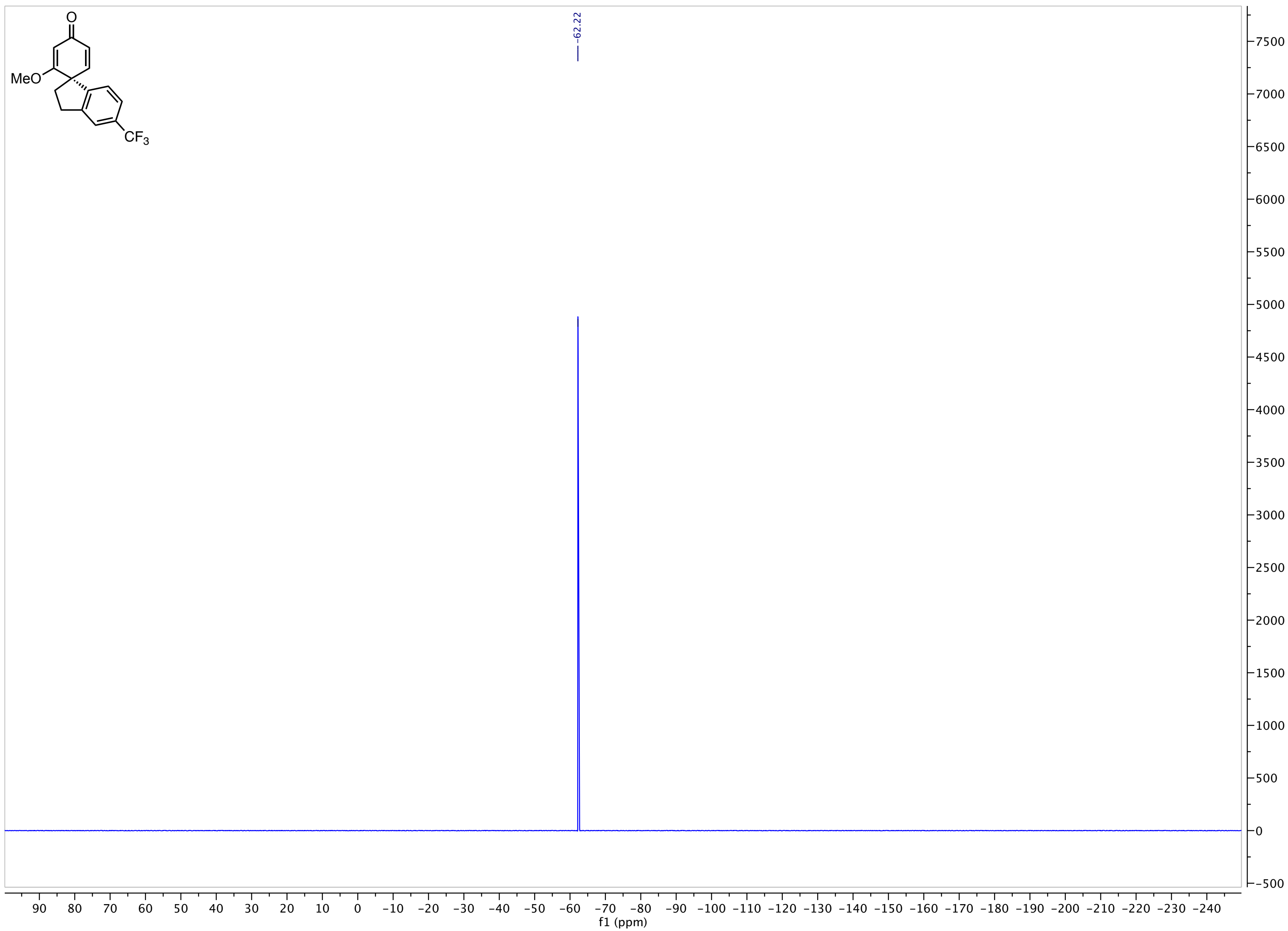

^{1}H NMR ($CDCl_3$): (*R*)-2-Methoxy-5'-(trifluoromethyl)-2',3'-dihydrospiro[cyclohexane-1,1'-indene]-2,5-dien-4-one **(2l)**

1.0 mmol

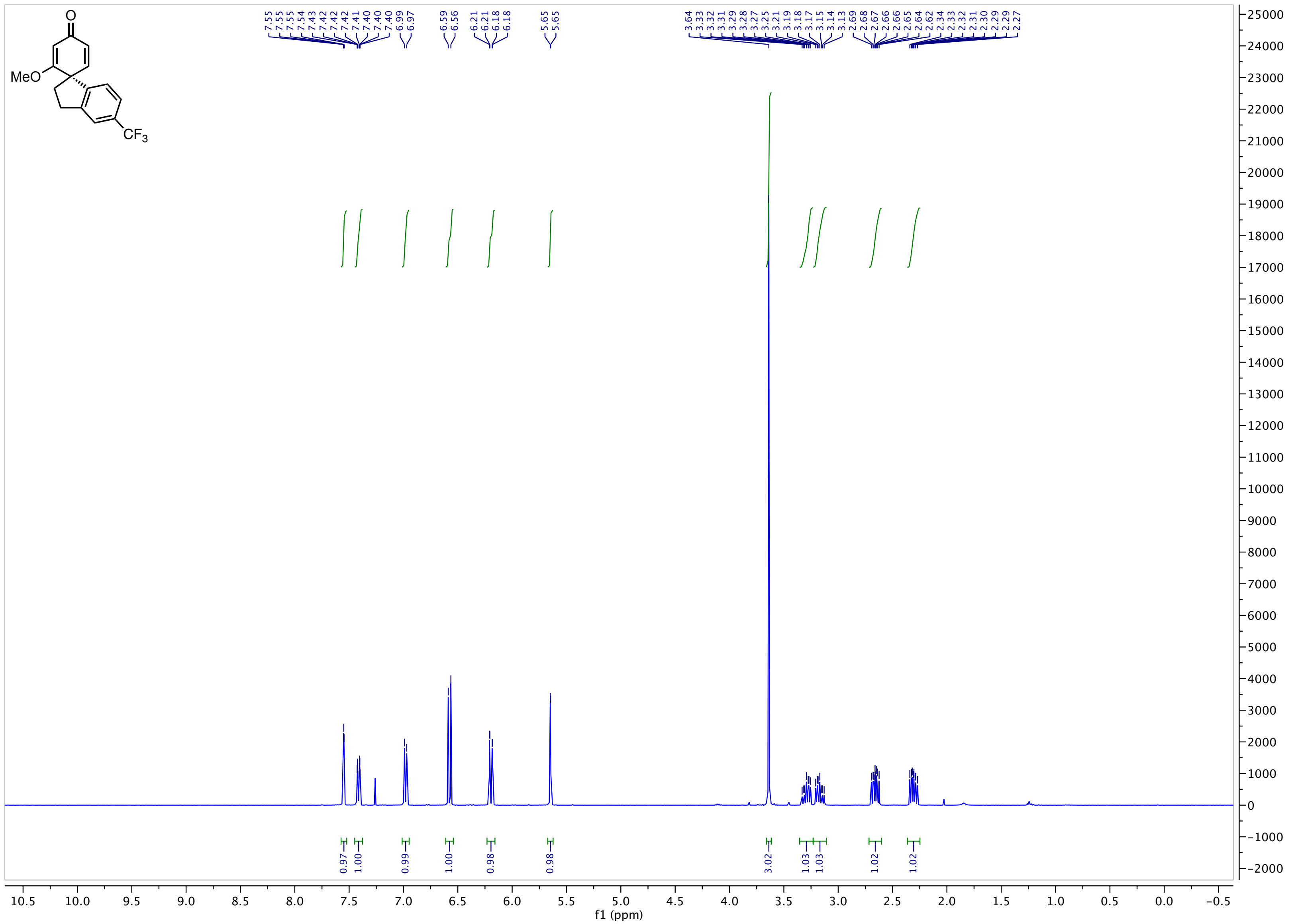

^{13}C **NMR** ($CDCl_3$): (*R*)-2-Methoxy-5'-(trifluoromethyl)-2',3'-dihydrospiro[cyclohexane-1,1'-indene]-2,5-dien-4-one **(2l)**

1.0 mmol

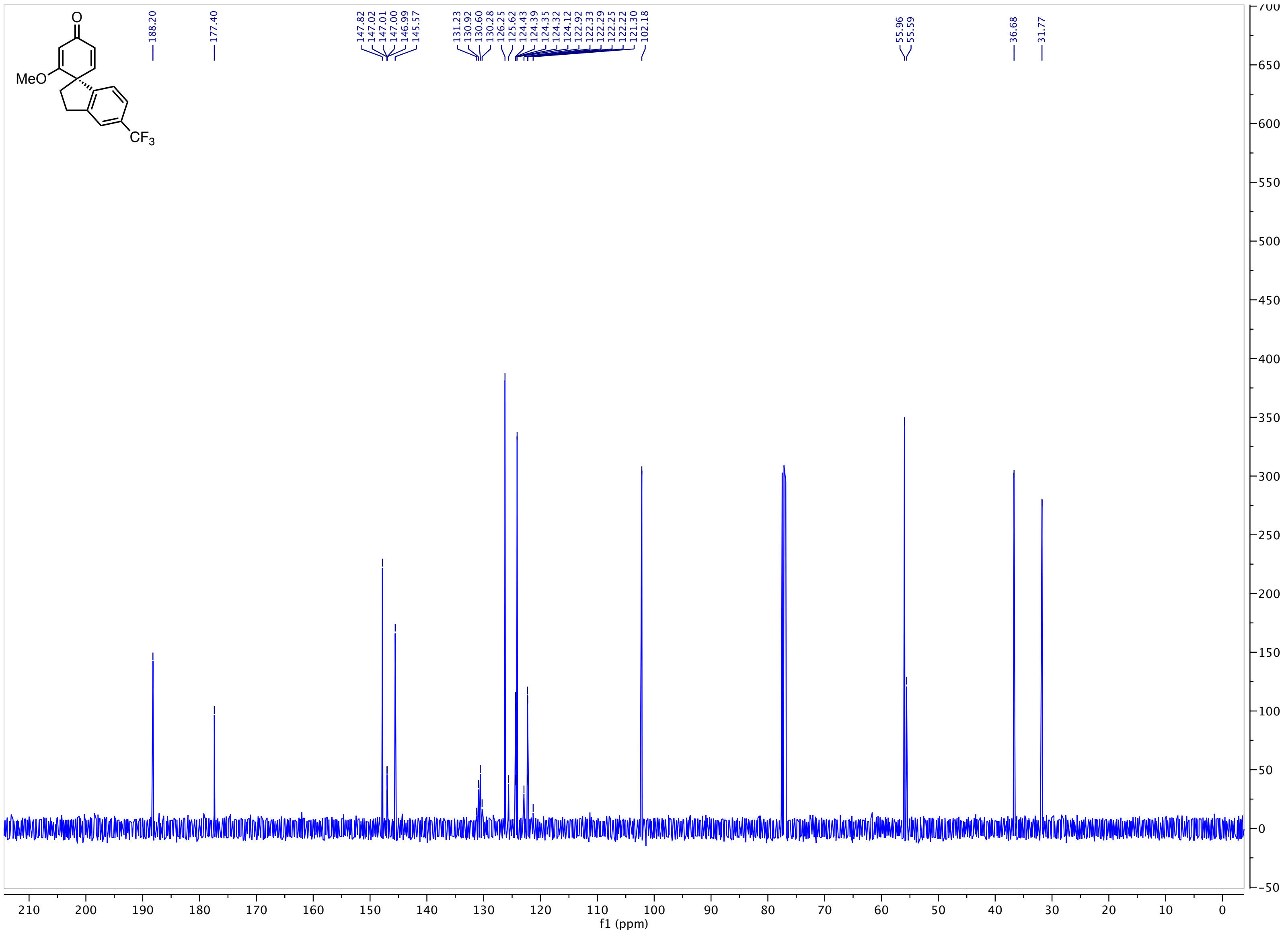

^{19}F NMR ($CDCl_3$): (*R*)-2-Methoxy-5'-(trifluoromethyl)-2',3'-dihydrospiro[cyclohexane-1,1'-indene]-2,5-dien-4-one **(2l)**

1.0 mmol

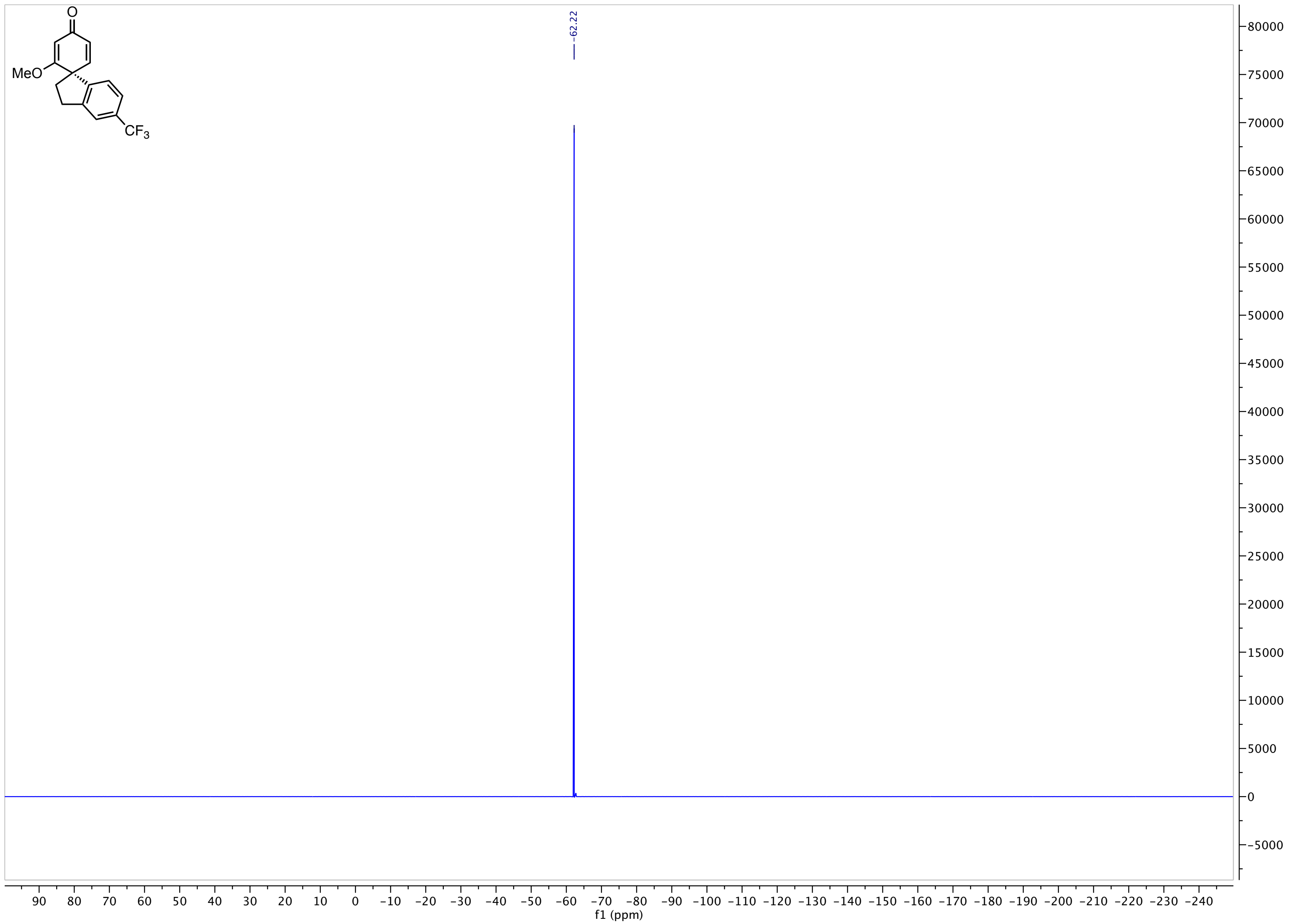

^{1}H NMR ($CDCl_3$): (*R*)-2,5',6'-Trimethoxy-2',3'-dihydrospiro[cyclohexane-1,1'-indene]-2,5-dien-4-one **(2m)**

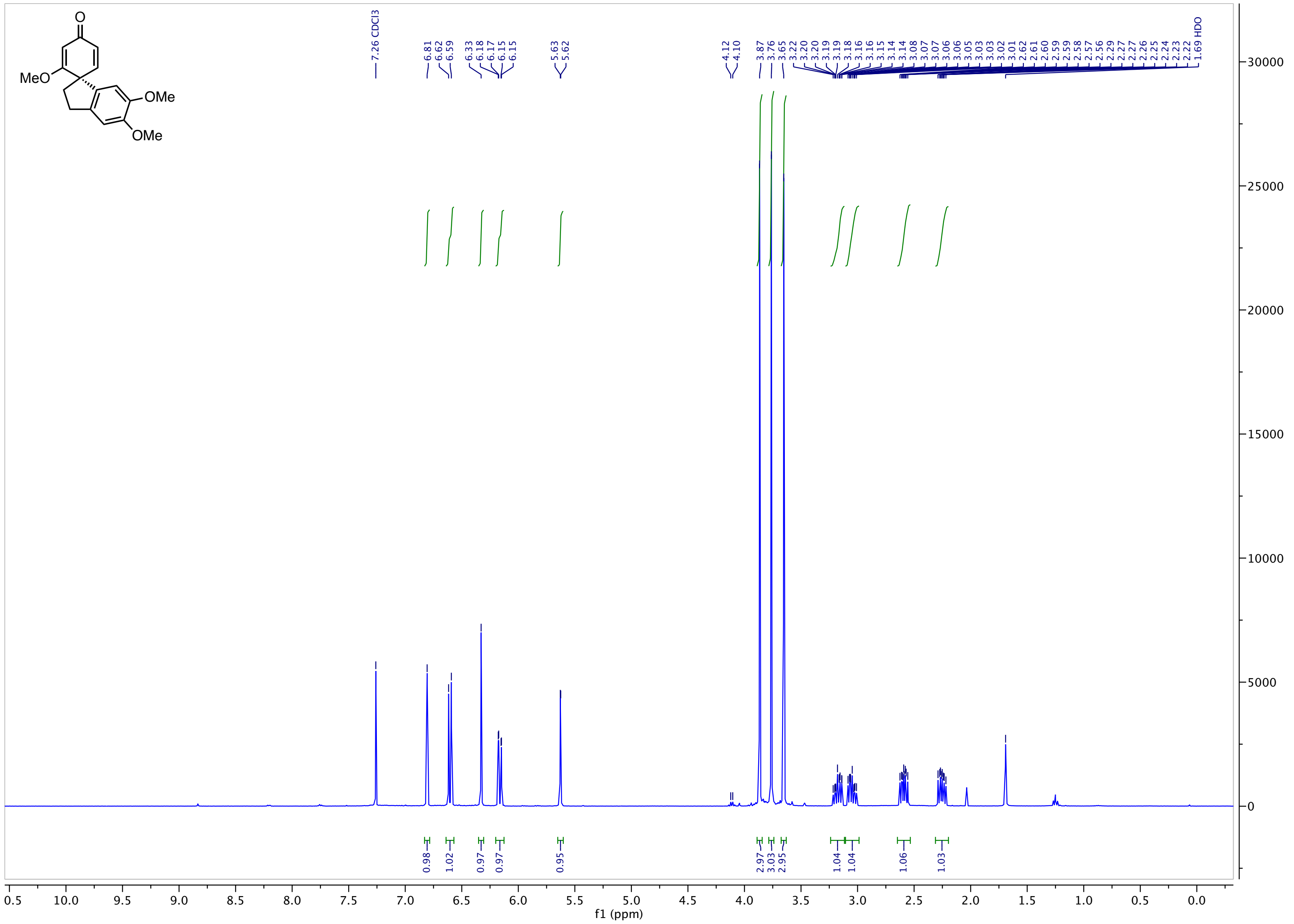

^{13}C NMR ($CDCl_3$): (*R*)-2,5',6'-Trimethoxy-2',3'-dihydrospiro[cyclohexane-1,1'-indene]-2,5-dien-4-one **(2m)**

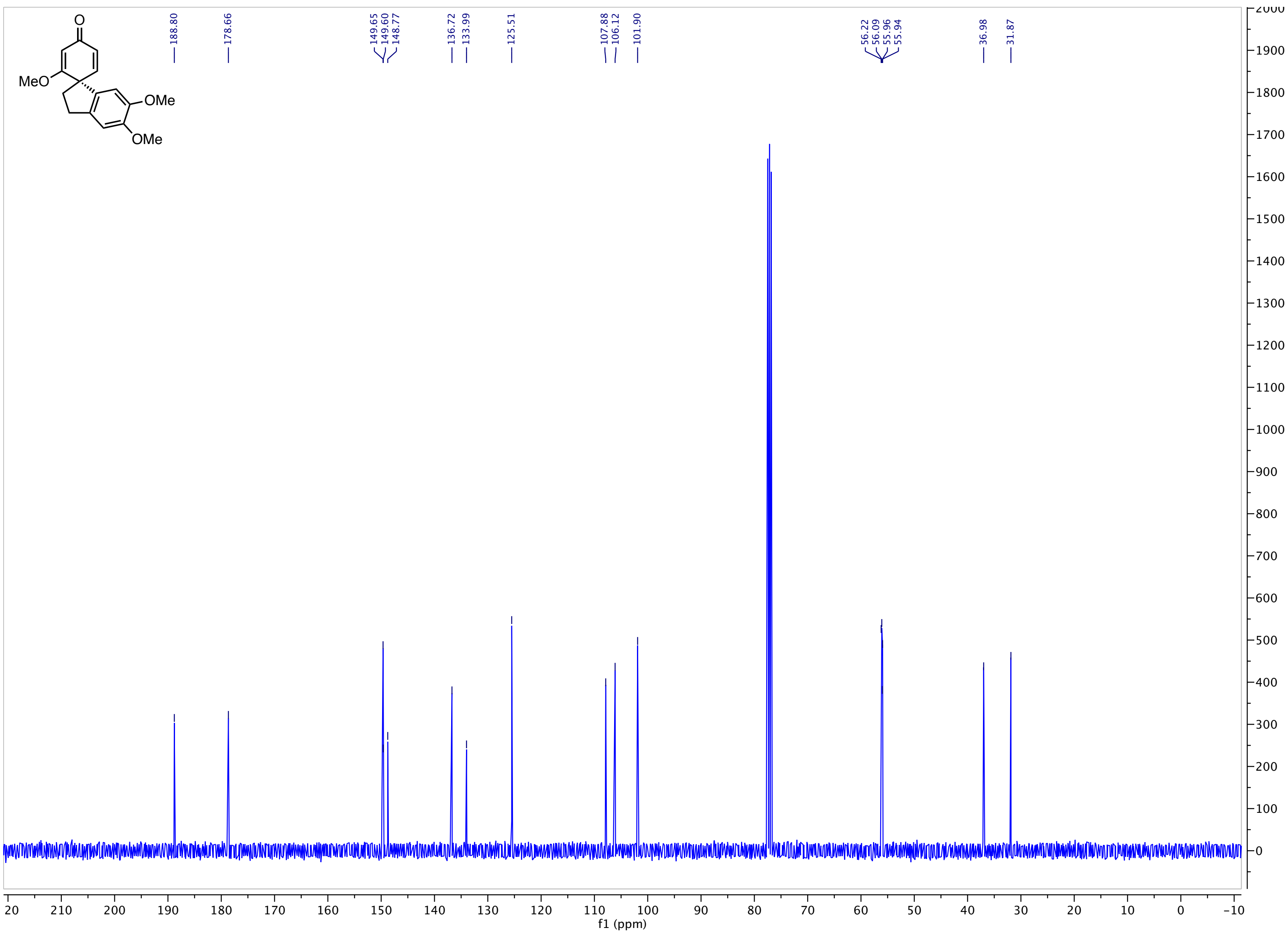

^{1}H NMR ($CDCl_3$): (*R*)-4'-Chloro-2-methoxy-2',3'-dihydrospiro[cyclohexane-1,1'-indene]-2,5-dien-4-one **(2n)**

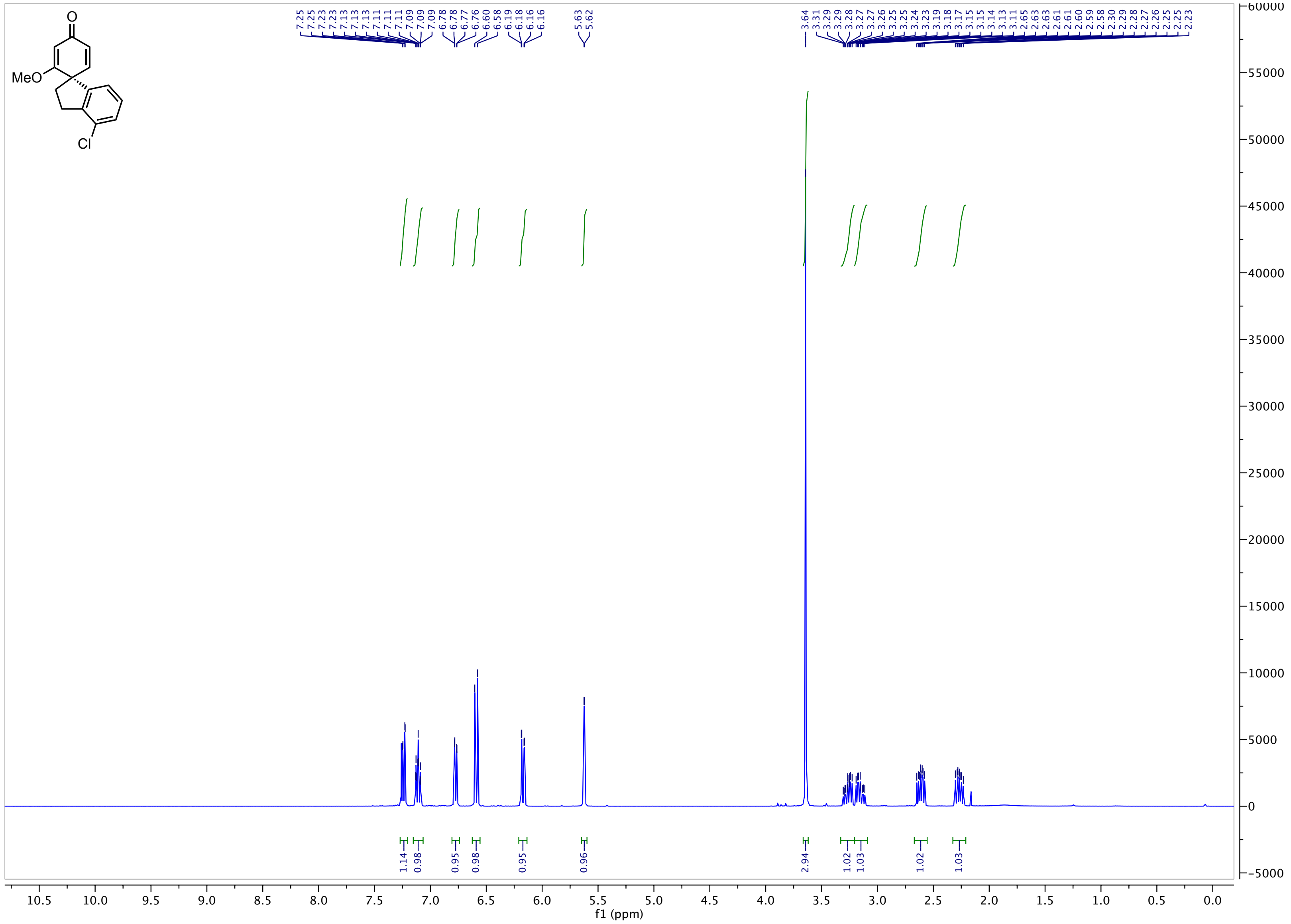

^{13}C NMR ($CDCl_3$): (*R*)-4'-Chloro-2-methoxy-2',3'-dihydrospiro[cyclohexane-1,1'-indene]-2,5-dien-4-one **(2n)**

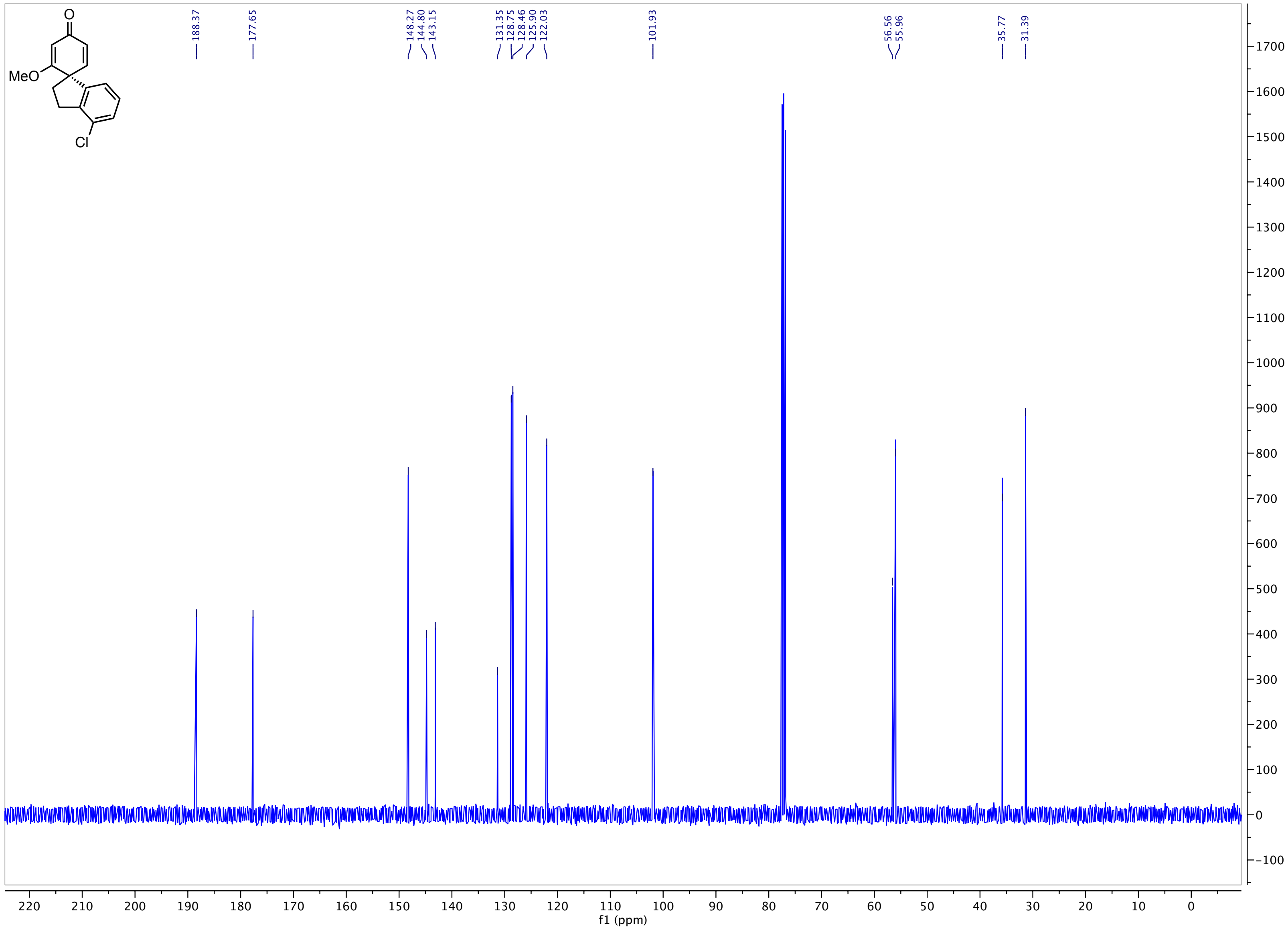

^{1}H NMR ($CDCl_3$): *tert*-Butyl (*R*)-(2-methoxy-4-oxo-2',3'-dihydrospiro[cyclohexane-1,1'-indene]-2,5-dien-6'-yl)carbamate **(2o)**

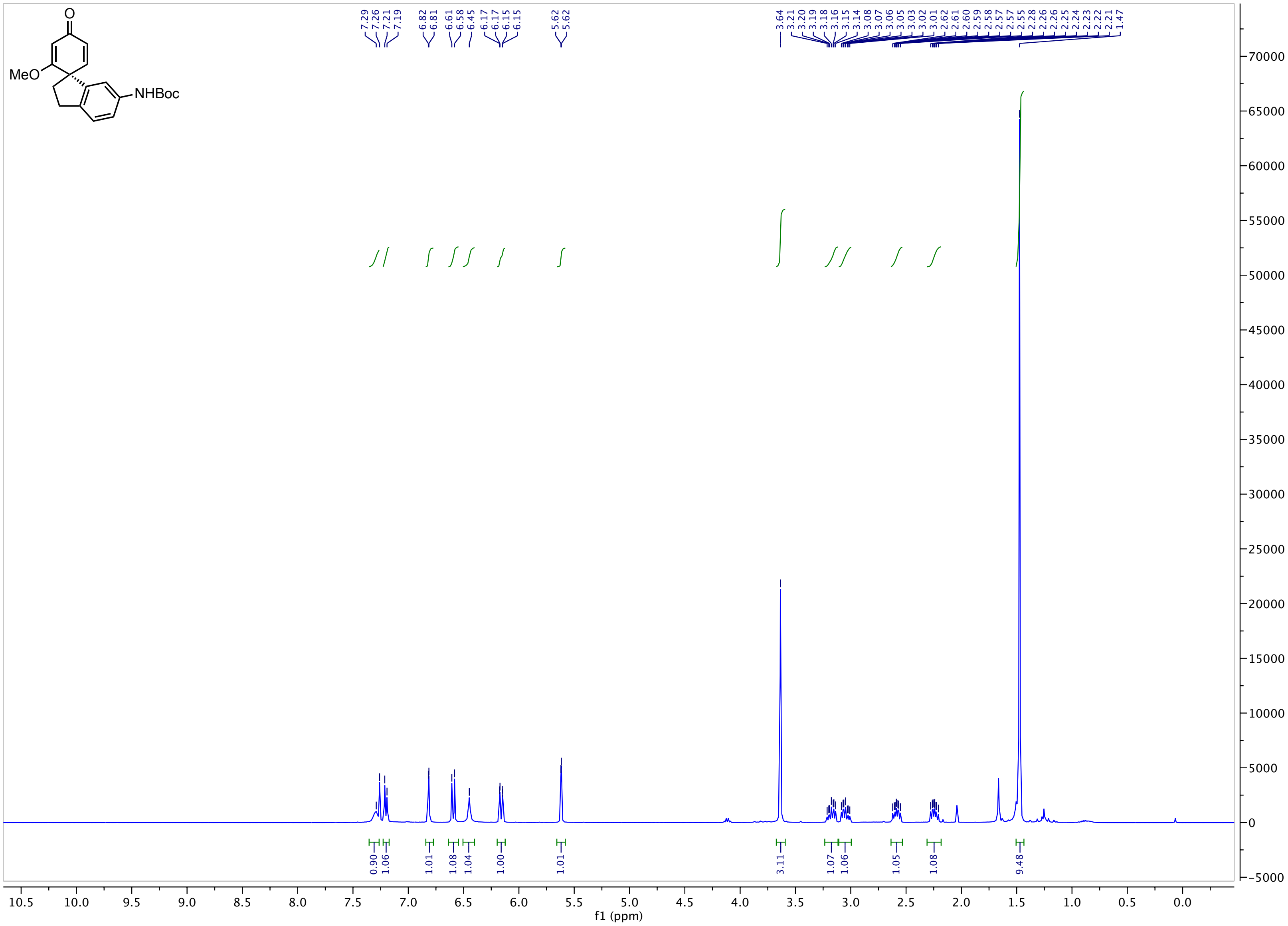

^{13}C NMR ($CDCl_3$): *tert*-Butyl (*R*)-(2-methoxy-4-oxo-2',3'-dihydrospiro[cyclohexane-1,1'-indene]-2,5-dien-6'-yl)carbamate **(2o)**

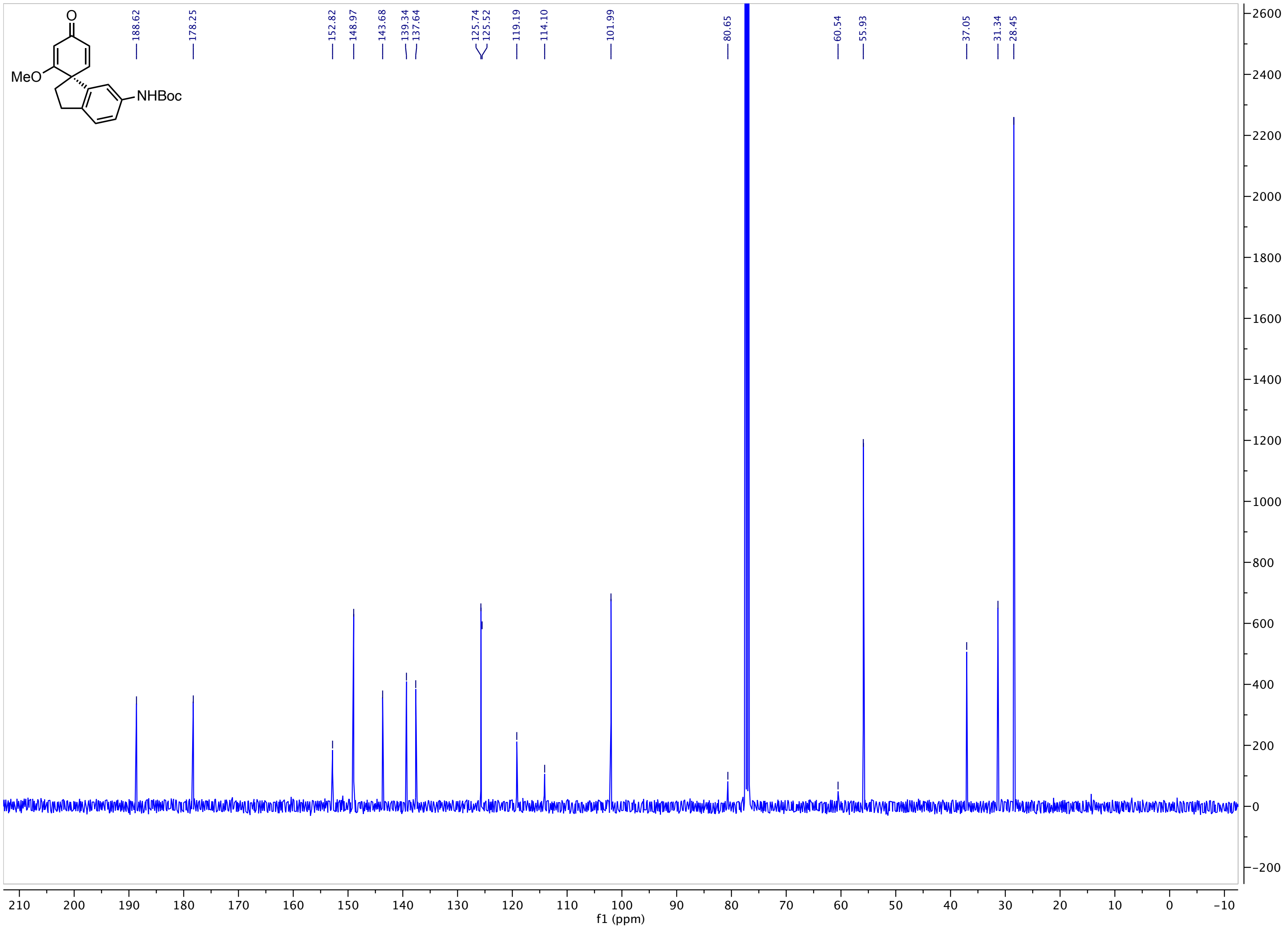

^{1}H NMR ($CDCl_3$): *tert*-Butyl (*R*)-2-methoxy-4-oxo-2',3'-dihydrospiro[cyclohexane-1,1'-indene]-2,5-diene-6'-carboxylate (**2p**)

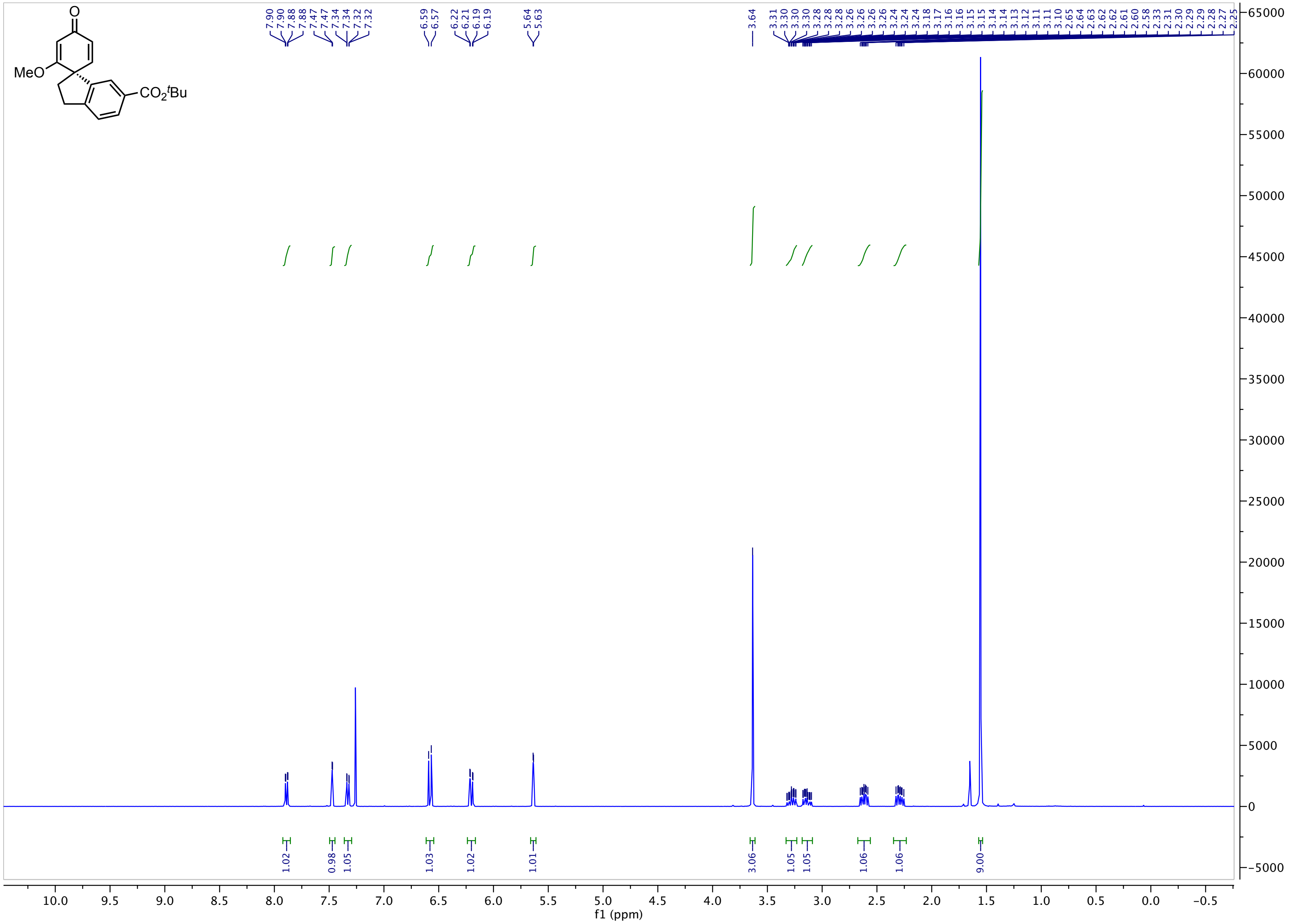

^{13}C NMR ($CDCl_3$): *tert*-Butyl (*R*)-2-methoxy-4-oxo-2',3'-dihydrospiro[cyclohexane-1,1'-indene]-2,5-diene-6'-carboxylate (**2p**)

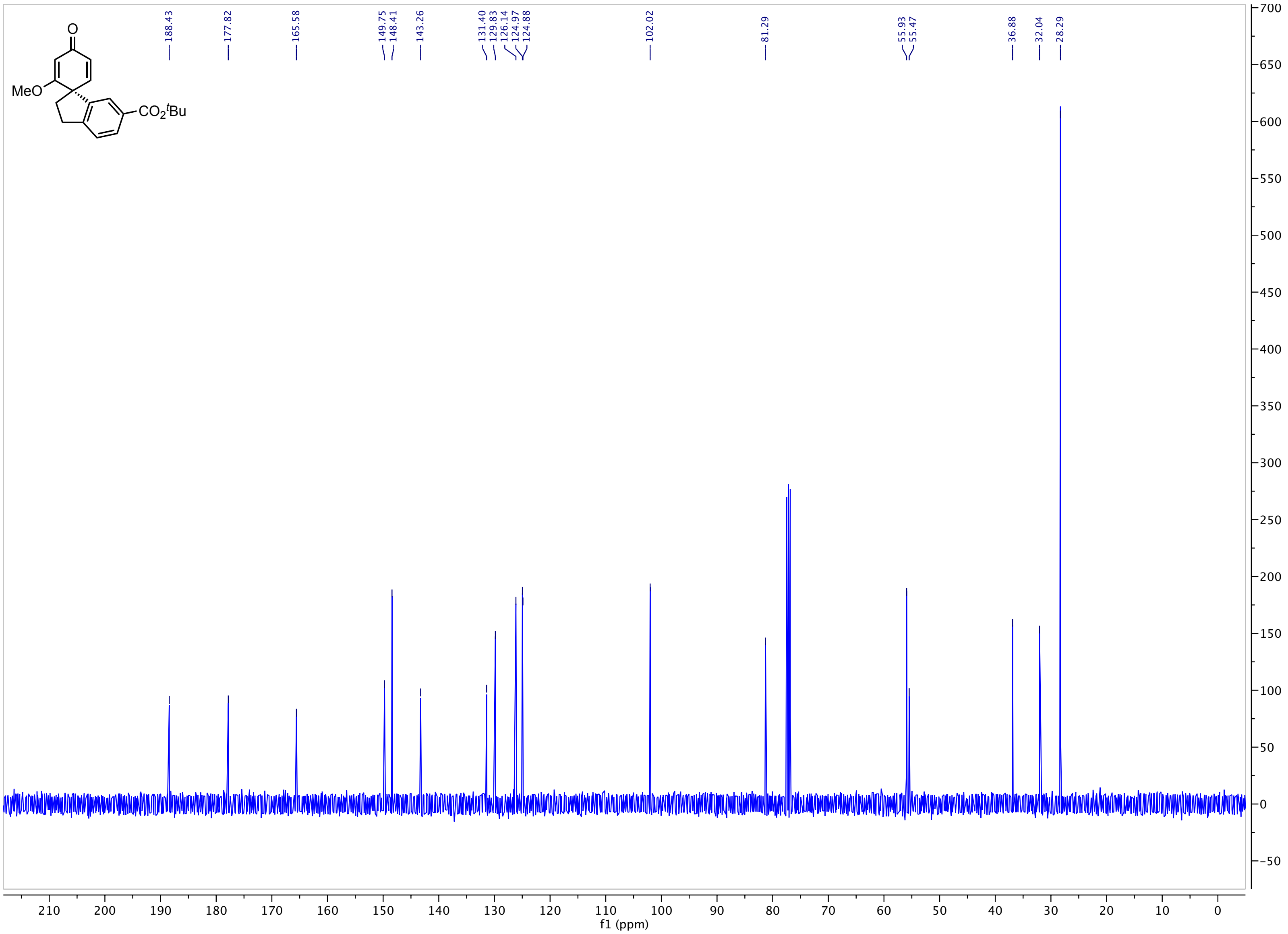

^{1}H NMR (CDCl$_3$): (*R*)-2-Methoxy-7'-methyl-2',3'-dihydrospiro[cyclohexane-1,1'-indene]-2,5-dien-4-one (**2q**)

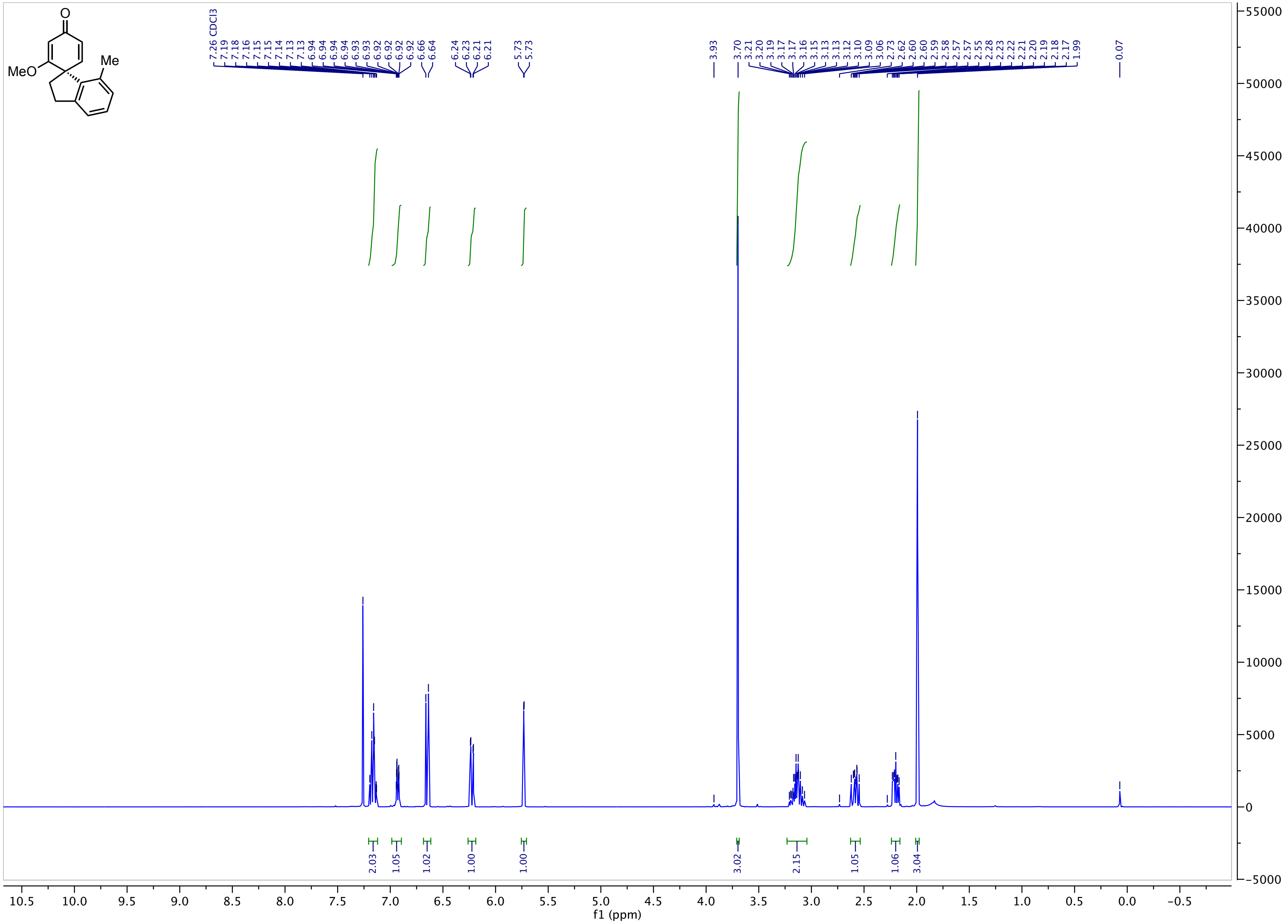

^{13}**C NMR** ($CDCl_3$): (*R*)-2-Methoxy-7'-methyl-2',3'-dihydrospiro[cyclohexane-1,1'-indene]-2,5-dien-4-one (**2q**)

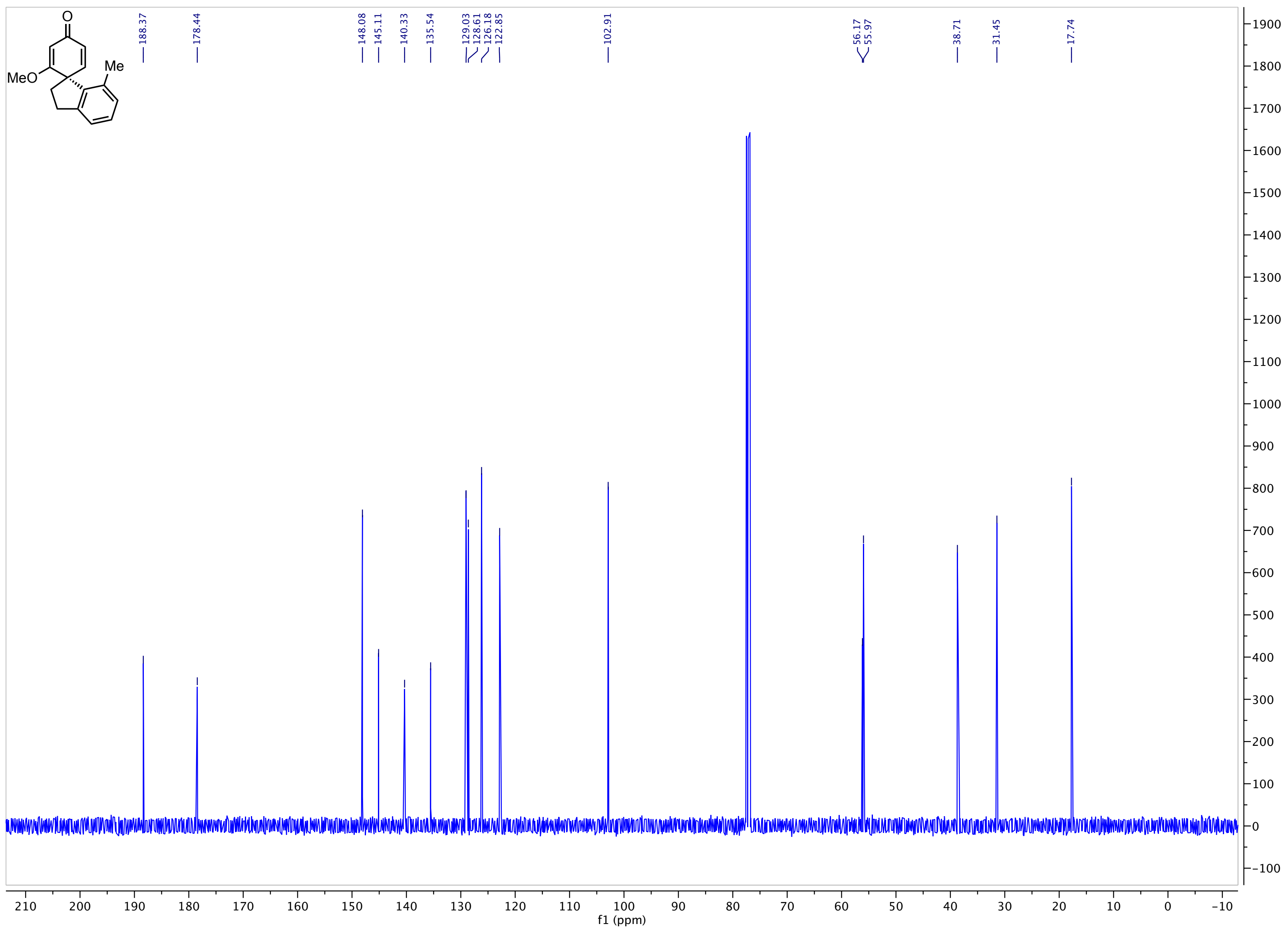

^{1}H NMR ($CDCl_3$): (*R*)-5'-Fluoro-2-methoxy-2',3'-dihydrospiro[cyclohexane-1,1'-indene]-2,5-dien-4-one **(2r)**

0.1 mmol

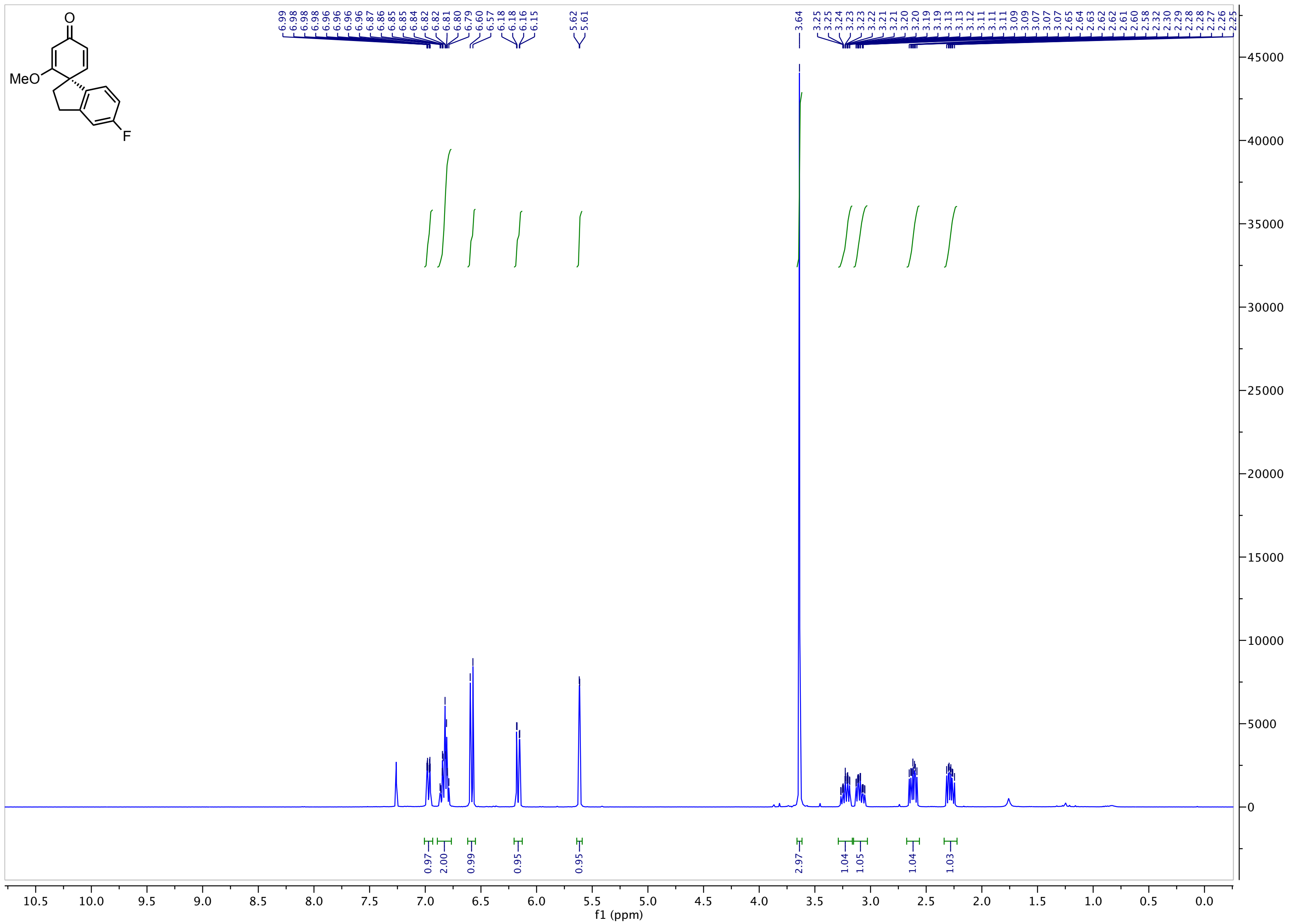

^{13}**C NMR** ($CDCl_3$): (*R*)-5’-Fluoro-2-methoxy-2',3'-dihydrospiro[cyclohexane-1,1'-indene]-2,5-dien-4-one **(2r)**

0.1 mmol

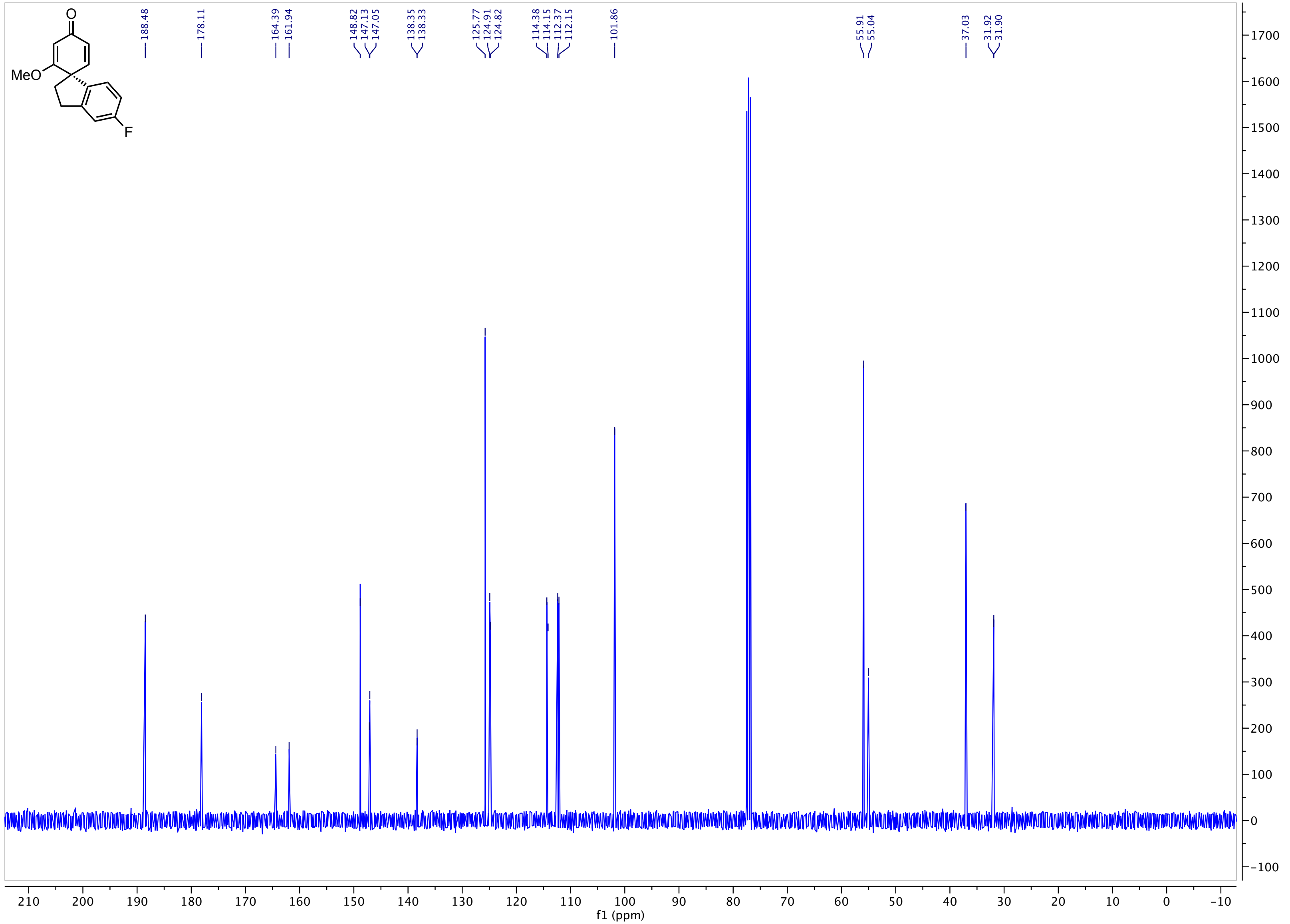

^{19}F NMR ($CDCl_3$): (*R*)-5’-Fluoro-2-methoxy-2',3'-dihydrospiro[cyclohexane-1,1'-indene]-2,5-dien-4-one **(2r)**

0.1 mmol

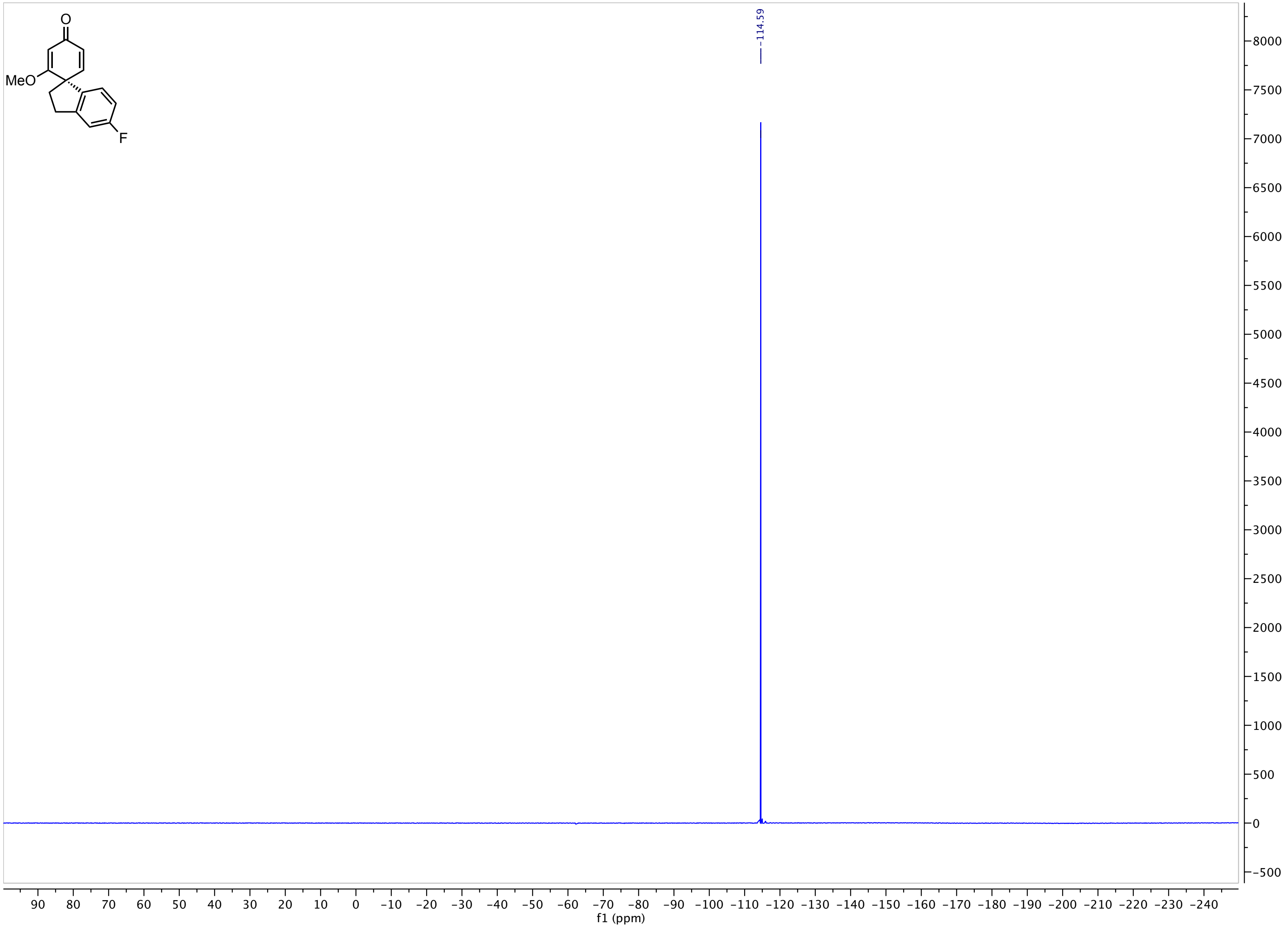

^{1}H NMR (CDCl$_3$): (*R*)-5'-Fluoro-2-methoxy-2',3'-dihydrospiro[cyclohexane-1,1'-indene]-2,5-dien-4-one **(2r)**

1.0 mmol

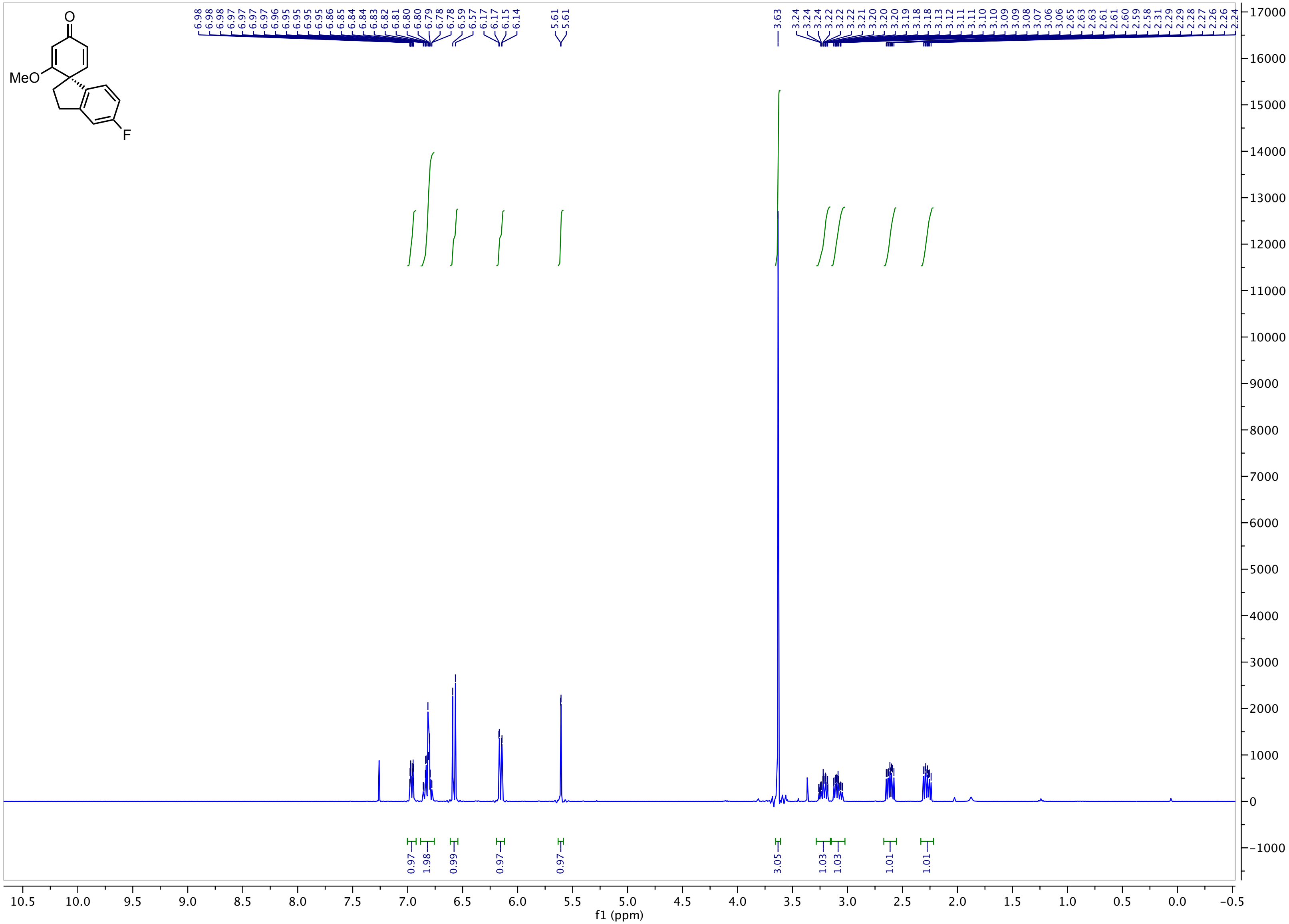

^{13}C NMR ($CDCl_3$): (*R*)-5’-Fluoro-2-methoxy-2',3'-dihydrospiro[cyclohexane-1,1'-indene]-2,5-dien-4-one **(2r)**

1.0 mmol

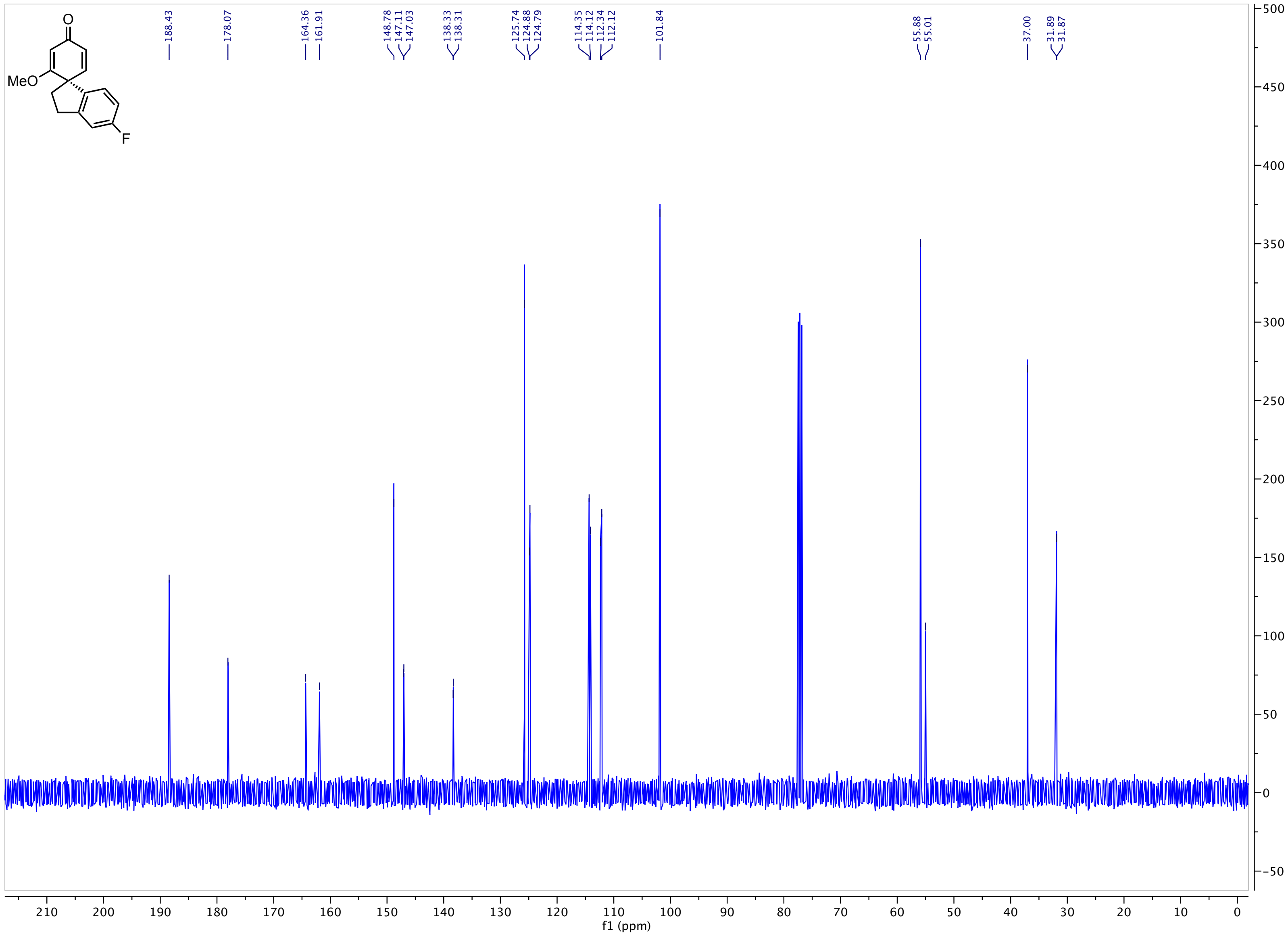

^{19}F NMR ($CDCl_3$): (*R*)-5'-Fluoro-2-methoxy-2',3'-dihydrospiro[cyclohexane-1,1'-indene]-2,5-dien-4-one **(2r)**

1.0 mmol

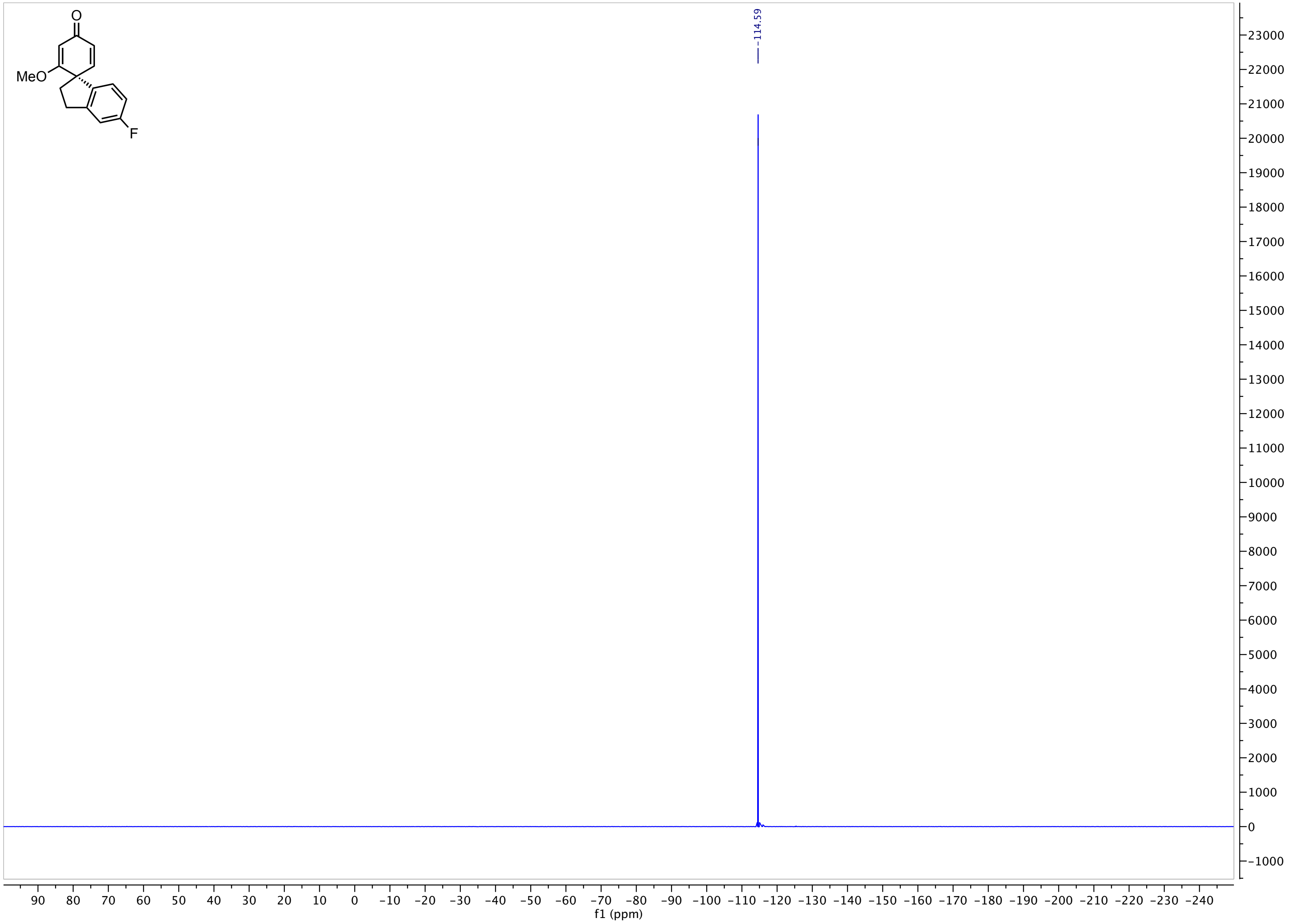

^{1}H NMR ($CDCl_3$): (*R*)-6’-Fluoro-2-methoxy-2',3'-dihydrospiro[cyclohexane-1,1'-indene]-2,5-dien-4-one **(2s)**

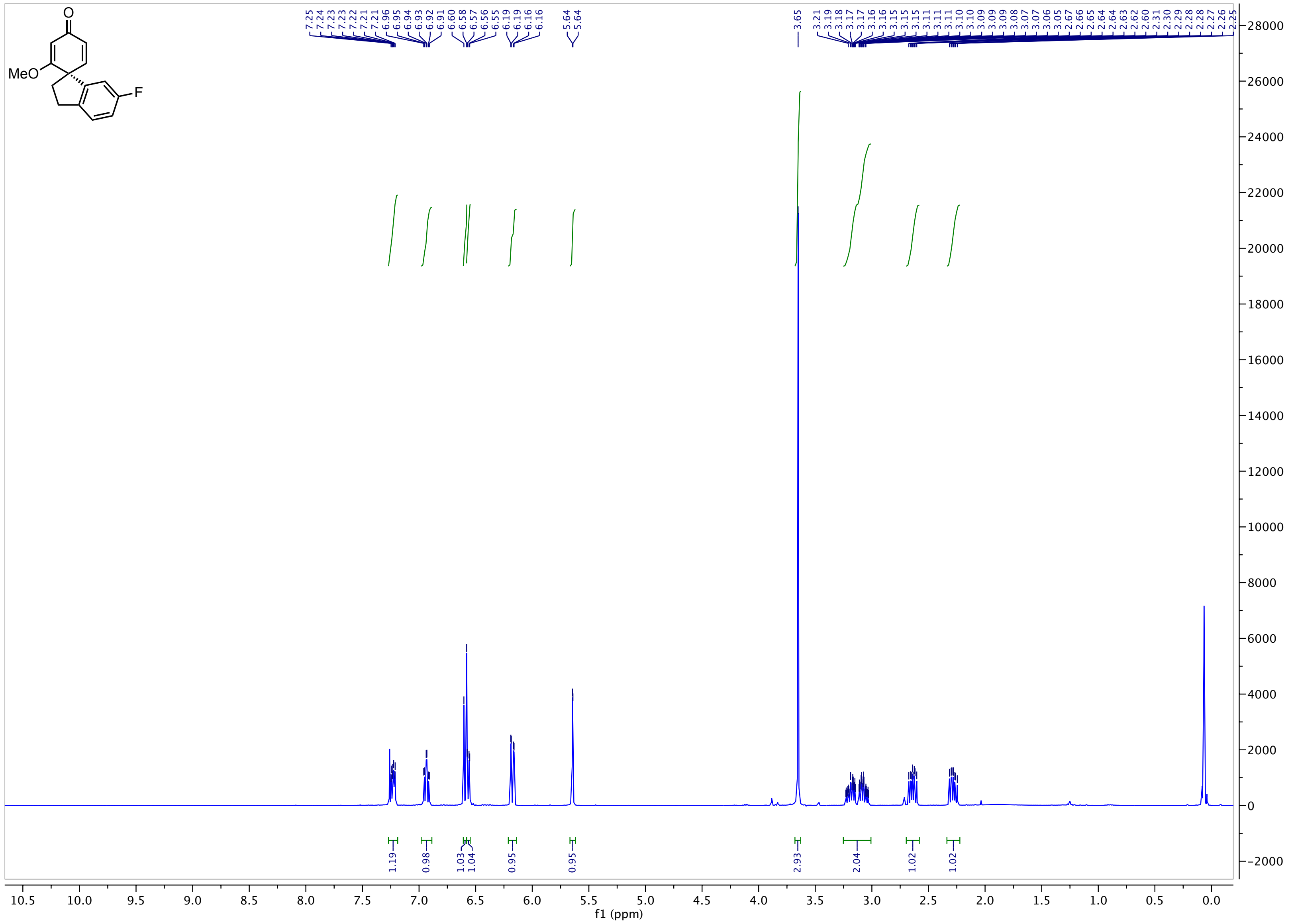

^{13}C NMR ($CDCl_3$): (*R*)-6’-Fluoro-2-methoxy-2',3'-dihydrospiro[cyclohexane-1,1'-indene]-2,5-dien-4-one **(2s)**

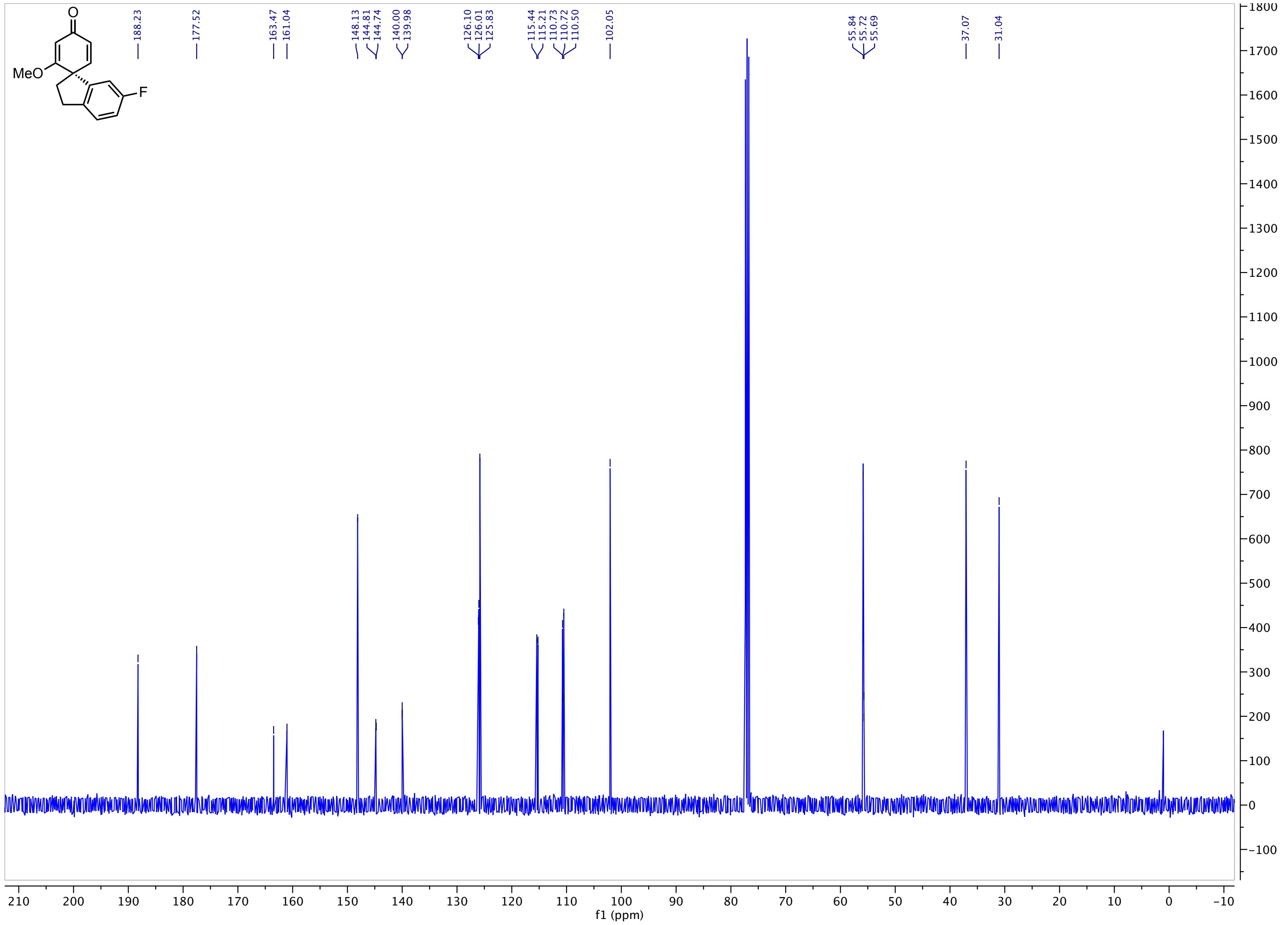

^{19}F NMR (CDCl$_3$): (*R*)-6'-Fluoro-2-methoxy-2',3'-dihydrospiro[cyclohexane-1,1'-indene]-2,5-dien-4-one **(2s)**

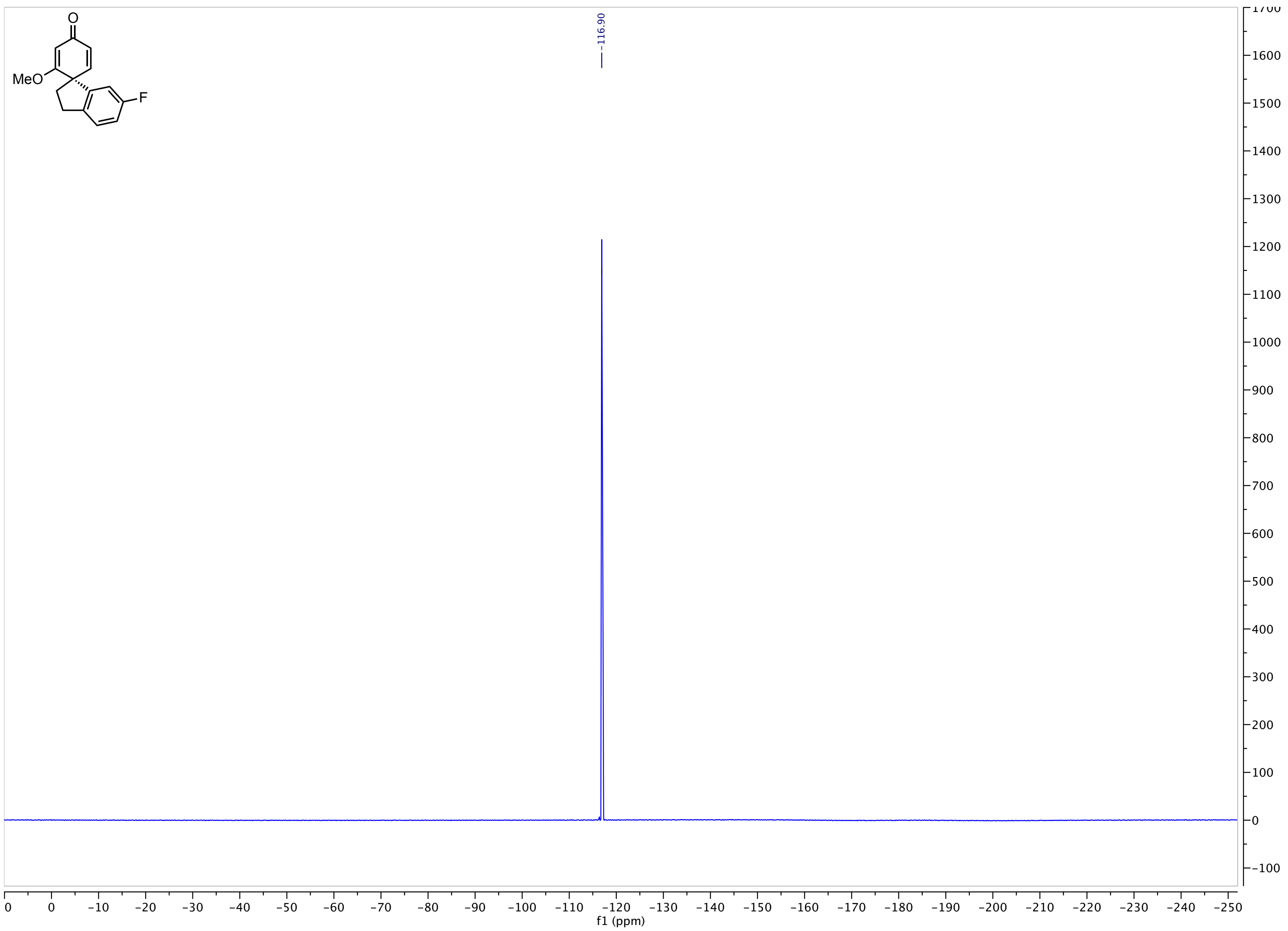

^{1}**H NMR** ($CDCl_3$): (*R*)-5,6,8,9-Tetrahydro-3*H*-indolo[7a,1-*a*]isoquinolin-3-one **(4a)**

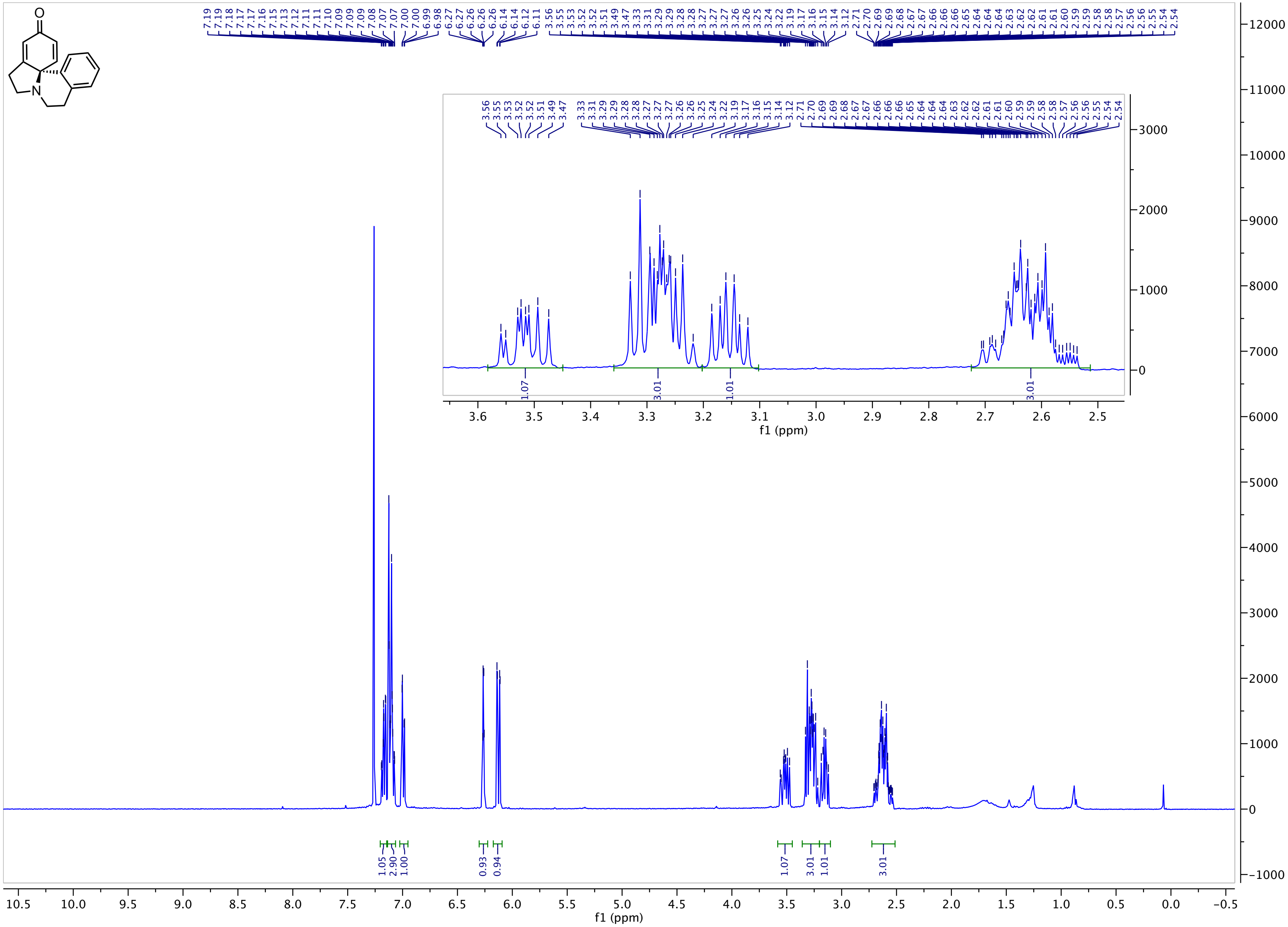

^{13}C NMR (CDCl$_3$): (*R*)-5,6,8,9-Tetrahydro-3*H*-indolo[7a,1-*a*]isoquinolin-3-one **(4a)**

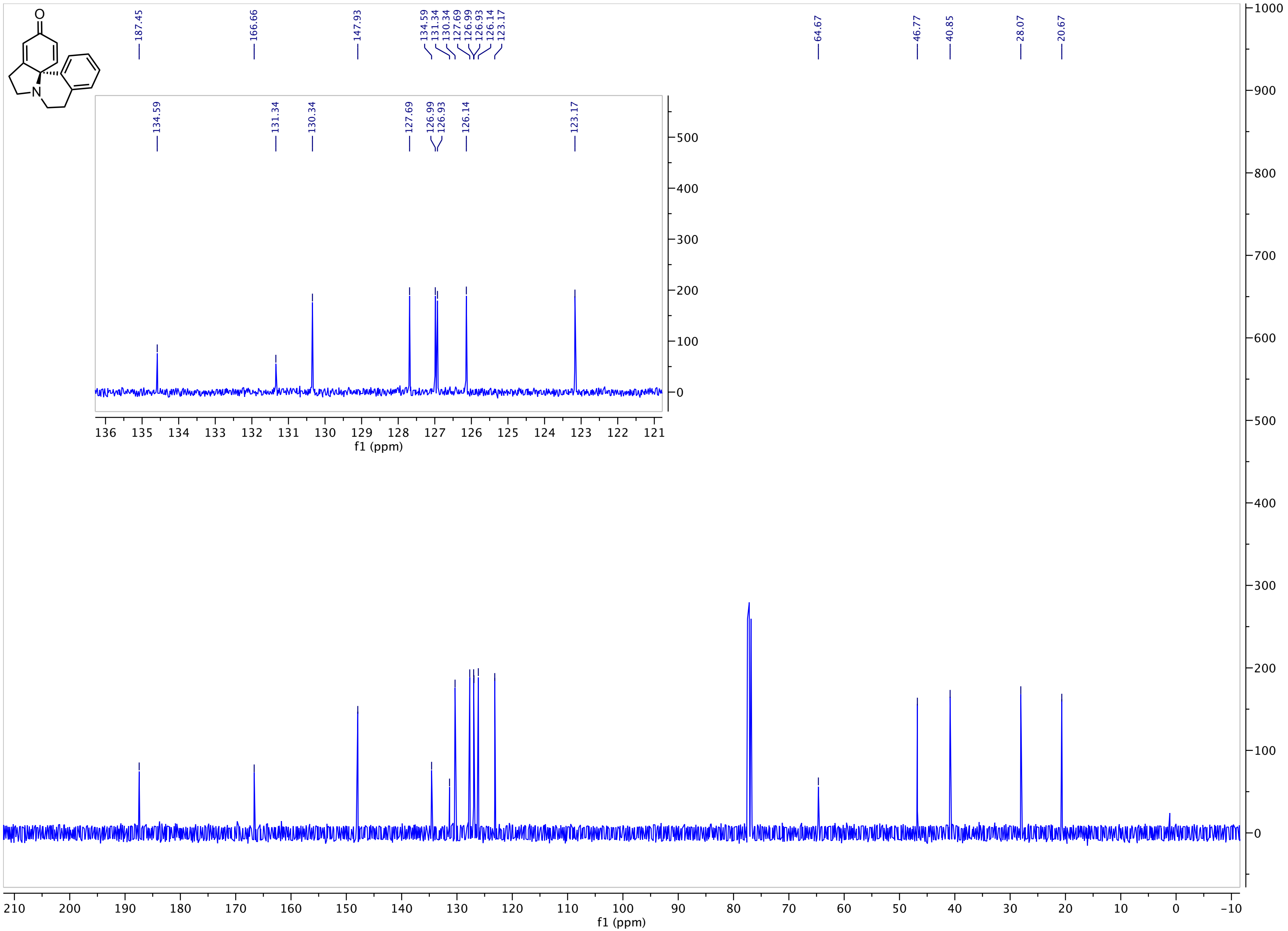

^{1}H NMR ($CDCl_3$): (*R*)-6,7,9,10-Tetrahydroisoquinolino[1,2-*j*]quinolin-3(5*H*)-one (**4b**)

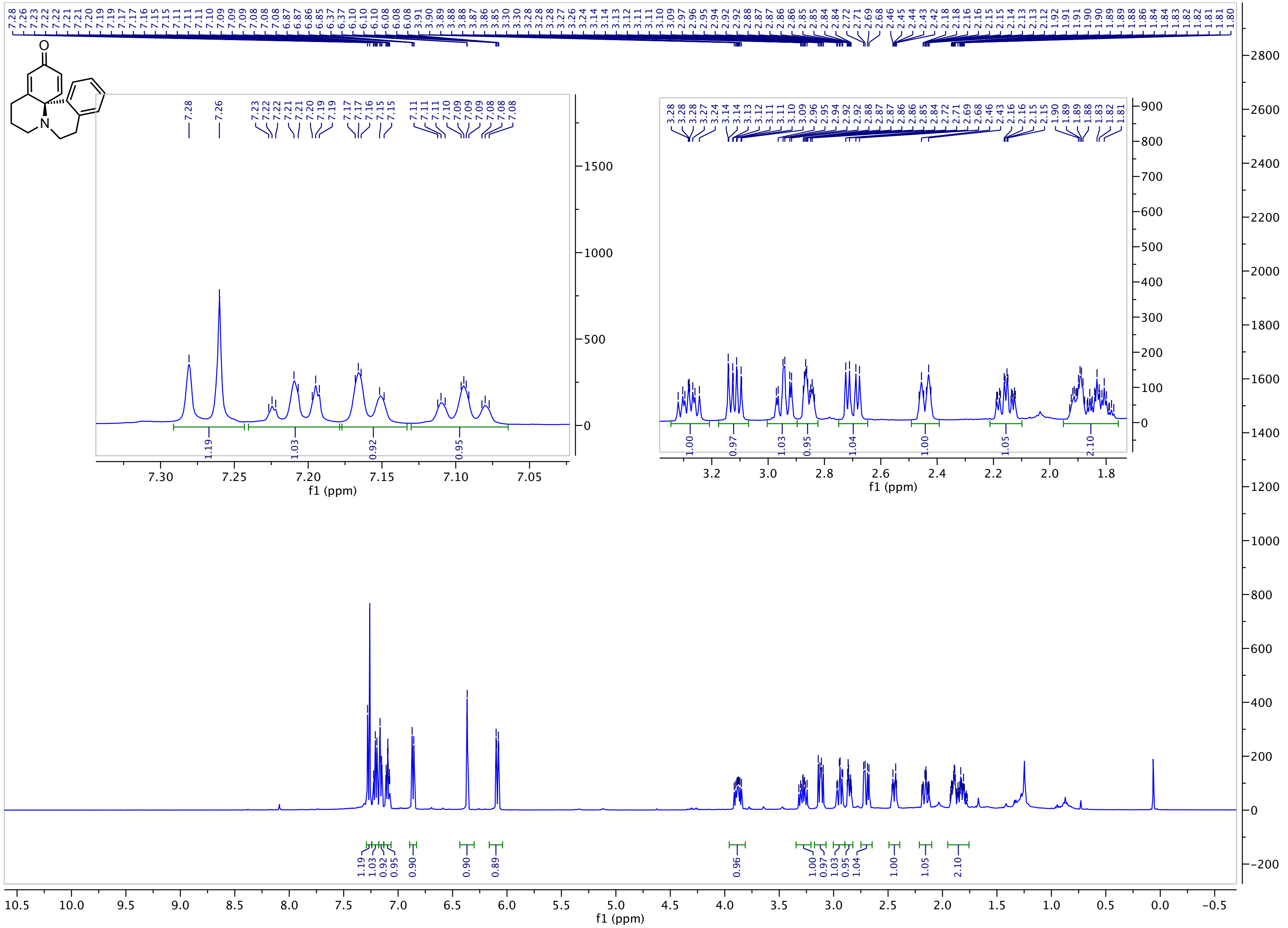

^{13}C NMR ($CDCl_3$): (*R*)-6,7,9,10-Tetrahydroisoquinolino[1,2-*j*]quinolin-3(5*H*)-one **(4b)**

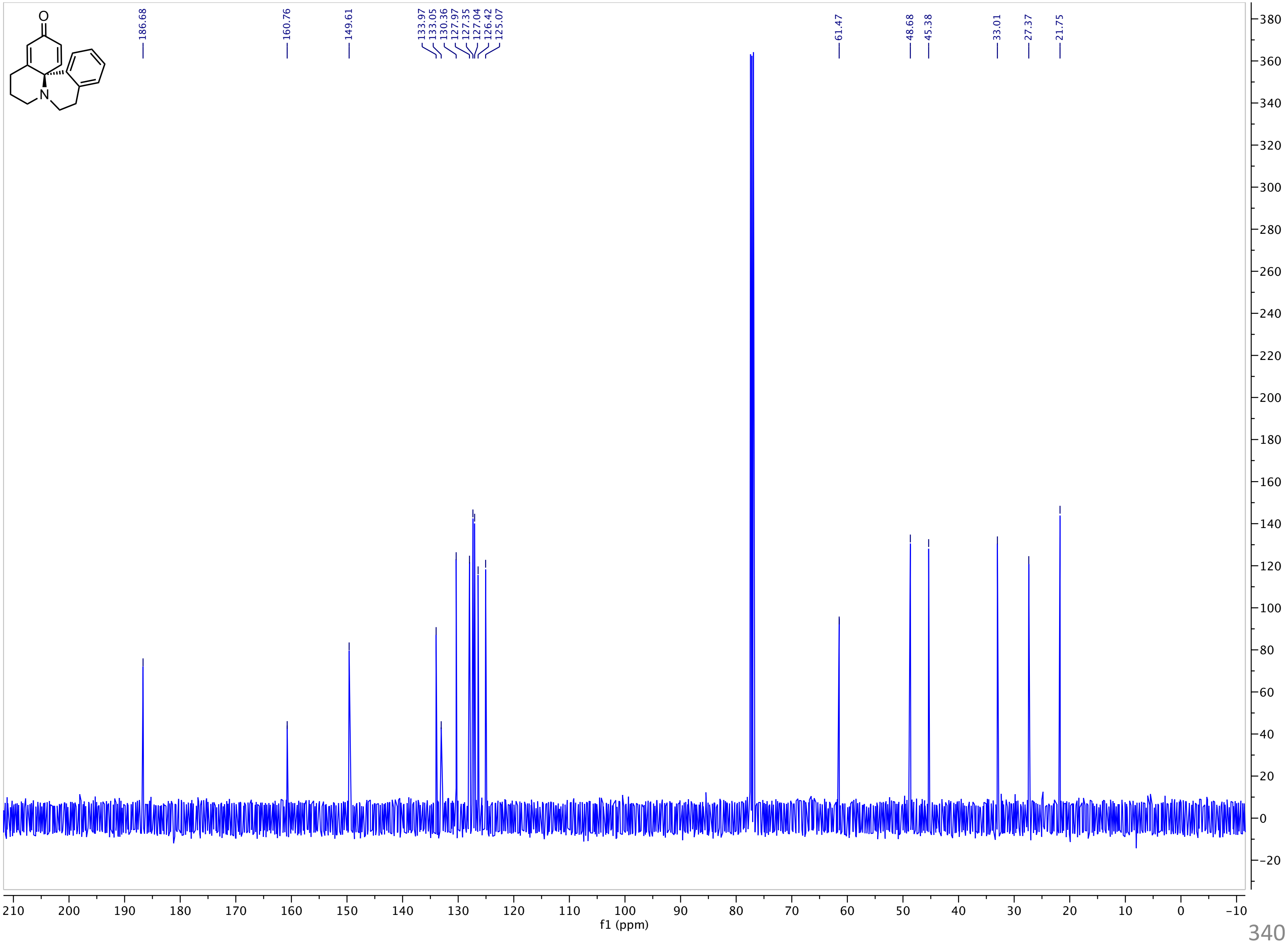

^{1}H NMR ($CDCl_3$): (*R*)-11-Methoxy-5,6,8,9-tetrahydro-3*H*-indolo[7a,1-*a*]isoquinolin-3-one **(4c)**

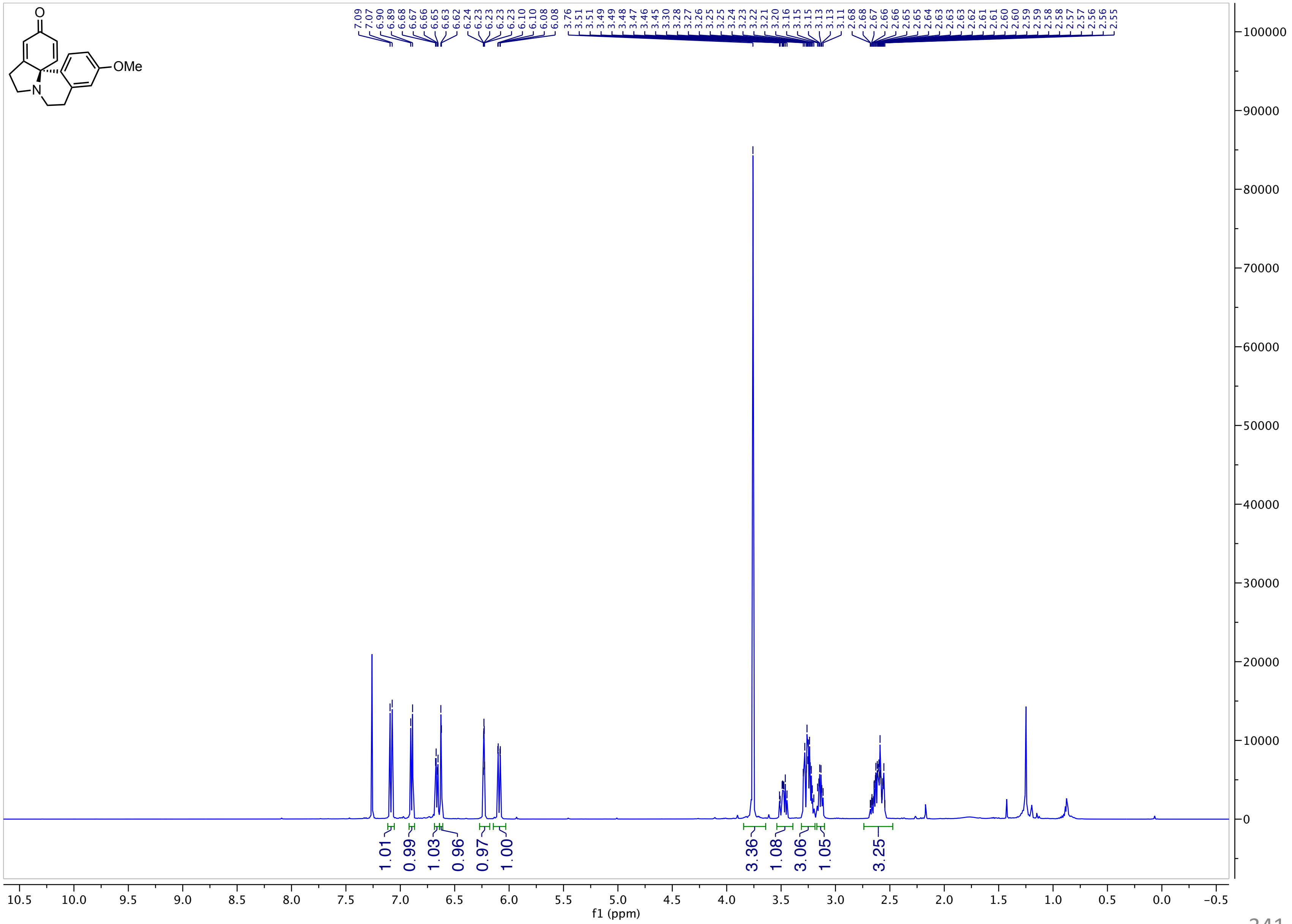

^{13}C NMR (CDCl$_3$): (*R*)-11-Methoxy-5,6,8,9-tetrahydro-3*H*-indolo[7a,1-*a*]isoquinolin-3-one **(4c)**

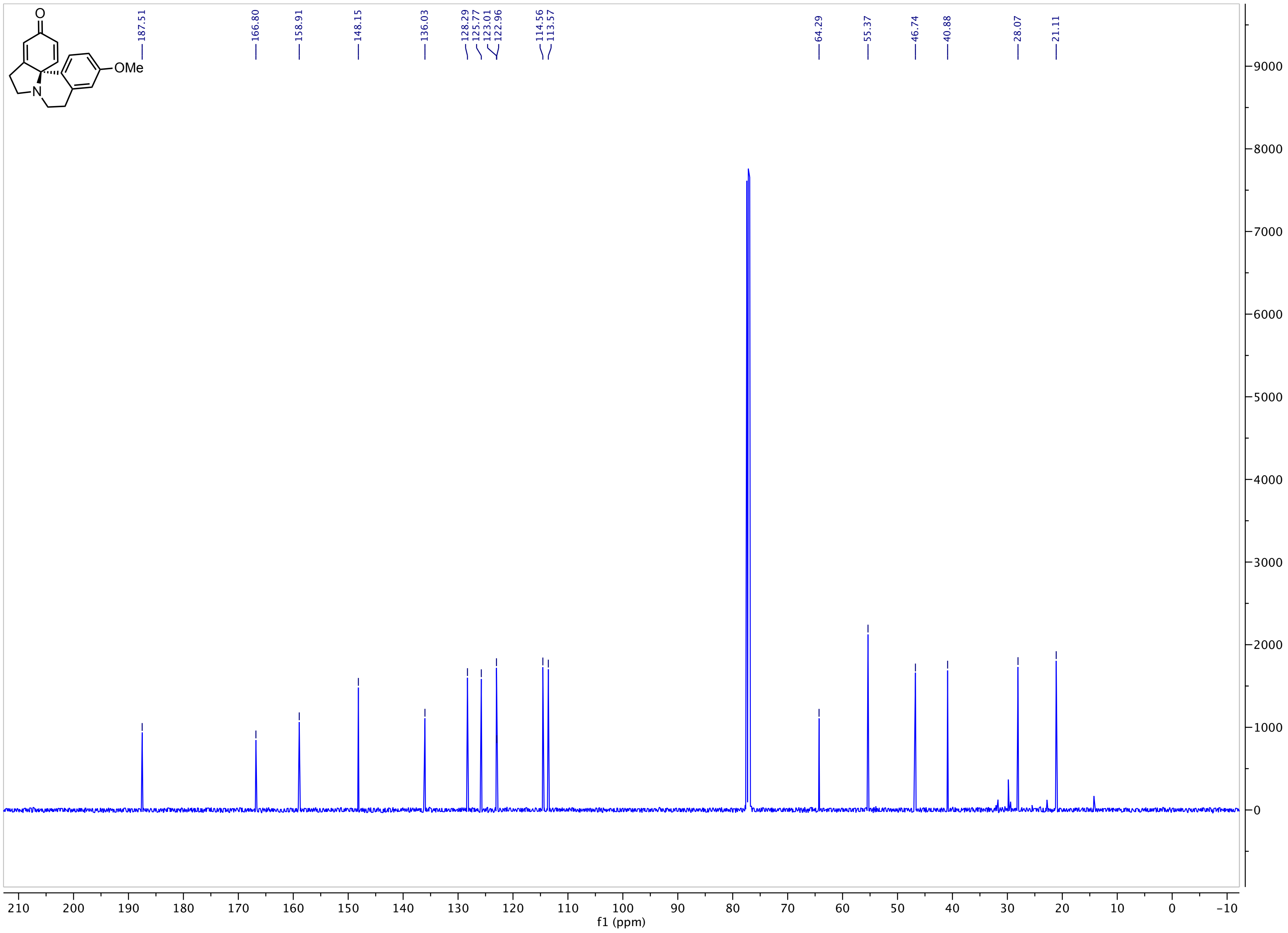

^{1}H NMR ($CDCl_3$): (*R*)-11-Chloro-5,6,8,9-tetrahydro-3*H*-indolo[7a,1-*a*]isoquinolin-3-one **(4d)**

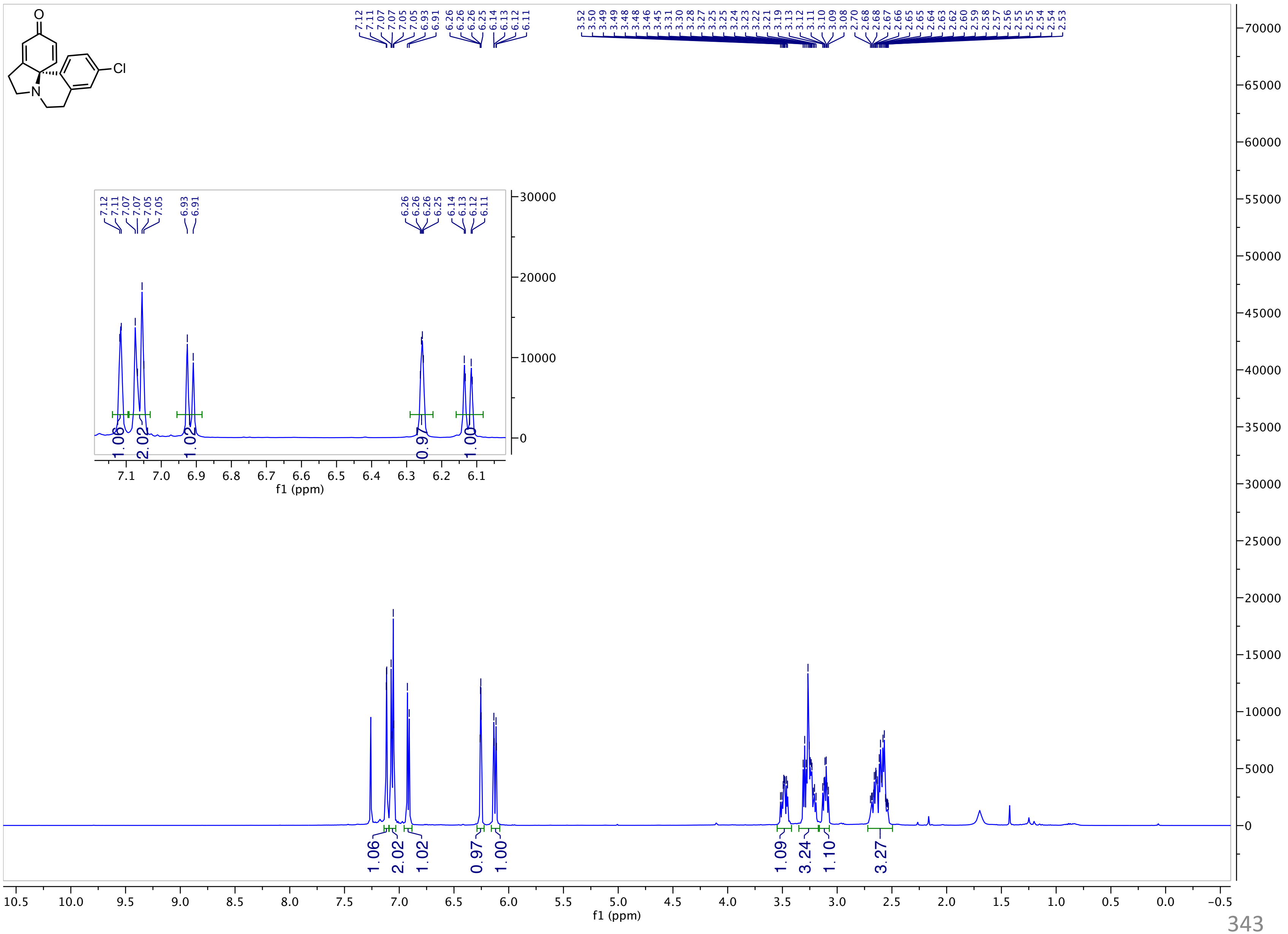

^{13}C NMR ($CDCl_3$): (R)-11-Chloro-5,6,8,9-tetrahydro-3H-indolo[7a,1-a]isoquinolin-3-one **(4d)**

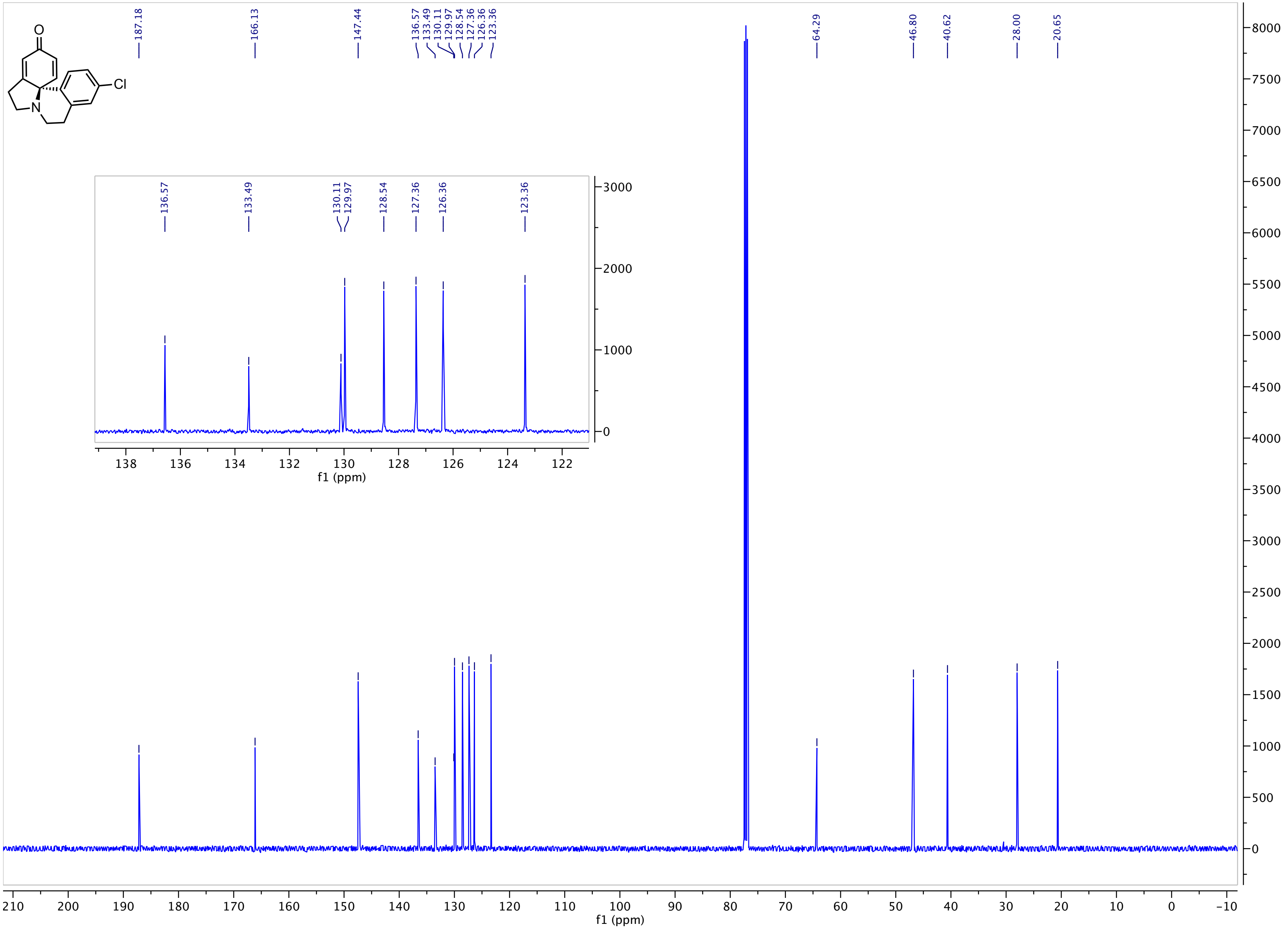

^{1}H NMR (CDCl$_3$): (*R*)-11-(trifluoromethyl)-5,6,8,9-Tetrahydro-3*H*-indolo[7a,1-*a*]isoquinolin-3-one **(4e)**

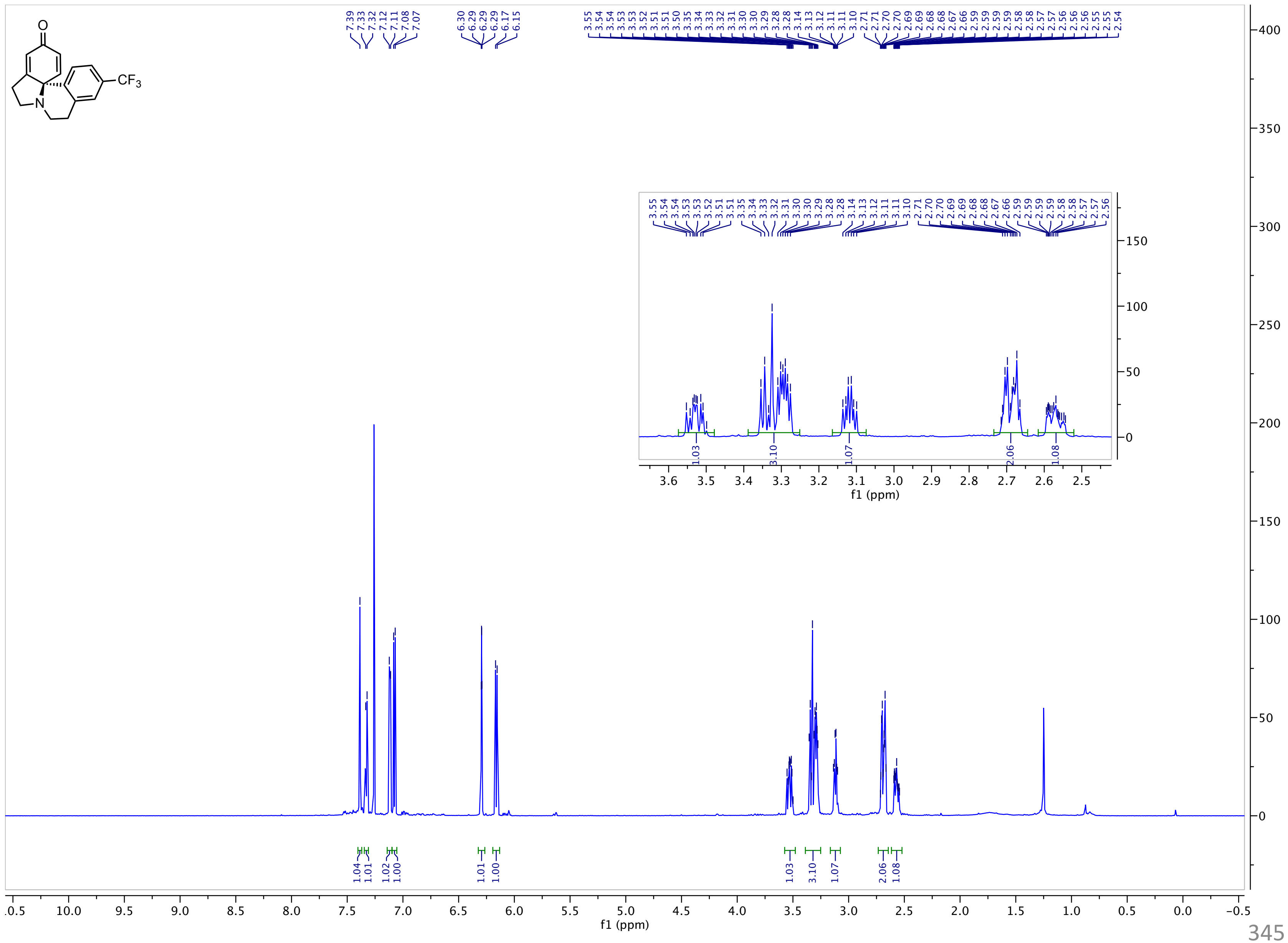

^{13}C NMR (CDCl$_3$): (*R*)-11-(trifluoromethyl)-5,6,8,9-Tetrahydro-3*H*-indolo[7a,1-*a*]isoquinolin-3-one **(4e)**

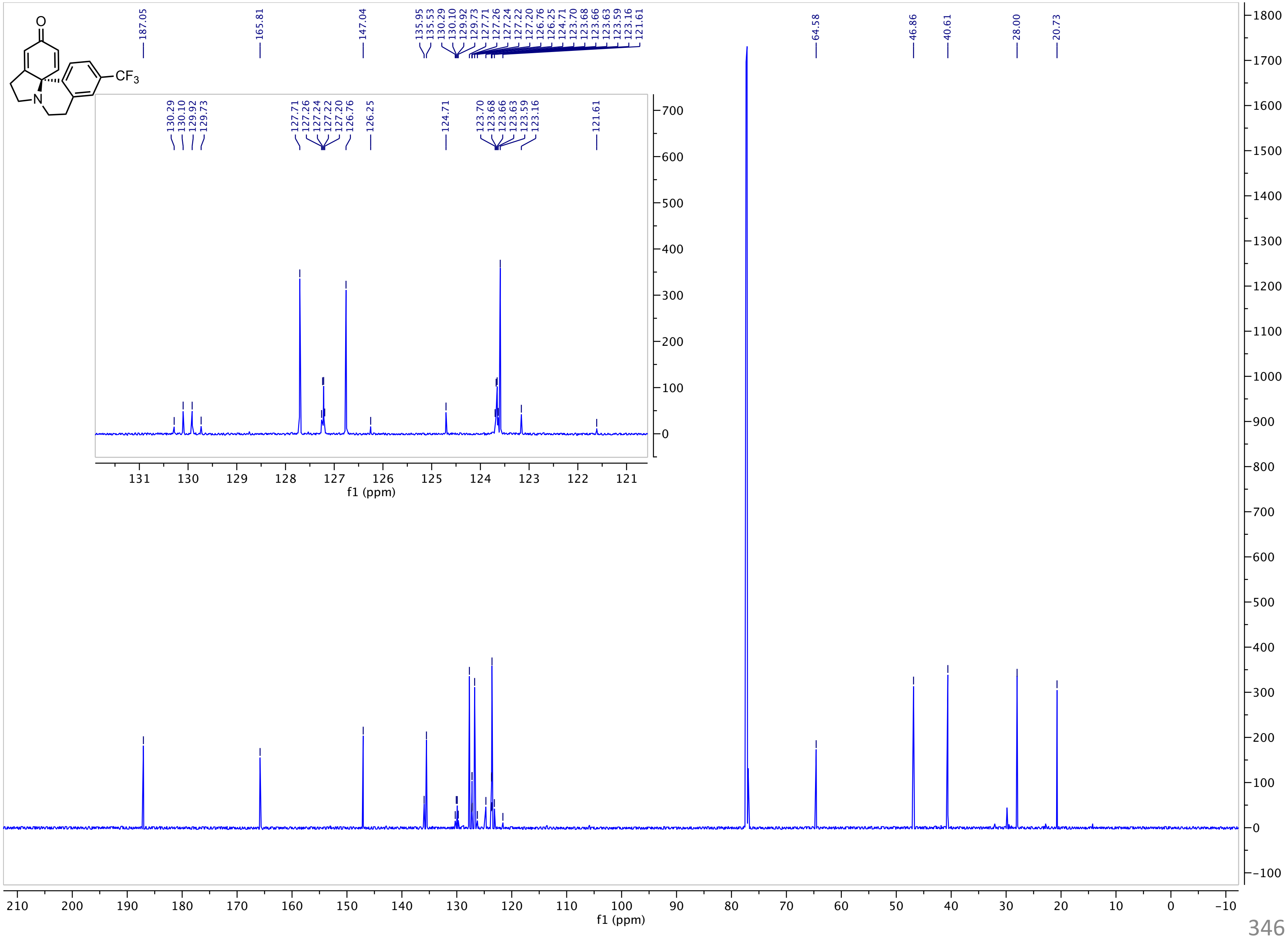

^{19}F NMR ($CDCl_3$): (*R*)-11-(trifluoromethyl)-5,6,8,9-Tetrahydro-3*H*-indolo[7a,1-*a*]isoquinolin-3-one **(4e)**

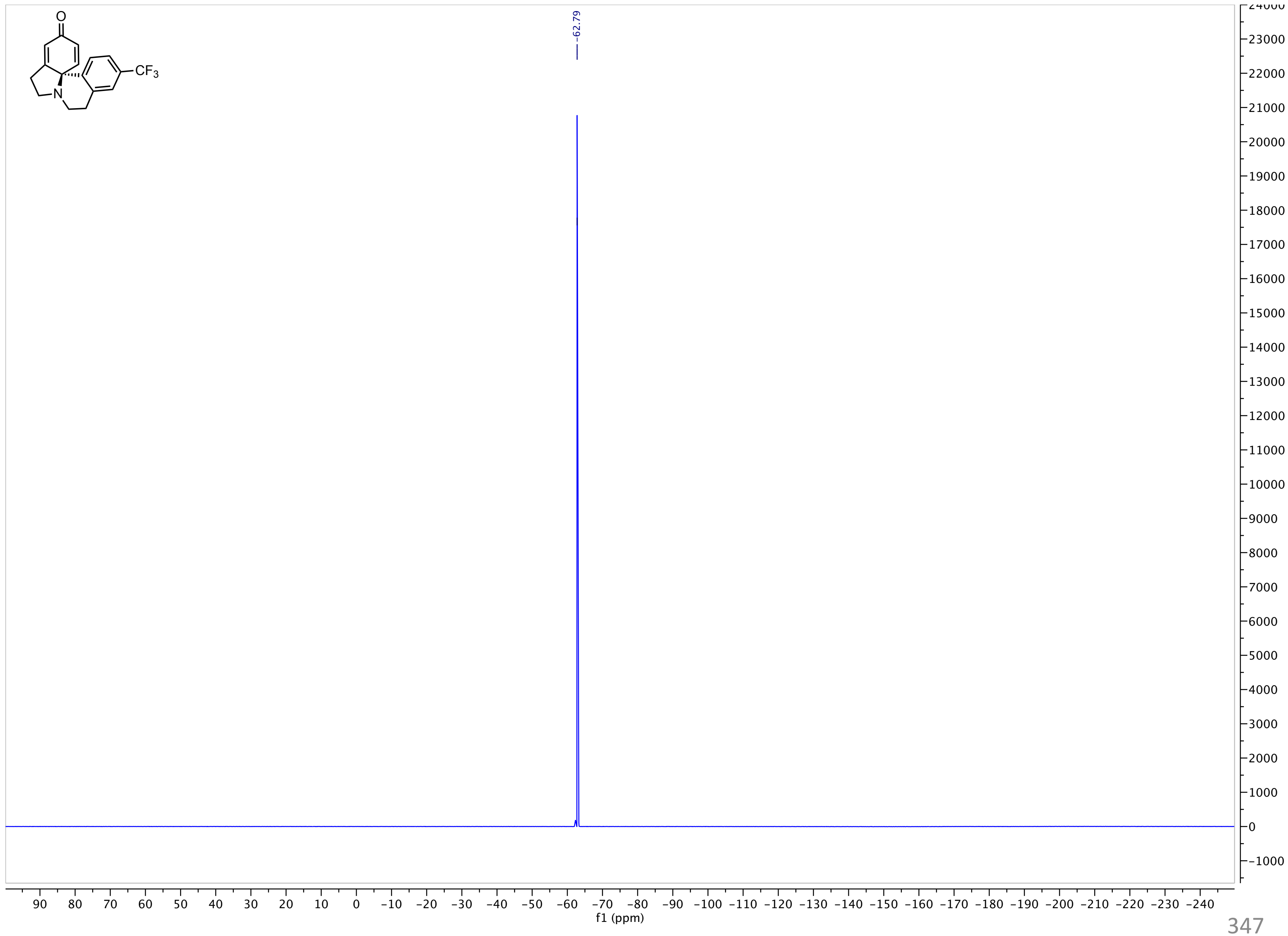

^{1}H NMR (CDCl$_3$): (*R*)-12,13-Dimethoxy-6,7,9,10-tetrahydroisoquinolino[1,2-*j*]quinolin-3(5*H*)-one **(4f)**

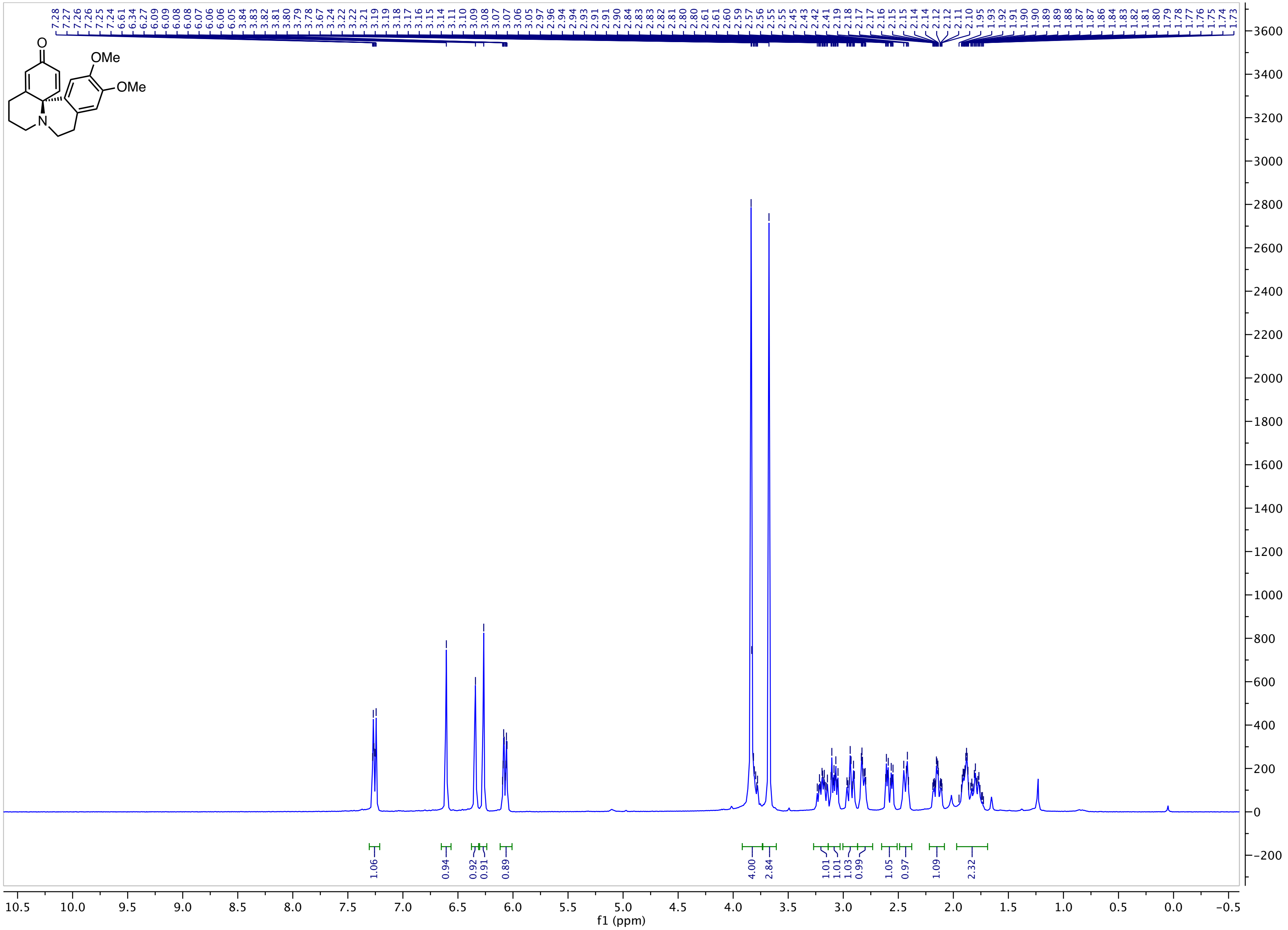

^{13}C NMR (CDCl$_3$): (*R*)-12,13-Dimethoxy-6,7,9,10-tetrahydroisoquinolino[1,2-*j*]quinolin-3(5*H*)-one **(4f)**

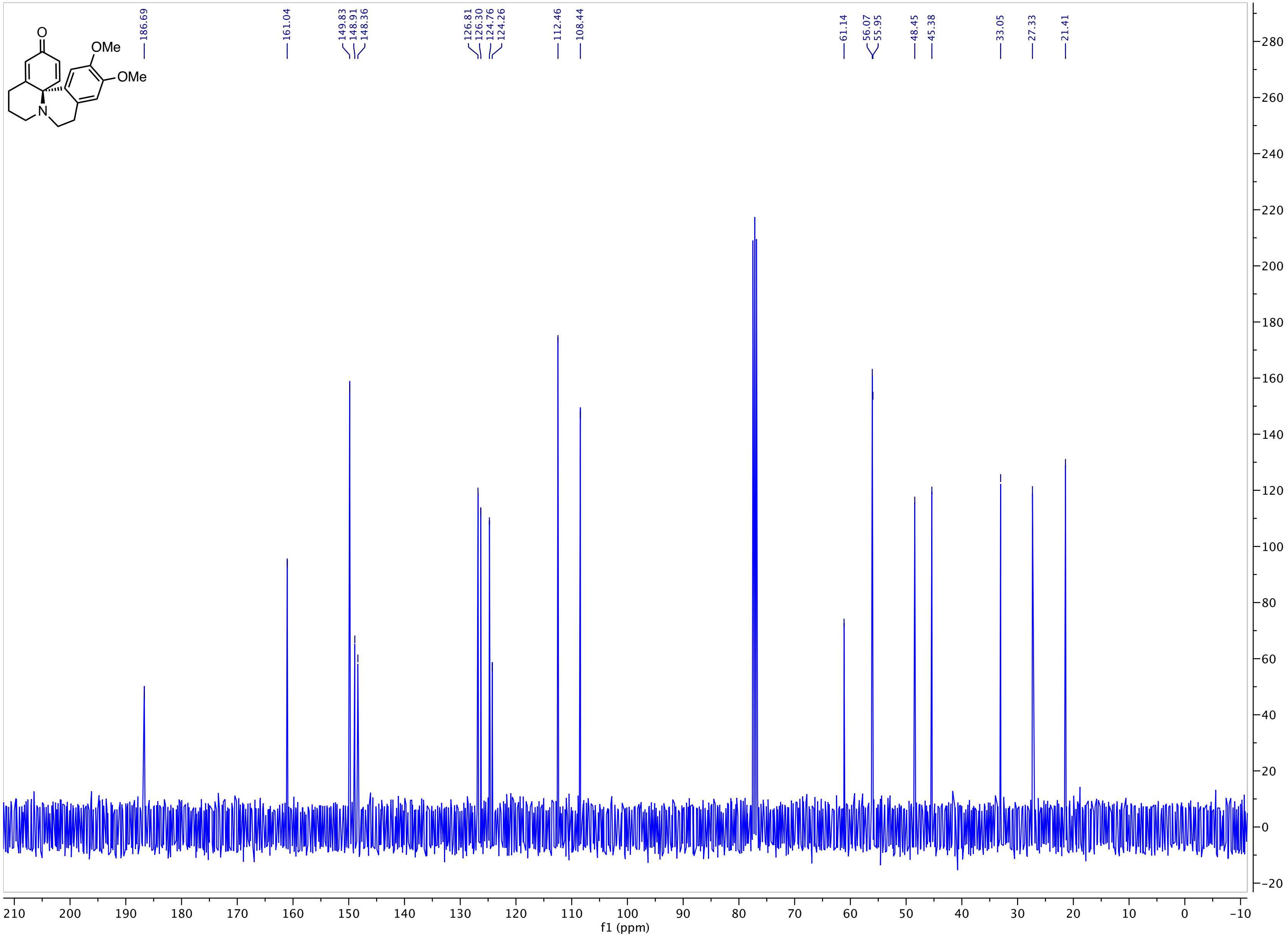

^{1}H NMR ($CDCl_3$): (*R*)-11,12-Dimethoxy-5,6,8,9-tetrahydro-3*H*-indolo[7a,1-*a*]isoquinolin-3-one **(4g)**

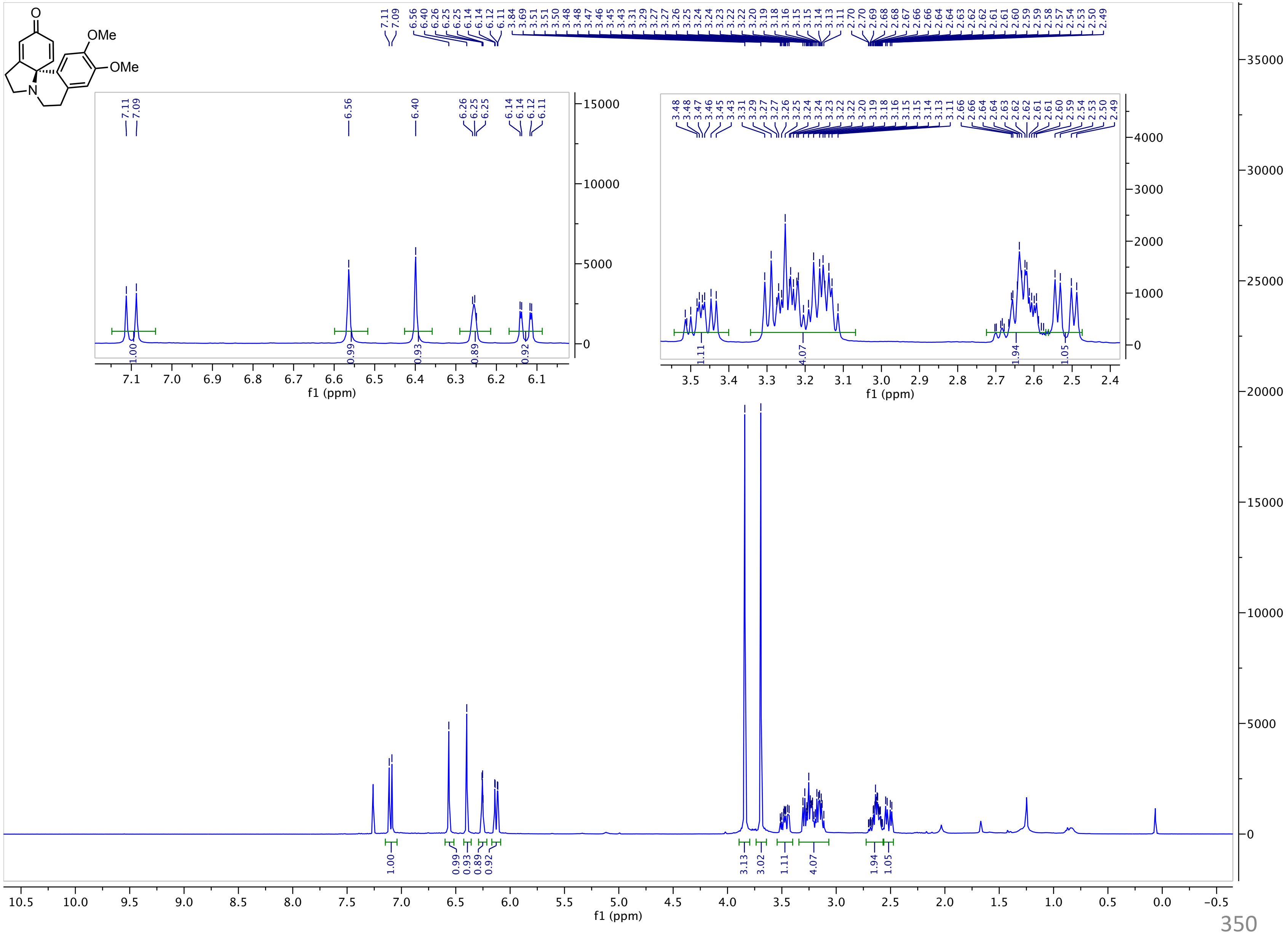

^{13}C NMR ($CDCl_3$): (*R*)-11,12-Dimethoxy-5,6,8,9-tetrahydro-3*H*-indolo[7a,1-*a*]isoquinolin-3-one **(4g)**

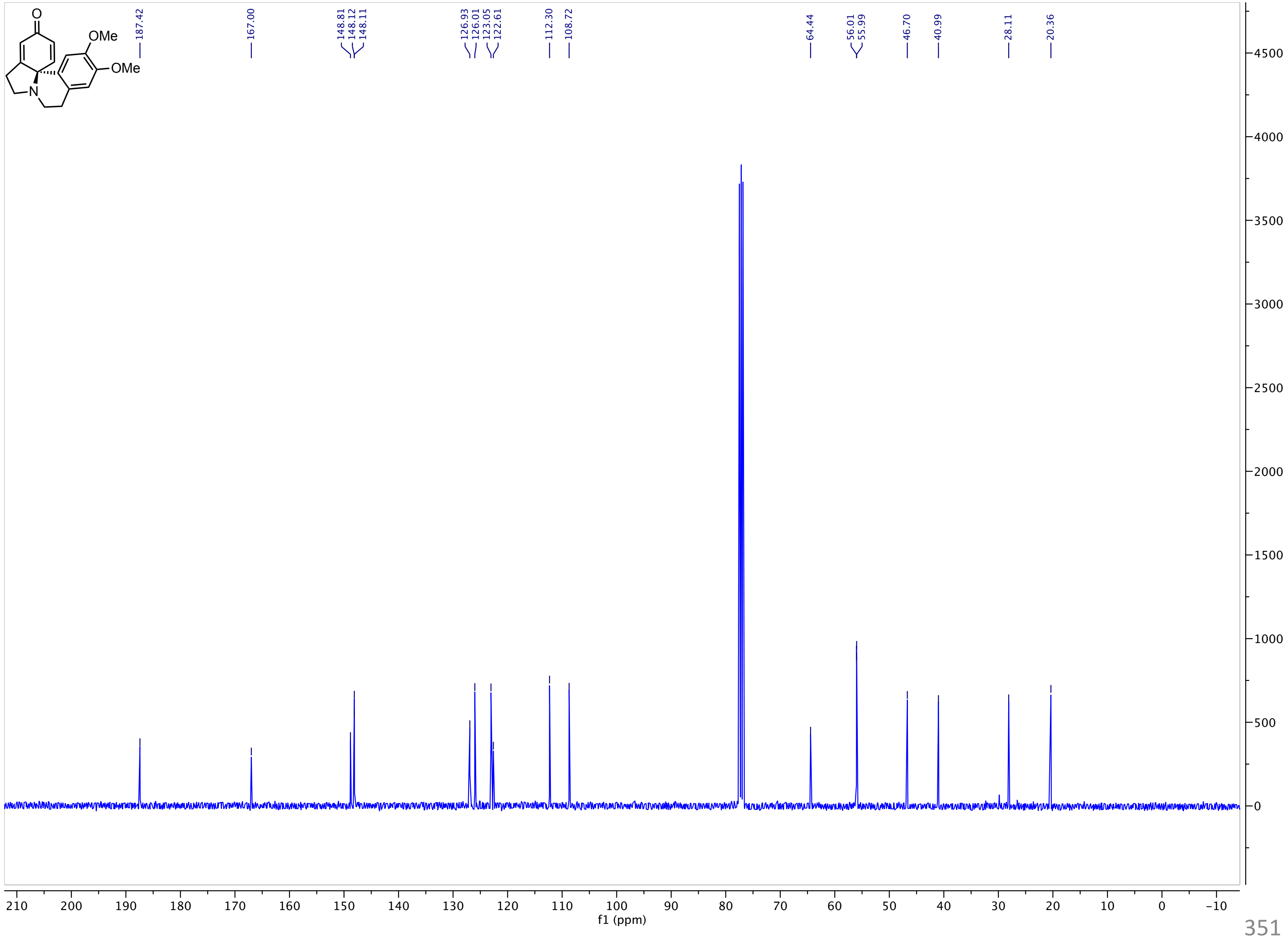

^{1}H NMR ($CDCl_3$): (*S*)-4a-Methyl-9,10-dihydrophenanthren-2(4a*H*)-one **(7a)**

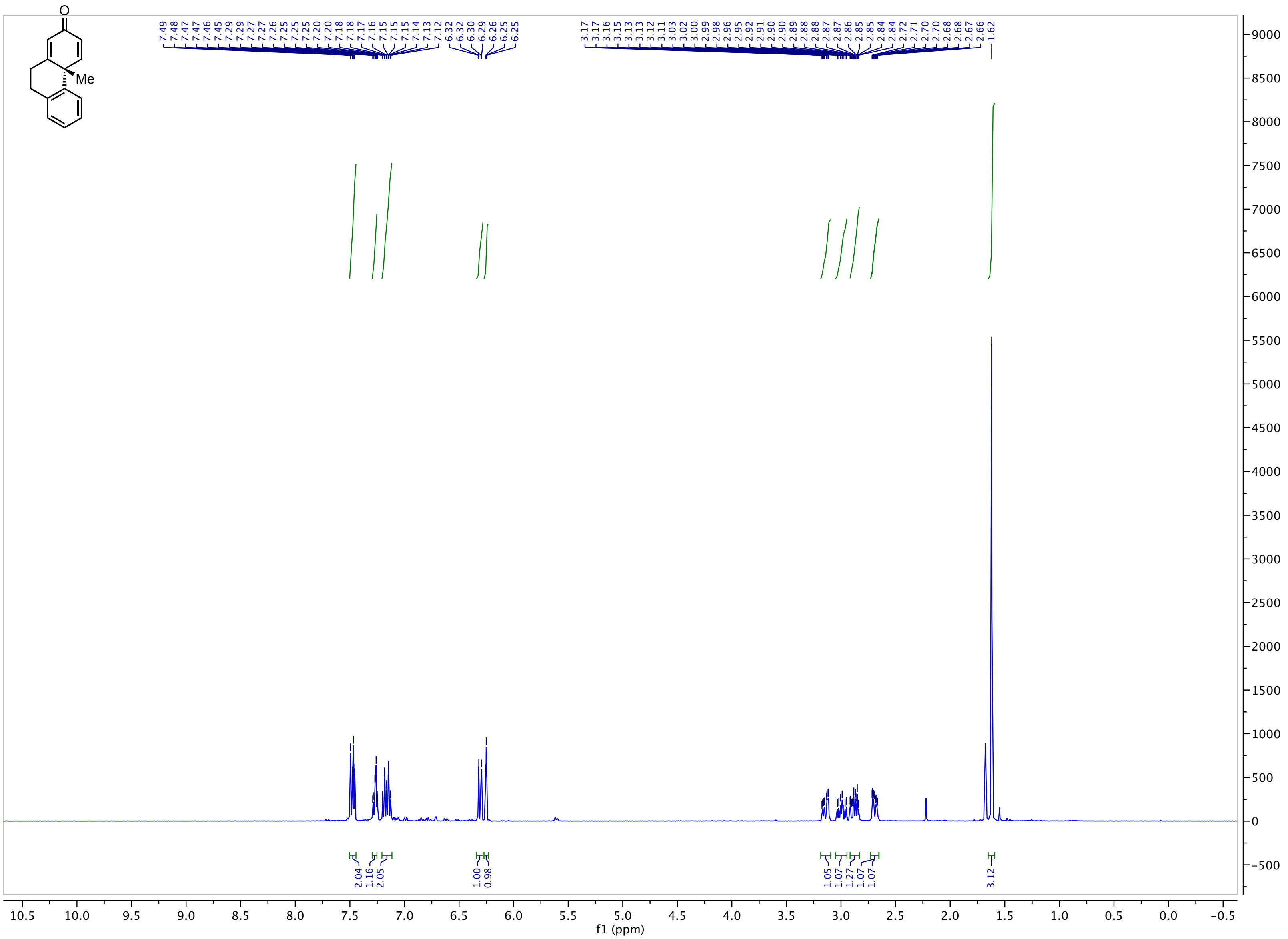

^{13}C NMR ($CDCl_3$): (*S*)-4a-Methyl-9,10-dihydrophenanthren-2(4a*H*)-one **(7a)**

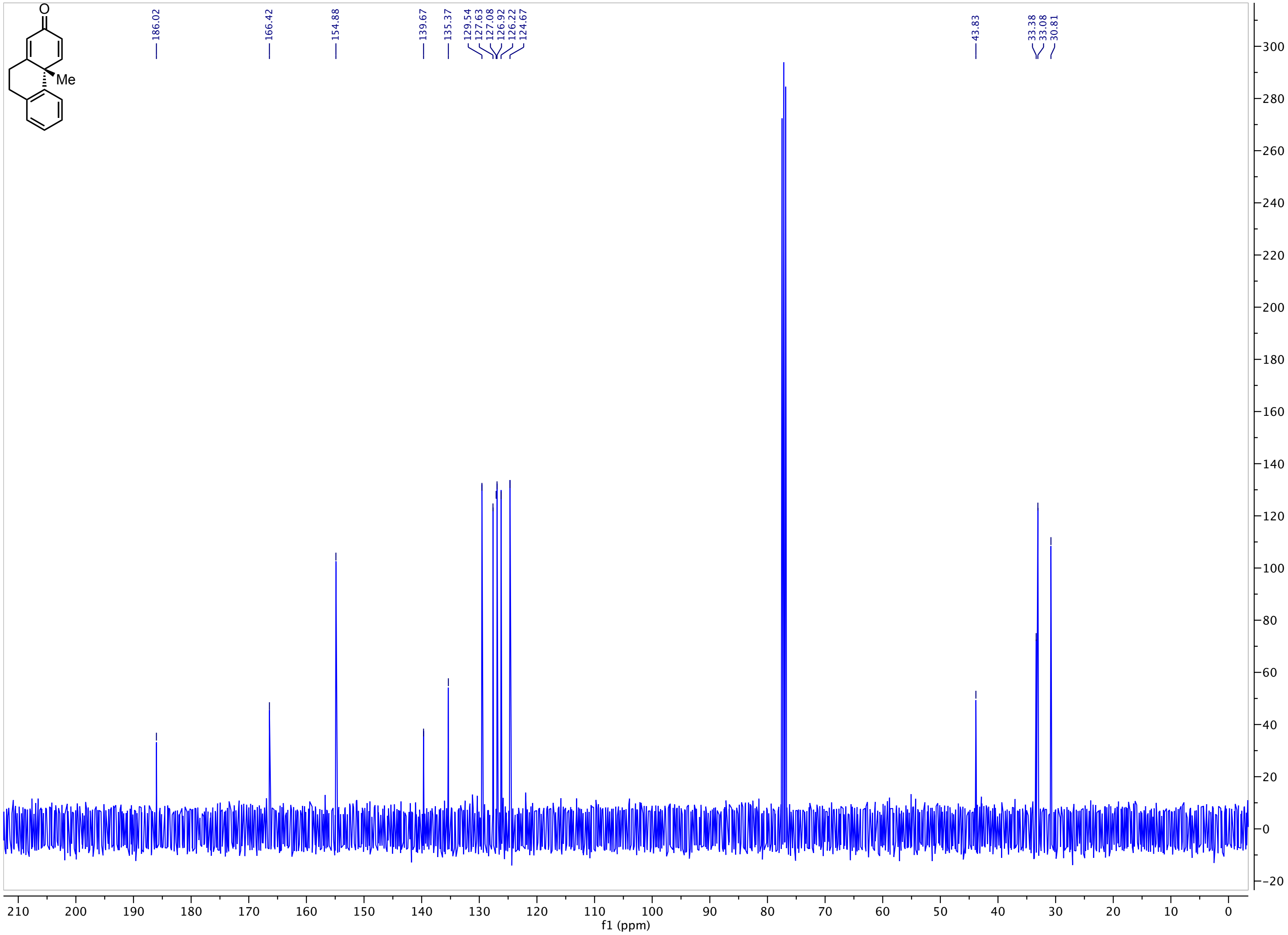

^{1}H NMR ($CDCl_3$): (*S*)-4a-Ethyl-9,10-dihydrophenanthren-2(4a*H*)-one **(7b)**

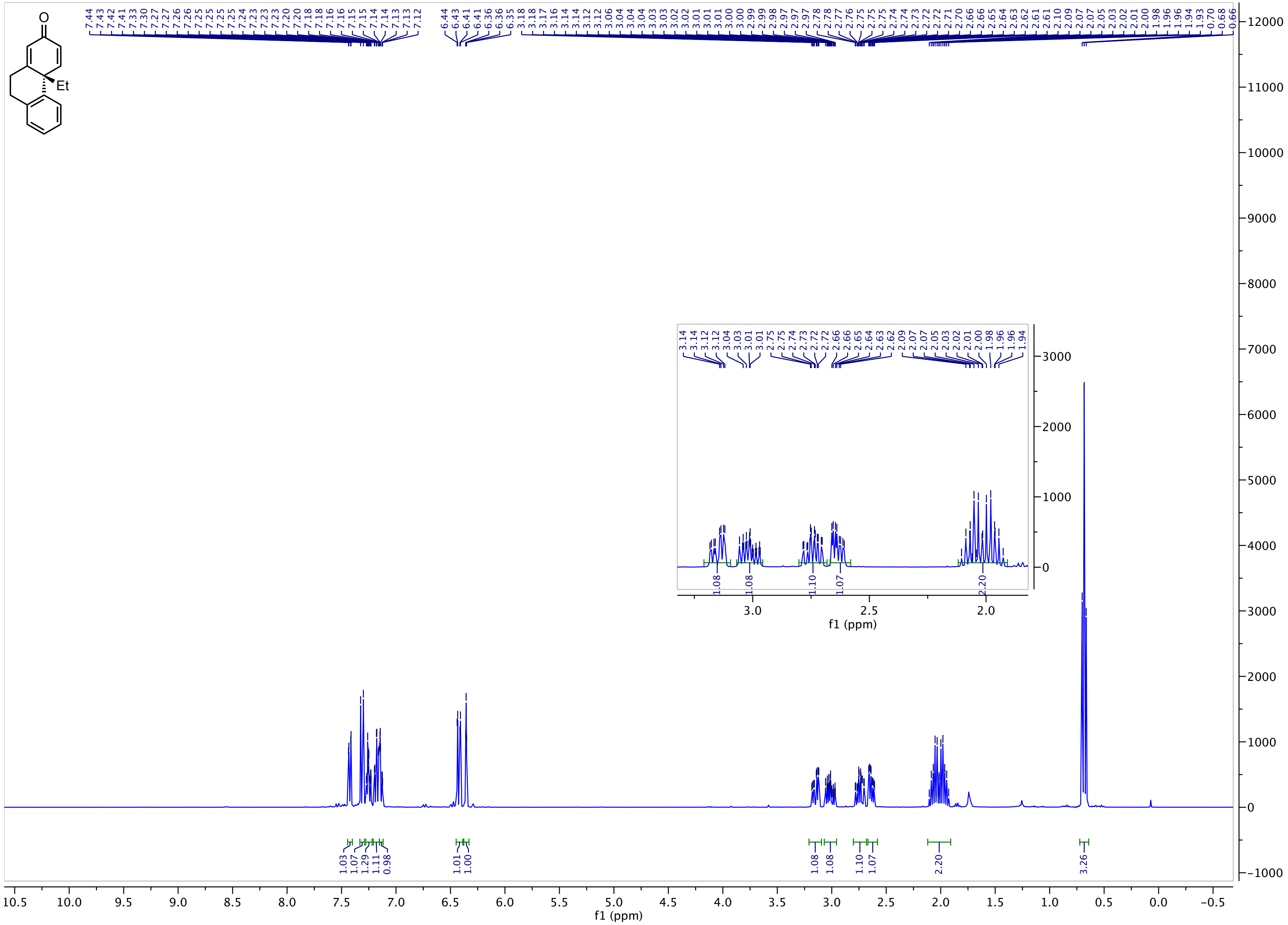

^{13}C NMR ($CDCl_3$): (*S*)-4a-Ethyl-9,10-dihydrophenanthren-2(4a*H*)-one **(7b)**

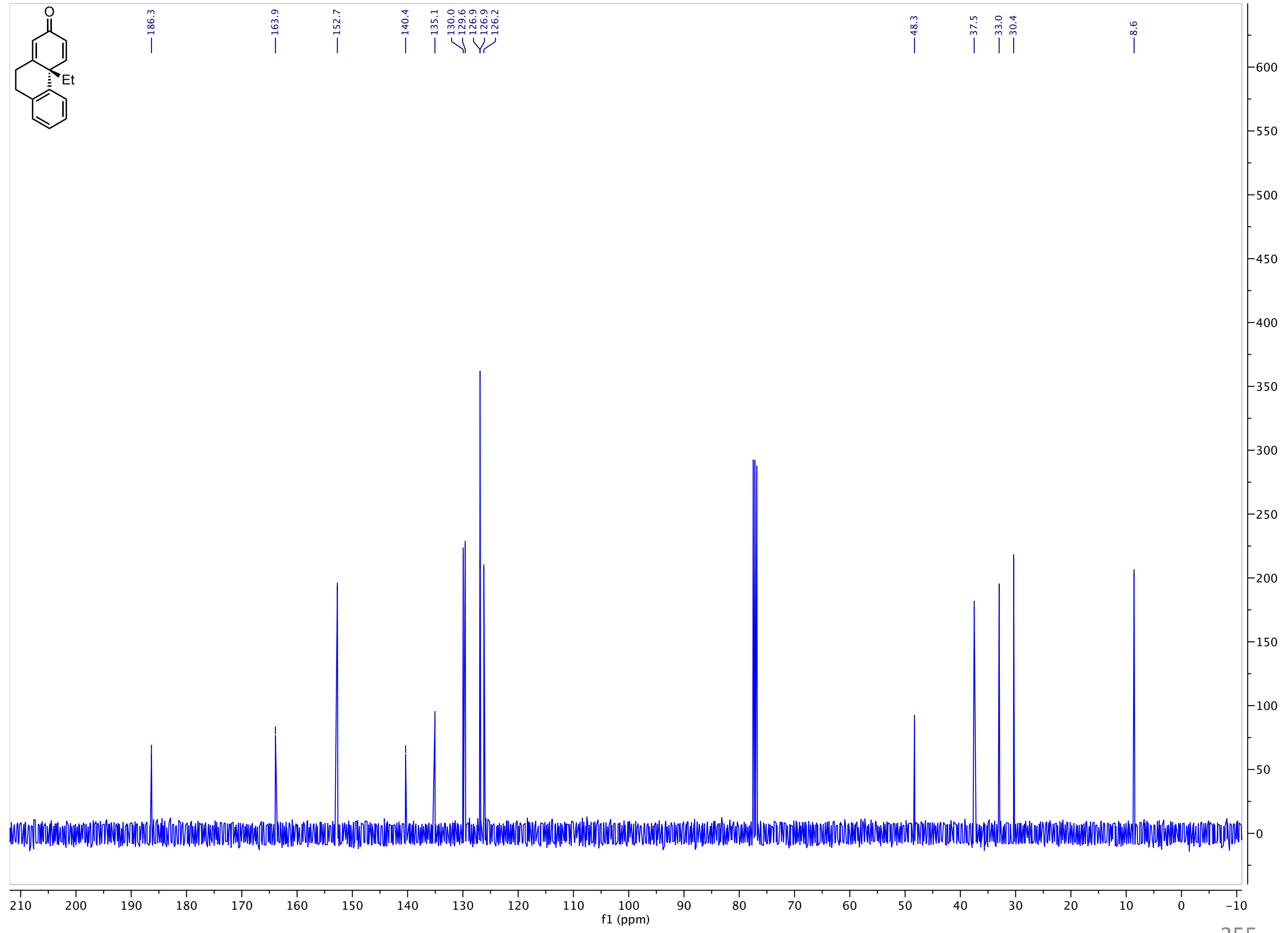

^{1}**H NMR** (CDCl$_3$): (*S*)-8-Chloro-4a-methyl-9,10-dihydrophenanthren-2(4a*H*)-one **(7c)**

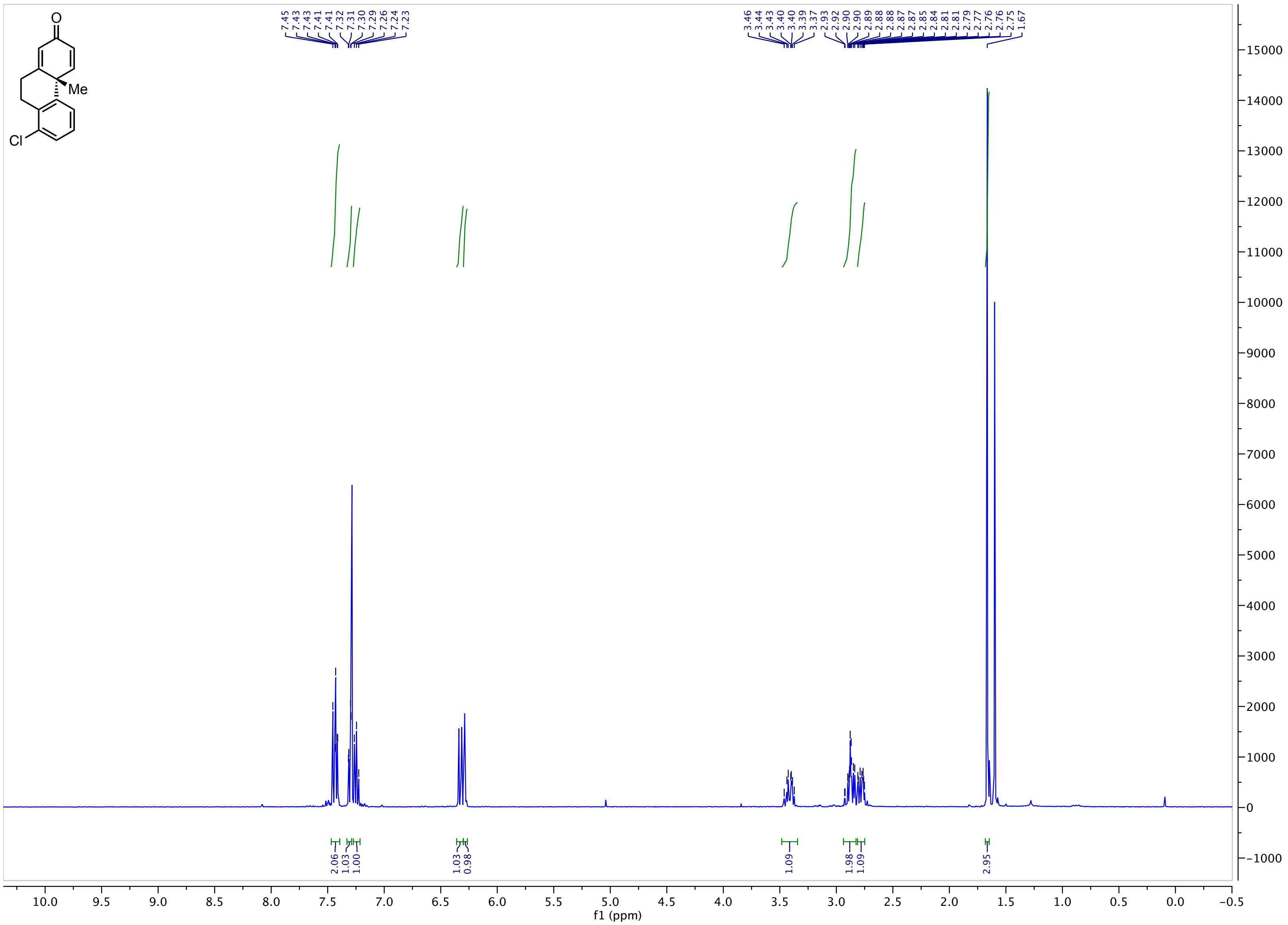

^{13}C NMR (CDCl$_3$): (*S*)-8-Chloro-4a-methyl-9,10-dihydrophenanthren-2(4a*H*)-one **(7c)**

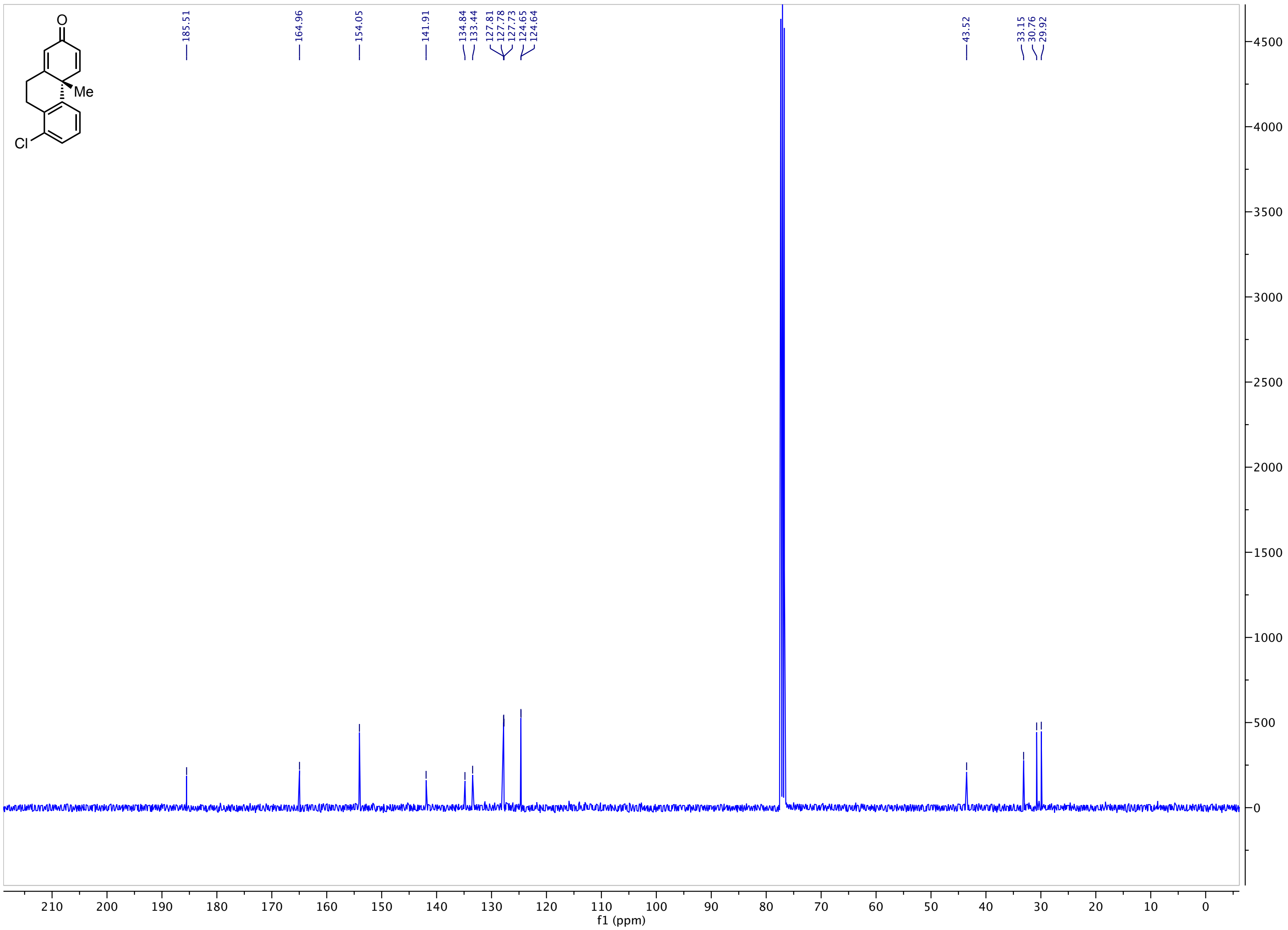

^{1}H NMR ($CDCl_3$): (*S*)-7-Fluoro-4a-methyl-9,10-dihydrophenanthren-2(4a*H*)-one **(7d)**

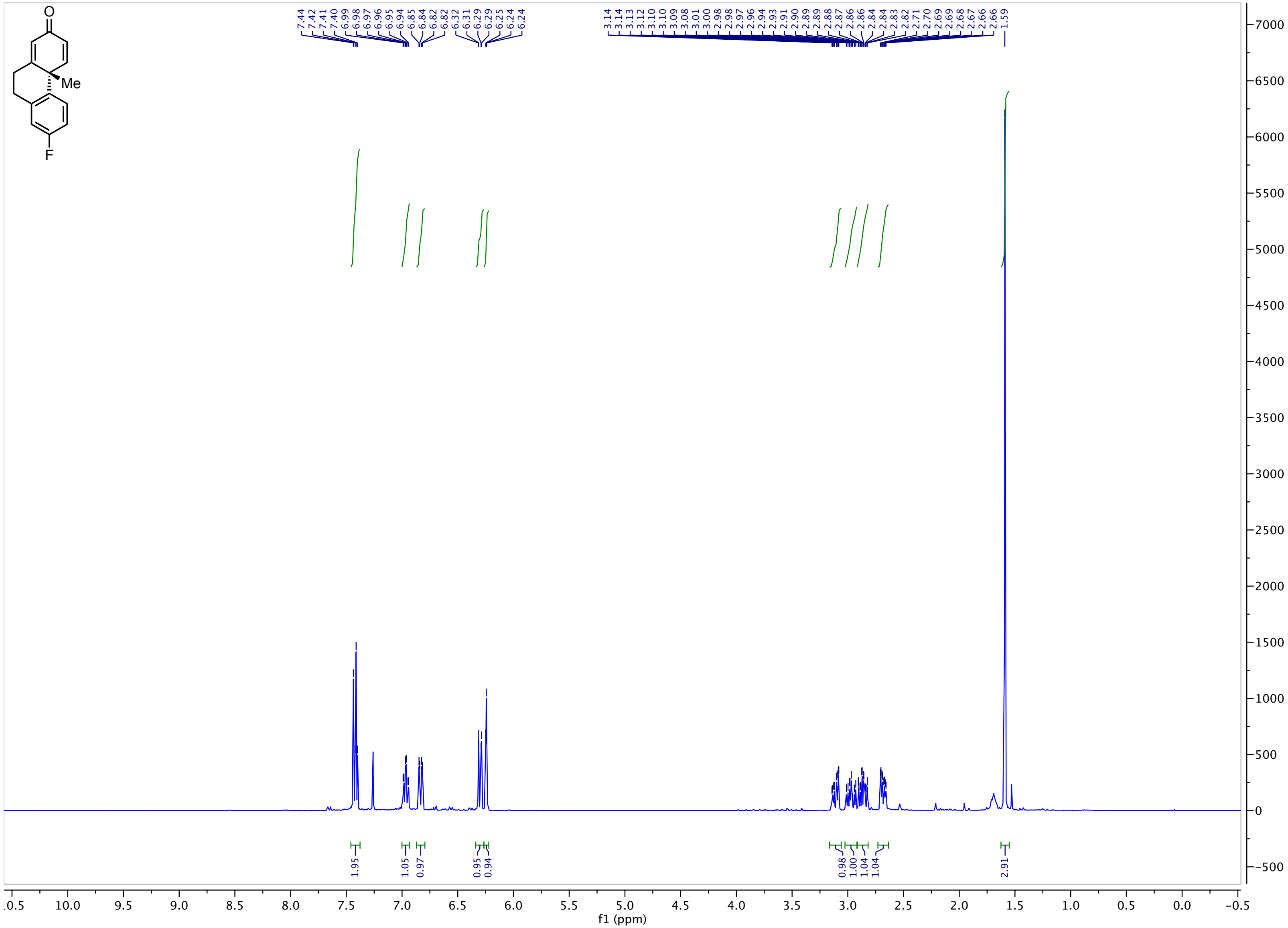

^{13}C NMR (CDCl$_3$): (*S*)-7-Fluoro-4a-methyl-9,10-dihydrophenanthren-2(4a*H*)-one (7d)

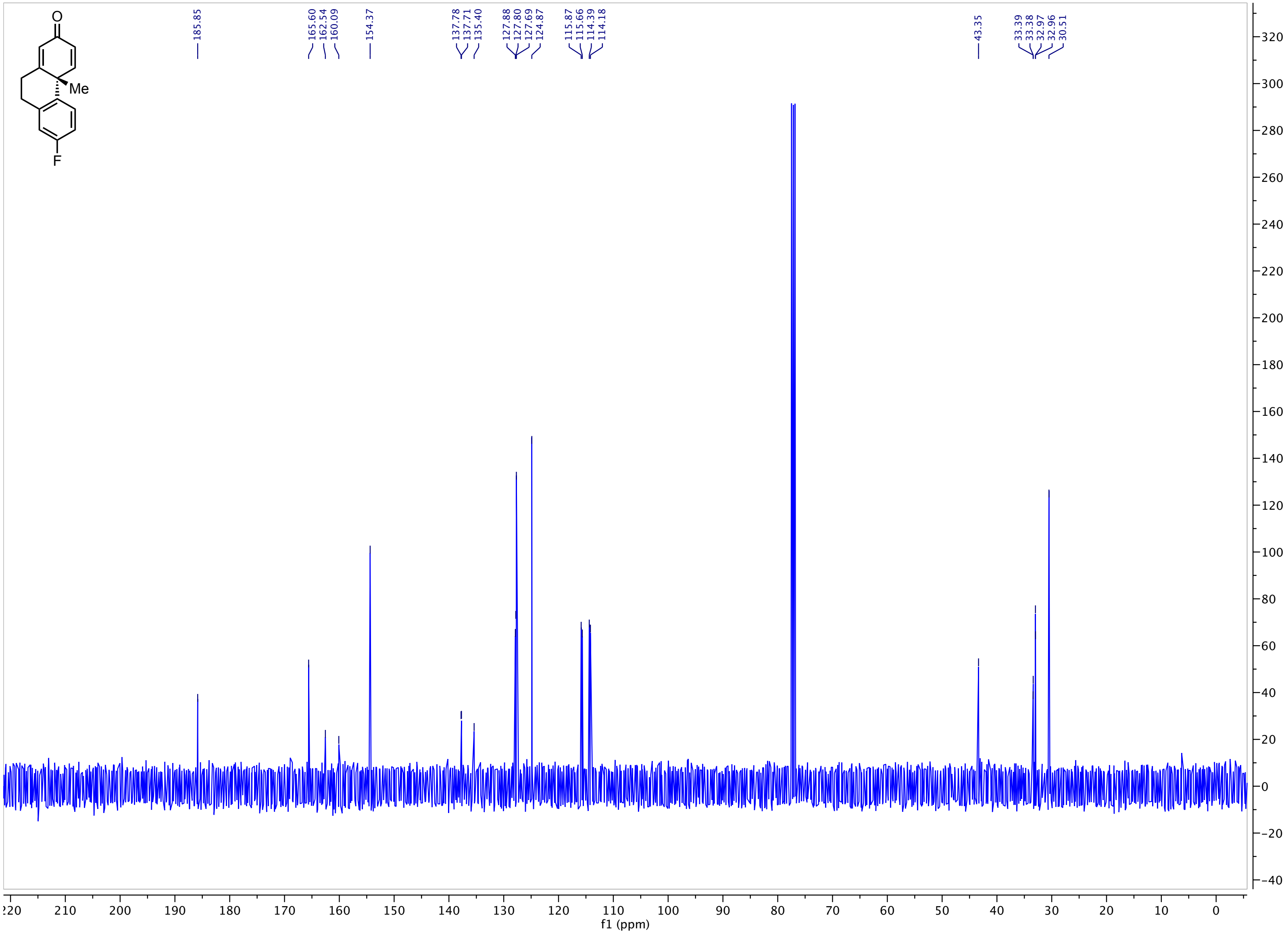

^{19}F NMR (CDCl$_3$): (*S*)-7-Fluoro-4a-methyl-9,10-dihydrophenanthren-2(4a*H*)-one **(7d)**

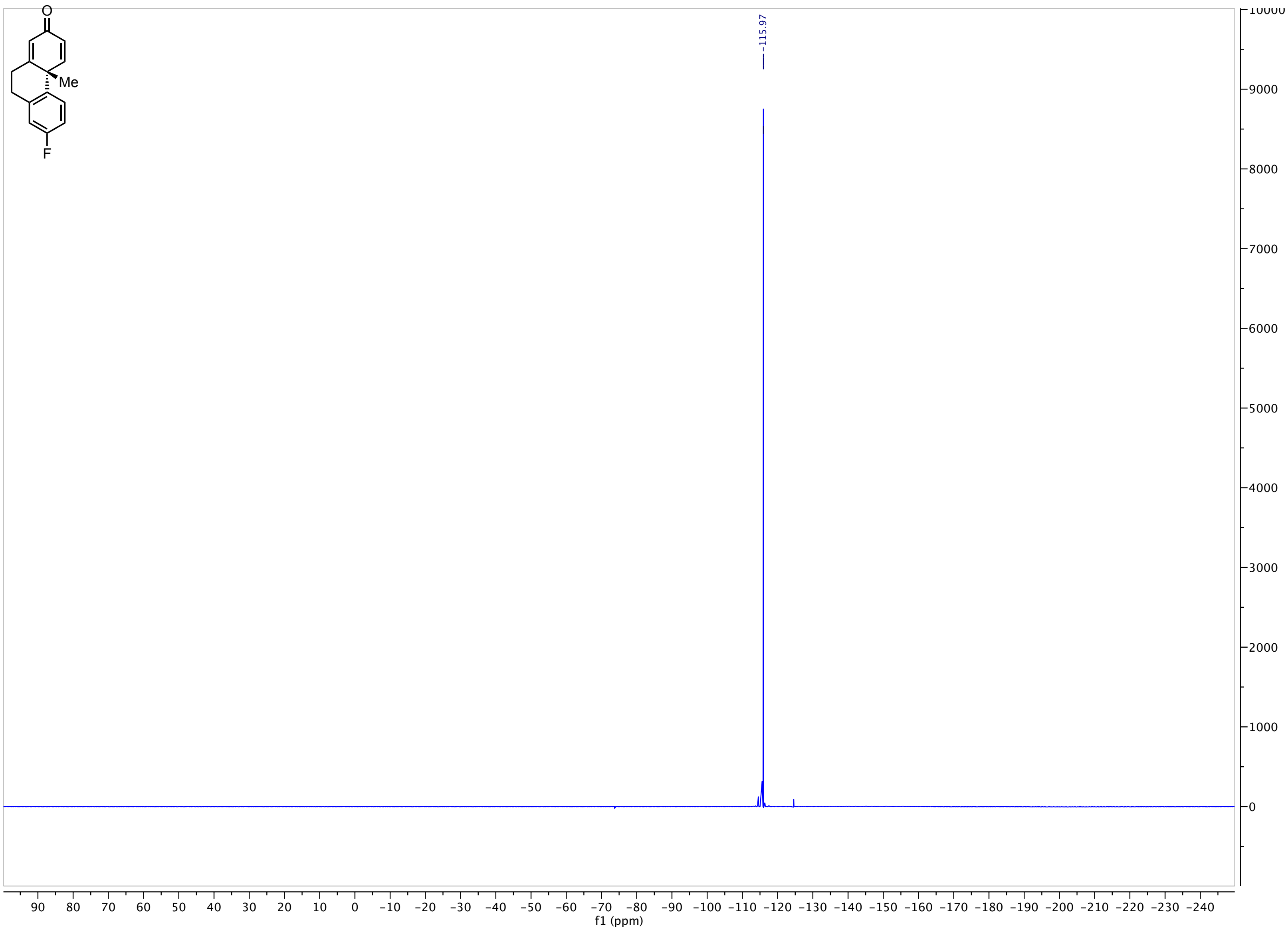

^{1}**H NMR** ($CDCl_3$): (*S*)-9,9,11b-Trimethyl-6,11b-dihydrophenanthro[2,3-*d*][1,3]dioxol-3(5*H*)-one **(7e)**

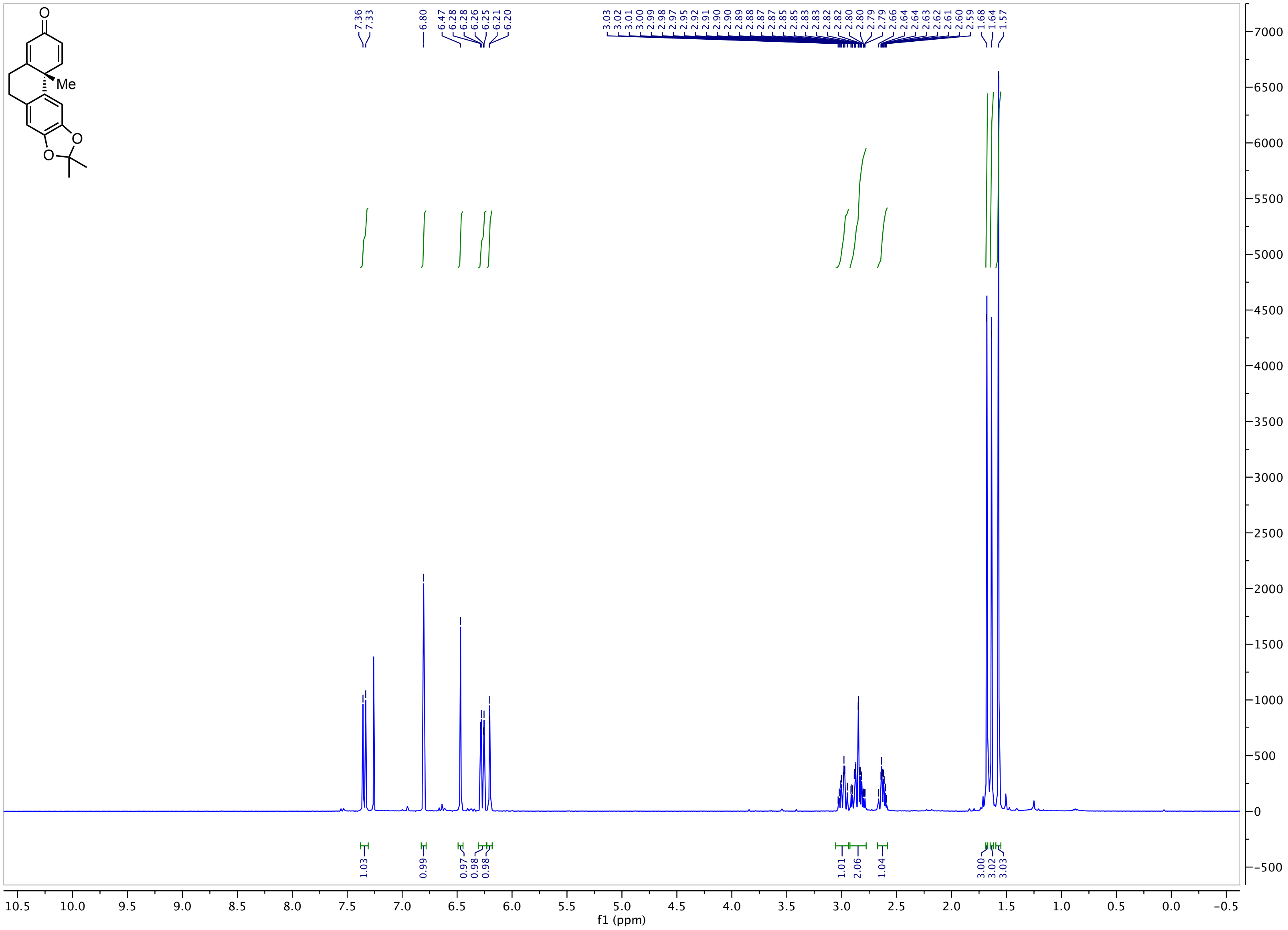

^{13}C NMR ($CDCl_3$): (*S*)-9,9,11b-Trimethyl-6,11b-dihydrophenanthro[2,3-*d*][1,3]dioxol-3(5*H*)-one **(7e)**

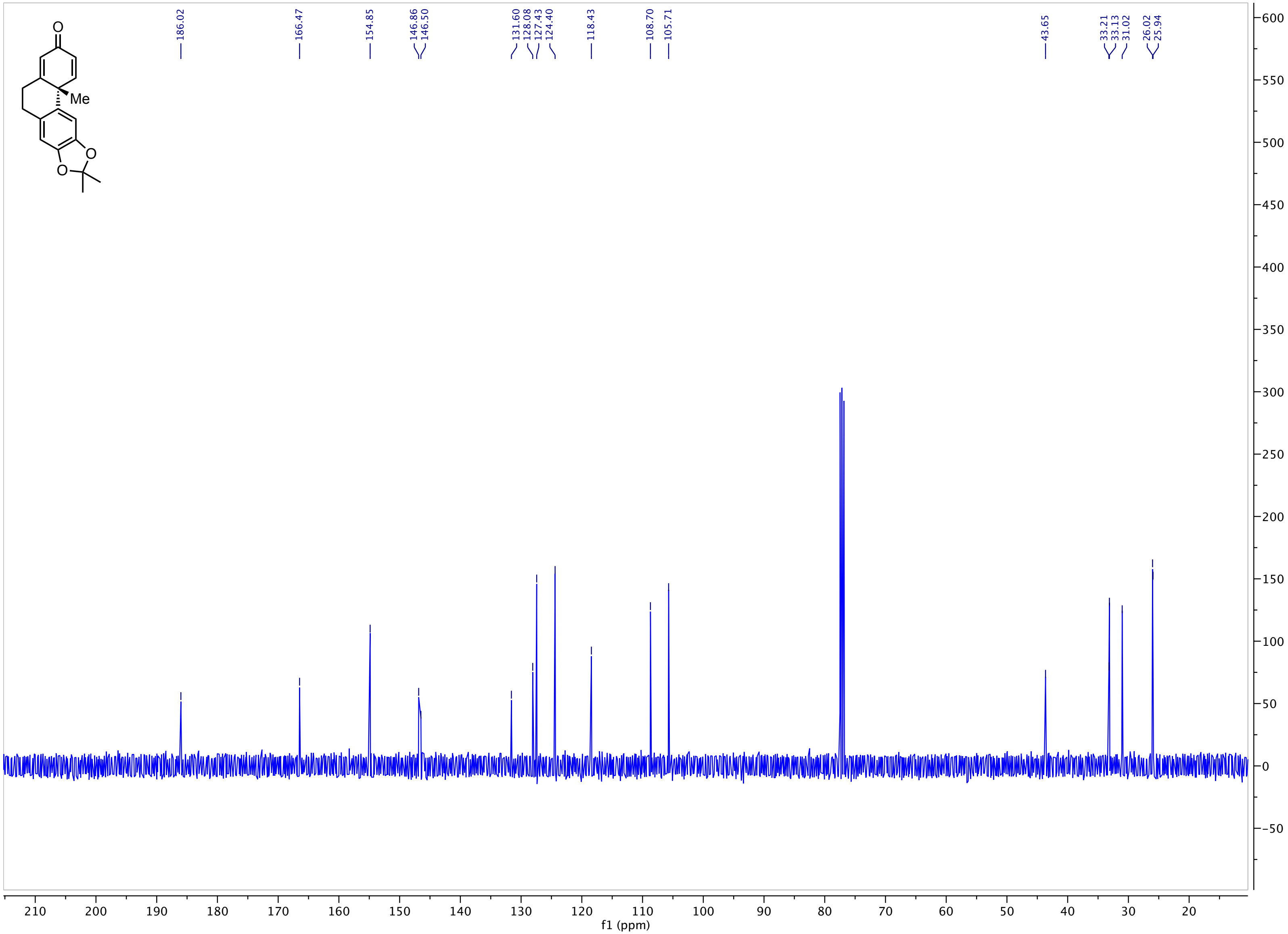

^{1}H NMR ($CDCl_3$): (*R*)-2-Methyl-3'*H*-spiro[cyclohexane-1,1'-isobenzofuran]-2,5-dien-4-one **(9a)**

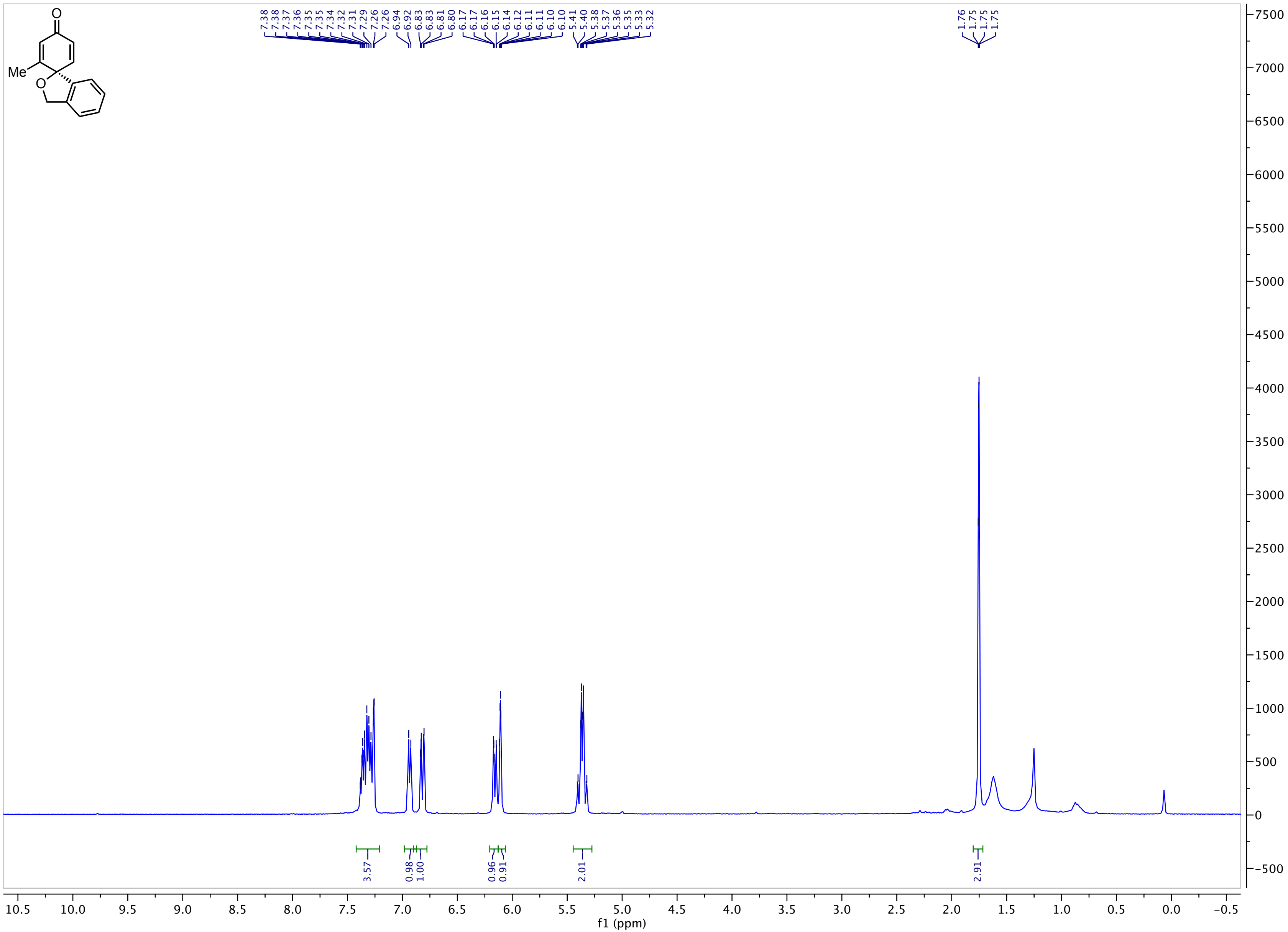

^{13}C NMR ($CDCl_3$): (*R*)-2-Methyl-3'*H*-spiro[cyclohexane-1,1'-isobenzofuran]-2,5-dien-4-one **(9a)**

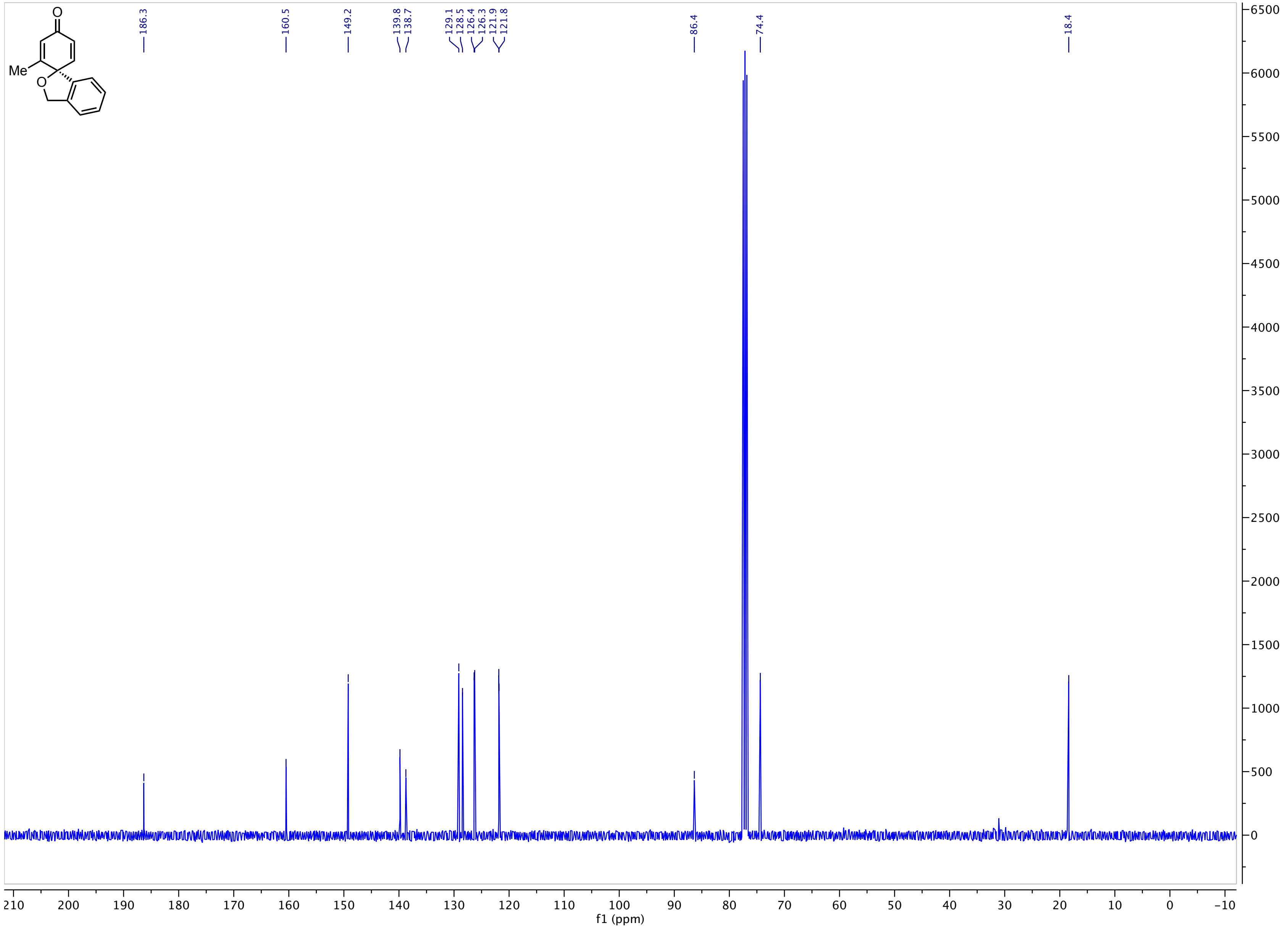

^{1}H NMR ($CDCl_3$): (*S*)-2-Methoxy-3'*H*-spiro[cyclohexane-1,1'-isobenzofuran]-2,5-dien-4-one **(9b)**

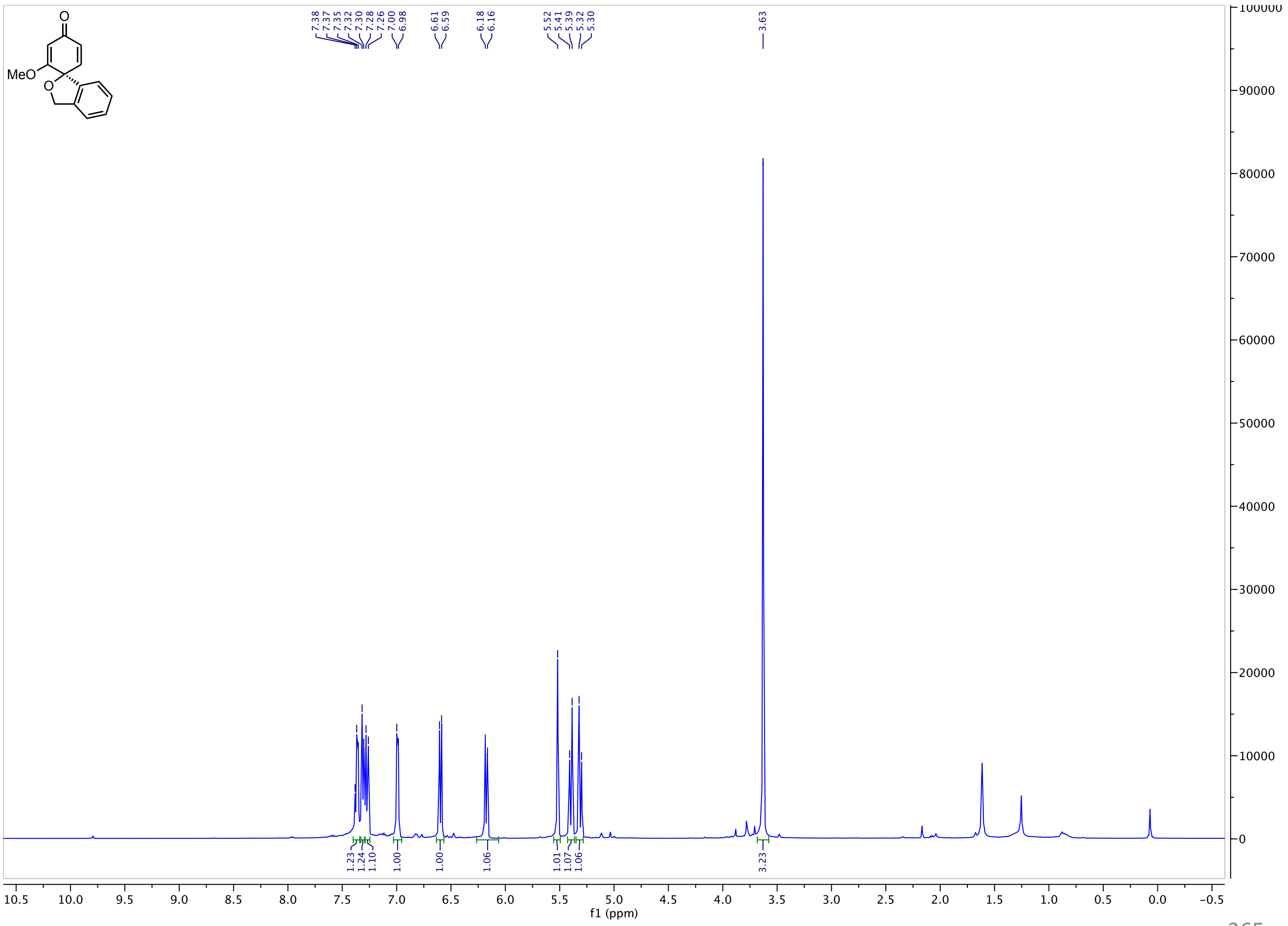

^{13}C NMR ($CDCl_3$): (*S*)-2-Methoxy-3'*H*-spiro[cyclohexane-1,1'-isobenzofuran]-2,5-dien-4-one **(9b)**

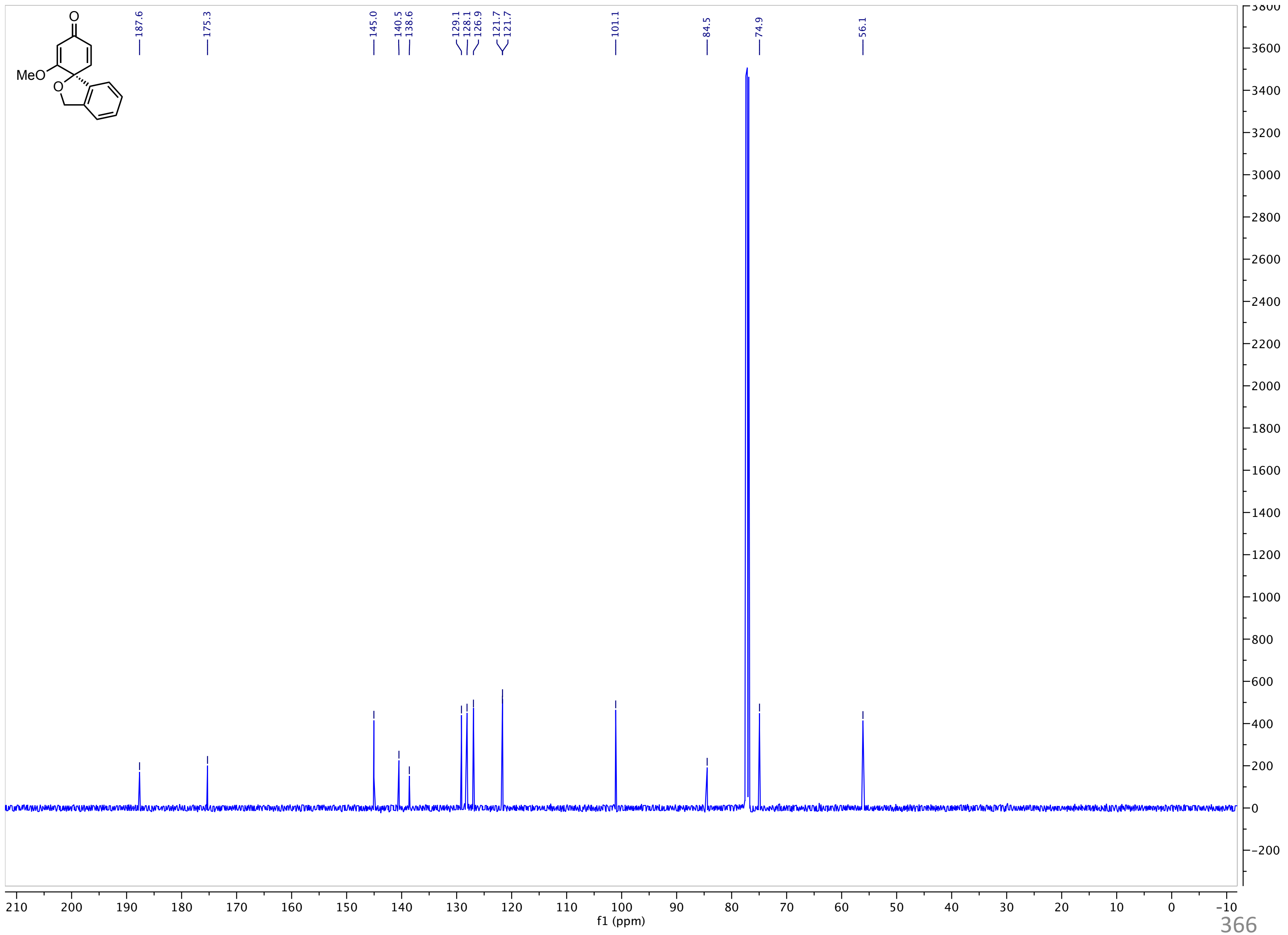

^{1}H NMR ($CDCl_3$): (*R*)-4'-Chloro-2,3-dimethyl-3'*H*-spiro[cyclohexane-1,1'-isobenzofuran]-2,5-dien-4-one **(9c)**

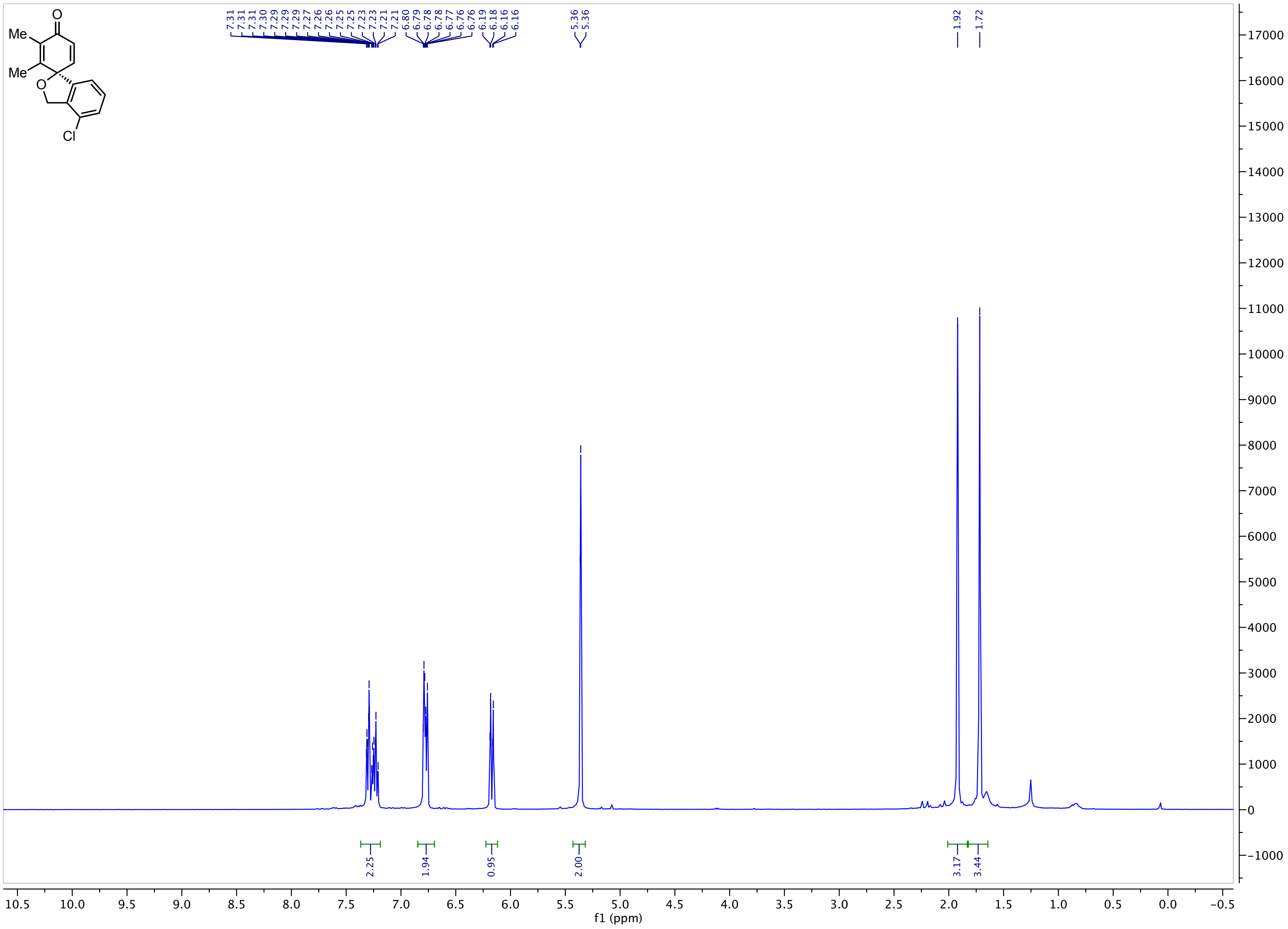

^{13}C NMR ($CDCl_3$): (R)-4'-Chloro-2,3-dimethyl-3'H-spiro[cyclohexane-1,1'-isobenzofuran]-2,5-dien-4-one **(9c)**

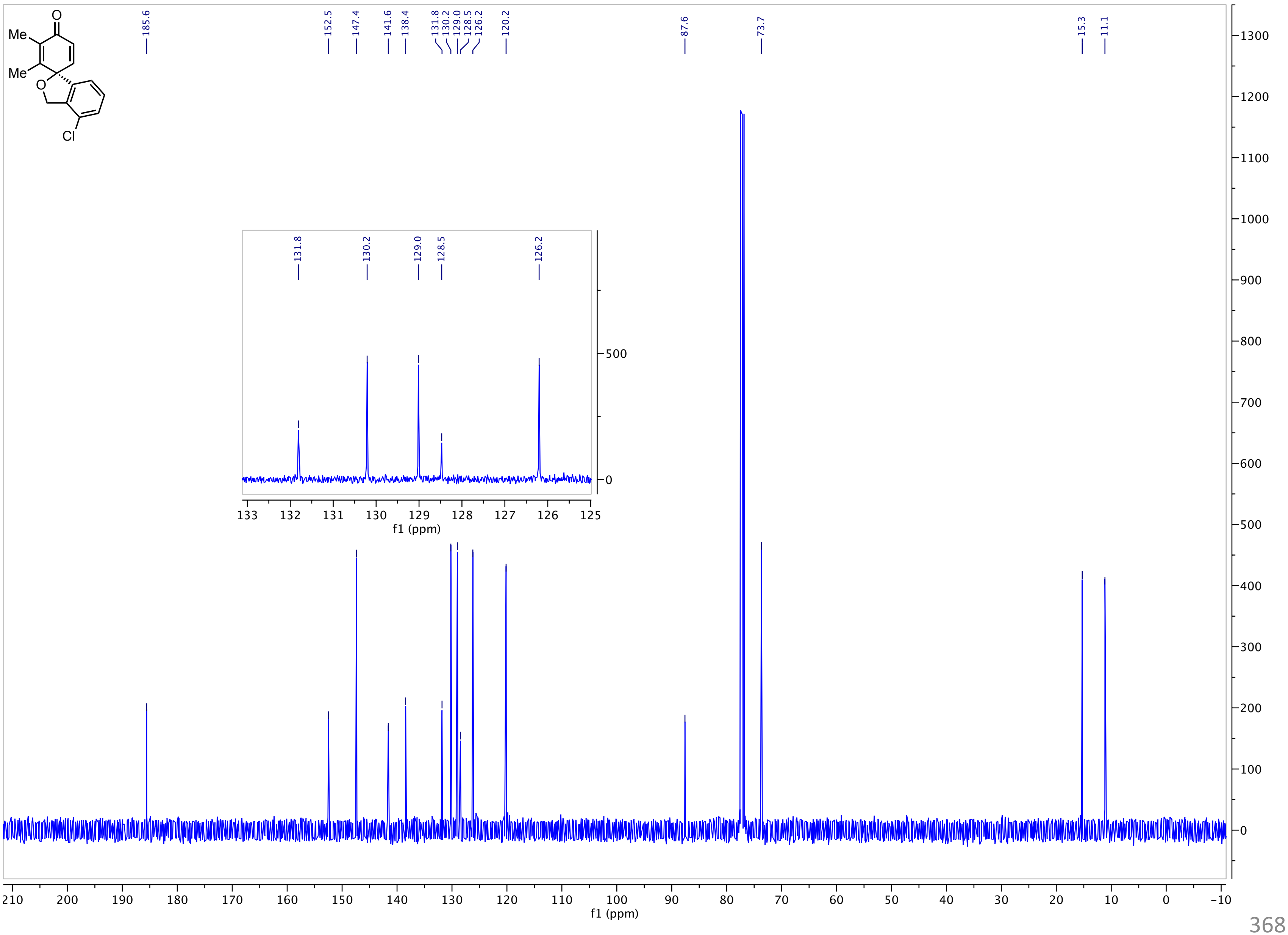

^{1}H NMR ($CDCl_3$): (*R*)-Neopentyl 2'-(dicyclohexylphosphoryl)-2,6-dimethoxy-[1,1'-biphenyl]-3-sulfonate (**S39**)

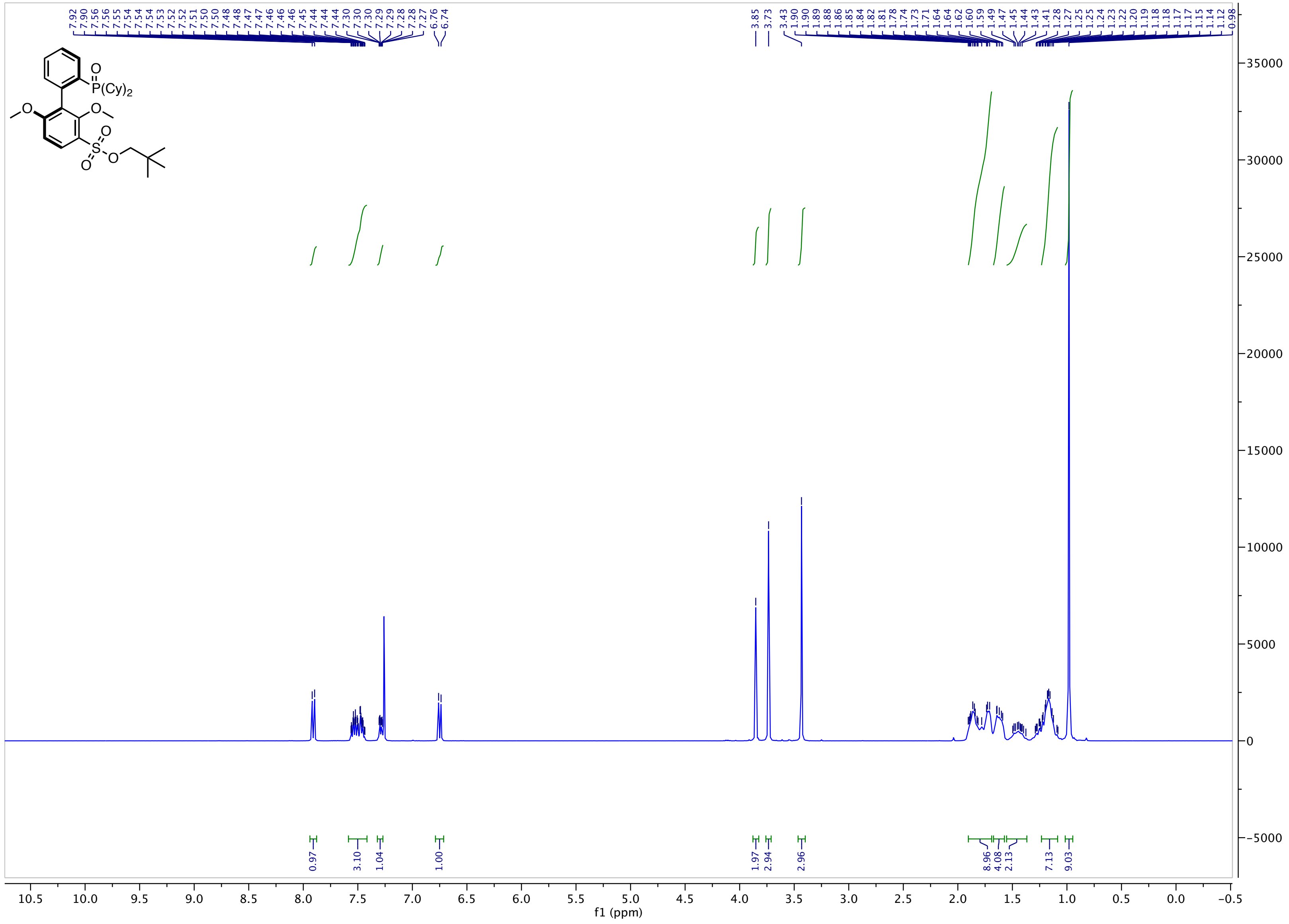

^{13}C **NMR** (CDCl$_3$): (*R*)-Neopentyl 2'-(dicyclohexylphosphoryl)-2,6-dimethoxy-[1,1'-biphenyl]-3-sulfonate (**S39**)

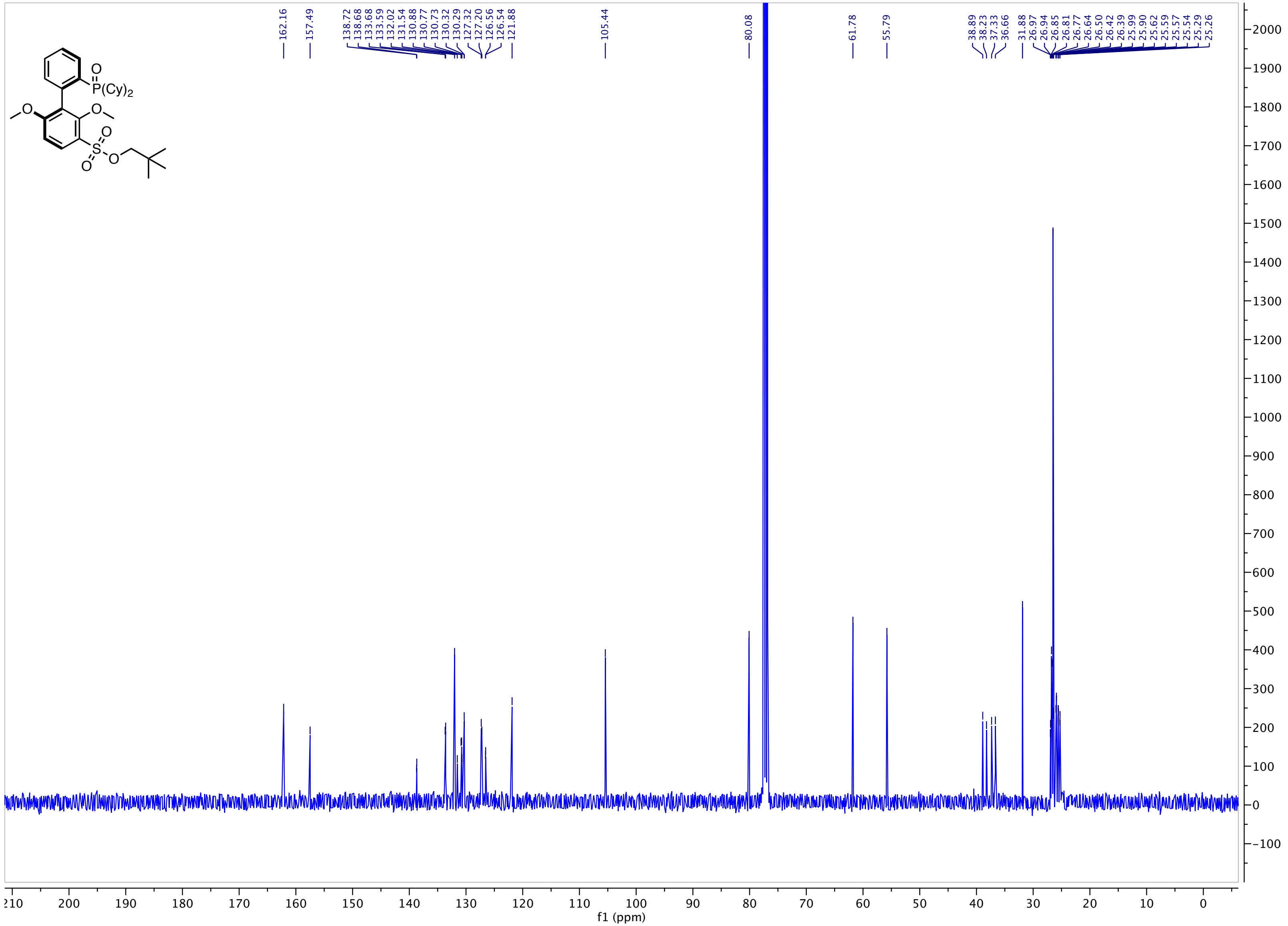

^{31}**P NMR** ($CDCl_3$): (*R*)-Neopentyl 2'-(dicyclohexylphosphoryl)-2,6-dimethoxy-[1,1'-biphenyl]-3-sulfonate (**S39**)

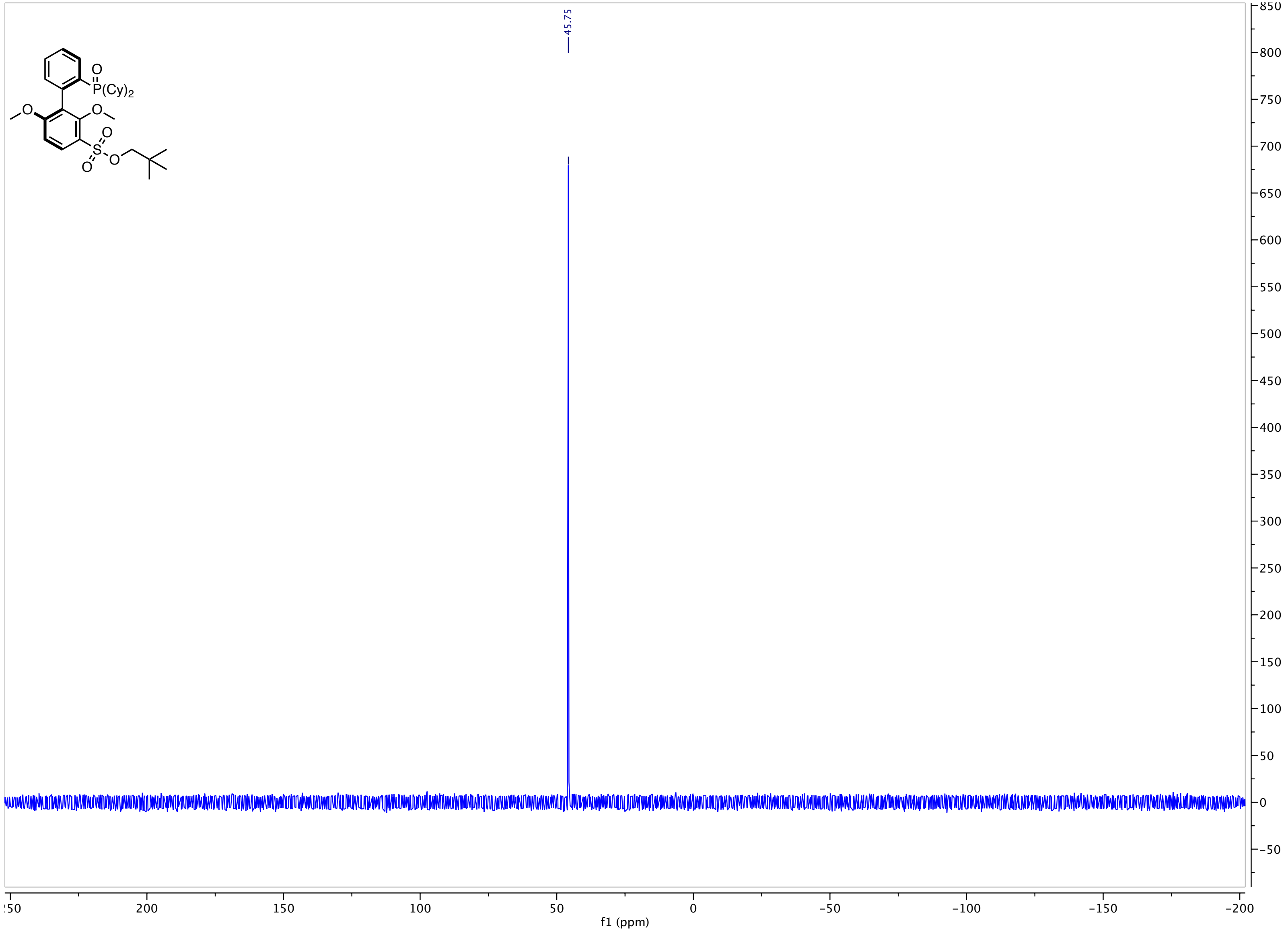

^{1}H NMR ($CDCl_3$): (R)-Neopentyl 2'-(dicyclohexylphosphaneyl)-2,6-dimethoxy-[1,1'-biphenyl]-3-sulfonate **[(R)-sSPhos-Np]**

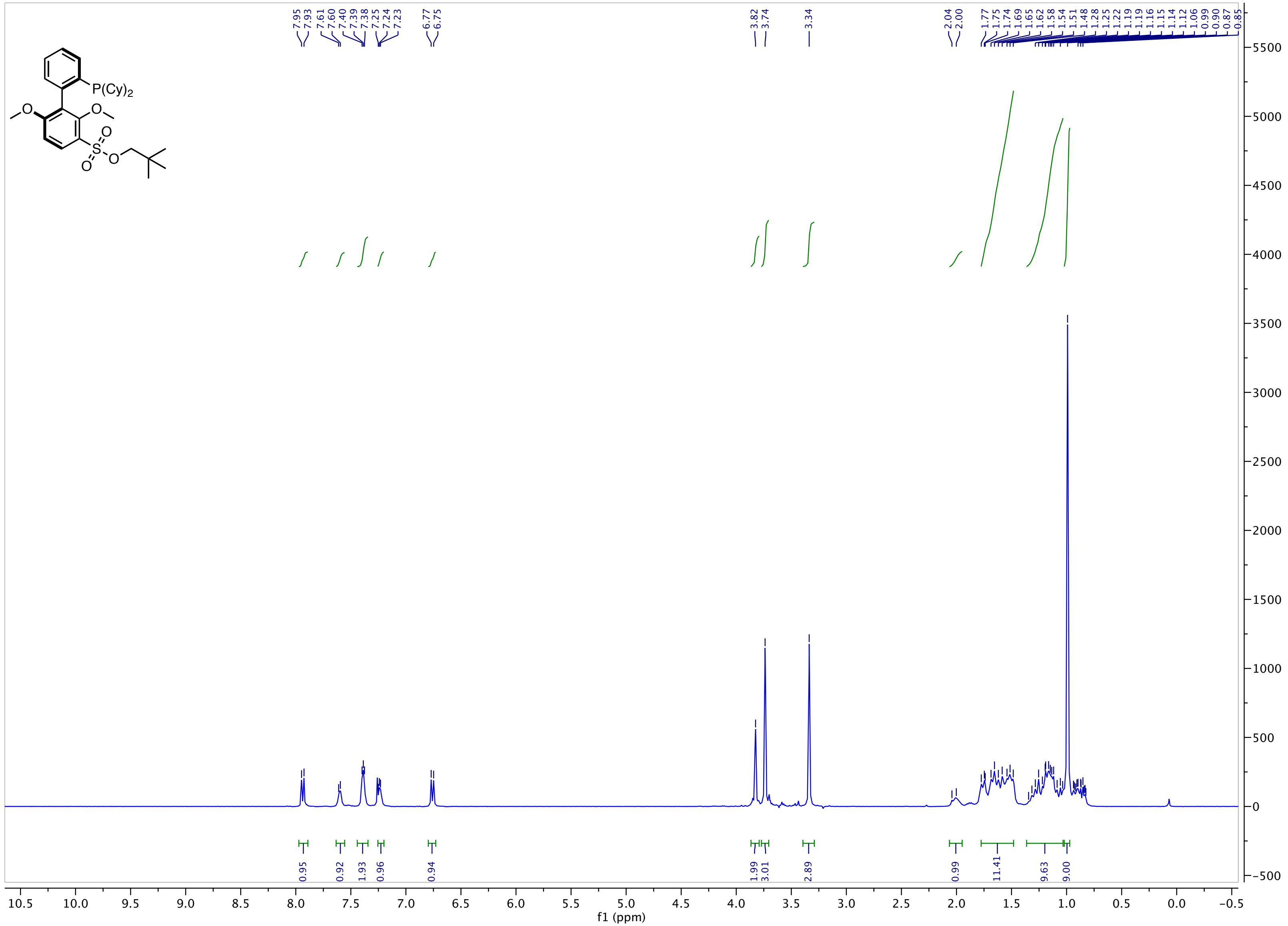

^{13}C NMR ($CDCl_3$): (*R*)-Neopentyl 2'-(dicyclohexylphosphaneyl)-2,6-dimethoxy-[1,1'-biphenyl]-3-sulfonate **[(*R*)-sSPhos-Np]**

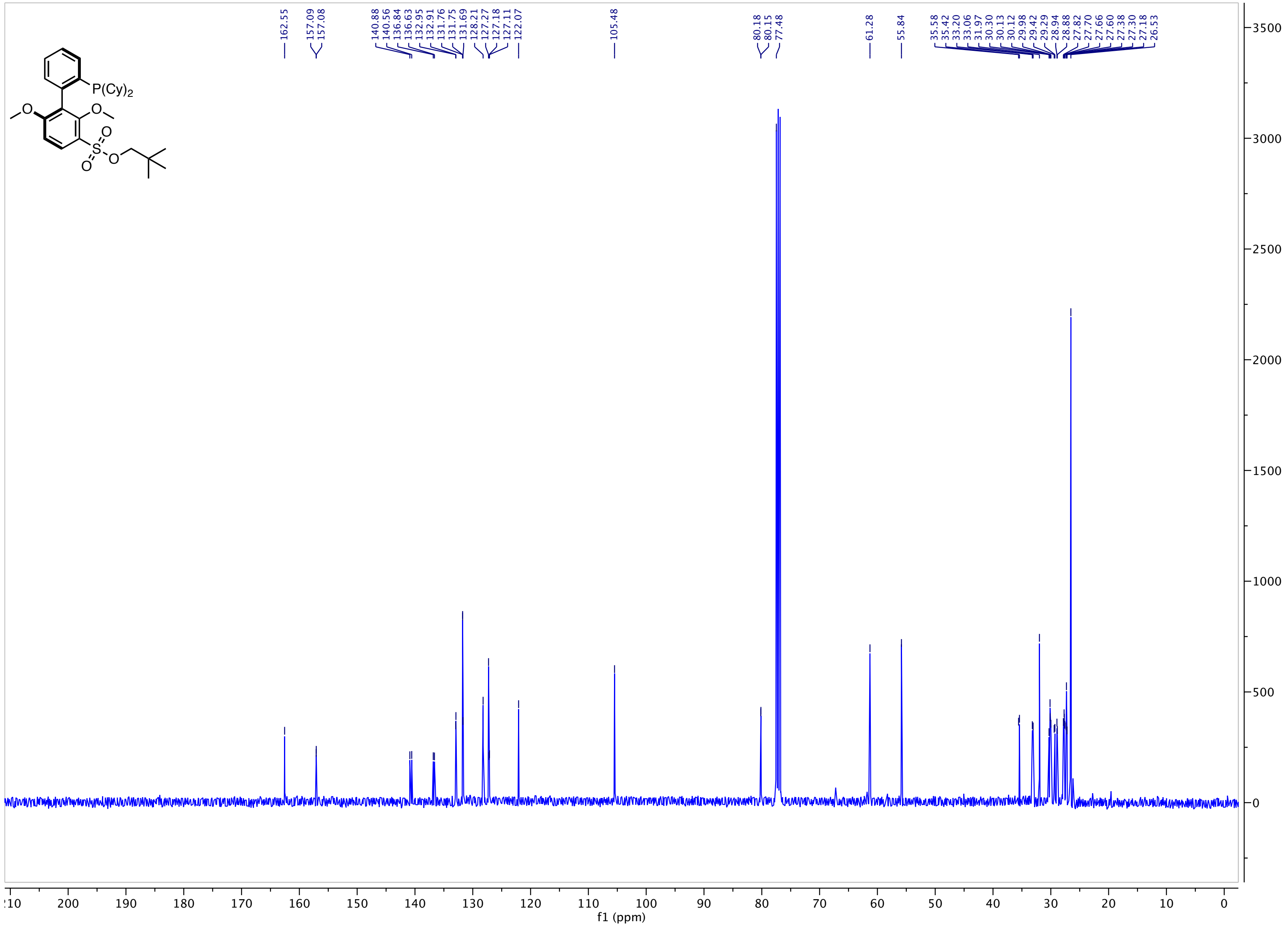

^{31}P NMR (CDCl$_3$): (*R*)-Neopentyl 2'-(dicyclohexylphosphaneyl)-2,6-dimethoxy-[1,1'-biphenyl]-3-sulfonate **[(*R*)-sSPhos-Np]**

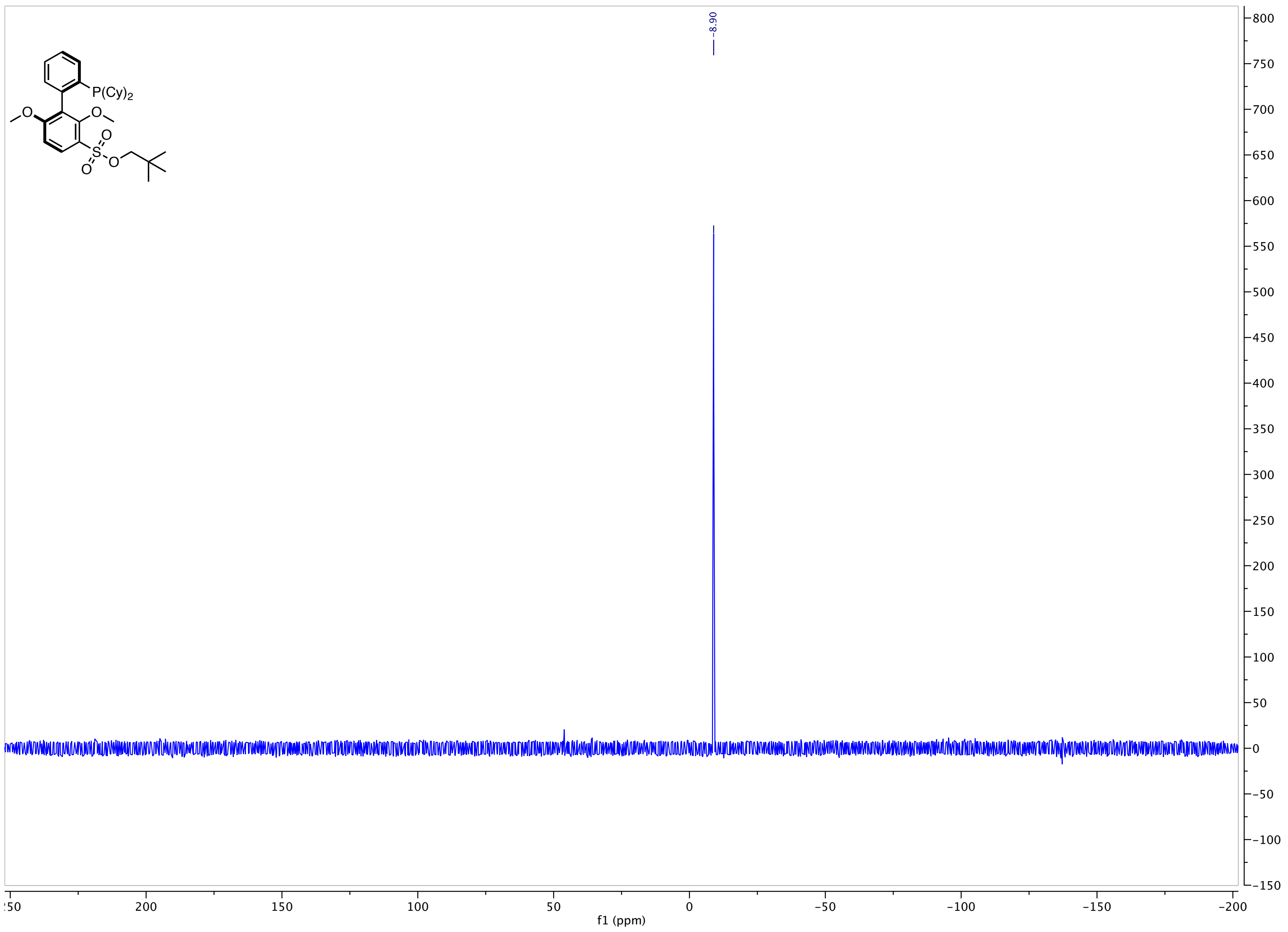

Supplement: Supplementary file 1 — ja3c10663_si_001.pdf [file ja3c10663_si_001.pdf]
